# The Radical Chemistry of 2,3‐Diguanidinopyridines: Coordination and Visible‐Light Photochemistry

**DOI:** 10.1002/chem.71582

**Published:** 2026-08-18

**Authors:** Hanna Koepcke, Etienne Engels, Elisabeth Kaifer, Hans‐Jörg Himmel

**Affiliations:** ^1^ Anorganisch‐Chemisches Institut Ruprecht‐Karls‐Universität Heidelberg Heidelberg Germany

**Keywords:** chemical reaction, chemistry, electron donor, homolysis, ligand, organic synthesis, photochemistry, pyridine, radical, redox

## Abstract

Guanidino‐functionalized aromatics (GFAs) comprise an important class of redox‐active organic molecules and ligands with a variety of applications. In this work, we report the synthesis and chemistry of novel GFAs with a pyridine core functionalized in 2 and 3 positions with two guanidino groups. The two guanidino groups turn the pyridine derivative into an electron donor that can be oxidized in two potentially separated one‐electron redox steps to the dicationic redox state. The redox properties and the stability of the radical monocationic redox state are tuned by the addition of further substituents at the pyridine core. The new redox‐active molecules are applied as ligands in mononuclear metal complexes, leading to significant anodic shifts of the ligand redox potentials. Depending on the substitution pattern, reaction with benzylbromide leads to benzylation at the pyridine N atom or the guanidino N atom. The pyridine *N*‐benzylated molecules are photoactive and undergo chemical reactions, initiated by visible‐light induced homolytic cleavage of the N─C bond with formation of radical monocationic 2,3‐diguanidinopyridine and benzyl radicals, leading to photoconversion to the higher‐energetic, photostable guanidino‐benzylated molecules.

## Graphical abstract

Substitution at the pyridine core of 2,3‐diguanidinopyridines was used to develop novel redox‐active molecules with three interconvertible redox states. They are applied as redox‐active ligands in coordination chemistry. Visible‐light irradiation of the pyridine *N*‐benzylated molecules leads to photoconversion to a higher‐energetic isomer.

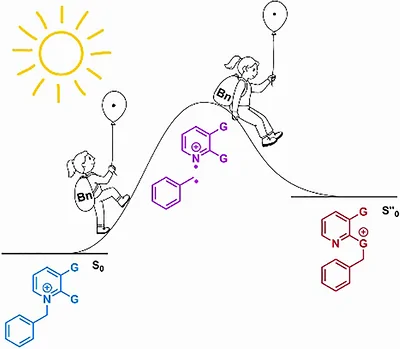

## 1 Introduction

Redox‐active organic molecules are important for a variety of applications [1, 2, 3, 4, 5, 6, 7, 8, 9, 10]. They are used, for example, in energy‐storage devices (e.g. batteries), opto‐electronic devices, and as redox‐active ligands in coordination chemistry.

Over the last two decades, we developed redox‐active guanidines to a powerful class of redox‐active organic molecules [11]. Redox‐active guanidines can be subdivided into two subclasses, namely guanidino‐functionalized aromatics (GFAs) and redox‐active (urea) azines (RAAs). Some time ago, we synthesized the GFA compound 2,3,5,6‐tetrakis(tetramethylguanidino)pyridine [12], denoted L1 in the following (Figure 1). L1 is a two‐electron donor. Two electrons are removed at similar potential in cyclic voltammetry (CV) experiments (*E*_1/2_ = −0.70 V vs. ferrocenium/ferrocene in CH_2_Cl_2_).

**FIGURE 1:**
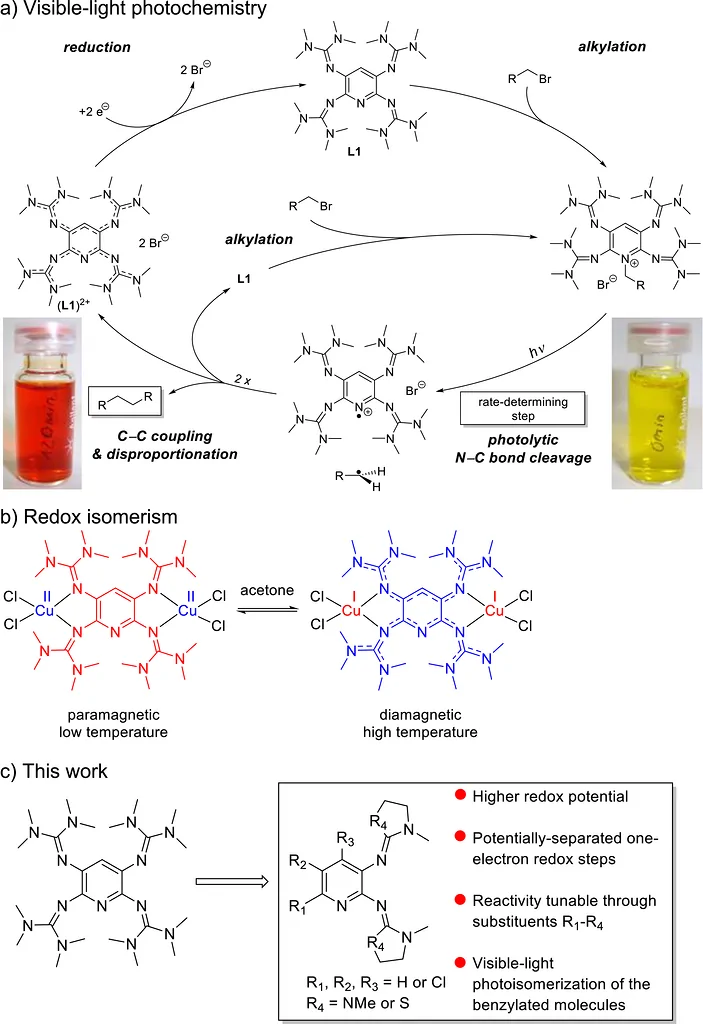
Examples for the chemistry of the 2,3,5,6‐tetraguanidinopyridine L1. (a) Photoinduced C─C coupling reactions. (b) Redox isomerism of dinuclear copper complexes. (c) Scope of this work on novel 2,3‐diguanidinopyridines.

L1 displays an interesting photoreactivity and coordination chemistry [12, 13, 14, 15]. Reaction with benzylbromides leads to benzylation at the pyridine N atom. The resulting monocation turned out to be photochemically active (Figure 1a). Visible‐light irradiation induced homolytic N─C bond cleavage and the formation of benzyl radicals, which couple to form 1,2‐diphenylethanes. Photoexcitation can be described as a GFA‐centered π‐π* transition. This excitation provides enough energy to break homolytically the weakest bond in the benzylated GFA, the C─N bond between the pyridine N atom and the benzyl group. Cross‐coupling reactions to give unsymmetrical 1,2‐diphenylethane derivatives were also developed [14]. The second product of the photoreaction, the radical monocationic GFA, instantly disproportionates to give the dicationic and the neutral redox state. For the C─C coupling reaction, the rapid disproportionation is essential, since it prohibits the attack of the benzyl radical on the GFA. Hence, the neutral, reduced redox state is eventually converted into the product of two‐electron oxidation, the oxidized, dicationic redox state.

With L1 as bridging ligand, the first dinuclear copper complexes were synthesized showing temperature‐dependent quantitative redox isomerism in solution (Figure 1b) [15, 16]. In acetone, the diamagnetic dinuclear Cu^I^ complex with a dicationic GFA ligand is in a fully reversible thermal equilibrium with the paramagnetic dinuclear Cu^II^ complex with a neutral GFA ligand. The GFA reversibly changes its redox state from neutral to dicationic. The radical monocationic redox state is not observed.

Herein, we study the redox and coordination chemistry of 2,3‐diguanidinopyridines as well as the photochemistry of the benzylated compounds. The first 2,3‐diguanidino‐pyridine molecules were synthesized that can be reversibly oxidized in two one‐electron steps to the radical monocationic and dicationic redox states. Visible light irradiation of the benzylated molecules induces endergonic photoisomerization from the pyridine *N*‐benzylated to the higher‐energetic guanidino‐benzylated molecules.

## 2 Results and Discussion

### 2.1 Ligand Synthesis and Characterization

Figure 2 shows the structures of the seven new 2,3‐diguanidinopyridines and the 2,3‐dithioguanidinopyridine that were synthesized in this work. All ligands were obtained from the corresponding diamino precursors (Table 1), which were reacted with 2‐chloro‐1,1,3,3‐tetramethylformamidinium chloride (synthesis of L2b), 2‐chloro‐3‐methyl‐4,5‐dihydrothiazolium chloride (synthesis of L5b) or 2‐chloro‐1,3‐dimethylimidazolinium chloride (synthesis of the other compounds). The new diguanidines/dithioguanidine were obtained in yields ranging from 28% to 80% (Table 1).

**FIGURE 2:**
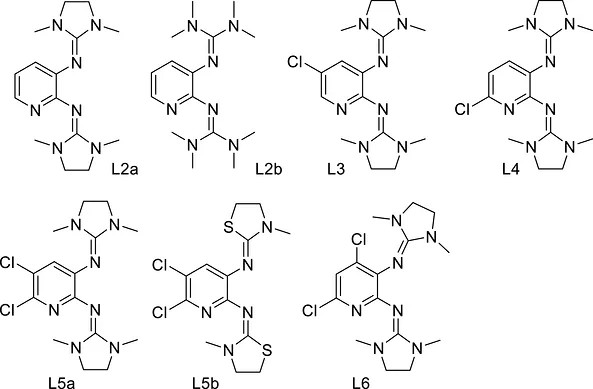
Structures and labeling of the new 2,3‐diguanidino‐pyridine derivatives synthesized in this work.

**TABLE 1:** Synthesis of the seven new 2,3‐diguanidinopyridine molecules.

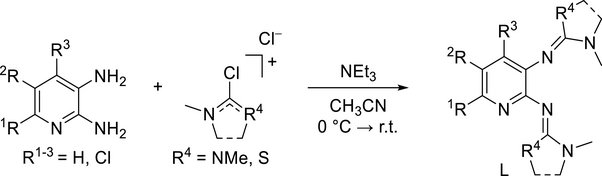						
L	R^1^	R^2^	R^3^	R^4^	Reaction time	Yield (%)
L2a	H	H	H	NMe	66 h	62
L2b	H	H	H	NMe	66 h	29
L3	H	Cl	H	NMe	18 h	52
L4	Cl	H	H	NMe	32 h	30
L5a	Cl	Cl	H	NMe	18 h	80
L5b	Cl	Cl	H	S	64 h	28
L6	Cl	H	Cl	NMe	32 h	38

Crystal structures were obtained for six of the seven new compounds (L2a, L3, L4, L5a, L5b, and L6, Figure 3). Some structural data are collected in the table imbedded in Figure 3. The imino N═C double bond lengths fall into a typical range for GFA compounds [11]. For all compounds, the C─N bonds connecting the pyridine ring with the guanidino group (bonds a and c) are significantly longer than the imino C═N bonds in the guanidino groups (b and e). It will be shown below that coordination, protonation and alkylation lead to significant changes in these bond lengths. As expected, the guanidino CN_3_ planes or thioguanidino CN_2_S planes are generally highly tilted with respect to the pyridine ring plane. This conformation arises from a mixture of electronic and steric effects (see the discussion in Ref. [17]).

**FIGURE 3:**
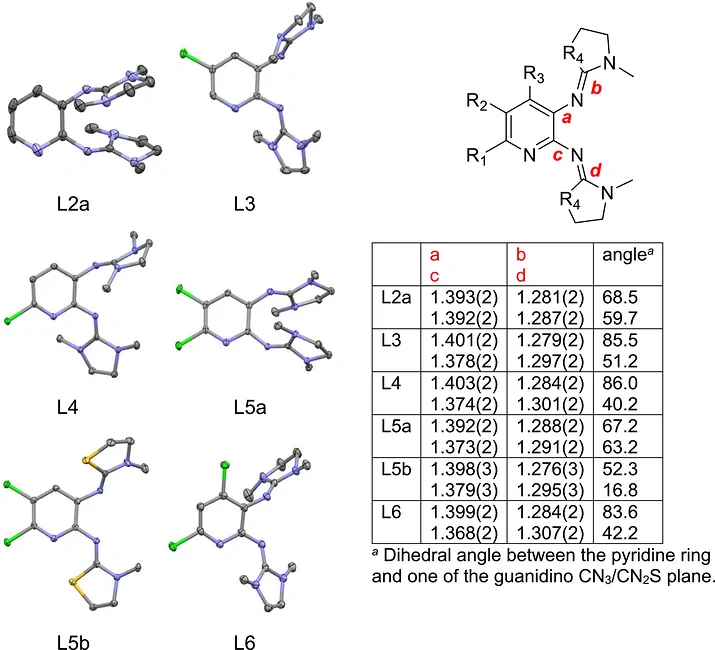
Structures of the new 2,3‐diguanidino‐substituted pyridines in the solid state. Hydrogen atoms omitted. Displacement ellipsoids drawn at the 50% probability level. Color code: N light blue, C grey, Cl green, S yellow. A table with selected structural data (bond lengths in Å, angles in °) is included.

CV measurements were carried out for all seven new compounds in CH_2_Cl_2_ solutions. In these experiments, a glassy carbon working electrode, together with an Ag/AgCl reference electrode and a Pt wire counter electrode, was used, and *n*Bu_4_NPF_6_ (0.1 M) was added as supporting electrolyte to the solution. Generally, the curves shown in this work were recorded with 20 mV s^−1^ scan speed. The potentials are given with respect to the reference redox couple ferrocenium/ferrocene (Fc^+^/Fc). In the case of L4, no clear oxidation wave was visible. Clear oxidation waves were observed for L2a, L2b, L3, and L5b, but they belong to irreversible oxidation processes. As expected, the potentials measured for the diguanidinopyridine derivatives are all significantly higher than that of the tetraguanidinopyridine L1 (*E*_1/2_ = −0.70 V, *E*_ox_ = −0.60 V). Compound L5b, with two thioguanidino groups, exhibits the highest oxidation potential (*E*_ox_ = 0.53 V), in line with previous work showing that thioguanidino groups lead to higher redox potentials than guanidino groups [18]. The lowest oxidation potentials (*E*_ox_ = −0.08 and −0.05 V) were measured for L2a and L2b (Table 2).

**TABLE 2:** Potentials (*E*_ox_ and *E*_1/2_ values in V vs. Fc^+^/Fc) for the redox steps of L1‐L6 in CH_2_Cl_2_ solutions.

Compounds	1^st^ redox step *E*_ox_ (*E*_1/2_)	2^nd^ redox step *E*_ox_ (*E*_1/2_)
L1	−0.60 (−0.70)^a^	−0.60 (−0.70)^a^
L2a	−0.08	—
L2b	−0.05	—
L3	−0.04	—
L5a	0.07 (−0.04)	0.36 (0.28)
L5b	0.53	0.65
L6	0.05 (−0.03)	0.40 (0.33)

^a^Ref. [12].

Two of the 2,3‐diguanidinopyridines, namely L5a and L6, show reversible one‐electron redox processes in the CV measurements (Figure 4). For L5a, with two chlorido substituents in 5 and 6 positions, potentials of *E*_1/2_ = −0.04 V (*E*_ox_ = 0.07 V) for the redox couple L5a^·+^/L5a, and *E*_1/2_ = 0.28 V (*E*_ox_ = 0.36 V) for the redox couple L5a^2+^/L5a^·+^ were found. For L6 with two chlorido‐substituents in 4 and 6 positions, the experiments yielded *E*_1/2_ = −0.03 V (*E*_ox_ = 0.05 V) and *E*_1/2_ = 0.33 V (*E*_ox_ = 0.40 V). As expected, these values are significantly higher than the two‐electron redox step at *E*_1/2_ = −0.70 V observed for L1 (included in Figure 4). Most importantly, L1 shows a two‐electron redox event, while L5a and L6 show two potentially separated one‐electron redox events. The stability of the radical monocations L5a**^·^** ^+^ and L6**^·^** ^+^ toward disproportionation to the neutral and dicationic redox states can be estimated from the potential difference between the two redox steps. With a Δ*G* ^0^ value for disproportionation of Δ*G* ^0^ ≈ F·Δ*E*_1/2_ and Δ*E*_1/2_ values of 0.32 V for L5a and 0.36 V for L6, one obtains Δ*G* ^0^ = 31 kJ mol^−1^ (*K*_c_ = 3.71·10^−6^) for disproportionation of L5a**^·^** ^+^, and 35 kJ mol^−1^ (*K*_c_ = 7.38·10^−7^) for disproportionation of L6**^·^** ^+^. These values underline the stability of the radical cations.

**FIGURE 4:**
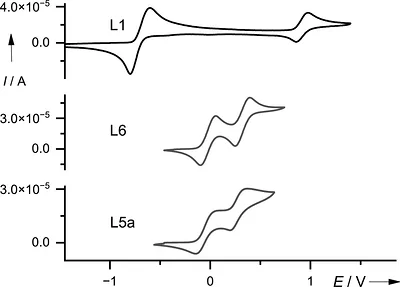
Comparison between the CV curves for L1, L5a and L6 in CH_2_Cl_2_ solutions. Potentials given versus the reference redox couple Fc^+^/Fc.

The two compounds with the lowest oxidation potential, L2a and L2b, were chemically oxidized to obtain information about possible oxidation‐induced follow‐up processes. Chemical oxidation was carried out in the presence or absence of an additional base (Cs_2_CO_3_). The NMR spectra recorded in experiments using two equivalents of FcPF_6_ as oxidizing reagent indicated the formation of a product mixture. Although it was impossible to isolate a larger amount of pure product, crystals suitable for a structural analysis were obtained. The structures gave some information about the oxidation‐induced reactions. In both cases, they arise from nucleophilic attack of the guanidino N atoms from a neutral, reduced L2a/L2b molecule on the pyridine ring of an oxidized molecule, initiating oxidation‐induced dimerization. In the case of L2b, both guanidino groups of L2b attack the ring of the oxidized dication (L2b)^2+^, resulting in a dimer product in which two guanidino groups connect the two pyridine rings (Figure 5a). The two substituted protons migrate to the two terminal guanidino groups, leading to an overall charge of +4. A similar reaction, proceeding selectively in 96% yield, was observed previously for the 1,2‐diguanidinobenzene included in Figure 5a [19]. In the case of L2a, oxidation triggers dimerization, too. However, only one guanidino bridge is installed. Instead of a second guanidino group, the pyridine N atom acts as a nucleophile, leading to a product with two six‐ and one five‐membered rings (Figure 5b). The two dimerization reactions are examples for redox‐induced aromatic substitutions (RIAS), that can be used for selectively functionalizing and connecting GFAs [19, 20, 21]. The new results demonstrate that pyridine ring substitution (in para position to the guanidino groups) is required to block follow‐up (RIAS) reactions of the oxidized redox states.

**FIGURE 5:**
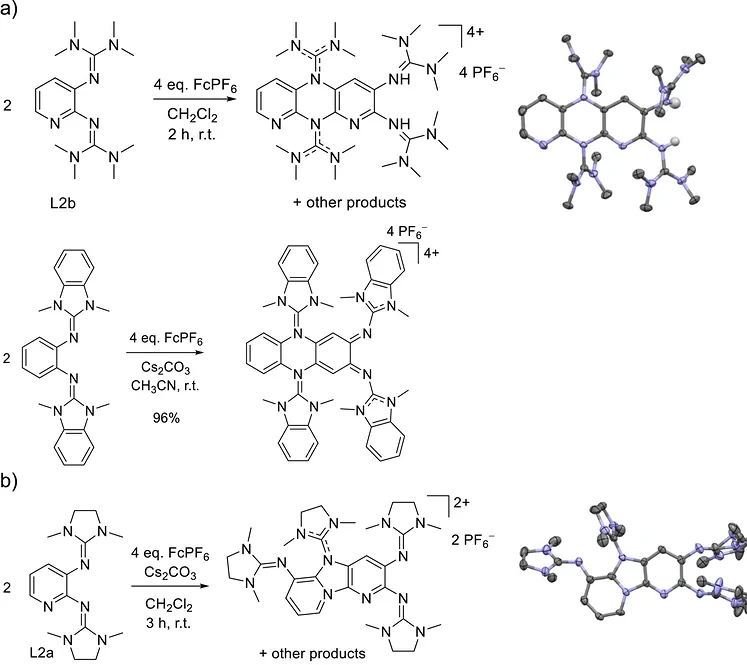
Oxidation of ligands L2b (a) and L2a (b) giving a mixture of products from which two dimerization products crystallized. Unnecessary hydrogen atoms omitted. Displacement ellipsoids drawn at the 50% probability level. Color code: N light blue, C grey, H white.

In line with the CV curves, chemical oxidation of compounds L5a and L6 gives relatively stable oxidation products. In these compounds, the chloro substituents prevent the pyridine ring from being attacked upon oxidation. Since we were particularly interested in the properties of the radical monocationic redox states L5a**^·^** ^+^ and L6**^·^** ^+^, FcPF_6_ was used as an oxidizing reagent. FcPF_6_ was chosen since the potentials for the second one‐electron redox step are already too high to obtain large quantities of the dications with FcPF_6_.

UV‐vis spectra of (L5a)**^·^** ^+^, obtained upon addition of up to 0.75 equivalents of FcPF_6_, showed a broad absorption in the visible region (centered at 646 nm) with shoulders at ca. 595 and 710 nm (Figure 6). Moreover, a band at 445 nm belongs to the monocation (L5a)**^·^** ^+^. Please note that substoichiometric amounts of Fc(PF_6_) were applied to avoid the formation of (L5a)^2+^ due to the disproportionation equilibrium. The UV‐vis spectrum of (L6)**^·^** ^+^, obtained in a similar way, shows a relatively sharp band at 424 nm, and a broad band centered at 631 nm with shoulders at 588 and 688 nm. (L5a)**^·^** ^+^ and (L6)**^·^** ^+^ show relatively broad EPR signals with a *g* value near the free electron *g* factor (spectra embedded in Figure 6), but hyperfine splitting was not resolved. This result is consistent with previous studies on the changes in the EPR spectra with the substitution pattern. For 1,4‐diguanidino‐ and 1,3‐diguanidinobenzene derivatives, clearly resolved hyperfine splitting was observed, but not for 1,2‐diguanidinobenzene derivatives [22].

**FIGURE 6:**
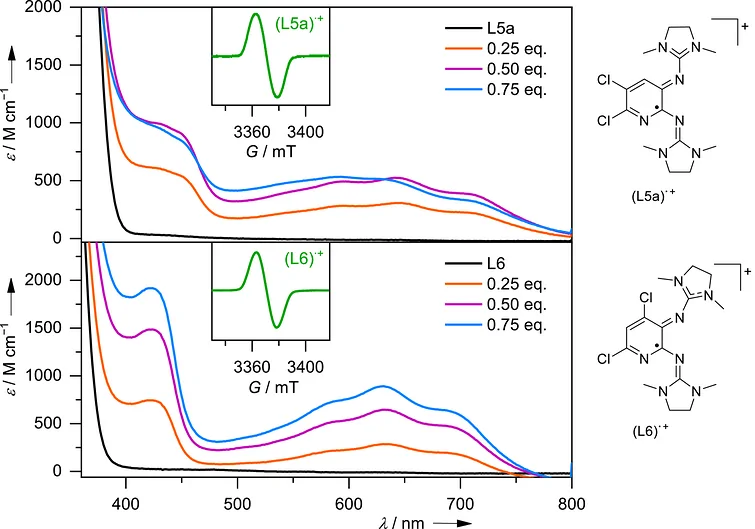
UV‐vis spectra recorded before and after addition of FcPF_6_ to solutions of L5a and L6 in CH_2_Cl_2_ (*c* = 4.75 · 10^−5^ mol L^−1^) at room temperature. EPR spectra (X­band) for (L5a)**^·^** ^+^ and (L6)**^·^** ^+^, formed upon addition of 0.7 eq. FcPF_6_ to a CH_2_Cl_2_ solution of L5a/L6, are embedded.

### 2.2 Coordination Chemistry

Next, we applied the new compounds in coordination chemistry. Experiments with L2a showed that the compounds are versatile ligands, that form strong bonds to metals such as Co^II^, Ni^II^, and Cu^II^ (Scheme 1). All complexes were isolated in high yield (92‐98%), and crystal structures were obtained for six representative complexes (Figure 7). As expected, metal coordination leads to an increase of the imino N═C bond lengths within the guanidino groups. This increase is partially caused by π‐donor contributions to the ligand‐metal bond (see the discussion of this issue in Ref. [23]). A large π‐donor contribution leads to a weak ligand‐field. Therefore, the Cu^II^ complexes adopt not a square‐planar coordination mode, but a coordination mode that is distorted in the direction toward tetrahedral, expressed by the large dihedral angle of 52°. As already observed previously [24], Cu^II^ coordination has a particularly large effect.

**SCHEME 1:**
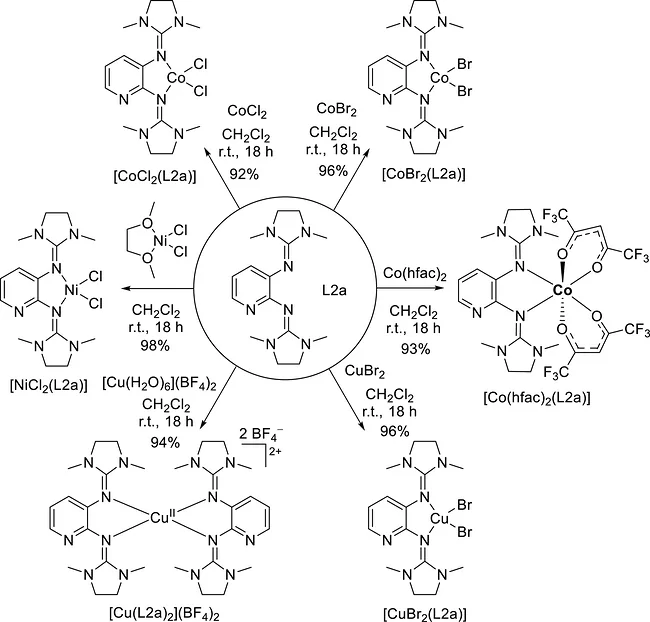
Synthesis of transition metal complexes of L2a.

**FIGURE 7:**
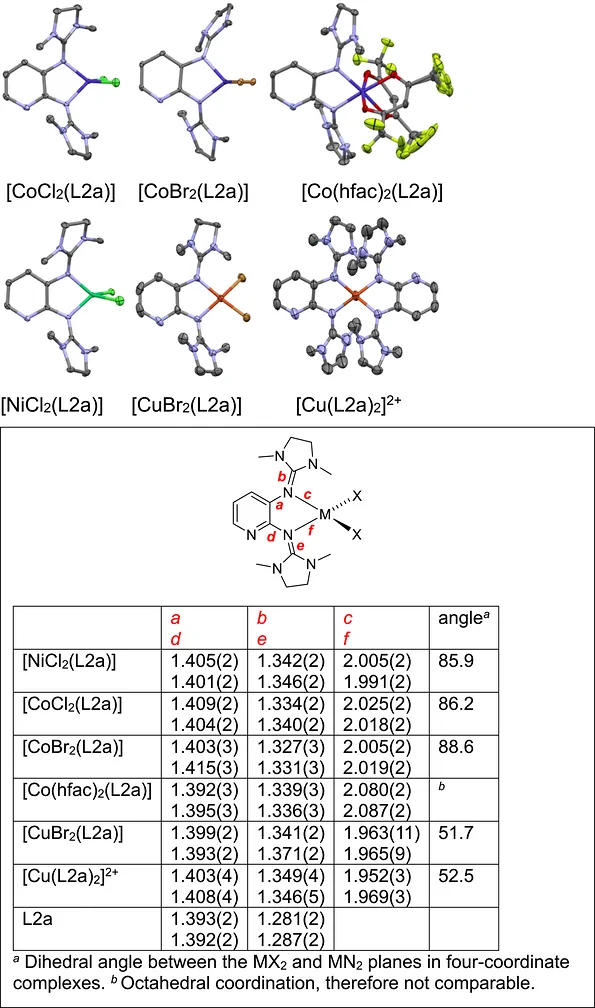
Structures of the complexes of L2a in the solid state. Displacement ellipsoids drawn at the 50% probability level. Hydrogen atoms omitted. Color code: C grey, N light blue, Cl light green, Co dark blue, Ni green, Cu dark orange, Br light orange, F yellow, O red. A table with structural parameters (bond lengths in Å, angles in °) is included.

According to CV measurements, coordination leads to significant anodic shifts of the *E*_ox_ values. Hence, the *E*_ox_ value in free L2a of −0.08 V shifts to 0.14 V for [Co(hfac)_2_(L2a)], 0.25 V for [NiCl_2_(L2a)], 0.76 V for [CoCl_2_(L2a)], and 0.75 V for [CoBr_2_(L2a)]. We also prepared the two complexes [ZnCl_2_(L4)] and [PdCl_2_(L6)] (Figure 8a). In both cases, it was possible to obtain the crystal structures from SC‐XRD measurements. For the complex [PdCl_2_(L6)], two reversible, potentially separated redox processes were measured in CH_2_Cl_2_ solution with CV (Figure 8b), that were assigned to the redox couples [PdCl_2_(L6)]**^·^** ^+^/[PdCl_2_(L6)] (*E*_1/2_ = 0.40 V, *E*_ox_ = 0.45 V) and [PdCl_2_(L6)]^2+^/[PdCl_2_(L6)]**^·^** ^+^ (*E*_1/2_ = 0.94 V, *E*_ox_ = 0.89 V). Hence, the palladium coordination leads to significant anodic shifts of 0.43 V for the first and 0.61 V for the second redox step, with respect to the free ligand L6. The stability of the radical monocationic ligand with respect to disproportionation increases upon coordination. From the potential difference between the first and second one‐electron redox steps, the Δ*G* value for disproportionation was estimated to be 52 kJ mol^−1^ (*K*_c_ = 7.76·10^−10^). This value compares with Δ*G* = 35 kJ mol^−1^ (*K*_c_ = 7.38·10^−7^) for free L6**^·^** ^+^.

**FIGURE 8:**
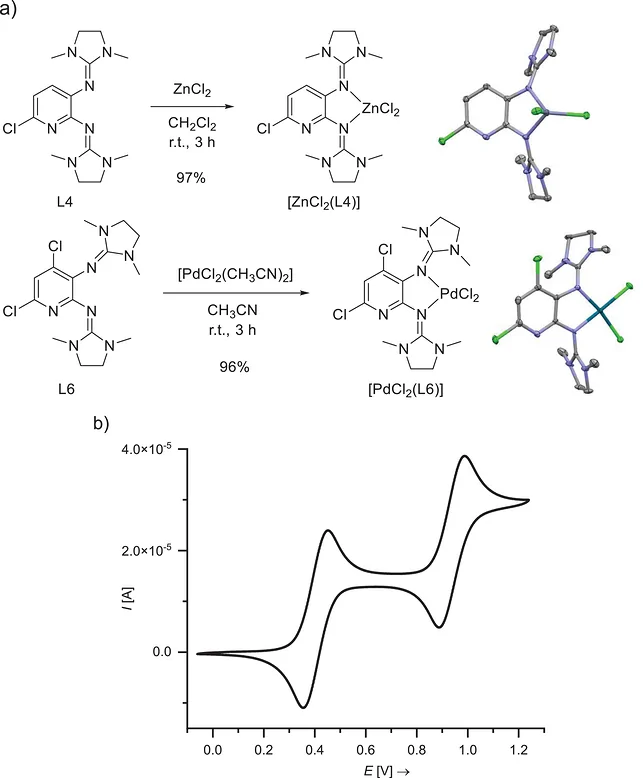
(a) Synthesis of the new complexes [ZnCl_2_(L4)] and [PdCl_2_(L6)]. Illustrations of the structures in the solid state from SC‐XRD measurements are included. Hydrogen atoms omitted. Displacement ellipsoids drawn at the 50% probability level. Color code: N light blue, C grey, Zn dark grey, Pd turquoise, Cl green. (b) CV curve recorded for [PdCl_2_(L6)] in CH_2_Cl_2_ solution referenced to Fc^+^/Fc.

### 2.3 Protonation and Alkylation Experiments

The new compounds exhibit three Lewis‐basic sites, the two imino N atoms of the guanidino groups and the pyridine N atom. Test reactions, namely protonation and alkylations, were carried out to obtain information about the relative nucleophilicities of the three sites. Protonation, tested for L2b, occurs at the guanidino groups. Reaction with excess HCl leads to the diprotonated compound with the protons bound to the guanidino groups, but not to the pyridine N atom. The twofold protonated compound was crystallized with BF_4_^−^ counterions (Figure 9a); the structural analysis confirms protonation at the guanidino groups, but not at the pyridine ring. Protonation leads to a significant elongation of the imino N═C bond within the guanidino groups (1.349(3)/1.363(3) Å in (L2b+2H)^2+^). Quantum‐chemical calculations (B3LYP/def2‐TZVP) on (L2b+H)^+^ found the tautomer with the proton attached to the pyridine N atom to be energetically favored. The tautomers with a protonated guanidino group are by 10 (2‐position) or 41 kJ mol^−1^ (3‐position) less favorable. These results are in line with those obtained previously for 2,3,5,6‐tetrakis(tetramethylguanidino)pyridine, L1. Protonation of one of the guanidino groups in L1 was found to be energetically disfavored with respect to protonation at the pyridine N atom (Δ*G* = 24 kJ mol^−1^) [12], although the experiments showed protonation to occur exclusively at the guanidino groups.

**FIGURE 9:**
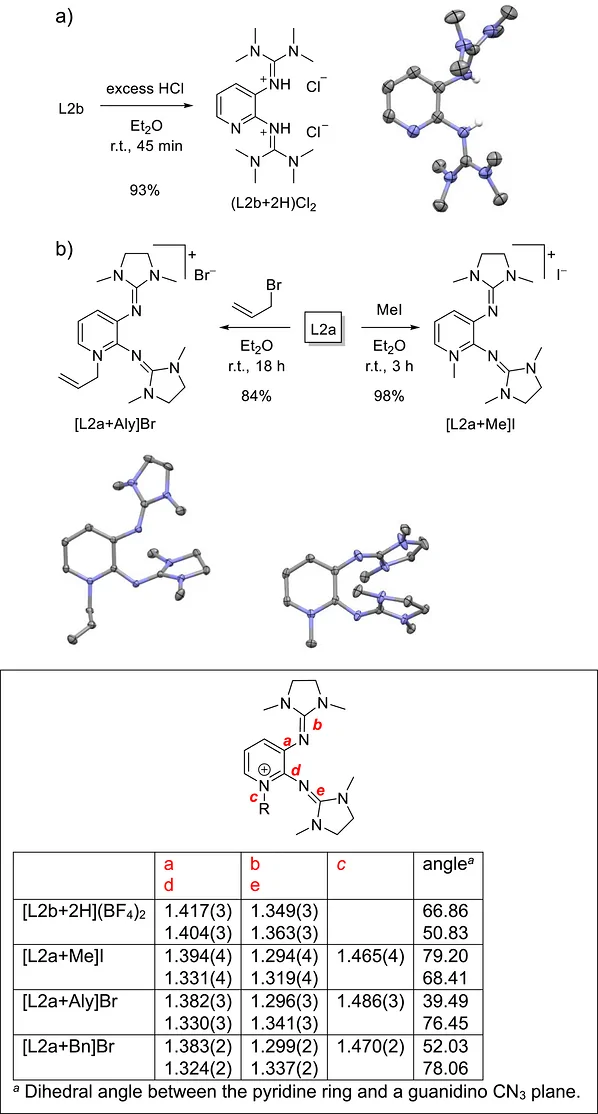
Equations for the protonation (a), as well as methylation and allylation (b) of L2a or L2b, and illustrations of the solid‐state structures from SC‐XRD. Displacement ellipsoids drawn at the 50% probability level. C─H hydrogen atoms and counterions omitted. Color code: C grey, N light blue, H white. A table with structural parameters (bond lengths in Å, angles in °) is included.

While protonation and metal coordination occur preferentially at the guanidino groups, methylation, allylation and benzylation reactions of unsubstituted 2,3­diguanidinopyridines (e.g. L2a) occur at the pyridine N atom. In Figure 9b, examples for methylation and allylation reactions are given. Interestingly, the two N═C bond lengths of the guanidino groups become quite different upon methylation, allylation, or benzylation. The N═C bond of the guanidino group adjacent to the pyridine N atom is considerably elongated, while the N═C bond in the other guanidino group remains short.

In the following, we focused on benzylation reactions, that are most relevant for the subsequently discussed photochemistry. We synthesized a variety of derivatives of benzylated L2a and L3, with different substituents attached to the phenyl ring of the benzyl group, since these compounds are of interest for the kinetic analysis of the photoreactions discussed below. All benzylated compounds were obtained in high yield from the benzyl bromide and the 2,3‐diguanidinopyridine and fully characterized. Figure 10 shows the structures of the compounds in the solid state as obtained from SC‐XRD measurements. Similar to methylation and allylation, the N═C imino bond in the guanidino group adjacent to the ring N atom is significantly elongated upon benzylation. In [L3+Bn(*o*,*p*‐CF_3_)_2_]Br, this bond (denoted e in Figure 10) is already 1.343(2) Å long. On the other hand, the C─N bond between the pyridine ring and the guanidino group becomes unusually short (1.312(2) Å), being shorter than the former N═C double bond. These peculiar bond parameters argue for contributions of two mesomeric structures to the electron distribution in the benzylated compounds (Scheme 2). One of them can be described as a pyridinium ion, with an intact 6π ring system and the positive charge localized on the pyridinium N atom. The second mesomeric structure is a guanidinium ion, in which the 6π ring system is removed and the positive charge localized on the guanidino group (Scheme 2). Please note that the two structures are no isomers, since they do not belong to two energy minimum structures on the potential‐energy hypersurface.

**FIGURE 10:**
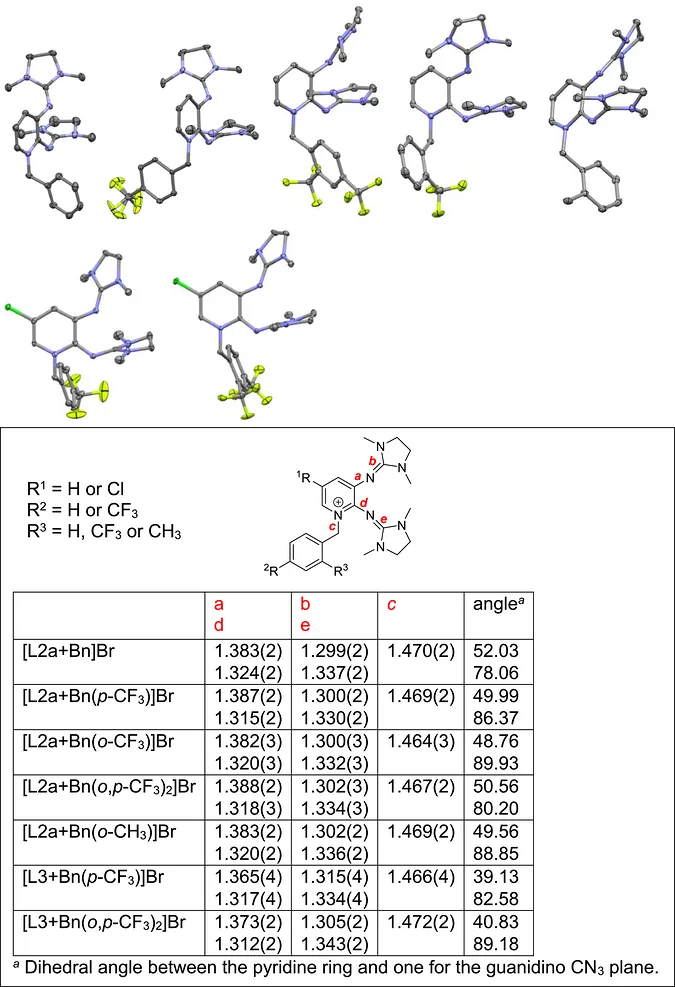
(a) Structures of the benzylated compounds of L2a and L3. Displacement ellipsoids drawn at the 50% probability level. Hydrogen atoms and counterions omitted. Color code: C grey, N light blue, Cl green, F yellow. A table with selected structural data (bond lengths in Å, angles in °) is included.

**SCHEME 2:**
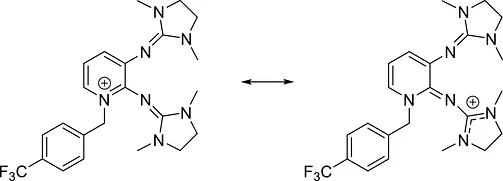
Mesomeric structures for the benzylated compounds.

Interestingly, benzylation does not occur at the pyridine ring for all compounds. There are also derivatives of 2,3‐diguanidinopyridine that are benzylated at the guanidino group adjacent to the ring N atom. Such behavior was observed for molecule L5a (Figure 11). The benzylated molecules without and with a CF_3_ group attached to the phenyl ring of the benzyl group were structurally characterized (Figure 11b). The table with the structural data shows that benzylation leads to a significant elongation of the C═N double bond. In this case, the C─N bond between the pyridine ring and the guanidino group remains long, since benzylation already accumulates the positive charge on this guanidino group. Benzyl bromides with two CF_3_ substituents at the phenyl ring show no reaction with L5a and L6. Moreover, no reaction was observed between L5b and unsubstituted or substituted benzyl bromides. Hence, the introduction of thioguanidino groups hampers benzylation.

**FIGURE 11:**
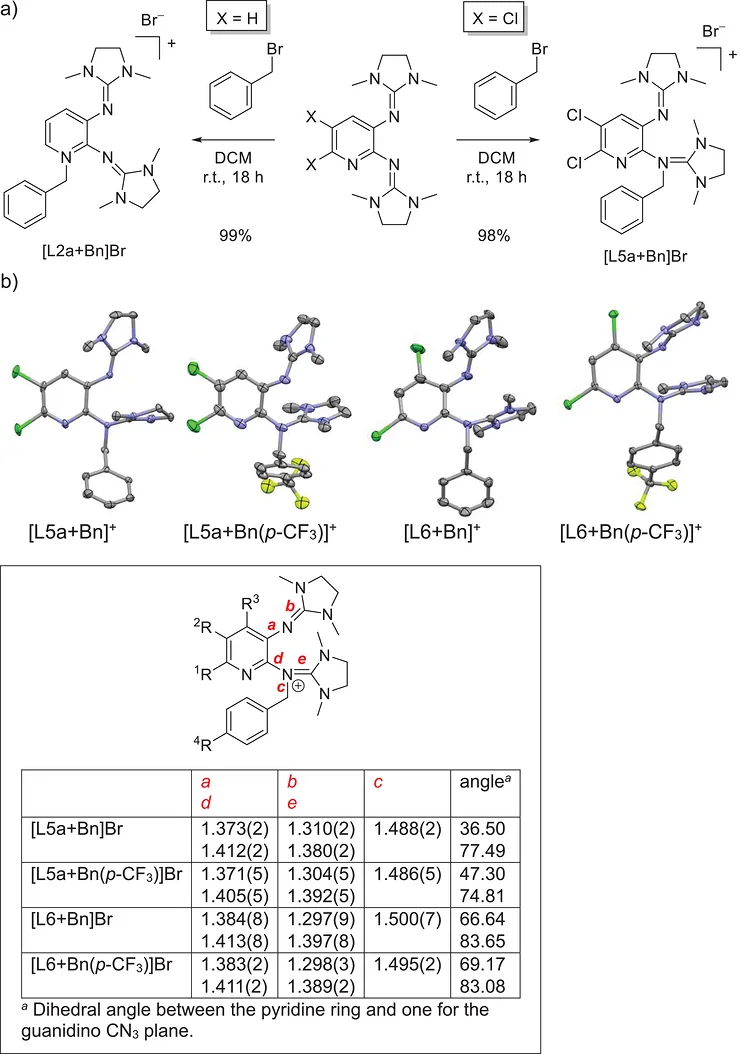
(a) Equations for benzylation at the pyridine N and the guanidino N atom. (b) Crystal structures of the benzylated compounds of L5a and L6. Hydrogen atoms, solvent molecules and counterions omitted. Displacement ellipsoids drawn at the 50% probability level. Color code: C grey, N blue, Cl green, F yellow. A table with selected structural data (bond lengths in Å, angles in °) is included.

Figure 12 compares the UV‐vis spectra obtained for pyridine N‐benzylated and guanidino‐benzylated molecules. It can be seen that the lowest energy excitation is more red‐shifted and more intense for the pyridine *N*‐benzylated molecules. An intense band centered near 360 nm, that extends into the visible region, appears in the spectra of the pyridine *N*‐benzylated compounds. By contrast, the lowest energy band is blue‐shifted to ca. 350 nm and of relatively low intensity for guanidino‐benzylated compounds. These differences have consequences on the photoreactivity (see the discussion below). Visible‐light irradiation initiates photoreactions in the case of pyridine *N*‐benzylated compounds, but not for the guanidino‐benzylated isomers.

**FIGURE 12:**
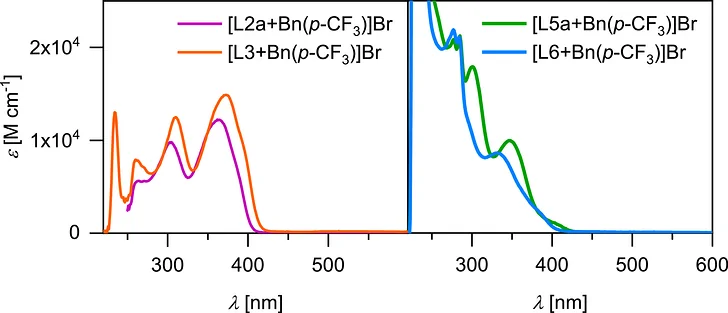
UV‐vis spectra for the benzylated molecules L2a, L3, L5a, and L6 with 4­(trifluoromethyl)benzyl group in dichloromethane. Left: Benzylation at the pyridine N. Right: Benzylation at the guanidino N.

Finally, there are also molecules that are benzylated both at the pyridine N and at the guanidino N atom, leading to a mixture of both isomers. Such behavior was observed for L4 and L6 (Scheme 3). In both cases, benzylation at the guanidino group is favored, but the pyridine *N*‐benzylated isomer is also clearly visible in the NMR spectra. From NMR signal integration, the ratios between the two isomers can be estimated (72:28 for L4 and 80:20 for L6, Scheme 3). The guanidino‐benzylated isomers crystallized from the reaction mixtures; illustrations of their crystal structures and selected structural parameters are included in Figure 11. The benzylation reaction was repeated in different solvents to see if the isomer ratio can be varied with the solvent polarity. However, no significant changes were observed. It was not possible to separate the two isomers, due to their similar properties. For example, both isomers are soluble in CH_3_CN and CH_2_Cl_2_, but both are insoluble in Et_2_O.

**SCHEME 3:**
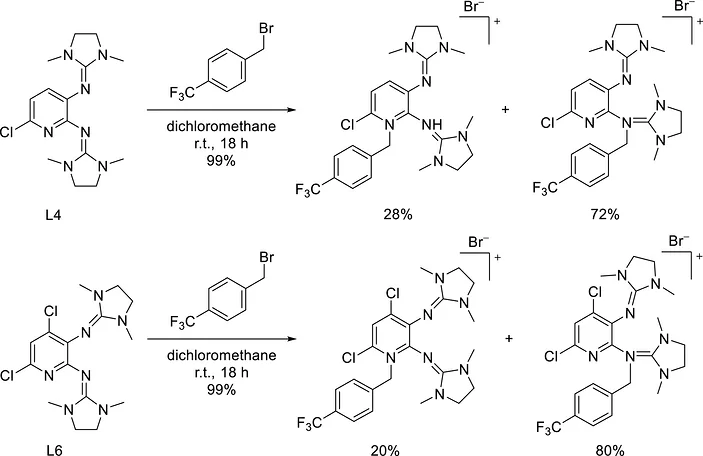
Benzylation reactions (NMR scale) leading to a mixture of two isomers, with the benzyl group attached to the pyridine N atom or the guanidino N atom.

### 2.4 Photochemistry

Experiments in which the benzylated GFAs were irradiated with visible light showed that the pyridine *N*‐benzylated compounds are photolabile. By contrast, the guanidino‐benzylated compounds are stable under visible light irradiation. First, we studied the photochemistry of benzylated L2a with different substituents at the phenyl ring of the benzyl group. Benzylation of L2a exclusively occurs at the pyridine N atom in high yields for all considered substituents at the benzyl group. All photo‐experiments were carried out with a 300 W lamp (385‐740 nm light).

Figure 13a shows, as an example, the UV‐vis spectra recorded before and after irradiation of a solution of [L2a+Bn(*o*,*p*‐CF_3_)_2_]Br in CH_2_Cl_2_ at different irradiation times. The intense, sharp absorptions of the starting compound at 366 and 303 nm rapidly decreased upon visible‐light irradiation. A new band of the photoisomerization product appeared at 283 nm. In addition, a weak, broad band around 650 nm and a weak shoulder near 450 nm appeared, that might belong to the radical monocationic ligand L2a**^·^** ^+^. However, an EPR signal was not detected. Presumably, the concentration of radicals is too low and their lifetime too short.

**FIGURE 13:**
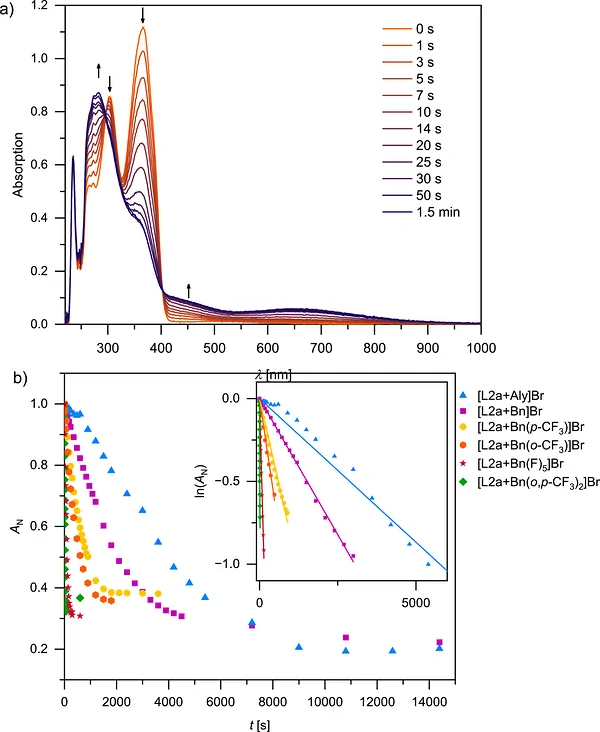
(a) UV‐vis spectra of [L2a+Bn(*o*,*p*‐CF_3_)_2_]Br (CH_2_Cl_2_, room temperature, *c* = 7.80·10^−5^ mol L^−1^) before and after visible‐light irradiation. (b) Normalized absorption *A*_N_ at the band maximum (ca. 360 nm) as a function of time for several benzyl‐pyridinium salts. Fits of the ln(*A*_N_) versus *t* plots to determine the rate constant *k* are embedded.

An NMR and mass spectrometric analysis of the photoproducts indicated photoisomerization to products in which the benzyl group is either attached to one of the guanidino groups (see discussion below) or substitutes one of the protons in the pyridine ring. Mass spectrometric data also indicated formation of di‐, tri‐ and even tetra‐benzylated molecules at very high concentrations of benzylated L2a.

Obviously, visible light initiates cleavage of the C─N bond between the benzyl group and the pyridine N atom. The characteristic band of the pyridine *N*‐benzylated compound vanishes upon visible‐light irradiation. In line with the results obtained with 1,2,4,5‐tetraguanidinopyridines (L1) [14], this photocleavage step is likely to be the rate‐determining step of the photoreaction. In Figure 13b, the absorption *A* is plotted as a function of time. For all compounds, the decline of the strong and characteristic band near 360 nm with irradiation time was followed. On the assumption of a first‐order kinetics for the bond cleavage step, the ln(A) versus time plots shown in the inlet of Figure 13b were fitted linearly to obtain the first‐order rate constants. The resulting rate constants *k* are collected in Table 3.

**TABLE 3:** First‐order rate constants *k* for the photoreaction of benzylated 2,3‐diguanidinopyridines and calculated Δ*G* values as well as *K*_c_.

Compounds	*k* [10^−4^ s^−1^]	Calculation Δ*G* [kJ mol^−1^]^a^	Compound	*k* [10^−4^ s^−1^]^b^
[L2a+Allyl]^+^	1.73 ± 0.03	163		
[L2a+Bn]^+^	3.29 ± 0.02	185	[L1+Bn]^+^	25.0 ± 1.7
[L2a+Bn(*p*‐CF_3_)]^+^	8.33 ± 0.13	171	[L1+Bn(*p*‐CF_3_)]^+^	300 ± 12
[L2a+Bn(*o*‐CF_3_)]^+^	12.8 ± 0.2	174	[L1+Bn(*o*‐CF_3_)]^+^	50.0 ± 1.7
[L2a+Bn(F)_5_]^+^	69.4 ± 1.3	163	[L1+Bn(F)_5_]^+^	96.7 ± 3.3
[L2a+Bn(*o*,*p*‐CF_3_)_2_]^+^	488 ± 11	163		

^a^Calculated values for homolytic bond cleavage to give benzyl radicals and L2a**^·^** ^+^.

^b^Values from Ref. [14].

The smallest rate constant is found for the allyl group ((1.73 ± 0.03)·10^−4^ s^−1^), and the largest rate constant for the compound with two CF_3_ substituents at the benzyl group ((488 ± 11)·10^−4^ s^−1^). As expected, the rate constants for the benzylated 2,3­diguandinopyridine molecules are smaller than those for the benzylated 1,2,4,5­tetraguanidinopyridine L1, if compounds with the same benzyl group are compared. The C─N bond between the benzyl group and the pyridine N atom is much weaker for L1 than for all 2,3‐diguanidinopyridines (Table 4). In the case of L1, the largest rate constant was obtained for the compound with the *p*‐CF_3_ group. Generally, electron‐withdrawing substituents at the phenyl ring of the benzyl group increase the rate constant. The highest *k* value of the 2,3‐diguanidinopyridines (488 ± 11 s^−1^) was obtained for [L2a+Bn(*o*,*p*‐CF_3_)_2_]^+^.

**TABLE 4:** Adiabatic Gibbs free energy changes (in kJ mol^−1^, calculated with B3LYP/def2‐TZVP) for C─N bond dissociation of benzylated pyridine derivatives. Bn = benzyl.

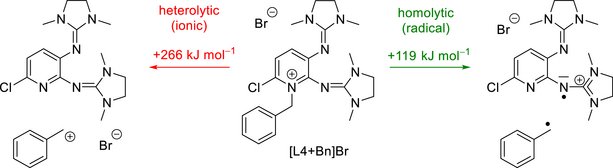		
Compounds	Δ*G* heterolytic	Δ*G* homolytic
[Pyridine+Bn]^+^	167	338
[L1+Bn]^+^	323	91
[L2a+Bn]^+^	301	185
[L2b+Bn]^+^	307	179
[L3+Bn]^+^	283	144
[L4+Bn]^+^	266	119
[L5a+Bn]^+^	246	133
[L5b+Bn]^+^	200	122
[L6+Bn]^+^	259	124

Quantum‐chemical calculations show that the HOMO and LUMO have no significant contribution from an orbital with pyridine‐benzyl N─C bond contribution (see Supporting Information). The electronic excitation is essentially a GFA‐centered π‐π* transition. The energy provided by excitation amounts to ca. 360 nm (332 kJ mol^−1^). Obviously, the energy required for bond cleavage should not exceed this limit. Quantum‐chemical calculations were carried out to obtain information about the energy needed to (adiabatically) cleave the N─C bond homolytically (radical pathway) or heterolytically (ionic pathway). Table 4 shows the results of these calculations. Homolytic bond cleavage, leading to a benzyl radical, is energetically preferred for all compounds with respect to heterolytic cleavage to give a benzyl cation. The electron‐donating effect of the guanidino groups is responsible for the preference for homolytic bond cleavage. Hence, for benzylated unsubstituted pyridine (first entry in Table 4), the homolytic N─C bond cleavage is highly disfavored compared to heterolytic bond cleavage leading to a benzyl cation. For L1 with four guanidino groups, a small Δ*G* value of 91 kJ mol^−1^ was obtained for homolytic cleavage. For the corresponding compound L2b with two guanidino groups, the Δ*G* value is nearly twice this value (Δ*G* = 179 kJ mol^−1^).

For all studied compounds, the energy of ca. 330 kJ mol^−1^ provided by photoexcitation is sufficient for homolytic bond cleavage. However, similar to the results obtained for the tetraguanidine L1, there is no correlation between the bond energy and the rate constant *k* for the photoreaction. The previous study on benzylated L1 [14] already showed that the rate *k* for photocleavage does not correlate with the C─N bond cleavage energy but critically depends on the relaxation rate of the excited molecule into the ground state. This rate for relaxation correlates with the differences between the ground state and excited state structures. A large structural difference leads to a slow rate of relaxation. The relaxation rate is likely to determine the overall rate for the photoreactions of benzylated 2,3‐diguanidinopyridines in a similar way.

The benzyl radical formed in the visible‐light induced N─C bond cleavage step remains in the solvent cage and quickly reacts further with the radical cationic 2,3­diguanidinopyridine. Therefore, a radical‐trapping experiment with TEMPO showed no significant difference, and an EPR spectrum recorded immediately after irradiation showed no signal. The benzyl radical either binds to the guanidino group or attacks a pyridine carbon atom. Relatively clean photoreactions should occur for compounds in which an attack at the pyridine ring (especially in 6‐position) is prohibited by a substituent. Therefore, the photochemistry of benzylated L4 and L6 was tested. In both cases, the solutions contain a mixture between the photo‐active benzyl‐pyridinium ion and the photo‐stable guanidino‐benzylated isomer (Scheme 3). Figure 14 shows the ^1^H NMR spectra recorded before and after irradiation of a solution of benzylated L4 (mixture of pyridine *N*‐benzylated and guanidino‐benzylated isomers). It can be seen that irradiation leads to the extinction of the signals of the pyridine *N*‐benzylated isomer (highlighted with green bars) and the growth of the signals due to the guanidino‐benzylated isomer (highlighted with blue bars). Hence, the pyridine *N*‐benzylated salt is photoconverted into the guanidino‐benzylated isomer.

**FIGURE 14:**
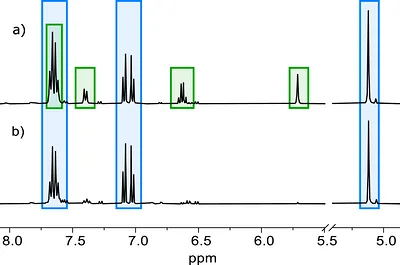
^1^H NMR spectra before (a) and after 5 min visible‐light irradiation (b) of a solution of [L4+Bn(*p*‐CF_3_)]Br (mixture of the pyridine *N*‐benzylated (green) and the guanidino‐benzylated (blue) isomers) in CD_3_CN (in the presence of added Cs_2_CO_3_).

The photoreaction can also be followed with UV‐vis spectroscopy. The mixture of the two isomers of [L4+Bn(*p*‐CF_3_)]^+^ contains characteristic bands at 299 nm and a shoulder between 385 and 415 nm for the pyridine *N*‐benzylated and at 285 nm for the guanidino‐benzylated isomer. Upon irradiation of the mixture, the shoulder and the band at 299 nm due to the pyridine *N*‐benzylated isomer vanished.

Coordination prohibits the alkylation of a guanidino group and might therefore change the photochemistry of pyridine *N*‐benzylated *o*‐diguanidino‐pyridines. We tested the synthesis of a transition‐metal complex of a pyridine‐*N* benzylated 2,3‐diguanidino‐pyridine. However, complexes of 2,3‐diguanidino‐pyridines (e.g. [ZnCl_2_(L4)]) did not react with benzyl bromides. Additional experiments were carried out to synthesize transition‐metal complexes starting with the benzylated 2,3‐diguanidino‐pyridine. In the case of reaction of benzylated L2a, [L2a+Bn(*p*‐CF_3_)]Br, with CuBr_2_, we isolated small amounts of a bromo‐substituted diguanidino‐pyridine. The proton removed from the pyridine ring adds to a guanidino N atom to give eventually the benzylated and protonated dication [L2a+Br+Bn(*p*‐CF_3_)]^2+^, that crystallized as salt [L2a+Br+Bn(*p*‐CF_3_)]CuBr_4_ (protocol and crystal structure included in the Supporting Information). In summary, coordination cannot be used to change the photochemistry.

According to quantum‐chemical calculations (B3LYP+D3/def2‐TZVP), the isomerization of the pyridine *N*‐benzylated to the guanidino‐benzylated isomer is endergonic (Δ*G* = 10.2 kJ mol^−1^) for benzylated L6 (Table 5). Hypothetical benzylation at the guanidino group in 3‐position (*meta*‐position) is thermodynamically less favorable for all compounds (Δ*G* = 16.5 kJ mol^−1^ in the case of L6 (see Supporting Information)). Hence, kinetic and thermodynamic arguments explain why only the guanidino group in 2‐position (*ortho*‐position) is benzylated.

**TABLE 5:** Gibbs free energy change Δ*G* (in kJ mol^−1^) for the isomerization of benzylated 2,3‐diguanidinopyridines.

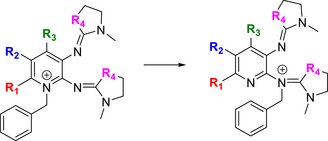					
Compounds	R_1_	R_2_	R_3_	R_4_	Δ*G*
[L2a+Bn]^+^	H	H	H	NCH_3_	42.4
[L3+Bn]^+^	H	Cl	H	NCH_3_	29.7
[L4+Bn]^+^	Cl	H	H	NCH_3_	13.4
[L5a+Bn]^+^	Cl	Cl	H	NCH_3_	6.0
[L5b+Bn]^+^	Cl	Cl	H	S	22.2
[L6+Bn]^+^	Cl	H	Cl	NCH_3_	10.2

Obviously, the energy difference between the two relevant isomers can be varied by modifying the guanidino group. In line with the experimental results, showing that benzylation exclusively occurs at the pyridine N atom for compound L2a, the calculations found the highest isomerization energy for benzylated L2a. In the case of L5a, the experiments found exclusively benzylation at the guanidino group. Although the calculations on benzylated L5a still predict the pyridine *N*‐benzylated isomer to be lower in energy than the guanidino‐benzylated isomer, the energy difference is very small (6 kJ mol^−1^). Ligand L5b exhibits two thioguanidino groups, leading to a higher ionization energy in comparison to L5a and a larger difference between the energetically favored pyridine *N*‐benzylated and the guanidino‐benzylated isomer. Since benzylation is exclusively observed at the guanidino group for L5a, we experimentally tested benzylation of L5b. The thioguanidino group has a reduced ability to stabilize a positive charge. Therefore, guanidino‐benzylation should be less favorable for L5b than for L5a, shifting the isomerization equilibrium toward the pyridine *N*‐benzylated compound. However, the experiments showed that benzylation of L5b does not occur at either position; a reaction between L5b and benzylbromide was not observed.

The Δ*G* values in Table 5 show that the relative energies of the two isomers can be varied over a wide range by substitution and choice of the guanidino/thioguanidino group. Hence, photoisomerization, converting the energetically favored pyridine *N*‐benzylated form into the more energetic guanidino‐benzylated form, might be a way to store solar energy in molecular compounds. Molecular compounds that can be isomerized by light are attractive for a variety of applications, for example, in photoswitching or energy storage devices [25, 26]. Examples include curved anthracences [27], 1,2‐dihydro‐1,2‐azaborinines [28], azabenzene‐based compounds [29], fulvalene diruthenium compounds [26], and hydrazones [30]. Several compounds for photon‐energy storage devices rely on Dewar isomerization processes. A strained photoproduct is formed that can be thermally (in some cases in a chemical reaction, for example, initiated by the addition of an acid) converted back with release of energy. Most compounds put forward up to date require UV irradiation. By contrast, isomerization of benzylated 2,3­diguanidinopyridines is achieved with visible‐light.

## 3 Conclusions

This work establishes 2,3‐diguanidinopyridines as new redox‐active molecules and platforms for coordination and photochemistry. Based on an analysis of the redox properties, pyridine ring substitution is used to build the first diguanidinopyridine molecules that are reversibly oxidized in two potentially‐separated one‐electron steps to the radical monocationic and dicationic redox states. The compounds are excellent ligands in transition metal complexes, with three accessible, stable redox states, and metal coordination leads to considerable anodic shifts of the ligand redox potentials. Protonation and metal coordination occur exclusively at the guanidino groups. On the other hand, the situation is more complex for benzylation reactions. Benzylation of unsubstituted 2,3‐diguanidinopyridines occurs at the pyridine N atom. The introduction of chloro substituents at the pyridine ring leads, in dependence of the substitution pattern, to pyridine N or guanidino‐benzylated compounds, or mixtures of both isomers. All pyridine *N*‐benzylated 2,3‐diguanidinopyridine molecules are photo‐sensitive; irradiation with visible light triggers isomerization to give the higher‐energetic, photostable guanidino‐benzylated isomers. The results might pave the way for the development of a novel photoreactivity, that is interesting in the context of solar energy storage, since the visible‐light photoreaction leads to conversion to a higher‐energetic isomer. This work provides a basis for further research in this direction. Obviously, much more work, being outside the scope of the present study, is required to test the potential of these molecules in applications.

## Conflicts of Interest

The authors declare no conflicts of interest.

## Supporting information



The authors have cited additional references within the Supporting Information [31, 32, 33, 34, 35, 36, 37, 38, 39, 40, 41, 42, 43, 44, 45, 46, 47, 48, 49, 50, 51, 52, 53, 54].
**Supporting File 1**: chem71582‐sup‐0001‐SuppMat.pdf.

**Supporting File 2**: chem71582‐sup‐0001‐SuppMat.pdf.

## Data Availability

The data that supports the findings of this study are available in the Supporting Information of this article.
