# Supplementary material for: The Radical Chemistry of 2,3‐Diguanidinopyridines: Coordination and Visible‐Light Photochemistry
Source: Chemistry. 2026 Aug 18;32(41):e71582. doi: 10.1002/chem.71582 (PMC13729947; doi:10.1002/chem.71582)
Supplement: Supplementary file 1 [file CHEM-32-e71582-s001.pdf]

Table of contents

1. General information 4
1.1 Experimental details 4
1.2 X-ray crystallography 6
1.3 DFT Calculation details 7
2. Synthesis 8
2.1 Ligands 8
2.1.1 L2a 8
2.1.2 L2b 12
2.1.3 $[L2bH_2]Cl_2$ 16
2.1.4 L3 18
2.1.5 L4 22
2.1.6 $[L4H_2]Cl_2$ 26
2.1.7 L5a 28
2.1.8 $[L5a]PF_6$ 32
2.1.9 L5b 34
2.1.10 L6 38
2.1.11 $[L6]PF_6$ 42
2.2 Complexes 44
2.2.1 $[NiCl_2(L2a)]$ 44
2.2.2 $[CoCl_2(L2a)]$ 46
2.2.3 $[CoBr_2(L2a)]$ 48
2.2.4 $[Co(hfac)_2(L2a)]$ 50
2.2.5 $[CuBr_2(L2a)]$ 52
2.2.6 $[Cu(L2a)_2](BF_4)_2$ 54
2.2.7 $[ZnCl_2(L4)]$ 56
2.2.8 $[PdCl_2(L6)]$ 59
2.2.9 $[PdCl_2(L6)]PF_6$ 62
2.3 Pyridinium salts 64
2.3.1 General procedure 1 64
2.3.2 General procedure 2 64
2.3.3 [L2a+Me]I 65
2.3.4 [L2a+Aly]Br 67

2.3.5 [L2a+Bn]Br 70
2.3.6 [L2a+Bn(*p*-CF3)]Br 74
2.3.7 [L2a+Bn(*o*,*p*-CF_3)$_2$]Br 78
2.3.8 [L2a+Bn(*o*-CF_3)]Br 83
2.3.9 [L2a+Bn(F)$_5$]Br 88
2.3.10 [L2aBrH+Bn(*p*-CF_3)]$CuBr_4$ 93
2.3.11 [L2b+Bn]Br 94
2.3.12 [L3+Bn(*p*-CF_3)]Br 96
2.3.13 [L3+Bn(*o*,*p*-CF_3)$_2$]Br 99
2.3.14 [L4+Bn(*p*-CF_3)]Br 102
2.3.15 [L5a+Bn^b(*p*-CF_3)]Br 105
2.3.16 [L6+Bn^b(*p*-CF_3)]Br 108
3. Crystal structures 111
3.1 Ligands 111
3.1.1 L2a 111
3.1.2 [L2b+2H](BF_4)$_2$ 112
3.1.3 L3 113
3.1.4 L4 114
3.1.5 L5a 115
3.1.6 L5b 116
3.1.7 L6 117
3.1.8 [L2b$_2$H$_2$](PF_6)$_4$ 118
3.1.9 [L2a$_2$](PF_6)$_2$ 119
3.2 Complexes 120
3.2.1 [$NiCl_2$(L2a)] 120
3.2.2 [$CoCl_2$(L2a)] 121
3.2.3 [$CoBr_2$(L2a)] 122
3.2.4 [Co(hfac)$_2$(L2a)] 123
3.2.5 [$CuBr_2$(L2a)] 124
3.2.6 [Cu(L2a)$_2$](BF_4)$_2$ 125
3.2.7 [$ZnCl_2$(L4)] 126
3.2.8 [$PdCl_2$(L6)] 127
3.3 Pyridinium salts 128
3.3.1 [L2a+Me]I 128

3.3.2 [L2a+Aly]Br 129
3.3.3 [L2a+Bn]Br 130
3.3.4 [L2a+Bn(*p*-CF_3)]Br 131
3.3.5 [L2a+Bn(*o*,*p*-CF_3)$_2$]Br 132
3.3.6 [L2a+Bn(*o*-CF_3)]Br 133
3.3.7 [L2a+Bn(*o*-Me)]Br 134
3.3.8 [L2aBrH+Bn(*p*-CF_3)]$CuBr_4$ 135
3.3.9 [L2b+Bn]Br 136
3.3.10 [L3+Bn(*p*-CF_3)]Br 137
3.3.11 [L3+Bn(*o*,*p*-CF_3)$_2$]Br 138
3.3.12 [L5a+Bn^b]Br 139
3.3.13 [L5a+Bn^b(*p*-CF_3)]Br 140
3.3.14 [L6+Bn^b]Br 141
3.3.15 [L6+Bn^b(*p*-CF_3)]Br 142
4. DFT Calculations 143
4.1 Protonation 145
4.2 Bond cleavage energies 145
4.3 HOMOs 147
4.4 Gibbs free energy changes 151

1. General information

1.1 Experimental details

All synthetic work was carried out using standard Schlenk techniques under argon atmosphere. The solvents acetonitrile, dichloromethane, diethyl ether and pentane were dried with a MBraun MB-SPS-800 Solvent Purification System and stored over molecular sieves (3 Å and 4 Å). Other solvents were purchased from Acros Organics. Deuterated solvents were purchased at Eurisotop. The following chemicals were purchased and used as delivered: Pyridine-2,3-diamine (95%, Sigma Aldrich), 5-Chloropyridine-2,3-diamine (98%, BLD pharm), 6-Chloropyridine-2,3-diamine (98%, BLD pharm), 5,6-Dichloropyridine-2,3-diamine (95%, fluorochem), 4,6-Dichloropyridine-2,3-diamine (95%, BLD pharm) triethylamine (Sigma Aldrich), hydrochloride solution (2.0 M in diethylether, Sigma Aldrich), nickel(II) chloride dimethoxyethane adduct (98%, Sigma Aldrich), cobalt(II) chloride (97%, Sigma Aldrich), cobalt(II) bromide (99%, Sigma Aldrich), dried cobalt(II)hexafluoroacetylacetonate hydrate (97%, abcr), copper(II) bromide (99%, Sigma Aldrich), copper(II) tetrafluoroborate hexahydrate (96%, Sigma Aldrich), zinc chloride (98%, Sigma Aldrich), bis(acetonitril)dichlorpalladium(II) (99%, Sigma Aldrich), 4-(trifluoromethyl)benzylbromide (98%, BLD pharm), 1-(bromomethyl)-2,4-bis(trifluoromethyl)benzol (97%, thermo scientific), 1-(bromomethyl)-2-(trifluoromethyl)benzene (99%, Sigma Aldrich), 1-(bromomethyl)-2-methylbenzene (98%, Sigma-Aldrich), 1-(bromomethyl)-2,3,4,5,6-pentafluorobenzene (99%, Sigma Aldrich). The synthesis of 2-chloro-1,3-dimethylimidazolium chloride (1),[1] 2-chloro-1,1‘,3,3‘-tetramethylformamidinium chloride (2),[2] 2-chloro-3-methyl-4,5-dihydrothiazol-3-ium chloride (3)[3] and ferrocenium hexafluorophosphate[4] were synthesized by literature known procedures.

The analytical data was plotted using OriginPro 2026 (64-bit) SR1. Elemental analysis were performed at the Microanalytical Laboratory of Heidelberg University using the vario EL and vario MICRO cube devices from Elementar Analysensysteme GmbH. Please note that the compounds are strong Brønsted bases and redox-active. Therefore, some deviations are caused by reactions with traces of water or dioxygen during the elemental analysis measurements. NMR spectra were recorded on a Bruker Avance II 400, Bruker AVANCE III 600 or Bruker 600 Ultrashield system at 298 K. Solvent resonances were taken as references for all ^{1}H NMR spectra. Analyses of the

spectra were performed using MestReNova. UV-Vis spectra were recorded with a Cary 5000 spectrophotometer. Irradiation measurements were carried out using an Excalibur Lab solid-quarz probe made of quarz glass by Hellma, with an optical path length of 10 mm and a UV-Vis spectral range of 210-1100 nm. The optical fibres with SMA connectors, which have a high OH content, cover a wavelength range of 210-1100 nm. The probe was connected to the spectrometer using an optical fibre coupler from Aglient Technologies for the Cary 4000-6000i, suitable of optical fibres with SMA connectors. CV measurements were carried out with a Metrohm Autolab PGSTAT 204 potentiostate/galvanostate and an Ag/AgCl reference electrode, Pt rod counter electrode and glassy carbon working electrode. All voltammograms were recorded at room temperature. CH_2Cl_2 and CH_3CN were used as solvents for the individual compounds (concentration around 10^{-3} M, if not stated otherwise), whereas $n\mathrm{Bu_4N(PF_6)}$ (electrochemical grade (≥99.0%), Fluka) was employed as supporting electrolyte (c = 0.1 M). The potentials are given vs. the reference redox pair ferrocenium/ferrocene (Fc^+/Fc), measured at $E_{1/2}$ = 0.42 V in CH_3CN and 0.46 V in CH_2Cl_2. HR-ESI spectra were recorded with a Bruker ApexQe hybrid 9.4 T FT-ICR spectrometer and HR-EI spectra with a JEOL AccuTOF GCx. X-band EPR spectra were measured at a magnettech MiniScope MS400 spectrometer (room temperature measurements, ~9.44 GHz) in NMR tubes. Irradiation reactions were carried out using a MAX-303 Compact Xenon Light Source from ASAHI SPECTRA, with a MAX303-UV.L.C lamp (300 W), MAX-VIS (385-740) optics and a MAX-VIS.F.S lens.

[1] A. Peters, E. Kaifer, H.-J. Himmel, Eur. J. Org. Chem. 2008, 5907–5914.
[2] P. Roquette, C. König, O. Hübner, A. Wagner, E. Kaifer, M. Enders, H.-J. Himmel, Eur. J. Inorg. Chem. 2010, 4770– 4782.
[3] U. Wild, O. Hübner, S. Jaworski, E. Kaifer, H.-J. Himmel, Chem. Eur. J. e202670981.
[4] U. Jahn, P. Hartmann, I. Dix, P.G. Jones, Eur. J. Org. Chem. 2001, 17, 3333−3355.

1.2 X-ray crystallography

Suitable crystals for single-crystal structure determination were taken directly from the mother liquor, taken up in per-fluorinated polyether oil and fixed on a cryo loop. Full shells of intensity data were collected at low temperature with a Bruker D8 Venture, dual source (Mo-Kα radiation, microfocus X-ray tube, Photon III detector). Data were processed with the standard Bruker (SAINT, APEX3/4/6) software package.[5] Multiscan absorption correction was applied using the SADABS program.[6] The structures were solved by intrinsic phasing[7] and refined using the SHELXTL software package (Version 2018/3).[8] Graphical handling of the structural data during solution and refinement were performed with OLEX2.[9] All non-hydrogen atoms were given anisotropic displacement parameters. Hydrogen atoms bound to carbon were input at calculated positions and refined with a riding model. Hydrogen atoms bound to nitrogen were located in difference Fourier syntheses and refined, either fully or with appropriate distance and/or symmetry. Split atom models were used to refine disordered groups and/or solvent molecules. When found necessary, suitable geometry and adp restraints were applied.[10,11] Due to severe disorder and fractional occupancy, electron density attributed to the solvent of crystallization was removed from some of the structures with the BYPASS procedure,[12] as implemented in PLATON (squeeze/hybrid). The visualization of the structures was performed using Mercury 4.2.0. Displacement ellipsoids are depicted with 50% probability.[13]

Deposition Numbers 2562868 for L2a, 2562869 for [L2b$_2$H$_2$](PF$_6$)$_4$, 2562871 for [L2a$_2$](PF$_6$)$_2$, 2562870 for [L2bH$_2$](BF$_4$)$_2$, 2562872 for L5b, 2562873 for L4, 2562874 for L6, 2562875 for L5a, 2562876 for L3, 2562880 for [CuBr$_2$(L2a)], 2562881 for [CoBr$_2$(L2a)], 2562882 for [CoCl$_2$(L2a)], 2562883 for [NiCl$_2$(L2a)], 2562884 for [Co(hfac)$_2$(L2a)], 2562885 for [Cu(L2a)$_2$](BF$_4$)$_2$, 2562886 for [ZnCl$_2$(L4)], 2562887 for [PdCl$_2$(L6)], 2562888 for [L2b+Bn]Br, 2562889 for [L2a+Bn]Br, 2562890 for [L2a+Bn(*p*-CF$_3$)]Br, 2562891 for [L2aBr+Bn(*p*-CF$_3$)]CuBr$_4$, 2562892 for [L2a+Me]I, 2562893 for [L2a+Aly]Br, 2562894 for [L2a+Bn(*o*-Me)]Br, 2562895 for [L2a+Bn(*o*-CF$_3$)]Br, 2562896 for [L2a+Bn(*o*,*p*-CF$_3$)$_2$]Br, 2562897 for [L6+Bn^b]Br, 2562898 for [L5a+Bn^b]Br, 2562899 for [L5a+Bn^b(*p*-CF$_3$)]Br, 2562900 for [L6+Bn^b(*p*-CF$_3$)]Br, 2562901 for [L3+Bn(*o*,*p*-CF$_3$)$_2$]Br, 2562902 for [L3+Bn(*p*-CF$_3$)]Br contain the supplementary crystallographic data for this paper. These data are provided free of charge by the joint Cambridge Crystallographic Data Centre and Fachinformationszentrum Karlsruhe Access Structure service.

[5] SAINT (APEX 3/4/6) Bruker AXS GmbH, Karlsruhe, Germany 2016/2021/2025.
[6] a) G. M. Sheldrick, SADABS, Bruker AXS GmbH, Karlsruhe, Germany 2004- 2014; b) L. Krause, R. Herbst-Irmer, G. M. Sheldrick, D. Stalke, J. Appl. Cryst. 2015, 48, 3–10.
[7] a) G. M. Sheldrick, SHELXT, Program for Crystal Structure Solution, University of Göttingen, Germany 2014-2018; b) G. M. Sheldrick, Acta Cryst. 2015, C71, 3–8.
[8] a) G. M. Sheldrick, SHELXL-20xx, University of Göttingen and Bruker AXS GmbH, Karlsruhe, Germany 2012-2018; b) W. Robinson, G. M. Sheldrick in: N. W. Isaaks, M. R. Taylor (eds.) „Crystallographic Computing 4“, Ch. 22, IUCr and Oxford University Press, Oxford, UK, 1988; c) G. M. Sheldrick, Acta Cryst. 2008, A64, 112–122.
[9] O. V. Dolomanov, L. J. Bourhis, R. J. Gildea, J. A. K. Howard, H. Puschmann, J. Appl. Cryst. 2009, 42, 339–341.
[10] A. Thorn, B. Dittrich, G. M. Sheldrick, Acta Cryst. 2012, A68, 448–451.
[11] a) P. v. d. Sluis, A. L. Spek, Acta Cryst. 1990, A46, 194–201; b) A. L. Spek, Acta Cryst. 2015, C71, 9–18.
[12] A. L. Spek, PLATON, Utrecht University, The Netherlands; b) A. L. Spek, J. Appl. Cryst. 2003, 36, 7–13.
[13] C. F. Macrae, I. Sovago, S. J. Cottrell, P. T. A. Galek, P. McCabe, E. Pidcock, M. Platings, G. P. Shields, J. S. Stevens, M. Towler and P. A. Wood, J. Appl. Cryst. 2020, 53, 226–235.

1.3 DFT Calculation details

All density functional calculations were performed with the Orca 5.0.1 version,[14] using the B3LYP[15] functional in combination with dispersion correction D3[16] and def2-TZVP[17] as basis sets. The calculations used the resolution of identity approximation[18] for the coulomb integrals with the def2/J auxiliary basis set, and numerical chain-of-sphere integration for the HF Exchange integrals. As starting point for the structure optimizations existing crystal structure coordinates were used. For some calculations, the crystal structures were adapted using Chemcraft 1.8. The final structures were visualized using Mercury 2022.3.0, orbitals were visualized using Avogadro 1.2.0.

[14] a) F. Neese, WIREs Comput. Mol. Sci. 2012, 2, 73–78; b) F. Neese, WIREs Comput. Mol. Sci. 2022, 12, e1606.
[15] A. D. Becke, J. Chem. Phys. 1993, 98, 5648.
[16] S. Grimme, J. Antony, S. Ehrlich, H. Krieg, J. Chem. Phys. 2010, 132, 154104.
[17] A. Schäfer, H. Horn, R. Ahlrichs, J. Chem. Phys. 1992, 97, 2571–2577.
[18] K. Eichkorn, O. Treutler, H. Ohm, M. Häser, R. Ahlrichs, Chem. Phys. Lett. 1995, 240, 283–290.

2. Synthesis

2.1 Ligands

2.1.1 L2a

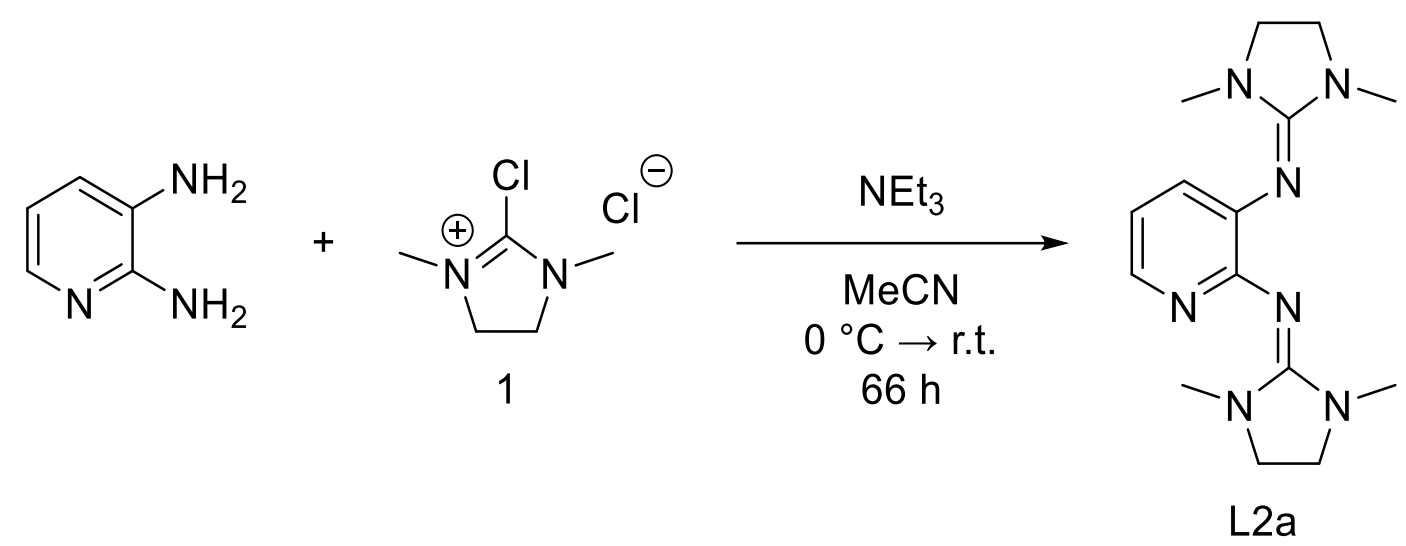

Pyridine-2,3-diamine (250 mg, 2.29 mmol, 1.00 eq.) was added to a Schlenk flask and dissolved in acetonitrile. A solution of 2-chloro-1,3-dimethylimidazolinium chloride (1) in acetonitrile (1 M, 5.73 mL, 968 mg, 5.73 mmol, 2.50 eq.) was added and the reaction mixture was stirred at 0 °C for 15 min before slowly adding triethylamine (1.28 mL, 927 mg, 9.16 mmol, 4.00 eq.). The brown reaction mixture was then stirred for additional 66 h while slowly warming up to room temperature. After removing the solvent under reduced pressure, the residue was solved with HCl_{aq} (5.0 mL, 10%) and $NaOH_{aq}$ (15.0 mL, 25 %) was added while stirring. The aqueous phase was extracted with dichloromethane (5 x 10 mL) and dried over sodium sulphate. After removing the solvent, the residue was solved in 8 mL toluene and heated to 100 °C while stirring. The mixture was cooled slowly and stored at −30 °C for 66 h, the solution was purified by filtration, and the product was dried under reduced pressure, yielding L2a (427 mg, 1.42 mmol, 62%) as brown viscose liquid.

Crystals for structural characterisation were obtained though spontaneous crystallisation of this viscose liquid after some time.

^{1}H NMR (600.15 MHz, CD_2Cl_2)

δ = 7.71-7.70 (m, 1H, CH_{py}), 6.88-6.87 (m, 1H, CH_{py}), 6.61-6.58 (m, 1H, CH_{py}), 3.28-3.28 (m, 4H, CH_2), 3.22-3.22 (m, 4H, CH_2), 2.62-2.61 (m, 12H, CH_3) ppm.

^{13}C{^{1}H} NMR (150.93 MHz, CD_2Cl_2)

δ = 155.5 (1C, C_q), 155.4 (1C, C_q), 154.6 (1C, C_q), 139.3 (1C, CH), 138.3 (1C, C_q), 128.0 (1C, CH), 115.5 (1C, CH), 48.6 (2C, CH_2), 48.3 (2C, CH_2), 34.6 (2C, CH_3), 34.3 (2C, CH_3) ppm.

Mass spectrometry (ESI^+ in DCM)

m/z = 302.2090 ($[L2a+H]^+$, calc. 302.2088).

UV-vis (DCM) ($1.06 \cdot 10^{-4}$ mol L^{-1})

λ (ε) = 319 (8080), 284 (9510), 229 (14800) nm (M^{-1} cm^{-1}).

CV (DCM, [*n*-Bu_4N][PF_6], 20 mV s^{-1}, referenced to Fc/Fc^+)

E_{Ox} = −74.3 mV.

CV (MeCN, [*n*-Bu_4N][PF_6], 20 mV s^{-1}, referenced to Fc/Fc^+)

E_{Ox} = −129 mV.

Elemental analysis (C, H, N in %) ($C_{15}H_{23}N_7 \cdot 0.1$ eq. DCM)

calc.: C 58.53, H 7.55, N 31.64.

found: C 58.53, H 7.41, N 31.04.

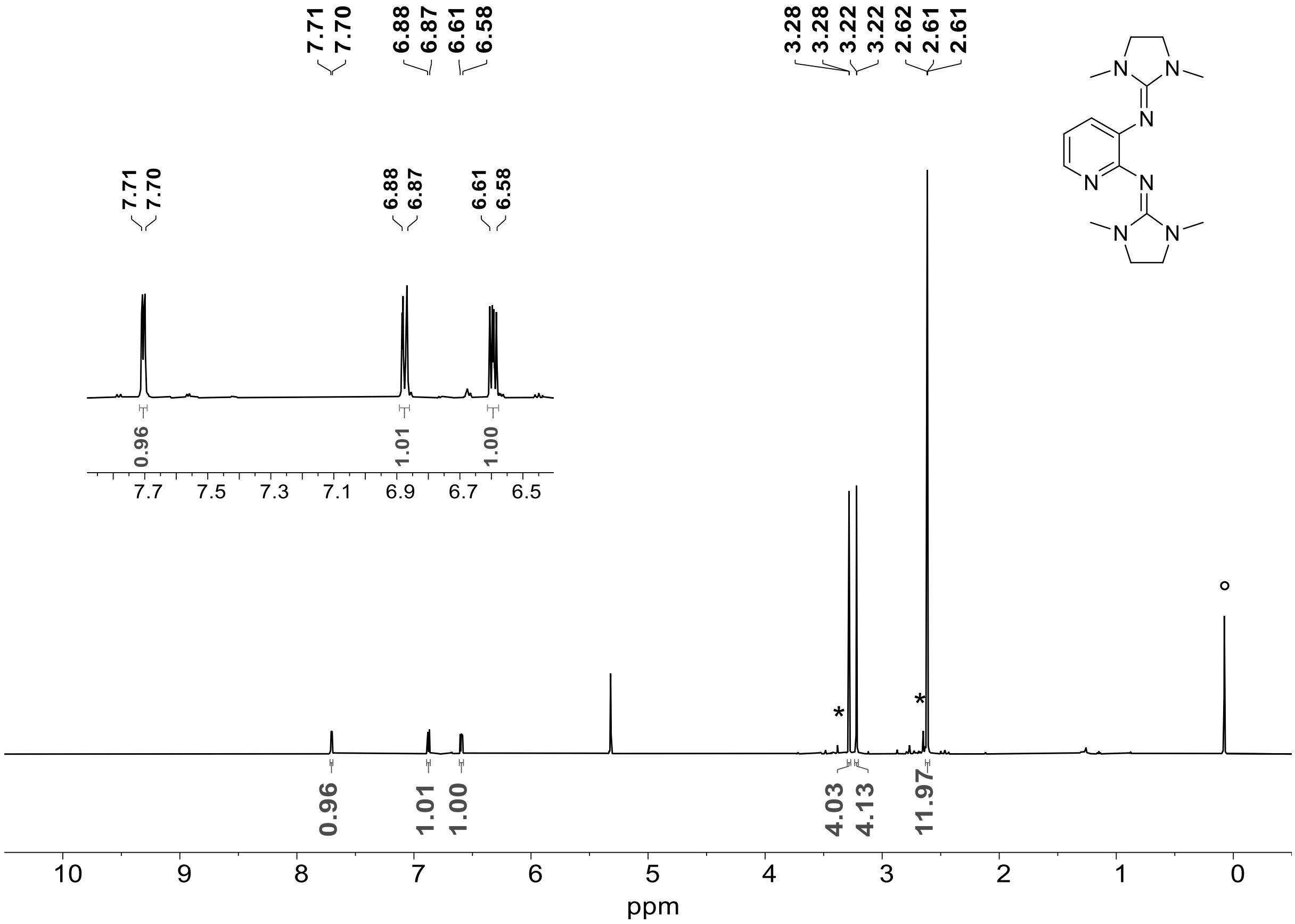

Figure S1: ^{1}H NMR spectrum of L2a, measured at 600 MHz in CD_2Cl_2, *deactivated 1, °grease.

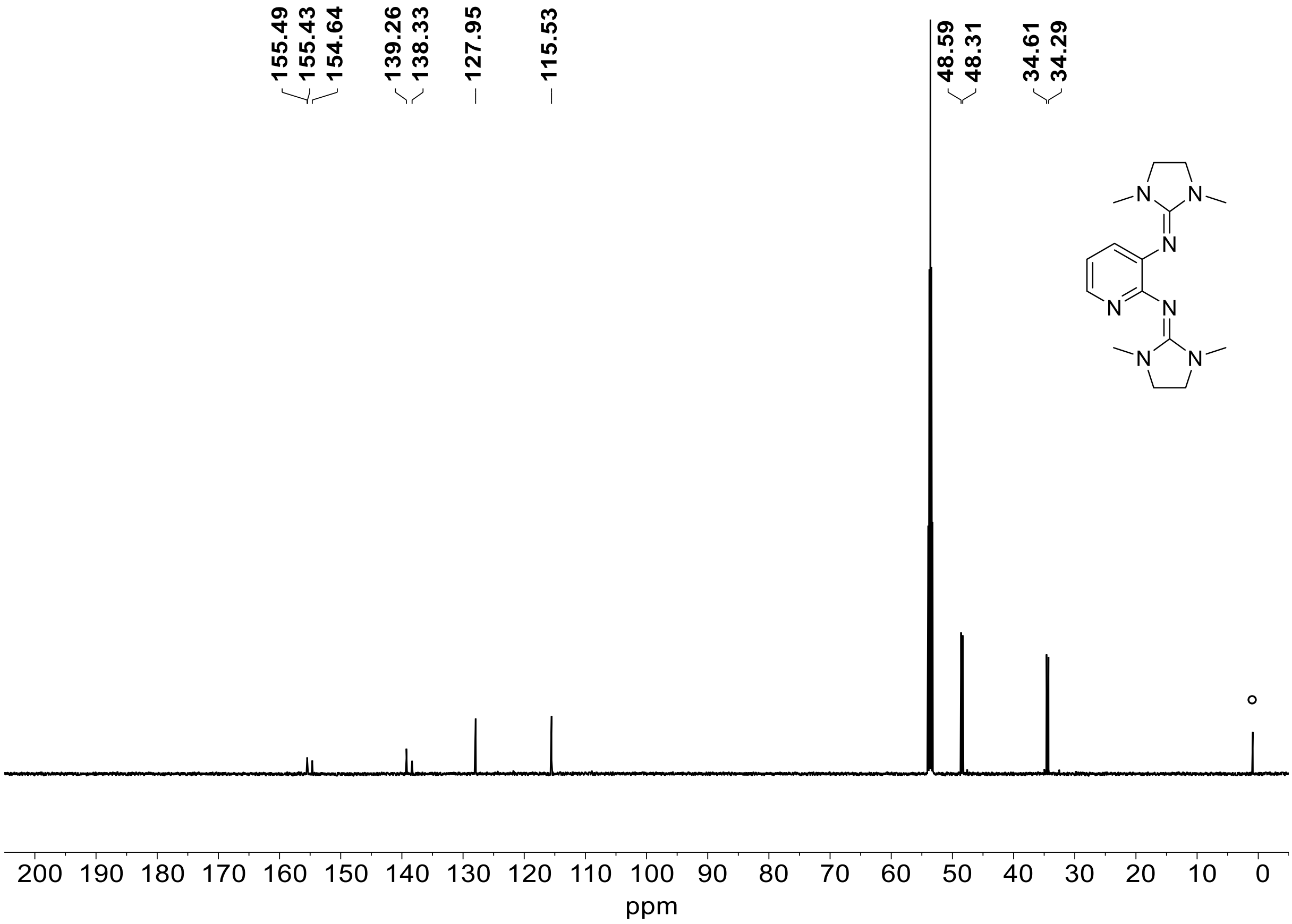

Figure S2: ^{13}C NMR spectrum of L2a, measured at 151 MHz in CD_2Cl_2, °grease.

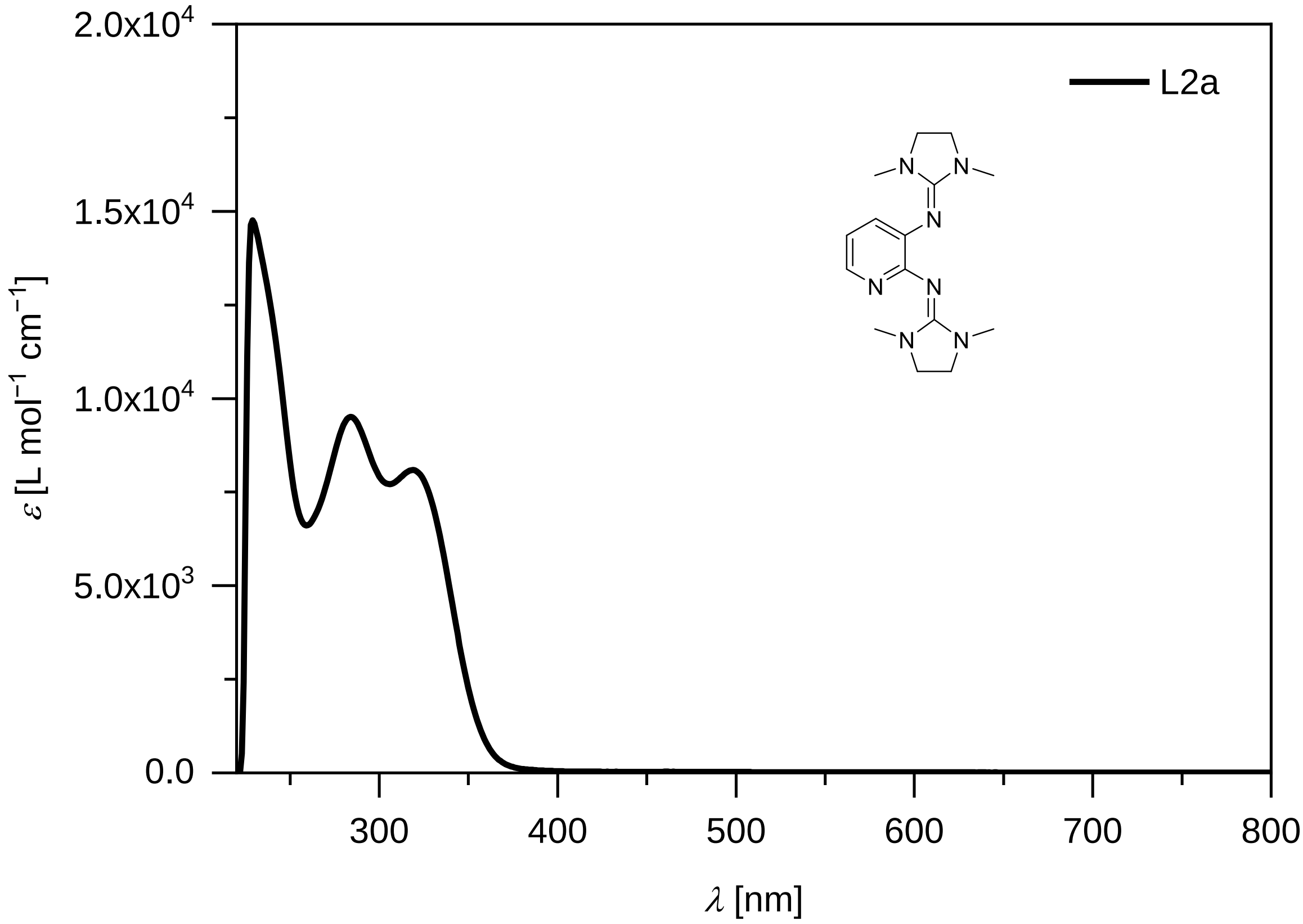

Figure S3: UV-vis absorption spectrum of L2a in dichloromethane at a concentration of $1.06 \cdot 10^{-4}$ mol L^{-1} at r.t.

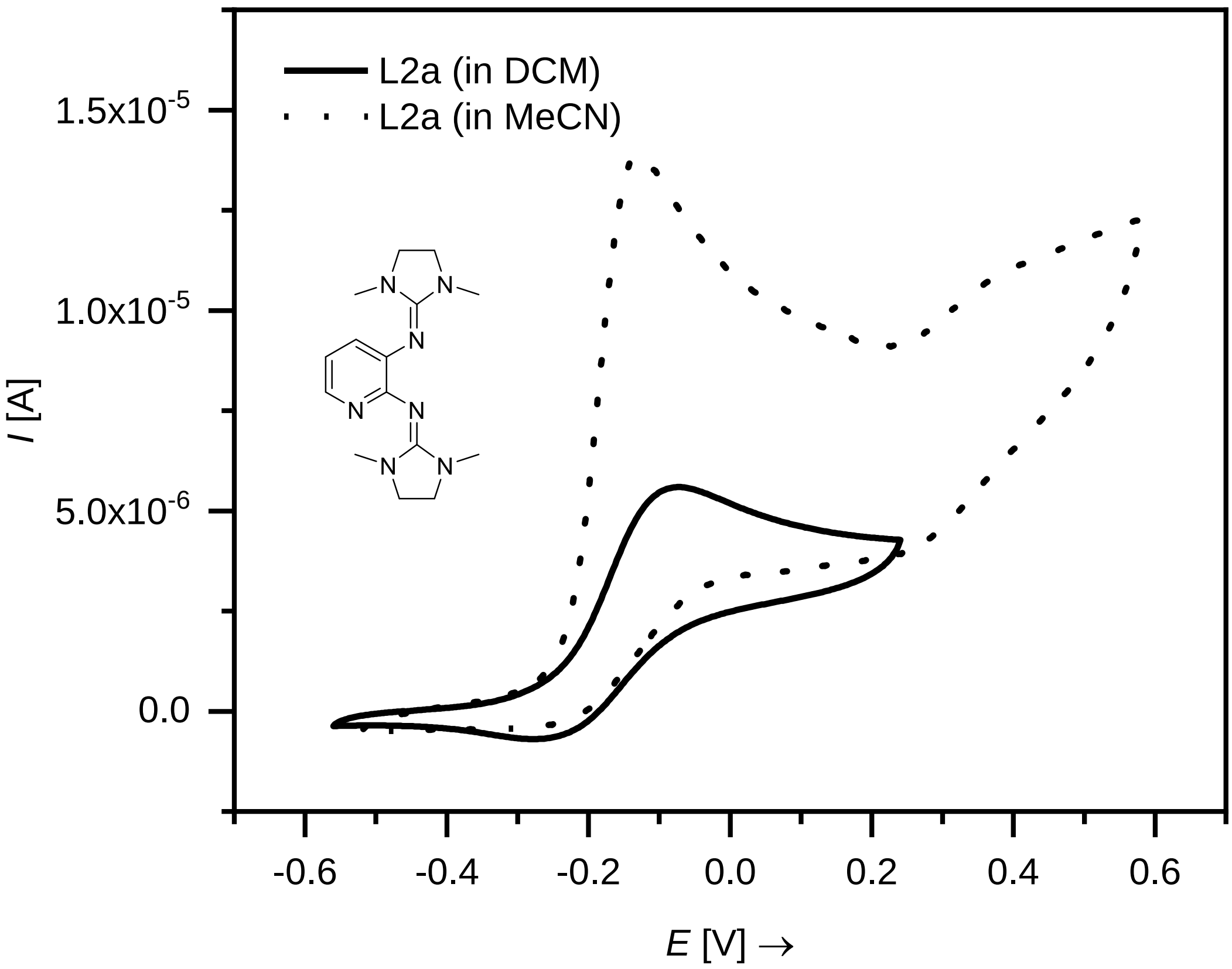

Figure S4: Cyclic voltammogram of L2a in dichloromethane and acetonitrile ([n-Bu_4N][PF_6], 20 mV s^{-1}, referenced to Fc/Fc^+).

2.1.2 L2b

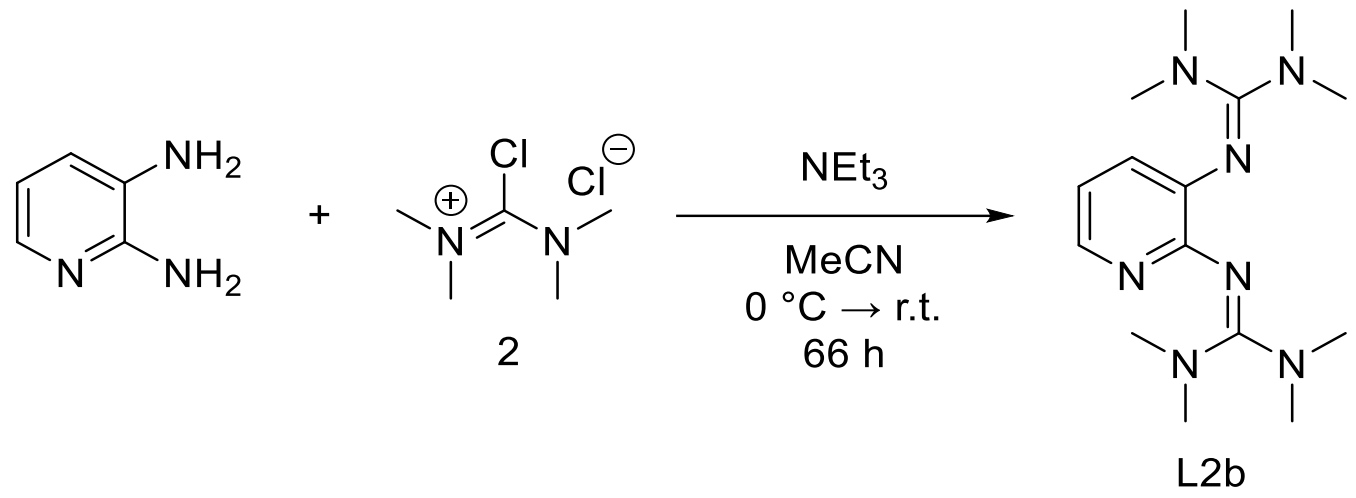

Pyridine-2,3-diamine (250 mg, 2.29 mmol, 1.00 eq.) and 2-chloro-1,1,3,3-tetramethylformamidinium chloride (2) (980 mg, 5.73 mmol, 2.50 eq.) were added to a Schlenk flask and dissolved in acetonitrile. The reaction mixture was stirred at 0 °C for 15 min before slowly adding triethylamine (1.28 mL, 927 mg, 9.16 mmol, 4.00 eq.). Then, the brown reaction mixture was stirred for additional 66 h while slowly warming up to room temperature. After removing the solvent under reduced pressure, the residue was solved with HCl_{aq} (5.0 mL, 10%) and $NaOH_{aq}$ (15.0 mL, 25%) was added while stirring. The aqueous phase was extracted with diethyl ether (5 x 10 mL) and dried over sodium sulphate. After removing the solvent, the residue was treated with HCl_{aq} (2.0 mL, 10%) and $NaOH_{aq}$ (6.0 mL, 25%), extracted with diethyl ether (3 · 10 mL) and dried over sodium sulphate again. The product was dried under reduced pressure, yielding L2b (206 mg, 674 µmol, 29%) as brown viscose liquid.

^{1}H NMR (600.15 MHz, CD_2Cl_2)

δ = 7.71-7.68 (m, 1H, CH_{py}), 6.62-6.57 (m, 2H, CH_{py}), 2.68-2.65 (m, 24H, CH_3) ppm.

^{13}C{^{1}H} NMR (150.93 MHz, CD_2Cl_2), **^{13}C-DEPT-135**

δ = 161.1 (1C, C_q), 159.5 (1C, C_q), 157.6 (1C, C_q), 140.1 (1C, C_q) 139.8 (1C, CH), 127.2 (1C, CH), 115.9 (1C, CH), 39.7 (4C, CH_3), 39.6 (4C, CH_3) ppm.

Mass spectrometry (EI^+)

m/z = 305.2410 ([L2b]$^+$, calc. 305.2328).

Mass spectrometry (ESI^+ in DCM)

m/z = 306.2401 ([L2b+H]$^+$, calc. 306.2401).

UV-vis (DCM) ($7.23 \cdot 10^{-5}$ mol L^{-1})

λ (ε) = 321 (shoulder, 8250), 292 (10700), 230 (17800) nm (M^{-1} cm^{-1}).

CV (DCM, [n-Bu_4N][PF_6], 20 mV s^{-1}, referenced to Fc/Fc^+)

E_{Ox} = −45.0 mV.

CV (MeCN, [n-Bu_4N][PF_6], 20 mV s^{-1}, referenced to Fc/Fc^+)

E_{Ox} = −46.5 mV.

Elemental analysis (C, H, N in %) ($C_{15}H_{27}N_7 \cdot 0.3$ eq. Et_2O)

calc.: C 59.38, H 9.23, N 29.92.

found: C 58.97, H 9.27, N 29.05.

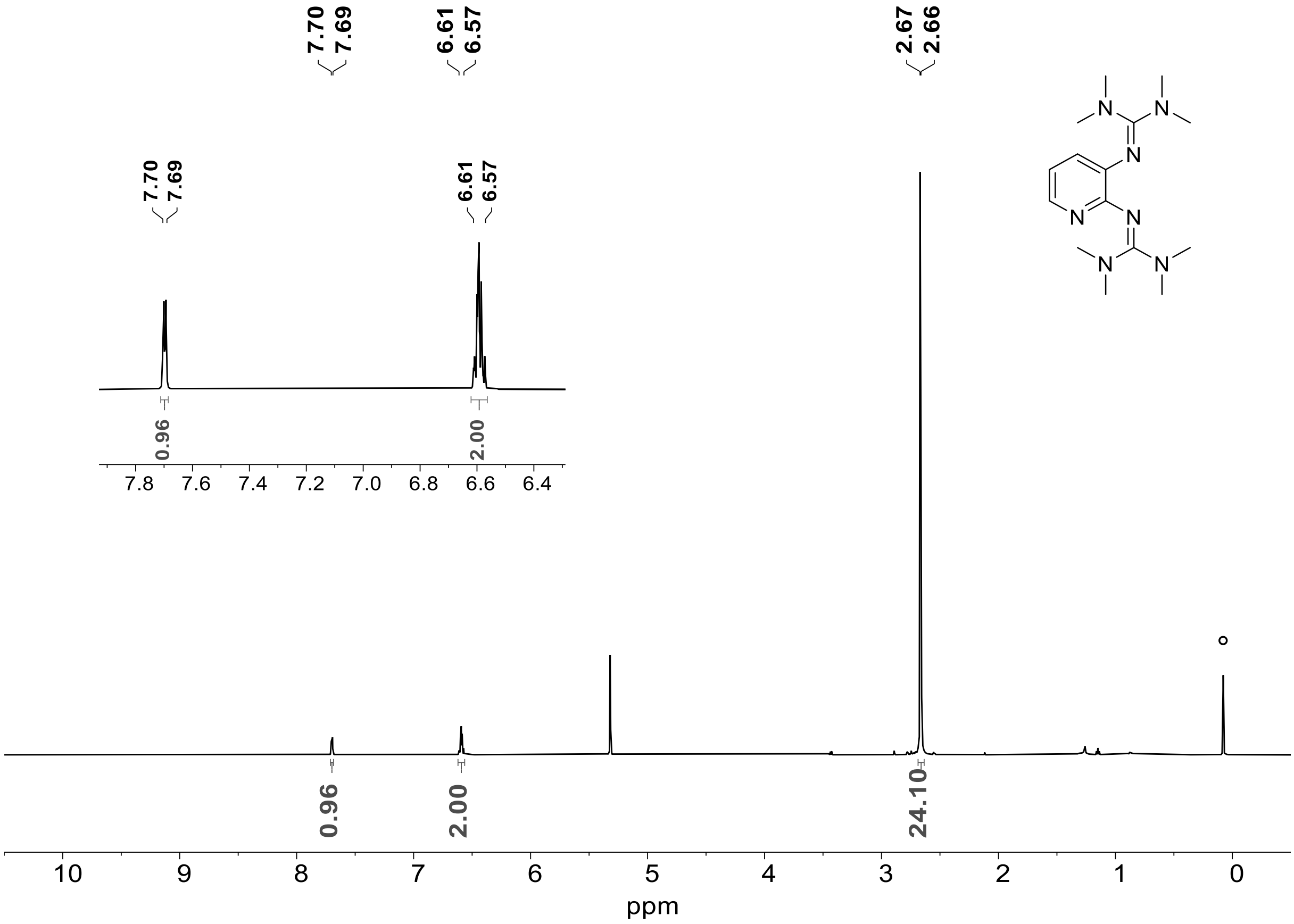

Figure S5: ^{1}H NMR spectrum of L2b, measured at 600 MHz in CD_2Cl_2, °grease.

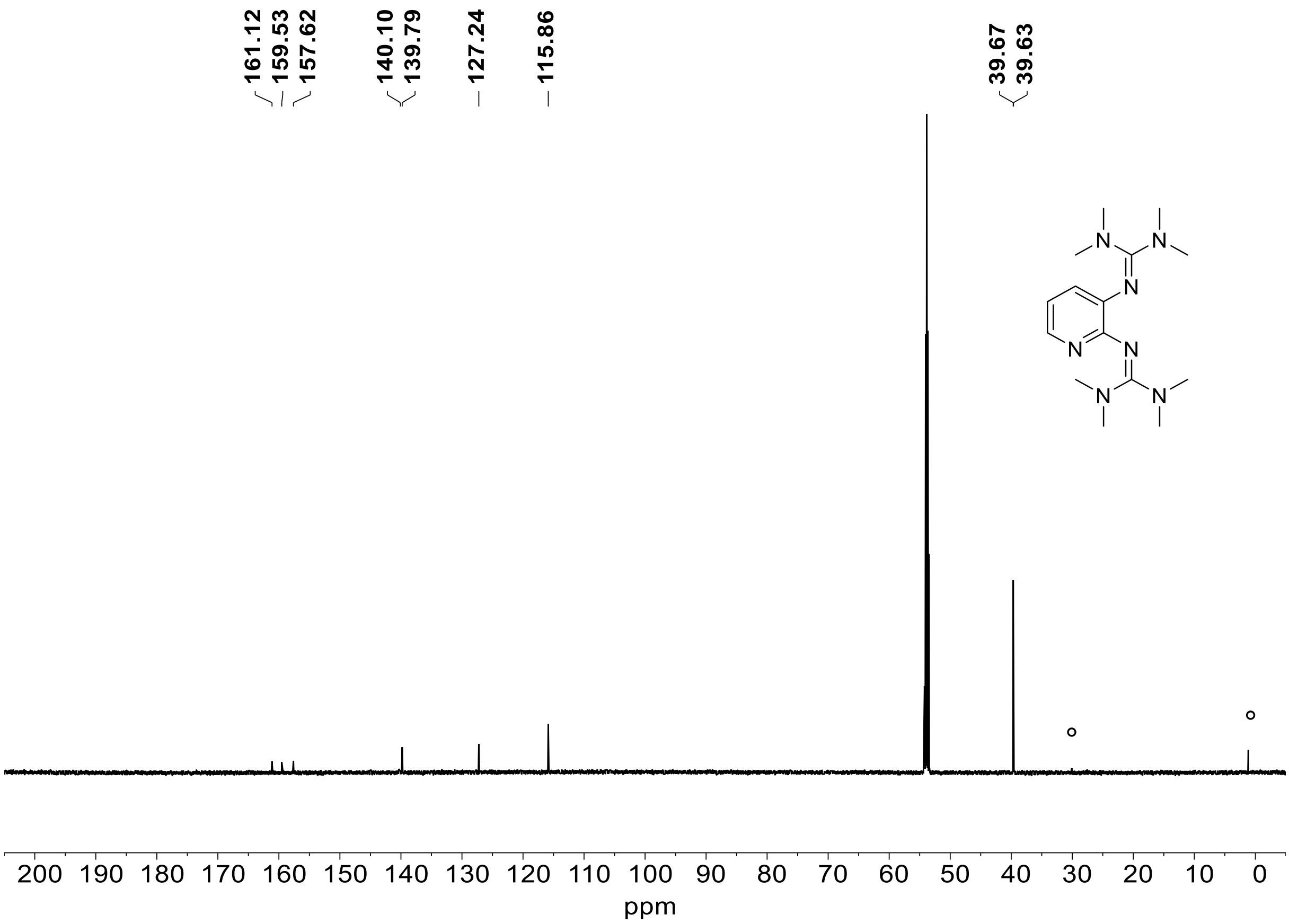

Figure S6: ^{13}C NMR spectrum of L2b, measured at 151 MHz in CD_2Cl_2, °grease.

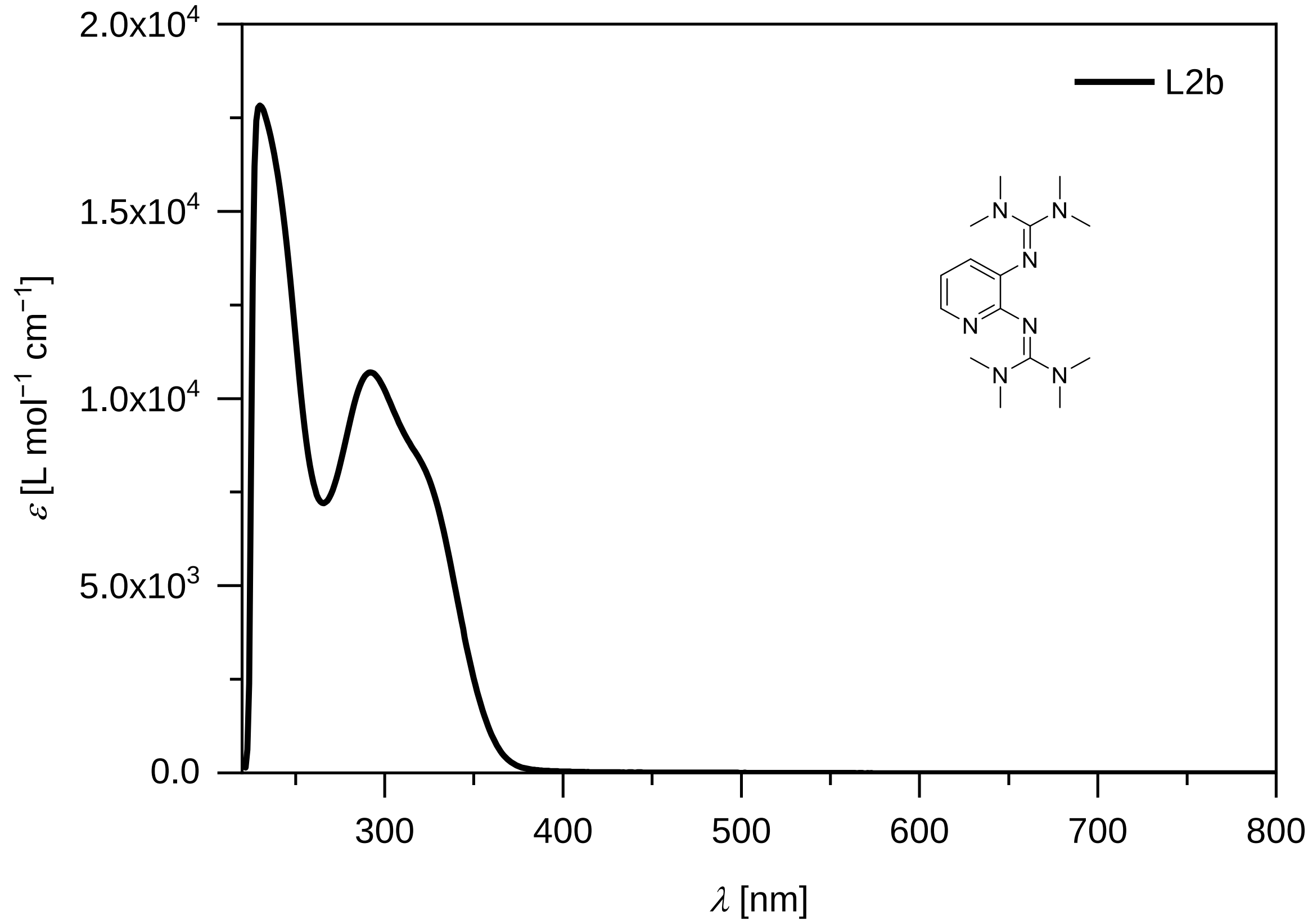

Figure S7: UV-vis absorption spectrum of L2b in dichloromethane at a concentration of $7.23 \cdot 10^{-5}$ mol L^{-1} at r.t.

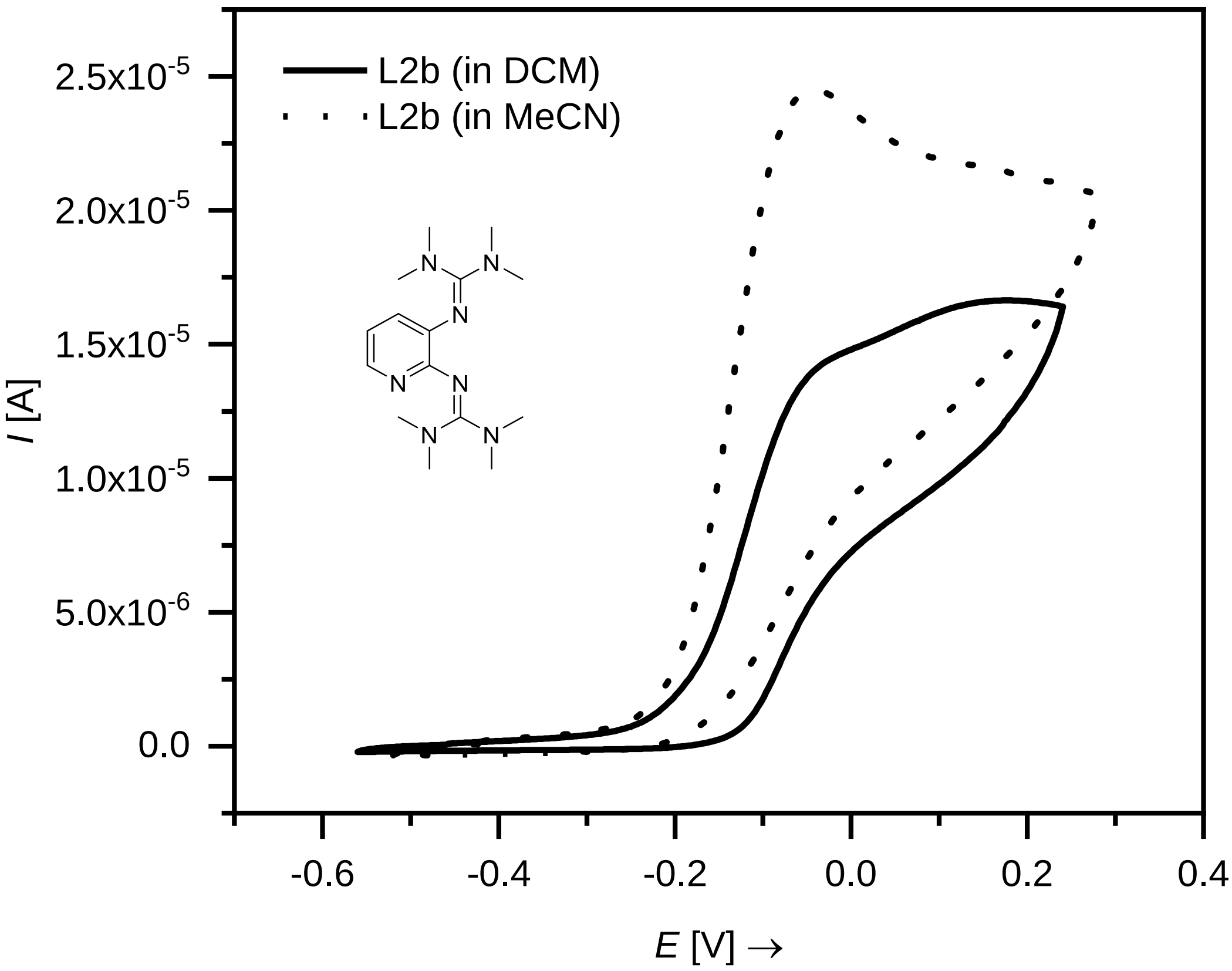

Figure S8: Cyclic voltammogram of L2b in dichloromethane and acetonitrile ([n-Bu_4N][PF_6], 20 mV s^{-1}, referenced to Fc/Fc^+).

2.1.3 (L2b+2H)Cl_2

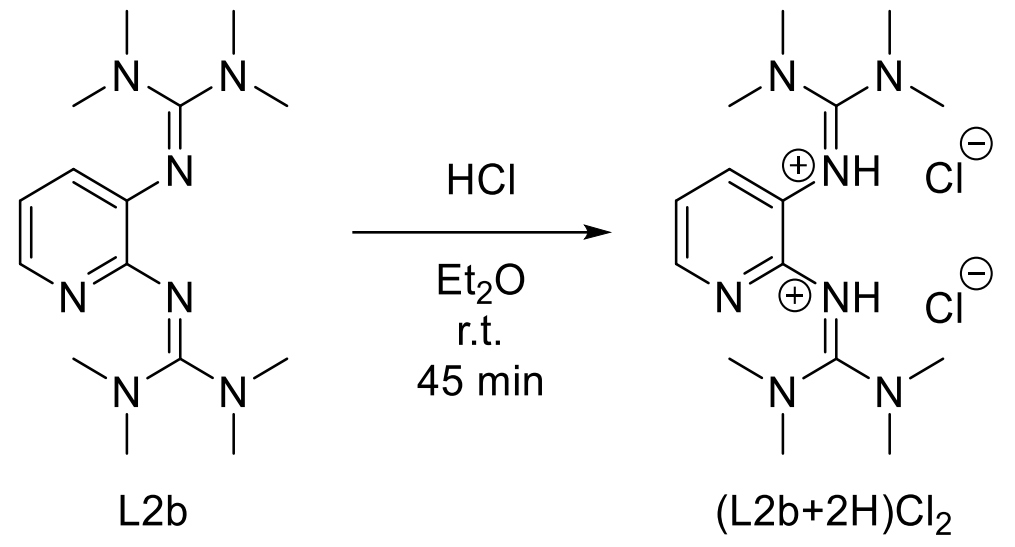

L2b (20.0 mg, 65.5 µmol, 1.00 eq.) was dissolved in diethyl ether (3 mL) and HCl in diethyl ether (2.00 M, 63.8 µL, 4.66 mg, 128 µmol, 1.95 eq.) was added dropwise. The reaction mixture was stirred at r.t. for 45 min. Subsequently the pale, orange precipitate was filtered off and dried. The product (L2b+2H)Cl_2 (23.1 mg, 61.1 µmol, 93%) was obtained as pale orange solid.

A crystal for single crystal structure analysis was obtained from an acetonitrile solution by diffusing with diethyl ether.

^{1}H NMR (600.15 MHz, CD_3CN)

δ = 11.62 (s, 1H), 11.45 (s, 1H), 8.19 (dd, J = 4.7, 1.6, 1H), 7.36 (dd, J = 8.0, 1.7, 1H), 7.22 (dd, J = 8.0, 4.7, 1H), 2.98 (d, J = 15.9, 24H).

^{13}C{^{1}H} NMR (150.93 MHz, CD_3CN), **^{13}C-DEPT-135**

δ = 159.5 (1C, C_q), 159.2 (1C, C_q), 146.0 (1C, CH), 144.7 (1C, C_q), 132.1 (1C, CH), 125.9 (1C, C_q), 121.7 (1C, CH), 40.9 (8C, CH_3).

Mass spectrometry (ESI$^+$ in DCM)

m/z = 306.2397 ([L2b+H]$^+$, calc. 306.2401).

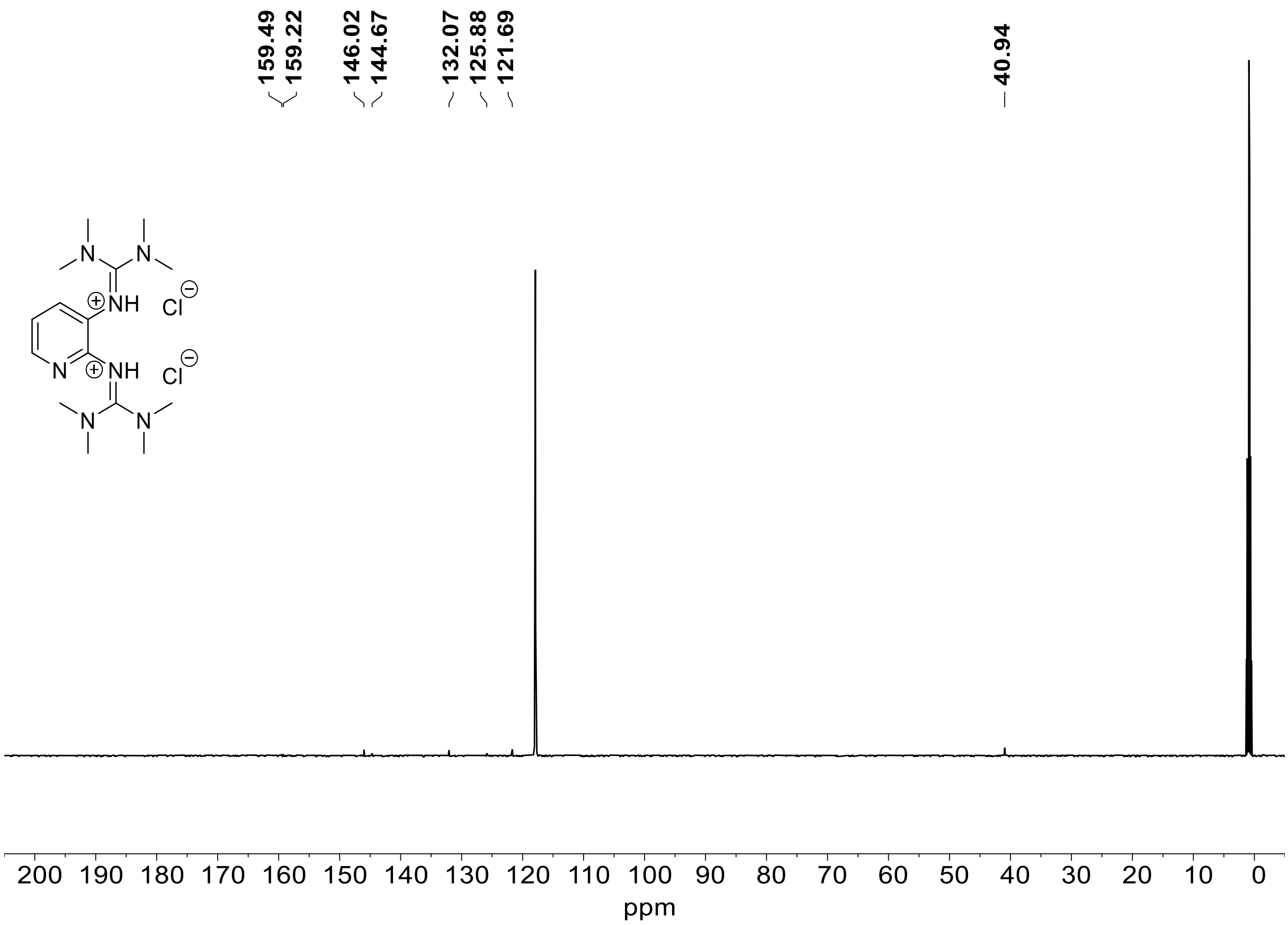

Figure S9: ^{13}C NMR spectrum of (L2b+2H)Cl_2, measured at 151 MHz in CD_3CN.

2.1.4 L3

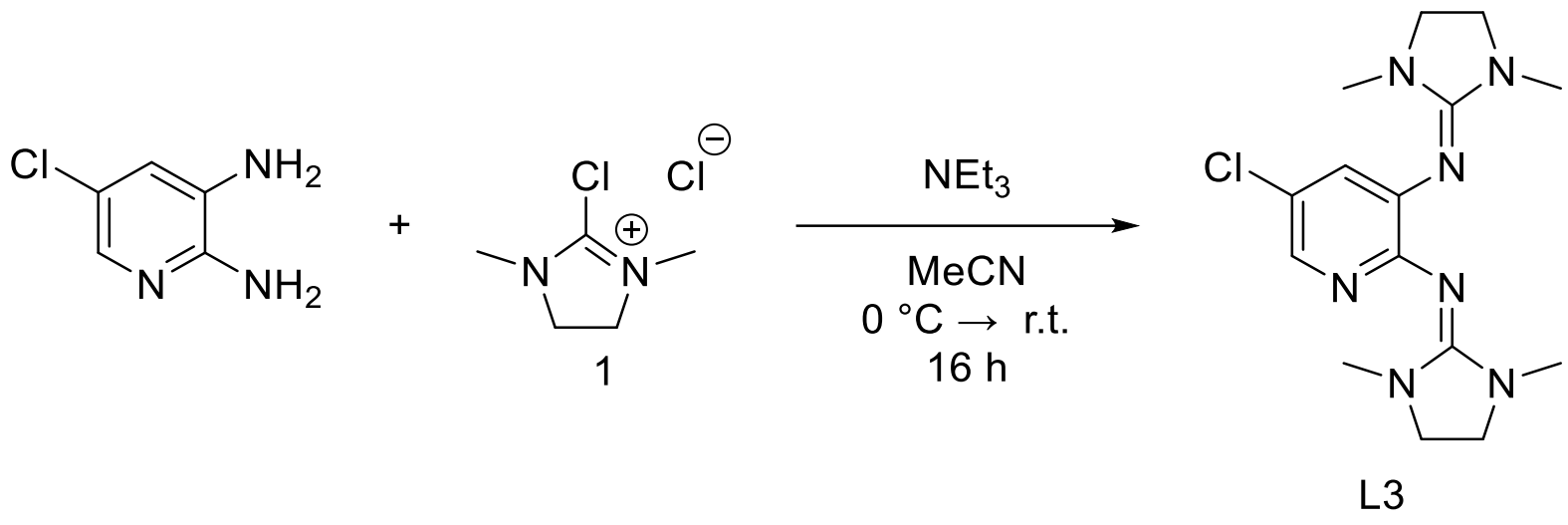

5-Chloropyridine-2,3-diamine (195 mg, 1.36 mmol, 1.00 eq.) was dissolved in acetonitrile (10 ml) and 2-chloro-1,3-dimethylimidazolinium chloride (1) in acetonitrile (1 M, 3.20 mL, 541 mg, 3.20 mmol, 2.36 eq.) was added. The mixture was stirred at 0 °C for 15 minutes before adding triethylamine (770 µL, 562 mg, 5.55 mmol, 4.09 eq.) dropwise over five minutes. Slowly warming up, the mixture was stirred for 16 hours. After filtration, the solvent was removed from the filtrate *in vacuo,* and the residue was stirred vigorously with degassed $NaOH_{aq}$ (25%, 10.0 mL). The aqueous phase was extracted with diethyl ether (5 x 10 mL). The organic phase was dried over $NaSO_4$, filtered and evaporated *in vacuo* to obtain a brown oil. Due to an incomplete reaction the process was repeated with the resulting oil in acetonitrile (10 ml), 1 in acetonitrile (1 M, 1.70 mL, 287 mg, 1.70 mmol, 1.25 eq.) and triethylamine (380 µL, 277 mg, 2.74 mmol, 2.02 eq.) yielding a brown oil after extraction. The product was further purified by first dissolving the oil in toluene (5 mL) in an ultrasound bath, removing the solvent under reduced pressure and second dissolving the oil in diethyl ether (5 mL) in an ultrasound bath and removing the solvent under reduced pressure yielding L3 (238 mg, 709 µmol, 52%) as a brown oil.

A crystal for single crystal structure analysis was obtained from a dichloromethane solution by layering with pentane.

^{1}**H NMR** (400 MHz, CD_3CN)

δ = 7.59 (d, *J* = 2.5 Hz, 1H, CH^{Py}), 6.83 (d, *J* = 2.6 Hz, 1H, CH^{Py}), 3.27 (s, 4H, CH_2), 3.23 (s, 4H, CH_2), 2.58 – 2.57 (m, 12H, CH_3) ppm.

^{13}**C NMR** (101 MHz, CD_3CN) ^{13}**C-DEPT-135**

δ = 156.2 (1C, C_q), 155.8 (1C, C_q), 155.3 (1C, C_q), 140.8 (1C, C_q), 136.8 (1C, CH), 127.1 (1C, CH), 122.3 (1C, C_q), 48.9 (2C, CH_2), 48.7 (2C, CH_2), 34.7 (2C, CH_3), 34.5 (2C, CH_3) ppm.

Mass spectrometry (ESI^+ in DCM)

m/*z* = 336.1693 $(L3+H]^+$, calc. 336.1698).

UV-vis (DCM) ($4.80 \cdot 10^{-5}$ mol L^{-1})

λ (ε) = 334 (7340), 289 (9110), 228 (15000) nm (M^{-1} cm^{-1}).

CV (DCM, [*n*-Bu_4N][PF_6], 20 mV s^{-1}, referenced to Fc/Fc^+)

E_{Ox}^1 = −42.7 mV, E_{Ox}^2 = 206 mV, E_{Ox}^3 = 597 mV.

E_{Red}^1 =−228 mV, E_{Red}^2 = 155 mV.

Elemental analysis (C, H, N in %) ($C_{15}H_{22}N_7Cl$)

calc.: C 53.65, H 6.60, N 29.20.

found: C 53.35, H 6.54, N 28.38.

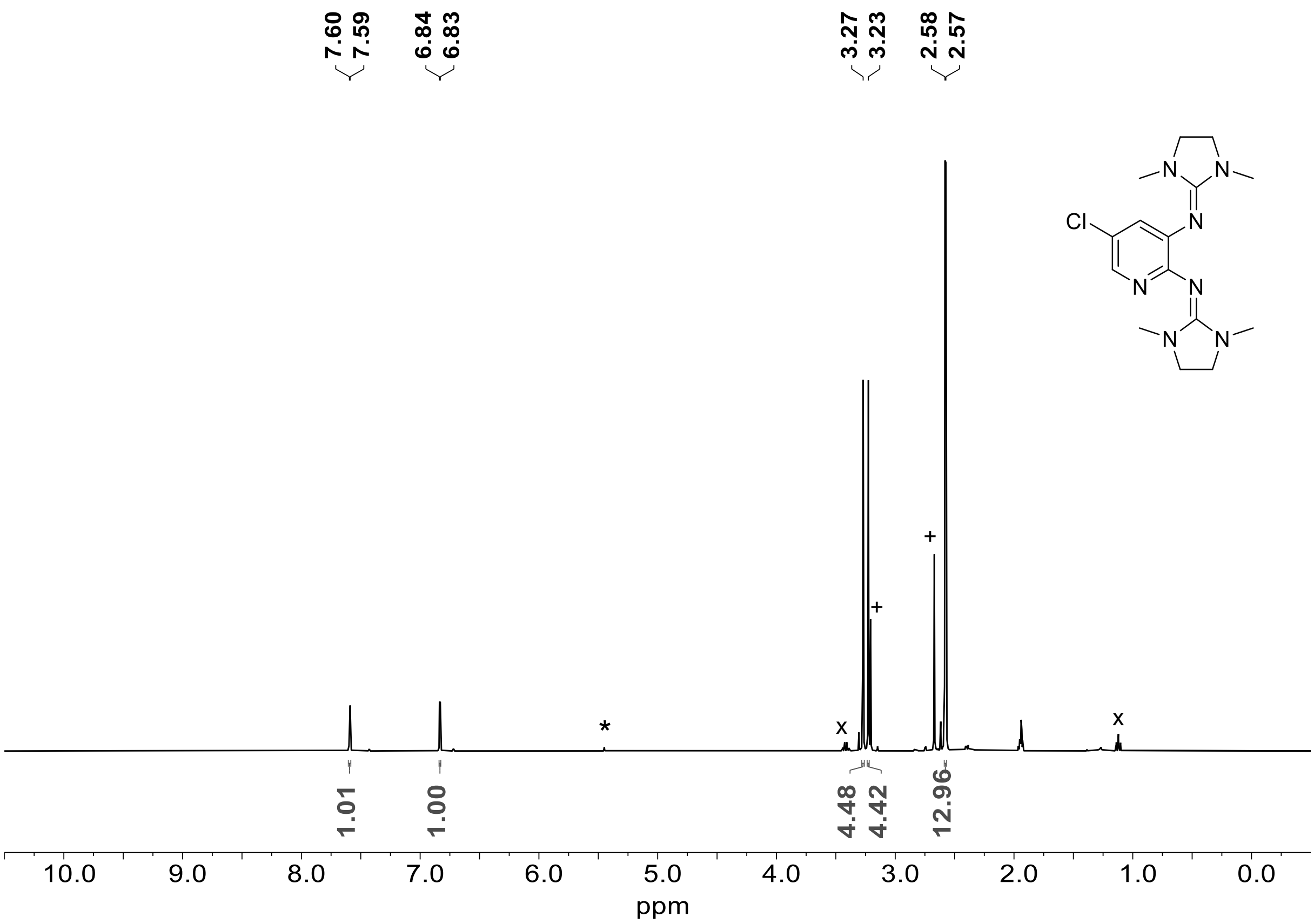

Figure S10: ^{1}H NMR spectrum of L3 measured at 400 MHz in CD_3CN, *DCM, ^{x}diethyl ether, $^+$deactivated 1.

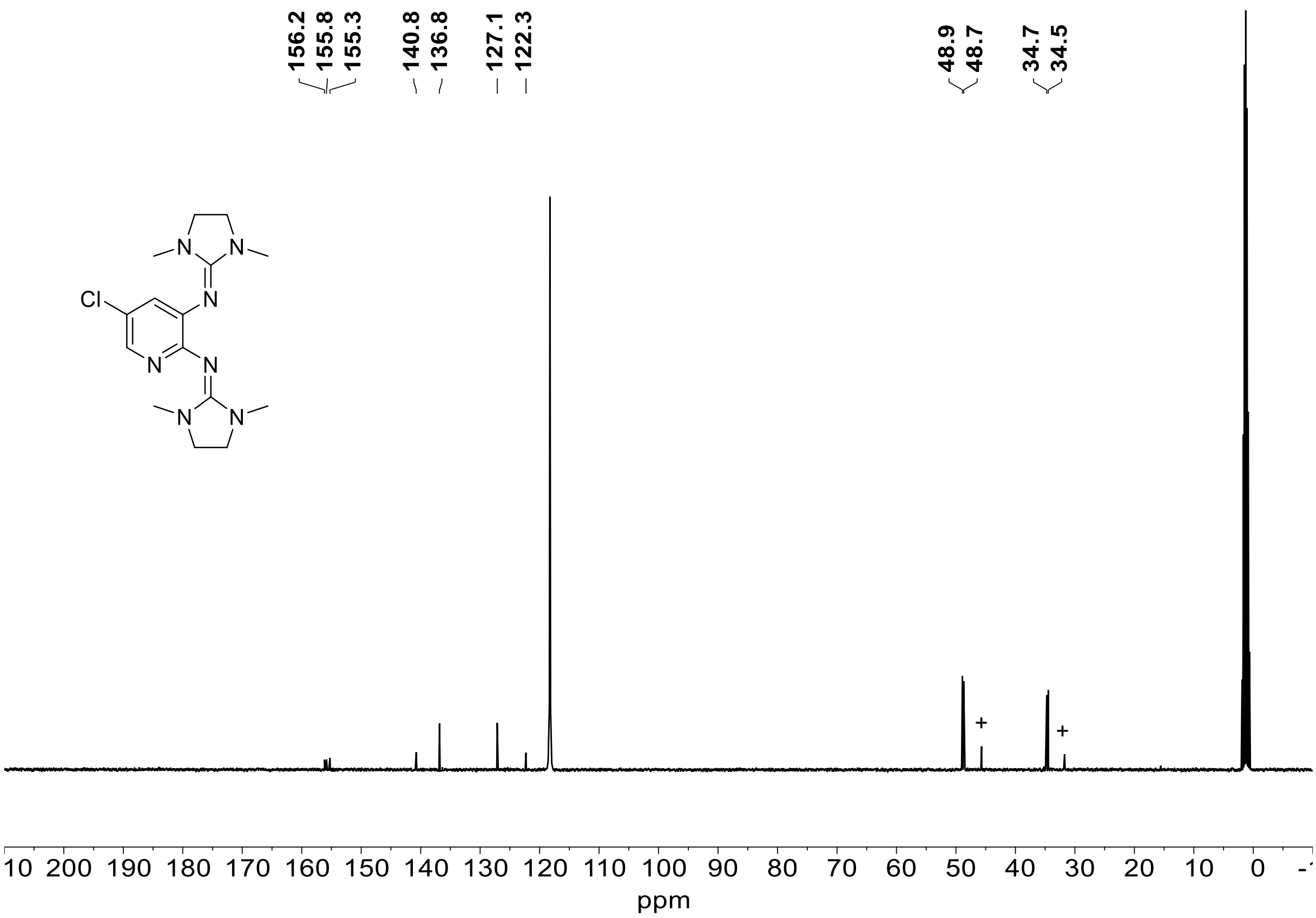

Figure S11: ^{13}C NMR spectrum of L3, measured at 101 MHz in CD_3CN, $^+$deactivated 1.

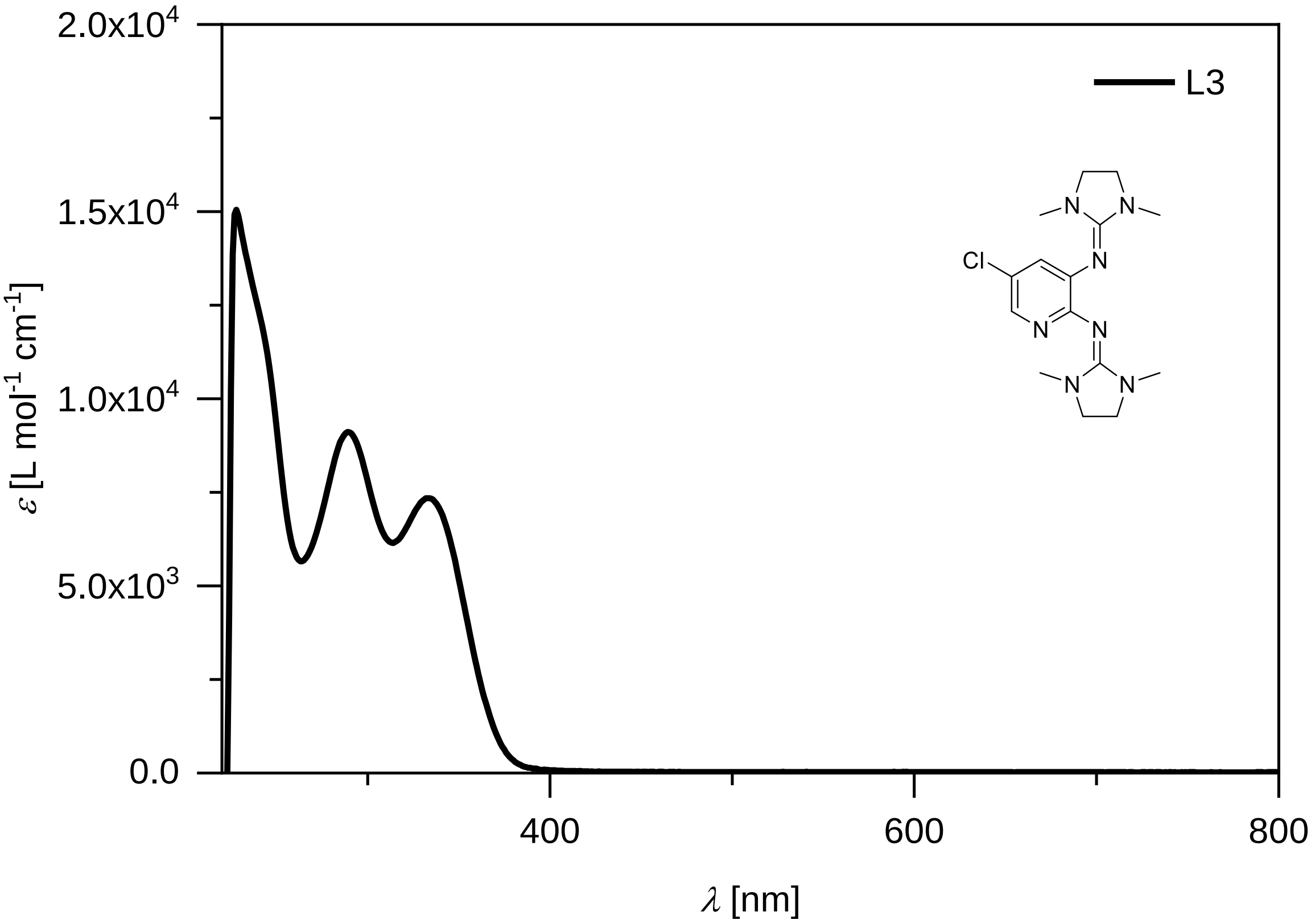

Figure S12: UV-vis absorption spectrum of L3 in dichloromethane at a concentration of $4.80 \cdot 10^{-5}$ mol L^{-1} at rt.

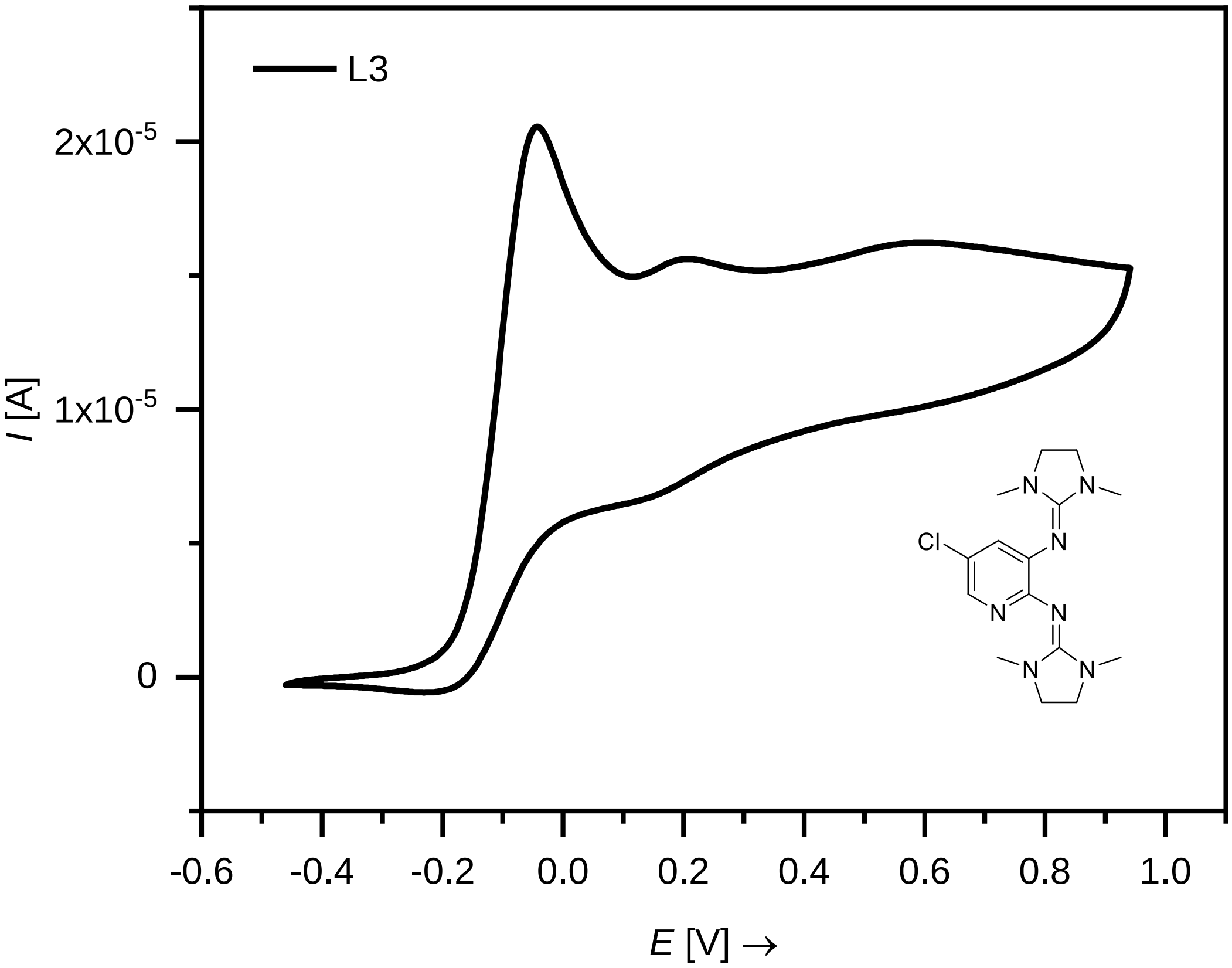

Figure S13: Cyclic voltammogram of L3 in dichloromethane ([n-Bu_4N][PF_6], 20 mV s^{-1}, referenced to Fc/Fc^+).

2.1.5 L4

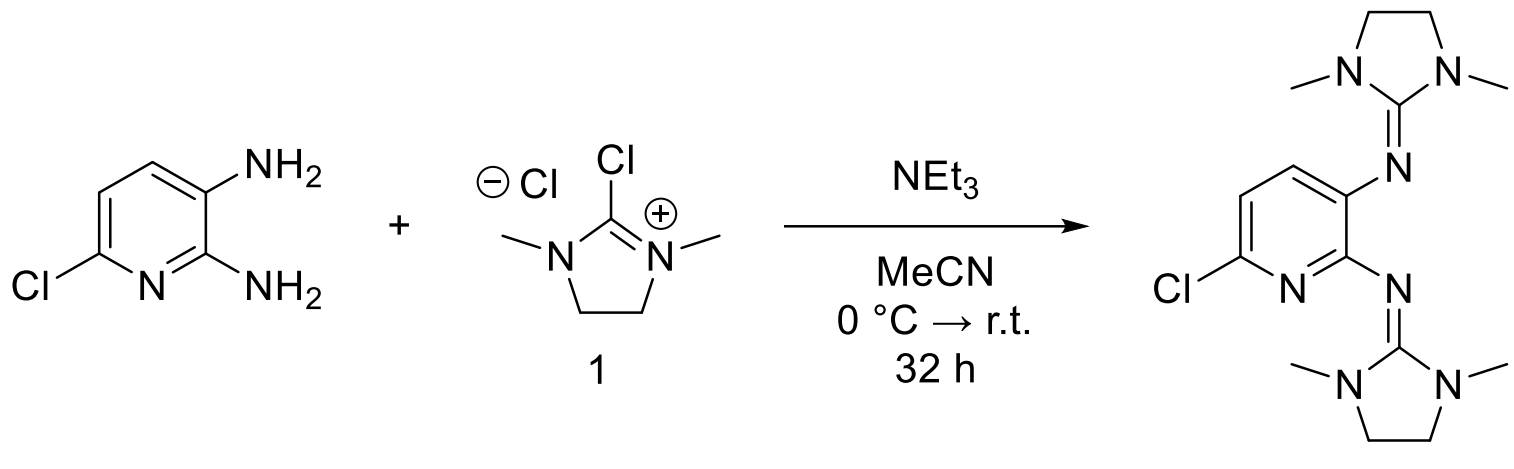

6-Chloropyridine-2,3-diamine (200 mg, 1.39 mmol, 1.00 eq.) was dissolved in acetonitrile (10 ml) and 2-chloro-1,3-dimethylimidazolinium chloride (1) in acetonitrile (1 M, 3.06 mL, 518 mg, 3.06 mmol, 2.20 eq.) was added. The mixture was stirred at 0 °C for 15 min before adding triethylamine (777 µL, 564 mg, 5.57 mmol, 4.00 eq.) dropwise over five minutes. Slowly warming up, the mixture was stirred for 32 hours. After filtration, the solvent was removed from the filtrate *in vacuo,* and the residue was stirred vigorously with degassed $NaOH_{aq}$ (25%, 10 mL). The aqueous phase was extracted with diethyl ether (3·10 mL) and the organic phase was dried over $NaSO_4$, filtered and evaporated *in vacuo* to obtain a light brown solid. Due to an incomplete reaction, the process was repeated with the resulting solid (280 mg) in acetonitrile (10 ml), 1 in acetonitrile (1 M, 975 µL, 165 mg, 975 mmol, 0.70 eq.) and triethylamine (272 µL, 197 mg, 4.47 mmol, 1.40 eq.) yielding a colourless solid in yellow oil after extraction. The product was further purified by dissolving the mixture in toluene (5 mL) in an ultrasound bath and removing the solvent under reduced pressure. Diethyl ether (10 mL) was added, and the resulting solid was filtered off and dried, yielding the product L4 (140 mg, 417 mmol, 30%) as a colourless solid.

A crystal for single crystal structure analysis was obtained from a dichloromethane solution by layering with pentane.

^{1}H NMR (400 MHz, CD_3CN)

δ = 6.80 (d, J = 7.8 Hz, 1H, CH), 6.57 (d, J = 7.8 Hz, 1H, CH), 3.30 (s, 4H, CH_2), 3.20 (s, 4H, CH_2), 2.60 (s, 6H, CH_3), 2.57 (s, 6H, CH_3) ppm.

^{13}C{^{1}H} NMR (101 MHz, CD_3CN), **^{13}C-DEPT-135**

δ = 156.7 (1C, C_q), 155.9 (1C, C_q), 155.6 (1C, C_q), 139.1 (1C, C_q), 138.5 (1C, C_q), 130.9 (4C, CH), 114.6 (4C, CH), 49.0 (4C, CH_2), 48.7 (4C, CH_2), 34.8 (4C, CH_3), 34.5 (4C, CH_3) ppm.

Mass spectrometry (ESI^+ in DCM)

m/z = 336.1694 ($[L4+H]^+$, calc. 336.1698).

UV-vis (DCM) ($4.76 \cdot 10^{-5}$ mol L^{-1})

λ (ε) = 335 (9650), 289 (12200), 229 (17400) nm (M^{-1} cm^{-1}).

CV (DCM, [n-Bu_4N][PF_6], 20 mV s^{-1}, referenced to Fc/Fc^+)

$E_{1/2}{}^{1}$ = −111 mV, $E_{1/2}{}^{2}$ = 222 mV.
Potential estimated due to low intensity (Figure S17).

Elemental analysis (C, H, N in %) ($C_{15}H_{22}N_7Cl$)

calc.: C 53.65, H 6.60, N 29.20.

found: C 53.72, H 5.90, N 29.00.

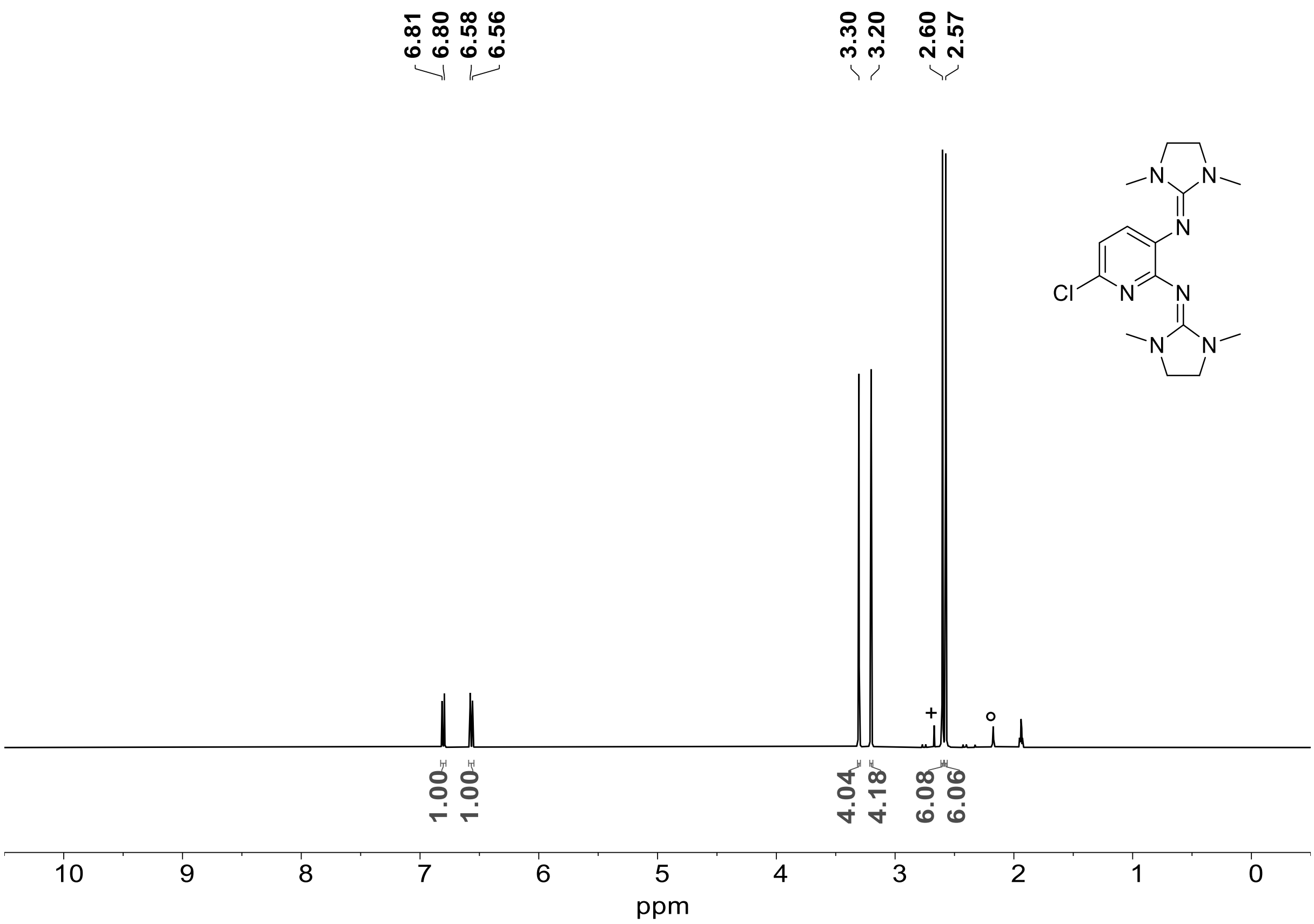

Figure S14: ^{1}H NMR spectrum of L4 measured at 400 MHz in CD_3CN, °water, $^{+}$deactivated 1.

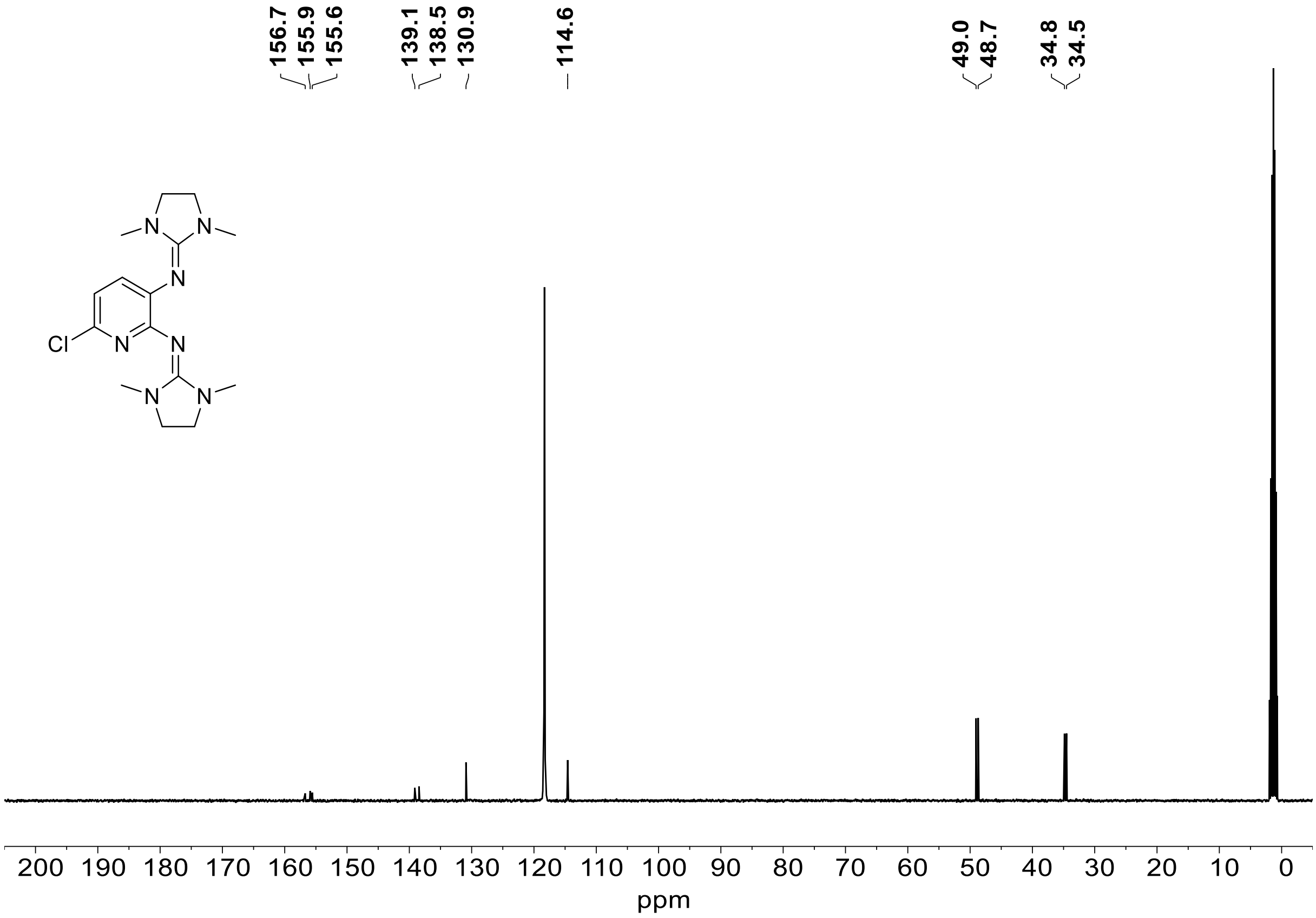

Figure S15: ^{13}C NMR spectrum of L4, measured at 101 MHz in CD_3CN.

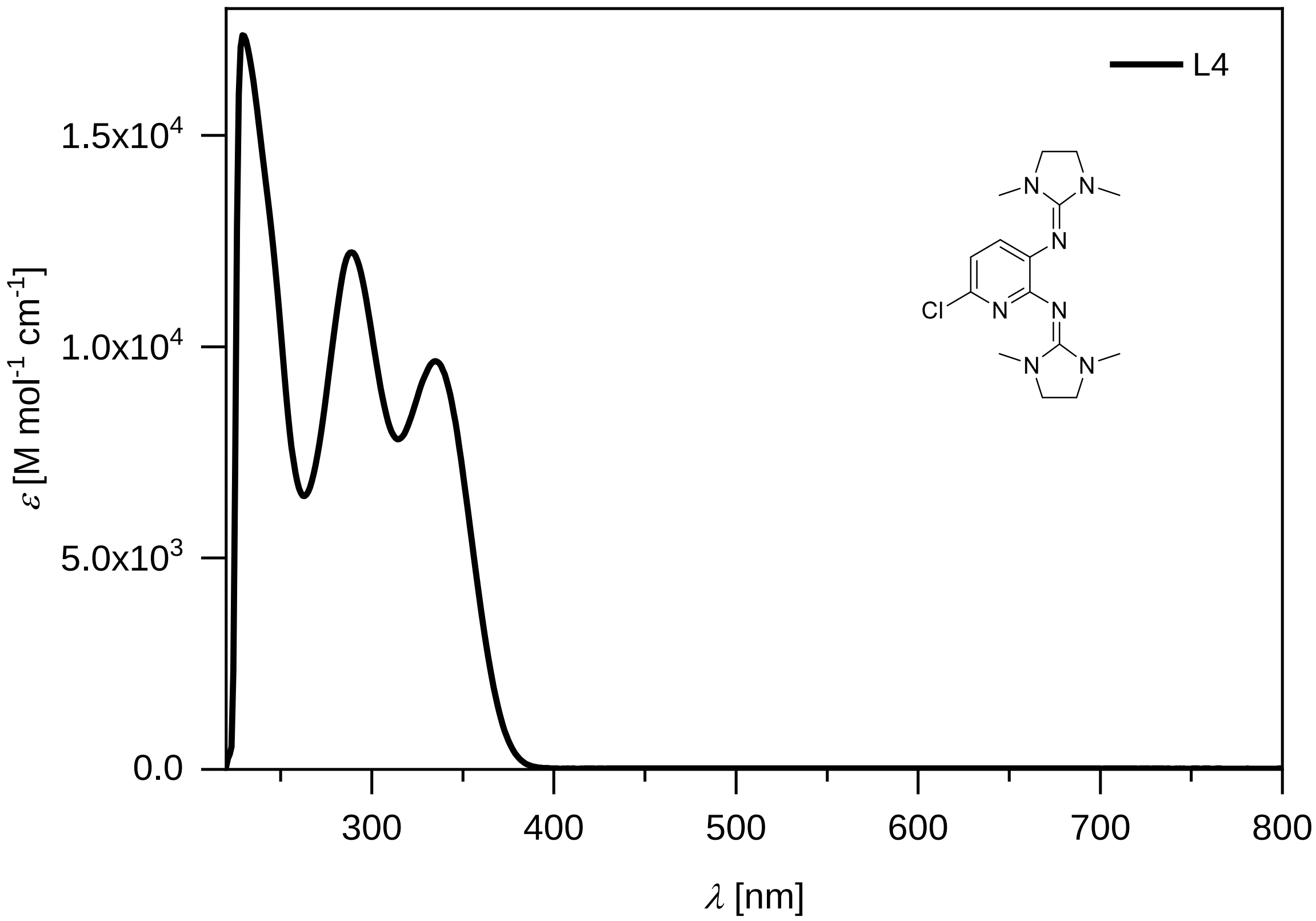

Figure S16: UV-vis absorption spectrum of L4 in dichloromethane at a concentration of $4.76 \cdot 10^{-5}$ mol L^{-1} at rt.

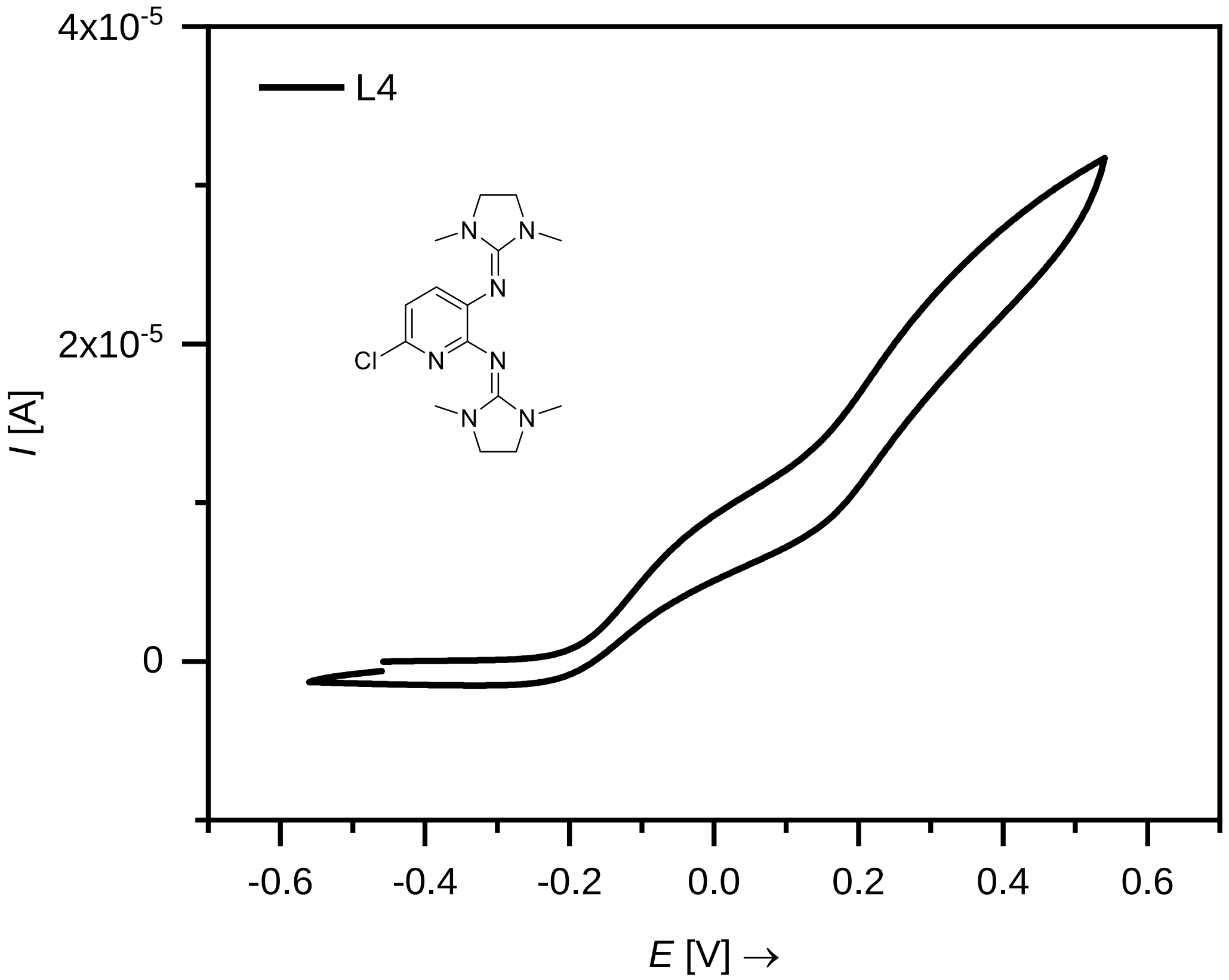

Figure S17: Cyclic voltammogram of L4 in dichloromethane ([n-Bu_4N][PF_6], 20 mV s^{-1}, referenced to Fc/Fc^+).

2.1.6 (L4+2H)Cl_2

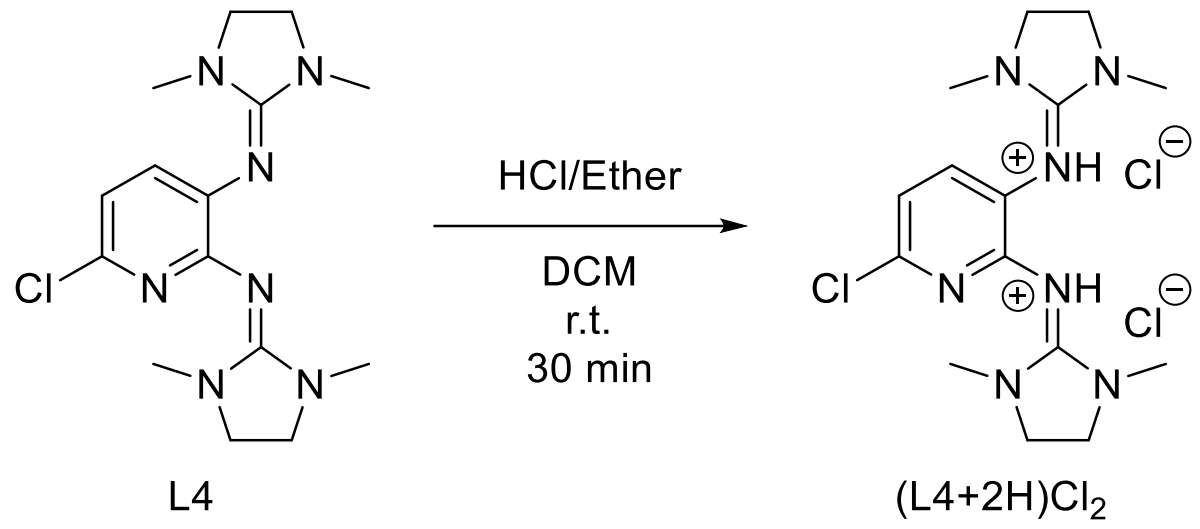

L4 (20.0 mg, 59.6 μmol, 1.00 eq.) was solved in dichloromethane (4 mL) and HCl in diethyl ether (2.00 M, 89.3 μL, 6.51 mg, 179 μmol, 3.00 eq.) was added dropwise. The reaction mixture was stirred at rt for 30 min. Subsequently the solvent was removed and the residue was dried. The product (L4+2H)Cl_2 (23.9 mg, 58.5 μmol, 98%) was obtained as colourless solid.

^{1}H NMR (400 MHz, CD_3CN)

δ = 11.65 (s, 1H, NH), 11.46 (s, 1H, NH), 7.65 (d, J = 8.4 Hz, 1H, CH), 7.31 (d, J = 8.4 Hz, 1H, CH), 3.86 (s, 4H, CH_2), 3.77 (s, 4H, CH_2), 2.98 (s, 6H, CH_3), 2.90 (s, 6H, CH_3) ppm.

Mass spectrometry (ESI$^+$ in DCM)

m/z = 336.1694 ([L4+H]$^+$, calc. 336.1698).

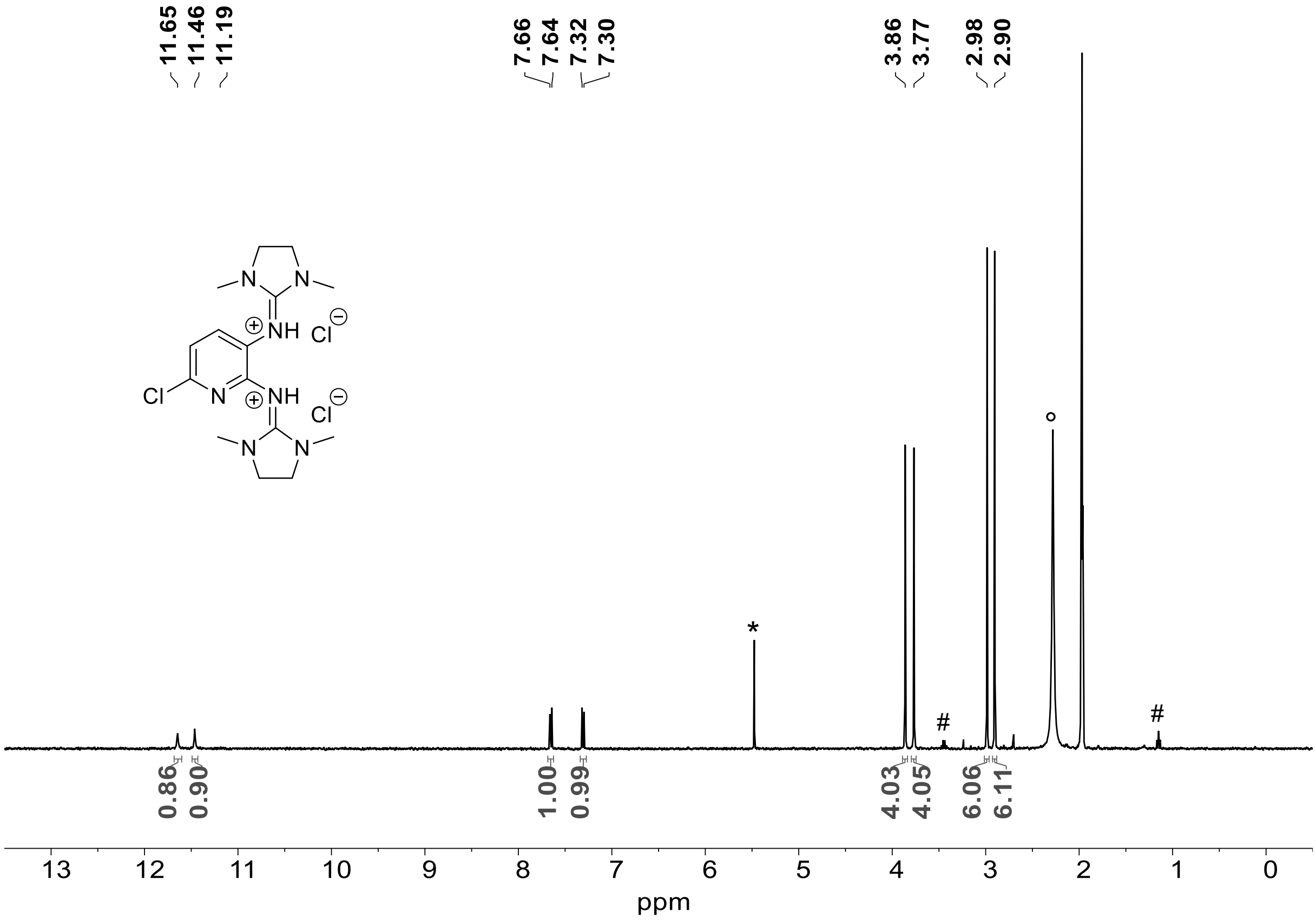

Figure S18: ^{1}H NMR spectrum of $(L4+2H)Cl_2$ measured at 400 MHz in CD_3CN, *dichloromethane, #diethyl ether, °water.

2.1.7 L5a

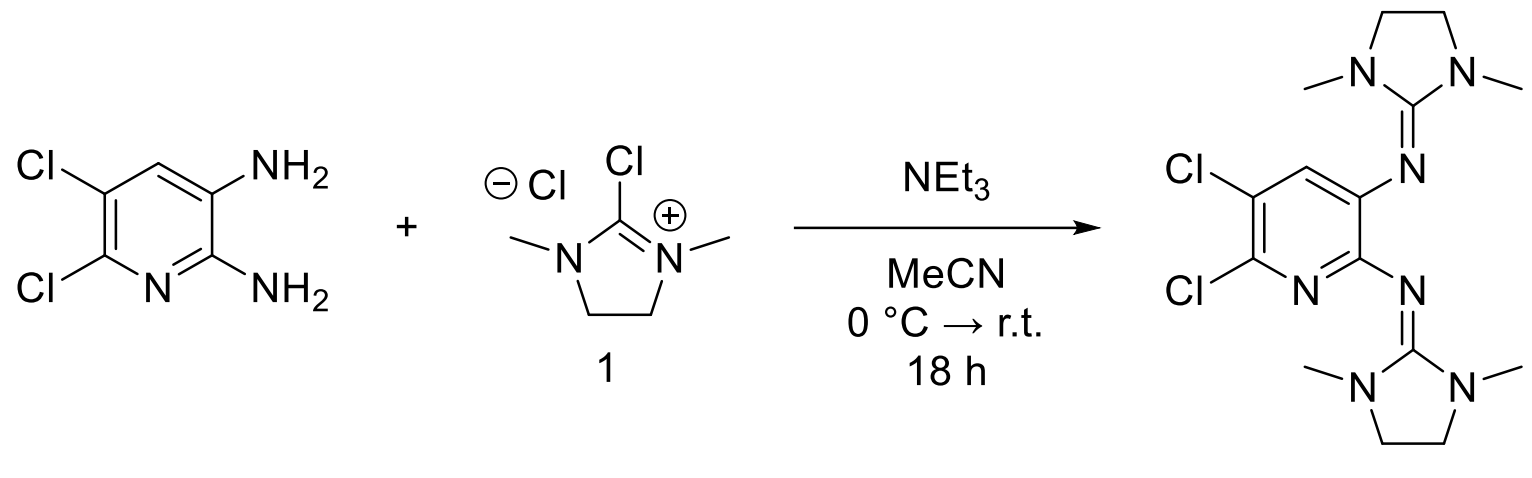

5,6-Dichloropyridine-2,3-diamine (400 mg, 2.25 mmol, 1.00 eq.) was added to a Schlenk flask and dissolved in acetonitrile. A solution of 2-chloro-1,3-dimethylimidazolinium chloride (1) in acetonitrile (1 M, 4.94 mL, 836 mg, 4.94 mmol, 2.20 eq.) was added and the reaction mixture was stirred at 0 °C for 15 min before slowly adding triethylamine (1.25 mL, 910 mg, 8.99 mmol, 4.00 eq.). The brown reaction mixture was then stirred for additional 18 h while slowly warming up to room temperature. After removing the solvent under reduced pressure, the residue was suspended in $NaOH_{aq}$ (10.0 mL, 25%) while stirring. The aqueous phase was extracted with dichloromethane (4 · 10 mL) and dried over sodium sulphate. The solvent was removed and the raw product was washed with diethyl ether (10 mL) and dried *in vacuo*, yielding L5a (669 mg, 1.81 mmol, 80%) as an orange solid.

A crystal for single crystal structure analysis was obtained from a dichloromethane solution by layering with pentane.

^{1}H NMR (400 MHz, CD_3CN)

δ = 6.92 (s, 1H, CH^{Py}), 3.32 (s, 4H, CH_2), 3.23 (s, 4H, CH_2), 2.61 (s, 6H, CH_3), 2.59 (s, 6H, CH_3) ppm.

^{13}C NMR (101 MHz, CD_3CN) **^{13}C-DEPT-135**

δ = 157.3 (1C, C_q), 156.3 (1C, C_q), 154.6 (1C, C_q), 140.3 (1C, C_q), 135.0 (1C, C_q), 129.6 (2C, CH), 49.0 (2C, CH_2), 48.7 (2C, CH_2), 34.8 (2C, CH_3), 34.5 (2C, CH_3) ppm.

Mass spectrometry (ESI^+ in DCM)

m/*z* = 370.1303 $(L5a+H]^+$, calc. 370.1308).

UV-vis (DCM) ($4.86 \cdot 10^{-5}$ mol L^{-1})

λ (ε) = 345 (8620), 295 (11000), 229 (16100) nm (M^{-1} cm^{-1}).

CV (DCM, [*n*-Bu_4N][PF_6], 20 mV s^{-1}, referenced to Fc/Fc^+)

$E_{1/2}^1$ = 46.6 mV, $E_{1/2}^2$ = 279 mV.

Elemental analysis (C, H, N in %) ($C_{15}H_{21}N_7Cl_2$)

calc.: C 48.66, H 5.72, N 26.48.

found: C 48.44, H 5.55, N 26.61.

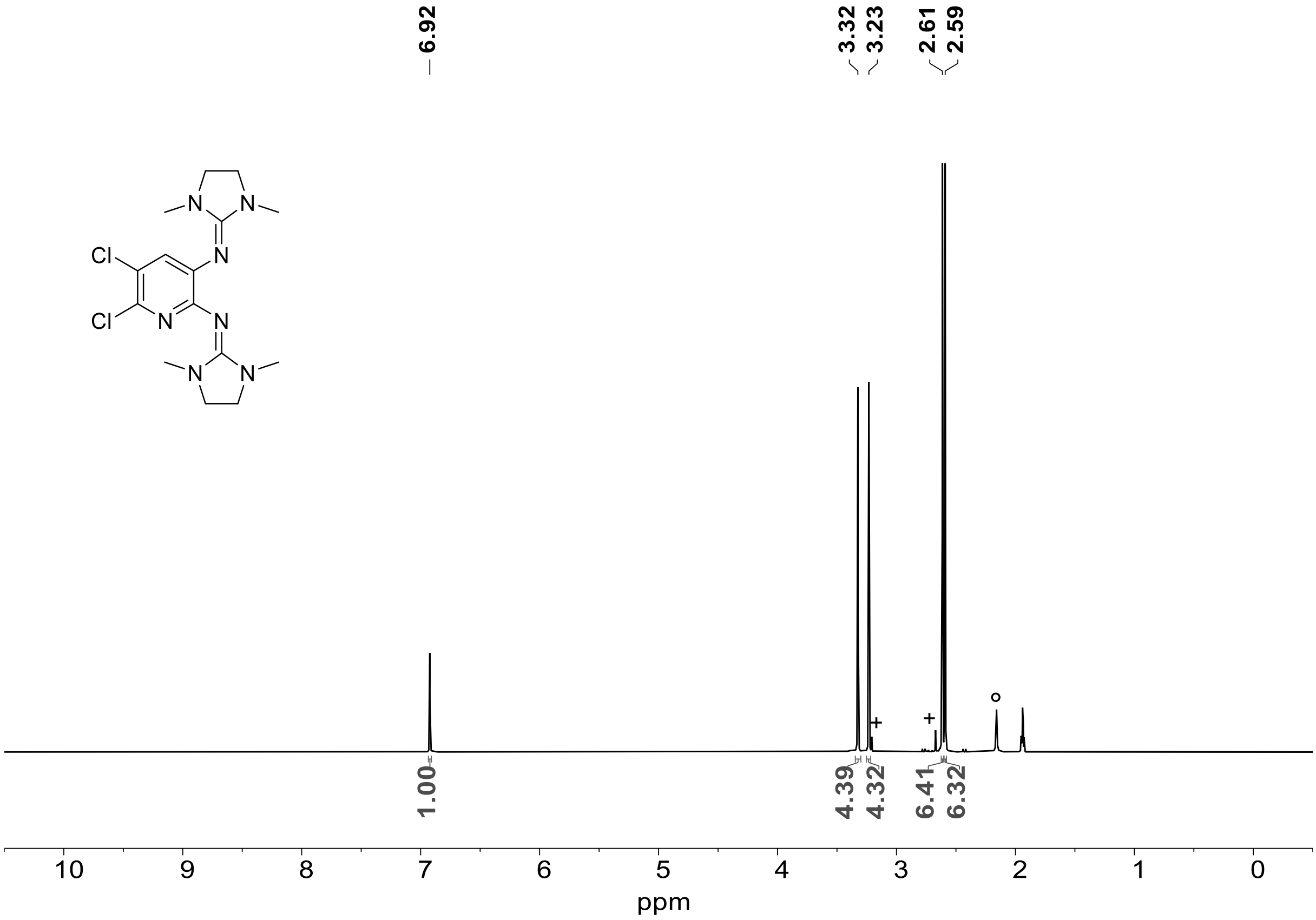

Figure S19: ^{1}H NMR spectrum of L5a measured at 400 MHz in CD_3CN, °water, $^{+}$deactivated 1.

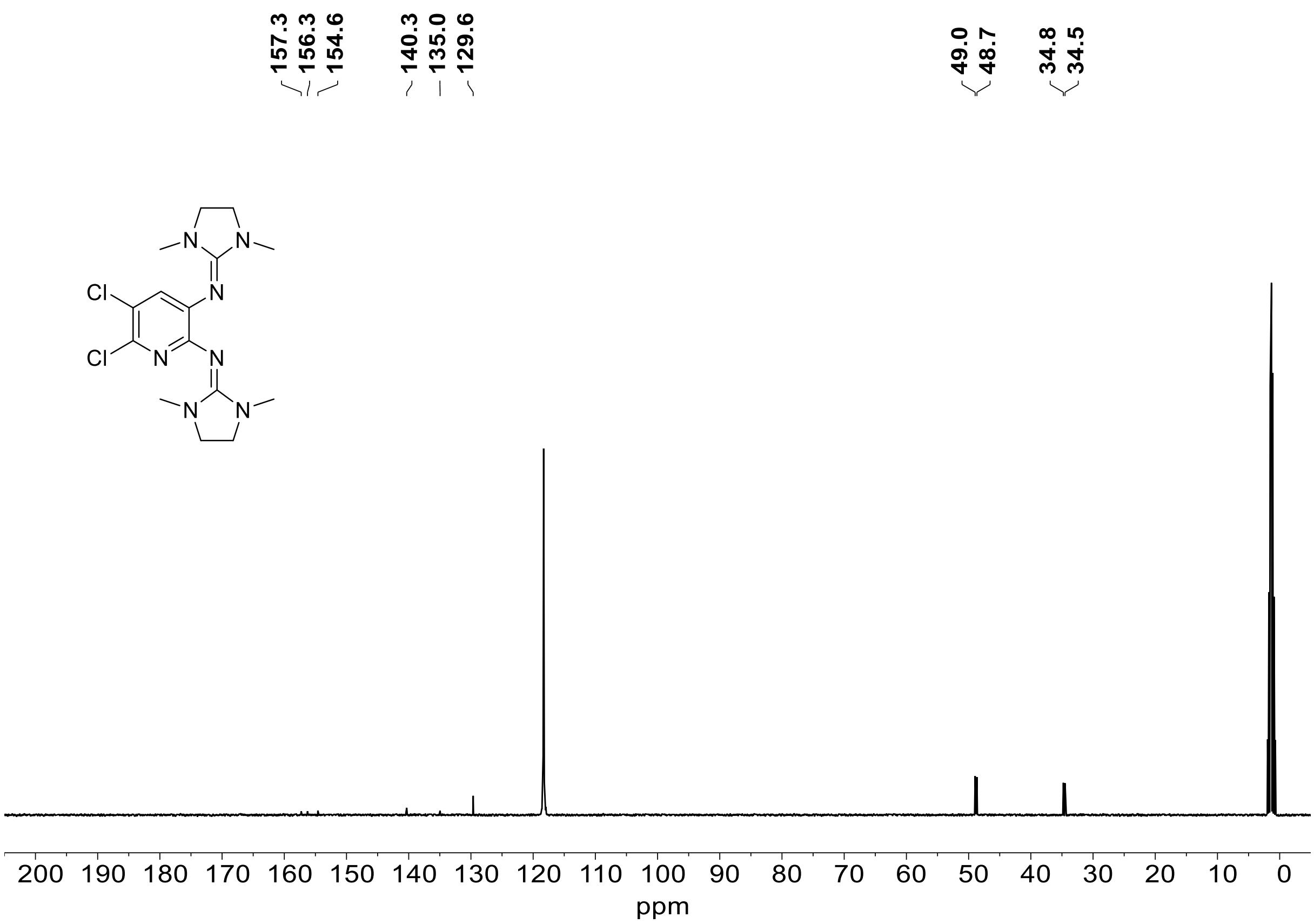

Figure S20: ^{13}C NMR spectrum of L5a, measured at 101 MHz in CD_3CN.

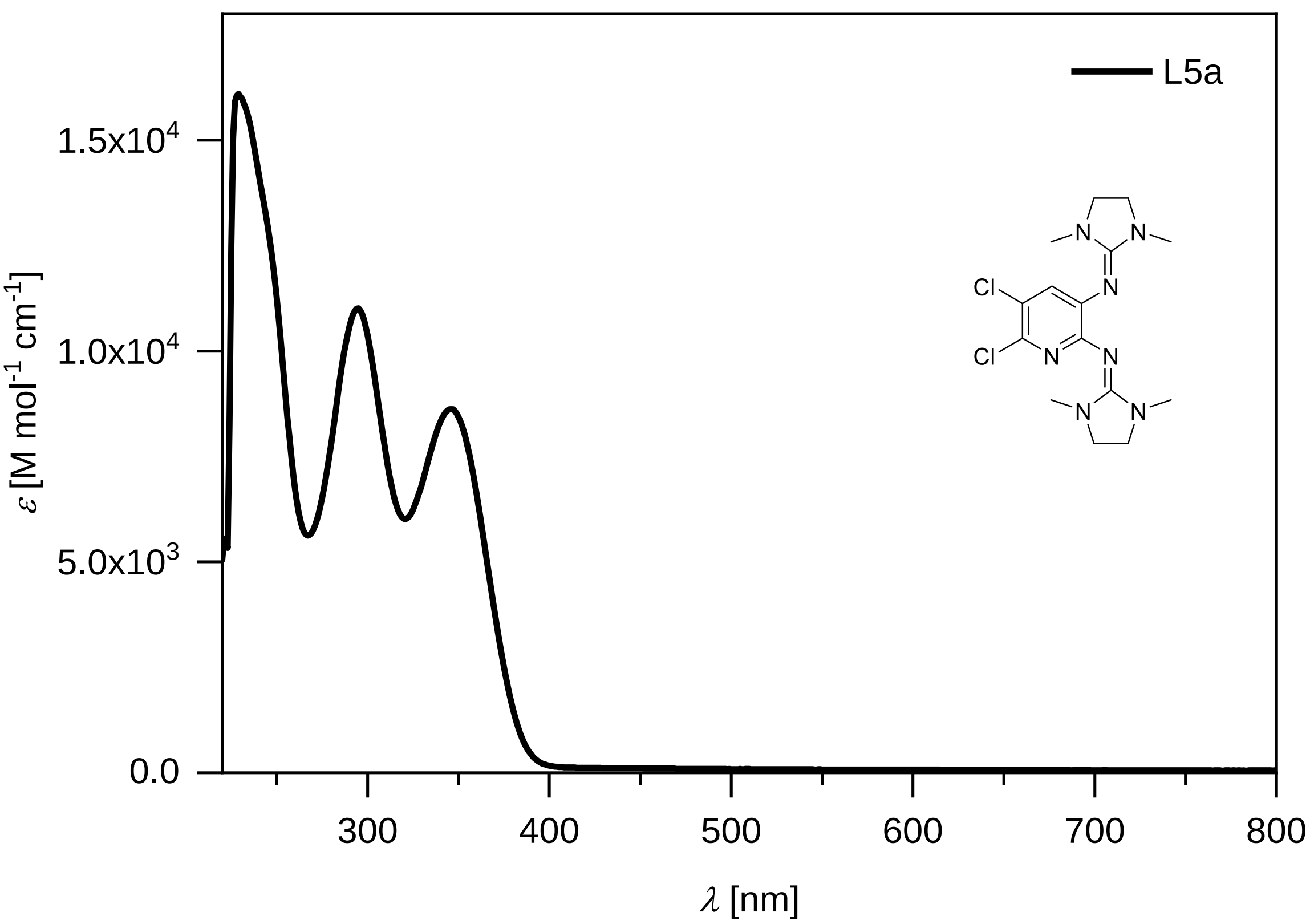

Figure S21: UV-vis absorption spectrum of L5a in dichloromethane at a concentration of $4.86 \cdot 10^{-5}$ mol L^{-1} at rt.

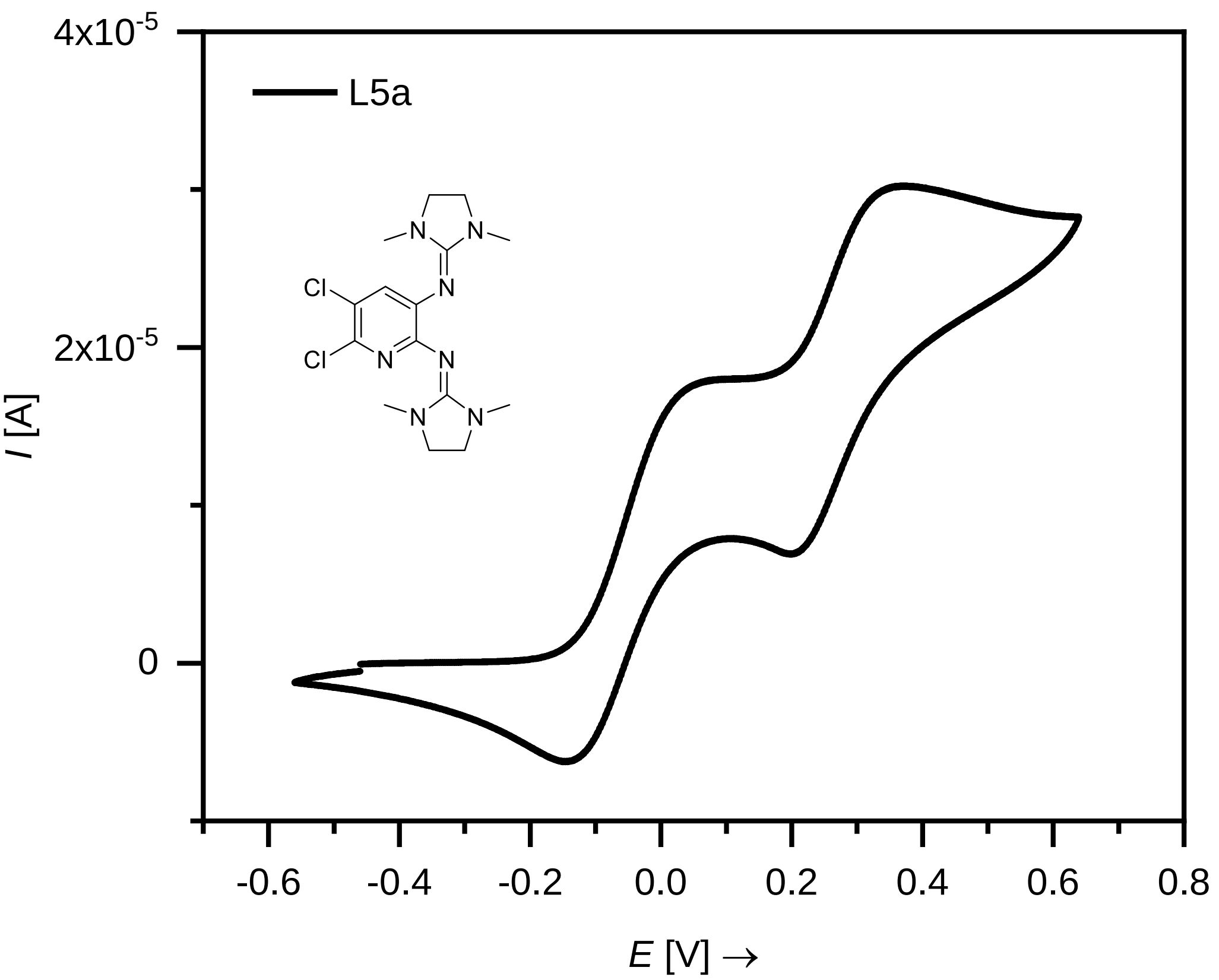

Figure S22: Cyclic voltammogram of L5a in dichloromethane ([n-Bu_4N][PF_6], 20 mV s^{-1}, referenced to Fc/Fc^+).

2.1.8 (L5a)PF_6

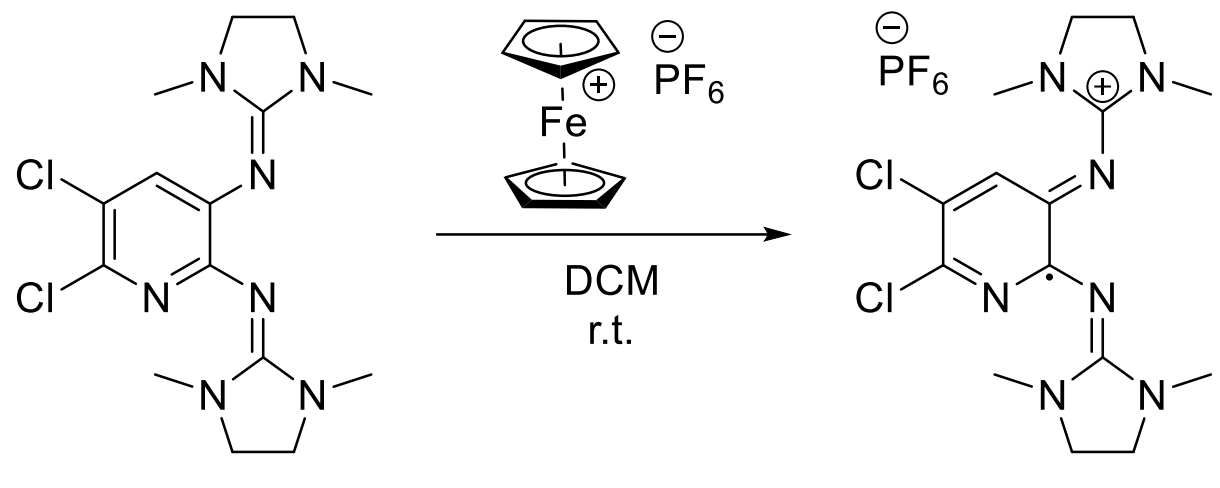

L5a (1.00 eq.) and ferrocenium hexafluorophosphate (0.70 eq.) were dissolved in 2 mL dichloromethane.

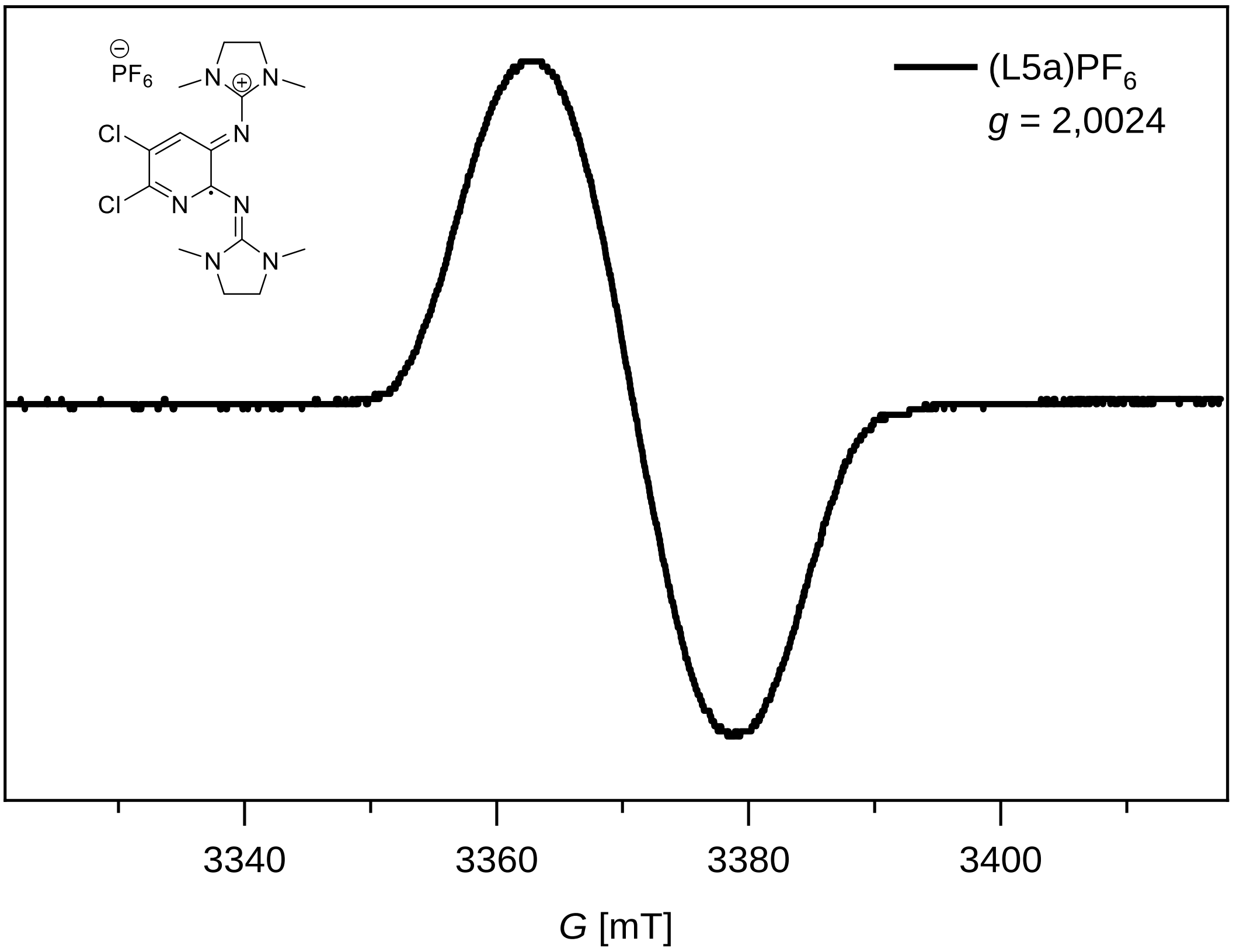

Figure S23: EPR spectrum of (L5a)PF_6 in DCM at room temperature.

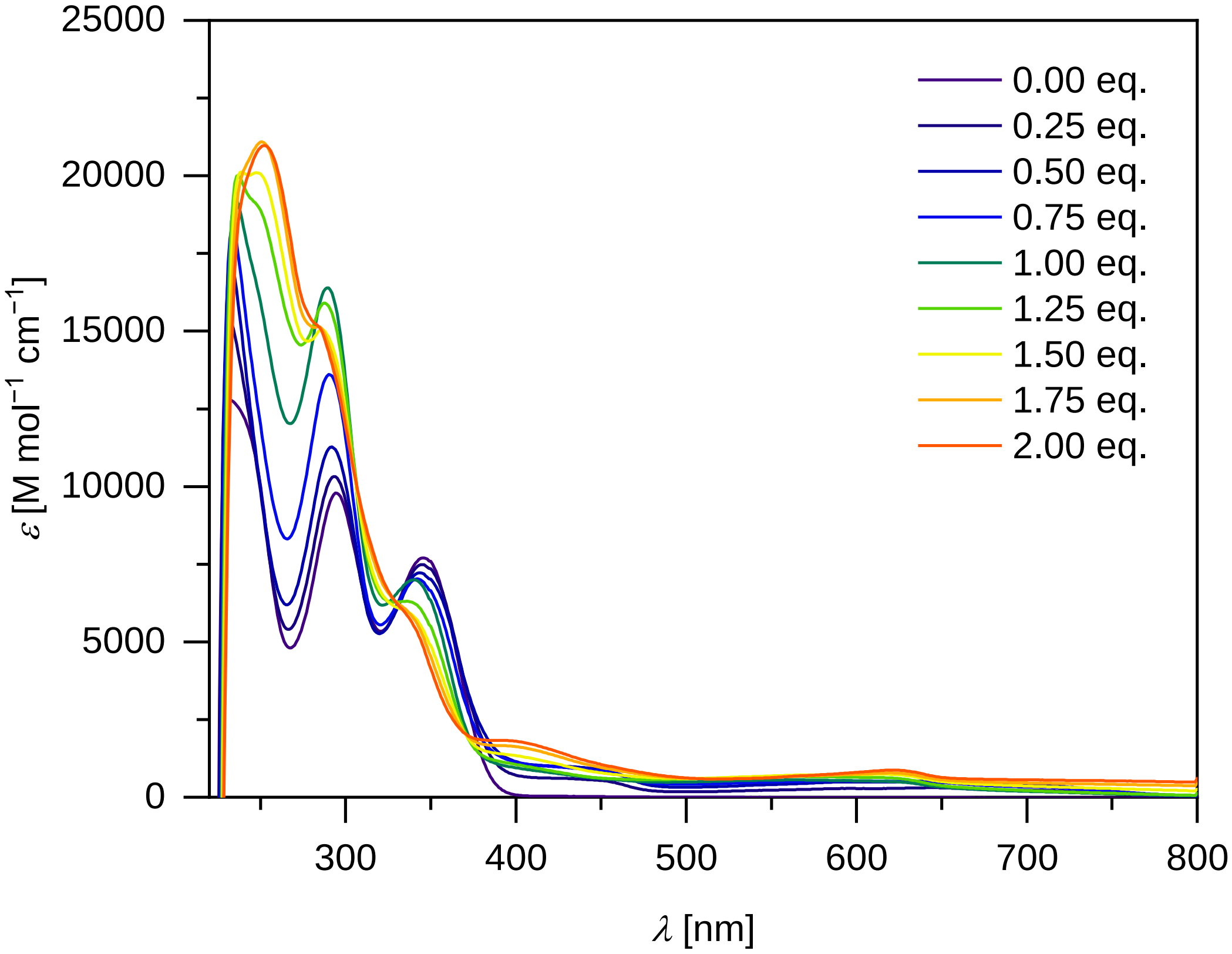

Figure S24: UV-vis absorption spectrum of the redox titration of L5a in dichloromethane at a concentration of $6.48 \cdot 10^{-5}$ mol L^{-1} with ferrocenium hexafluorophosphate at r.t.

2.1.9 L5b

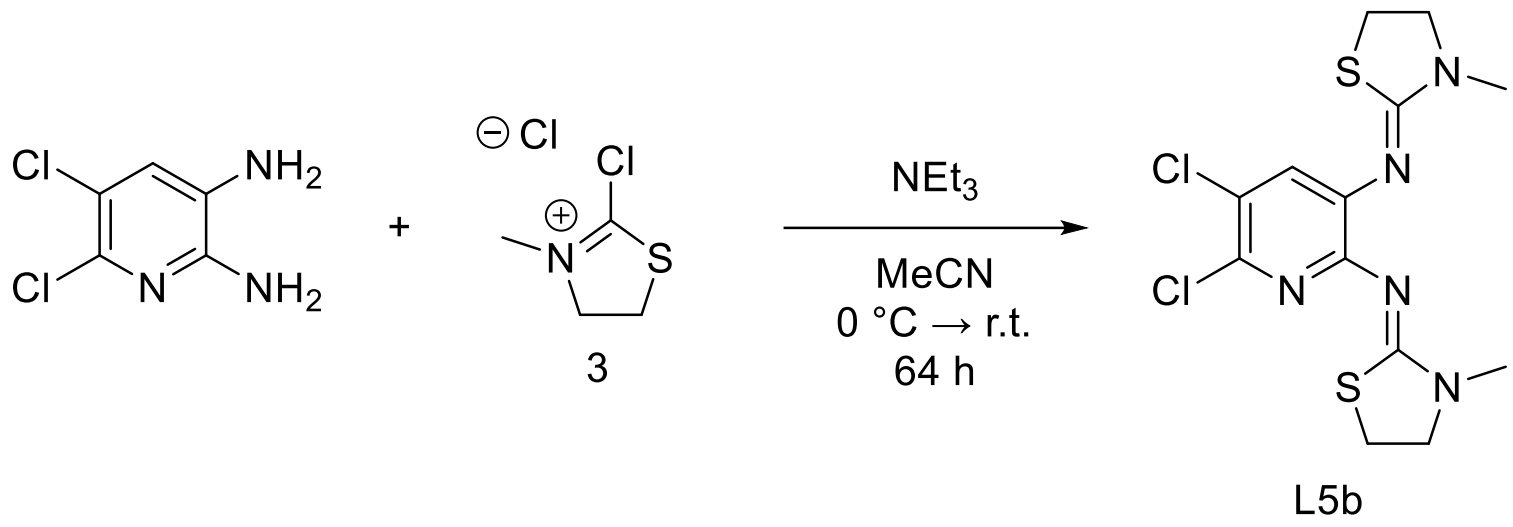

5,6-Dichloropyridine-2,3-diamine (300 mg, 1.69 mmol, 1.00 eq.) was added to a Schlenk flask and dissolved in acetonitrile. Then, 2-chloro-3-methyl-4,5-dihydrothiazolium chloride (3) (667 mg, 3.88 mmol, 2.30 eq.) was added and the reaction mixture was stirred at 0 °C for 15 minutes before slowly adding triethylamine (940 μL, 682 mg, 6.74 mmol, 4.00 eq.). The brown reaction mixture was then stirred for additional 64 h while slowly warming up to room temperature. The precipitate was filtered off, and the solution was dried under reduced pressure. The remaining residue was suspended in $NaOH_{aq}$ (10.0 mL, 25%) while stirring. The aqueous phase was extracted with dichloromethane (4 · 10 mL) and dried over sodium sulphate. The solvent was removed and the raw product was washed with diethyl ether (10 mL) and dried *in vacuo*, yielding L5b (176 mg, 468 μmol, 28%) as a yellow solid.

A crystal suitable for single crystal structure analysis was obtained from a dichloromethane solution by layering with pentane.

^{1}H NMR (399.89 MHz, CD_3CN)

δ = 7.17 (s, 1H), 3.61 – 3.56 (m, 4H), 3.16 (t, *J* = 6.9 Hz, 3H), 3.11 (t, *J* = 7.4 Hz, 3H), 3.04 (s, 3H), 2.97 (s, 3H) ppm.

^{13}C{^{1}H} NMR (100.56 MHz, CD_3CN)

δ = 132.0, 54.1, 52.3, 34.3, 34.0, 27.9, 27.7 ppm.
6 signals are coincident or not observed.

Mass spectrometry (ESI^+ in DCM)

m/z = 376.0212 ($[L5b+H]^+$, calc. 376.0219).

UV-vis (DCM) ($4.32\cdot10^{-5}$ mol L^{-1})

λ (ε) = 346 (14200), 296 (shoulder, 16000), 285 (20100), 278 (19241), 255 (18400), 232 (51200) nm (M^{-1} cm^{-1}).

CV (DCM, [*n*-Bu_4N][PF_6], 20 mV s^{-1}, referenced to Fc/Fc^+)

E_{Ox}^1 = 534 mV, E_{Ox}^2 = 657 mV, E_{Ox}^3 = 883 mV, E_{Ox}^4 = 1.09 V,

E_{Red}^1 = 556 mV, E_{Red}^2 = 792 mV, E_{Red}^3 = 980 mV.

Elemental analysis (C, H, N in %) ($C_{13}H_{15}Cl_2N_5S_2$ · 0.2 eq. DCM)

calc.: C 40.31, H 3.95, N 17.81.

found: C 40.31, H 3.69, N 17.78.

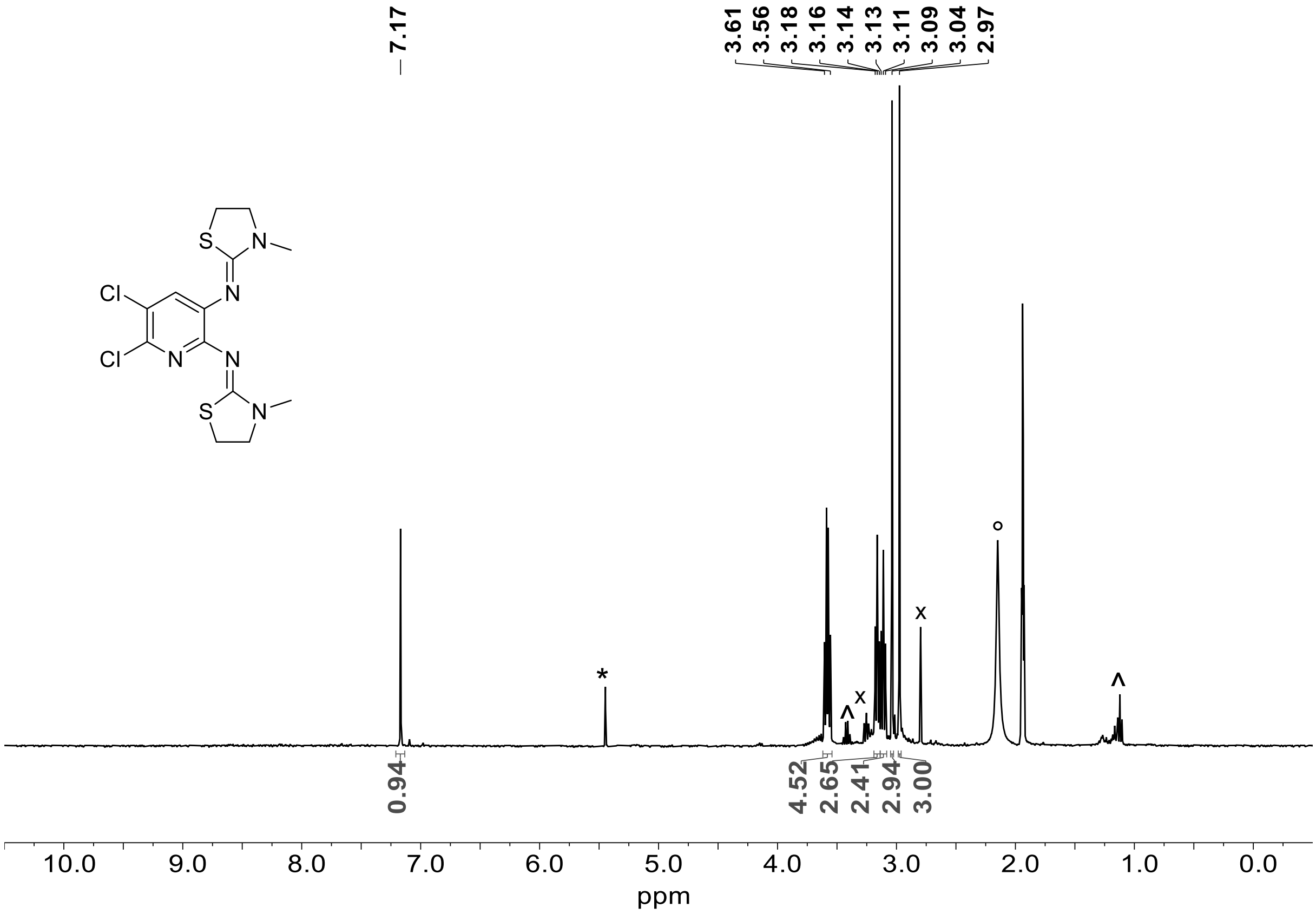

Figure S25: ^{1}H NMR spectrum of L5b, measured at 400 MHz in CD_3CN, *DCM, ^diethyl ether, ^{x}deactivated 1, °water.

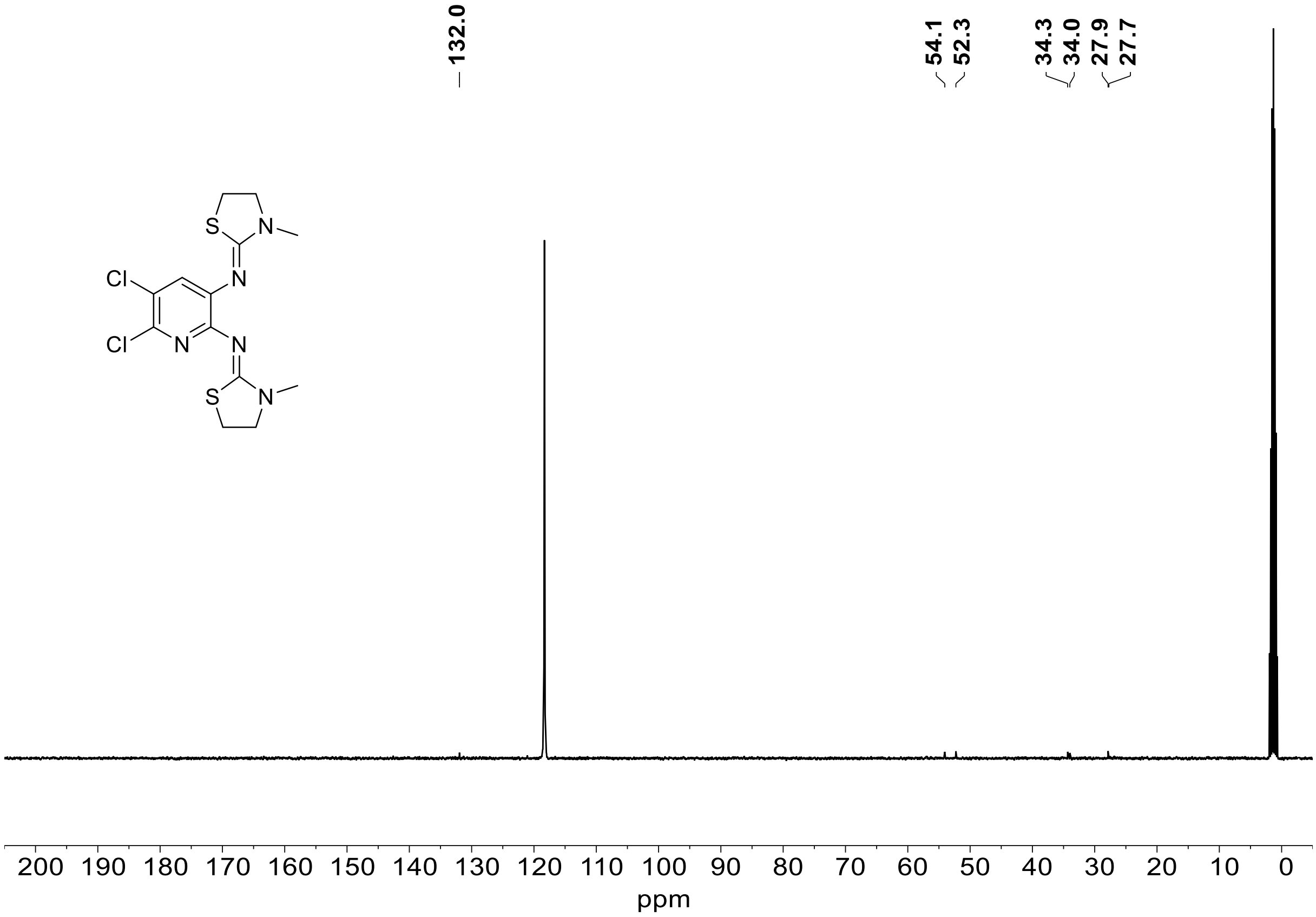

Figure S26: ^{13}C NMR spectrum of L5b, measured at 101 MHz in CD_3CN.

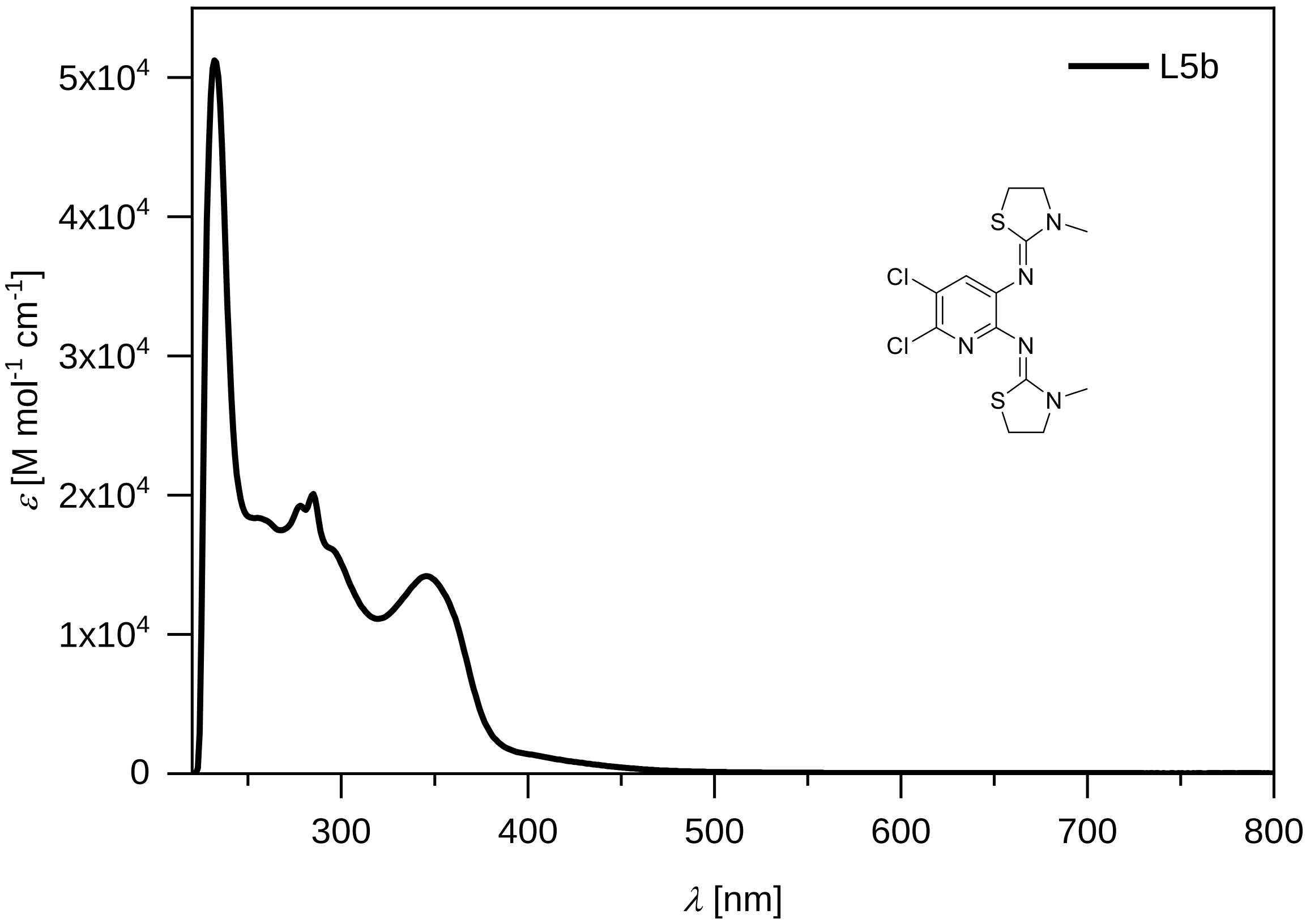

Figure S27: UV-vis absorption spectrum of L5b in dichloromethane at a concentration of $4.32 \cdot 10^{-5}$ mol L^{-1} at rt.

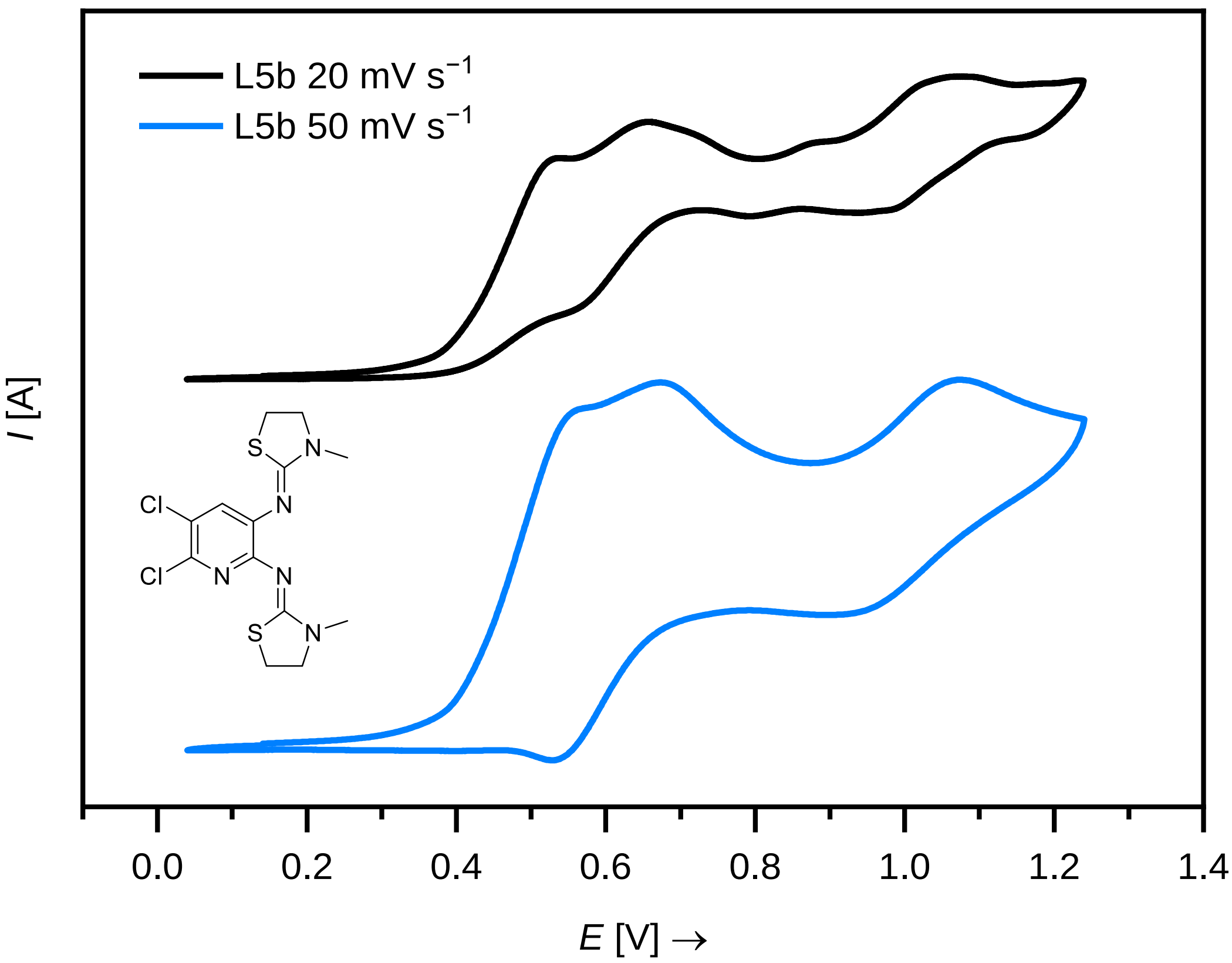

Figure S28: Cyclic voltammogram of L5b in dichloromethane ([n-Bu_4N][PF_6], 20 mV s^{-1} and 50 mV s^{-1}, referenced to Fc/Fc^+).

2.1.10 L6

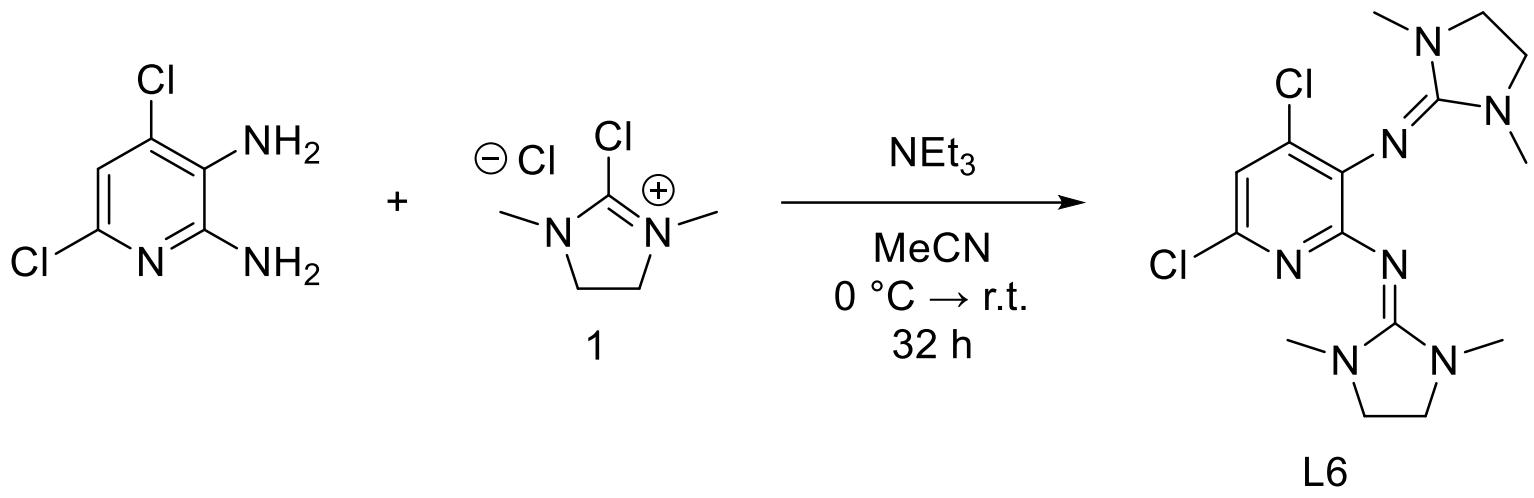

4,6-Dichloropyridine-2,3-diamine (196 mg, 1.10 mmol, 1.00 eq.) was dissolved in acetonitrile (10 ml) and 1 in acetonitrile (1 M, 2.60 mL, 440 mg, 2.60 mmol, 2.36 eq.) was added. The mixture was stirred at 0 °C for 15 min before adding triethylamine (630 µL, 460 mg, 4.54 mmol, 4.13 eq.) dropwise over five minutes. Slowly warming up, the mixture was stirred for 16 h. After filtration, the solvent was removed from the filtrate *in vacuo,* and the residue was stirred vigorously with degassed $NaOH_{aq}$ (25%, 10 mL). The aqueous phase was extracted with diethyl ether (4 x 10 mL) and DCM (3 x 10 mL), and both organic phases were processed separately. Both organic phases were dried over $NaSO_4$, filtered and evaporated *in vacuo* to obtain a light brown solid. The DCM phase was further purified by first dissolving the oil in toluene (5 mL) and then in diethyl ether (5 mL) in an ultrasound bath, removing the solvent under reduced pressure yielding the product as a light brown solid. For the diethyl ether phase, due to an incomplete reaction the process was repeated with the resulting solid in acetonitrile (10 ml), 1 in acetonitrile (1 M, 2.60 mL, 440 mg, 2.60 mmol, 2.36 eq.) and triethylamine (620 µL, 453 mg, 4.47 mmol, 4.10 eq.) yielding a light brown solid after extraction. The product was further purified by dissolving the oil first in toluene (5 mL) and then in diethyl ether (5 mL) in an ultrasound bath, removing the solvent under reduced pressure, yielding the product L6 (374 mg, 1.01 mmol, 38%) as a light brown solid.

A crystal suitable for single crystal structure analysis was obtained from a dichloromethane solution by layering with pentane.

^{1}H NMR (600 MHz, CD_3CN)

δ = 6.71 (s, 1H, CH^{Py}), 3.35 (s, 4H, CH_2), 3.25 – 3.22 (m, 4H, CH_2), 2.63 (s, 6H, CH_3), 2.59 (s, 6H, CH_3) ppm.

^{13}C NMR (101 MHz, CD_3CN) **^{13}C-DEPT-135**

δ = 157.4 (1C, C_q), 156.5 (1C, C_q), 156.1 (1C, C_q), 138.6 (1C, C_q), 136.1 (1C, C_q), 134.4 (1C, C_q), 114.1 (1C, CH), 48.9 (2C, CH_2), 48.6 (2C, CH_2), 34.4 (2C, CH_3), 34.2 (2C, CH_3) ppm.

Mass spectrometry (ESI^+ in DCM)

m/*z* = 370.1300 ($[L6+H]^+$, calc. 370.1308).

UV-vis (DCM) ($6.30\cdot10^{-5}$ mol L^{-1})

λ (ε) = 334 (5890), 289 (6530), 228 (10900) nm (M^{-1} cm^{-1}).

CV (DCM, [*n*-Bu_4N][PF_6], 20 mV s^{-1}, referenced to Fc/Fc^+)

$E_{1/2}^1$ = −20.7 mV, $E_{1/2}^2$ = 324 mV.

Elemental analysis (C, H, N in %) ($C_{15}H_{21}N_7Cl_2$)

calc.: C 48.66, H 5.72, N 26.48.

found: C 48.33, H 5.41, N 26.46.

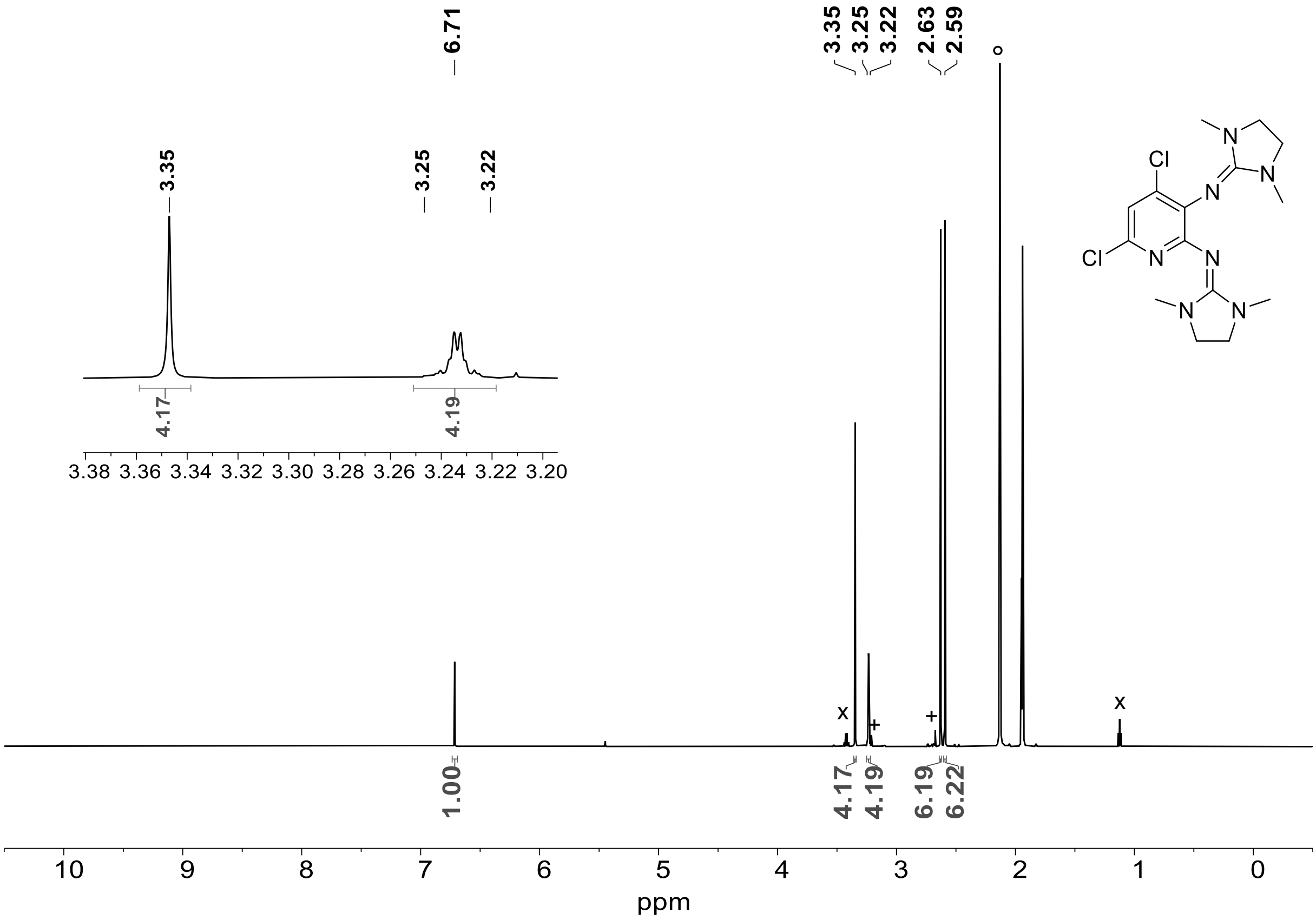

Figure S29: ^{1}H NMR spectrum of L6 measured at 600 MHz in CD_3CN, [+]deactivaded 1, [x]diethyl ether, [°]water.

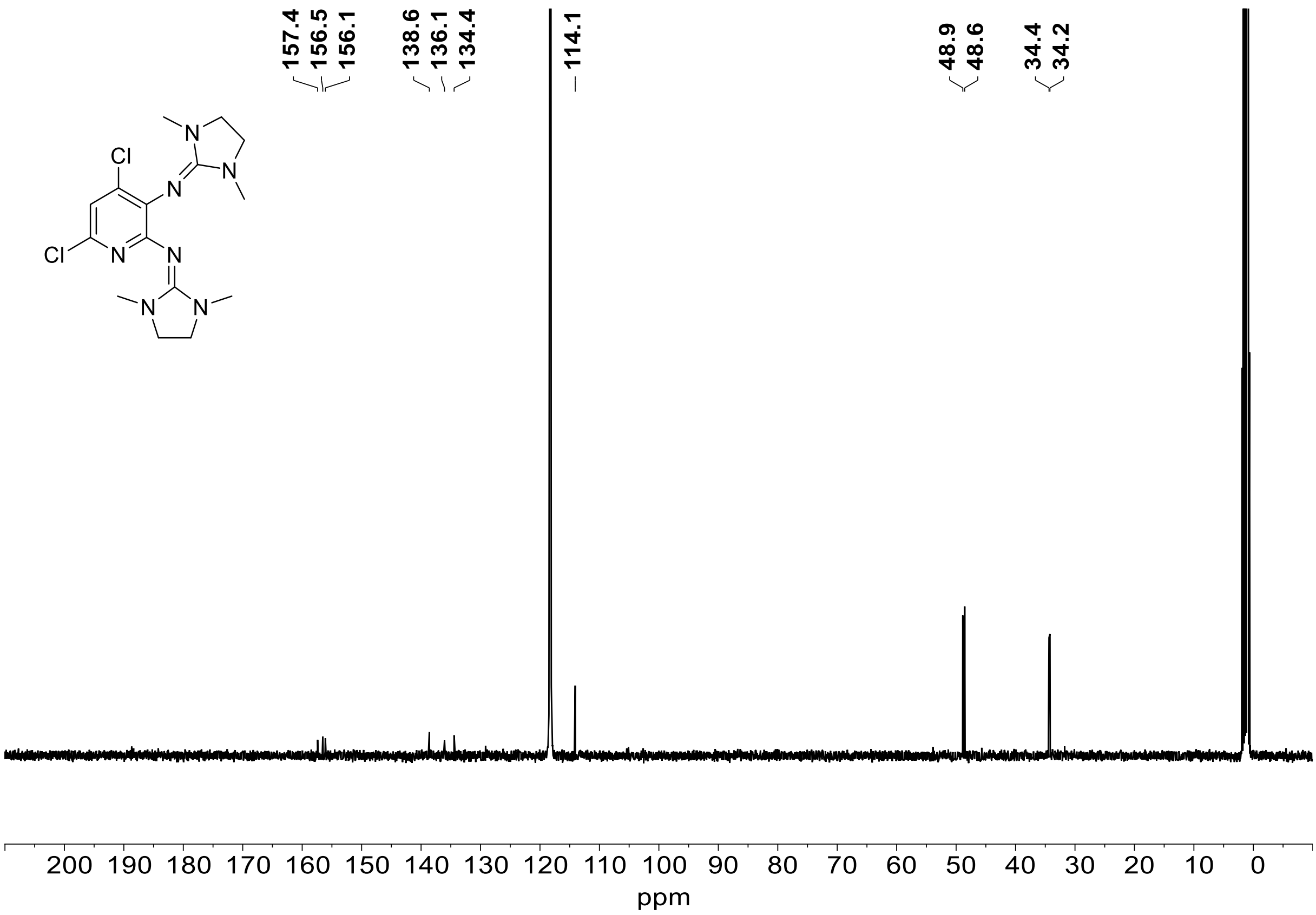

Figure S30: ^{13}C NMR spectrum of L6, measured at 101 MHz in CD_3CN.

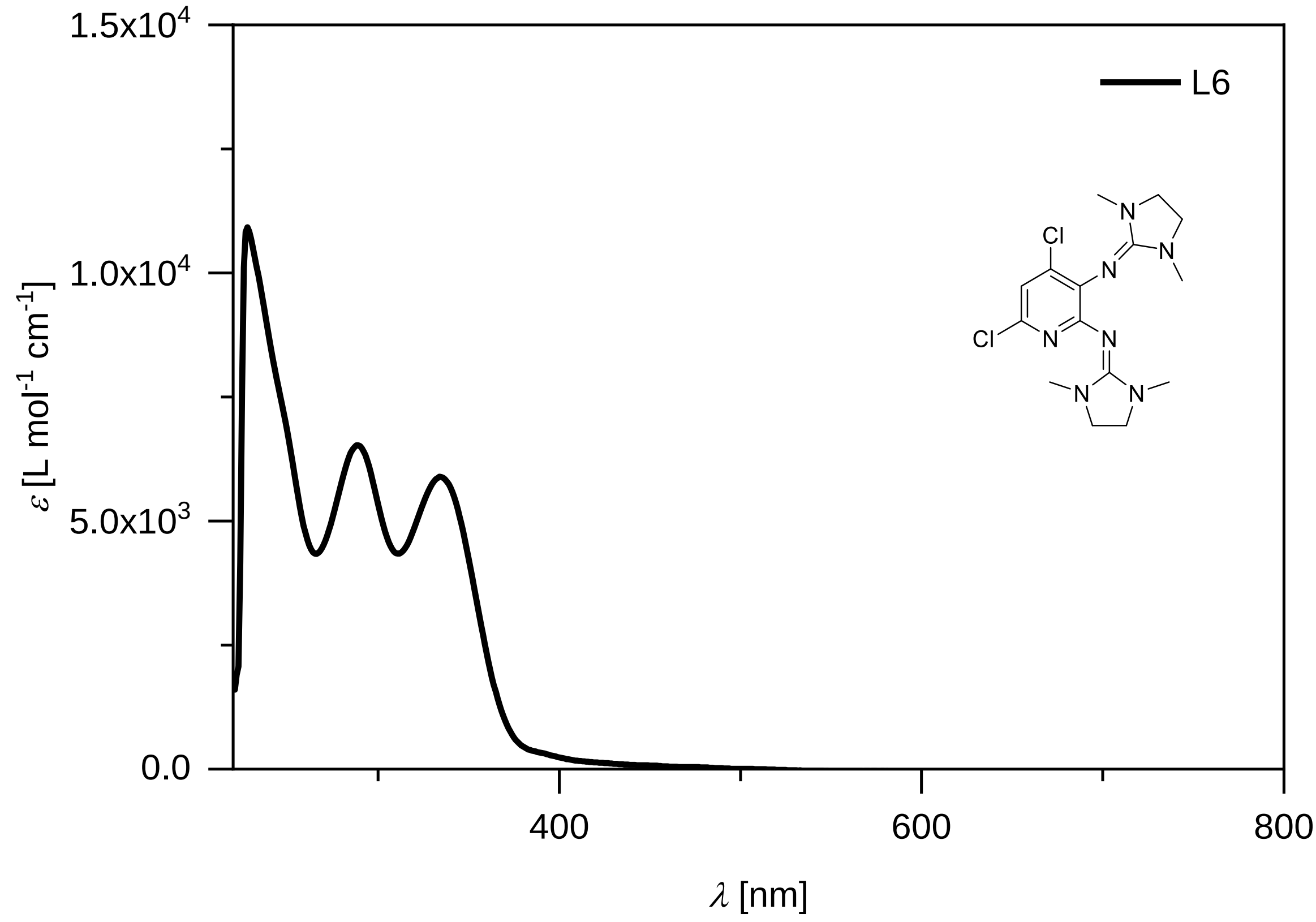

Figure S31: UV-vis absorption spectrum of L6 in dichloromethane at a concentration of $6.30 \cdot 10^{-5}$ mol L^{-1} at rt.

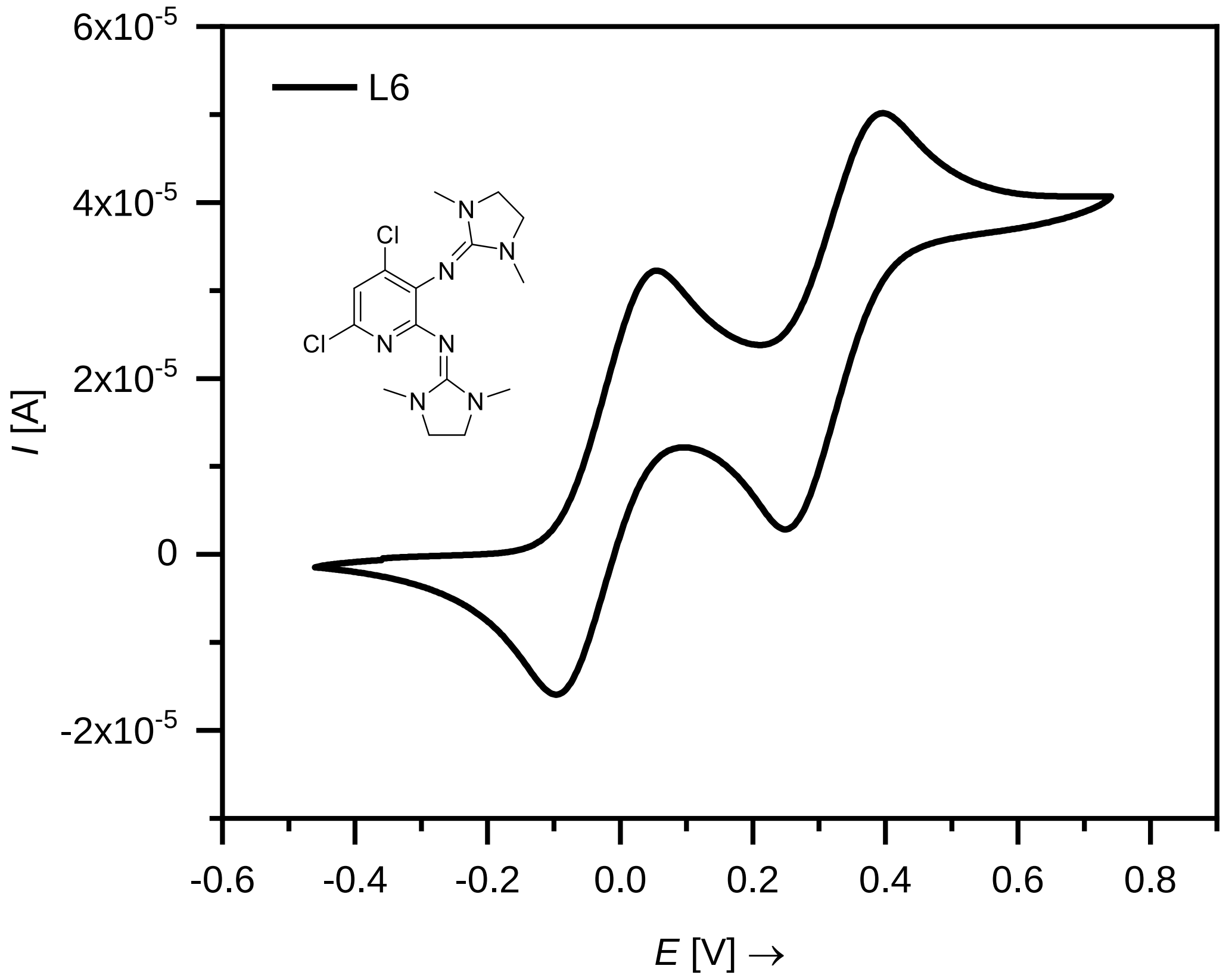

Figure S32: Cyclic voltammogram of L6 in dichloromethane ([n-Bu_4N][PF_6], 20 mV s^{-1}, referenced to Fc/Fc^+).

2.1.11 (L6)PF_6

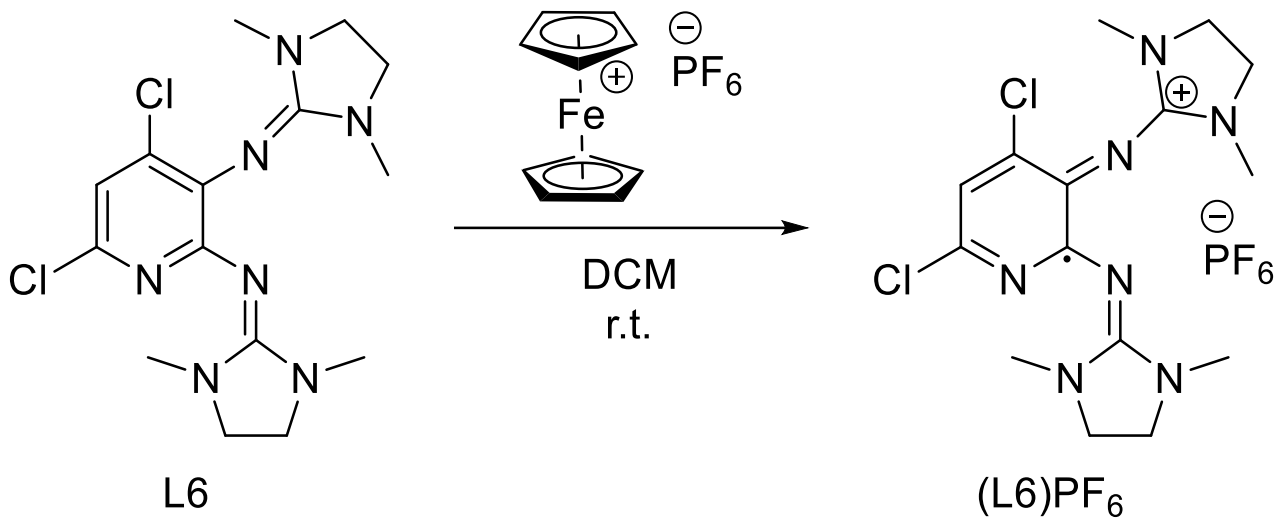

L6 (1.00 eq.) and ferrocenium hexafluorophosphate (0.70 eq.) were dissolved in 2 mL dichloromethane.

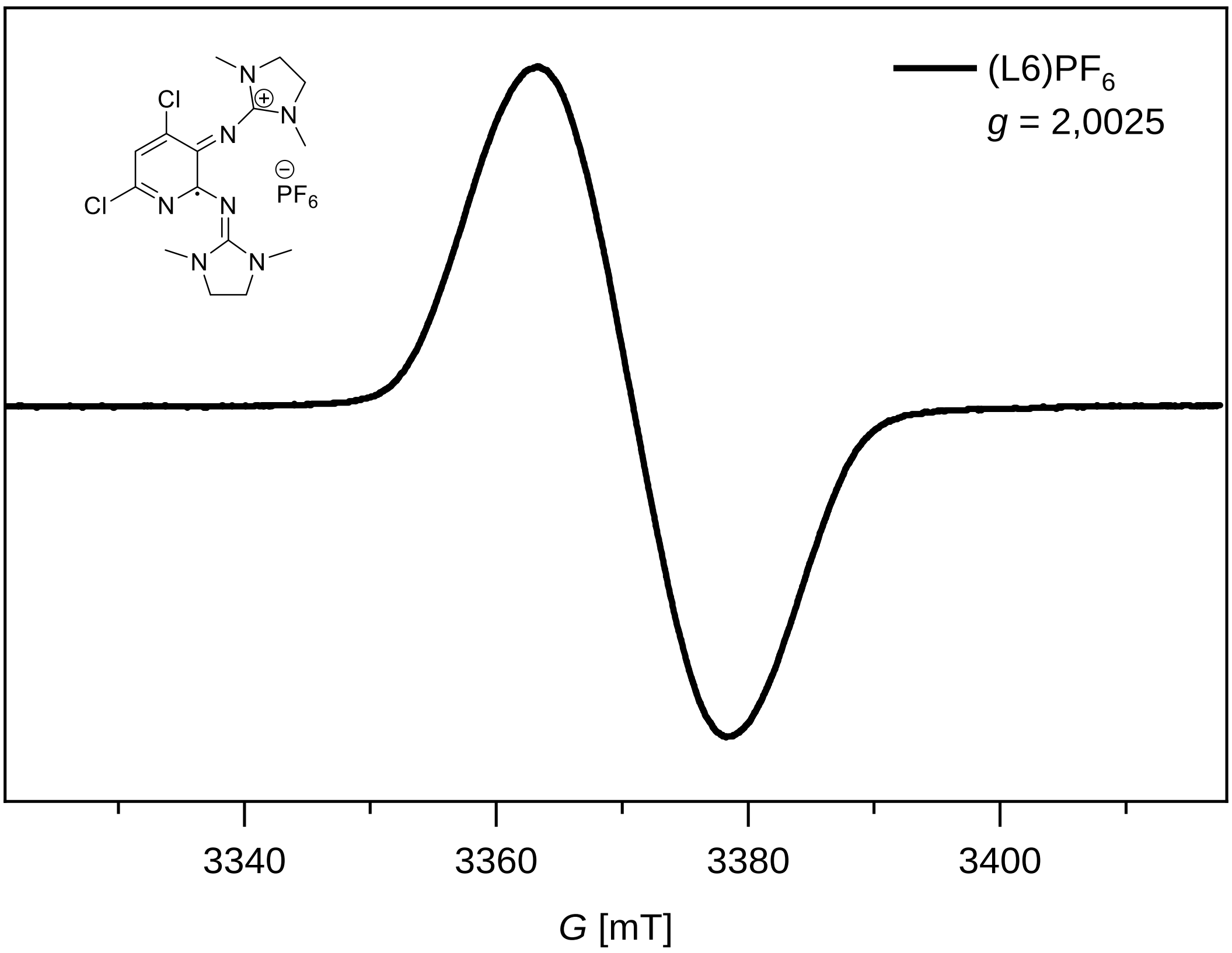

Figure S33: EPR spectrum of (L6)PF_6 in DCM at room temperature.

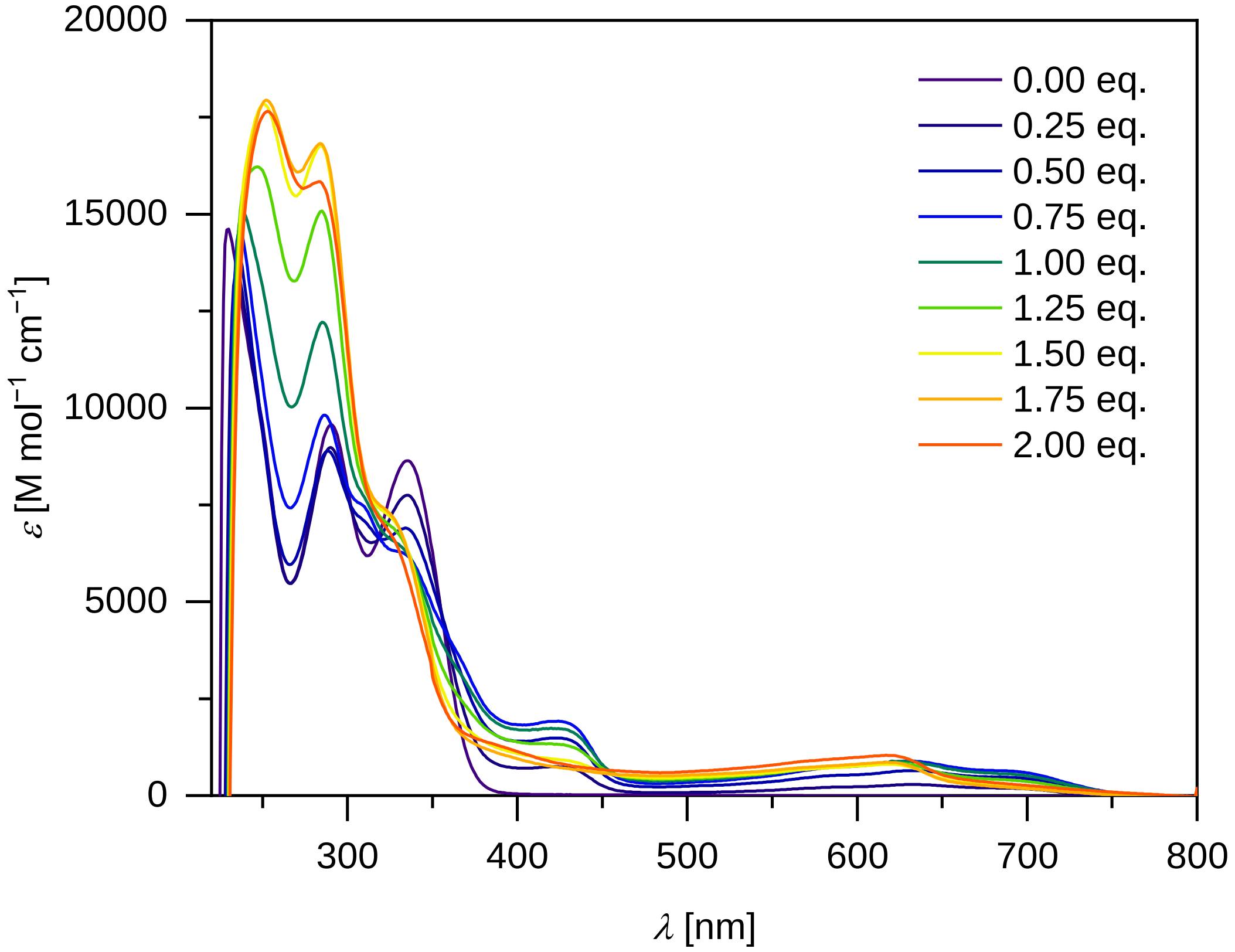

Figure S34: UV-vis absorption spectrum of the redox titration of L6 in dichloromethane at a concentration of $4.75\cdot10^{-5}$ mol L^{-1} with ferroceniumhexafluorophosphate at rt.

2.2 Complexes

2.2.1 [NiCl$_2$(L2a)]

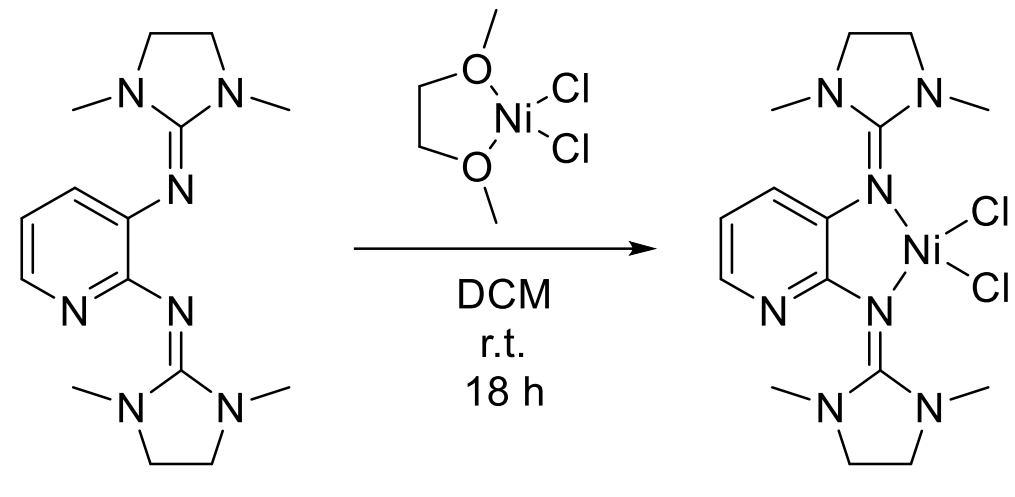

L2a (15.0 mg, 49.8 μmol, 1.00 eq.) was dissolved in dichloromethane (3 mL). Nickel(II) chloride dimethoxyethane adduct (12.0 mg, 54.7 μmol, 1.10 eq.) was added and the reaction mixture stirred for 18 h at r.t. while the reaction mixture turned red. Then, the suspension was filtered through a syringe filter, and the solution was overlayered with diethyl ether. After eight days, the dark crystals were filtered off and dried under reduced pressure, yielding the dark red complex [NiCl$_2$(L2a)] (21.0 mg, 48.7 μmol, 98%).

Mass spectrometry (ESI$^+$ in DCM)

m/z = 302.2090 ([L2a+H]$^+$, calc. 302.2088).

UV-vis (DCM) ($4.80 \cdot 10^{-5}$ mol L^{-1})

λ (ε) = 438 (591), 327 (shoulder, 9380), 292 (14800), 229 (21600) nm (M^{-1} cm^{-1}).

CV (DCM, [*n*-Bu$_4$N][PF$_6$], 20 mV s^{-1}, referenced to Fc/Fc$^+$)

E_{Ox}^1 = 255 mV, E_{Ox}^2 = 865 mV,

E_{Red}^1 = 123 mV.

Elemental analysis (C, H, N in %) ($C_{15}H_{23}Cl_2NiN_7$)

calc.: C 41.80, H 5.38, N 22.75.

found: C 41.66, H 5.38, N 22.75.

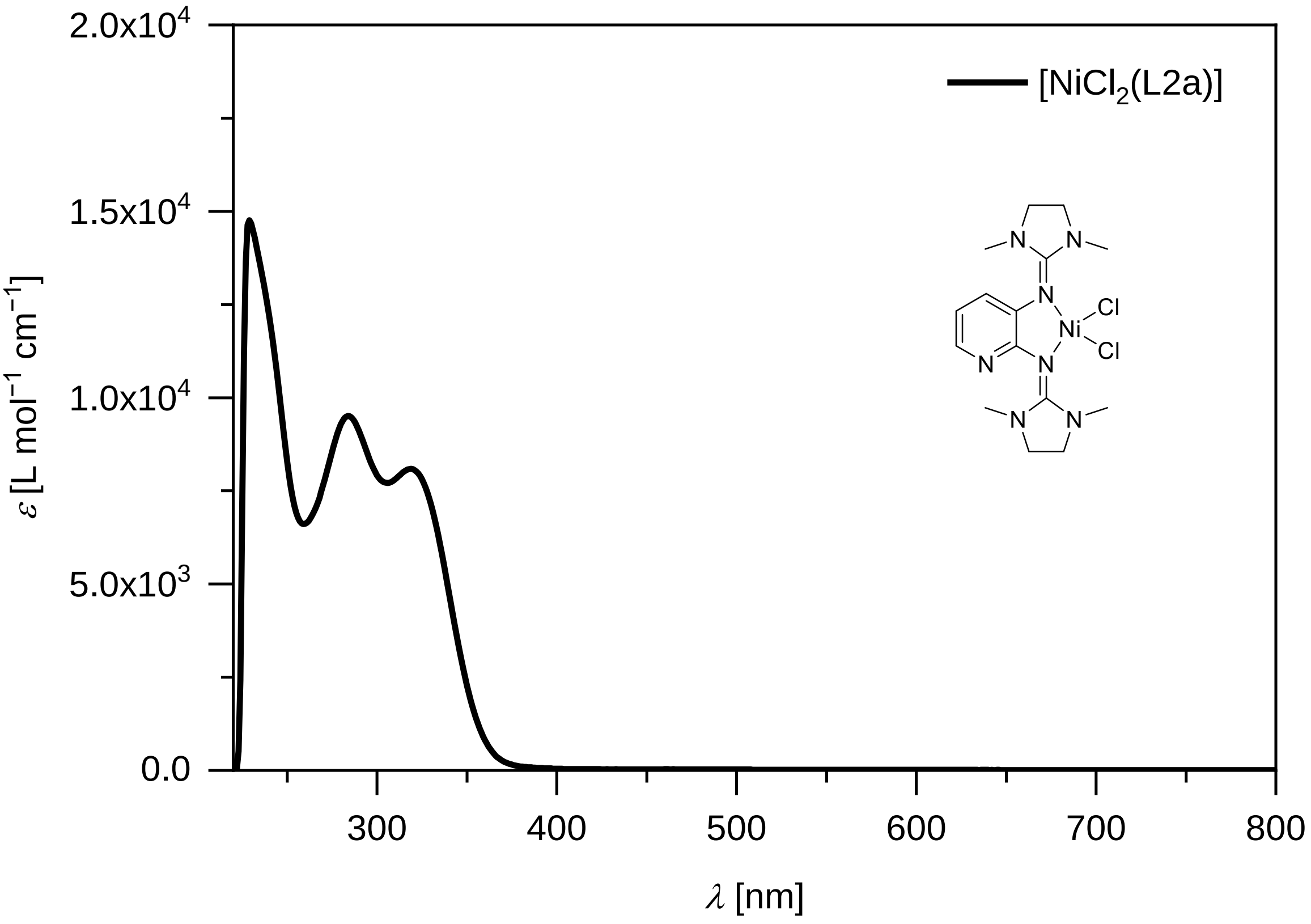

Figure S35: UV-vis absorption spectrum of [$NiCl_2$(L2a)] in dichloromethane at a concentration of $4.80 \cdot 10^{-5}$ mol L^{-1} at rt.

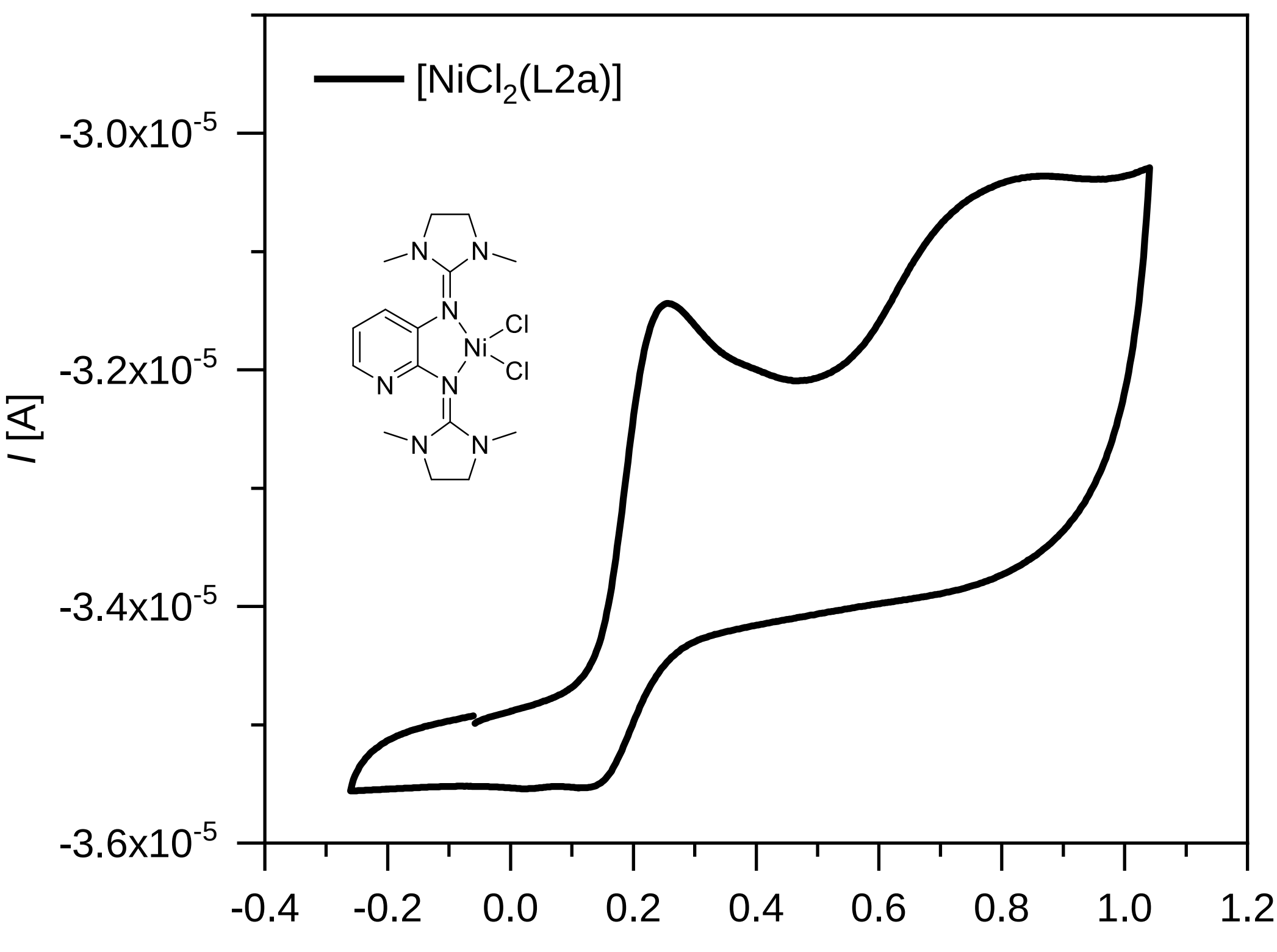

Figure S36: Cyclic voltammogram of [$NiCl_2$(L2a)] in dichloromethane ([*n*-Bu_4N][PF_6], 20 mV s^{-1}, referenced to Fc/Fc^+).

2.2.2 [$CoCl_2$(L2a)]

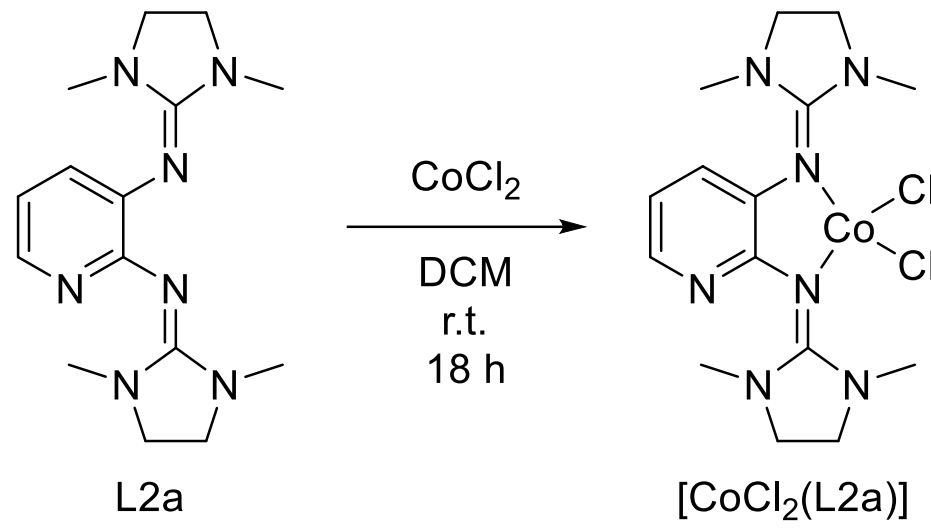

L2a (15.0 mg, 49.8 μmol, 1.00 eq.) was dissolved in dichloromethane (3 mL). Cobalt(II) chloride (8.40 mg, 64.7 μmol, 1.30 eq.) was added and the reaction mixture stirred for 18 h at r.t. while the mixture turned dark blue. The suspension was filtered through a syringe filter and the solution was overlayered with diethyl ether. After eight days, the blue-purple crystals were filtered off and dried under reduced pressure, yielding the cornflower blue complex [$CoCl_2$(L2a)] (19.8 mg, 45.9 μmol, 92%).

Mass spectrometry (ESI^+ in DCM)

m/z = 302.2090 ($[L2a+H]^+$, calc. 302.2088).

UV-vis (DCM) ($4.64 \cdot 10^{-5}$ mol L^{-1})

λ (ε) = 667 (281), 620 (172), 543 (216), 324 (shoulder, 7740), 286 (12000), 229 (17000) nm (M^{-1} cm^{-1}).

CV (DCM, [*n*-Bu_4N][PF_6], 20 mV s^{-1}, referenced to Fc/Fc^+)

E_{Ox}^1 = 303 mV, E_{Ox}^2 = 614 mV,

E_{Red}^1 = 179 mV.

Elemental analysis (C, H, N in %) ($C_{15}H_{23}Cl_2CoN_7$ · 0.3 eq. DCM)

calc.: C 40.24, H 5.21, N 21.47.

found: C 40.38, H 5.54, N 21.59.

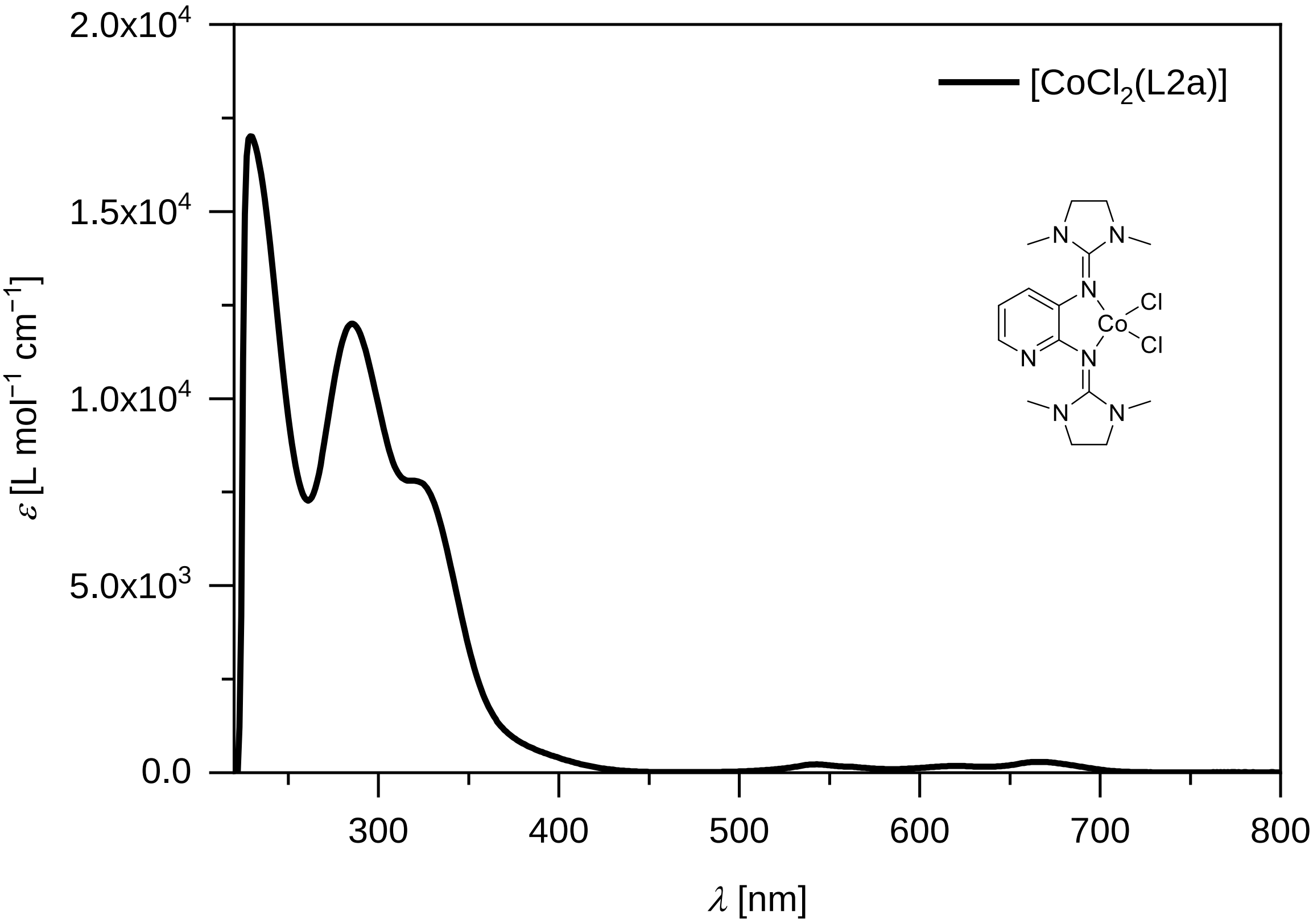

Figure S37: UV-vis absorption spectrum of [$CoCl_2$(L2a)] in dichloromethane at a concentration of $4.64 \cdot 10^{-5}$ mol L^{-1} at rt.

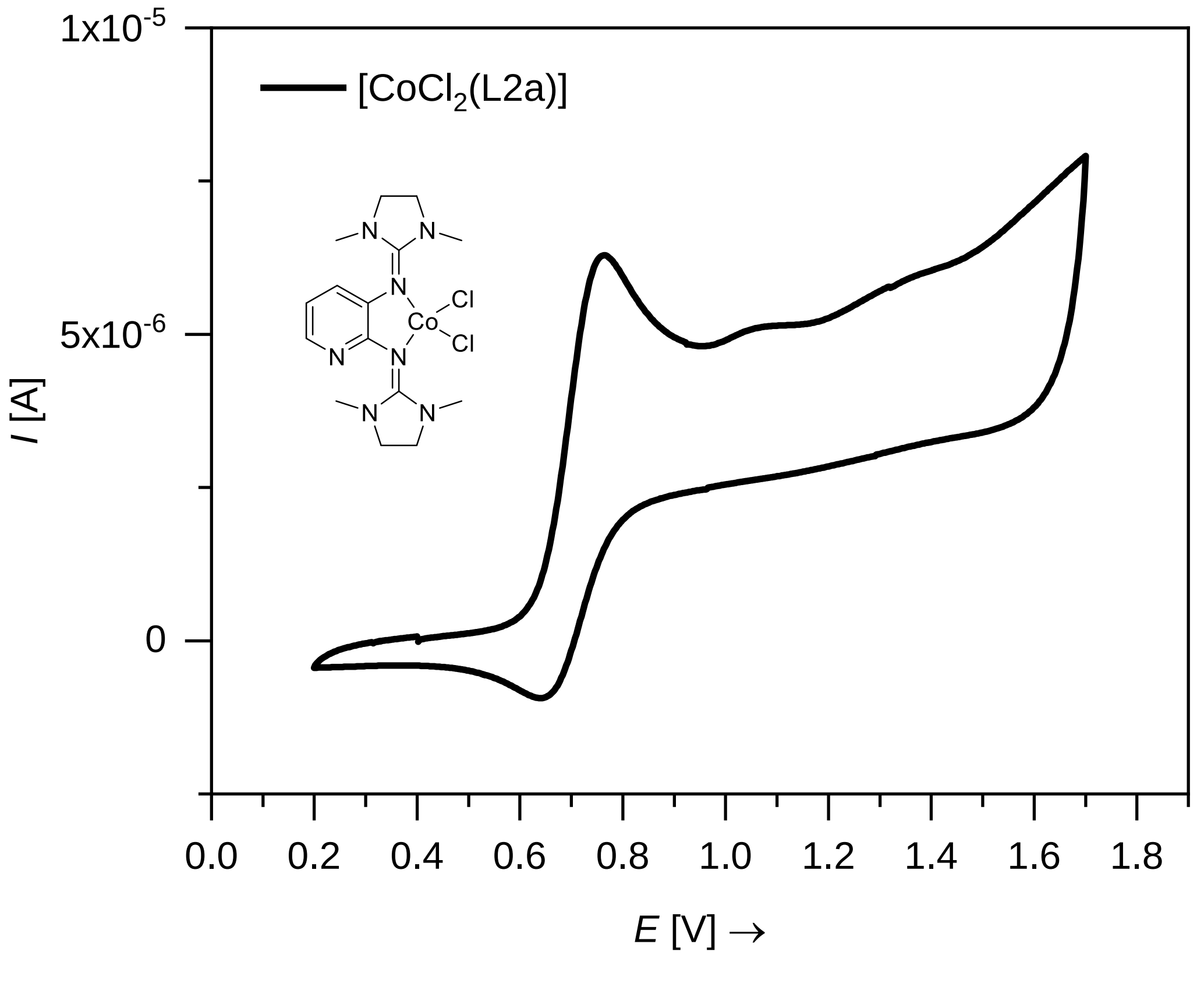

Figure S38: Cyclic voltammogram of [$CoCl_2$(L2a)] in dichloromethane ([n-Bu_4N][PF_6], 20 mV s^{-1}, referenced to Fc/Fc^+).

2.2.3 [$CoBr_2$(L2a)]

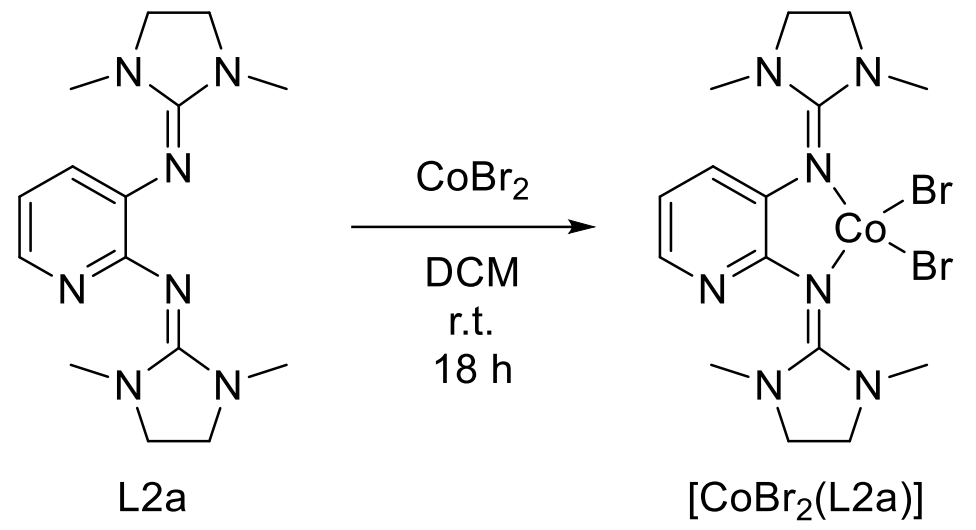

L2a (15.0 mg, 49.8 µmol, 1.00 eq.) was dissolved in dichloromethane (3 mL). Cobalt(II) bromide (14.2 mg, 64.7 µmol, 1.30 eq.) was added and the reaction mixture stirred for 18 h at r.t. while the mixture turned from green to turquoise. The suspension was filtered using a syringe filter and the solution was overlayered with diethyl ether. After eight days, the turquoise crystals were filtered off and dried under reduced pressure, yielding the turquoise complex [$CoBr_2$(L2a)] (24.9 mg, 47.8 µmol, 96%).

Mass spectrometry (ESI^+ in DCM)

m/z = 820.1623 ([$CoBr_2(L2a)_2$-H]$^+$, calc. 820.1630).

UV-vis (DCM) ($6.41 \cdot 10^{-5}$ mol L^{-1})

λ (ε) = 674 (309), 562 (167), 325 (shoulder, 8950), 287 (15900), 229 (22100) nm (M^{-1} cm^{-1}).

CV (DCM, [n-Bu_4N][PF_6], 20 mV s^{-1}, referenced to Fc/Fc^+)

$E_{Ox}{}^1$ = 752 mV,

$E_{Red}{}^1$ = 647 mV, $E_{Red}{}^2$ = 302 mV.

Elemental analysis (C, H, N in %) ($C_{15}H_{23}Br_2CoN_7 \cdot 0.5$ eq. DCM)

calc.: C 33.09, H 4.30, N 17.43.

found: C 32.25, H 4.33, N 17.43.

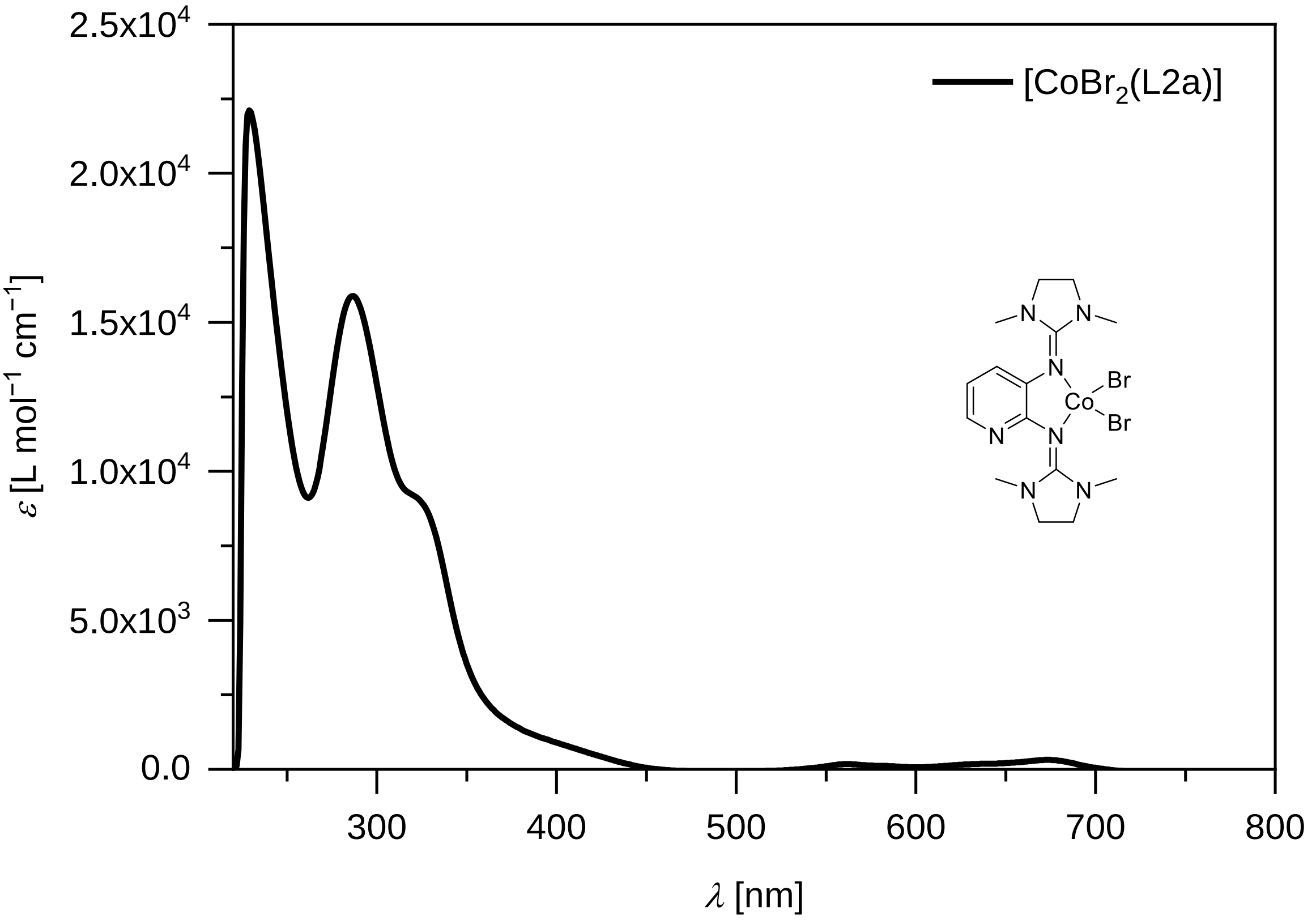

Figure S39: UV-vis absorption spectrum of [$CoBr_2$(L2a)] in dichloromethane at a concentration of 6.41· 10^{-5} mol L^{-1} at rt.

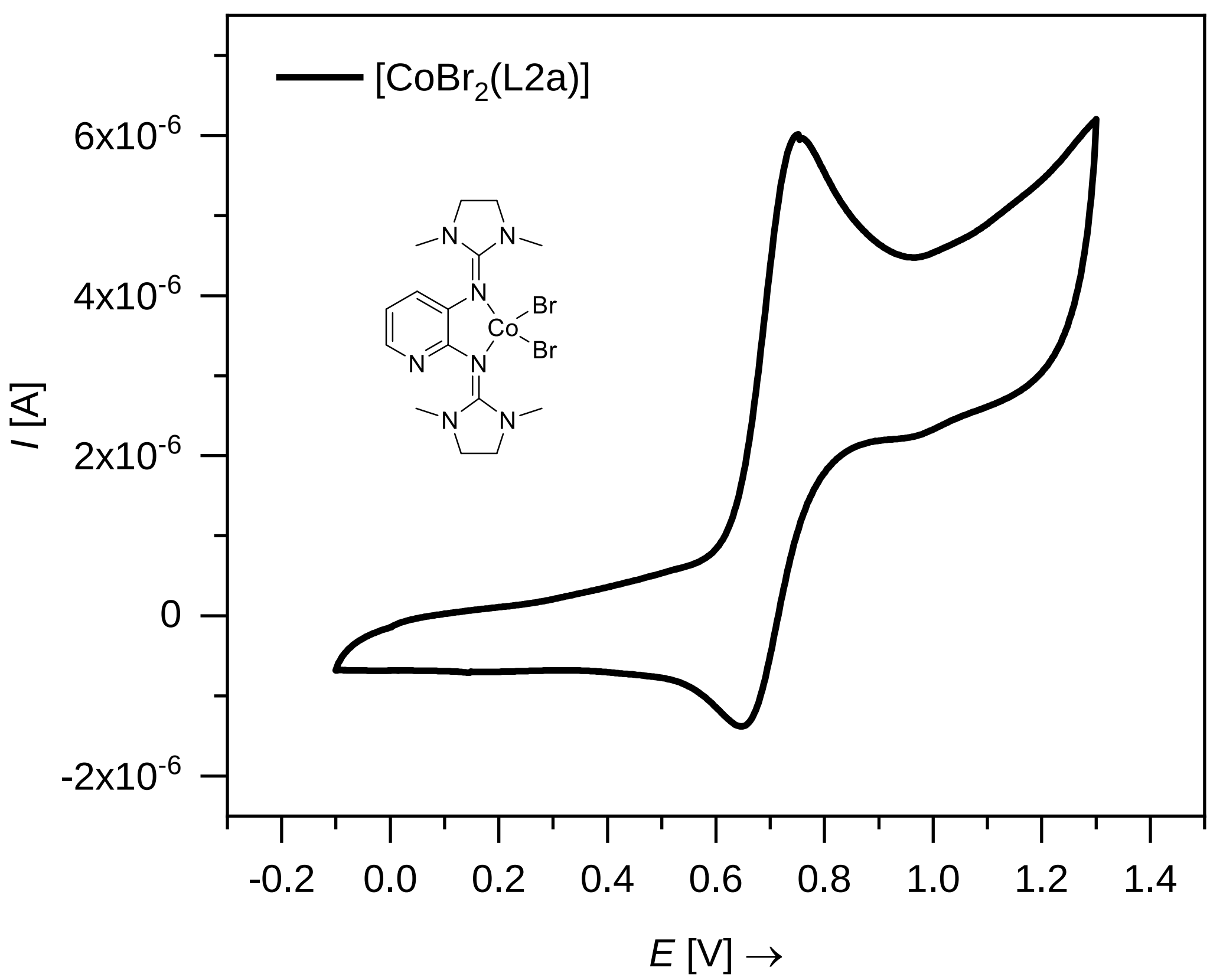

Figure S40: Cyclic voltammogram of [$CoBr_2$(L2a)] in dichloromethane and acetonitrile ([*n*-Bu_4N][PF_6], 20 mV s^{-1}, referenced to Fc/Fc^+).

2.2.4 [Co(hfac)$_2$(L2a)]

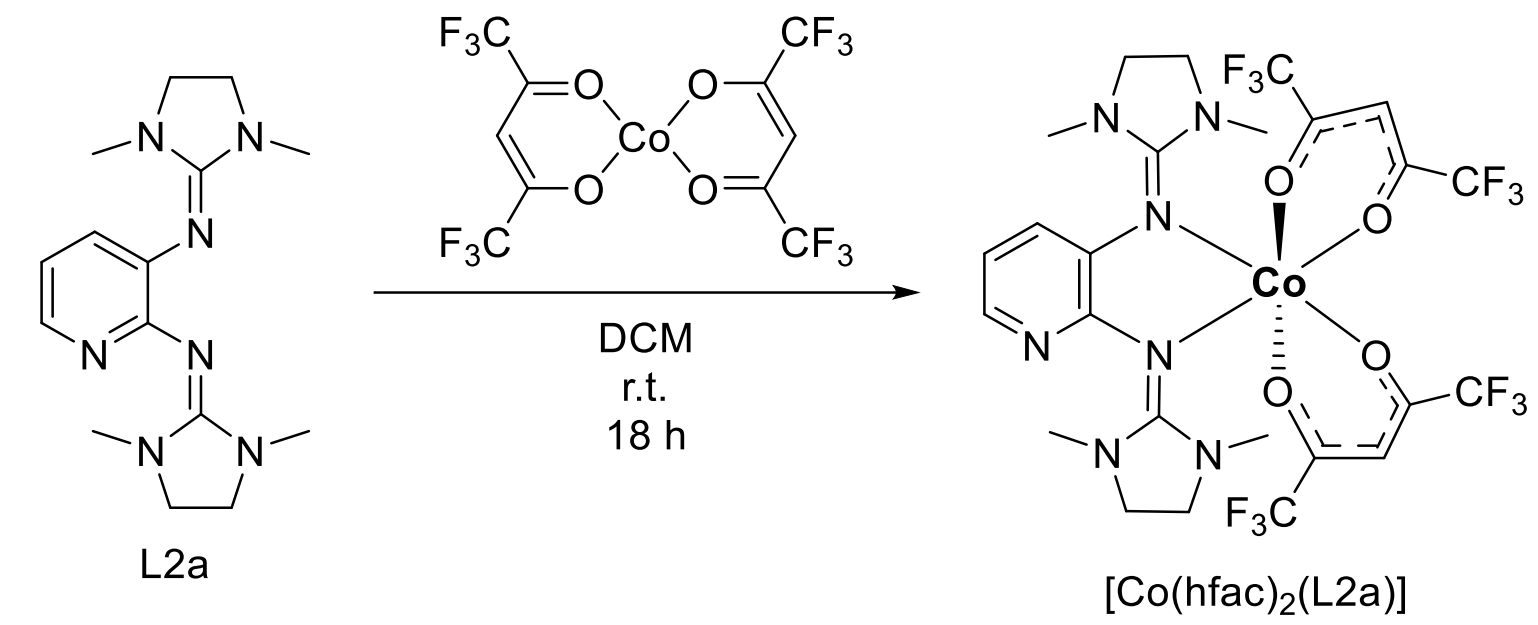

L2a (15.0 mg, 49.8 μmol, 1.00 eq) and cobalt(II)hexafluoroacetylacetonate (23.5 mg, 49.8 μmol, 1.00 eq.) were dissolved in dichloromethane (3 mL) The reaction mixture was stirred for 18 h at r.t. while the mixture was turning red. The solvent was evaporated under reduced pressure and the product was solved in diethyl ether. Slow evaporation of the solvent yielded [Co(hfac)$_2$(L2a)] (35.9 mg, 46.4 μmol, 93%) as red crystals.

Mass spectrometry (ESI$^+$ in DCM)

m/z = 775.1180 ([Co(hfac)$_2$(L2a)]+H]$^+$, calc. 775.1181)

UV-vis (DCM) ($2.67 \cdot 10^{-5}$ mol L^{-1})

λ (ε) = 460 (shoulder, 426), 298 (18600), 228 (17700) nm (M^{-1} cm^{-1}).

CV (DCM, [*n*-Bu$_4$N][PF$_6$], 20 mV s^{-1}, referenced to Fc/Fc$^+$)

$E_{1/2}{}^1$ = 70.9 mV, $E_{1/2}{}^2$ = 714 mV.

Elemental analysis (C, H, N in %) ($C_{25}H_{25}CoF_{12}N_7O_4 \cdot 0.3$ eq. Et_2O)

calc.: C 39.50, H 3.54, N 12.31.

found: C 38.66, H 4.13, N 11.36.

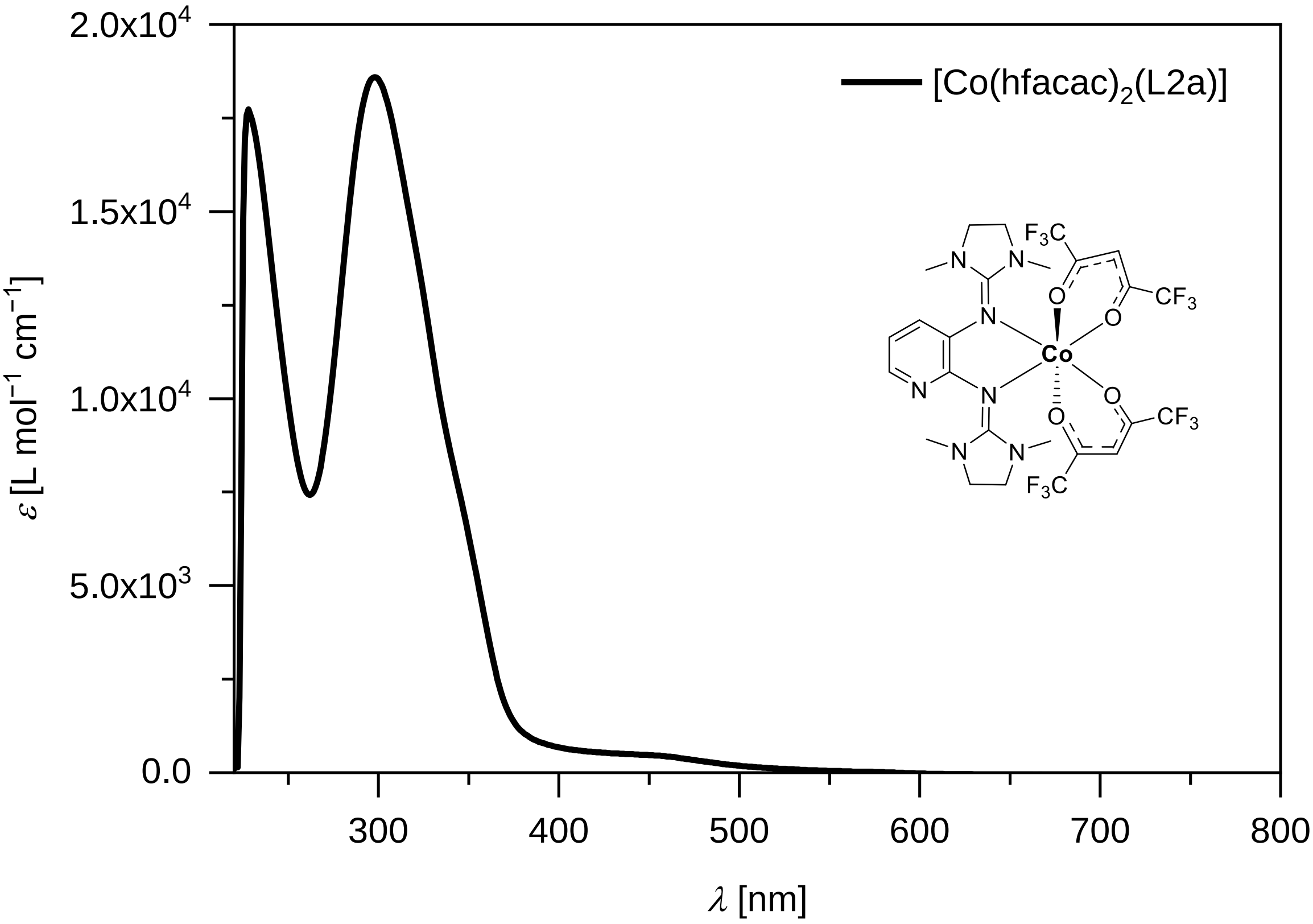

Figure S41: UV-vis absorption spectrum of [Co(hfac)$_2$(L2a)] in dichloromethane at a concentration of $2.67 \cdot 10^{-5}$ mol L^{-1} at rt.

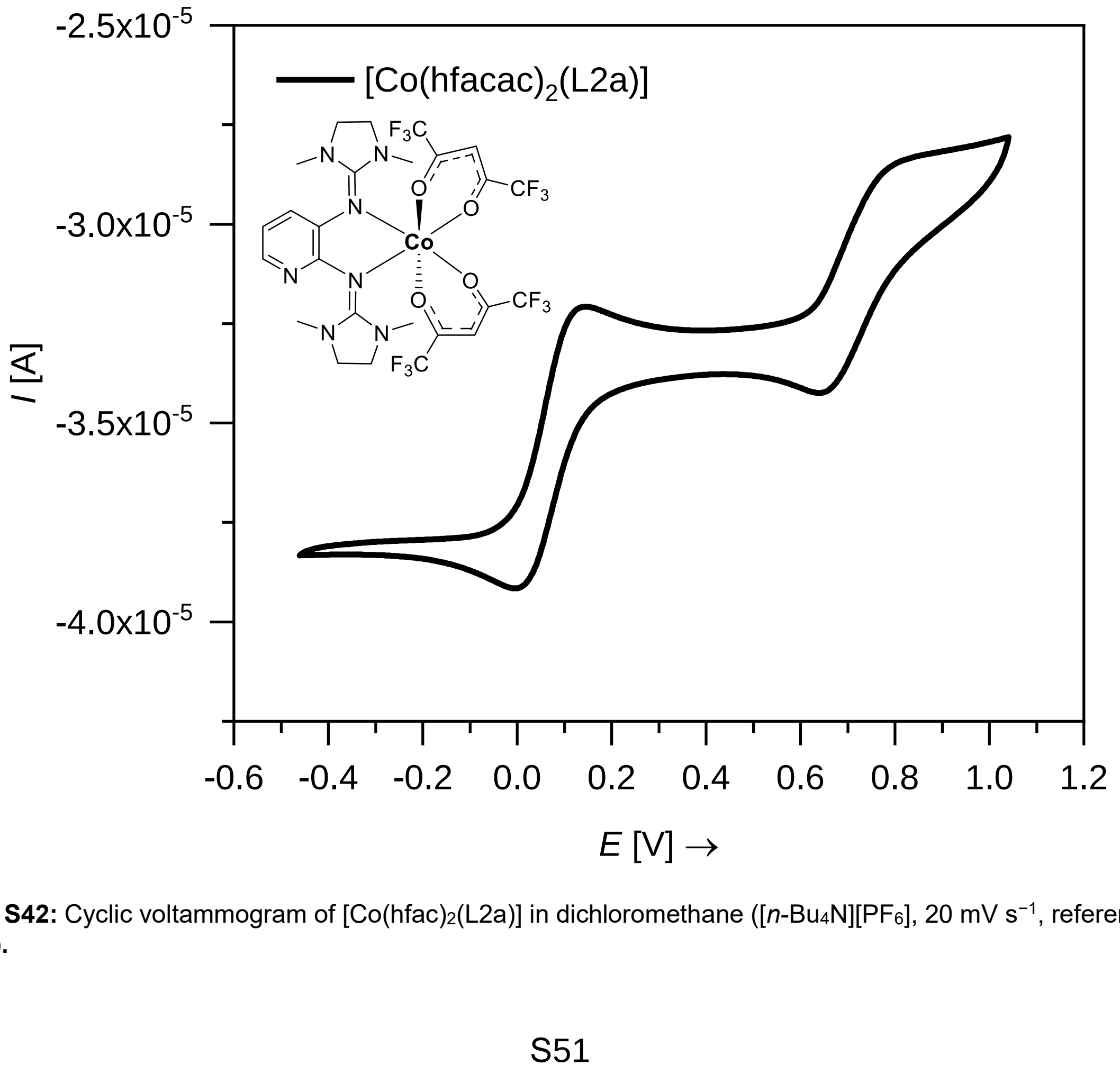

Figure S42: Cyclic voltammogram of [Co(hfac)$_2$(L2a)] in dichloromethane ([n-Bu_4N][PF_6], 20 mV s^{-1}, referenced to Fc/Fc^+).

2.2.5 [$CuBr_2$(L2a)]

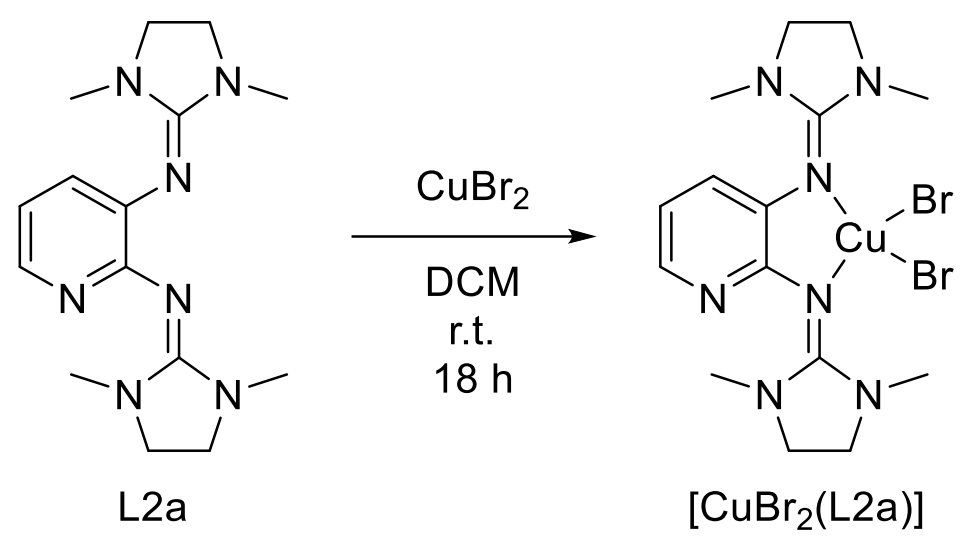

L2a (15.0 mg, 49.8 µmol, 1.00 eq.) was dissolved in dichloromethane (3 mL). Copper(II) bromide (11.1 mg, 49.8 µmol, 1.00 eq.) was added and the reaction mixture stirred for 18 h at r.t. while the mixture was turned dark green. The suspension was filtered through a syringe filter and overlayered with pentane. After eight days, the dark green crystals were filtered off and dried under reduced pressure, yielding the complex [$CuBr_2$(L2a)] (25.1 mg, 47.8 µmol, 96%).

UV-vis (DCM) ($3.94 \cdot 10^{-5}$ mol L^{-1})

λ (ε) = 665 (851), 414 (1930), 329 (10200), 298 (12900), 229 (18400) nm (M^{-1} cm^{-1}).

CV (DCM, [*n*-Bu_4N][PF_6], 20 mV s^{-1}, referenced to Fc/Fc^+)

$E_{1/2}^1$ = –413 mV,

E_{Ox}^1 = 248 mV, E_{Ox}^2 = 895 mV,

E_{Red}^1 = 239 mV.

Elemental analysis (C, H, N in %) ($C_{15}H_{23}Br_2CuN_7$)

calc.: C 34.33, H 4.42, N 18.68.

found: C 34.23, H 4.61, N 18.15.

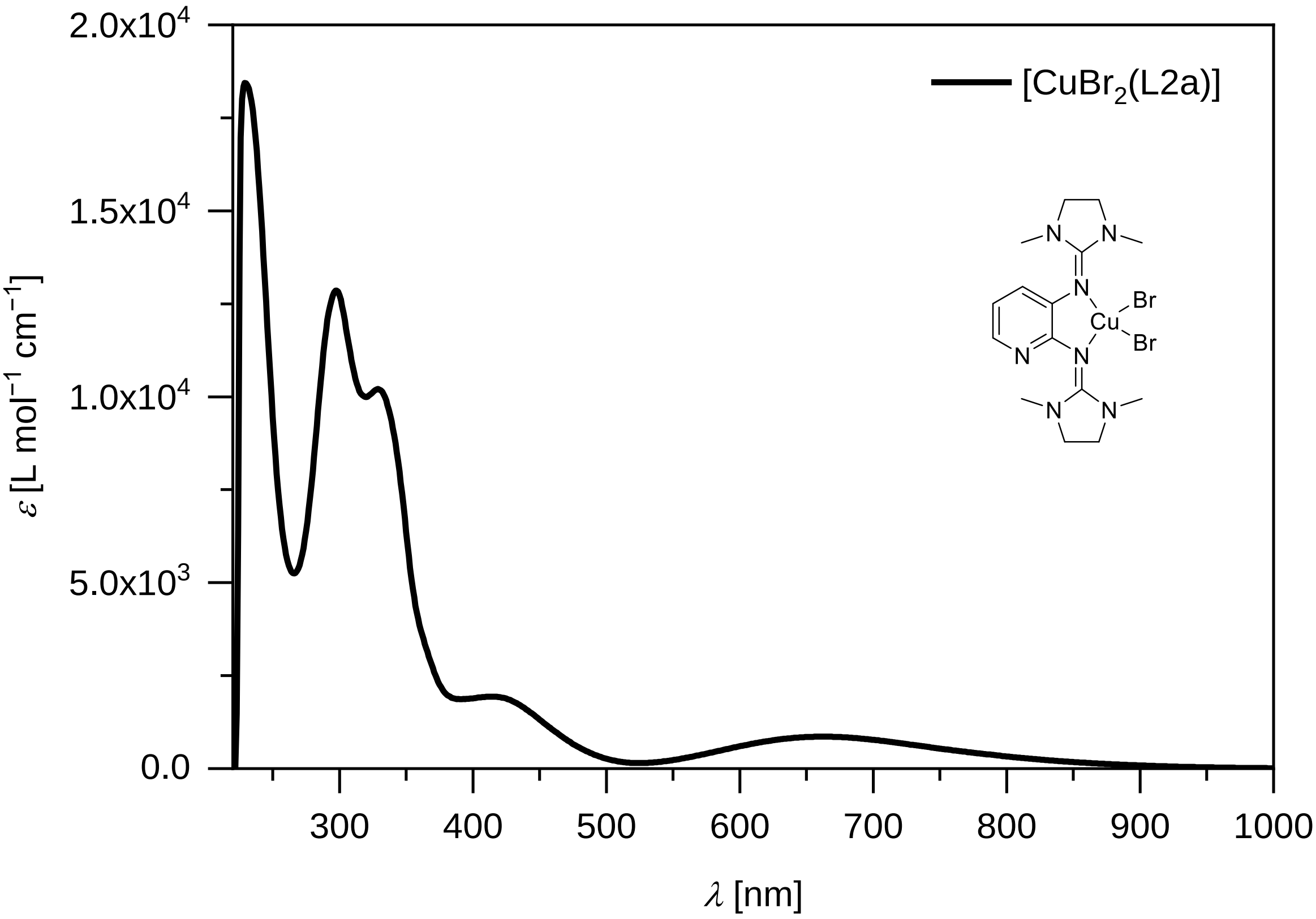

Figure S43: UV-vis absorption spectrum of [CuBr$_2$(L2a)] in dichloromethane at a concentration of $3.94 \cdot 10^{-5}$ mol L^{-1} at rt.

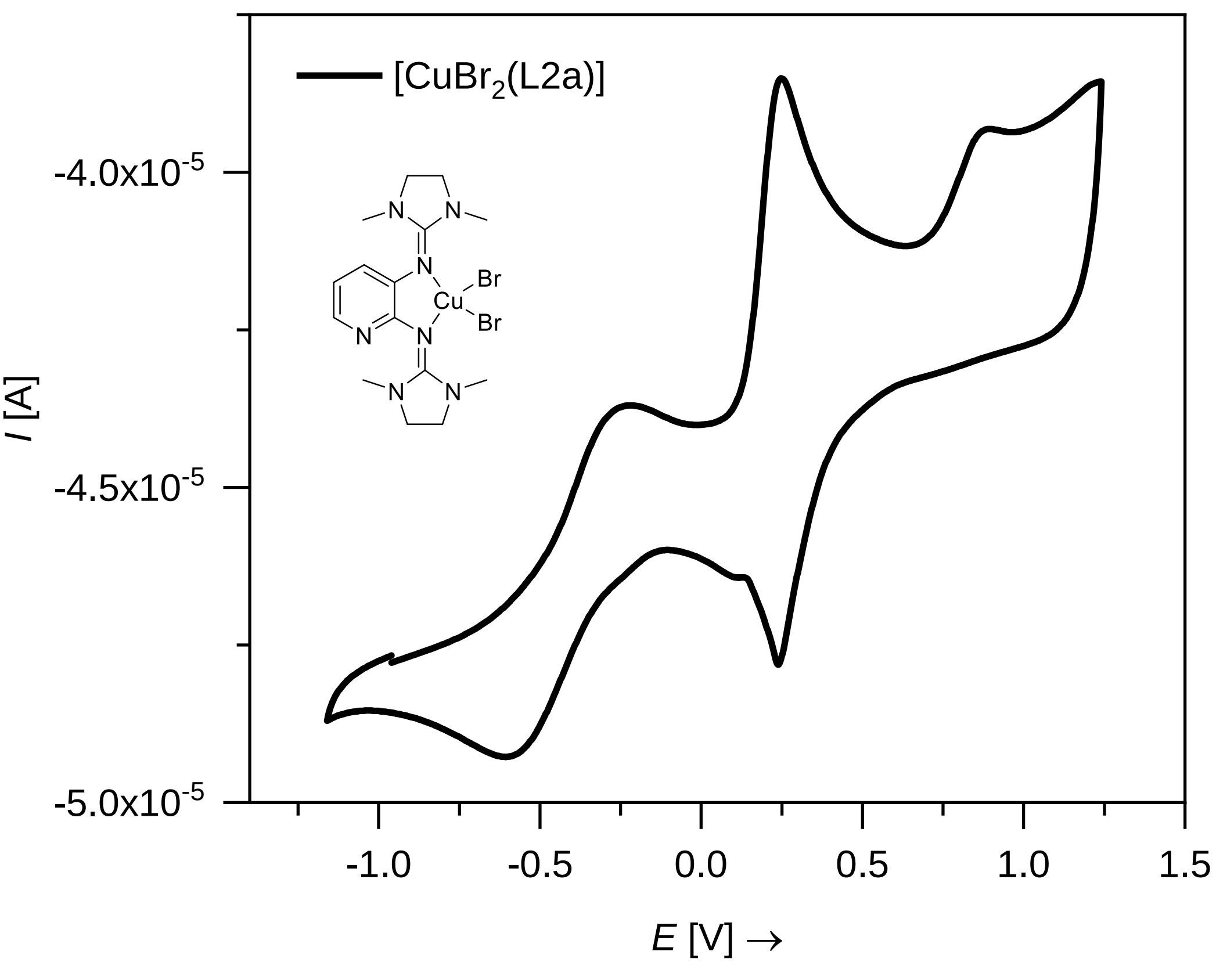

Figure S44: Cyclic voltammogram of [CuBr$_2$(L2a)] in dichloromethane ([*n*-Bu$_4$N][PF$_6$], 20 mV s^{-1}, referenced to Fc/Fc$^+$).

2.2.6 $[Cu(L2a)_2](BF_4)_2$

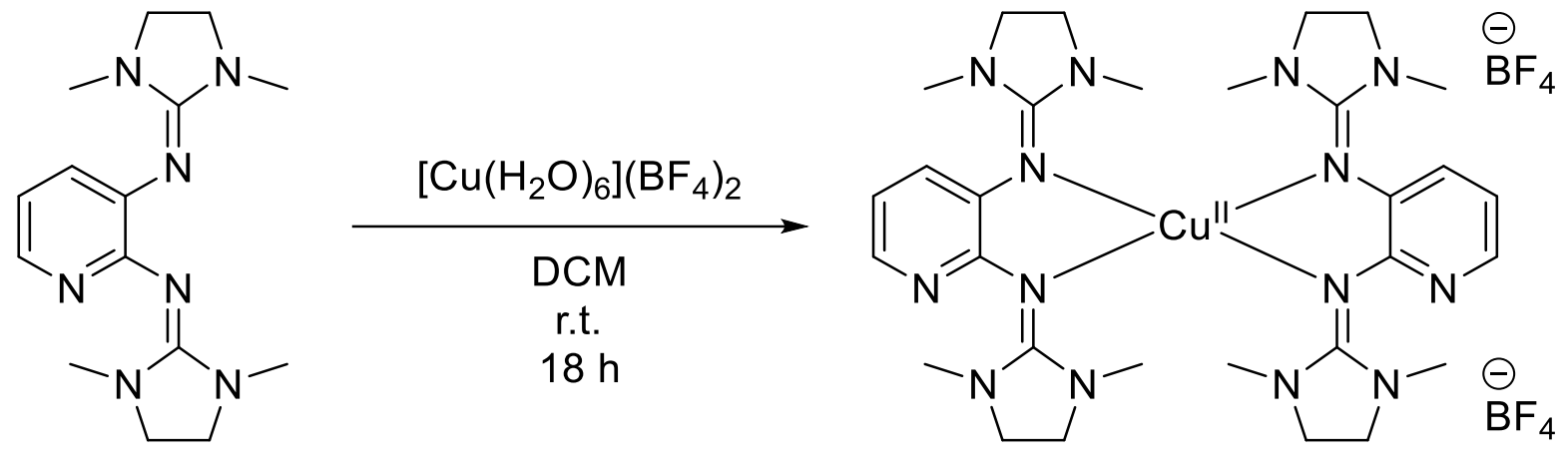

L2a (28.7 mg, 95.2 μmol, 2.25 eq.) and copper(II) tetrafluoroborate hexahydrate (14.6 mg, 42.3 μmol, 1.00 eq.) were dissolved in dichloromethane (3 mL). The reaction mixture was stirred for 18 h at r.t., slowly turning brown. The suspension was filtered through a syringe filter, and the solution was overlayered with diethyl ether. After seven days, the dark crystals were filtered off and dried. The complex $[Cu(L2a)_2](BF_4)_2$ (33.4 mg, 39.8 μmol. 94%) was obtained as a dark grey-green solid.

Mass spectrometry (ESI^+ in DCM)

m/z = 665.3320 ([**$L2a_2Cu$**]$^+$, calc. 665.3321).

UV-vis (DCM) ($3.89 \cdot 10^{-5}$ mol L^{-1})

λ (ε) = 328 (shoulder, 7460), 293 (10100), 228 (20400) nm (M^{-1} cm^{-1}).

CV (DCM, [n-Bu_4N][PF_6], 20 mV s^{-1}, referenced to Fc/Fc^+)

$E_{1/2}^1$ = –372 mV,

E_{Ox}^1 = –120 mV, E_{Ox}^2 = 129 mV, E_{Ox}^3 = 365 mV, E_{Ox}^4 = 512 mV,

E_{Red}^1 = 35.8 mV.

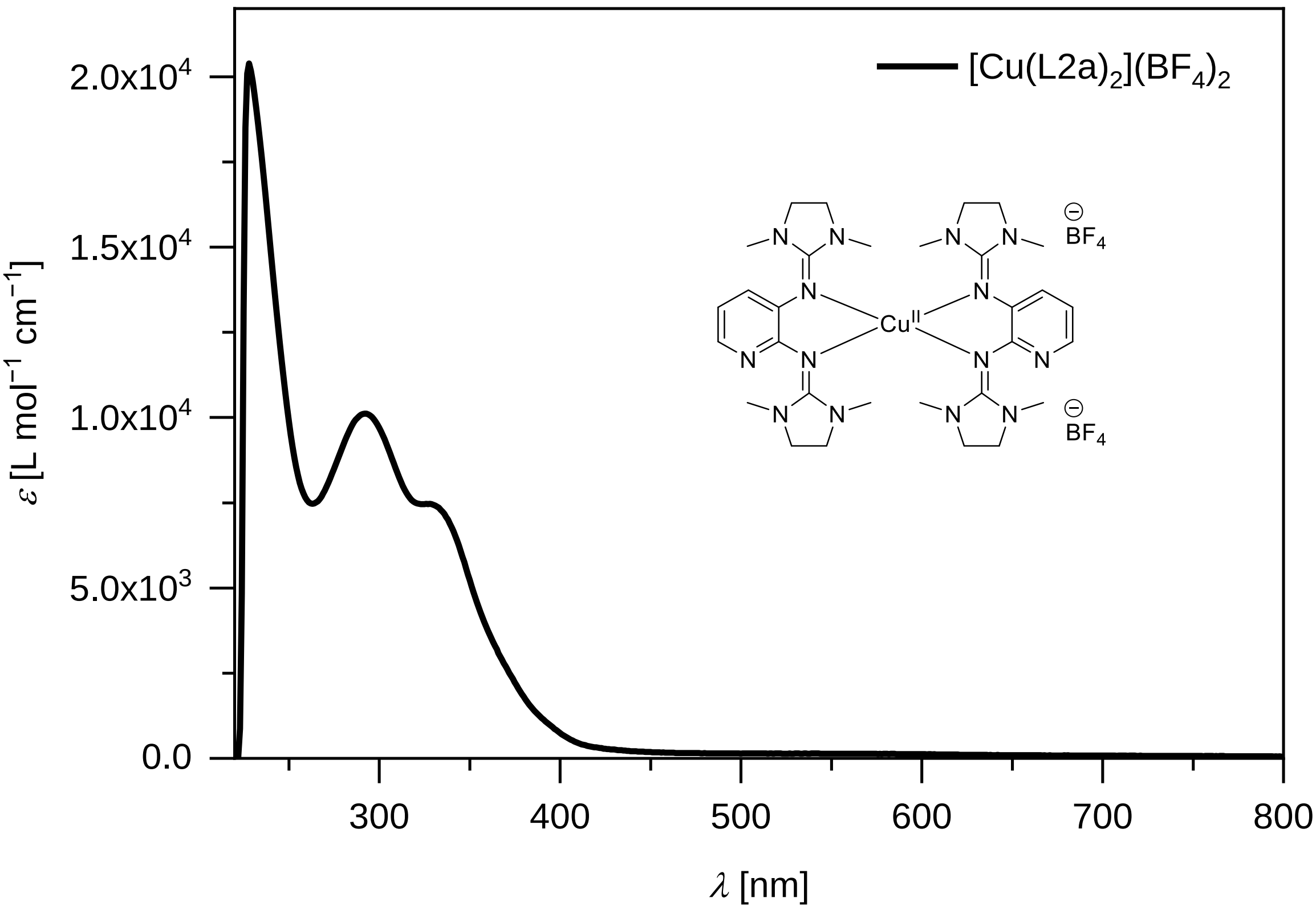

Figure S45: UV-vis absorption spectrum of $[Cu(L2a)_2](BF_4)_2$ in dichloromethane at a concentration of $3.89 \cdot 10^{-5}$ mol L^{-1} at rt.

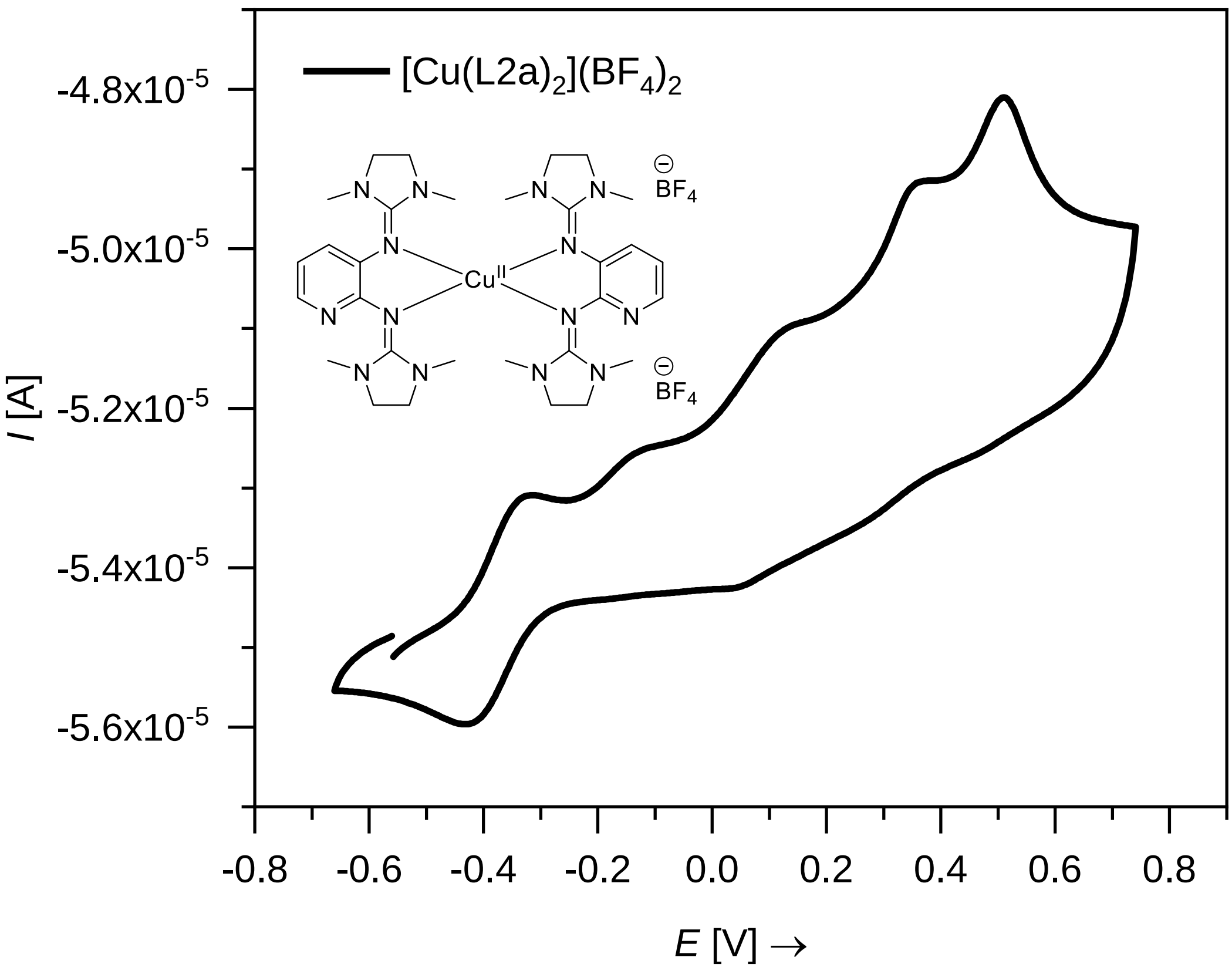

Figure S46: Cyclic voltammogram of $[Cu(L2a)_2](BF_4)_2$ in dichloromethane and acetonitrile ([*n*-Bu_4N][PF_6], 20 mV s^{-1}, referenced to Fc/Fc^{+}).

2.2.7 [$ZnCl_2$(L4)]

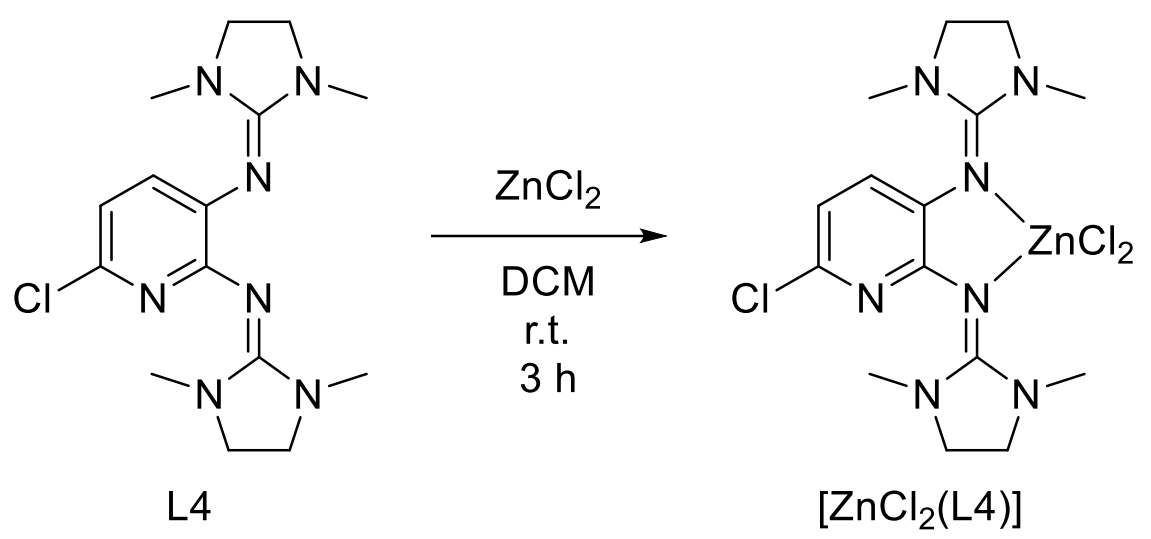

L4 (20.0 mg, 59.6 µmol, 1.00 eq.) was dissolved in dichloromethane (3 mL). Zinc(II) chloride (9.74 mg, 71.5 µmol, 1.20 eq.) was added and the reaction mixture stirred for 3 h at r.t. The solvent was removed and diethyl ether (5 mL) was added to dissolve impurities in the ultrasonic bath. The precipitate was filtered off to obtain [$ZnCl_2$(L4)] (27.2 mg, 59.6 µmol, 97%) as colourless solid.

^{1}H NMR (400 MHz, CD_3CN)

δ = 7.00 (d, J = 8.0 Hz, 1H, CH), 6.73 (d, J = 8.0 Hz, 1H, CH), 3.71 – 3.59 (m, 8H, CH_2), 2.86 (s, 6H, CH_3), 2.83 (s, 6H, CH_3) ppm.

^{13}C NMR (101 MHz, CD_3CN) **^{13}C-DEPT-135**

δ = 164.0 (1C, C_q), 163.6 (1C, C_q), 151.9 (1C, C_q), 139.2 (1C, C_q), 133.1 (1C, C_q), 128.5 (1C, CH), 114.9 (1C, CH), 48.8 (2C, CH_2), 48.4 (2C, CH_2), 35.6 (2C, CH_3), 35.0 (2C, CH_3) ppm.

Mass spectrometry (ESI^+ in DCM)

m/z = 336.1692 ([L4+H]$^+$, calc. 336.1698).

UV-vis (DCM) ($2.67 \cdot 10^{-5}$ mol L^{-1})

λ (ε) = 336 (11500), 293 (sh, 19800), 286 (21100), 231 (47100) nm (M^{-1} cm^{-1}).

CV (DCM, [*n*-Bu_4N][PF_6], 100 mV s^{-1}, referenced to Fc/Fc^+)

E_{Ox} = 490 mV,

E_{Red} = 177 mV.

Elemental analysis (C, H, N in %) ($C_{15}H_{22}Cl_3N_7Zn$ · 1 eq. Et_2O)

calc.: C 41.78, H 5.91, N 17.95.

found: C 40.83, H 5.42, N 17.34.

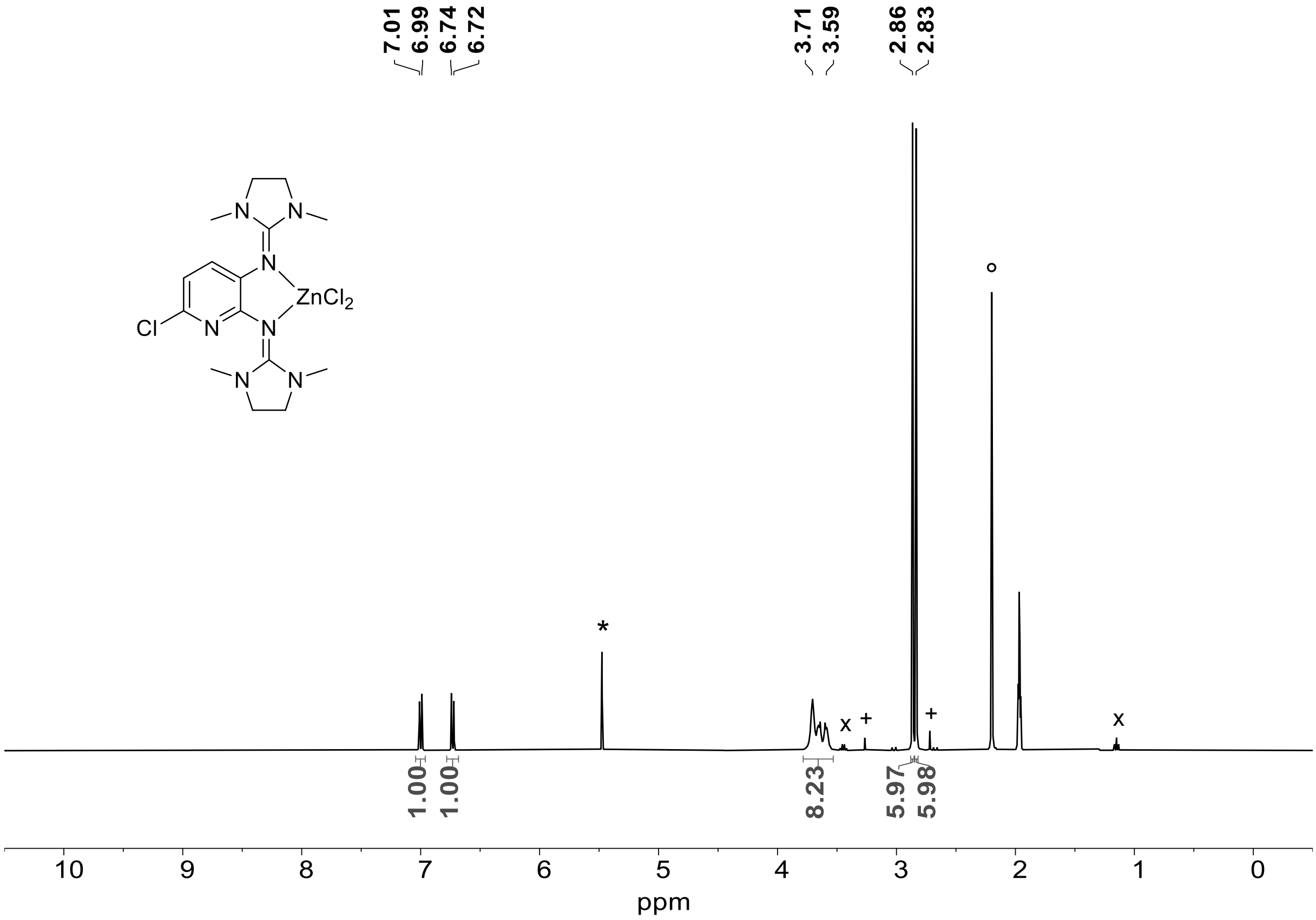

Figure S47: ^{1}H NMR spectrum of $[ZnCl_2(L4)]$ measured at 400 MHz in CD_3CN, *DCM, $^{x}Et_2O$ °water, $^{+}$deactivated 1.

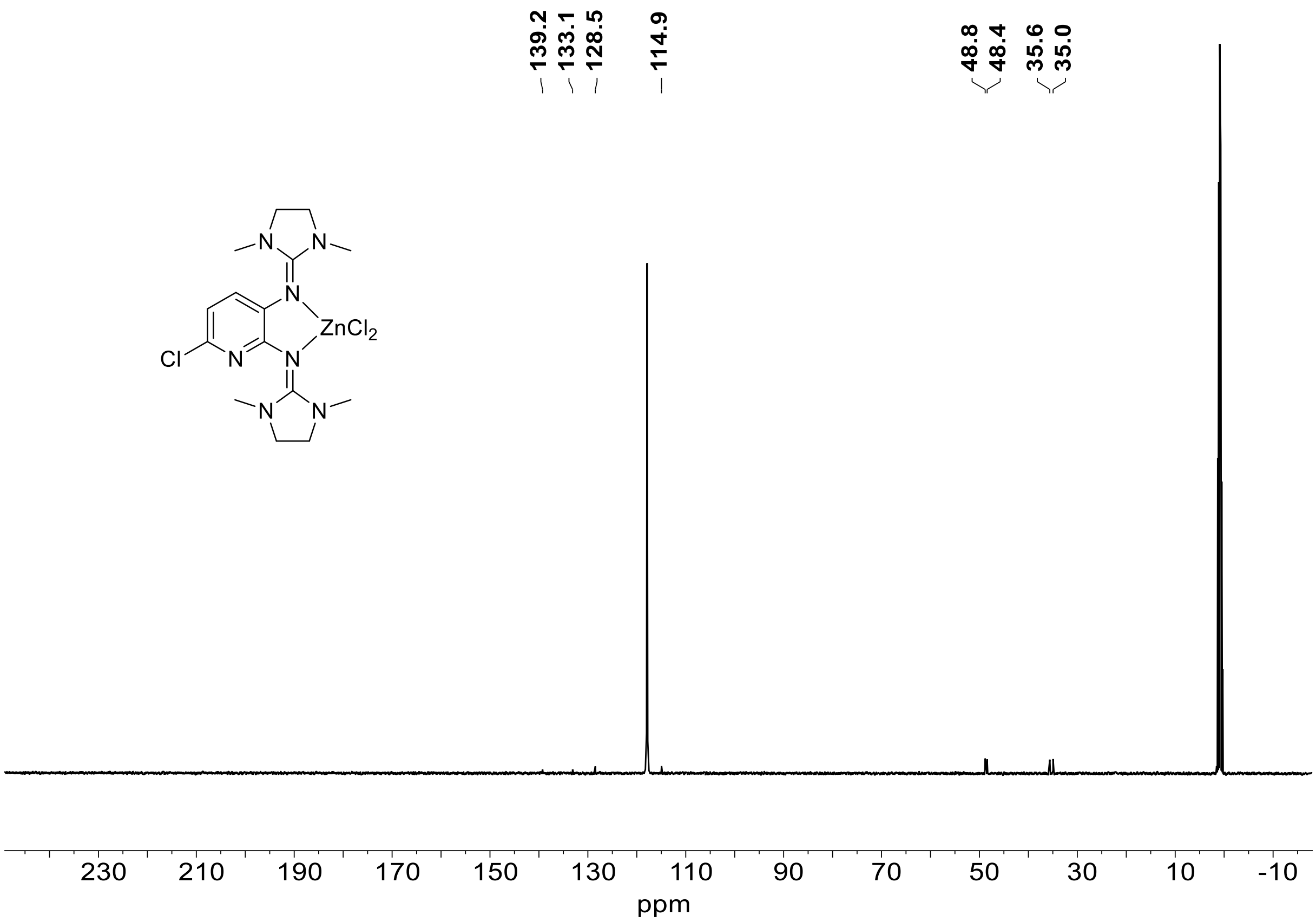

Figure S48: ^{13}C NMR spectrum of $[ZnCl_2(L4)]$ measured at 101 MHz in CD_3CN.

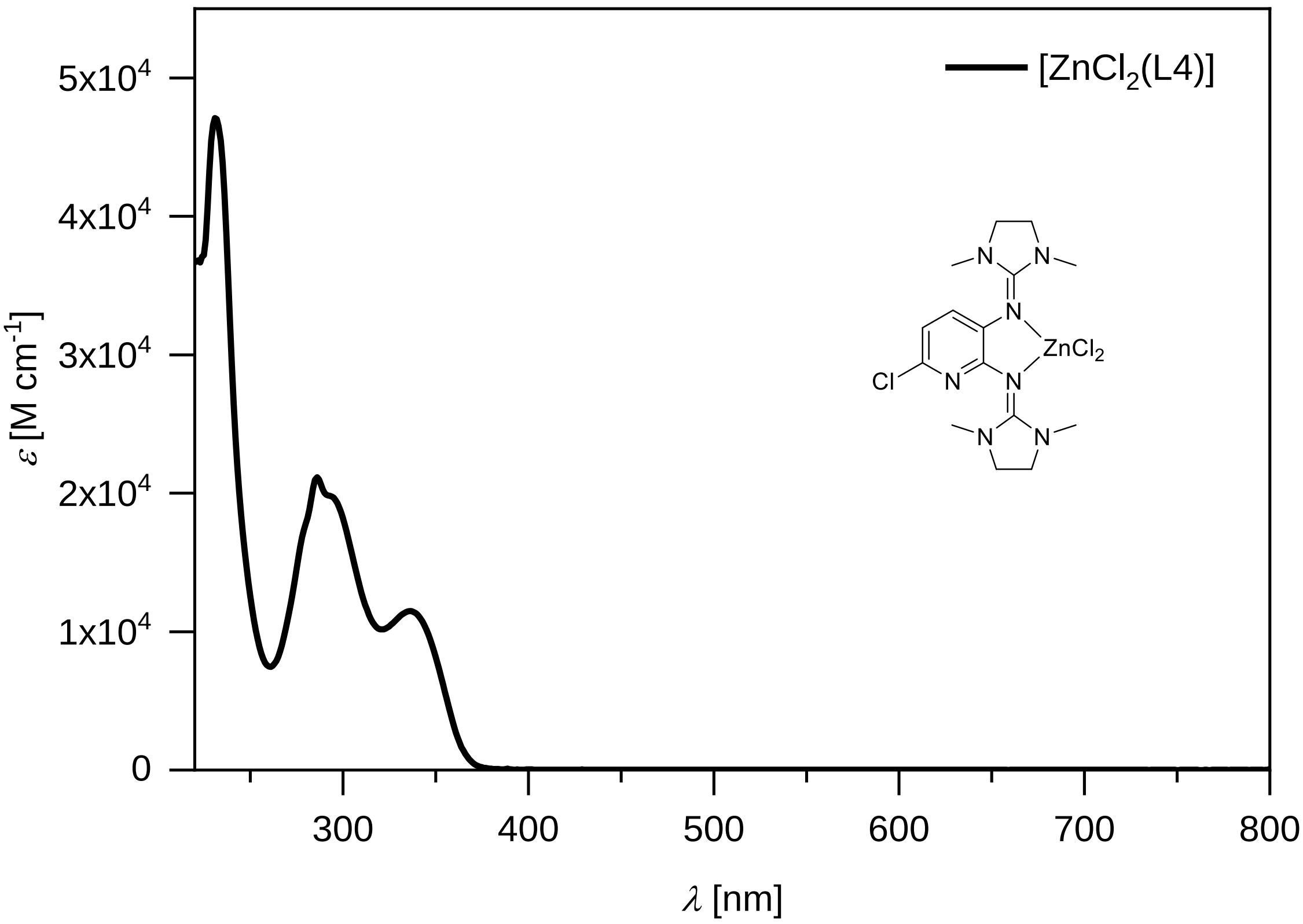

Figure S49: UV-vis absorption spectrum of [$ZnCl_2$(L4)] in dichloromethane at a concentration of $3.20 \cdot 10^{-5}$ mol L^{-1} at rt.

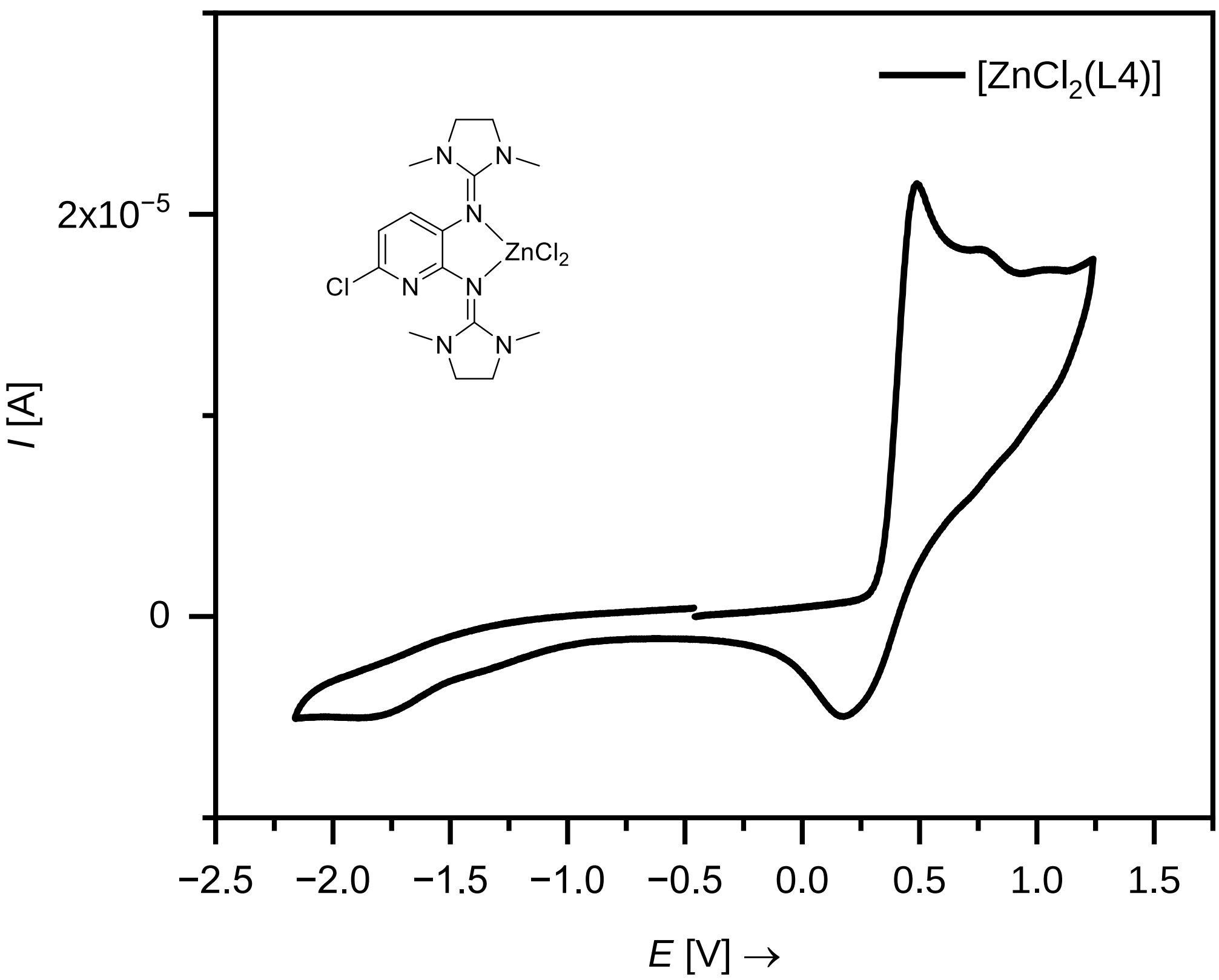

Figure S50: Cyclic voltammogram of [$ZnCl_2$(L4)] in dichloromethane ([n-Bu_4N][PF_6], 100 mV s^{-1}, referenced to Fc/Fc^+).

2.2.8 [$PdCl_2$(L6)]

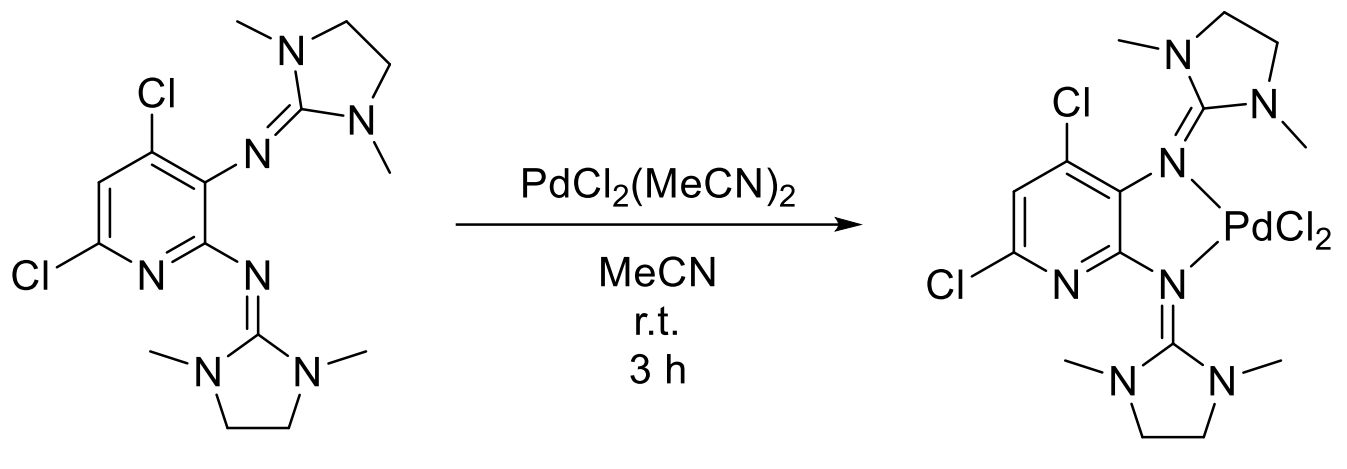

L6 (47.1 mg, 127 µmol, 1.00 eq.) was dissolved in acetonitrile (3 mL). Bis(acetonitril)dichlorpalladium(II) (33.0 mg, 127 µmol, 1.00 eq.) was added and the reaction mixture stirred for 3 h at r.t. The solvent was removed and diethyl ether (5 mL) was added to dissolve impurities in the ultrasonic bath. The precipitate was filtered off to obtain [$PdCl_2$(L6)] (67.0 mg, 122 µmol, 96%) as a brown solid.

A crystal suitable for single crystal structure analysis was obtained from a dichloromethane solution layered with diethyl ether.

^{1}H NMR (600 MHz, CD_3CN)

δ = 6.40 (s, 1H, CH), 3.77 – 3.69 (m, 4H, CH_2), 3.68 – 3.65 (m, 4H, CH_2), 2.97 (s, 6H, CH_3), 2.94 (s, 6H, CH_3) ppm.

^{13}C NMR (151 MHz, CD_3CN) **^{13}C-DEPT-135**

δ = 167.3 (1C, C_q), 166.6 (1C, C_q), 159.6 (1C, C_q), 137.6 (1C, C_q), 134.2 (1C, C_q), 127.4 (1C, C_q), 111.9 (1C, CH), 48.0 (2C, CH_2), 47.3 (2C, CH_2), 33.8 (2C, CH_3), 33.8 (2C, CH_3) ppm.

Mass spectrometry (ESI^+ in DCM)

m/z = 547.9697 ([[$PdCl_2$(L6)]+H]$^+$, calc. 547.9691).

UV-vis (DCM) ($2.67 \cdot 10^{-5}$ mol L^{-1})

λ (ε) = 567 (361), 417 (605), 349 (11400), 298 (12200), 285 (17400), 278 (16800), 232 (88400) nm (M^{-1} cm^{-1}).

CV (DCM, [*n*-Bu_4N][PF_6], 20 mV s^{-1}, referenced to Fc/Fc^+)

$E_{1/2}^1$ = 404 mV, $E_{1/2}^2$ = 938 mV.

Elemental analysis (C, H, N in %) ($C_{15}H_{21}Cl_4N_7Pd$)

calc.: C 32.90, H 3.87, N 17.91.

found: C 33.33, H 3.99, N 17.99.

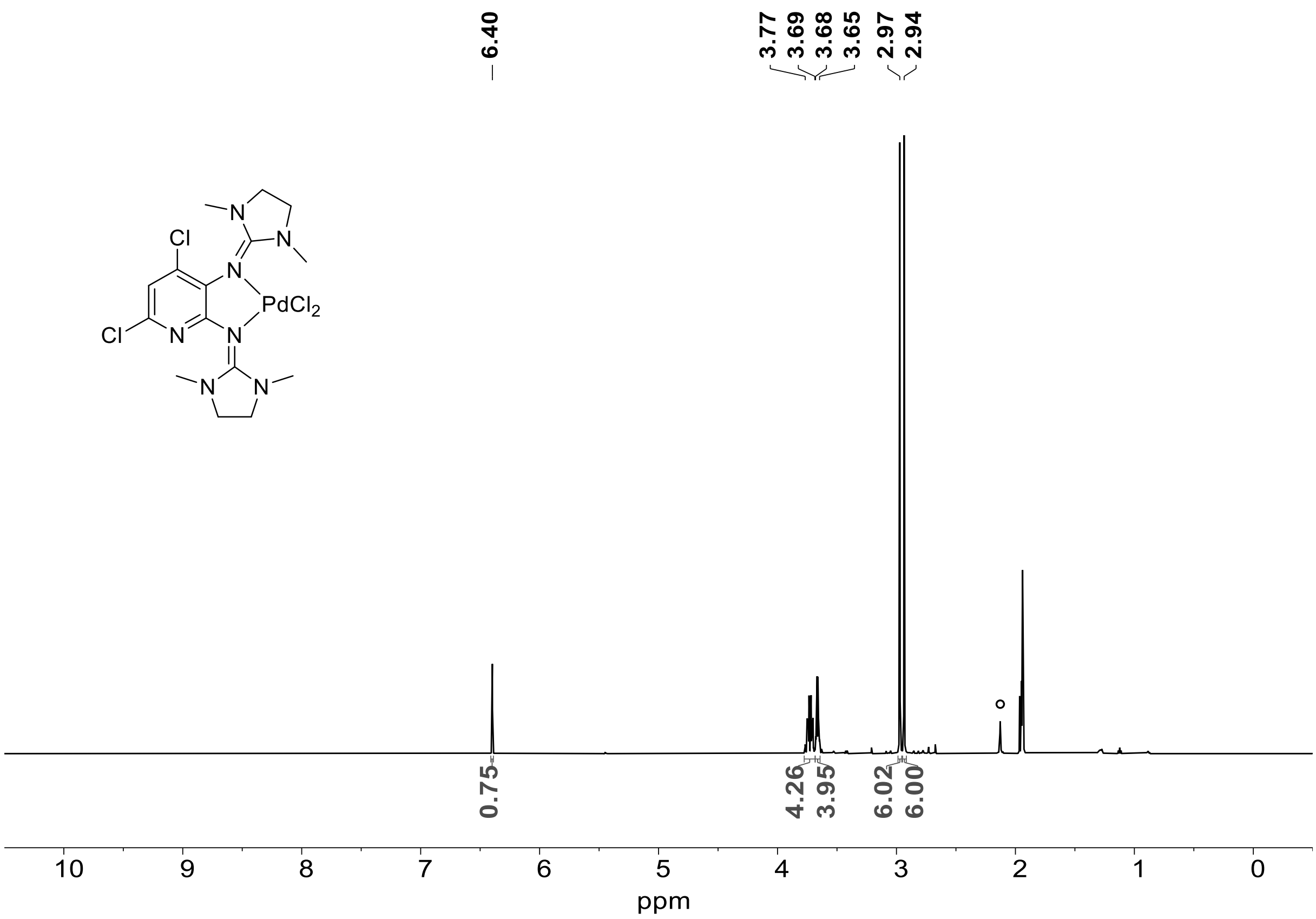

Figure S51: ^{1}H NMR spectrum of [$PdCl_2$(L6)] measured at 600 MHz in CD_3CN, °water.

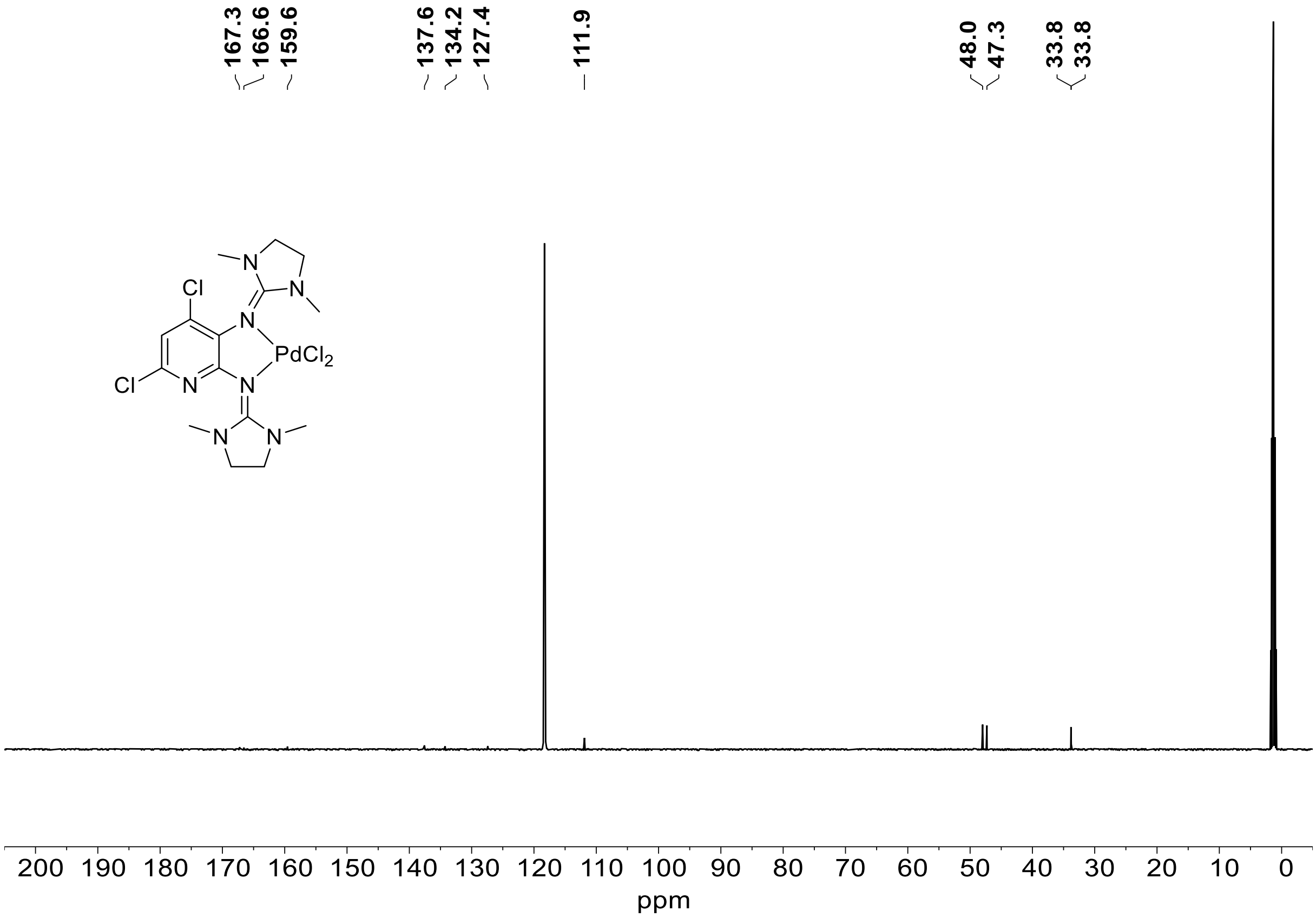

Figure S52: ^{13}C NMR spectrum of [$PdCl_2$(L6)], measured at 151 MHz in CD_3CN.

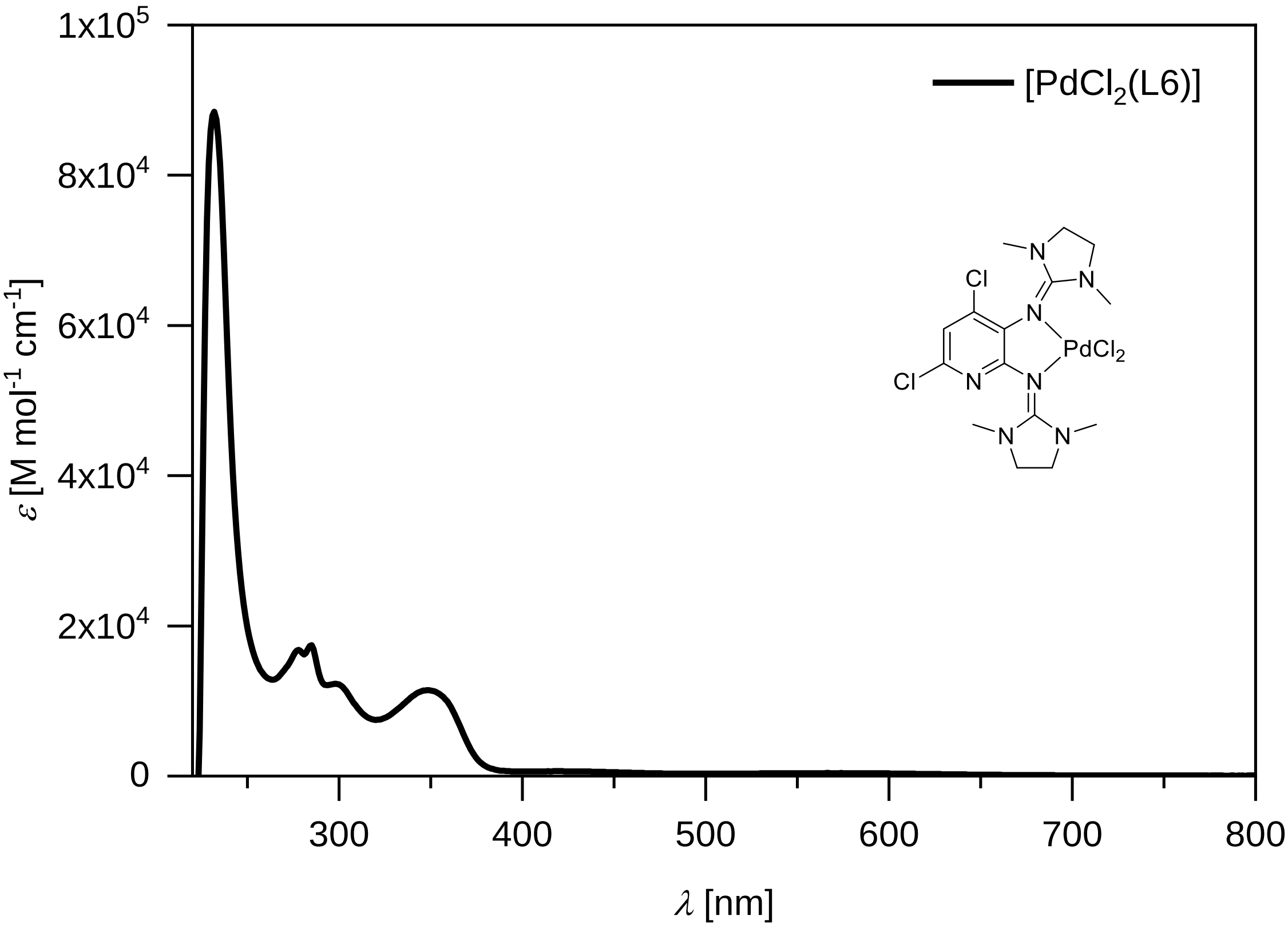

Figure S53: UV-vis absorption spectrum of $[PdCl_2(L6)]$ in dichloromethane at a concentration of $2.67 \cdot 10^{-5}$ mol L^{-1} at rt.

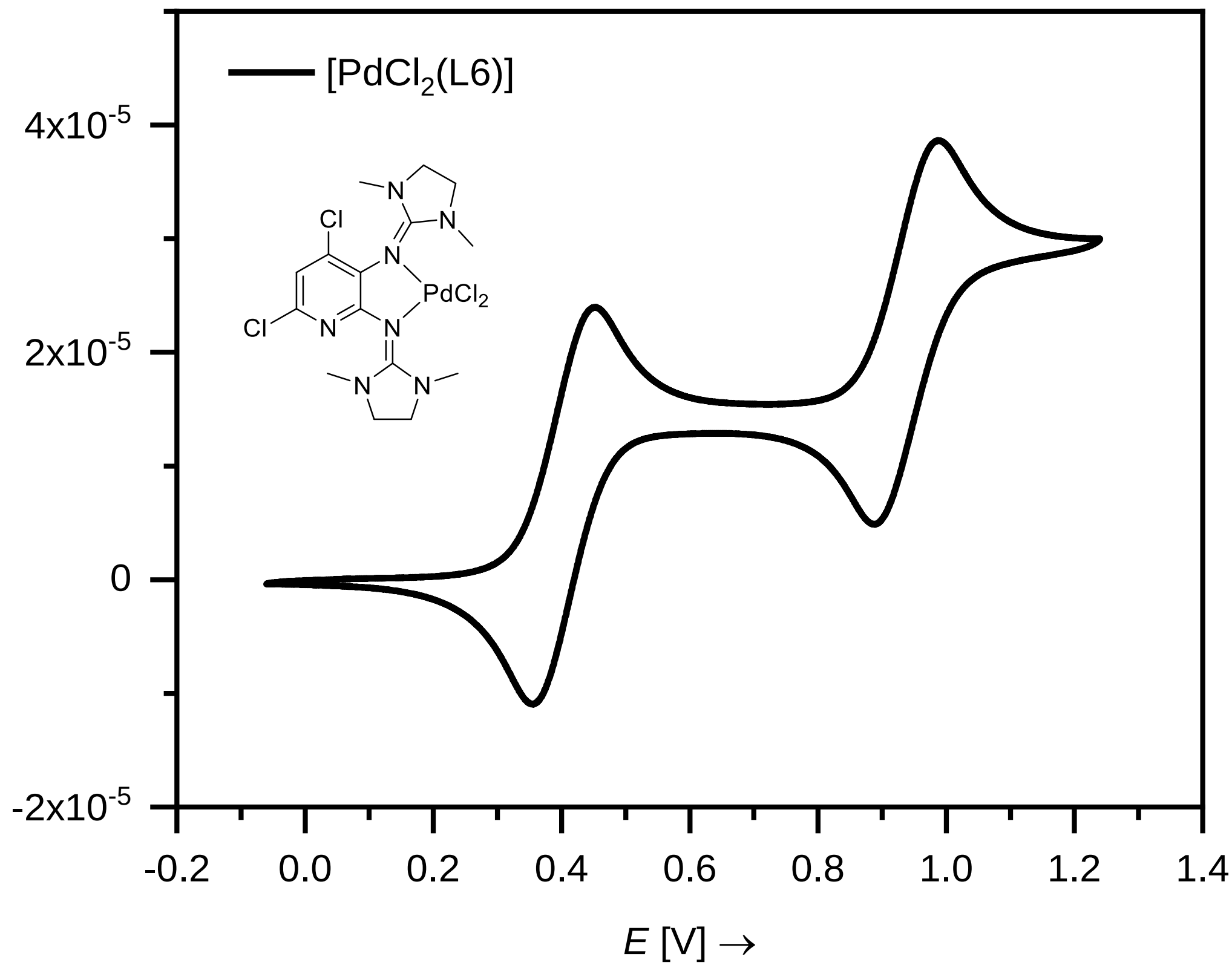

Figure S54: Cyclic voltammogram of $[PdCl_2(L6)]$ in dichloromethane ($[n\text{-}Bu_4N][PF_6]$, 20 mV s^{-1}, referenced to Fc/Fc^+).

2.2.9 [PdCl$_2$(L6)]PF$_6$

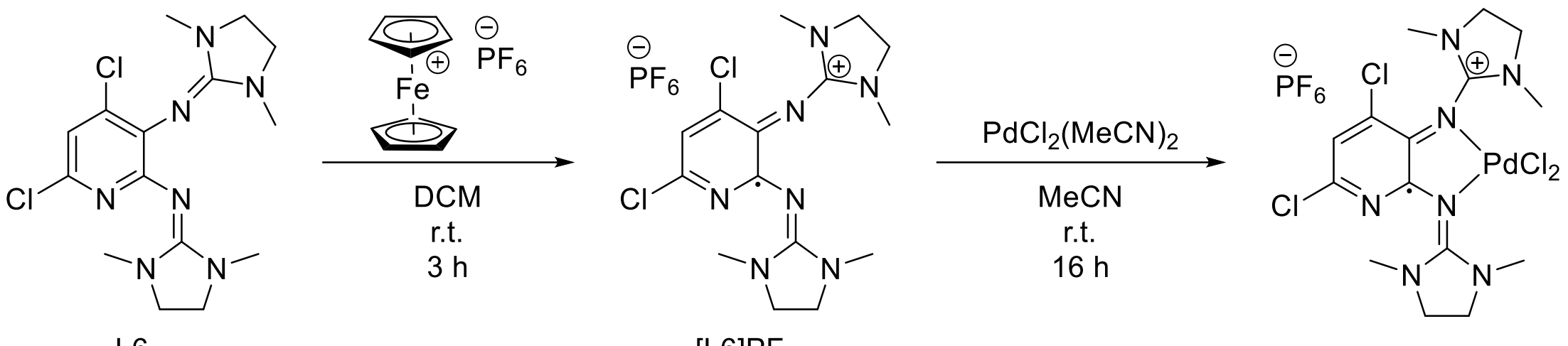

L6 (20.0 mg, 54.0 μmol, 1.00 eq.) and ferroceniumhexafluorophosphate (25.7 mg, 54.0 μmol, 1.00 eq.) were dissolved in 5 mL dichloromethane and the reaction mixture stirred 3 h at room temperature. After solvent removal under reduced pressure, the solid was washed with diethyl ether (2 x 10 mL) and dried. Bis(acetonitrile)dichloropalladium(II) (14.0 mg, 54 μmol, 1.00 eq.) was added, the solid dissolved in 5 mL acetonitrile and the solution stirred for 16 h at room temperature. Then, the solvent was removed and diethyl ether (5 mL) was added to dissolve impurities in the ultrasonic bath. The precipitate was filtered off to obtain [PdCl$_2$(L6)]PF$_6$ (40.2 mg, 48.0 μmol, 89%) as a brown solid.

Mass spectrometry (ESI$^+$ in DCM)

m/z = 547.9697 ([[PdCl$_2$(L6)]+H]$^+$, calc. 547.9691).

UV-vis spectrum (DCM) (4.14·10^{-5} mol L^{-1})

λ (ε) = 352 (8660), 294 (12300), 230 (37500) nm (M^{-1} cm^{-1}).

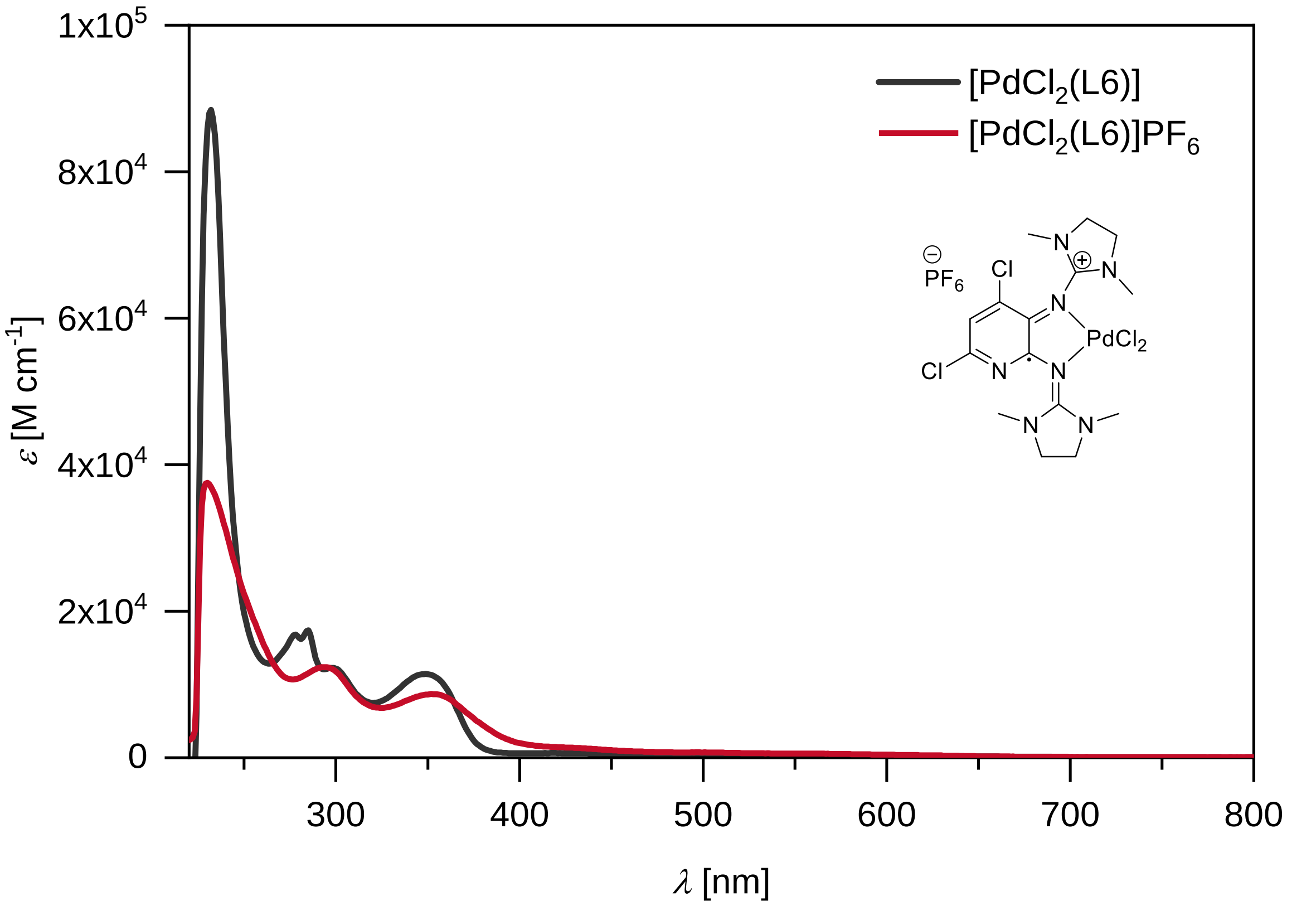

Figure S55: UV-vis absorption spectrum of $[PdCl_2(L6)]$ and $[PdCl_2(L6)]PF_6$ in dichloromethane at a concentration of $4.14 \cdot 10^{-5}$ mol L^{-1} at rt.

2.3 Pyridinium salts

2.3.1 General procedure 1

The guanidine ligand L was dissolved in diethyl ether (10 mL). The organohalide was added and the reaction solution was stirred for 18 h under exclusion of light. Half of the solvent was removed under reduced pressure, and the precipitated solid was then filtered off and washed with diethyl ether (2 · 10 mL). After drying under reduced pressure, the product was obtained.

2.3.2 General procedure 2

The guanidine ligand L was dissolved in dichloromethane (2 mL). The organohalide was added and the reaction solution was stirred for 18 h under exclusion of light. The solvent was removed and the residue was washed with diethyl ether (10 mL) in ultrasound. After drying under reduced pressure, the product was obtained.

2.3.3 [L2a+Me]I

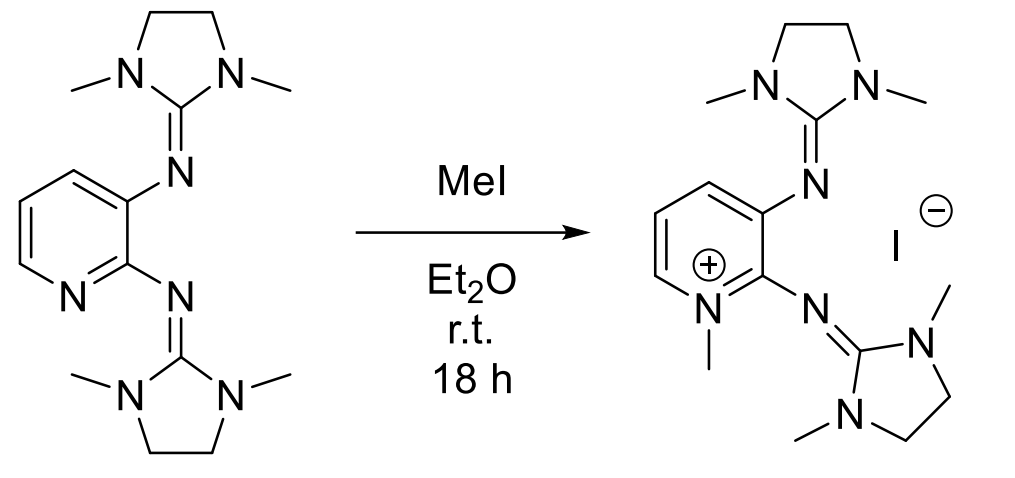

Following general procedure 1, L2a (45.0 mg, 149µmol, 1.00 eq.) and methyl iodide (10.2 µL, 23.3 mg, 164 µmol, 1.10 eq.) were used to obtain [L2a+Me]**I** (65.0 mg, 147 µmol, 98%) as a colourless solid.

A crystal suitable for single crystal structure analysis was obtained from an acetonitrile solution by layering with diethyl ether.

^{1}H NMR (600 MHz, CD_3CN)

δ = 7.21 (dd, *J* = 6.6, 1.5 Hz, 1H), 6.75 (dd, *J* = 7.6, 1.6 Hz, 1H), 6.47 (dd, *J* = 7.5, 6.6 Hz, 1H), 3.68 (s, 3H), 3.59-3.47 (m, 4H), 3.33 (s, 4H), 2.70 (s, 6H), 2.63 (s, 6H). ppm.

Mass spectrometry (ESI+ in DCM)

m/z = 316.2227 ($[L2a+Me]^+$, calc. 316.2250),

m/z = 158.6146 ($[L2a+Me]^{2+}$, calc. 158.6136).

CV (DCM, [*n*-Bu_4N][PF_6], 20 mV s^{-1}, referenced to Fc/Fc^+)

E_{Ox}^1 = 104 mV, E_{Ox}^2 = 375 mV,

E_{Red}^1 = 167 mV.

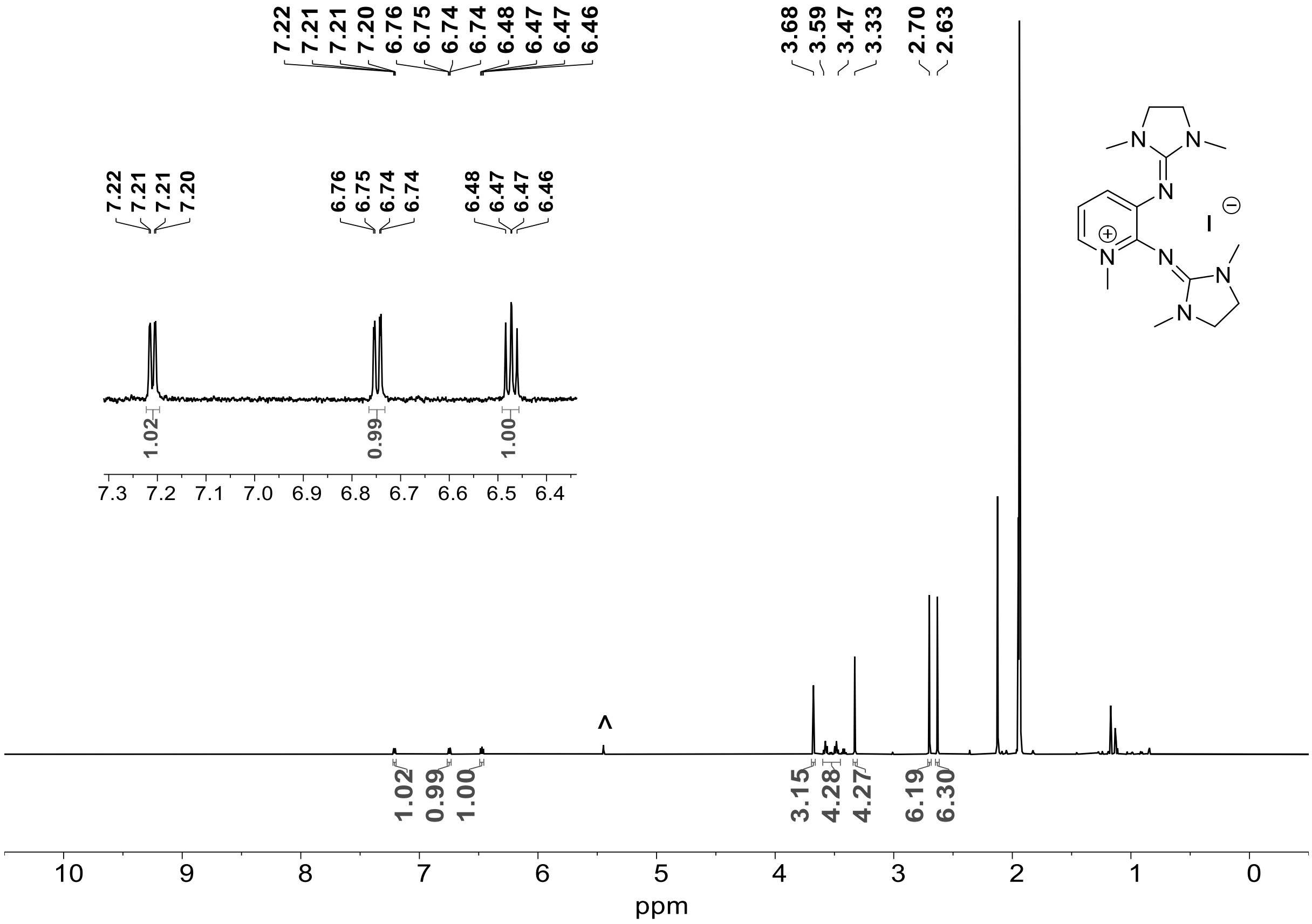

Figure S56: ^{1}H NMR spectrum of [L2a+Me]I, measured at 600 MHz in CD_3CN, ^DCM.

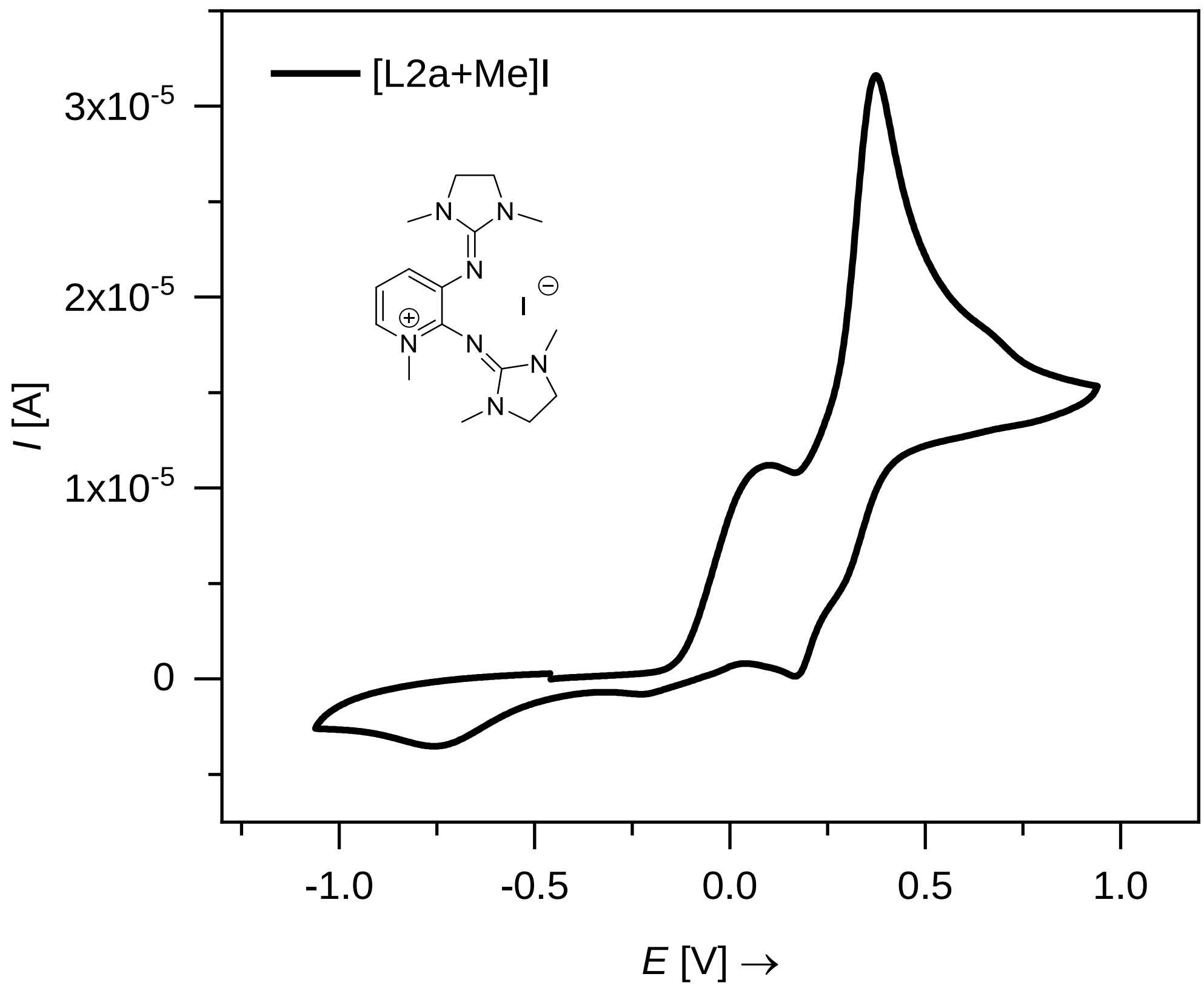

Figure S57: Cyclic voltammogram of [L2a+Me]I in dichloromethane ([n-Bu_4N][PF_6], 20 mV s^{-1}, referenced to Fc/Fc^+).

2.3.4 [L2a+Aly]Br

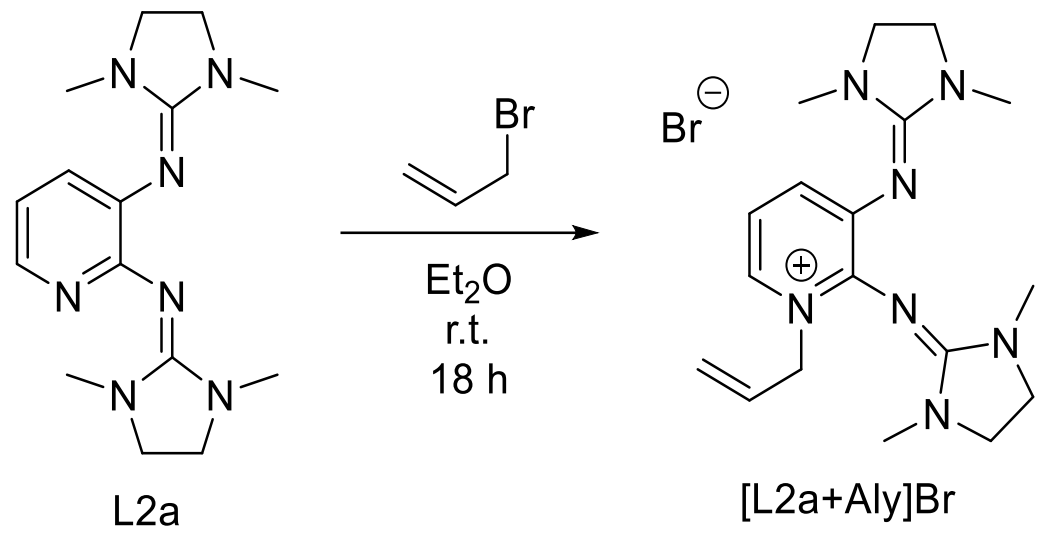

Following general procedure 1, L2a (50.0 mg, 166 μmol, 1.00 eq.) and allyl bromide (15.8 μL, 22.1 mg, 182 μmol, 1.10 eq.) were used to obtain [L2a+Aly]Br (59.0 mg, 140 μmol, 84%) as a colourless solid.

A Crystal for single crystal structure analysis was obtained from an acetonitrile solution by layering with diethyl ether.

^{1}H NMR (400 MHz, CD_3CN)

δ = 7.20 (dd, *J* = 6.6, 1.5 Hz, 1H), 6.74 (dd, *J* = 7.5, 1.5 Hz, 1H), 6.51 (dd, *J* = 7.5, 6.6 Hz, 1H), 6.04 (ddt, *J* = 17.3, 10.5, 5.5 Hz, 1H), 5.27 (dq, *J* = 10.4, 1.4 Hz, 1H), 5.14 (dq, *J* = 17.3, 1.7 Hz, 1H), 4.76 (dt, *J* = 5.5, 1.6 Hz, 2H), 3.53 (d, *J* = 57.7 Hz, 4H), 3.33 (s, 4H), 2.68 (s, 6H), 2.63 (s, 6H) ppm.

^{13}C{^{1}H} NMR (101 MHz, CD_3CN), **^{13}C-DEPT-135**, **HMBC**, **HSQC**

δ = 163.5 (1C, C_q), 158.5 (1C, C_q), 151.8 (1C, C_q), 139.5 (1C, C_q), 132.9 (1C, CH), 129.5 (1C, CH), 125.3 (1C, CH), 118.7 (1C, CH_2), 111.6 (1C, CH), 56.1 (1C, CH_2), 48.9 (2C, CH_2), 47.9 (2C, CH_2), 35.1 (2C, CH_3), 32.9 (2C, CH_3) ppm.

Mass spectrometry (ESI$^+$ in DCM)

m/z = 342.2402 ([L2a+Aly]$^+$, calc. 342.2401).

UV-vis (DCM) ($5.95 \cdot 10^{-5}$ mol L^{-1})

λ (ε) = 363 (6080), 303 (4500), 259 (2340), 234 (5350) nm (M^{-1} cm^{-1}).

Elemental analysis (C, H, N in %) ($C_{18}H_{28}BrN_7$ · 0.2 eq. DCM)

calc.: C 49.75, H 6.52, N 22.32.

found: C 49.67, H 6.59, N 22.25.

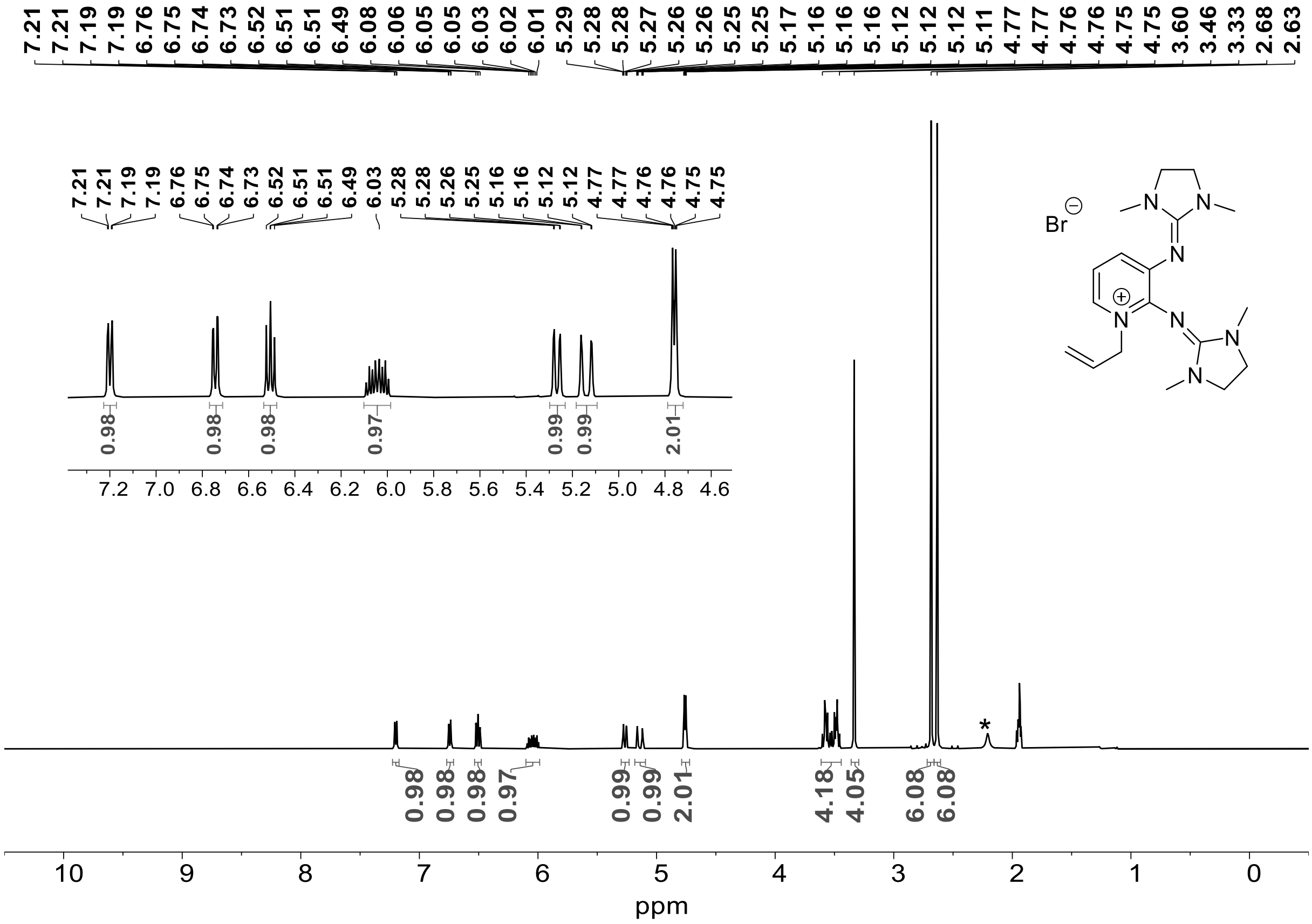

Figure S58: ^{1}H NMR spectrum of [L2a+Aly]Br, measured at 400 MHz in CD_3CN, *water.

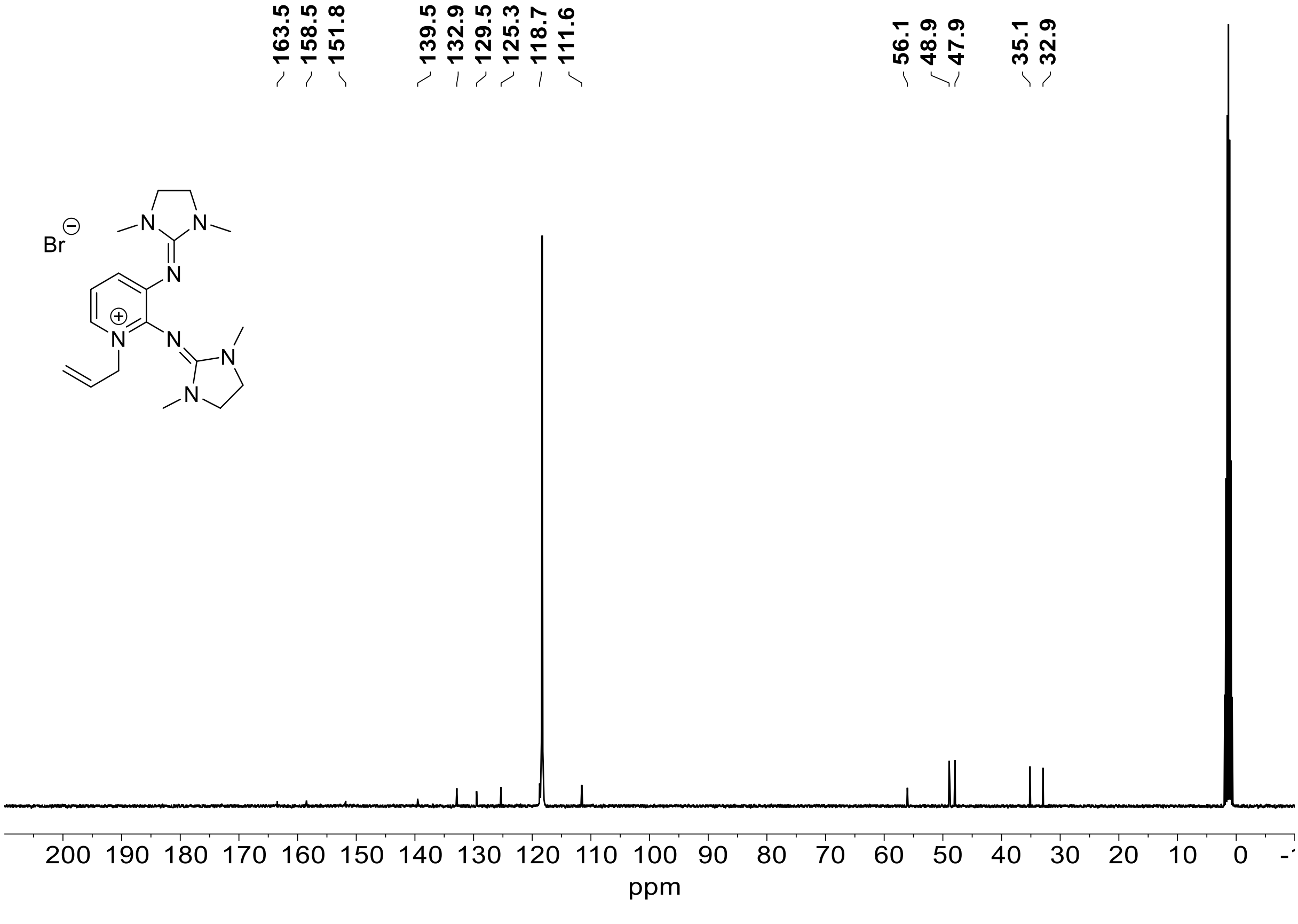

Figure S59: ^{13}C NMR spectrum of [L2a+Aly]Br, measured at 101 MHz in CD_3CN.

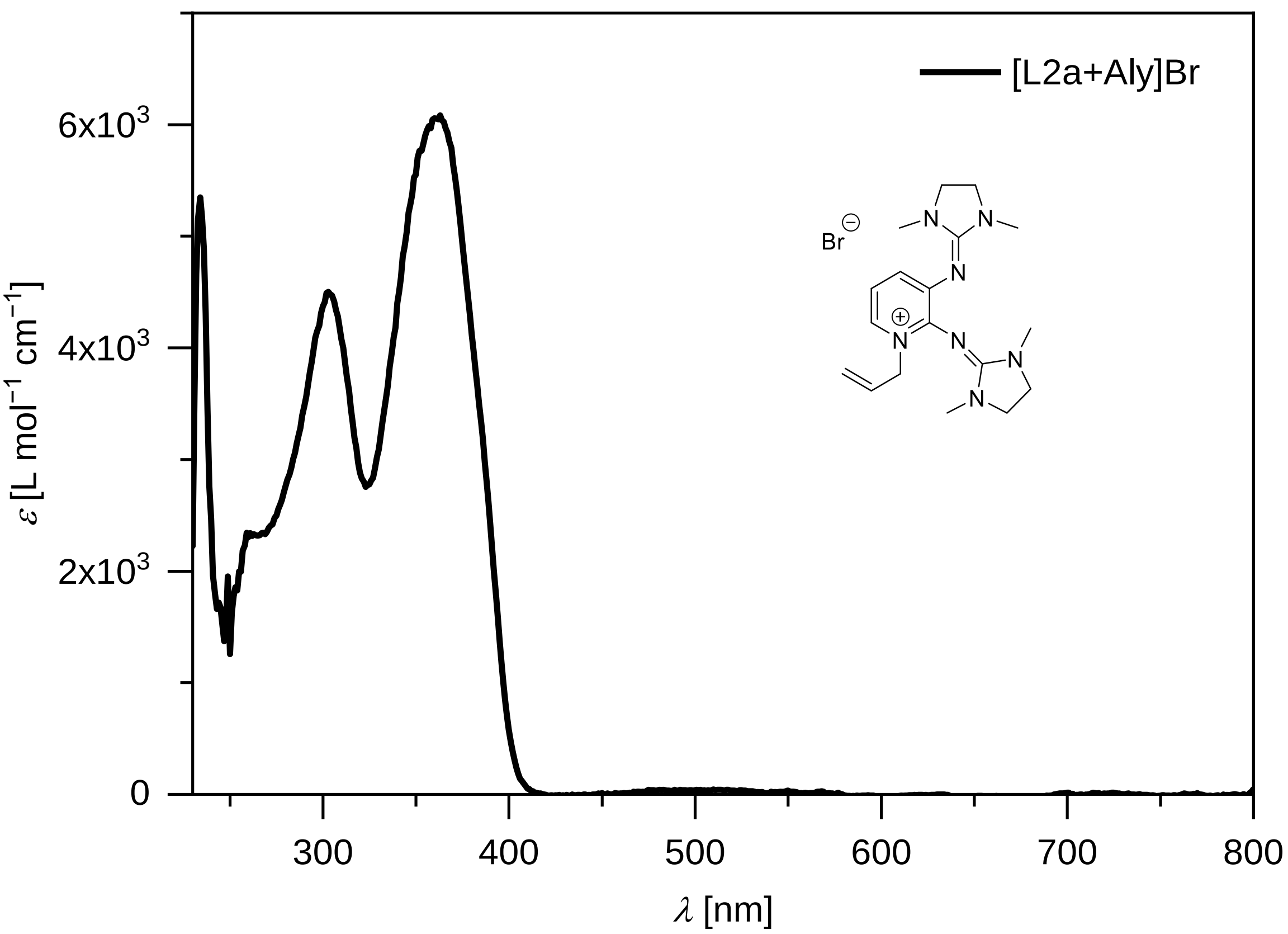

Figure S60: UV-vis absorption spectrum of [L2a+Aly]Br in dichloromethane at a concentration of $5.95 \cdot 10^{-5}$ mol L^{-1} at rt.

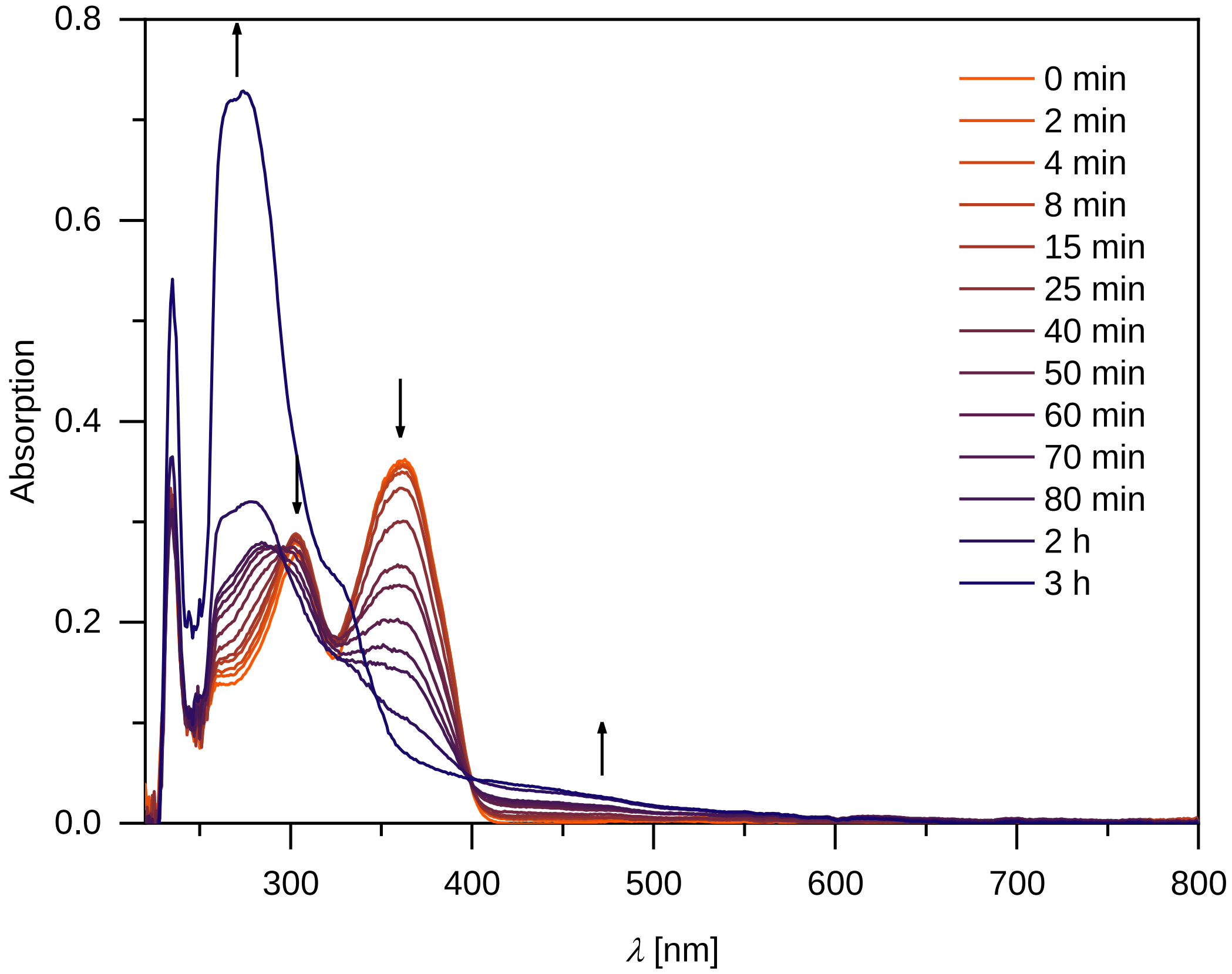

Figure S61: UV-vis absorption spectrum of [L2a+Aly]Br in dichloromethane at a concentration of $5.95 \cdot 10^{-5}$ mol L^{-1} at rt under irradiation with broad spectrum visible light.

2.3.5 [L2a+Bn]Br

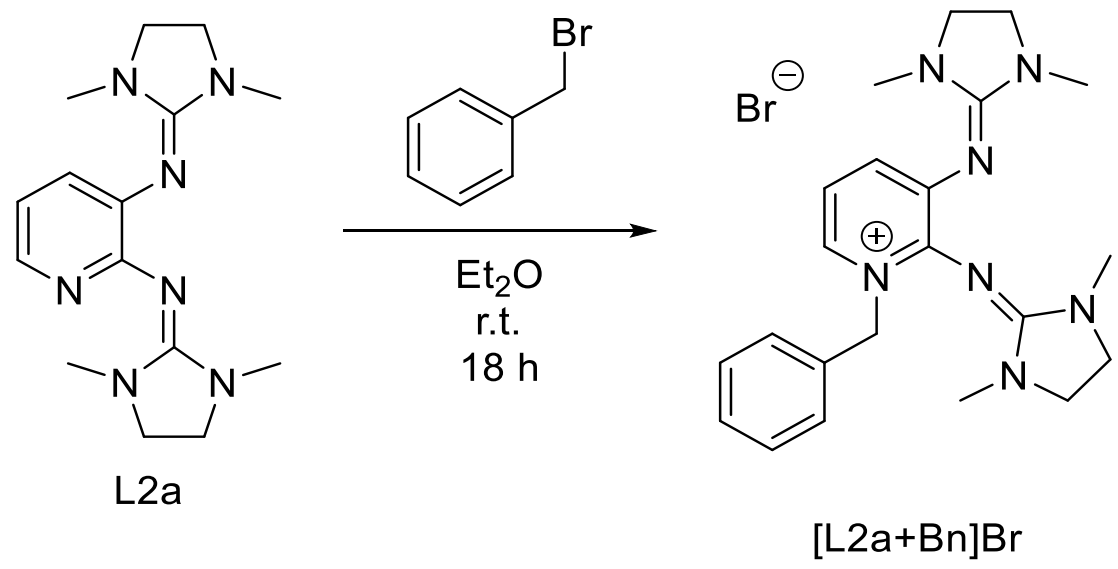

Following general procedure 1, L2a (20.5 mg, 68.1 μmol, 1.00 eq.) and benzyl bromide (16.2 μL, 23.3 mg, 136 μmol, 2.00 eq.) were used to obtain [L2a+Bn]Br (31.7 mg, 67.1 μmol, 99%) as a colourless solid.

A Crystal for single crystal structure analysis was obtained from an acetonitrile solution by layering with diethyl ether.

^{1}H-NMR (600.15 MHz, CD_3CN)

δ = 7.41 (dd, *J*=6.6, 1.6, 1H, CH), 7.37-7.34 (m, 2H, CH), 7.32-7.29 (m, 1H, CH), 7.27-7.25 (m, 2H, CH), 6.76 (dd, *J*=7.6, 1.5, 1H, CH), 6.55 (dd, *J*=7.5, 6.6, 1H, CH_2), 5.33 (s, 2H, CH_2), 3.52-3.41 (m, 4H, CH_2), 3.32 (s, 4H, CH_2), 2.61 (s, 6H, CH_3), 2.48 (s, 6H, CH_3).

^{13}C{^{1}H}-NMR (150.93 MHz, CD_3CN), **^{13}C-DEPT-135**, **HMBC**, **HSQC**

δ = 163.1 (1C, C_q), 158.4 (1C, C_q), 151.8 (1C, C_q), 139.8 (1C, C_q), 136.9 (1C, C_q), 130.1 (1C, CH), 129.6 (2C, CH), 128.8 (3C, CH), 125.4 (1C, CH), 111.6 (1C, CH), 57.6 (1C, CH_2), 48.9 (2C, CH_2), 47.9 (2C, CH_2), 35.1 (2C, CH_3), 32.7 (2C, CH_3).

Mass spectrometry (ESI$^+$ in DCM)

m/z = 392.2558 ([L2a+Bn]$^+$, calc. 392.2563).

UV-vis spectrum (DCM) ($9.81\cdot10^{-5}$ mol L^{-1})

λ (ε) = 361 (11400), 304 (9160), 264 (shoulder, 5400), 235 (5820) nm (M^{-1} cm^{-1}).

CV (DCM, [*n*-Bu_4N][PF_6], 20 mV s^{-1}, referenced to Fc/Fc$^+$)

E_{Ox}^1 = 394 mV. E_{Ox}^2 = 599 mV.

Elemental analysis (C, H, N in %) ($C_{22}H_{30}BrN_7$)

calc.: C 55.93, H 6.40, N 20.75.

found: C 54.91, H 6.17, N 20.78.

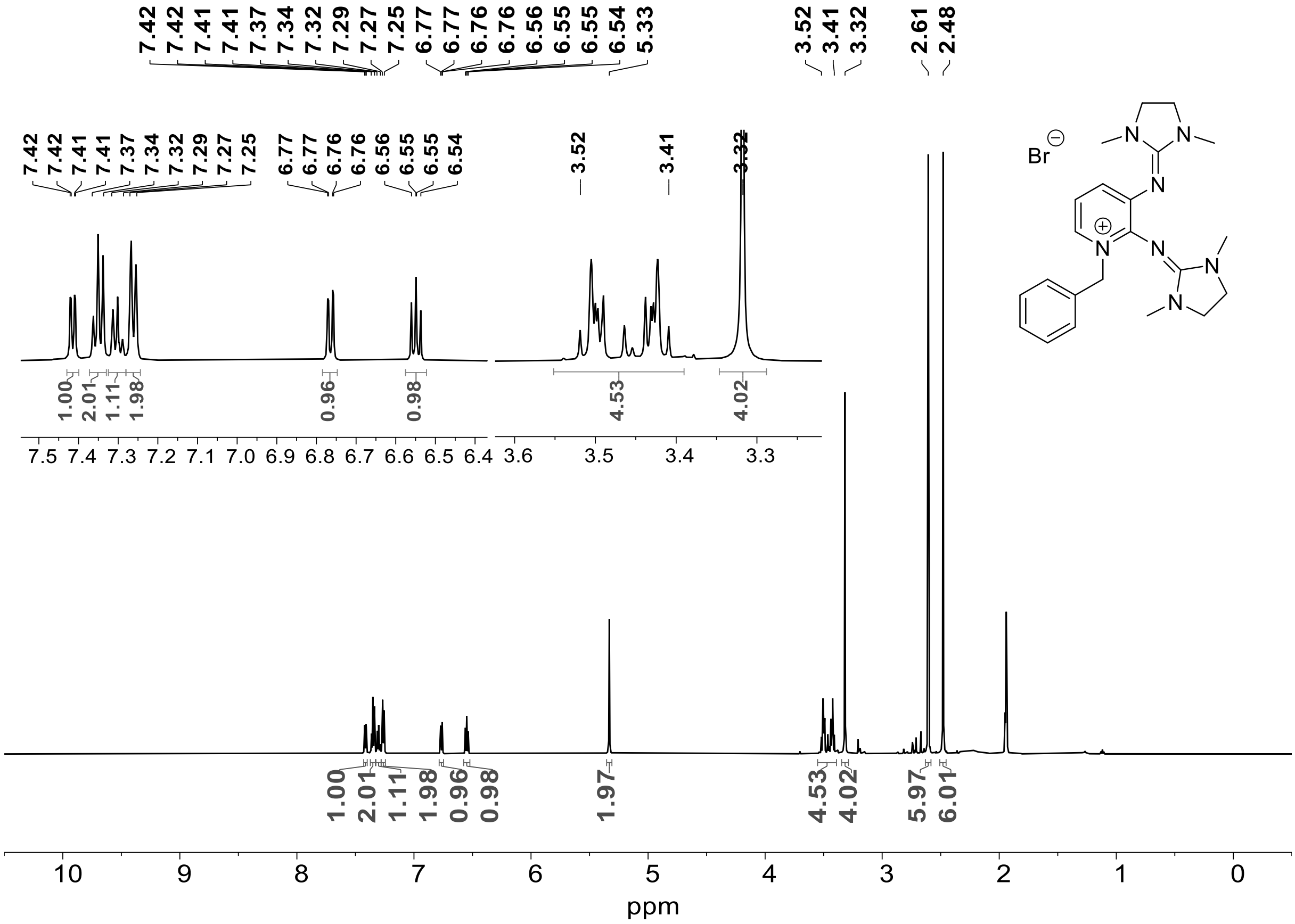

Figure S62: ^{1}H NMR spectrum of [L2a+Bn]Br, measured at 600 MHz in CD_3CN.

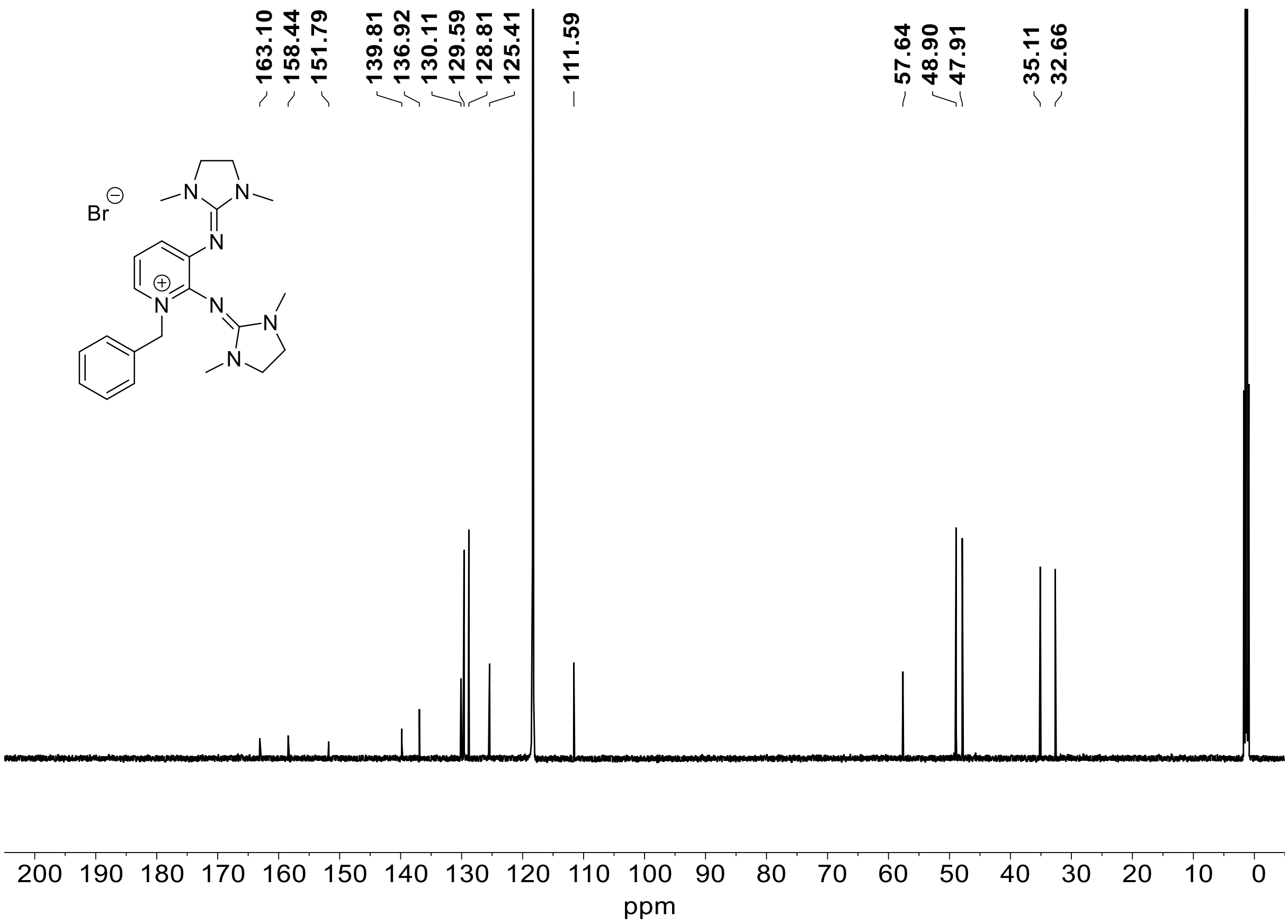

Figure S63: ^{13}C NMR spectrum of [L2a+Bn]Br, measured at 151 MHz in CD_3CN.

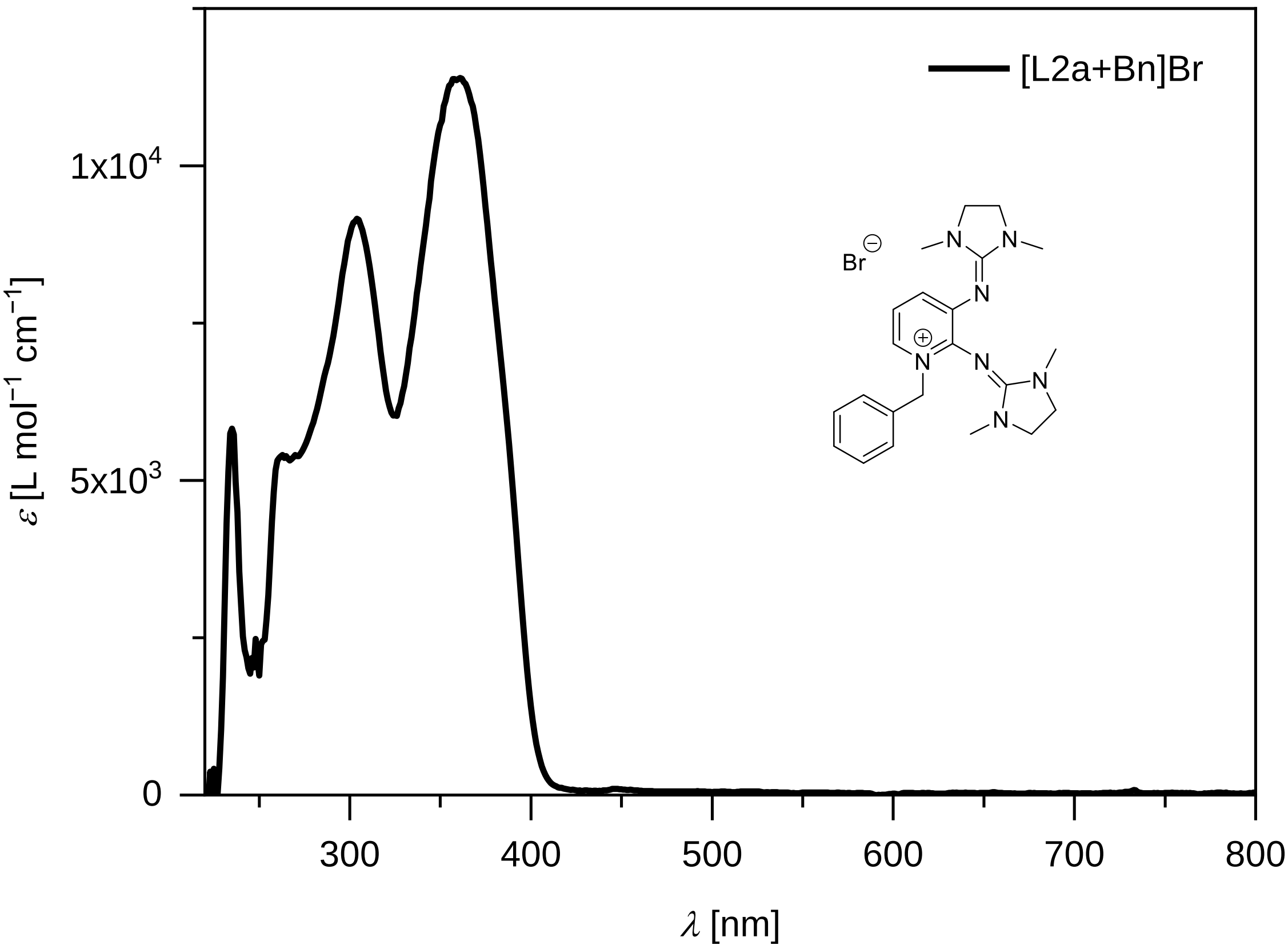

Figure S64: UV-vis absorption spectrum of [L2a+Bn]Br in dichloromethane at a concentration of 9.31 · 10^{-5} mol L^{-1} at rt.

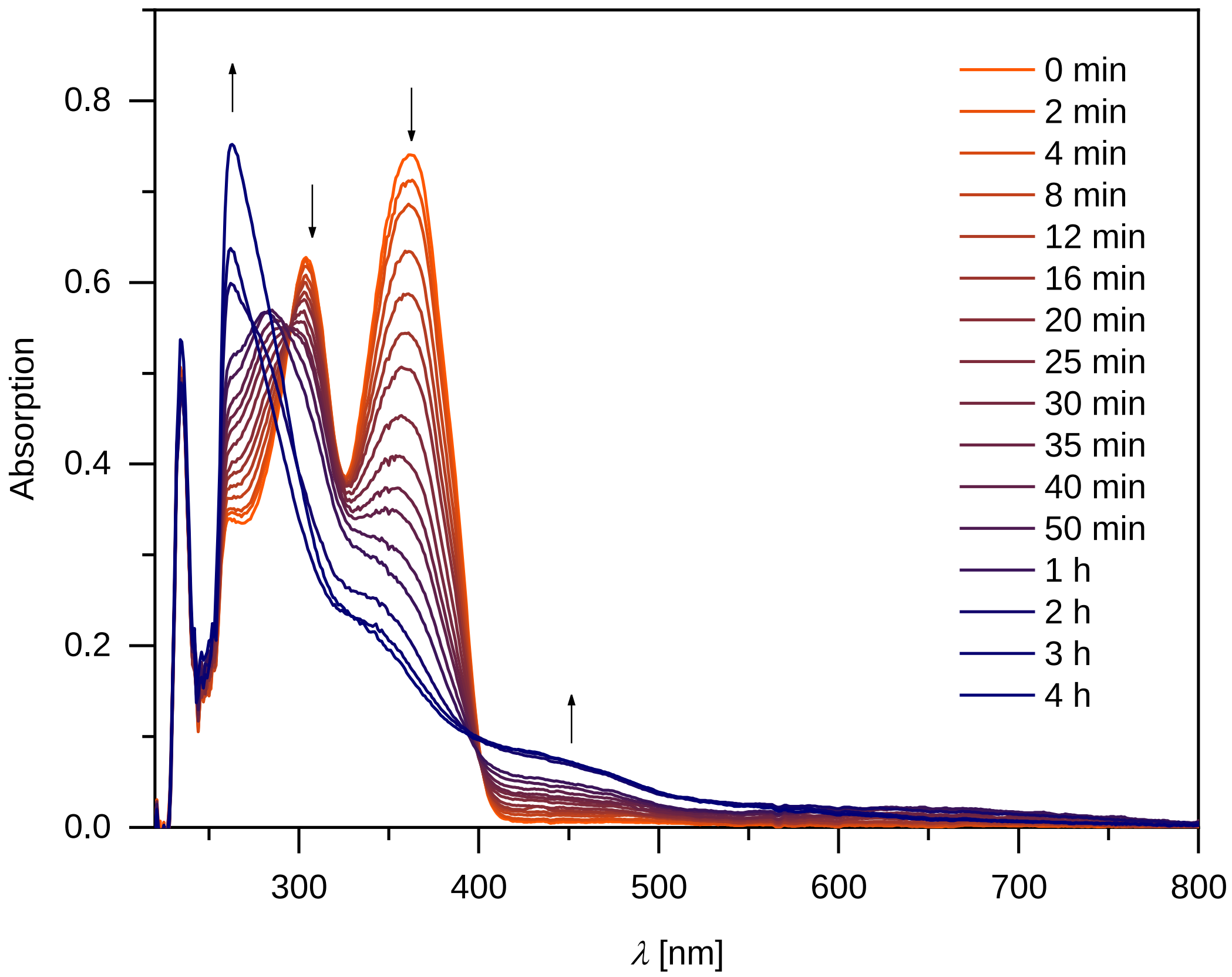

Figure S65: UV-vis absorption spectrum of [L2a+Bn]Br in dichloromethane at a concentration of 9.31 · 10^{-5} mol L^{-1} at rt under irradiation with broad spectrum visible light.

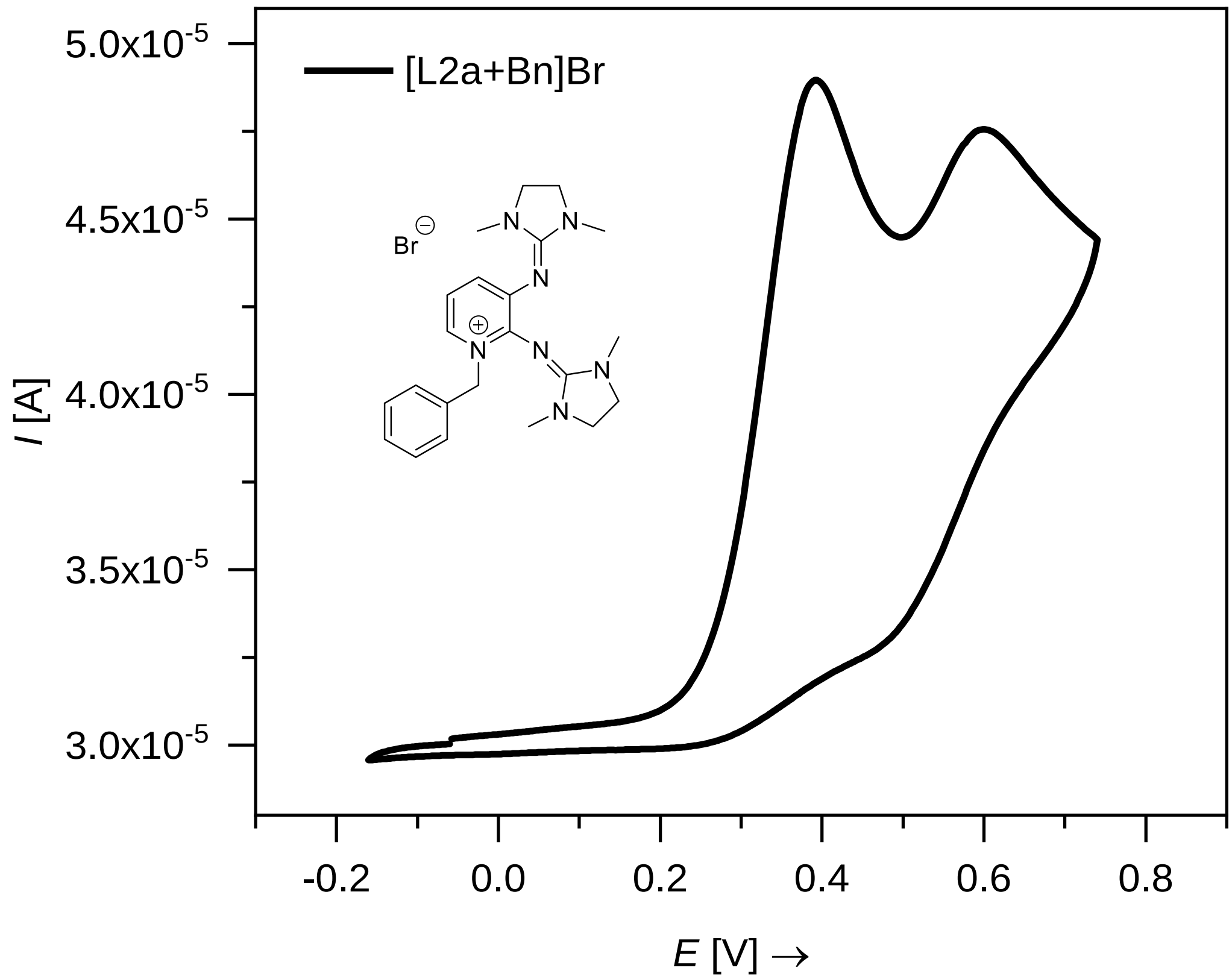

Figure S66: Cyclic voltammogram of [L2a+Bn]Br in dichloromethane ([n-Bu_4N][PF_6], 20 mV s^{-1}, referenced to Fc/Fc^+).

2.3.6 [L2a+Bn(*p*-CF3)]Br

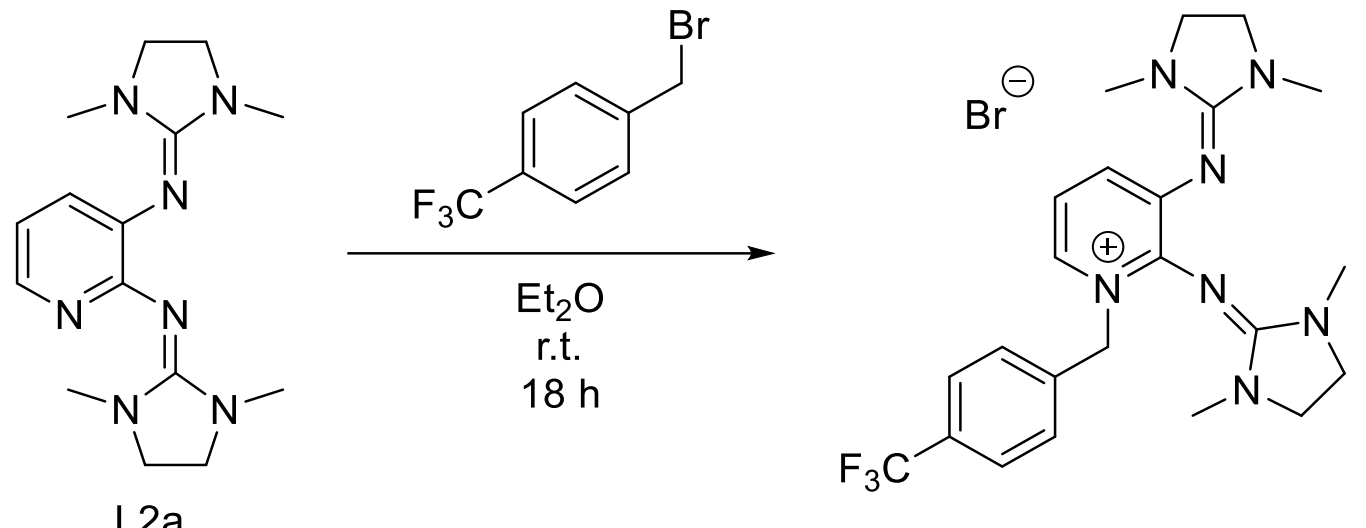

Following general procedure 1, L2a (50.6 mg, 168 μmol, 1.00 eq.) and 4-(trifluoromethyl)benzylbromide (80.2 mg, 336 μmol, 2.00 eq.) were used to obtain [L2a+Bn(*p*-CF_3)]Br (87.7 mg, 162 μmol, 97%) as a colourless solid.

A crystal suitable for single crystal structure analysis was obtained from an acetonitrile solution by layering with diethyl ether.

^{1}H NMR (600.15 MHz, CD_3CN)

δ = 7.67 (d, *J* = 8.2, 2H, CH), 7.41 (d, *J* = 8.3, 2H, CH), 7.37 (dd, *J* = 6.6, 1.5, 1H, CH), 6.76 (dd, *J* = 7.6, 1.5, 1H, CH), 6.58-6.52 (m, 1H, CH), 5.37 (s, 2H, CH_2), 3.52-3.40 (m, 4H, CH_2), 3.32 (s, 4H, CH_2), 2.61 (s, 6H, CH_3), 2.46 (s, 6H, CH_3) ppm.

^{13}C{^{1}H} NMR (150.93 MHz, CD_3CN), **^{13}C-DEPT-135**, **HMBC**, **HSQC**

δ = 163.5 (1C, C_q), 156.6 (1C, C_q), 151.7 (1C, C_q), 141,8 (1C, C_q), 139.7 (1C, CH), 130.0 (1C, CH), 129.3 (2C, CH), 126.5 (2C, CH), 125.2 (1C, CH), 111.5 (1C, CH), 57.3 (1C, CH_2), 48.9 (2C, CH_2), 47.9 (2C, CH_2), 35.2 (2C, CH_3), 32.6 (2C, CH_3) ppm.
Four of the six quaternary C atoms could be detected via the HMBC spectrum, the remaining C atoms are undetectable.

Mass spectrometry (ESI$^+$ in DCM)

m/z = 460.2431 ([L2a+Bn(*p*-CF_3)]$^+$, calc. 460.2432).

UV-vis spectrum (DCM) ($8.90 \cdot 10^{-5}$ mol L^{-1})

λ (ε) = 362 (12200), 305 (9770), 265 (shoulder. 5610), 236 (6430) nm (M^{-1} cm^{-1}).

Elemental analysis (C, H, N in %) ($C_{23}H_{29}BrF_3N_7$)

calc.: C 51.12, H 5.41, N 18.14.

found: C 51.10, H 5.71, N 18.38.

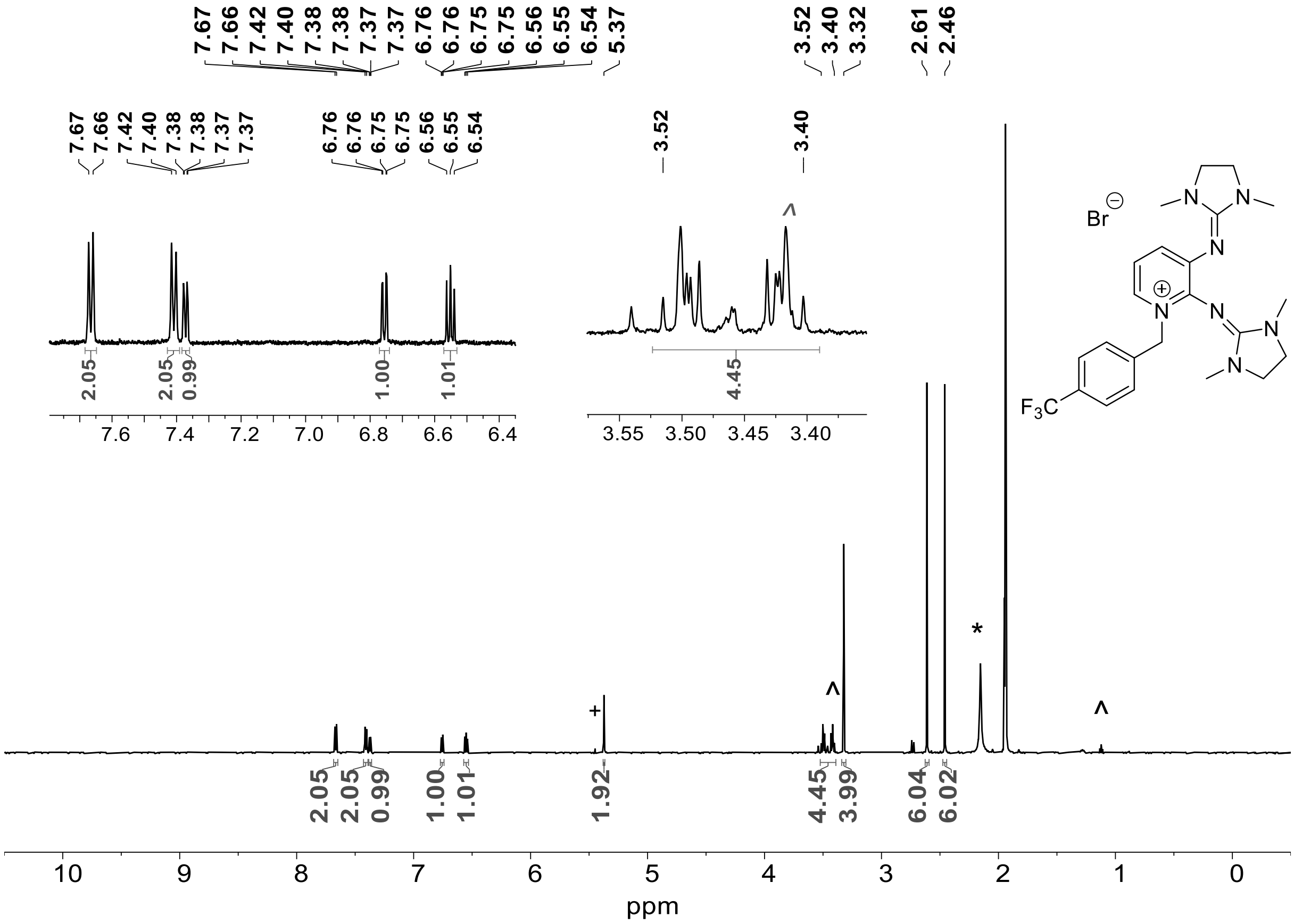

Figure S67: ^{1}H NMR spectrum of [L2a+Bn(p-CF_3)]Br, measured at 600 MHz in CD_3CN, $^{+}$DCM, *water, ^diethyl ether.

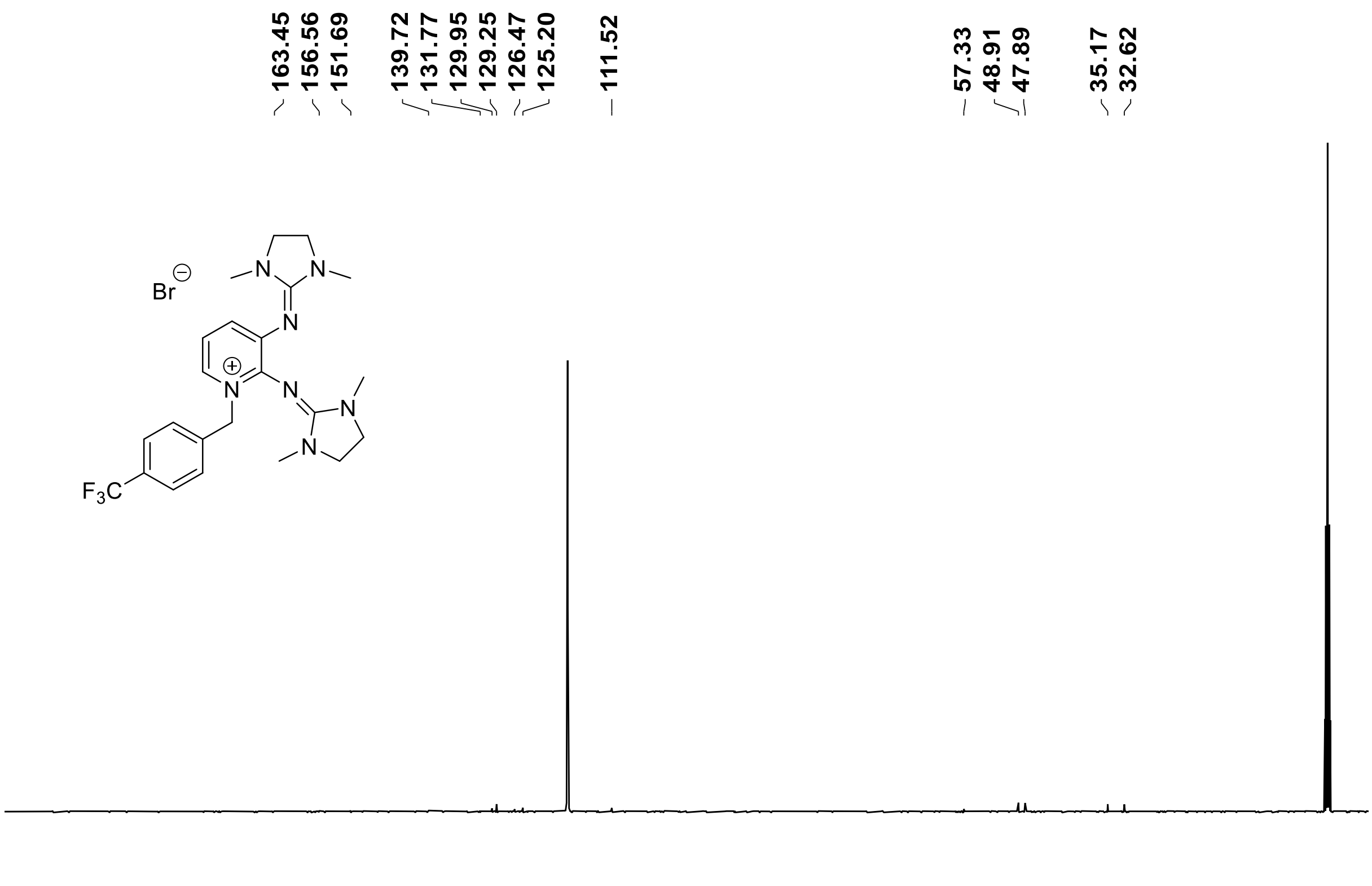

Figure S68: ^{13}C NMR spectrum of [L2a+Bn(p-CF_3)]Br, measured at 151 MHz in CD_3CN.

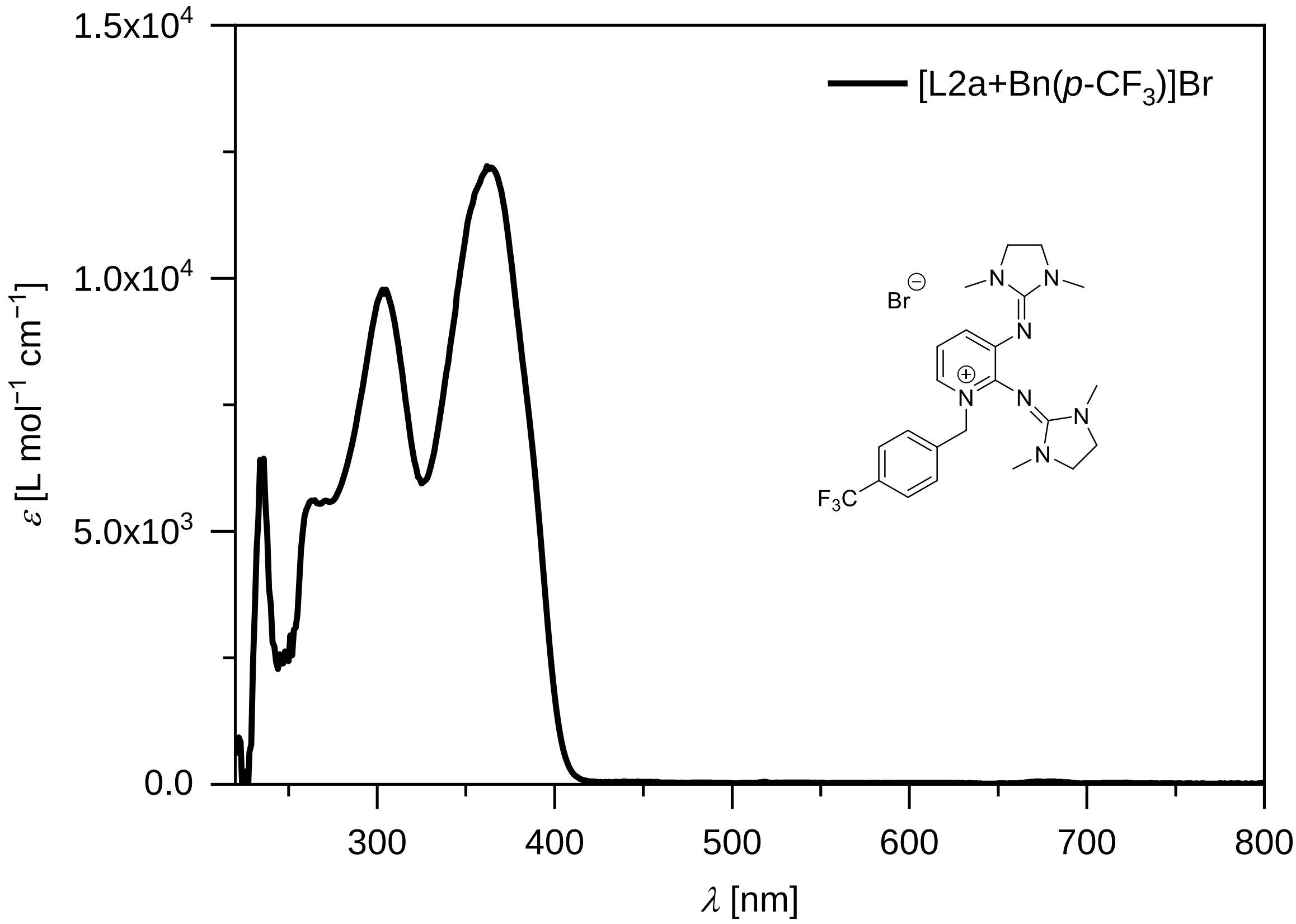

Figure S69: UV-vis absorption spectrum of [L2a+Bn(*p*-CF_3)]Br in dichloromethane at a concentration of $8.90 \cdot 10^{-5}$ mol L^{-1} at rt.

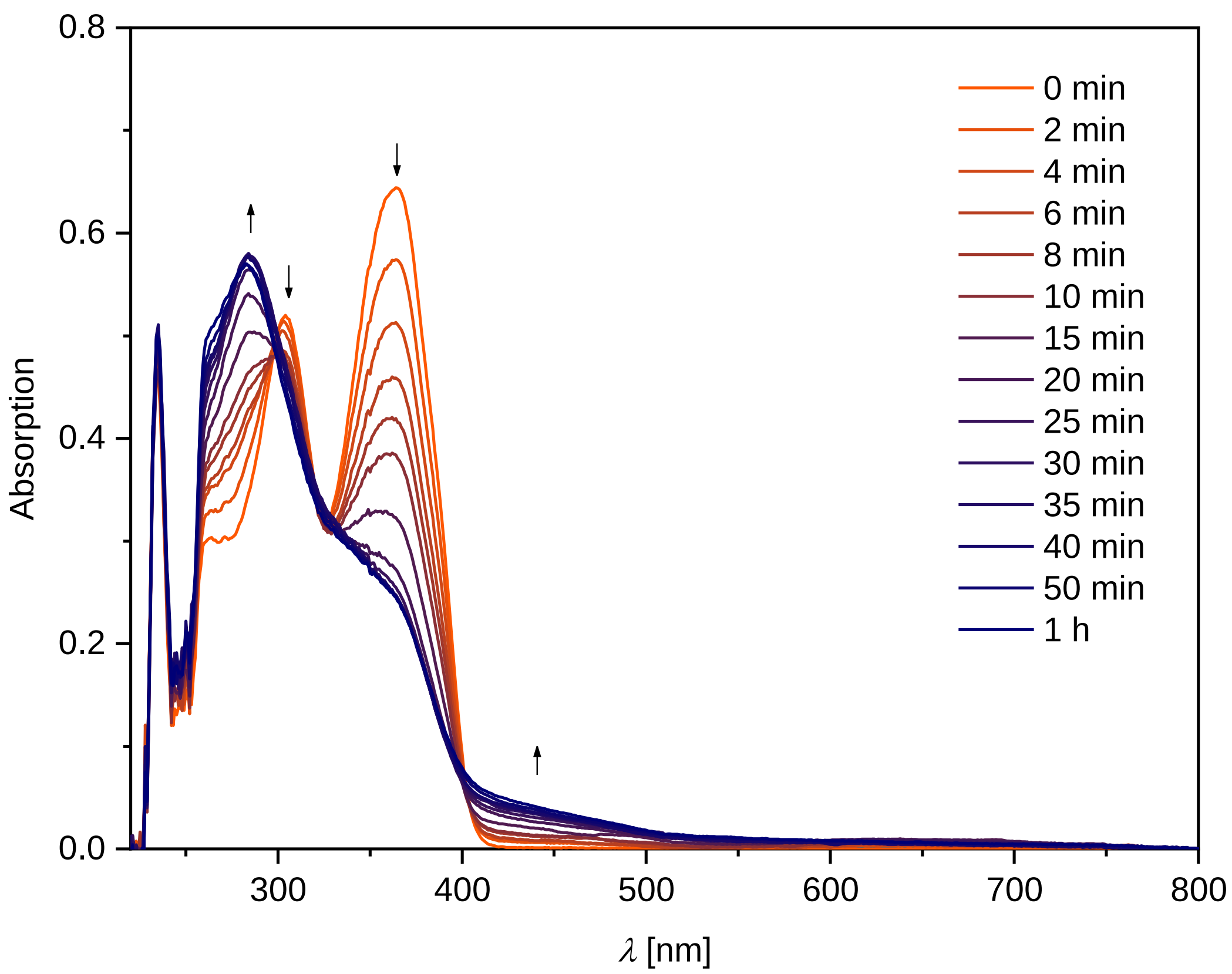

Figure S70: UV-vis absorption spectrum of [L2a+Bn(*p*-CF_3)]Br in dichloromethane at a concentration of $8.90 \cdot 10^{-5}$ mol L^{-1} at rt under irradiation with broad spectrum visible light.

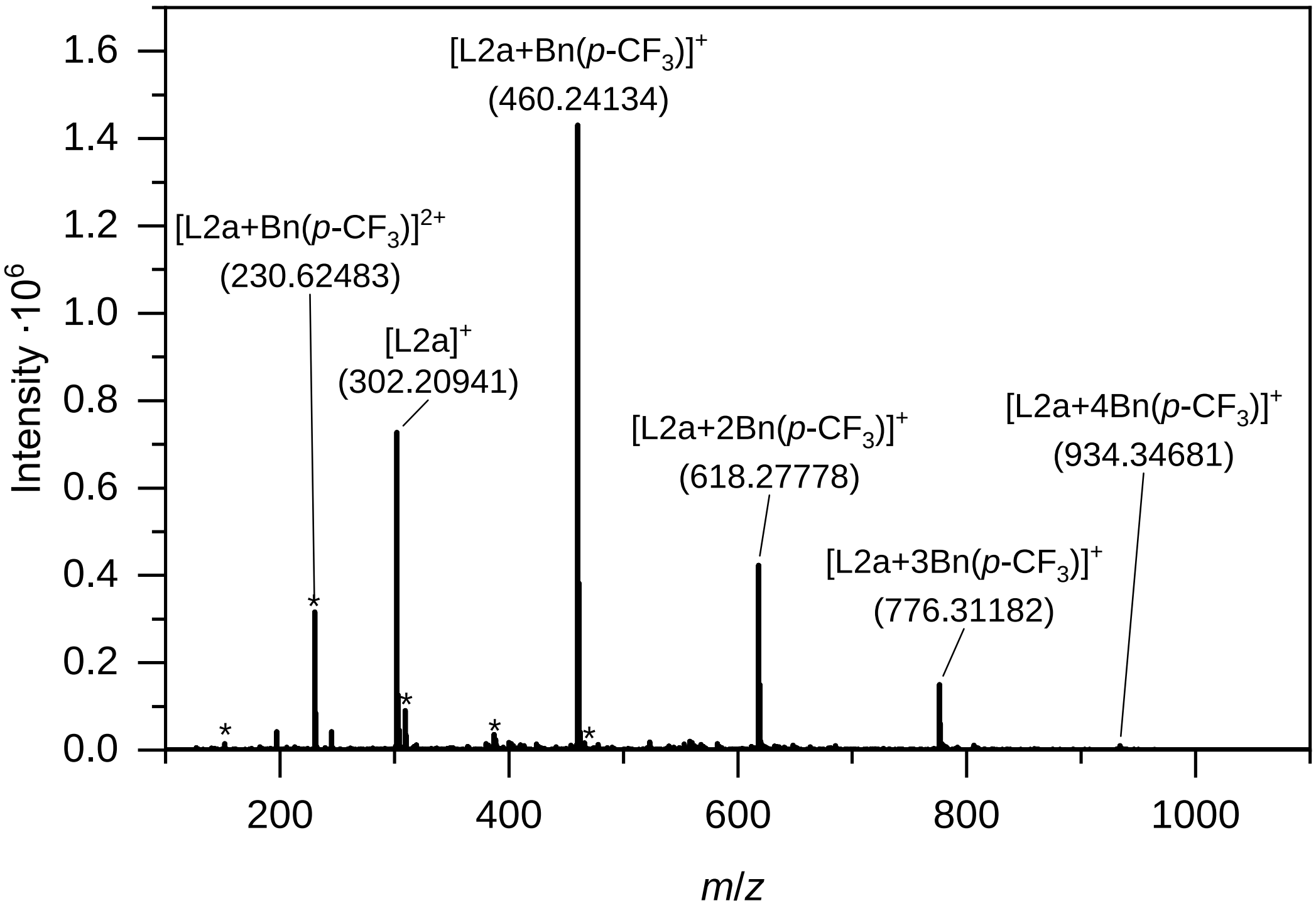

Figure S71: Plot of the mass spectrometry data recorded upon irradiation of [L2a+Bn(*p*-CF_3)$_2$]Br with visible light, *double charged species.

2.3.7 [L2a+Bn(*o*,*p*-CF_3)$_2$]Br

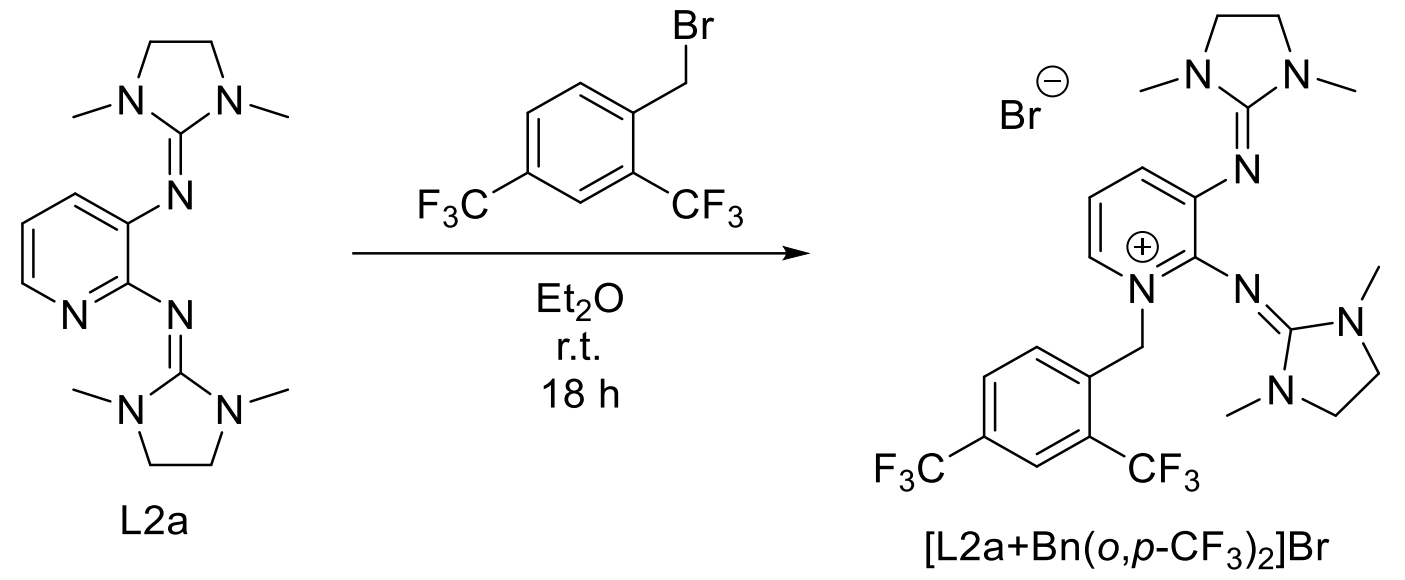

Following general procedure 1, L2a (45.0 mg, 149 µmol, 1.00 eq.) and 1-(bromomethyl)-2,4-bis(trifluoromethyl)benzol (56.0 µL, 91.7mg, 336 µmol, 2.00 eq.) were used to obtain [L2a+Bn(*o*,*p*-CF_3)$_2$]Br (88.1mg, 145 µmol, 97%) as a colourless solid.

A crystal suitable for single crystal structure analysis was obtained from an acetonitrile solution by layering with diethyl ether.

^{1}H NMR (600 MHz, CD_3CN)

δ = 8.07 (s, 1H, CH), 7.86 (d, J = 8.2 Hz, 1H, CH), 7.30 (dd, J = 6.7, 1.5 Hz, 1H, CH), 7.13 (d, J = 8.6 Hz, 1H, CH), 6.78 (dd, J = 7.5, 1.5 Hz, 1H, CH), 6.58 (dd, J = 7.6, 6.6 Hz, 1H, CH), 5.55 (s, 2H, CH_2), 3.49-3.39 (m, 4H, CH_2), 3.35 (s, 4H, CH_2), 2.65 (s, 6H, CH_3), 2.45 (s, 6H, CH_3) ppm.

^{13}C{^{1}H} NMR (151 MHz, CD_3CN)

δ = 163.9 (1C, C_q), 159.0 (1C, C_q), 151.6 (1C, C_q), 140.6 (1C, C_q), 139.6 (1C, C_q), 130.5 (1C, CH), 129.9 (1C, CH), 129.0 (1C, CH), 124.9 (1C, CH), 124.7 (1C, CH), 111.4 (1C, CH), 54.7 (1C, CH_2), 48.9 (2C, CH_2), 47.9 (2C, CH_2), 35.3 (2C, CH_3), 32.6 (2C, CH_3) ppm.

Two of the seven quaternary C atom signals are coincident or not observed.

^{19}F{^{1}H} NMR (565 MHz, CD_3CN)

δ = −61.60 (3F, CF_3), −63.37 (3F, CF_3) ppm.

Mass spectrometry (ESI$^+$ in DCM)

m/z = 528.2295 ([L2a+Bn$(CF_3)_2$]$^+$, calc. 528.2305),

m/z = 264.6184 ([L2a+Bn$(CF_3)_2$H]$^{2+}$, calc. 264.6189).

UV-vis (DCM) ($7.80 \cdot 10^{-5}$ mol L^{-1})

λ (ε) = 366 (14300), 303 (11000), 273 (6810), 266 (6710), 235 (7650) nm (M^{-1} cm^{-1}).

Elemental analysis (C, H, N in %) ($C_{24}H_{28}BrF_6N_7$)

calc.: C 47.38, H 4.64, N 16.12.

found: C 47.46, H 4.94, N 15.94.

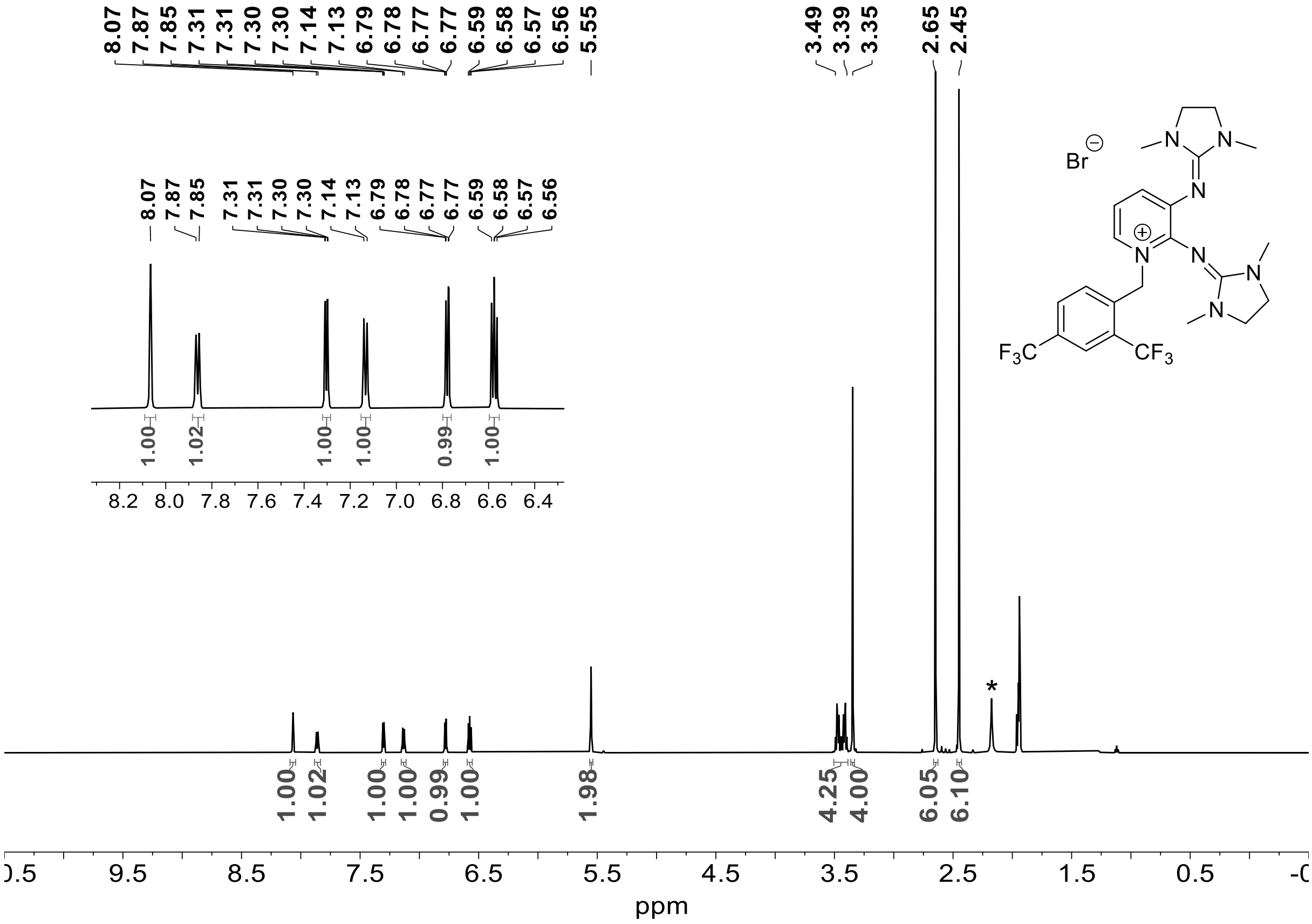

Figure S72: ^{1}H NMR spectrum of [L2a+Bn(*o*,*p*-CF_3)$_2$]Br, measured at 600 MHz in CD_3CN, *water.

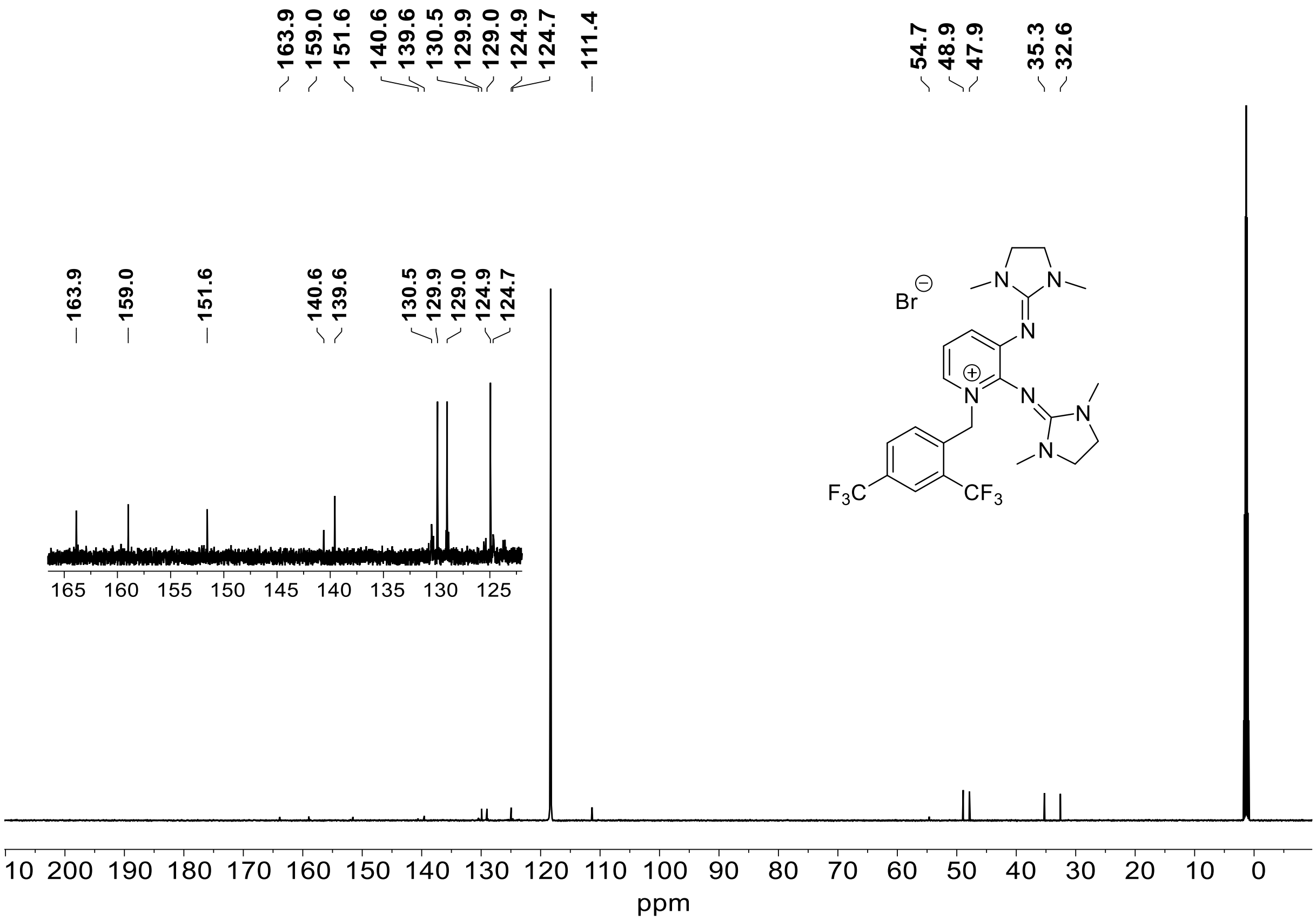

Figure S73: ^{13}C NMR spectrum of [L2a+Bn(*o*,*p*-CF_3)$_2$]Br, measured at 151 MHz in CD_3CN.

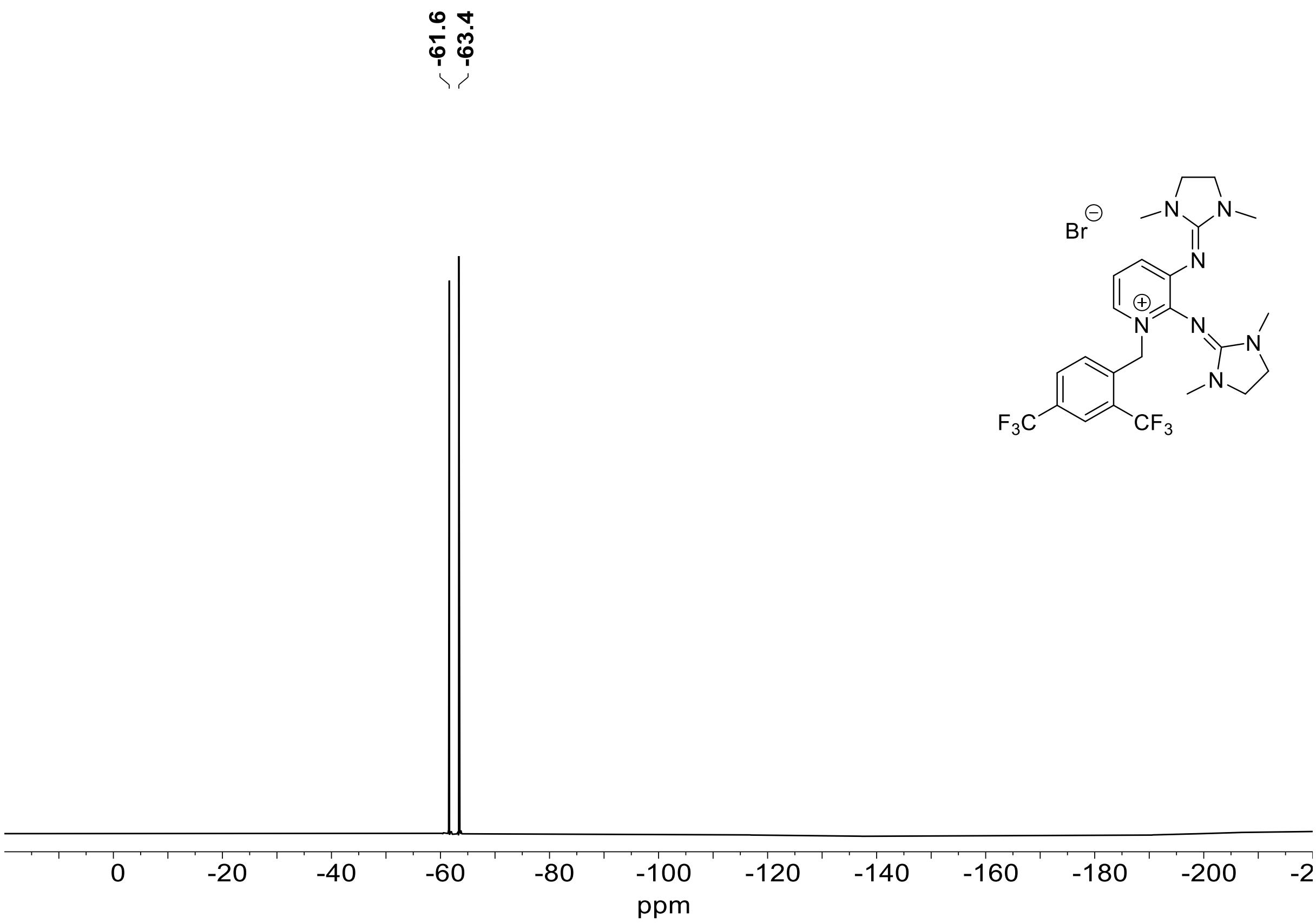

Figure S74: ^{19}F NMR spectrum of [L2a+Bn(o,p-CF_3)$_2$]Br, measured at 565 MHz in CD_3CN.

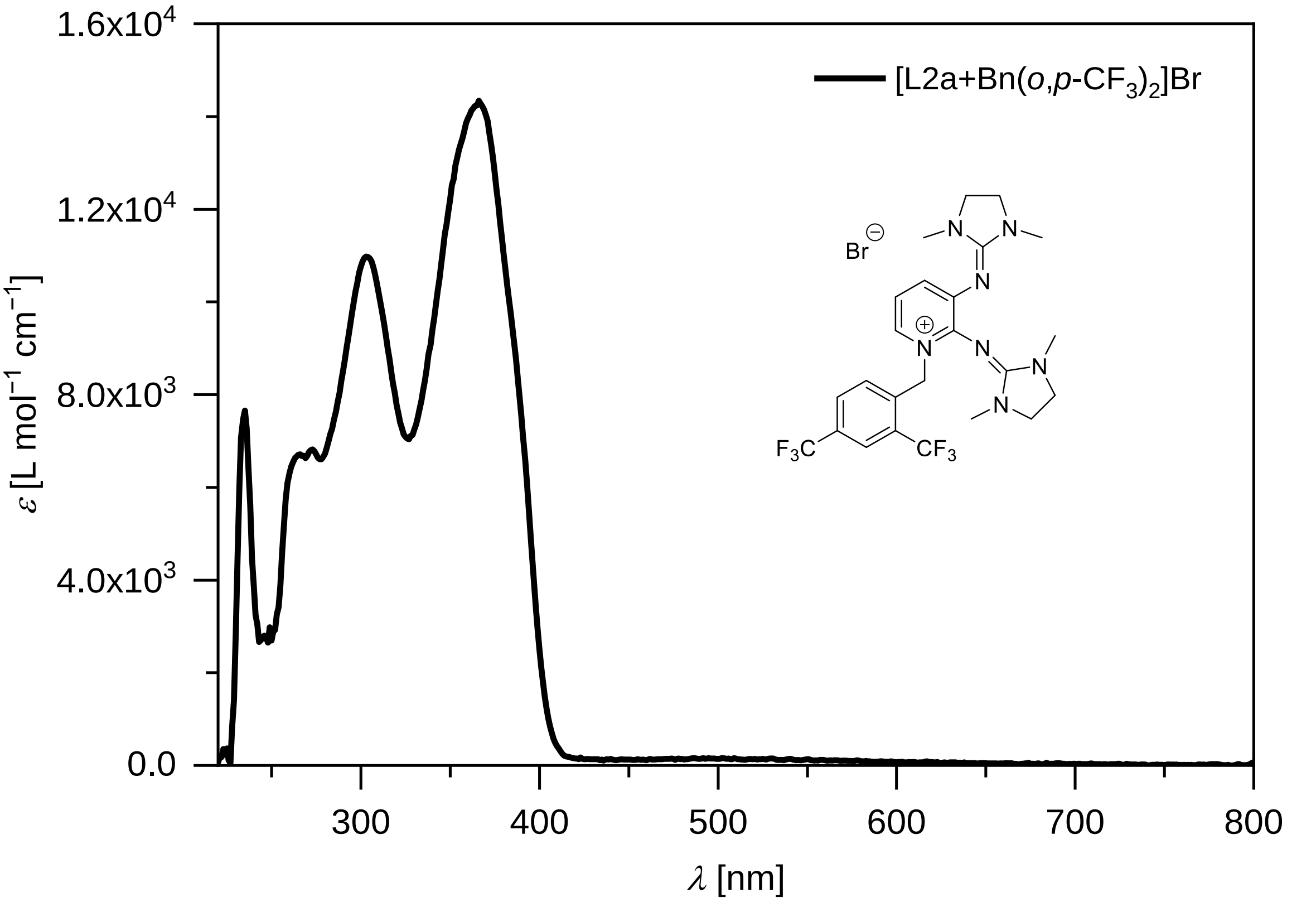

Figure S75: UV-vis absorption spectrum of [L2a+Bn(o,p-CF_3)$_2$]Br in dichloromethane at a concentration of $7.80 \cdot 10^{-5}$ mol L^{-1} at rt.

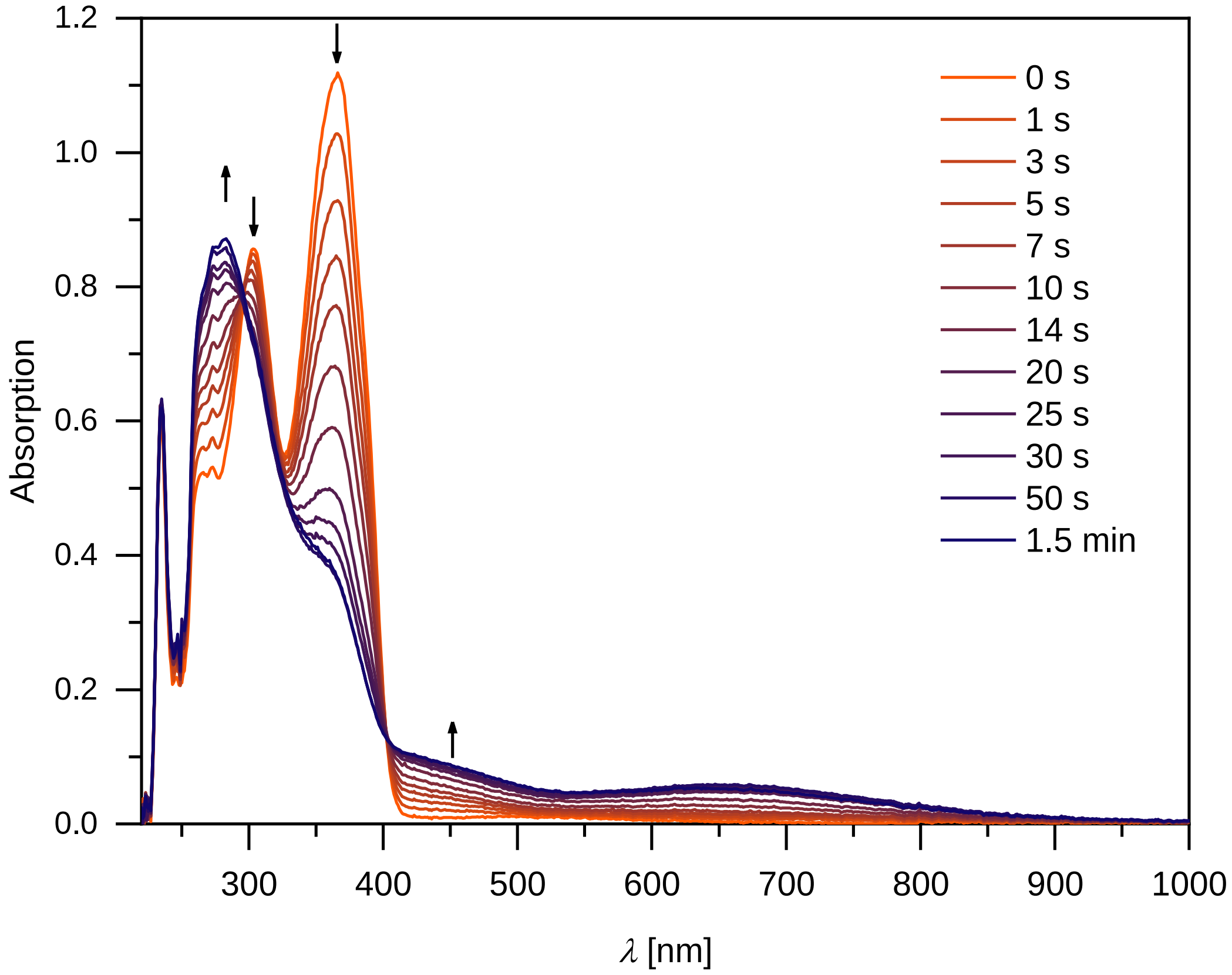

Figure S76: UV-vis absorption spectrum of [L2a+Bn(*o*,*p*-CF_3)$_2$]Br in dichloromethane at a concentration of $7.80\cdot10^{-5}$ mol L^{-1} at r.t. under irradiation with broad spectrum visible light.

2.3.8 [L2a+Bn(*o*-CF_3)]Br

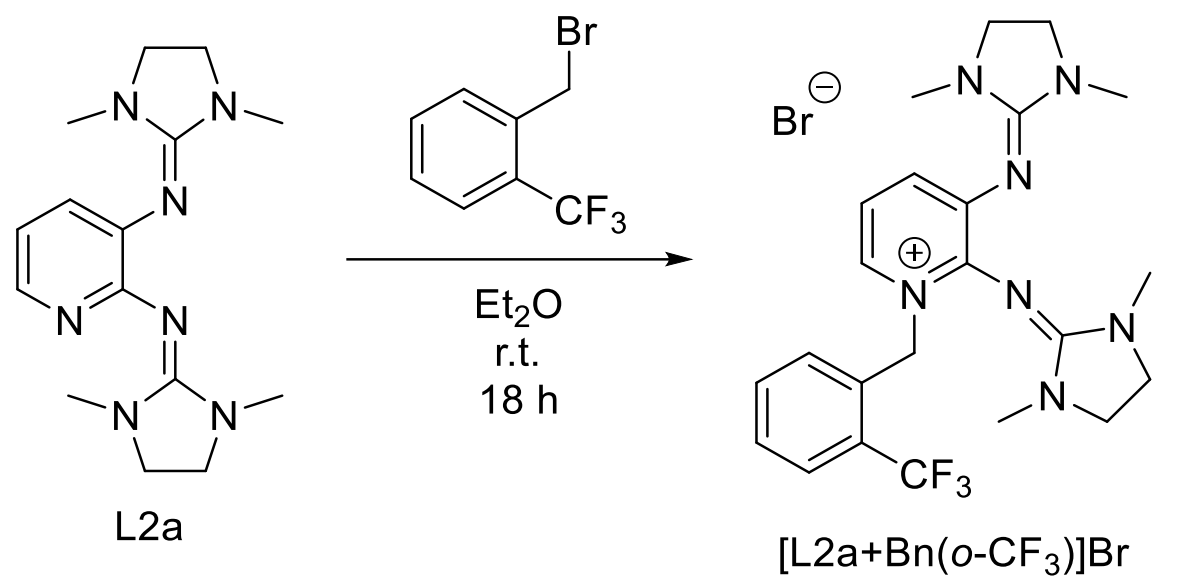

Following general procedure 1, L2a (57.0 mg, 189 µmol, 1.00 eq.) and 1-(bromomethyl)-2-(trifluoromethyl)benzene (57.6 µL, 90.4 mg, 378 µmol, 2.00 eq.) were used to obtain [L2a+Bn(*o*-CF_3)]Br (100 mg, 185 µmol, 98%) as a colourless solid.

A crystal suitable for single crystal structure analysis was obtained from an acetonitrile solution by layering with diethyl ether.

^{1}H NMR (600 MHz, CD_3CN)

δ = 7.78 (d, *J* = 8.3 Hz, 1H, CH), 7.56 (t, *J* = 7.5 Hz, 1H, CH), 7.48 (t, *J* 7.5 Hz, 1H, CH), 7.26 (dd, *J* = 6.6, 1.5 Hz, 1H, CH), 6.95 (d, *J* = 7.9 Hz, 1H, CH), 6.78 (dd, *J* = 7.5, 1.5 Hz, 1H, CH), 6.56 (dd, *J* = 7.5, 6.6 Hz, 1H, CH), 5.51 (s, 2H, CH_2), 3.49-3.40 (m, 4H, CH_2), 3.34 (s, 4H, CH_2), 2.65 (s, 6H, CH_3), 2.48 (s, 6H, CH_3) ppm.

^{13}C{^{1}H} NMR (150 MHz, CD_3CN)

δ = 163.7 (1C, C_q), 158.8 (1C, C_q), 151.7(1C, C_q), 139.6(1C, C_q), 135.5 (1C, C_q), 133.7 (1C, CH), 130.0 (1C, CH), 128.9 (1C, CH), 128.0 (1C, CH), 127.4 (1C, CH), 125.1 (1C, CH), 111.3 (1C, CH), 54.6 (1C, CH_2), 48.9 (2C, CH_2), 47.9 (2C, CH_2), 35.2 (2C, CH_3), 32.6 (2C, CH_3) ppm.

One of the seven quaternary C atom signals is coincident or not observed.

^{19}F{^{1}H} NMR (565 MHz, CD_3CN)

δ = −61.1 (3F, CF_3) ppm.

Mass spectrometry (ESI^+ in DCM)

m/z = 460.2430 ([L2a+Bn(*o*-CF_3)]$^+$, calc. 460.2437),

m/z = 230.6259 ([L2a+Bn(*o*-CF_3)H]$^{2+}$, calc. 230.6252),

UV-vis (DCM) ($5.43\cdot10^{-5}$ mol L^{-1})

λ (ε) = 363 (11900), 305 (9150), 272 (5800), 265 (5790), 235 (8690) nm (M^{-1} cm^{-1}).

Elemental analysis (C, H, N in %) ($C_{23}H_{29}BrF_3N_7$)

calc.: C 51.12, H 5.41, N 18.14.

found: C 51.71, H 5.76, N 18.28.

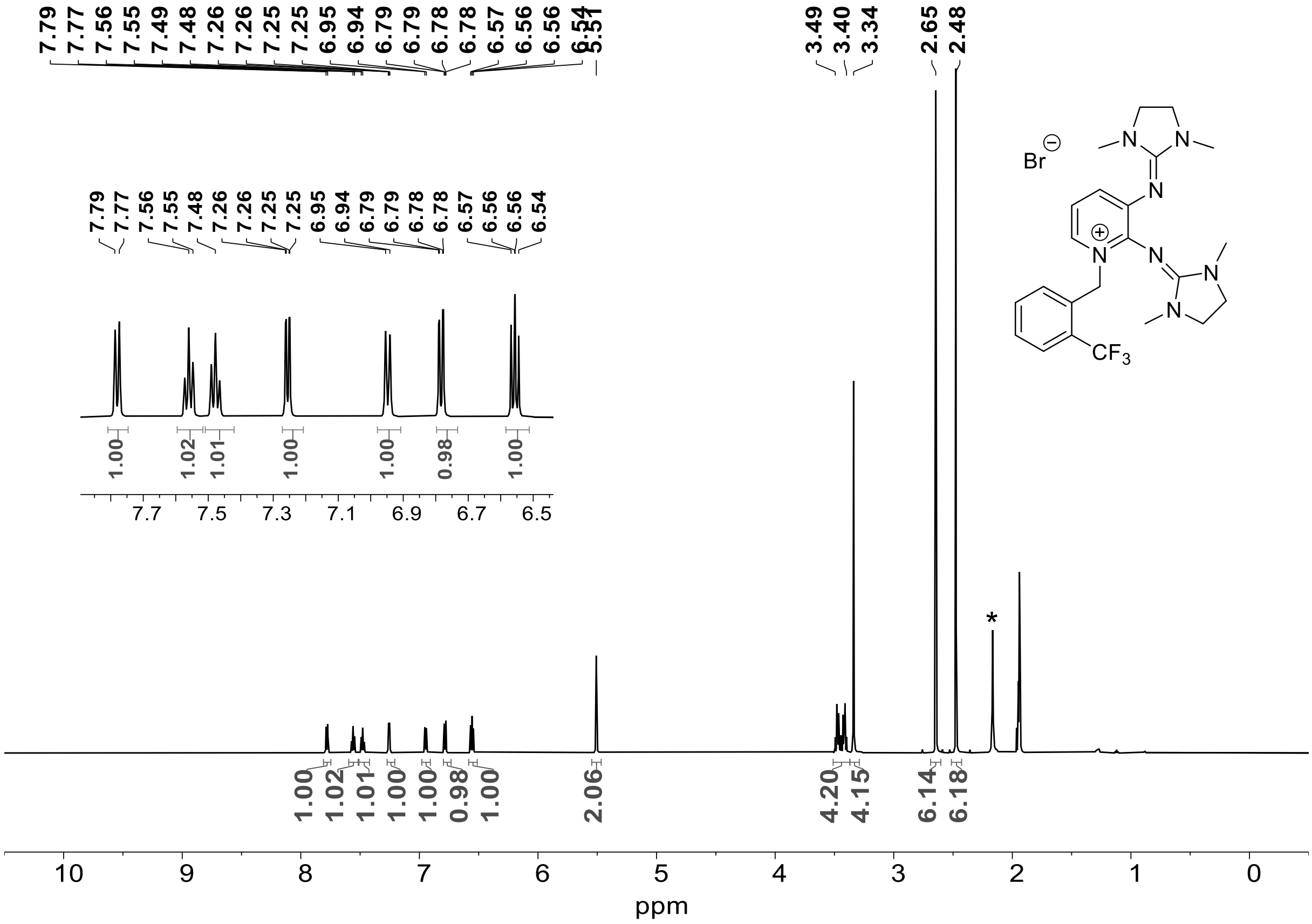

Figure S77: ^{1}H NMR spectrum of [L2a+Bn(*o*-CF$_3$)]Br, measured at 600 MHz in CD$_3$CN, *water.

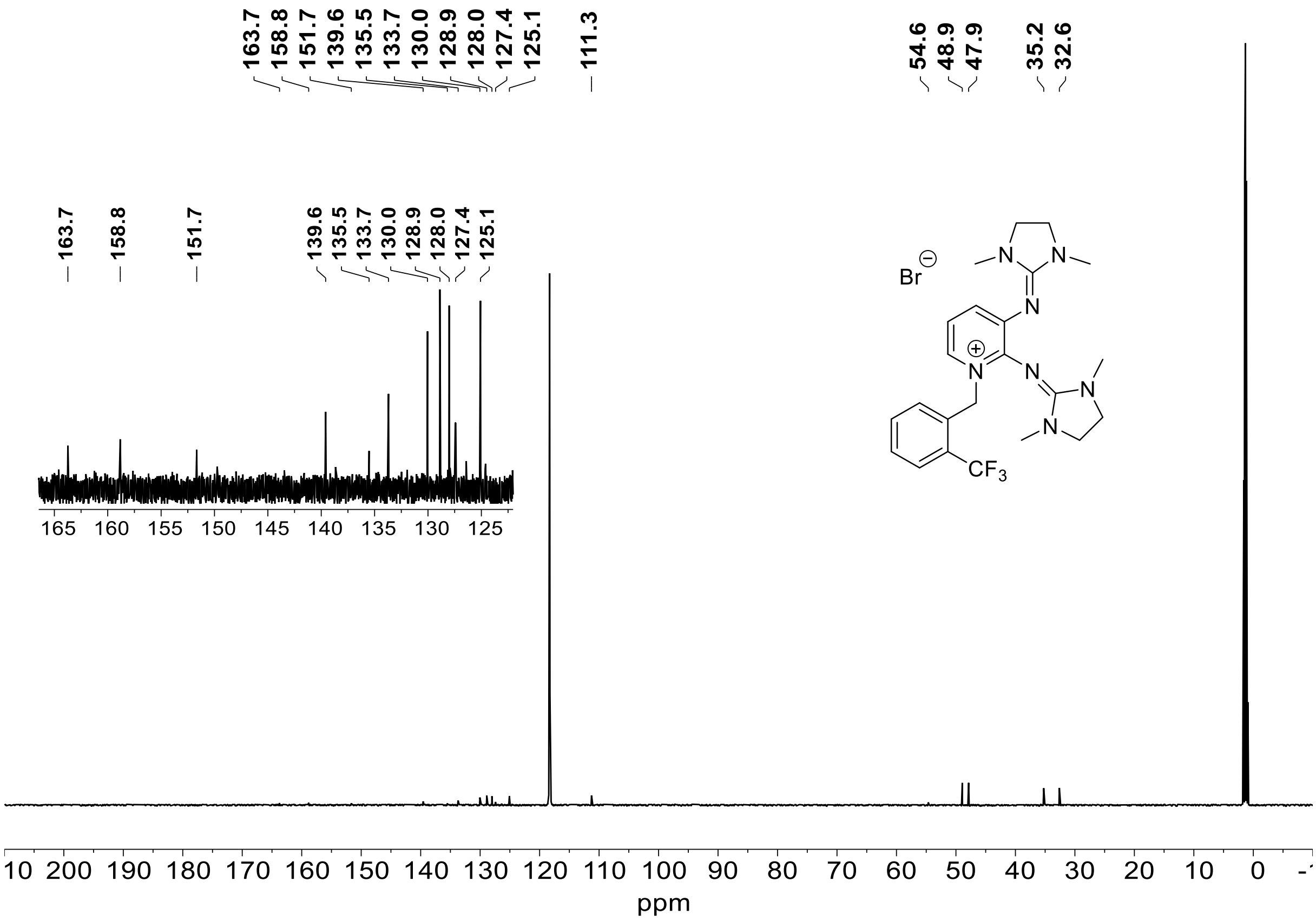

Figure S78: ^{13}C NMR spectrum of [L2a+Bn(*o*-CF$_3$)]Br, measured at 151 MHz in CD$_3$CN.

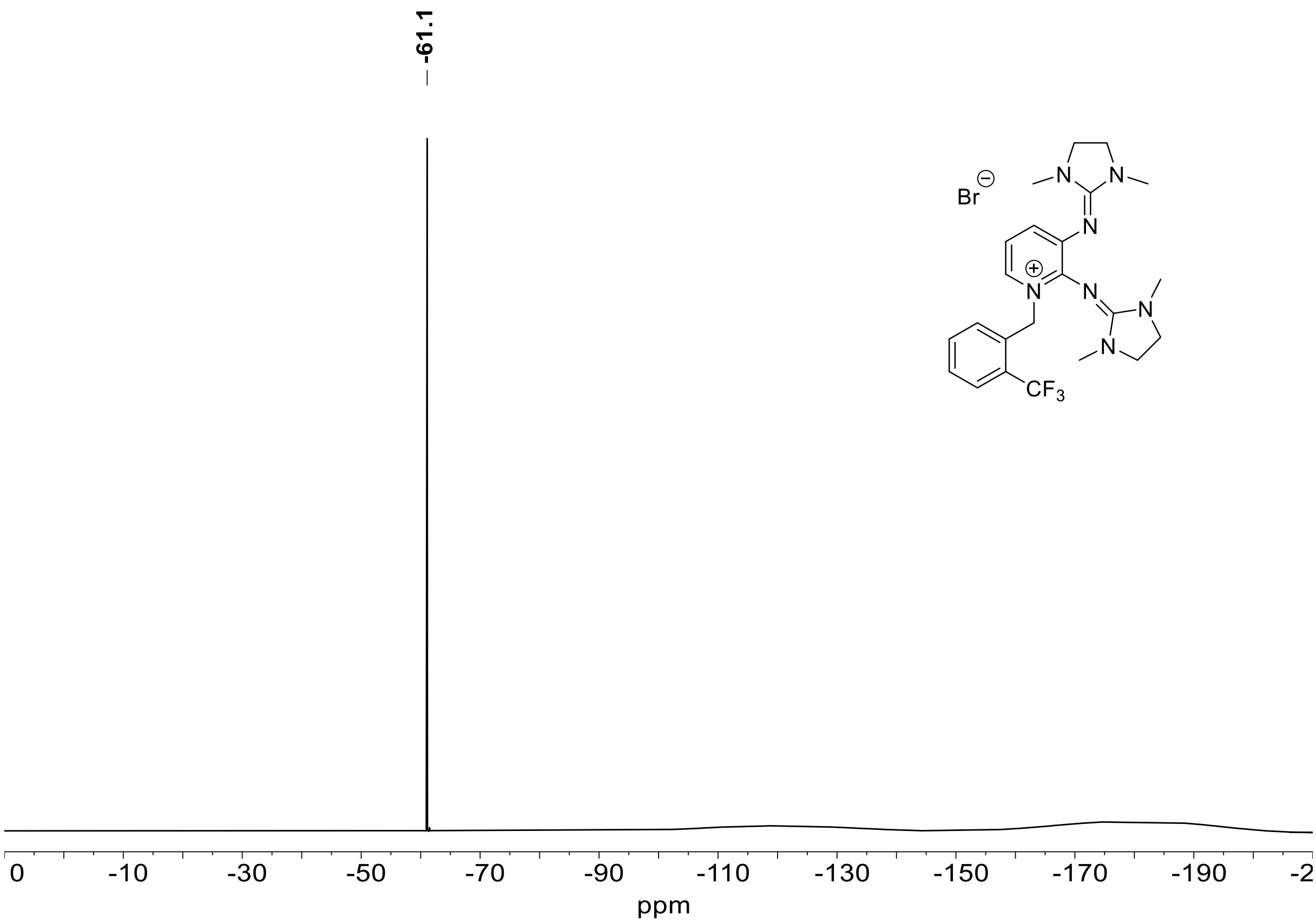

Figure S79: ^{19}F NMR spectrum of [L2a+Bn(*o*-CF_3)]Br, measured at 565 MHz in CD_3CN.

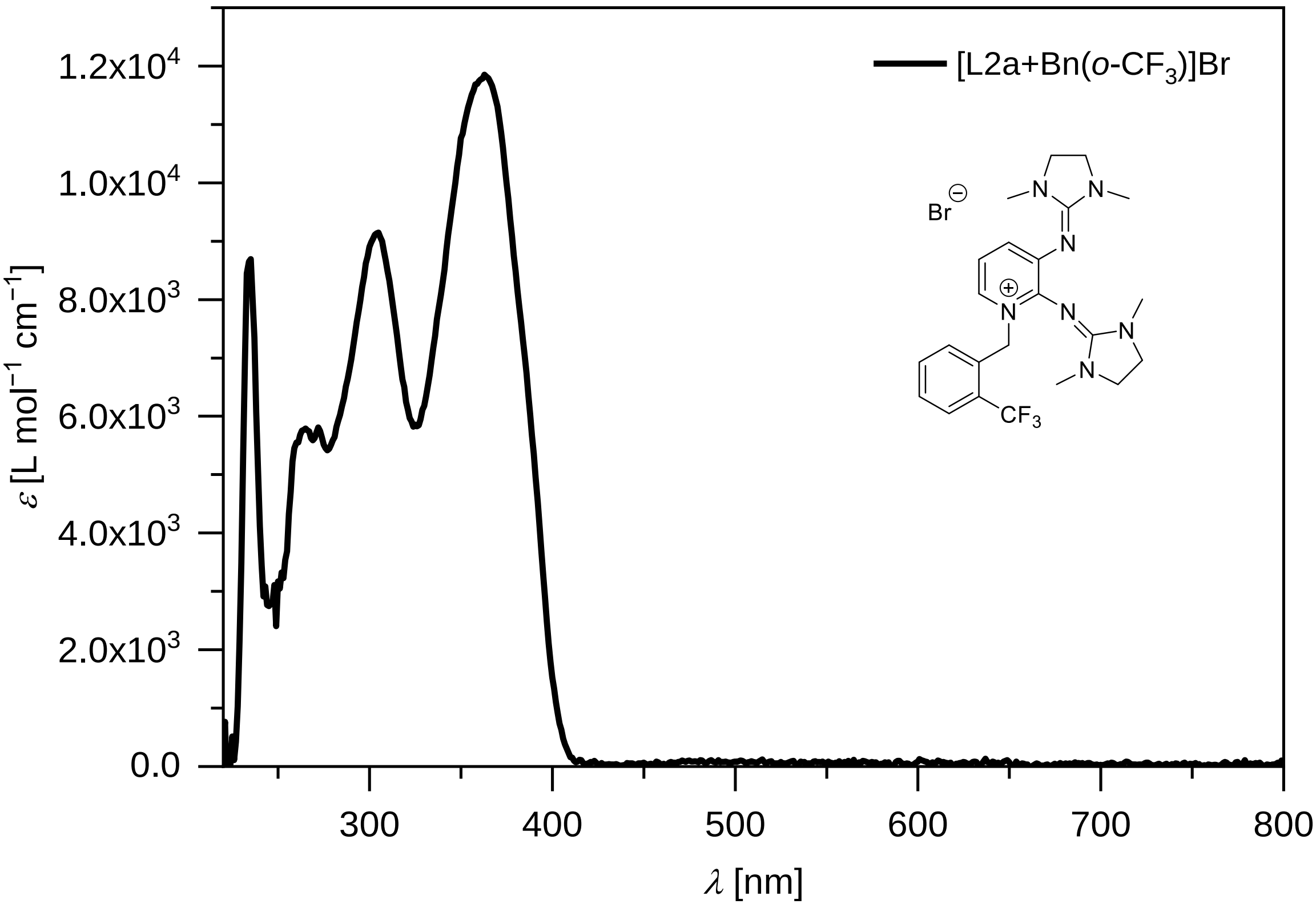

Figure S80: UV-vis absorption spectrum of [L2a+Bn(*o*-CF_3)]Br in dichloromethane at a concentration of $5.42 \cdot 10^{-5}$ mol L^{-1} at r.t.

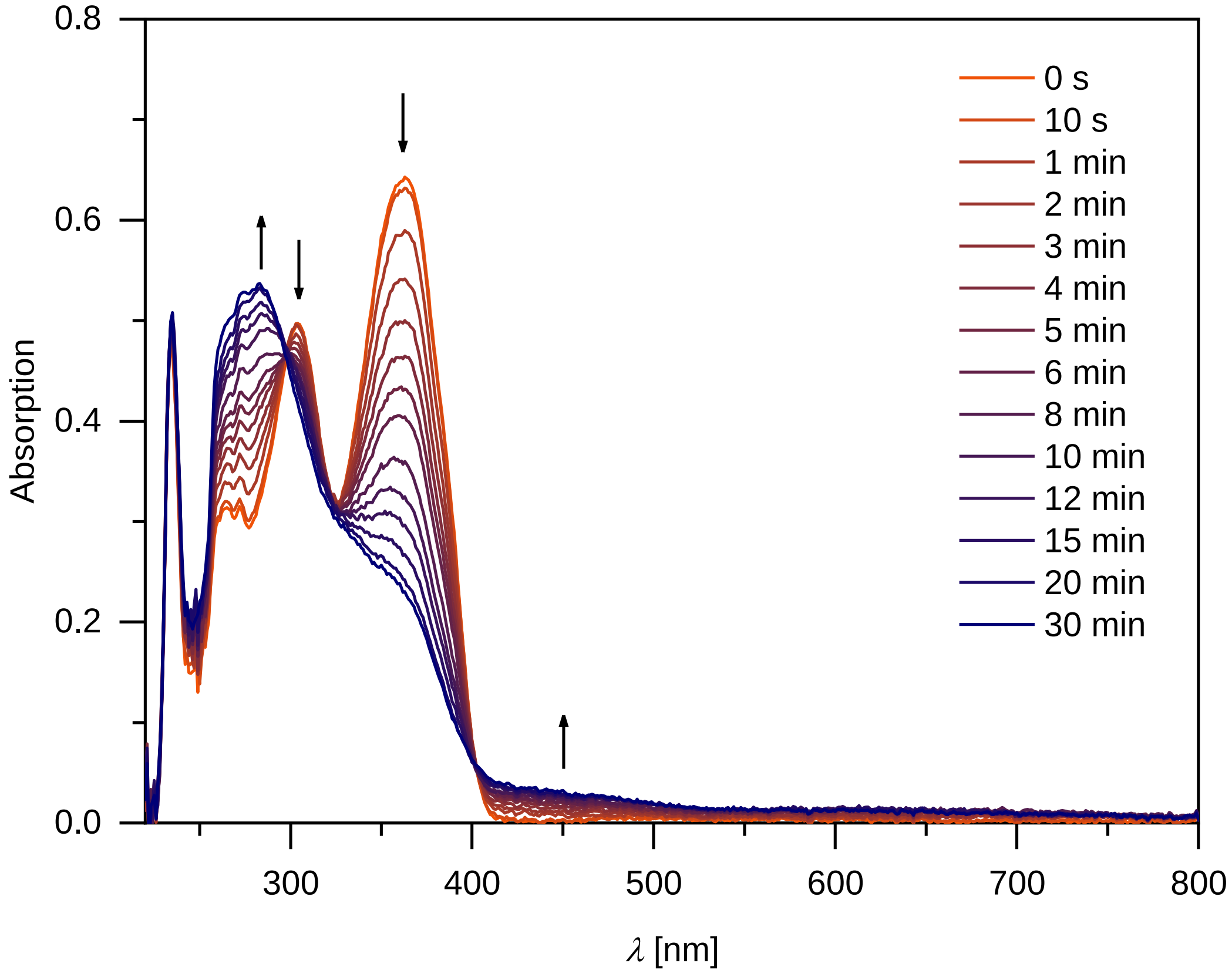

Figure S81: UV-vis absorption spectrum of [L2a+Bn(*o*-CF_3)]Br in dichloromethane at a concentration of $5.42 \cdot 10^{-5}$ mol L^{-1} at r.t. under irradiation with broad spectrum visible light.

2.3.9 [L2a+Bn(F)$_5$]Br

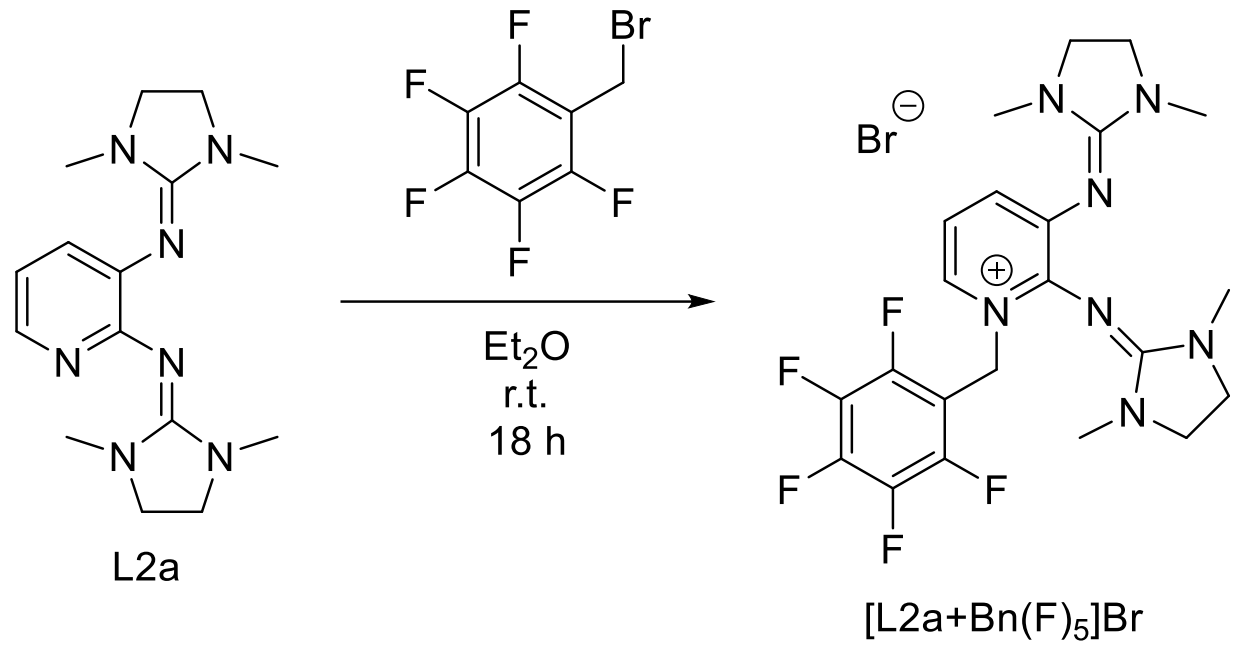

Following general procedure 1, L2a (42.0 mg, 139 µmol, 1.00 eq.) and 1-(bromomethyl)-2,3,4,5,6-pentafluorobenzene (42.1 µL, 72.7 mg, 279 µmol, 2.00 eq.) were used to obtain [L2a(F)$_5$]Br (71.1 mg, 126.4 µmol, 91%) as a colourless solid.

^{1}H NMR (600 MHz, CD_3CN)

δ = 7.43-7.41 (m, 1H, CH), 6.71 (dd, J = 7.5, 1.4 Hz, 1H, CH), 6.50 (dd, J = 7.5, 6.7 Hz, 1H, CH), 5.35 (s, 2H, CH_2), 3.56-3.43 (m, 4H, CH_2), 3.32 (s, 4H, CH_2), 2.59 (s, 6H, CH_3), 2.51 (s, 6H, CH_3) ppm.

^{13}C{^{1}H} NMR (151 MHz, CD_3CN)

δ = 163.4 (1C, C_q), 158.7(1C, C_q), 151.7(1C, C_q), 139.3(1C, C_q), 130.3 (1C, CH), 125.1 (1C, CH), 111.0 (1C, CH), 48.9 (2C, CH_2), 47.9 (2C, CH_2), 47.4 (1C, CH_2), 35.1 (2C, CH_3), 32.6 (2C, CH_3) ppm.

Four of the ten quaternary C atom signals are coincident or not observed.

^{19}F{^{1}H} NMR (565 MHz, CD_3CN)

δ = −142.07 (dd, J = 21.6, 7.7 Hz, 2F, CF), −155.92 (t, J = 20.1 Hz, 1F, CF), −164.43-−164.51 (m, 2F, CF) ppm.

Mass spectrometry (ESI^+ in DCM)

m/z = 482.2085 ($[L2a+Bn(F)_5]^+$, calc. 482.2086),

m/z = 241.6085 ($[L2a+Bn(F)_5H]^{2+}$, calc. 241.6080).

UV-vis (DCM) ($8.29\cdot10^{-5}$ mol L^{-1})

λ (ε) = 365 (14100), 302 (10900), 266 (6780), 235 (7450) nm (M^{-1} cm^{-1}).

Elemental analysis (C, H, N in %) ($C_{22}H_{25}BrF_5N_7$)

calc.: C 46.99, H 4.48, N 17.43.

found: C 46.13, H 4.87, N 17.01.

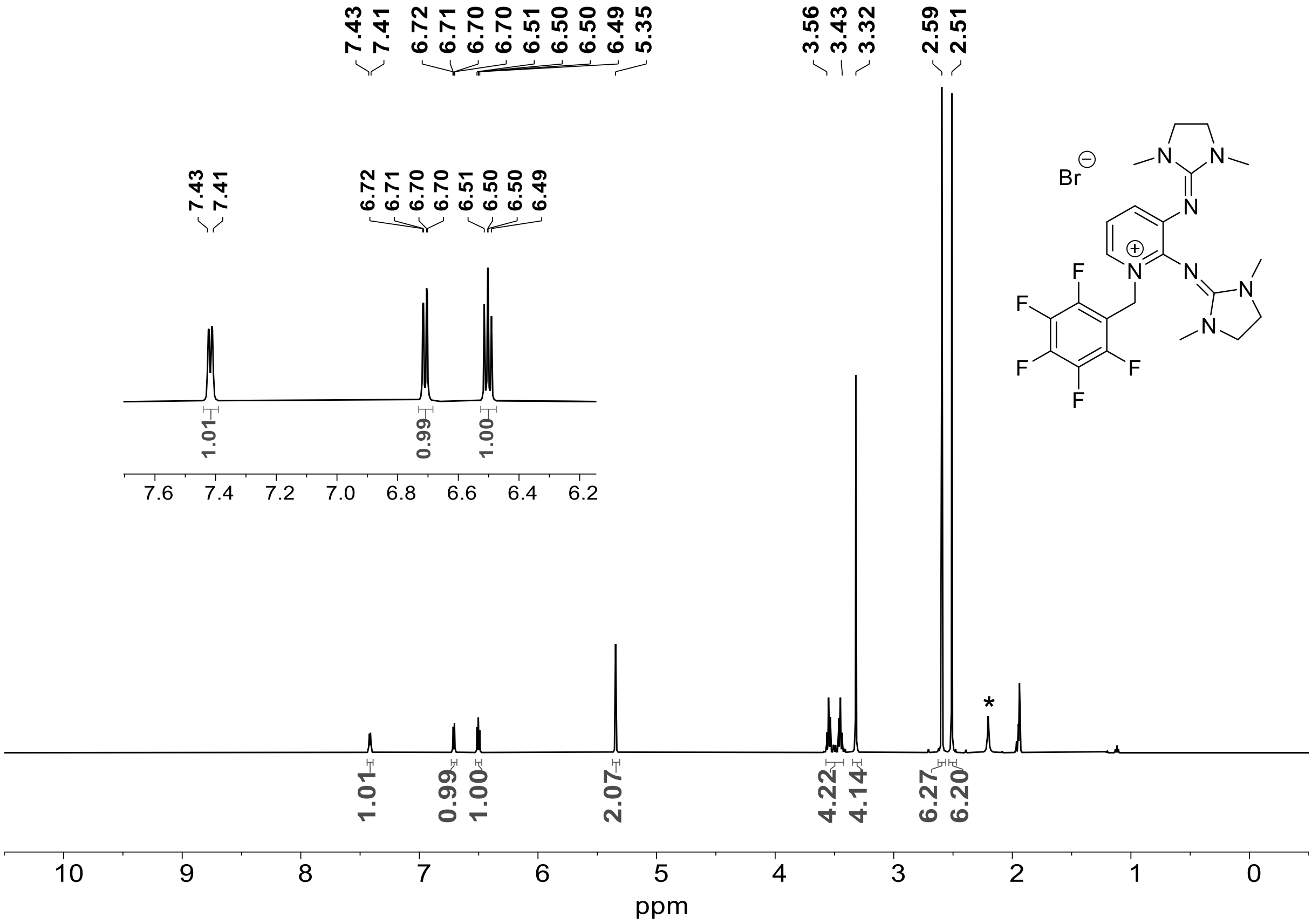

Figure S82: ^{1}H NMR spectrum of [L2a+Bn(F)5]Br, measured at 600 MHz in CD_3CN, *water.

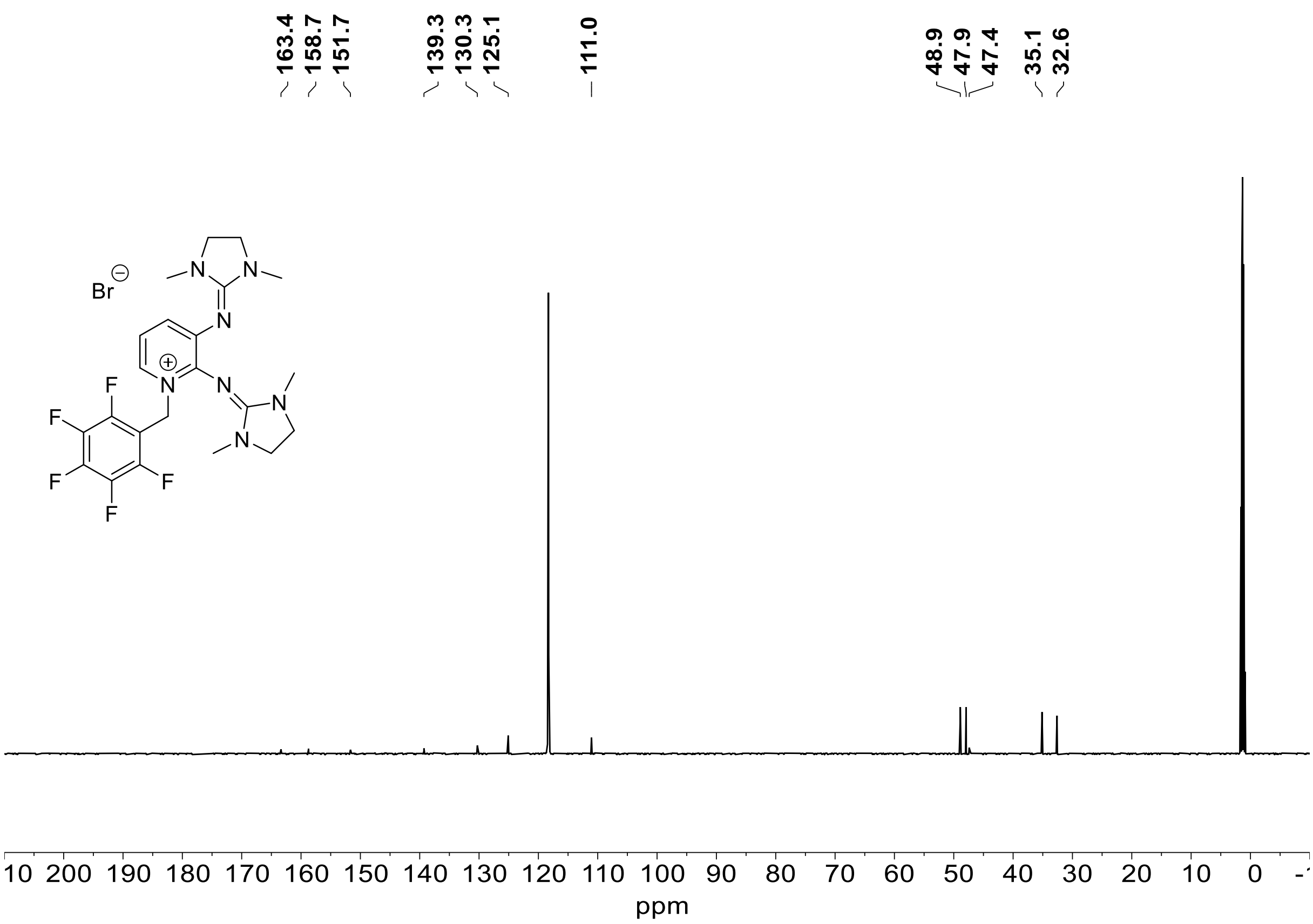

Figure S83: ^{13}C NMR spectrum of [L2a+Bn(F)5]Br, measured at 151 MHz in CD_3CN.

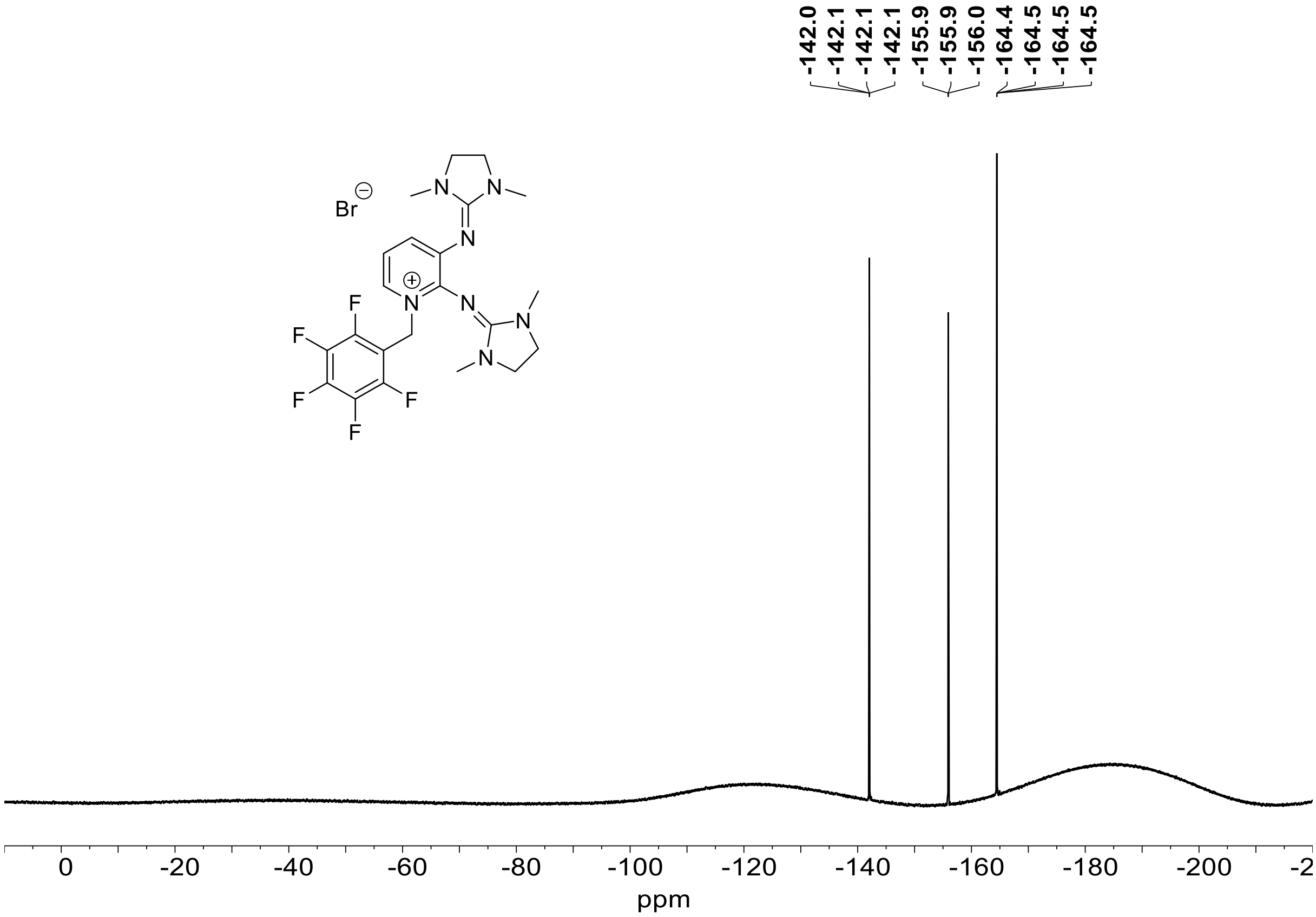

Figure S84: ^{19}F NMR spectrum of [L2a+Bn(F)$_5$]Br, measured at 565 MHz in CD_3CN.

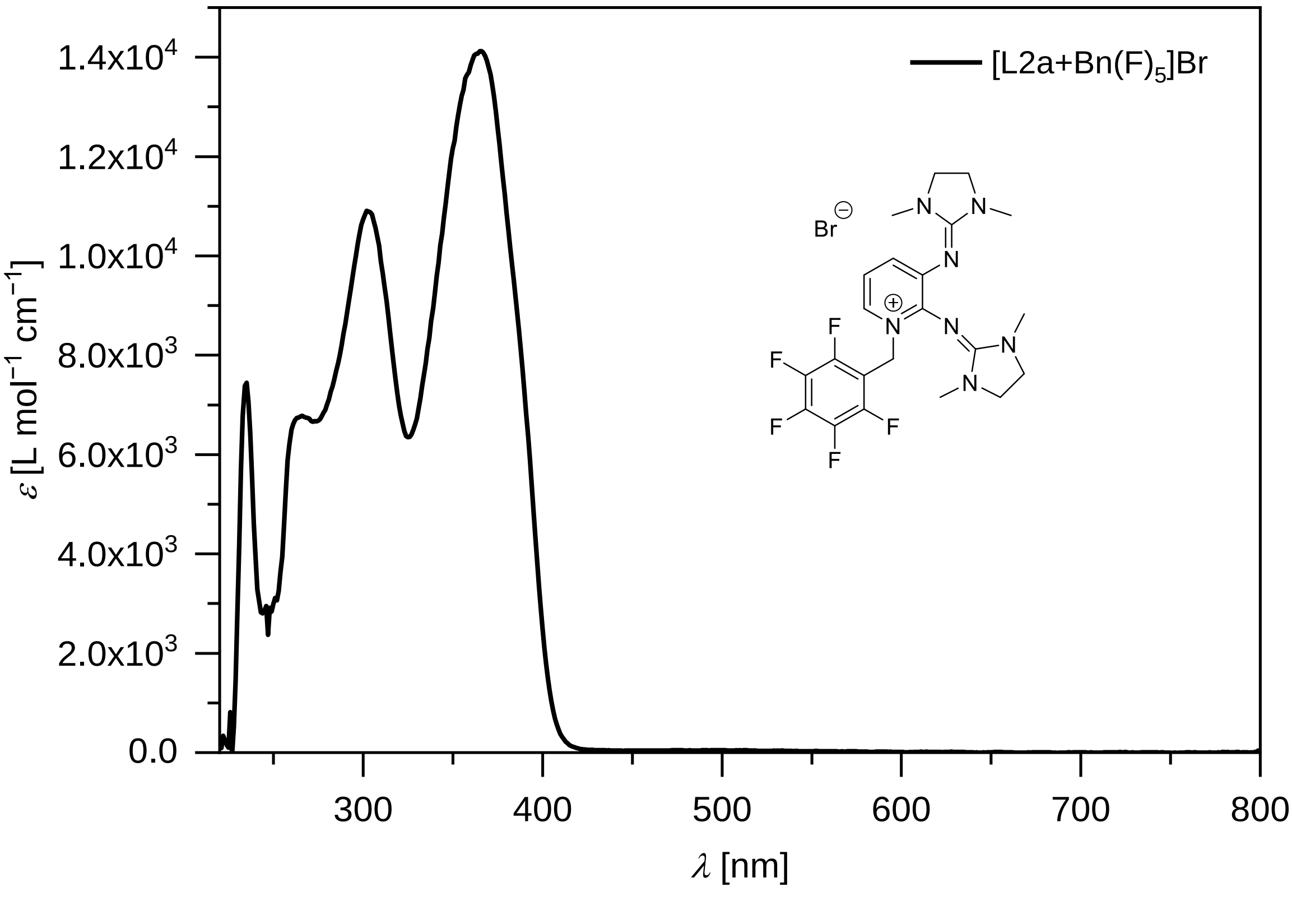

Figure S85: UV-vis absorption spectrum of [L2a+Bn(F)$_5$]Br in dichloromethane at a concentration of $8.29\cdot10^{-5}$ mol L^{-1} at r.t.

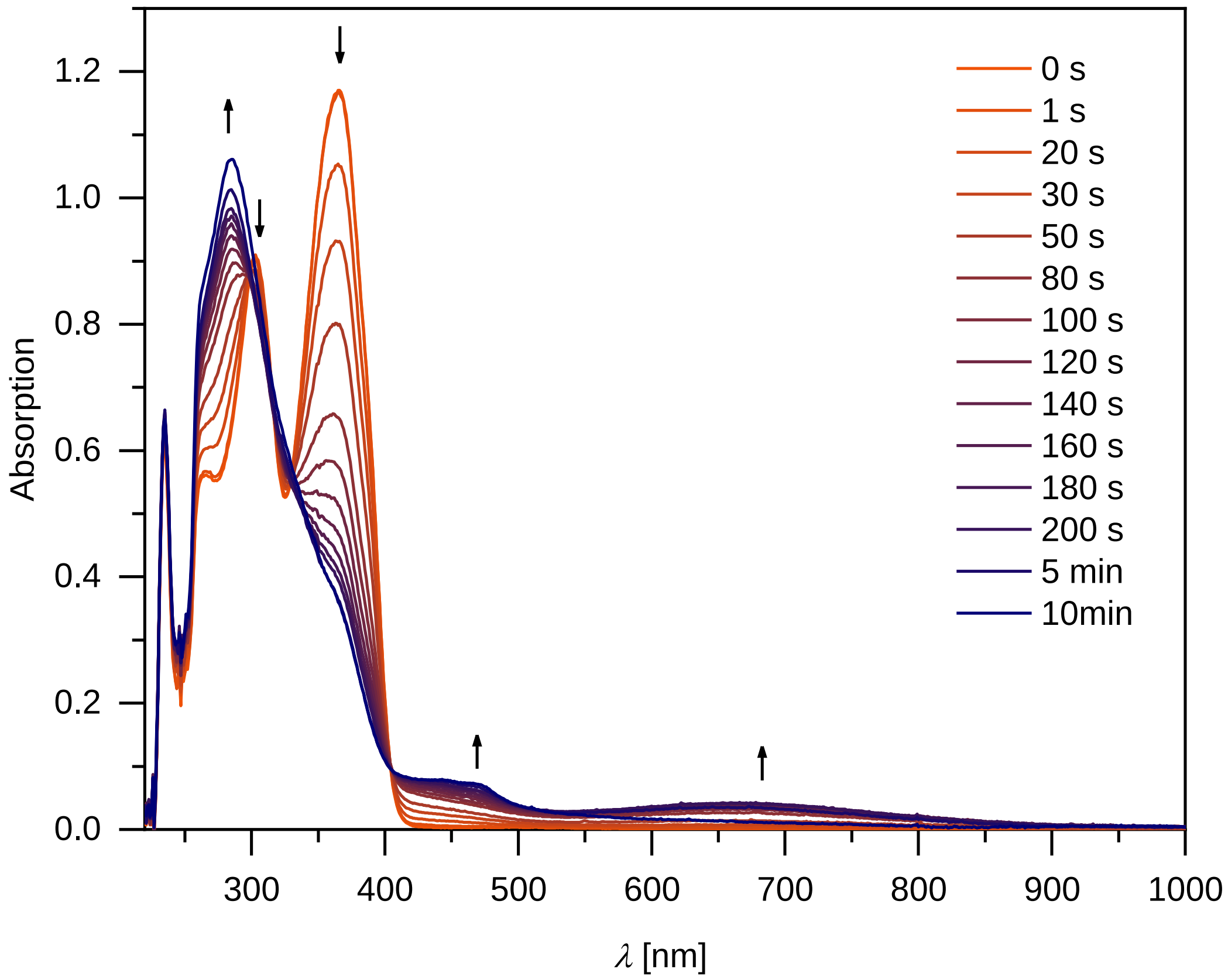

Figure S86: UV-vis absorption spectrum of [L2a+Bn(F)$_5$]Br in dichloromethane at a concentration of $8.29 \cdot 10^{-5}$ mol L^{-1} at r.t. under irradiation with broad spectrum visible light.

2.3.10 [L2a+Br+Bn(*p*-CF_3)]$CuBr_4$

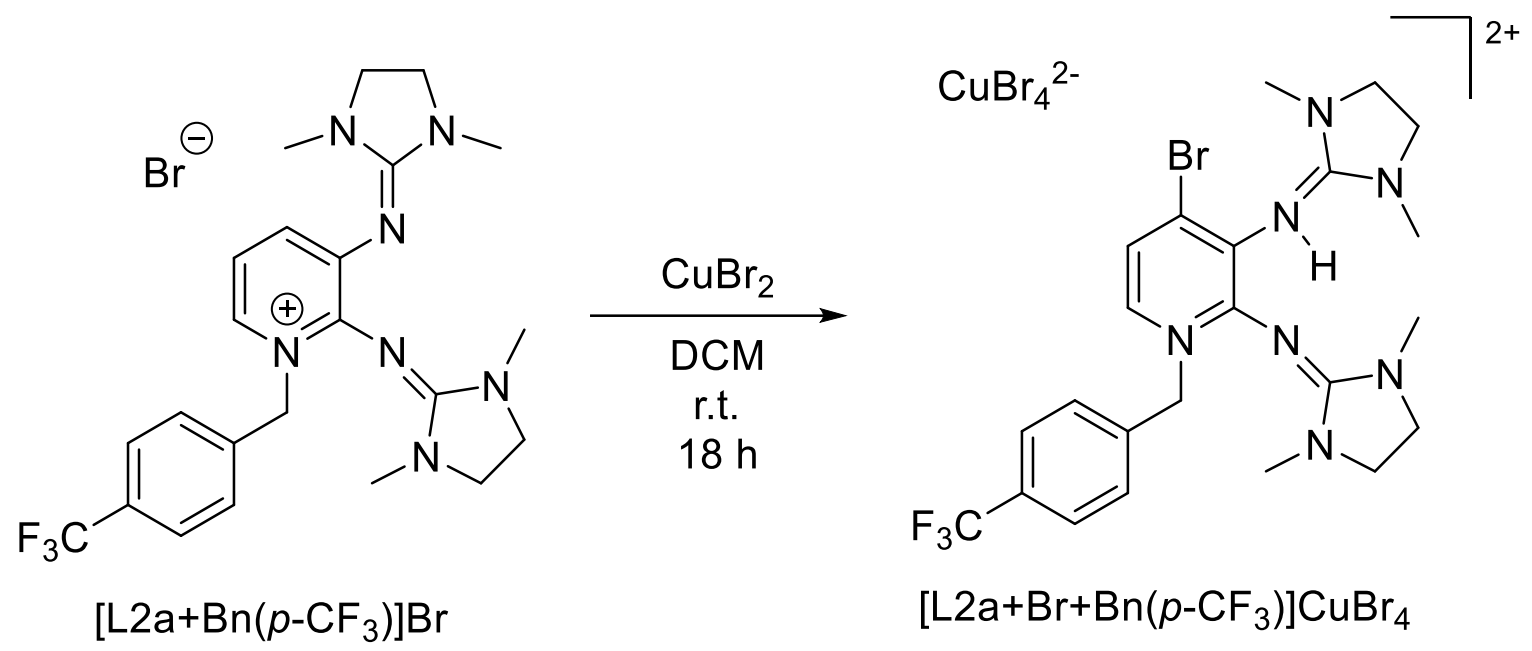

[L2a+Bn(*p*-CF_3)]Br (15.0 mg, 27.8 μmol, 1.00 eq.) and $CuBr_2$ (6.20 mg, 27.8 mmol, 1.00 eq.) were dissolved in 3.0 mL DCM and stirred for 18 h. The solution tuned dark green and the solvent was removed.

A crystal suitable for single crystal structure analysis of [L2a+Br+Bn(*p*-CF_3)]$CuBr_4$ was obtained from an acetonitrile solution by layering with diethyl ether.

2.3.11 [L2b+Bn]Br

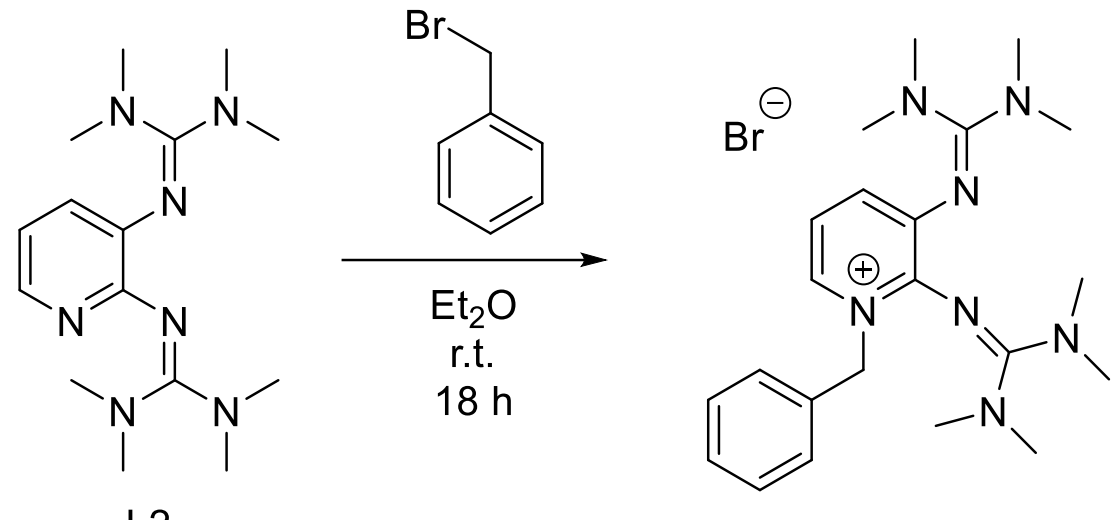

Following general procedure 1, L2a (45.6 mg, 149 µmol, 1.00 eq.) and benzyl bromide (16.9 µL, 24.3 mg, 142 µmol, 0.95 eq.) were used to obtain [L2b+Bn]Br (21.7 mg, 45.5 µmol, 31%) as a light beige solid.

A crystal suitable for single crystal structure analysis was obtained from an acetonitrile solution by layering with diethyl ether.

^{1}H NMR (600,15 MHz, CD_3CN)

δ = 7.40-7.27 (m, 6H), 6.59 (t, J = 7.1, 1H), 6.51 (dd, J = 7.5, 1.4, 1H), 5.37 (s, 2H), 2.70 (s, 12H), 2.68 (s, 12H) ppm.

Mass spectrometry (ESI$^+$ in DCM)

m/z = 396.2871 ([L2b+Bn]$^+$, calc. 396.2870)

CV (DCM, [n-Bu_4N][PF_6], 20 mV s^{-1}, referenced to Fc/Fc$^+$)

E_{Ox}^1 = 501 mV, E_{Ox}^2 = 850 mV.

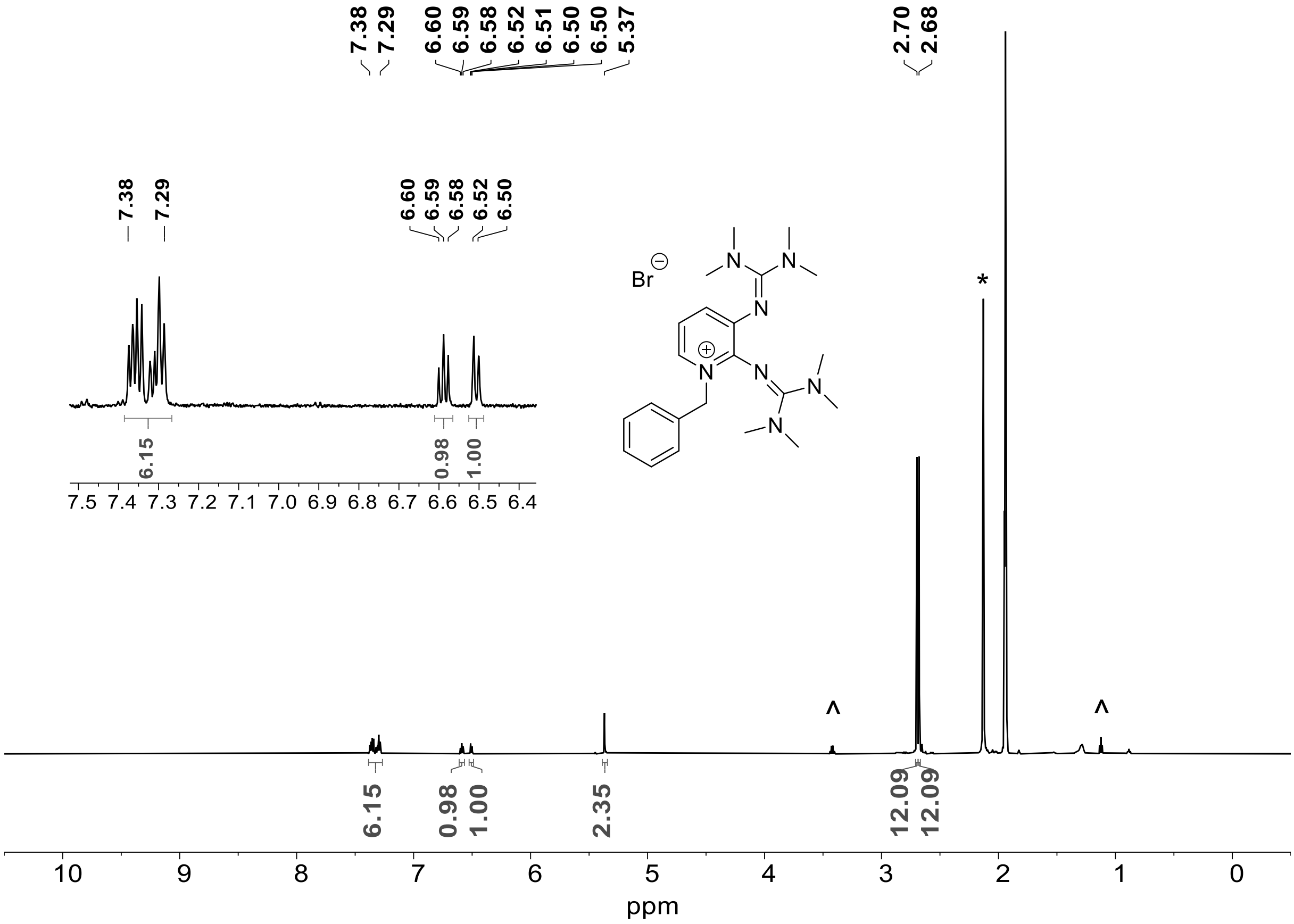

Figure S87: ^{1}H NMR spectrum of [L2b+Bn]Br, measured at 600 MHz in CD_3CN, *water, ^diethyl ether.

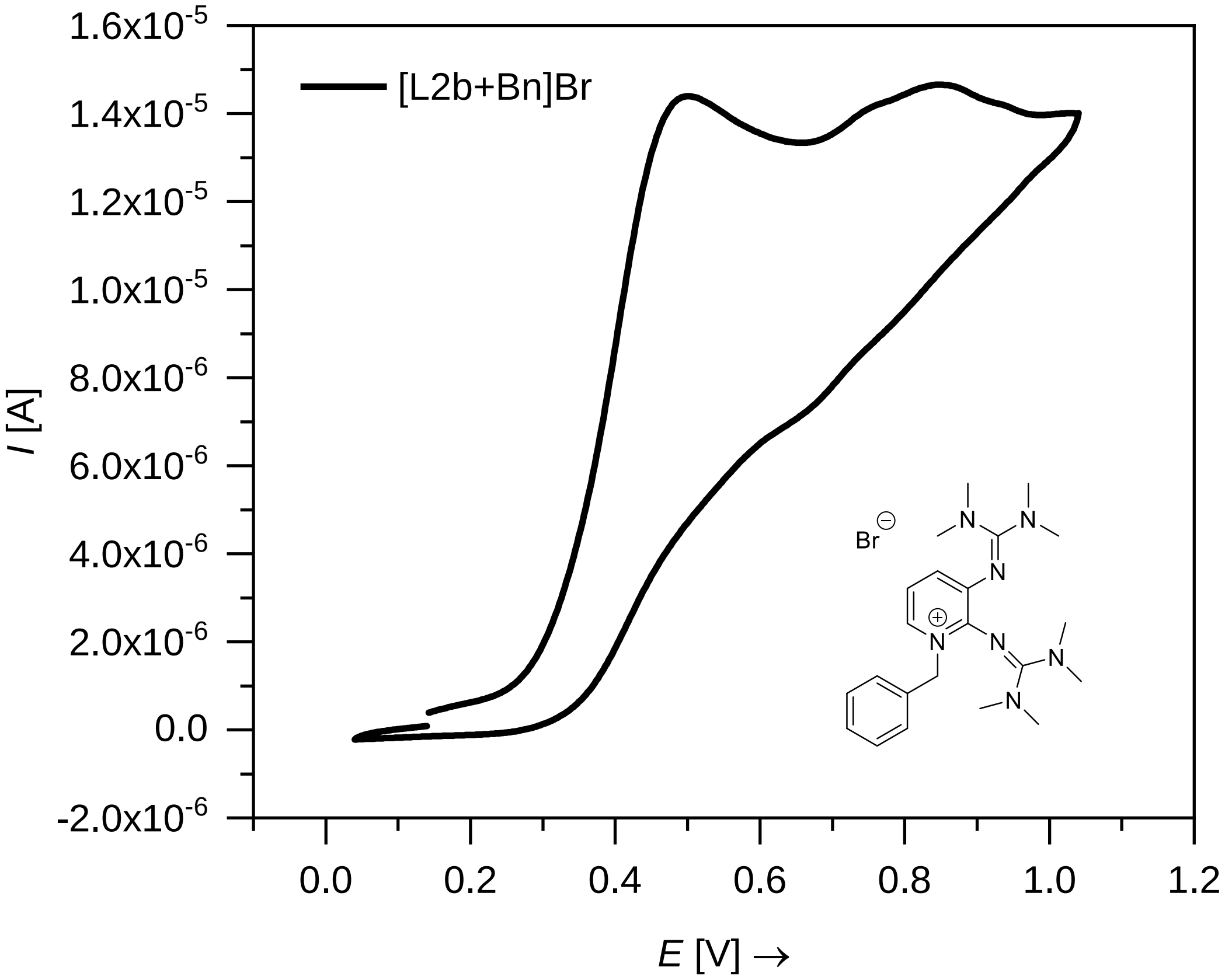

Figure S88: Cyclic voltammogram of [L2b+Bn]Br in dichloromethane ([*n*-Bu_4N][PF_6], 20 mV s^{-1}, referenced to Fc/Fc^+).

2.3.12 [L3+Bn(*p*-CF_3)]Br

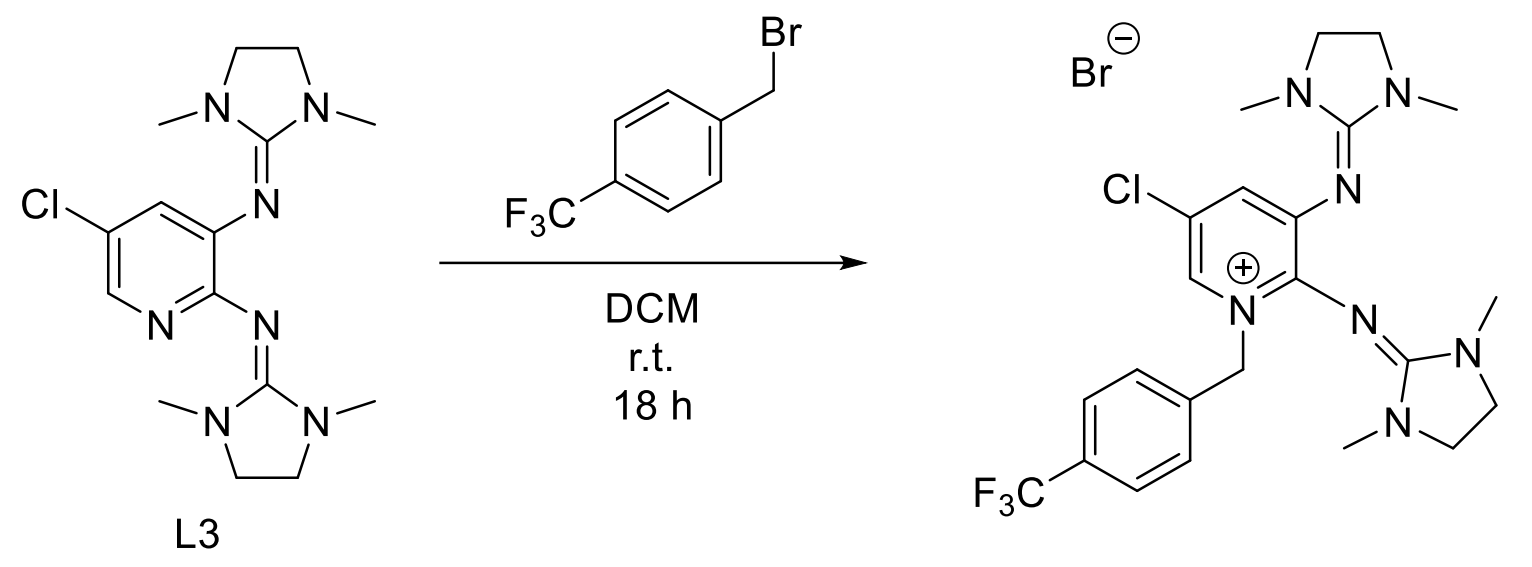

Following general procedure 2, L3 (60.0 mg, 179 μmol, 1.00 eq.) and 4-(trifluoromethyl)benzyl bromide (91.0 mg, 381 μmol, 2.13 eq.) were used to obtain [L3+Bn(*p*-CF_3)]Br (85.0 mg, 172 mmol, 96%) as a light yellow solid.

A crystal suitable for single crystal structure analysis was obtained from an acetonitrile solution by layering with diethyl ether.

^{1}H NMR (400 MHz, CD_3CN)
δ = 7.68 – 7.66 (m, 2H, CH), 7.46 – 7.43 (m, 3H, CH), 6.59 (s, 1H, CH), 5.33 (s, 2H, CH), 3.58 – 3.42 (m, 4H, CH_2), 3.40 (s, $4H_2$, CH_2), 2.65 (s, 6H, CH_3), 2.47 (s, 6H, CH_3) ppm.

^{13}C NMR (101 MHz, CD_3CN) **^{13}C-DEPT-135**, **HSQC**, **HMBC**
δ = 164.2 (1C, C_q), 160.3 (1C, C_q), 150.8 (1C, C_q), 141.2 (1C, C_q), 129.3 (2C, CH), 126.4 (2C, CH), 125. 8 (1C, CH), 122.4 (1C, CH), 57.3 (1C, CH_2), 48.8 (2C, CH_2), 47.9 (2C, CH_2), 35.2 (2C, CH_3), 32.6 (1C, CH_3) ppm.
4 signals are coincident or not observed.

^{19}F NMR (376 MHz, CD_3CN)
δ = −63.08 (s, 3F) ppm.

Mass spectrometry (ESI$^+$ in DCM)
m/*z* = 494.2028 ([L3+Bn(*p*-CF_3)]]$^+$, calc. 494.2041).

UV-vis (DCM) ($8.05\cdot10^{-5}$ mol L^{-1})
λ (ε) = 373 (10600), 310 (8880), 260 (5620), 234 (9240) nm (M^{-1} cm^{-1}).

Elemental analysis (C, H, N in %) ($C_{23}H_{28}BrClF_3N_7$)
Calc.: C 48.05, H 4.91, N 17.06.
found: C 47.66, H 4.96, N 16.37.

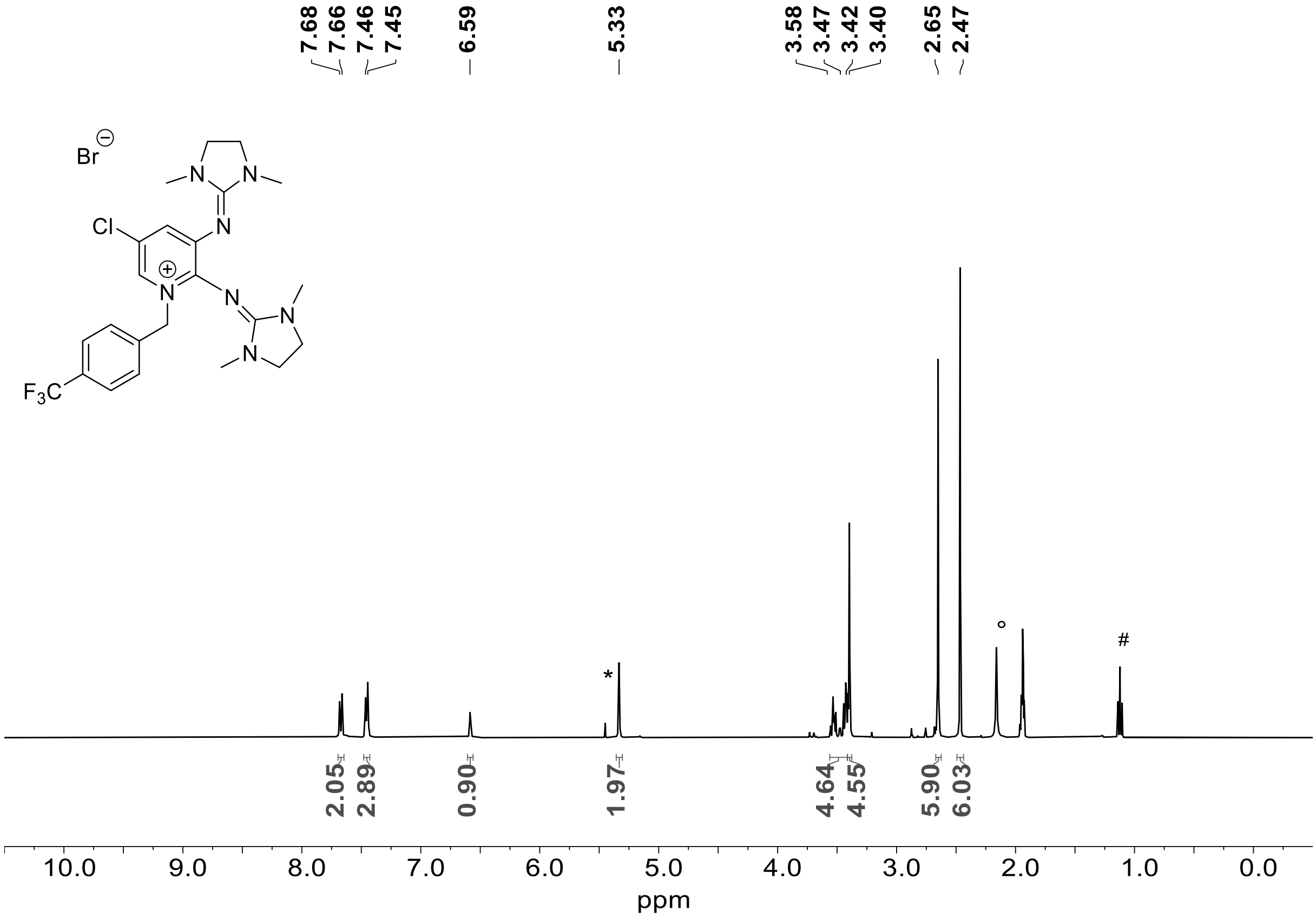

Figure S89: ^{1}H NMR spectrum of [L3+Bn(*p*-CF_3)]Br measured at 400 MHz in CD_3CN, *DCM, °water, #diethyl ether.

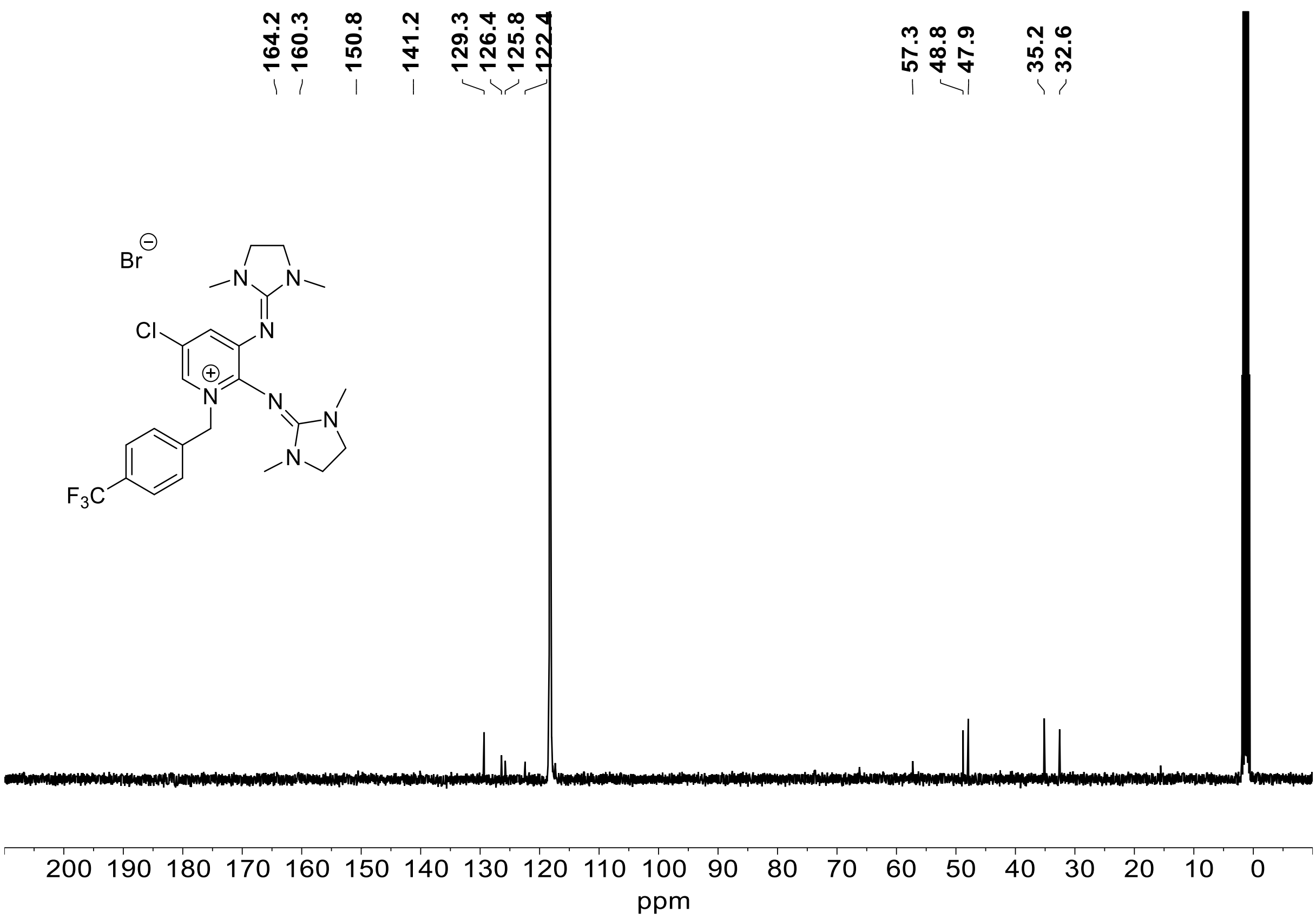

Figure S90: ^{13}C NMR spectrum of [L3+Bn(*p*-CF_3)]Br, measured at 101 MHz in CD_3CN.

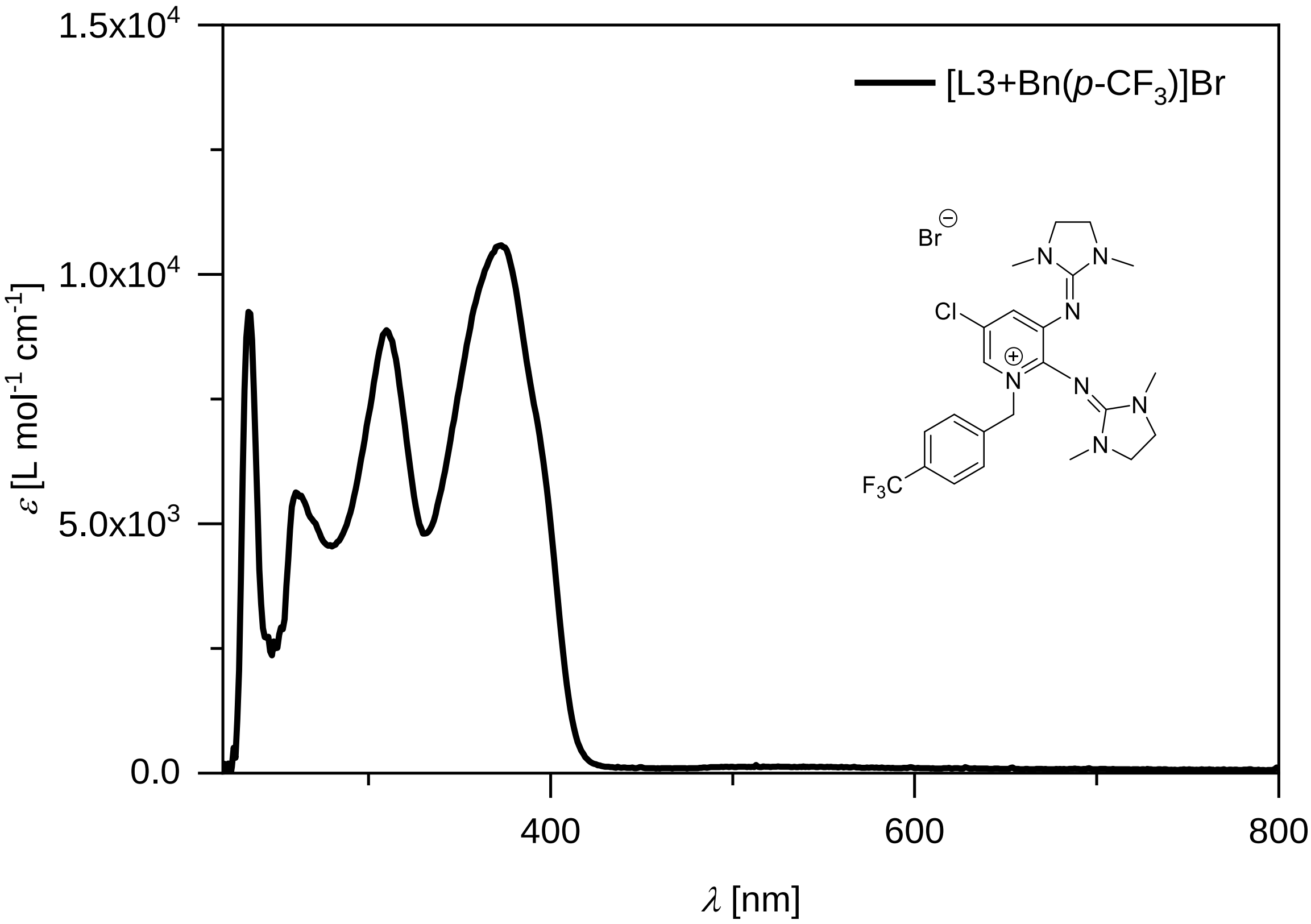

Figure S91: UV-vis absorption spectrum of [L3+Bn(*p*-CF_3)]Br in dichloromethane at a concentration of $8.05 \cdot 10^{-5}$ mol L^{-1} at r.t.

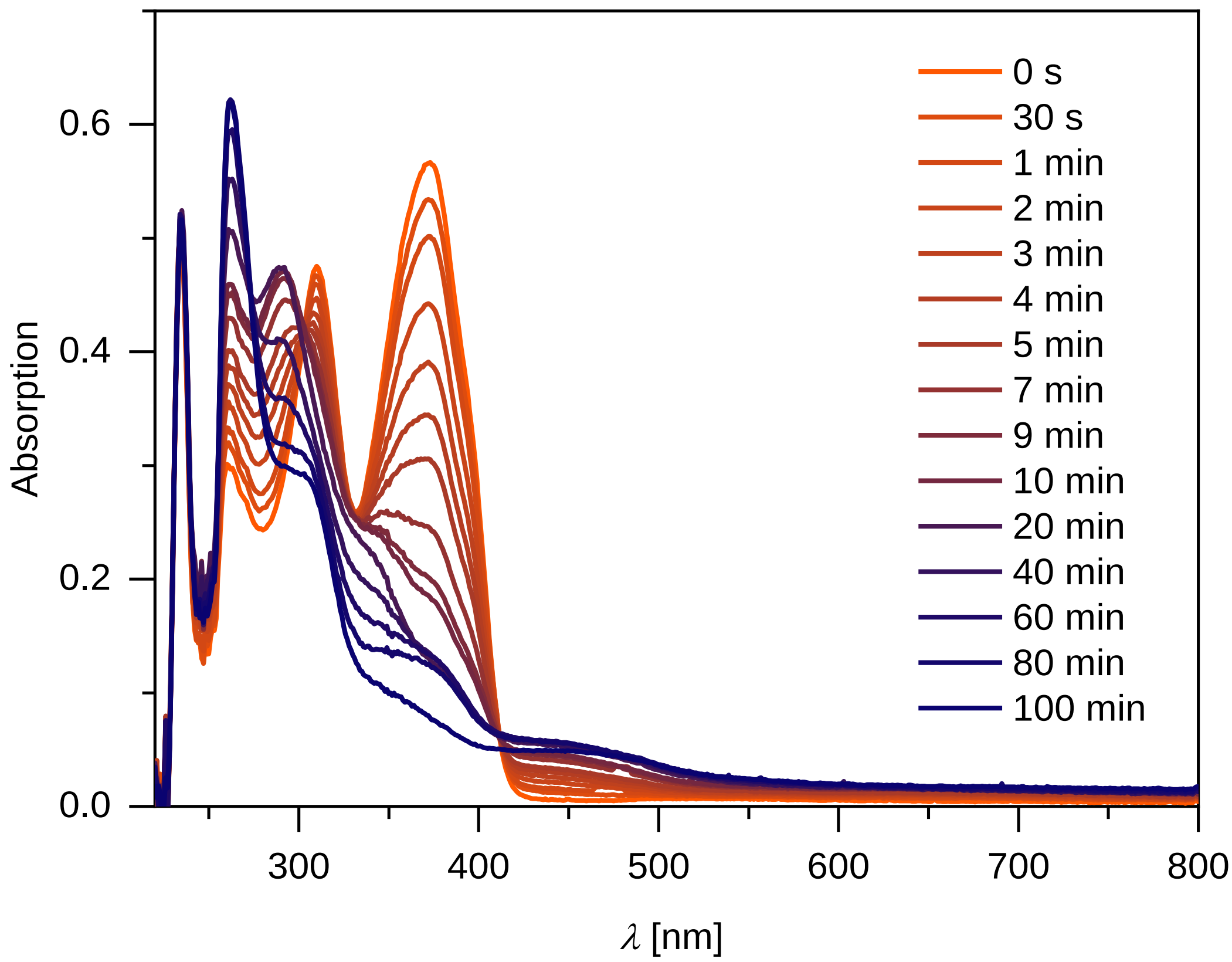

Figure S92: UV-vis absorption spectrum of [L3+Bn(*p*-CF_3)]Br in dichloromethane at a concentration of $3.81 \cdot 10^{-5}$ mol L^{-1} at r.t. under irradiation with broad spectrum visible light.

2.3.13 [L3+Bn(*o*,*p*-CF_3)$_2$]Br

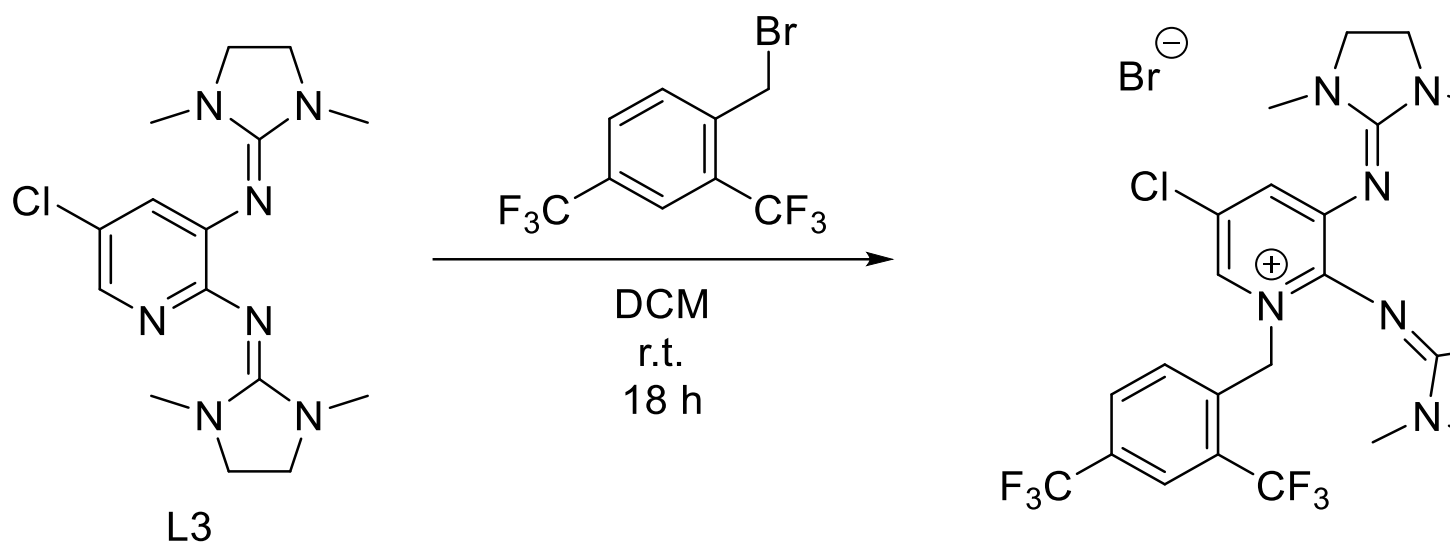

[L3+Bn(*o*,*p*-CF_3)]Br

Following general procedure 2, L3 (60.0 mg, 179 μmol, 1.00 eq.) and 2,4-bis(trifluoromethyl)benzyl bromide (75.0 μL, 117 mg, 382 μmol, 2.14 eq.) were used to obtain [L3+Bn(*o*,*p*-CF_3)$_2$]Br (99.0 mg, 176 μmol, 98%) as a colourless solid.

A crystal suitable for single crystal structure analysis was obtained from an acetonitrile solution by layering with diethyl ether.

^{1}H NMR (400 MHz, CD_3CN)
δ = 8.06 (s, 1H, CH), 7.87 (d, J = 8.3 Hz, 1H, CH), 7.39 – 7.38 (m, 1H, CH), 7.24 (d, J = 7.9 Hz, 1H, CH), 6.62 – 6.61 (m, 1H, CH), 5.51 (s, 2H, CH_2), 3.52 – 3.39 (m, 8H, CH_2), 2.68 (s, 6H, CH_3), 2.45 (s, 6H, CH_3) ppm.

^{19}F NMR (376 MHz, CD_3CN)
δ = −61.61 (s, 3F), −63.37 (s, 3F) ppm.

^{13}C NMR (101 MHz, CD_3CN), **^{13}C-DEPT-135**, **HSQC**, **HMBC**
δ = 164.4 (1C, C_q), 160.3 (1C, C_q), 150.4 (1C, C_q), 140.0 (1C, C_q), 130.5 (1C, CH), 129.1 (1C, C_q), 129.0 (1C, CH), 128.7 (1C, C_q), 125.6 (1C, CH), 124.7 (1C, CH), 122.2 (1C, CH), 117.3 (1C, C_q), 54.6 (1C, CH_2), 48.8 (2C, CH_2), 47.9 (2C, CH_2), 35.2 (2C, CH_3), 32.5 (2C, CH_3) ppm.
2 signals are coincident or not observed.

Mass spectrometry (ESI$^+$ in DCM)
m/z = 562.1901 ([L3+Bn(*o*,*p*-CF_3)]$^+$, calc. 562.1915).

UV-vis (DCM) ($3.83 \cdot 10^{-5}$ mol L^{-1})
λ (ε) = 373 (17700), 311 (14100), 262 (8650), 235 (12200) nm (M^{-1} cm^{-1}).

Elemental analysis (C, H, N in %) ($C_{24}H_{27}BrClF_6N_7$)
calc.: C 44.84, H 4.23, N 15.25.
found: C 44.77, H 4.09, N 15.03.

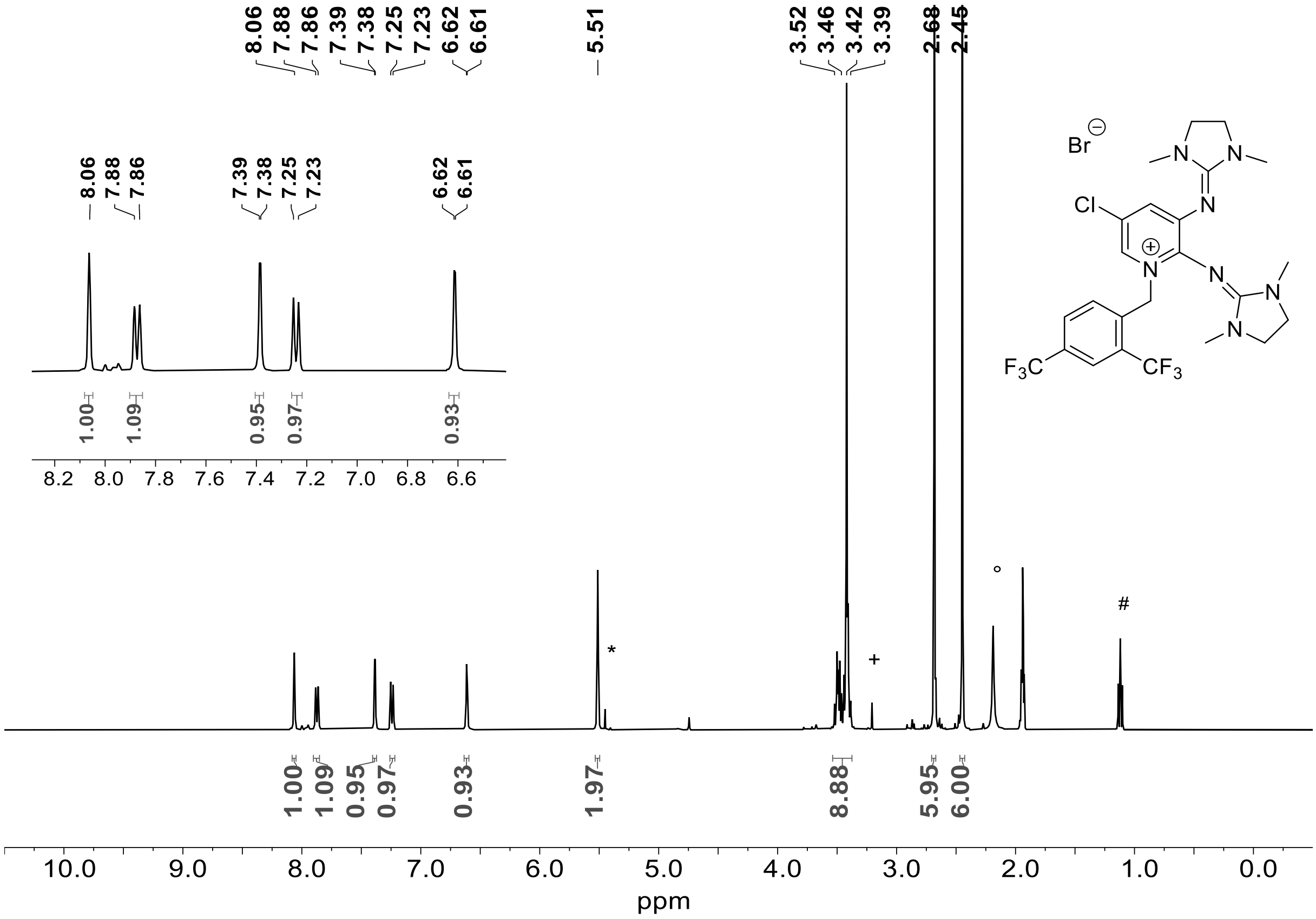

Figure S93: ^{1}H NMR spectrum of [L3+Bn(*o*,*p*-CF_3)$_2$]Br, measured at 400 MHz in CD_3CN, *DCM, °water, #diethyl ether, +deactivated 1.

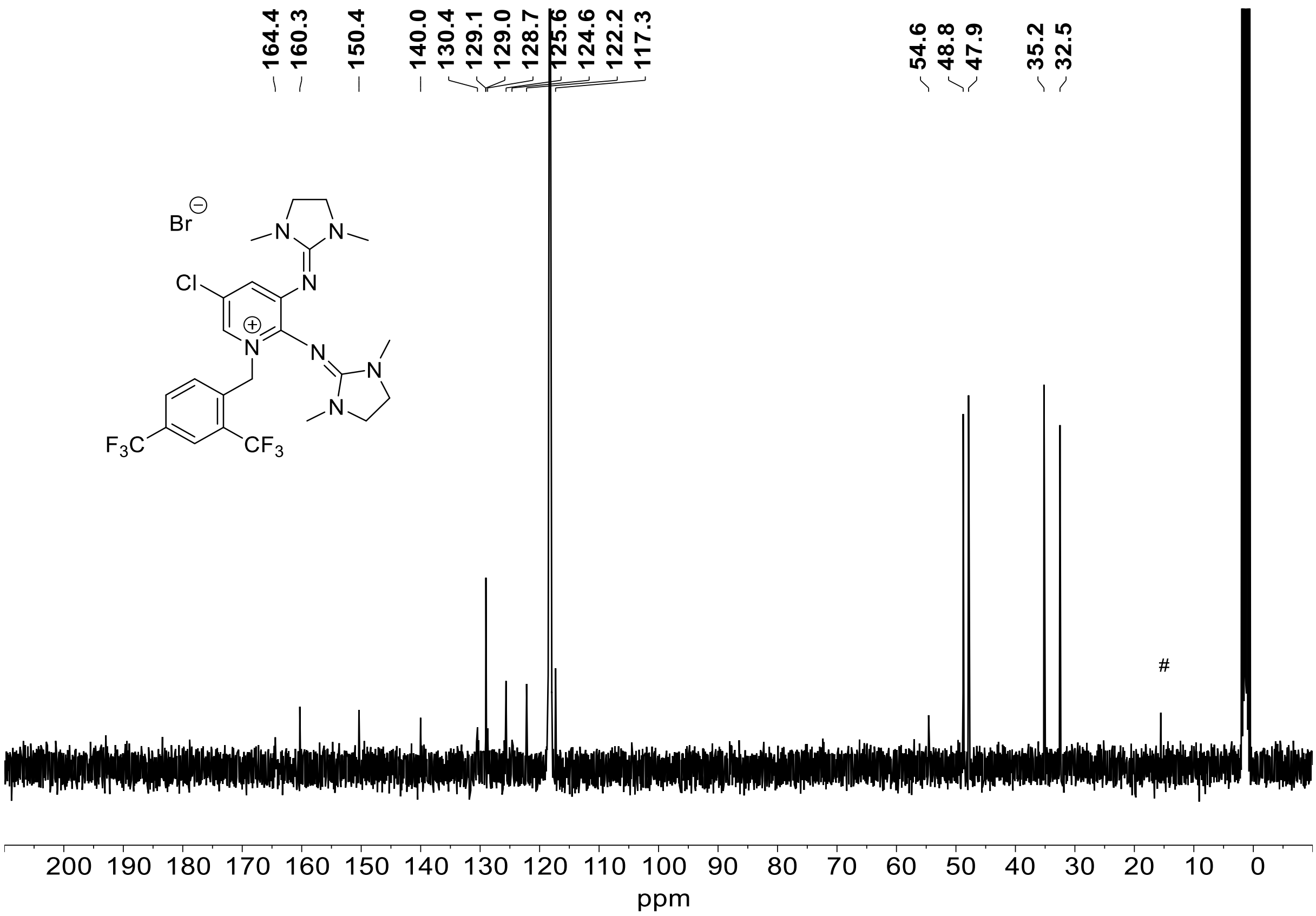

Figure S94: ^{13}C NMR spectrum of [L3+Bn(*o*,*p*-CF_3)$_2$]Br, measured at 101 MHz in CD_3CN, #diethyl ether.

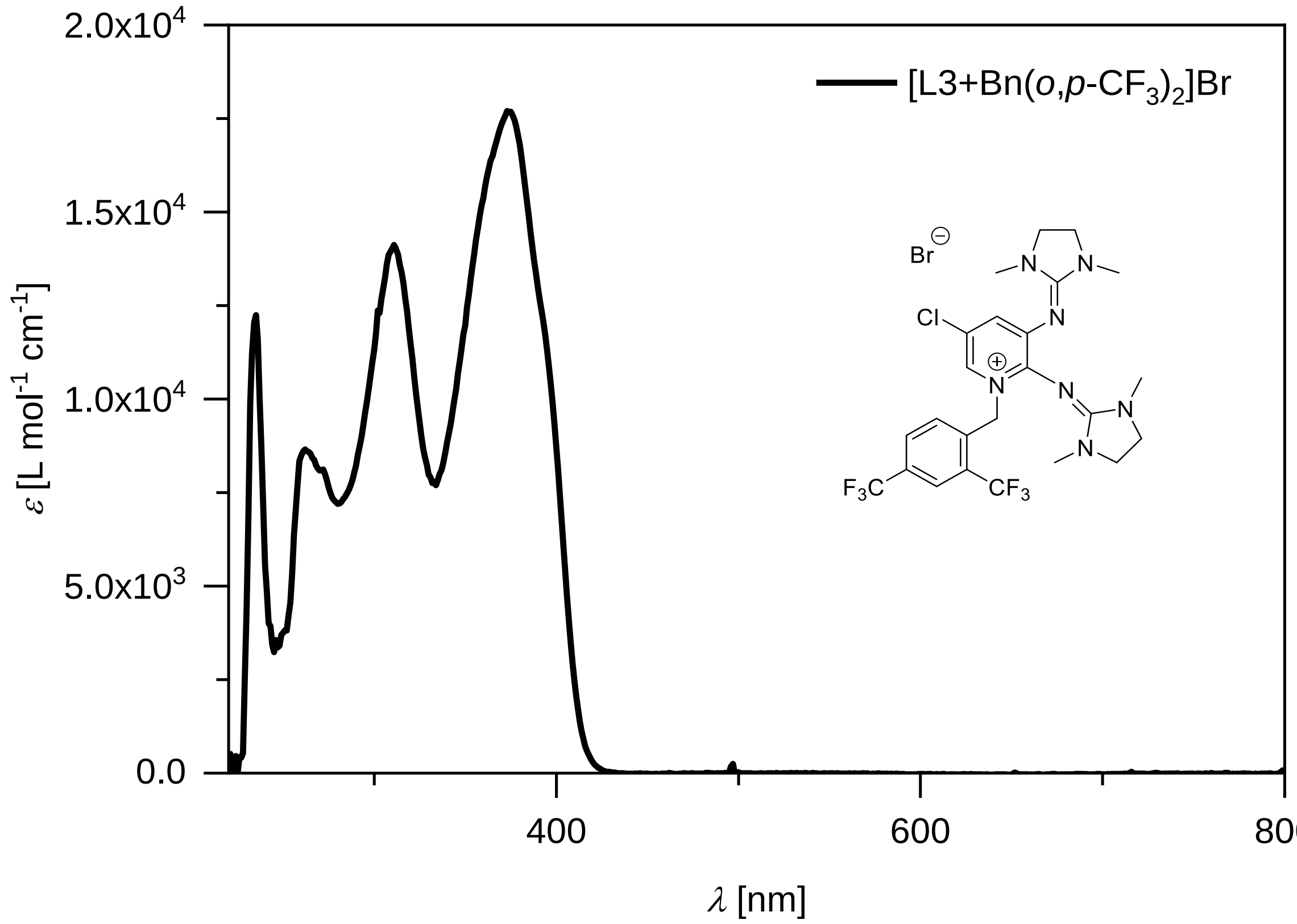

Figure S95: UV-vis absorption spectrum of [L3+Bn(*o*,*p*-CF_3)$_2$]Br in dichloromethane at a concentration of $3.83 \cdot 10^{-5}$ mol L^{-1} at r.t.

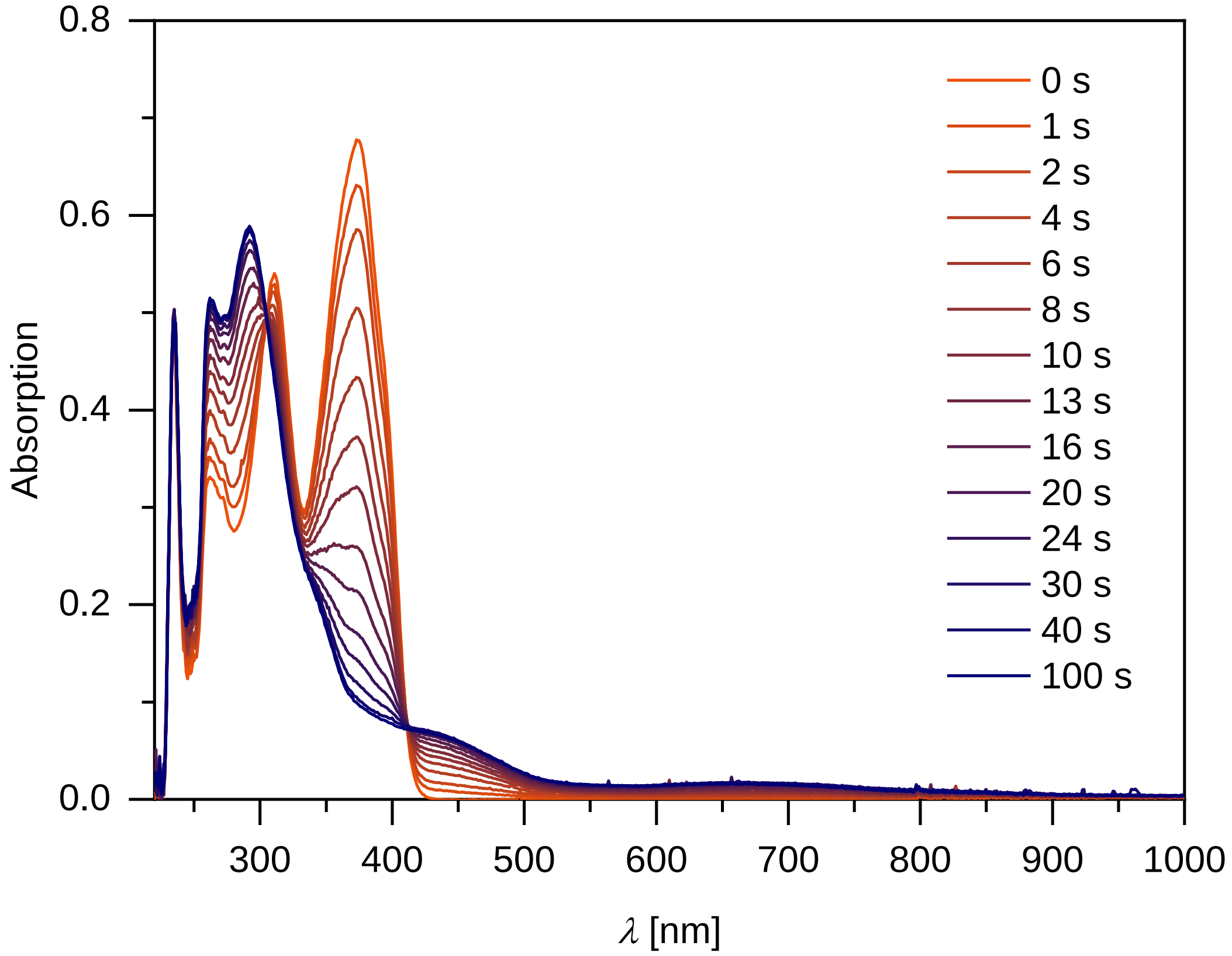

Figure S96: UV-vis absorption spectrum of [L3+Bn(*o*,*p*-CF_3)$_2$]Br in dichloromethane at a concentration of $3.83 \cdot 10^{-5}$ mol L^{-1} at r.t. under irradiation with broad spectrum visible light.

2.3.14 [L4+Bn(*p*-CF_3)]Br

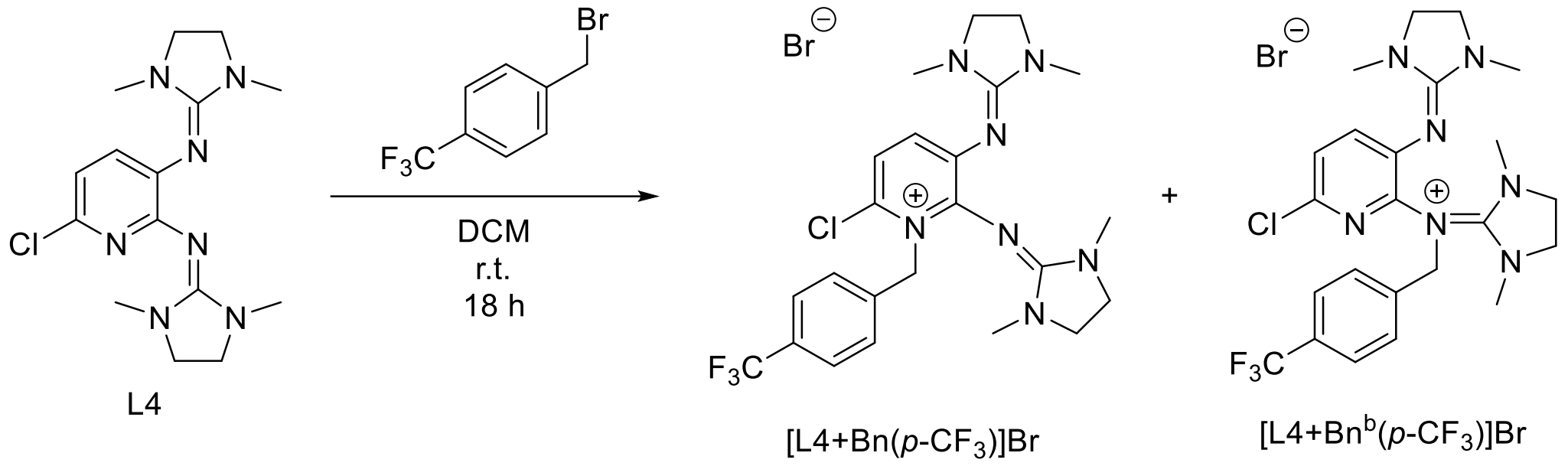

Following general procedure 2, L4 (60.0 mg, 179 μmol, 1.00 eq.) and 4-(trifluoromethyl)benzyl bromide (91.0 mg, 381 μmol, 2.13 eq.) were used to obtain [L4+Bn(*p*-CF_3)]Br and [L4+Bn^b(*p*-CF_3)]Br in a 1:2.4 ratio (53.5 mg, 83.1 μmol, 99%) as a light yellow solid.

^{1}H NMR [L4+Bn(*p*-CF_3)]Br (600 MHz, CD_3CN)

δ = 7.69 – 7.61 (m, 2H, CH), 7.40 (d, *J* = 7.9 Hz, 2H, CH), 6.67 – 6.59 (m, 2H, CH), 5.71 (s, 4H, CH_2), 3.56 – 3.43 (m, 4H, CH_2), 2.66 (s, 6H, CH_3), 2.52 (s, 6H, CH_3) ppm.

^{1}H NMR [L4+Bn^b(*p*-CF_3)]Br (600 MHz, CD_3CN)

δ = 7.69 – 7.61 (m, 4H, CH), 7.06 (dd, *J* = 36.7, 8.3 Hz, 2H, CH), 5.11 (s, 2H, CH_2), 3.74 (s, 4H, CH_2), 3.40 (s, 4H, CH_2), 2.75 (s, 6H, CH_3), 2.66 (s, 6H, CH_3) ppm.

UV-vis [L4+Bn^b(*p*-CF_3)]Br (DCM) ($4.06 \cdot 10^{-5}$ mol L^{-1})

λ (ε) = 338 (sh., 6600), 296 (13600), 285 (15100), 279 (sh., 13900), 232 (40900) nm (M^{-1} cm^{-1}).

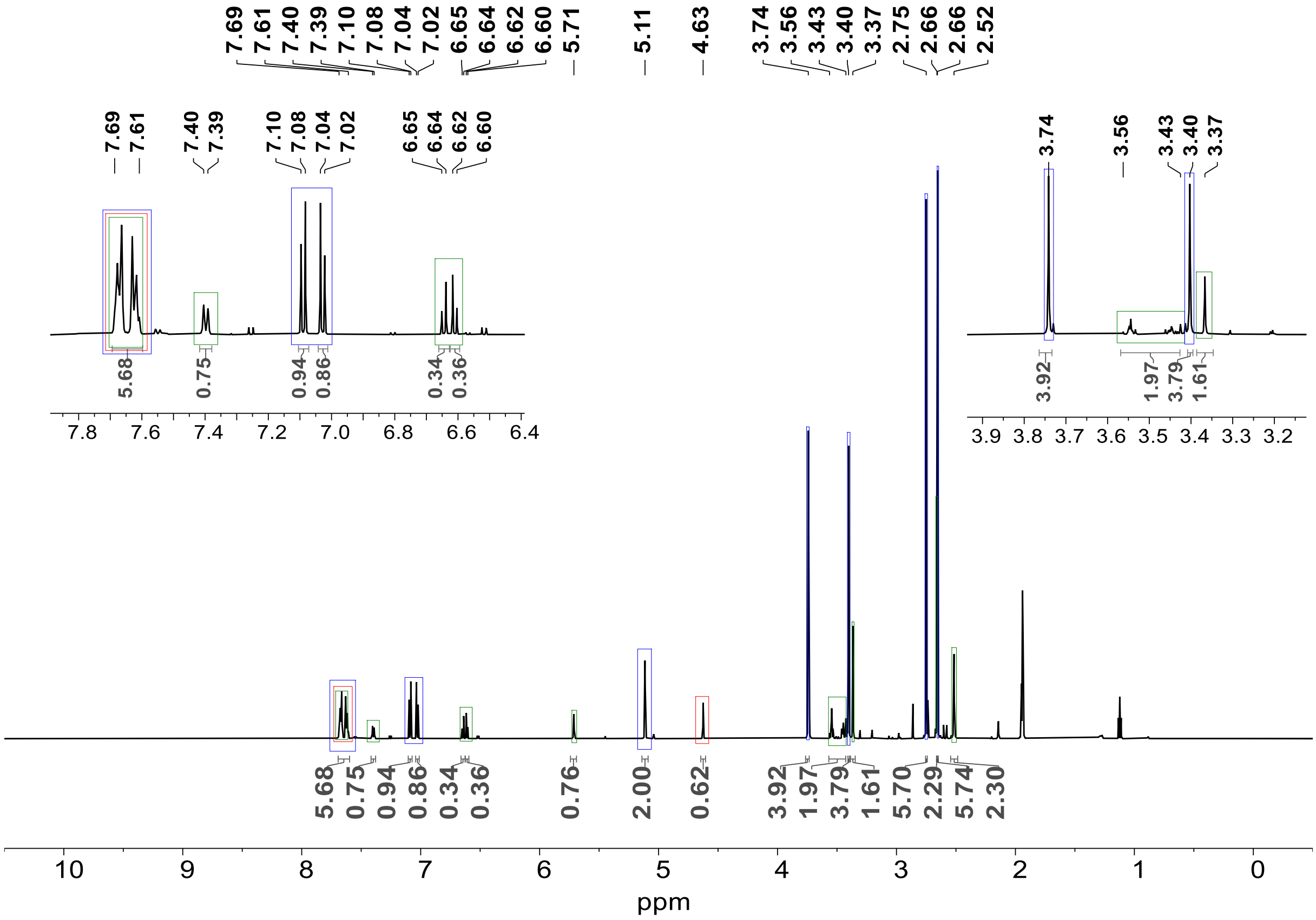

Figure S97: ^{1}H NMR spectrum of [L4+Bn(p-CF_3)]Br in green, [L4+Bn^b(p-CF_3)]Br in blue and Bn(p-CF_3)Br in red with Cs_2CO_3, measured at 600 MHz in CD_3CN.

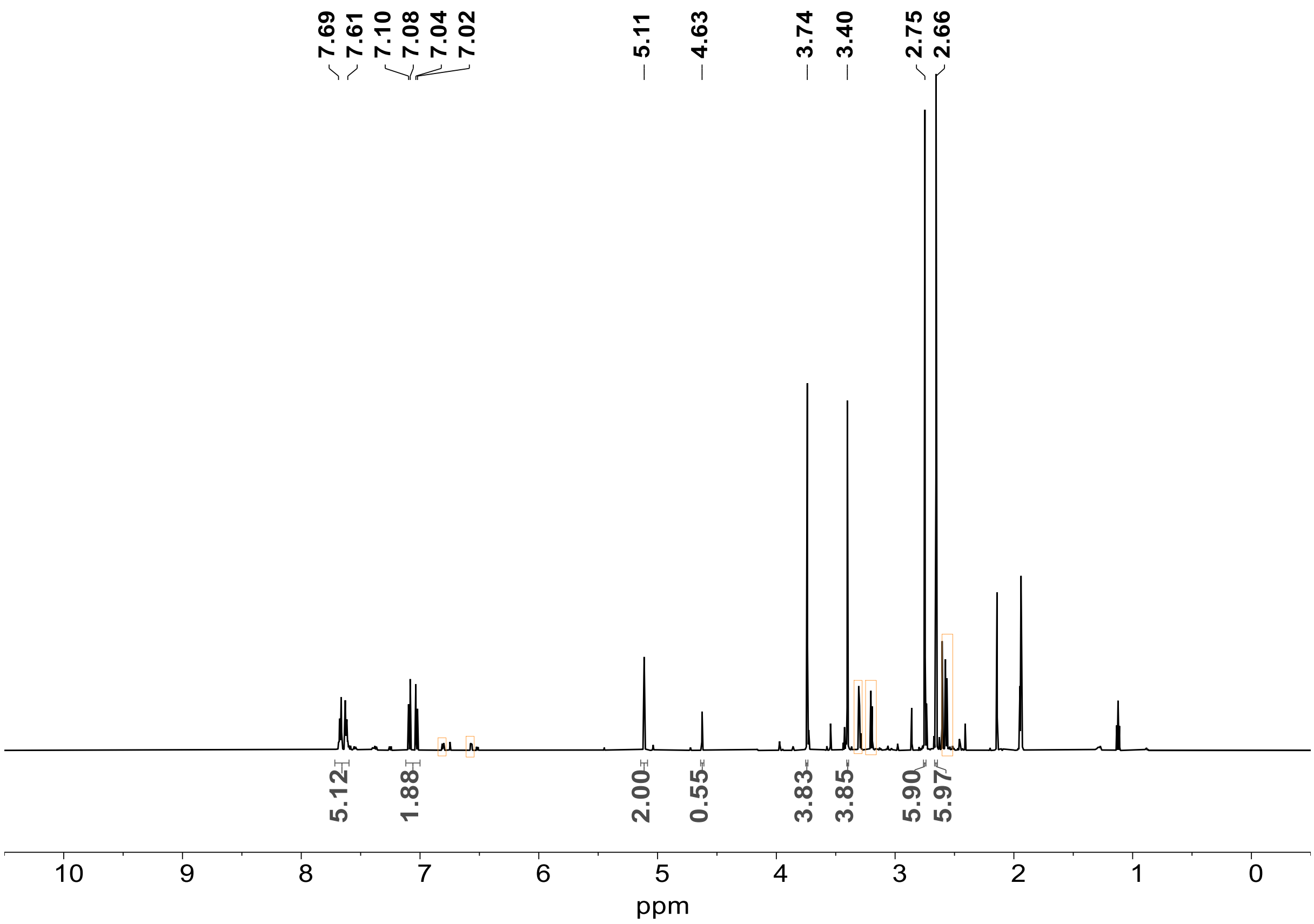

Figure S98: ^{1}H NMR spectrum of the mixture with Cs_2CO_3 after irradiation with visible light for 5 min with peaks of L4 (orange), measured at 600 MHz in CD_3CN.

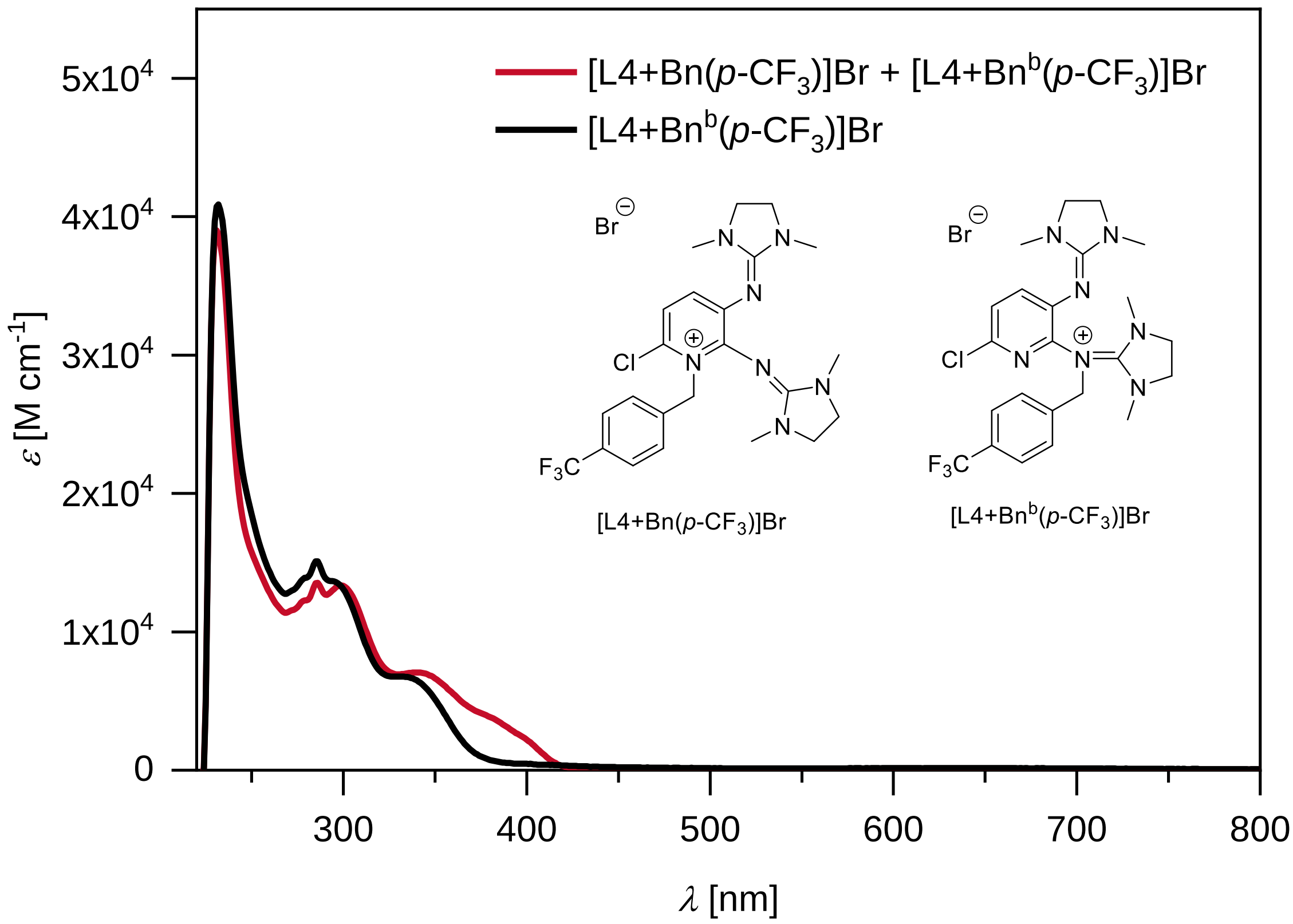

Figure S99: UV-vis absorption spectrum of the mixture and [L4+Bn^{b}(*p*-CF_3)]Br in dichloromethane at a concentration of 1.50·10^{-5} mol L^{-1} at r.t.

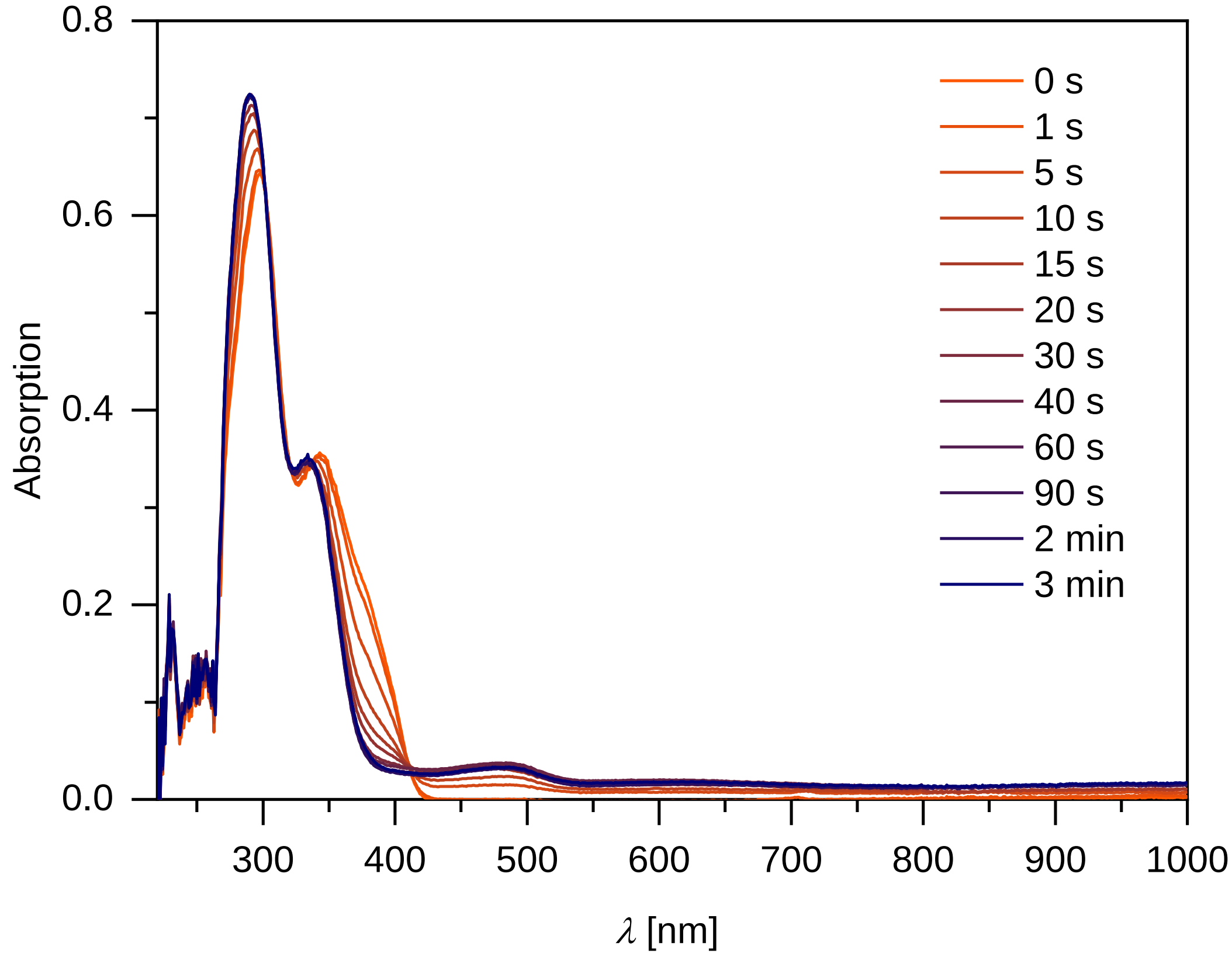

Figure S100: UV-vis absorption spectrum of [L4+Bn(*p*-CF_3)]Br and [L4+Bn^{b}(*p*-CF_3)]Br in dichloromethane at rt under irradiation with broad spectrum visible light.

2.3.15 [L5a+Bn^b(*p*-CF_3)]Br

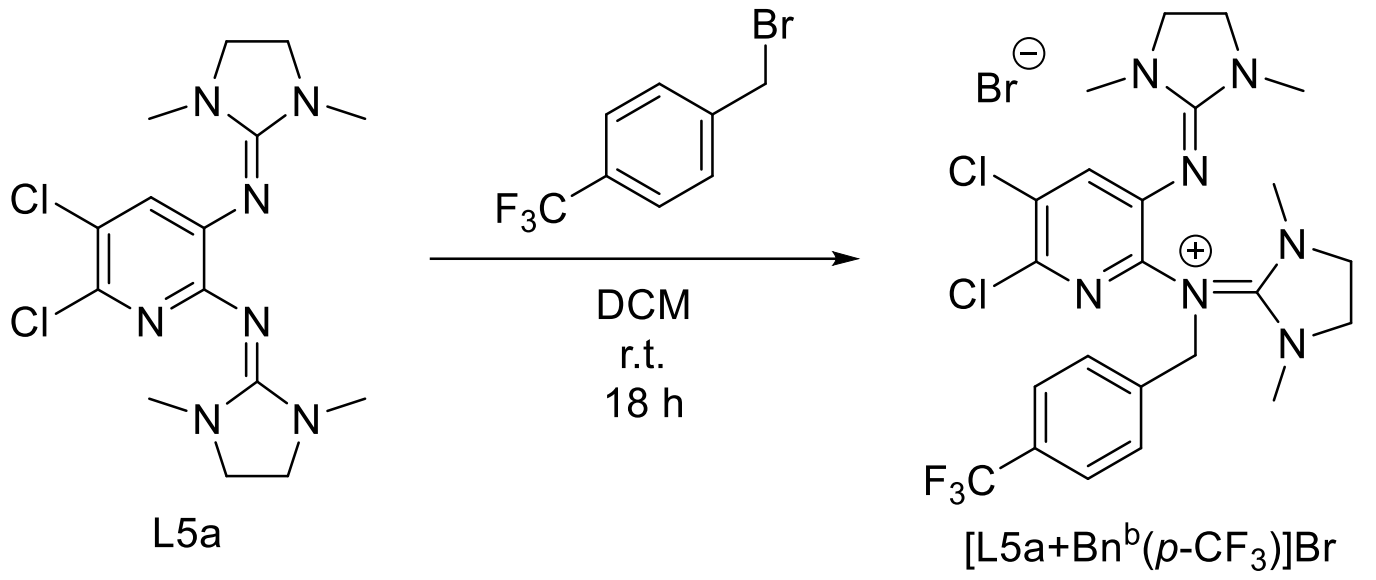

Following general procedure 2, L5a (30.1 mg, 83.7 μmol, 1.00 eq.) and 4-(trifluoromethyl)benzylbromide (40.0 mg, 167 μmol, 2.00 eq.) were used to obtain [L5a+Bn^b(*p*-CF_3)]Br (50.1 mg, 82.2 mmol, 98%) as a yellow-brown solid.

A Crystal for single crystal structure analysis was obtained from an acetonitrile solution by layering with diethyl ether.

^{1}H NMR (400 MHz, CD_3CN)

δ = 7.64 (dd, J = 24.2, 8.5 Hz, 4H), 7.17 (s, 1H), 5.09 (s, 2H), 3.75 (s, 4H), 3.44 (s, 4H), 2.75 (s, 7H), 2.67 (s, 6H) ppm.

^{13}C NMR (101 MHz, CD_3CN), **^{13}C-DEPT-135**, **HSQC**, **HMBC**

δ = 161.6 (1C, C_q), 160.8 (1C, C_q), 139.5 (1C, CH), 130.5 (1C, CH), 129.6 (1C, CH), 126.6 (1C, CH), 126.5 (1C, CH), 53.3 (1C, CH_2), 49.4 (1C, CH_2), 48.9 (1C, CH_2), 35.3 (1C, CH_3), 35.2 (1C, CH_3) ppm.

5 signals are coincident or not observed.

^{19}F NMR (376 MHz, CD_3CN)

δ = −63.0 (3F, CF_3) ppm.

Mass spectrometry (ESI$^+$ in DCM)

m/*z* = 528.1656 ([L5a+Bn^b(*p*-CF_3)]$^+$, calc. 528.1652).

UV-vis (DCM) ($2.20 \cdot 10^{-5}$ mol L^{-1})

λ (ε) = 348 (9930), 301 (17900), 285 (21200), 277 (20800), 230 (84500) nm (M^{-1} cm^{-1}).

Elemental analysis (C, H, N in %) ($C_{23}H_{27}BrCl_2F_3N_7$ · 1.0 eq. H_2O)

calc.: C 44.04, H 4.66, N 16.09.

found: C 43.95, H 5.23, N 15.19.

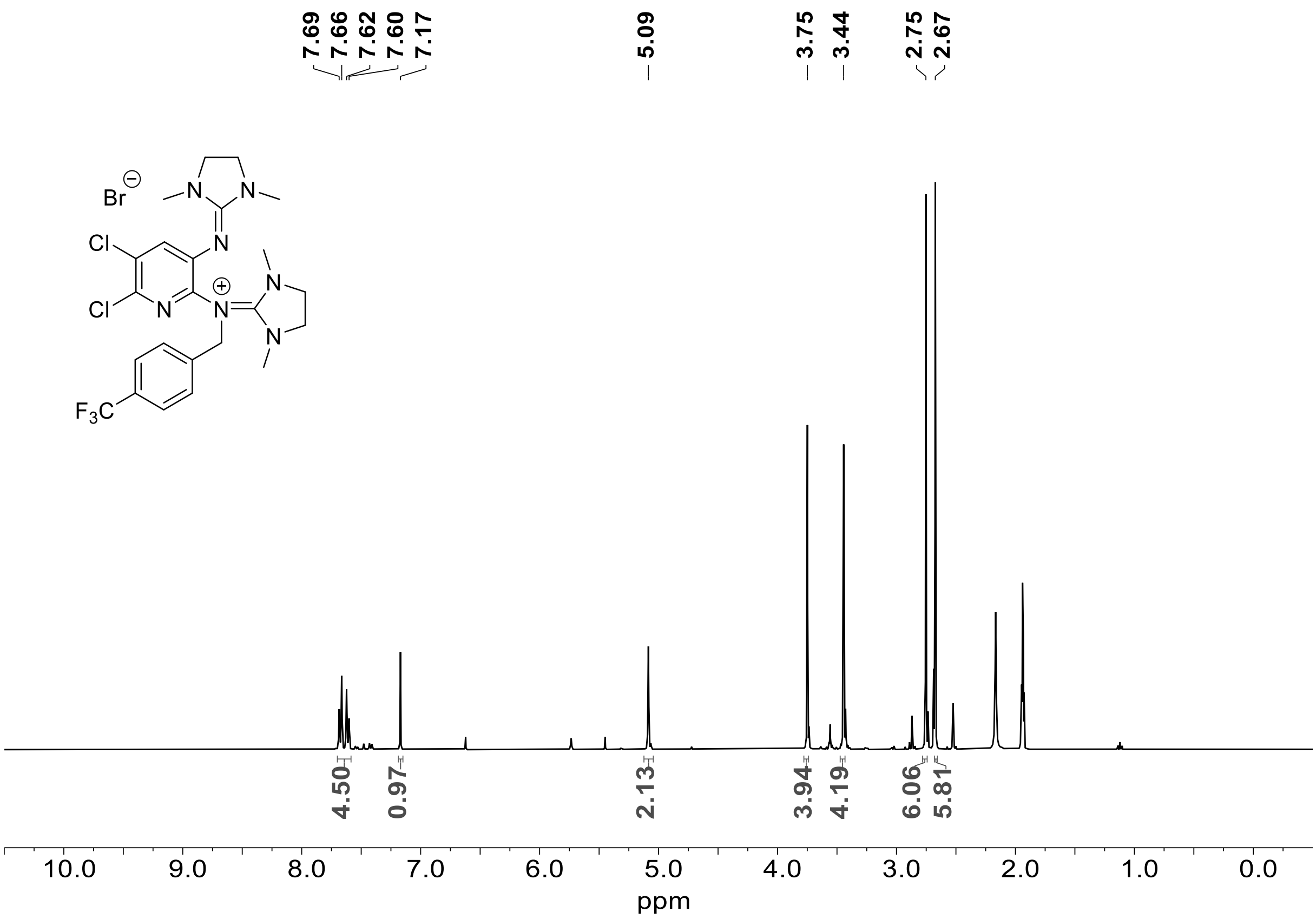

Figure S101: ^{1}H NMR spectrum of [L5a+Bn(*p*-CF_3)]Br measured at 400 MHz in CD_3CN, *DCM, °water, #diethyl ether.

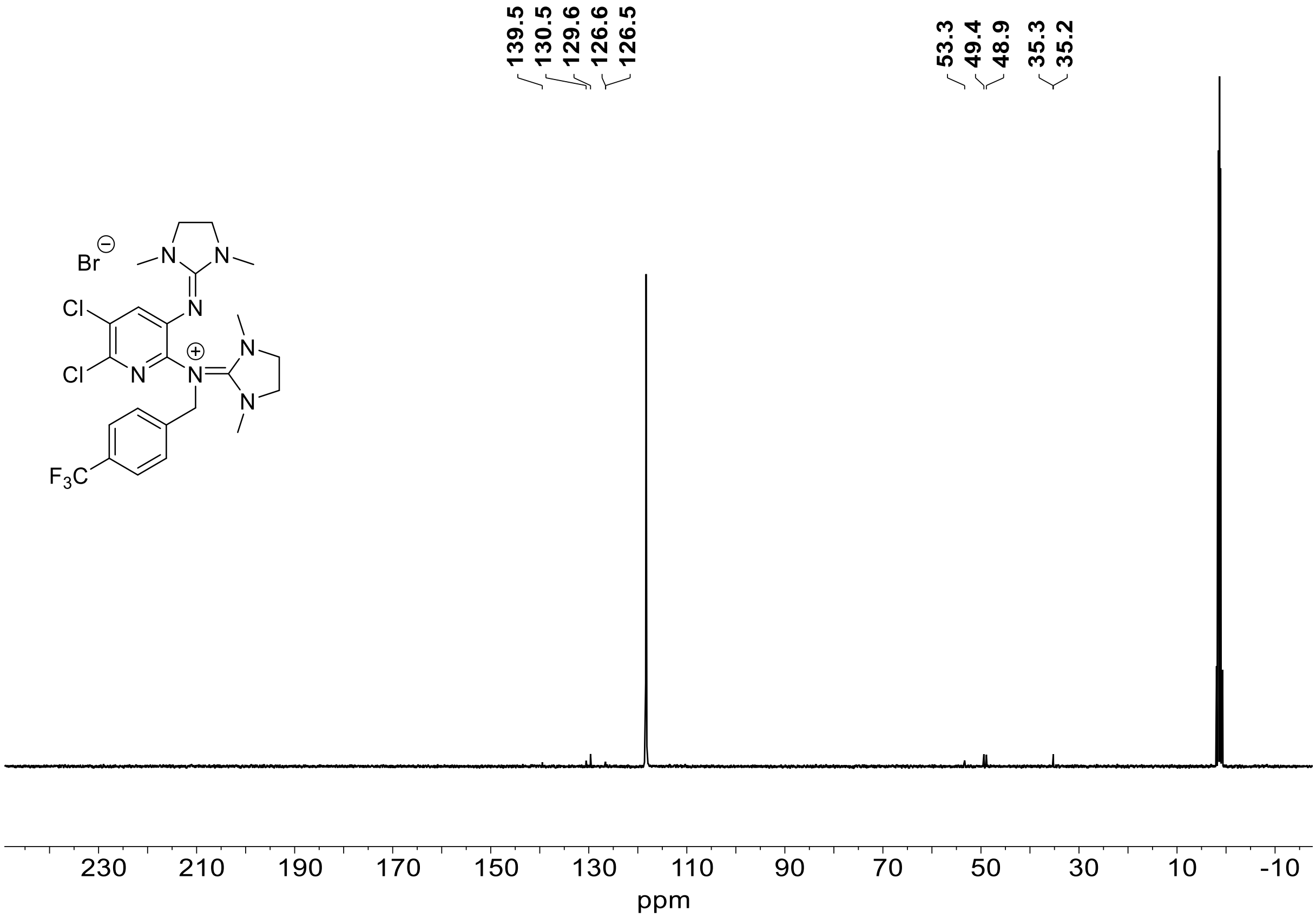

Figure S102: ^{13}C NMR spectrum of [L5a+Bn(*p*-CF_3)]Br, measured at 101 MHz in CD_3CN.

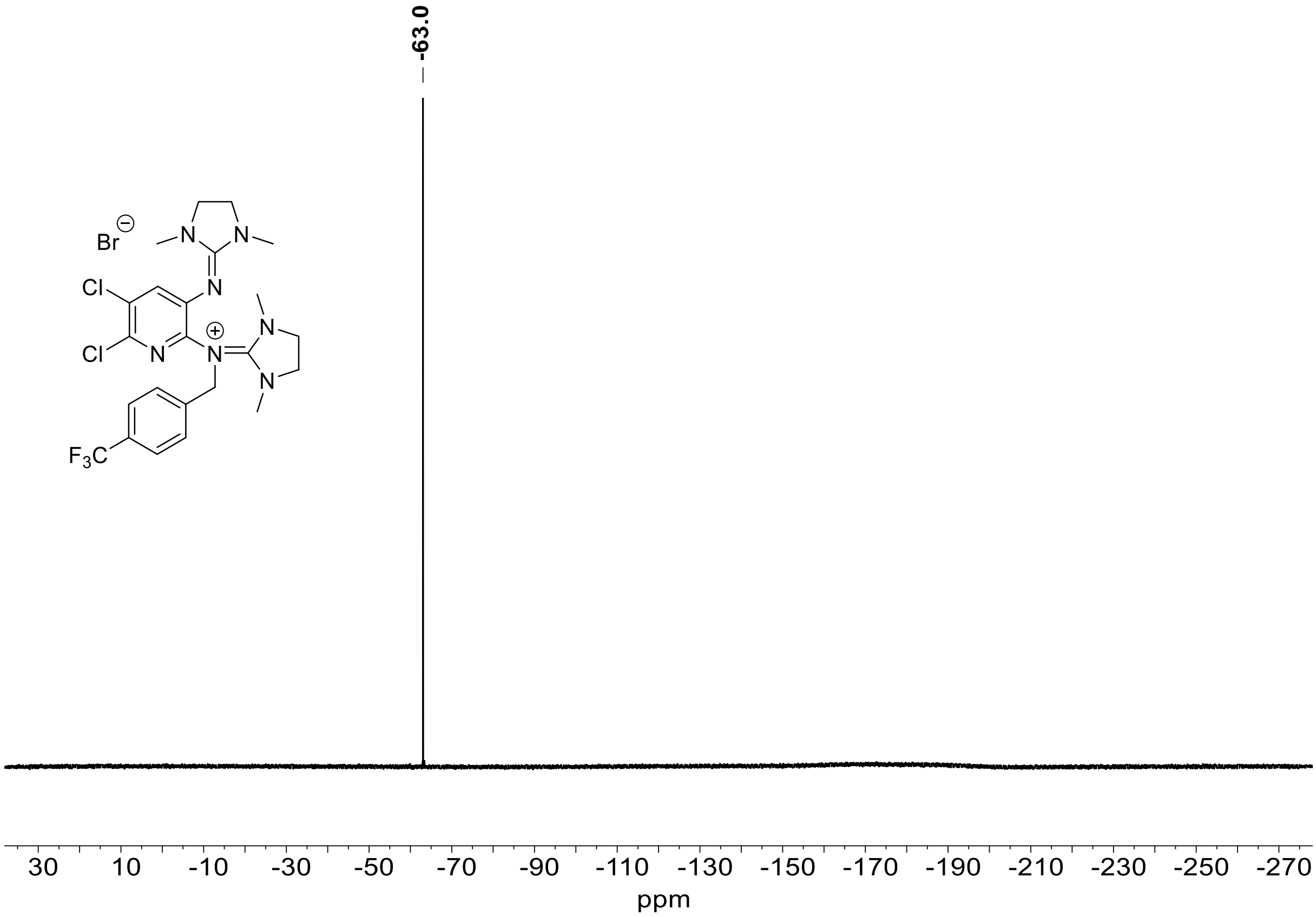

Figure S103: ^{19}F NMR spectrum of [L5a+Bn(*p*-CF_3)]Br, measured at 376 MHz in CD_3CN.

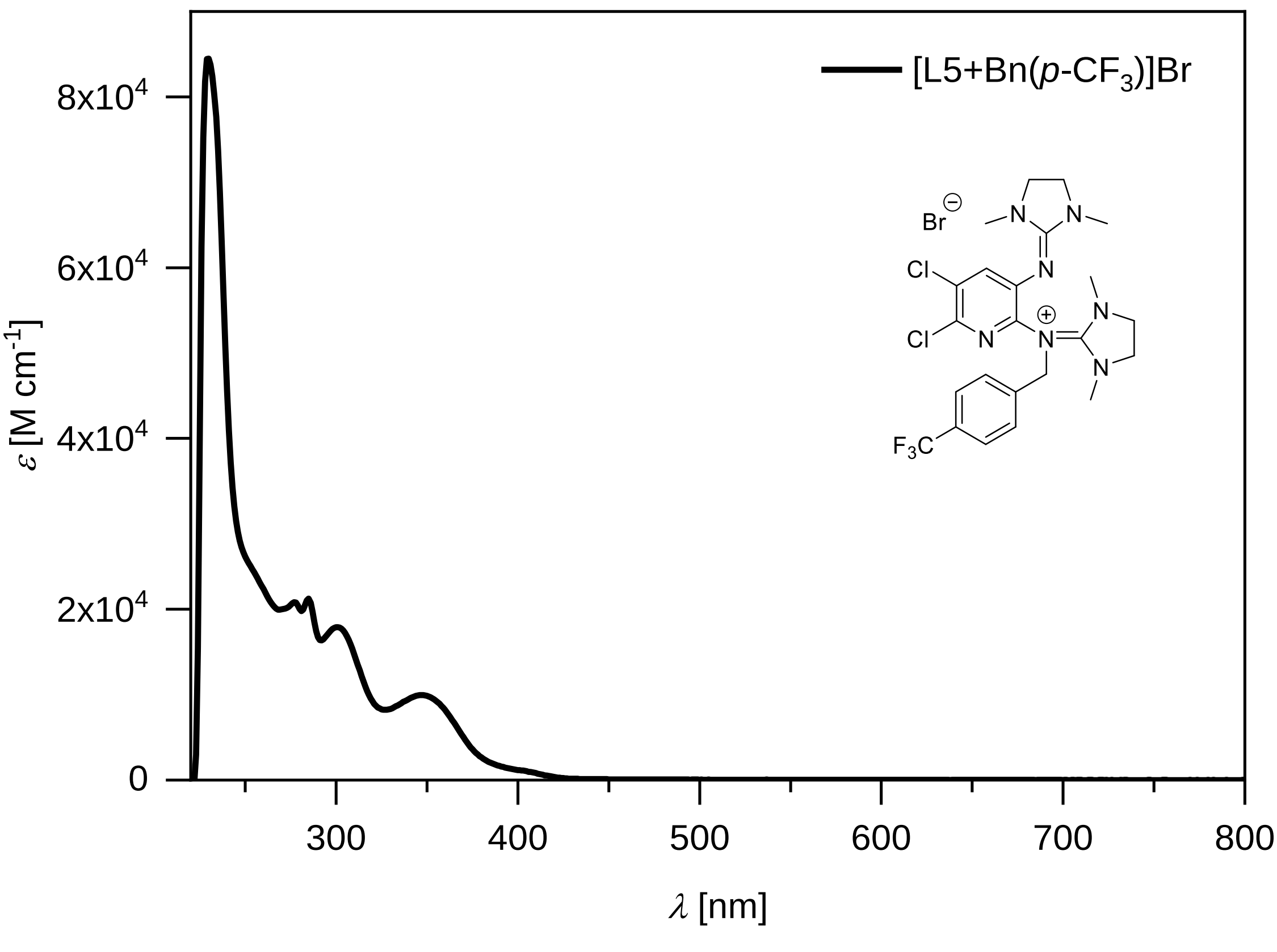

Figure S104: UV-vis absorption spectrum of [L5a+Bn^{b}(*p*-CF_3)]Br in dichloromethane at a concentration of $2.20 \cdot 10^{-5}$ mol L^{-1} at r.t.

2.3.16 [L6+Bn^b(*p*-CF3)]Br

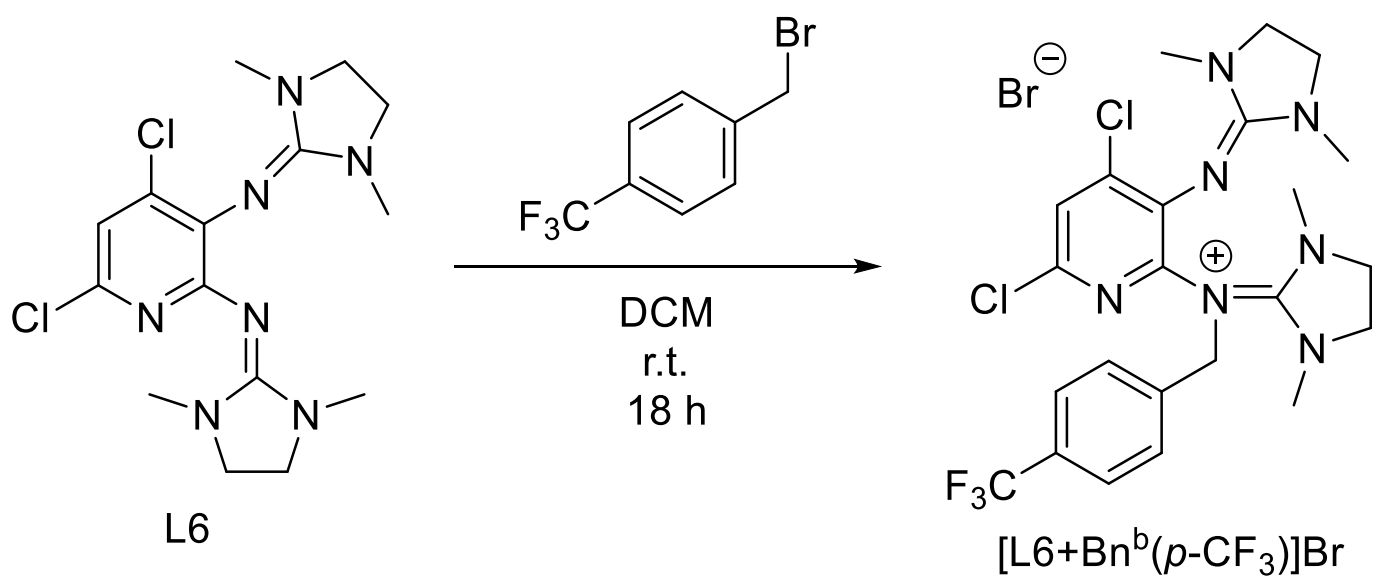

Following general procedure 2, L6 (60.0 mg, 162 μmol, 1.00 eq.) and 4-(trifluoromethyl)benzylbromide (79.0 mg, 331 μmol, 2.04 eq.) were used to obtain [L6+Bn^b(*p*-CF_3)]Br (85.0 mg, 161 mmol, 99%) as a yellow-brown solid. For purification, the crude product was irradiated with visible light for 2 h.

A Crystal for single crystal structure analysis was obtained from an acetonitrile solution by layering with diethyl ether.

^{1}H NMR (400 MHz, CD_3CN)

δ = 7.71 – 7.66 (m, 4H, CH[1]), 7.22 (s, 1H, CH), 5.06 (s, 2H, CH_2), 3.77 (s, 4H, CH_2), 3.45 – 3.33 (m, 4H, CH_2), 2.71 (s, 6H, CH_3), 2.61 (s, 6H, CH_3) ppm.

^{13}C NMR (101 MHz, CD_3CN), **^{13}C-DEPT-135**, **HSQC**, **HMBC**

δ = 161.1 (1C, C_q), 157.7 (1C, C_q), 146.85 (1C, C_q), 141.7 (1C, C_q), 141.2 (1C, C_q), 130.4 (2C, CH), 126.6 (2C, CH), 121.3 (1C, CH), 53.48 (1C, CH_2), 49.2 (2C, CH_2), 48.6 (2C, CH_2), 34.8 (2C, CH_3), 34.1 (2C, CH_3) ppm.

3 signals are coincident or not observed.

^{19}F NMR (376 MHz, CD_3CN)

δ = −63.0 (s, 3F) ppm.

UV-vis (DCM) ($1.50{\cdot}10^{-5}$ mol L^{-1})

λ (*ε*) = 332 ‘(8590), 284 (21100), 277 (21900), 229 (85600) nm (M^{-1} cm^{-1}).

Mass spectrometry (ESI^+ in DCM)

m/z = 528.1639 ([L6+Bn^b(*p*-CF_3)]$^+$, calc. 528.1652).

Elemental analysis (C, H, N in %) ($C_{23}H_{27}BrCl_2F_3N_7$)

calc.: C 45.34, H 4.47, N 16.09.

found: C 47.75, H 6.39, N 15.18.

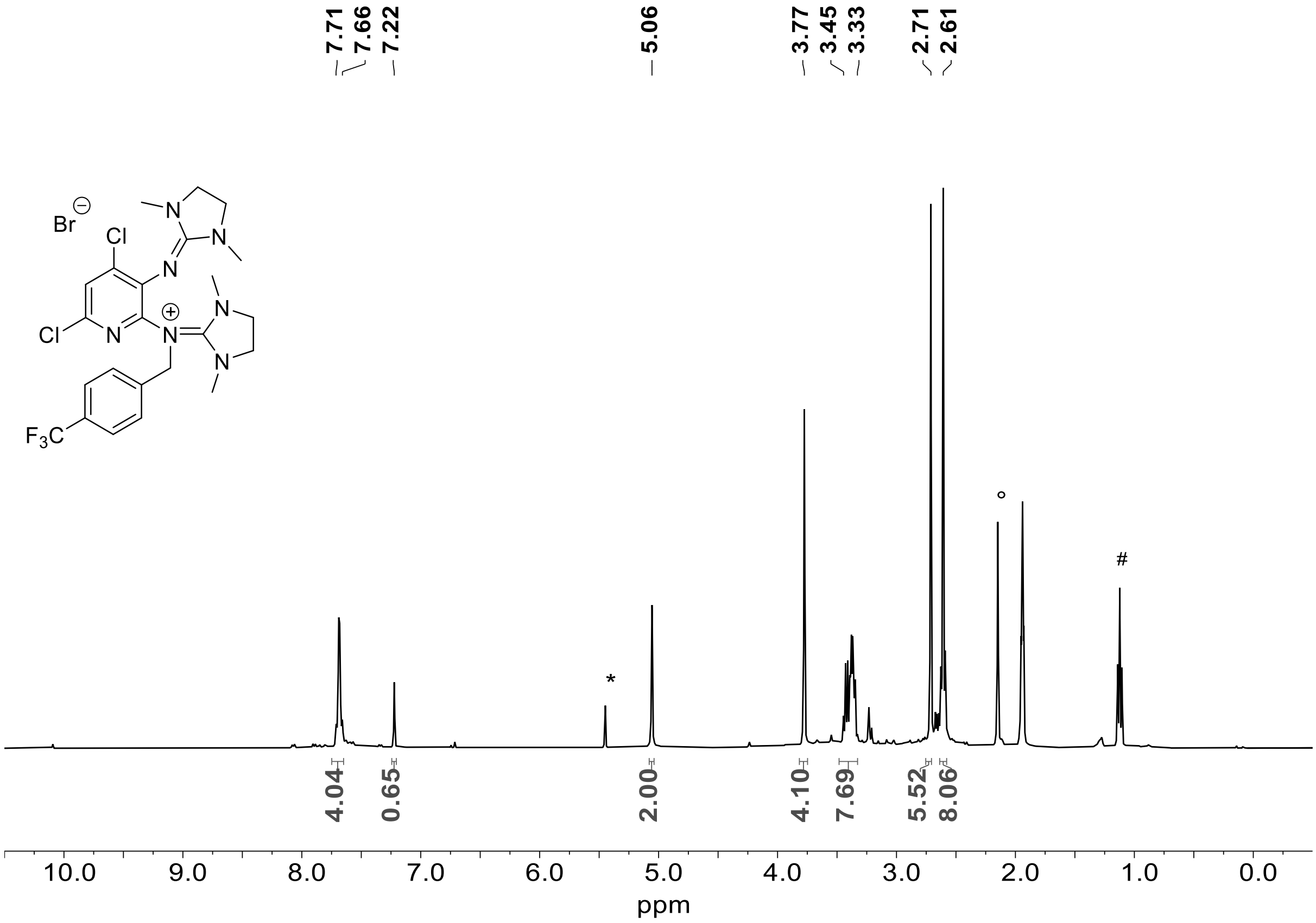

Figure S105: ^{1}H NMR spectrum of [L6+Bn^{b}(*p*-CF$_3$)]Br measured at 400 MHz in CD$_3$CN, *DCM, °water, #diethyl ether.

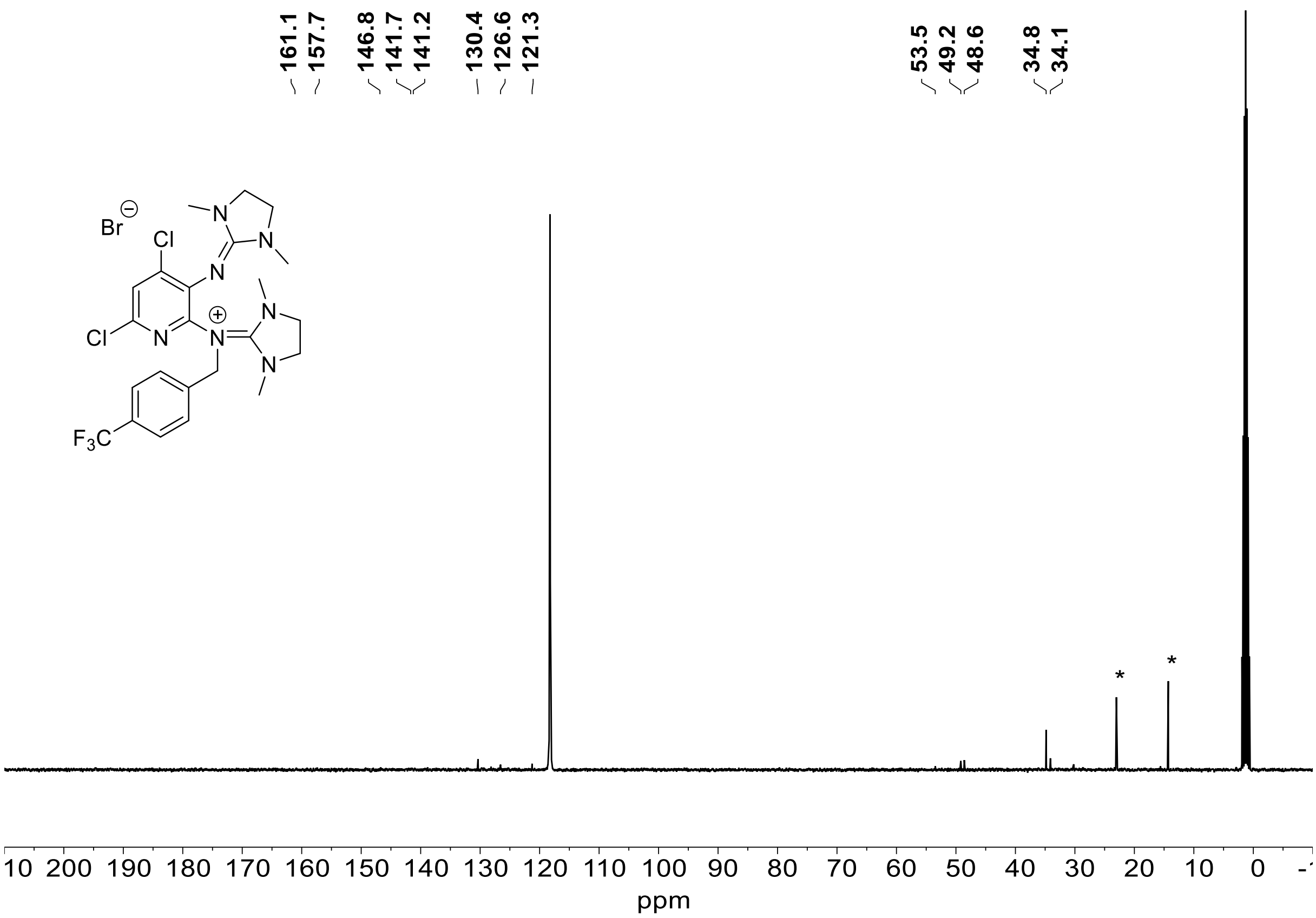

Figure S106: ^{13}C NMR spectrum of [L6+Bn^{b}(*p*-CF$_3$)]Br, measured at 101 MHz in CD$_3$CN, *pentane.

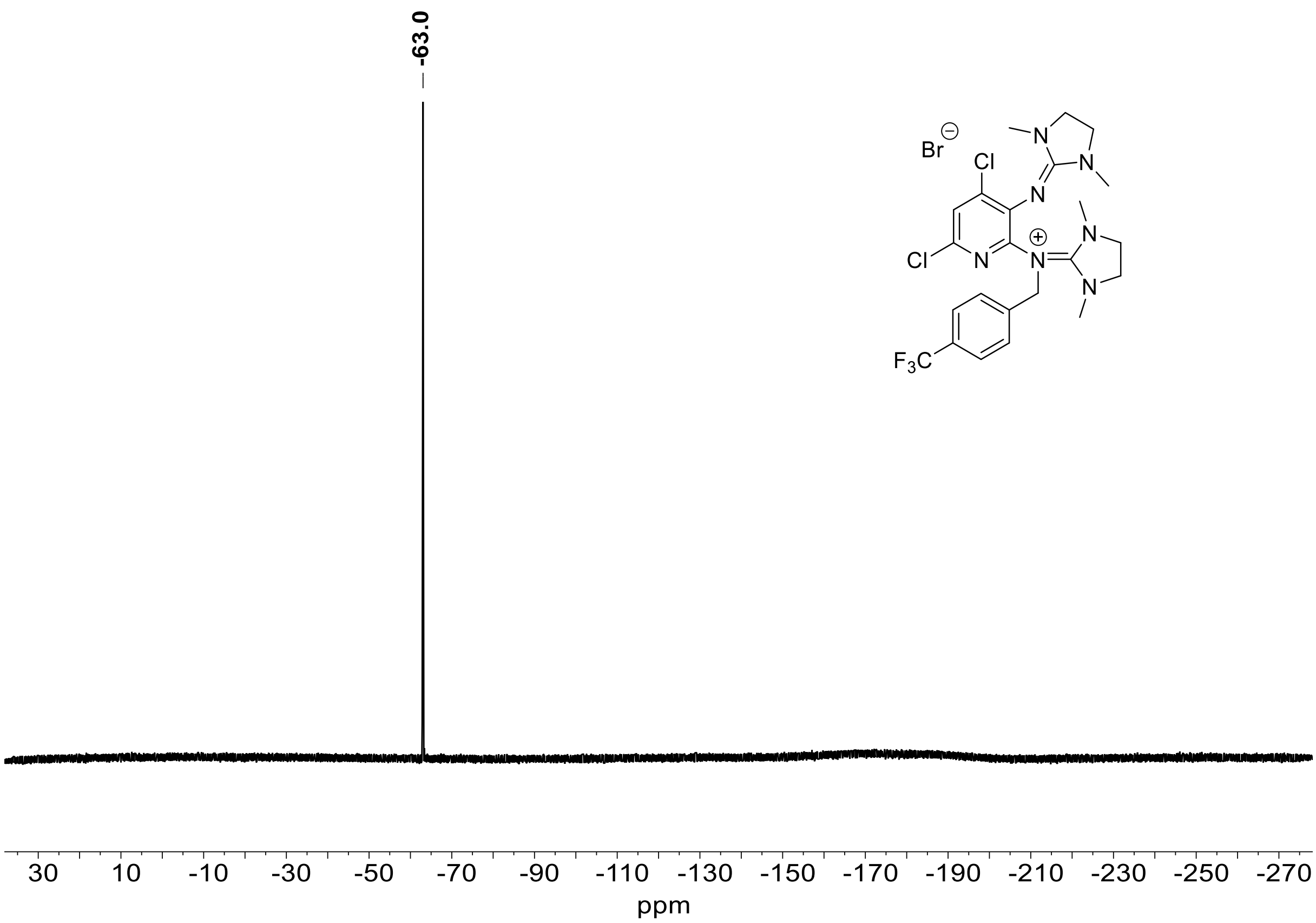

Figure S107: ^{19}F NMR spectrum of [L6+Bn(p-CF_3)]Br, measured at 367 MHz in CD_3CN.

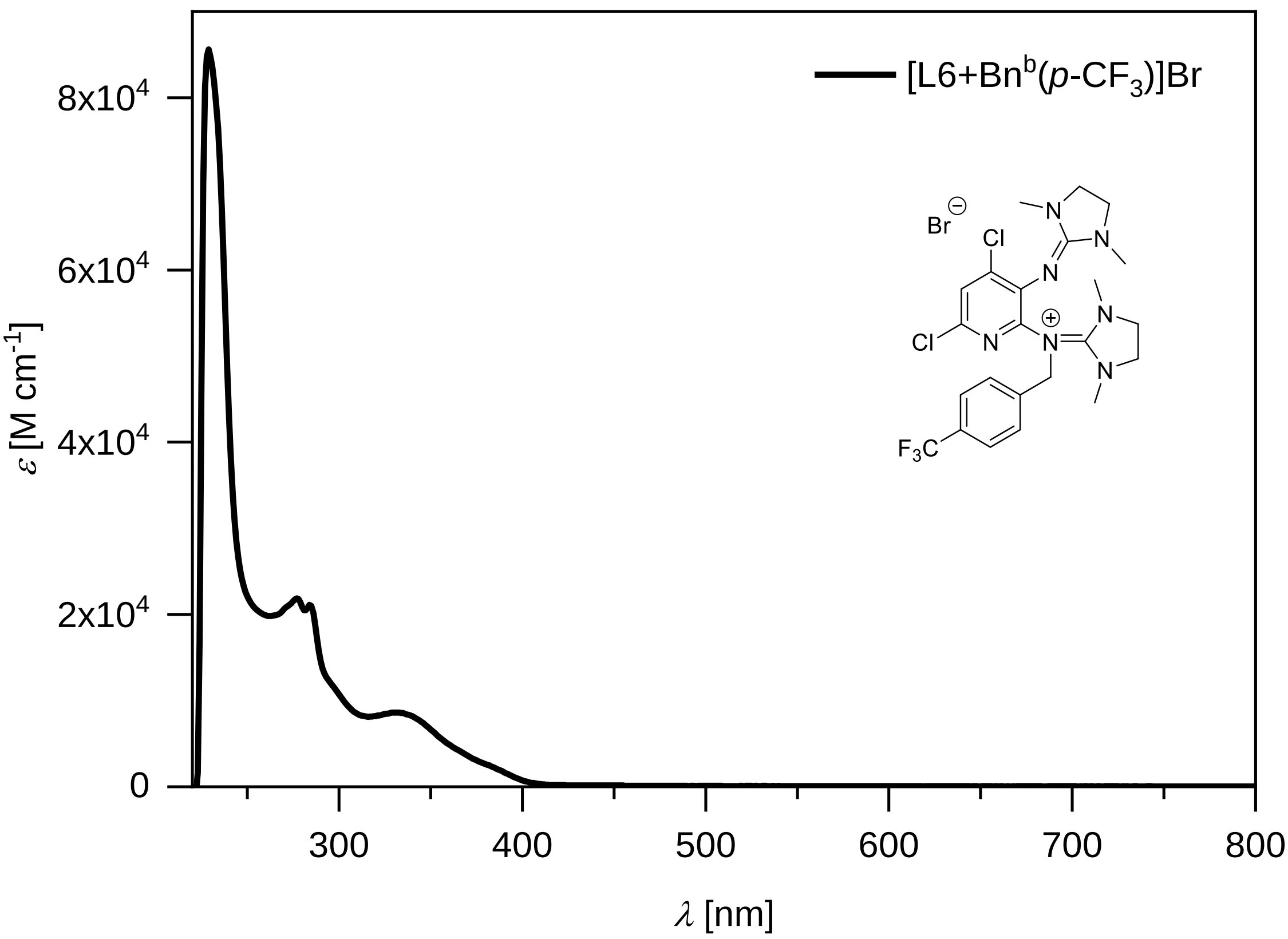

Figure S108: UV-vis absorption spectrum of [L6+Bn^b(p-CF_3)]Br in dichloromethane at a concentration of $1.50 \cdot 10^{-5}$ mol L^{-1} at r.t.

3. Crystal structures

3.1 Ligands

3.1.1 L2a

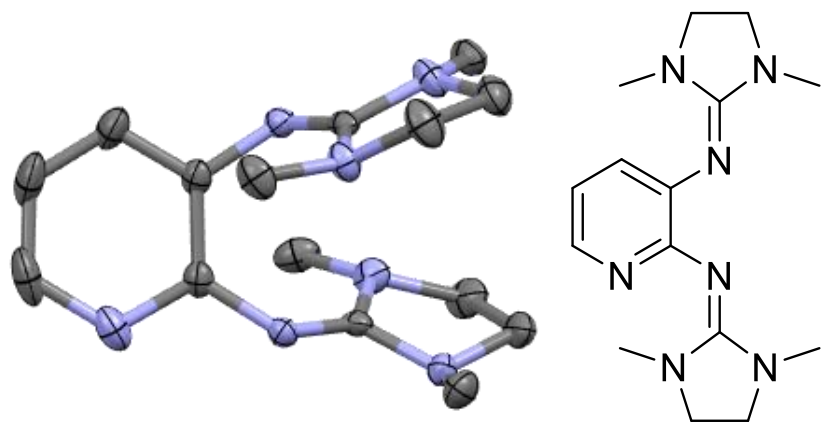

Table S1: Crystal data and structure refinement for L2a.

Identification code	mo_hk10_0ma
Empirical formula	$C_{15}H_{23}N_7$
Formula weight	301.40
Temperature/K	100.00
Crystal system	monoclinic
Space group	$P2_1/c$
a/Å	12.7616(11)
b/Å	9.2834(8)
c/Å	14.5260(12)
α/°	90
β/°	111.216(3)
γ/°	90
Volume/Å^3	1604.3(2)
Z	4
ρ_{calc}g/cm^3	1.248
μ/mm^{-1}	0.081
F(000)	648.0
Crystal size/mm^3	0.65 × 0.42 × 0.2
Radiation	MoKα (λ = 0.71073)
2Θ range for data collection/°	5.32 to 53
Index ranges	-16 ≤ h ≤ 16, -11 ≤ k ≤ 11, -18 ≤ l ≤ 18
Reflections collected	54622
Independent reflections	3333 [R_{int} = 0.1038, R_{sigma} = 0.0376]
Data/restraints/parameters	3333/0/203
Goodness-of-fit on F^2	1.059
Final R indexes [I>=2σ (I)]	R_1 = 0.0463, wR_2 = 0.1057
Final R indexes [all data]	R_1 = 0.0594, wR_2 = 0.1170
Largest diff. peak/hole / e Å^{-3}	0.24/-0.24

3.1.2 (L2b+2H)$(BF_4)_2$

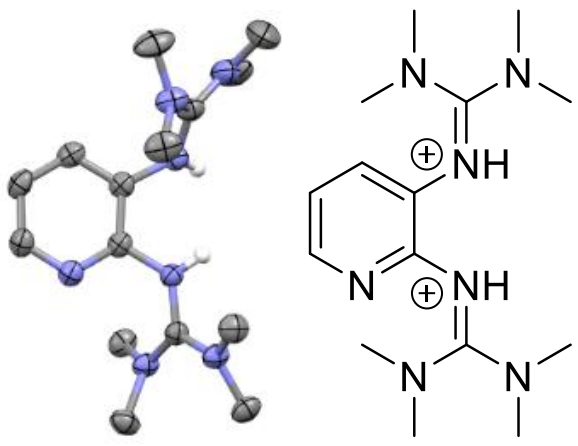

Table S2: Crystal data and structure refinement for $(L2bH_2)(BF_4)_2$.

Identification code	mo_2024_hk30_0ma
Empirical formula	$C_{15}H_{29}B_2F_8N_7$
Formula weight	481.07
Temperature/K	100.00
Crystal system	monoclinic
Space group	$P2_1/n$
a/Å	11.1540(16)
b/Å	16.515(2)
c/Å	12.4541(18)
α/°	90
β/°	100.676(4)
γ/°	90
Volume/Å^3	2254.4(6)
Z	4
ρ_{calc}g/cm^3	1.417
μ/mm^{-1}	0.134
F(000)	1000.0
Crystal size/mm^3	0.428 × 0.113 × 0.098
Radiation	MoKα (λ = 0.71073)
2Θ range for data collection/°	4.142 to 53.998
Index ranges	-14 ≤ h ≤ 14, -21 ≤ k ≤ 21, -15 ≤ l ≤ 15
Reflections collected	65034
Independent reflections	4916 [R_{int} = 0.1100, R_{sigma} = 0.0407]
Data/restraints/parameters	4916/27/344
Goodness-of-fit on F^2	1.041
Final R indexes [I>=2σ (I)]	R_1 = 0.0647, wR_2 = 0.1504
Final R indexes [all data]	R_1 = 0.0990, wR_2 = 0.1720
Largest diff. peak/hole / e Å^{-3}	0.39/-0.35

3.1.3 L3

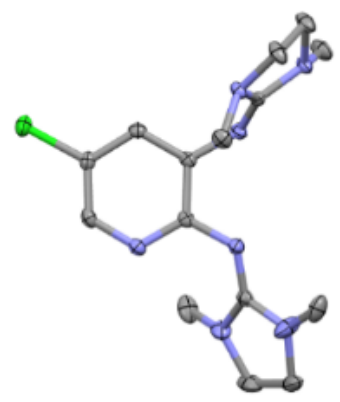

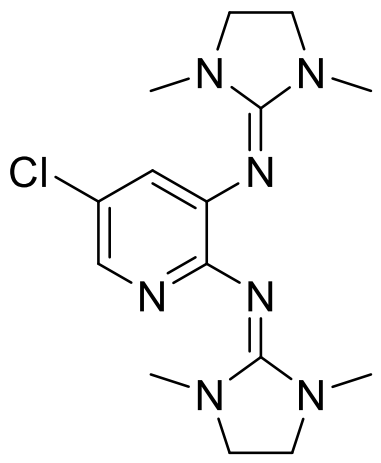

Table S3: Crystal data and structure refinement for L3.

Identification code	mo_2025_hkee23_4_0m
Empirical formula	$C_{15}H_{22}ClN_7$
Formula weight	335.84
Temperature/K	100.00
Crystal system	triclinic
Space group	P-1
a/Å	7.9146(9)
b/Å	14.0554(14)
c/Å	16.1704(15)
α/°	79.538(4)
β/°	82.035(4)
γ/°	74.572(4)
Volume/Å^3	1697.4(3)
Z	4
ρ_{calc}g/cm^3	1.314
μ/mm^{-1}	0.236
F(000)	712.0
Crystal size/mm^3	0.35 × 0.27 × 0.13
Radiation	MoKα (λ = 0.71073)
2Θ range for data collection/°	3.674 to 56
Index ranges	-10 ≤ h ≤ 10, -18 ≤ k ≤ 18, -21 ≤ l ≤ 21
Reflections collected	73685
Independent reflections	8205 [R_{int} = 0.0672, R_{sigma} = 0.0368]
Data/restraints/parameters	8205/0/423
Goodness-of-fit on F^2	1.037
Final R indexes [I>=2σ (I)]	R_1 = 0.0401, wR_2 = 0.0999
Final R indexes [all data]	R_1 = 0.0454, wR_2 = 0.1049
Largest diff. peak/hole / e Å^{-3}	0.30/-0.36

3.1.4 L4

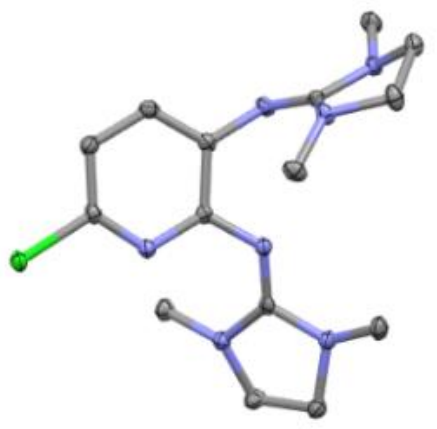

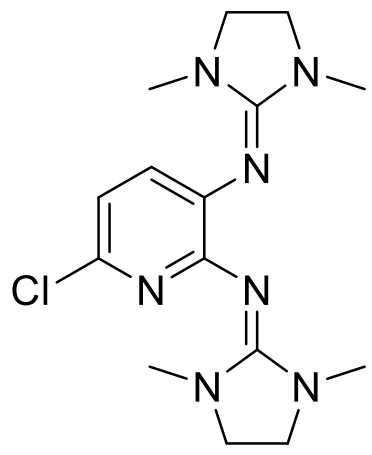

Table S4: Crystal data and structure refinement for L4.

Identification code	mo_2026_hk242_2_0ma
Empirical formula	$C_{15}H_{22}ClN_7$
Formula weight	335.84
Temperature/K	100.00
Crystal system	monoclinic
Space group	C2/c
a/Å	25.0491(17)
b/Å	8.3939(6)
c/Å	16.3393(12)
α/°	90
β/°	107.013(5)
γ/°	90
Volume/$Å^3$	3285.2(4)
Z	8
ρ_{calc}g/cm^3	1.358
μ/mm^{-1}	0.244
F(000)	1424.0
Crystal size/mm^3	0.27 × 0.24 × 0.1
Radiation	MoKα (λ = 0.71073)
2Θ range for data collection/°	5.142 to 57.644
Index ranges	-33 ≤ h ≤ 33, -11 ≤ k ≤ 11, -22 ≤ l ≤ 22
Reflections collected	51160
Independent reflections	4279 [R_{int} = 0.0849, R_{sigma} = 0.0325]
Data/restraints/parameters	4279/0/212
Goodness-of-fit on F^2	1.051
Final R indexes [I>=2σ (I)]	R_1 = 0.0352, wR_2 = 0.0833
Final R indexes [all data]	R_1 = 0.0419, wR_2 = 0.0883
Largest diff. peak/hole / e $Å^{-3}$	0.34/-0.27

3.1.5 L5a

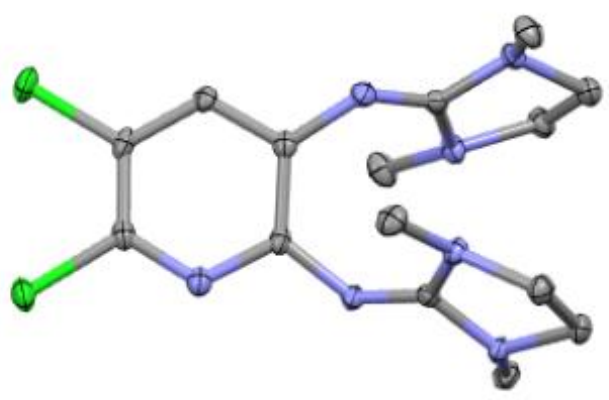

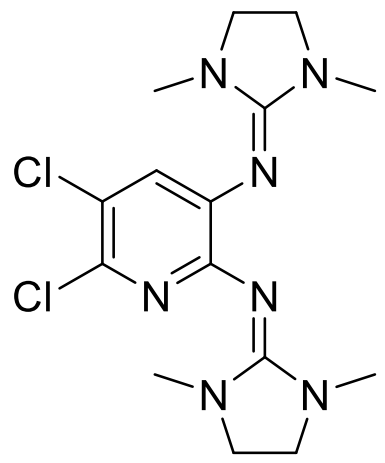

Table S5: Crystal data and structure refinement for L5a.

Identification code	mo_2026_hk245_0m
Empirical formula	$C_{15}H_{21}Cl_2N_7$
Formula weight	370.29
Temperature/K	100.00
Crystal system	monoclinic
Space group	$P2_1/c$
a/Å	10.2278(5)
b/Å	11.0979(6)
c/Å	15.3342(8)
α/°	90
β/°	104.292(2)
γ/°	90
Volume/Å^3	1686.67(15)
Z	4
ρ_{calc}g/cm^3	1.458
μ/mm^{-1}	0.398
F(000)	776.0
Crystal size/mm^3	0.21 × 0.18 × 0.12
Radiation	MoKα (λ = 0.71073)
2Θ range for data collection/°	4.11 to 54.334
Index ranges	-13 ≤ h ≤ 13, -14 ≤ k ≤ 14, -19 ≤ l ≤ 19
Reflections collected	118976
Independent reflections	3750 [R_{int} = 0.0770, R_{sigma} = 0.0171]
Data/restraints/parameters	3750/0/221
Goodness-of-fit on F^2	1.050
Final R indexes [I>=2σ (I)]	R_1 = 0.0304, wR_2 = 0.0734
Final R indexes [all data]	R_1 = 0.0346, wR_2 = 0.0759
Largest diff. peak/hole / e Å^{-3}	0.34/-0.28

3.1.6 L5b

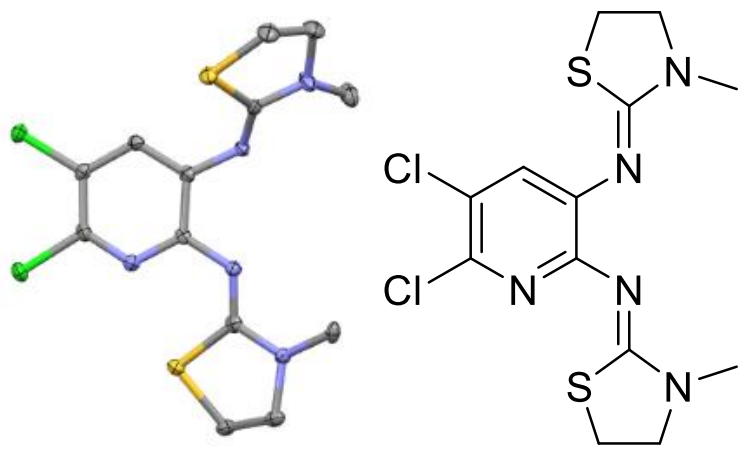

Table S6: Crystal data and structure refinement for L5b.

Identification code	mo_2026_hk227_3_0m
Empirical formula	$C_{13}H_{15}Cl_2N_5S_2$
Formula weight	376.32
Temperature/K	100.00
Crystal system	orthorhombic
Space group	$P2_12_12_1$
a/Å	8.1640(7)
b/Å	9.5212(7)
c/Å	20.4100(14)
α/°	90
β/°	90
γ/°	90
Volume/Å^3	1586.5(2)
Z	4
ρ_{calc}g/cm^3	1.576
μ/mm^{-1}	0.675
F(000)	776.0
Crystal size/mm^3	0.25 × 0.11 × 0.06
Radiation	MoKα (λ = 0.71073)
2Θ range for data collection/°	4.72 to 55.224
Index ranges	-10 ≤ h ≤ 10, -12 ≤ k ≤ 12, -26 ≤ l ≤ 25
Reflections collected	41596
Independent reflections	3692 [R_{int} = 0.0871, R_{sigma} = 0.0350]
Data/restraints/parameters	3692/0/201
Goodness-of-fit on F^2	1.070
Final R indexes [I>=2σ (I)]	R_1 = 0.0277, wR_2 = 0.0664
Final R indexes [all data]	R_1 = 0.0296, wR_2 = 0.0682
Largest diff. peak/hole / e Å^{-3}	0.44/-0.22

3.1.7 L6

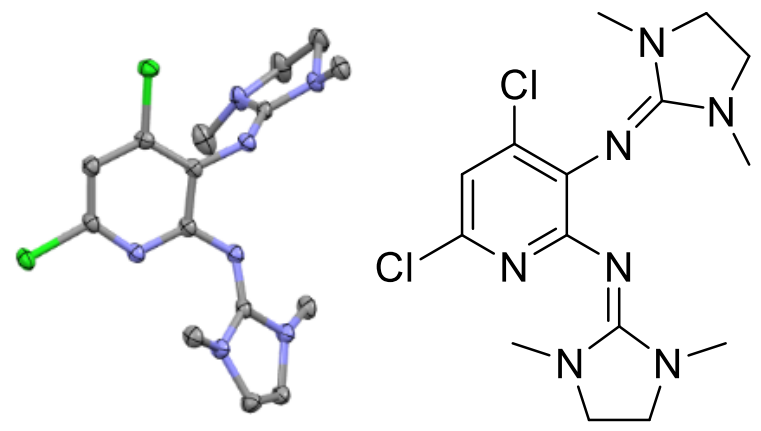

Table S7: Crystal data and structure refinement for L6.

Identification code	mo_hk243_0ma
Empirical formula	$C_{15}H_{21}Cl_2N_7$
Formula weight	370.29
Temperature/K	100.00
Crystal system	monoclinic
Space group	C2/c
a/Å	25.495(3)
b/Å	8.4848(10)
c/Å	16.7739(19)
α/°	90
β/°	102.480(7)
γ/°	90
Volume/Å^3	3542.8(7)
Z	8
ρ_{calc}g/cm^3	1.388
μ/mm^{-1}	0.379
F(000)	1552.0
Crystal size/mm^3	0.23 × 0.06 × 0.04
Radiation	MoKα (λ = 0.71073)
2Θ range for data collection/°	4.974 to 54.48
Index ranges	-32 ≤ h ≤ 32, -10 ≤ k ≤ 10, -21 ≤ l ≤ 21
Reflections collected	84624
Independent reflections	3949 [R_{int} = 0.0712, R_{sigma} = 0.0235]
Data/restraints/parameters	3949/0/221
Goodness-of-fit on F^2	1.052
Final R indexes [I>=2σ (I)]	R_1 = 0.0344, wR_2 = 0.0834
Final R indexes [all data]	R_1 = 0.0435, wR_2 = 0.0893
Largest diff. peak/hole / e Å^{-3}	0.30/-0.47

3.1.8 $(L2b_2{+}2H)(PF_6)_4$

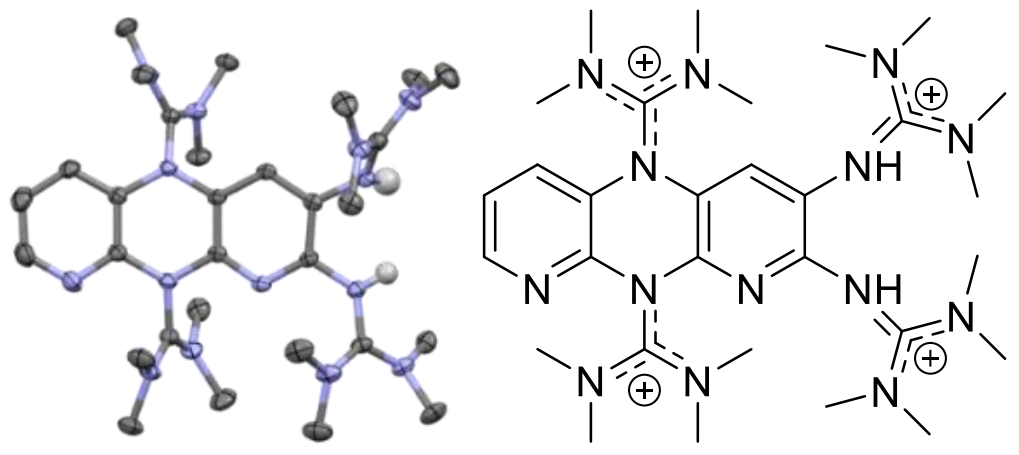

Table S8: Crystal data and structure refinement for $(L2b_2{+}2H)(PF_6)_4$.

Identification code	mo_2024_hk28_0m
Empirical formula	$C_{34}H_{60}F_{24}N_{16}P_4$
Formula weight	1272.86
Temperature/K	100.00
Crystal system	triclinic
Space group	P-1
a/Å	8.3298(10)
b/Å	13.3753(15)
c/Å	25.376(3)
α/°	84.826(4)
β/°	85.426(4)
γ/°	71.865(4)
Volume/Å^3	2671.8(6)
Z	2
ρ_{calc}g/cm^3	1.582
μ/mm^{-1}	0.271
F(000)	1304.0
Crystal size/mm^3	0.45 × 0.14 × 0.13
Radiation	MoKα (λ = 0.71073)
2Θ range for data collection/°	4.394 to 52.998
Index ranges	-10 ≤ h ≤ 10, -16 ≤ k ≤ 16, -31 ≤ l ≤ 31
Reflections collected	84976
Independent reflections	11083 [R_{int} = 0.0846, R_{sigma} = 0.0488]
Data/restraints/parameters	11083/304/764
Goodness-of-fit on F^2	1.030
Final R indexes [I>=2σ (I)]	R_1 = 0.0594, wR_2 = 0.1551
Final R indexes [all data]	R_1 = 0.0761, wR_2 = 0.1702
Largest diff. peak/hole / e Å^{-3}	1.08/-0.65

3.1.9 $(L2a_2)(PF_6)_2$

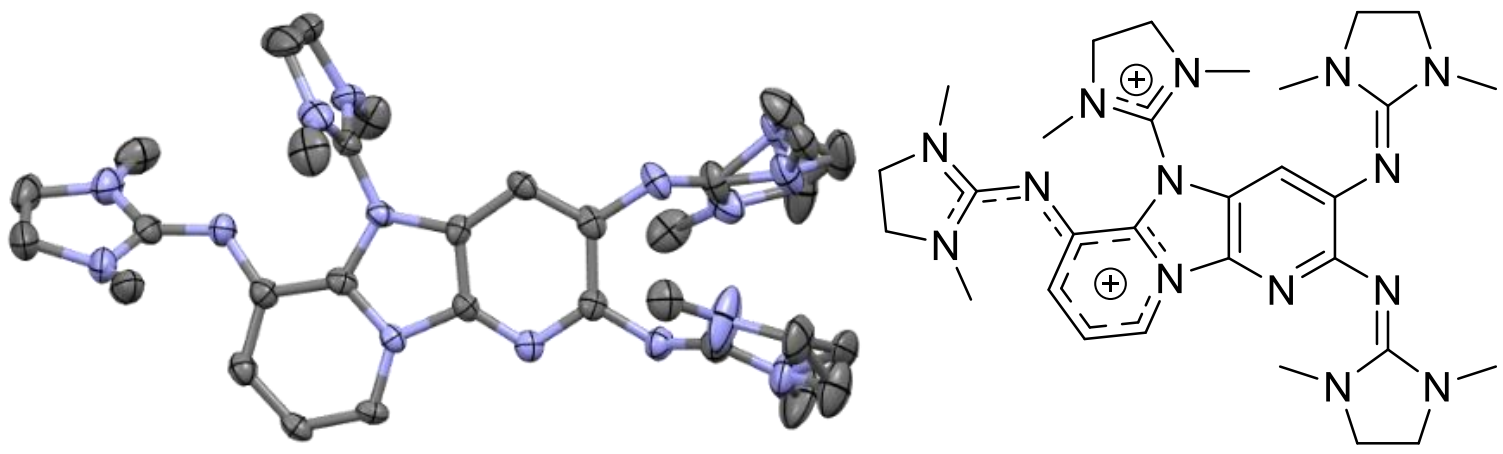

Table S9: Crystal data and structure refinement for $(L2a_2)(PF_6)_2$.

Identification code	mo_2024_hk50_1_0m
Empirical formula	$C_{32}H_{47}F_{12}N_{15}P_2$
Formula weight	931.78
Temperature/K	100.00
Crystal system	triclinic
Space group	P-1
a/Å	8.5277(11)
b/Å	15.500(2)
c/Å	17.5677(19)
α/°	111.405(4)
β/°	101.513(5)
γ/°	96.349(5)
Volume/Å^3	2075.2(4)
Z	2
ρ_{calc}g/cm^3	1.491
μ/mm^{-1}	0.205
F(000)	964.0
Crystal size/mm^3	0.085 × 0.083 × 0.05
Radiation	MoKα (λ = 0.71073)
2Θ range for data collection/°	4.566 to 51.998
Index ranges	-10 ≤ h ≤ 10, -19 ≤ k ≤ 19, -21 ≤ l ≤ 21
Reflections collected	58583
Independent reflections	8153 [R_{int} = 0.1837, R_{sigma} = 0.1166]
Data/restraints/parameters	8153/212/659
Goodness-of-fit on F^2	1.006
Final R indexes [I>=2σ (I)]	R_1 = 0.0680, wR_2 = 0.1534
Final R indexes [all data]	R_1 = 0.1432, wR_2 = 0.2027
Largest diff. peak/hole / e Å^{-3}	0.34/-0.48

3.2 Complexes

3.2.1 [$NiCl_2$(L2a)]

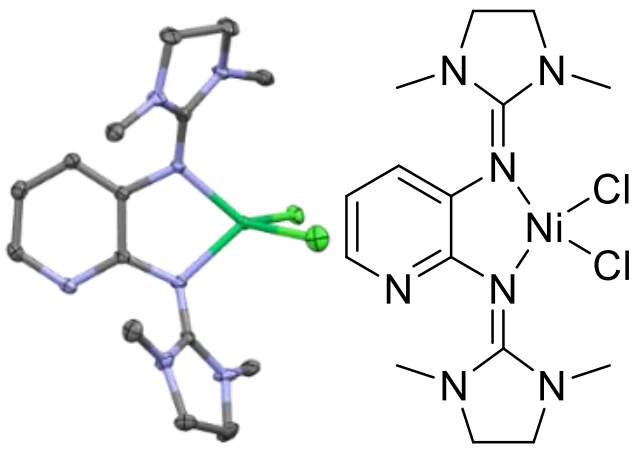

Table S10: Crystal data and structure refinement for [$NiCl_2$(L2a)].

Identification code	mo_hk44_0ma
Empirical formula	$C_{15}H_{23}N_7Cl_2Ni$
Formula weight	431.01
Temperature/K	100.00
Crystal system	monoclinic
Space group	$P2_1/c$
a/Å	11.4792(7)
b/Å	10.0071(7)
c/Å	16.3113(11)
α/°	90
β/°	91.605(2)
γ/°	90
Volume/Å^3	1873.0(2)
Z	4
ρ_{calc}g/cm^3	1.528
μ/mm^{-1}	1.335
F(000)	896.0
Crystal size/mm^3	0.44 × 0.38 × 0.37
Radiation	MoKα (λ = 0.71073)
2Θ range for data collection/°	4.776 to 56.734
Index ranges	-15 ≤ h ≤ 15, -13 ≤ k ≤ 13, -21 ≤ l ≤ 21
Reflections collected	98402
Independent reflections	4690 [R_{int} = 0.0535, R_{sigma} = 0.0203]
Data/restraints/parameters	4690/0/231
Goodness-of-fit on F^2	1.043
Final R indexes [I>=2σ (I)]	R_1 = 0.0329, wR_2 = 0.0883
Final R indexes [all data]	R_1 = 0.0358, wR_2 = 0.0904
Largest diff. peak/hole / e Å^{-3}	0.61/-0.42

3.2.2 [$CoCl_2$(L2a)]

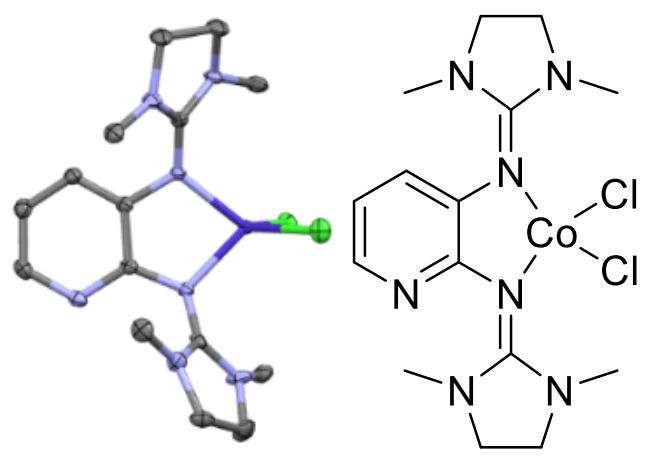

Table S11: Crystal data and structure refinement for [$CoCl_2$(L2a)].

Identification code	mo_2024_hk43_0ma
Empirical formula	$C_{15}H_{23}Cl_2CoN_7$
Formula weight	431.23
Temperature/K	100.00
Crystal system	monoclinic
Space group	$P2_1/c$
a/Å	11.5576(5)
b/Å	10.0180(4)
c/Å	16.3338(7)
α/°	90
β/°	91.974(2)
γ/°	90
Volume/Å^3	1890.07(14)
Z	4
ρ_{calc}g/cm^3	1.515
μ/mm^{-1}	1.204
F(000)	892.0
Crystal size/mm^3	0.5 × 0.24 × 0.17
Radiation	MoKα (λ = 0.71073)
2Θ range for data collection/°	4.77 to 55.994
Index ranges	-15 ≤ h ≤ 15, -13 ≤ k ≤ 13, -21 ≤ l ≤ 21
Reflections collected	116196
Independent reflections	4554 [R_{int} = 0.0680, R_{sigma} = 0.0228]
Data/restraints/parameters	4554/0/231
Goodness-of-fit on F^2	1.030
Final R indexes [I>=2σ (I)]	R_1 = 0.0332, wR_2 = 0.0836
Final R indexes [all data]	R_1 = 0.0380, wR_2 = 0.0867
Largest diff. peak/hole / e Å^{-3}	0.54/-0.38

3.2.3 [$CoBr_2$(L2a)]

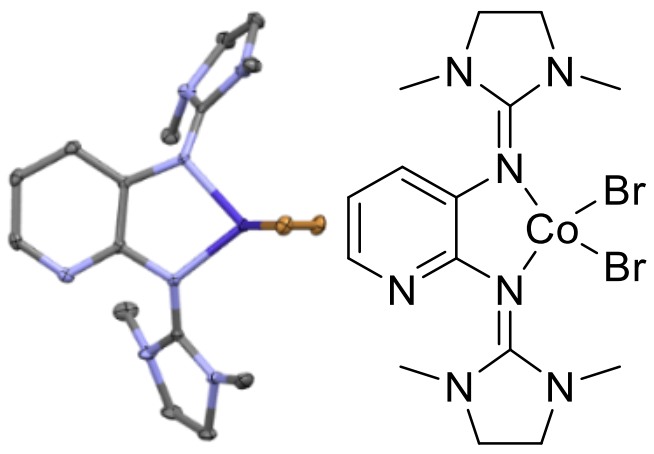

Table S12: Crystal data and structure refinement for [$CoBr_2$(L2a)].

Identification code	mo_2024_hk42_0ma
Empirical formula	$C_{15}H_{23}N_7CoBr_2$
Formula weight	520.15
Temperature/K	100.00
Crystal system	orthorhombic
Space group	Pbca
a/Å	16.3889(8)
b/Å	14.6737(9)
c/Å	16.4120(10)
α/°	90
β/°	90
γ/°	90
Volume/Å^3	3946.9(4)
Z	8
ρ_{calc}g/cm^3	1.751
μ/mm^{-1}	4.932
F(000)	2072.0
Crystal size/mm^3	0.29 × 0.12 × 0.1
Radiation	MoKα (λ = 0.71073)
2Θ range for data collection/°	4.476 to 52.994
Index ranges	-20 ≤ h ≤ 20, -18 ≤ k ≤ 18, -20 ≤ l ≤ 20
Reflections collected	144994
Independent reflections	4093 [R_{int} = 0.1117, R_{sigma} = 0.0323]
Data/restraints/parameters	4093/0/230
Goodness-of-fit on F^2	1.046
Final R indexes [I>=2σ (I)]	R_1 = 0.0281, wR_2 = 0.0683
Final R indexes [all data]	R_1 = 0.0360, wR_2 = 0.0747
Largest diff. peak/hole / e Å^{-3}	0.71/-0.50

3.2.4 [Co(hfac)$_2$(L2a)]

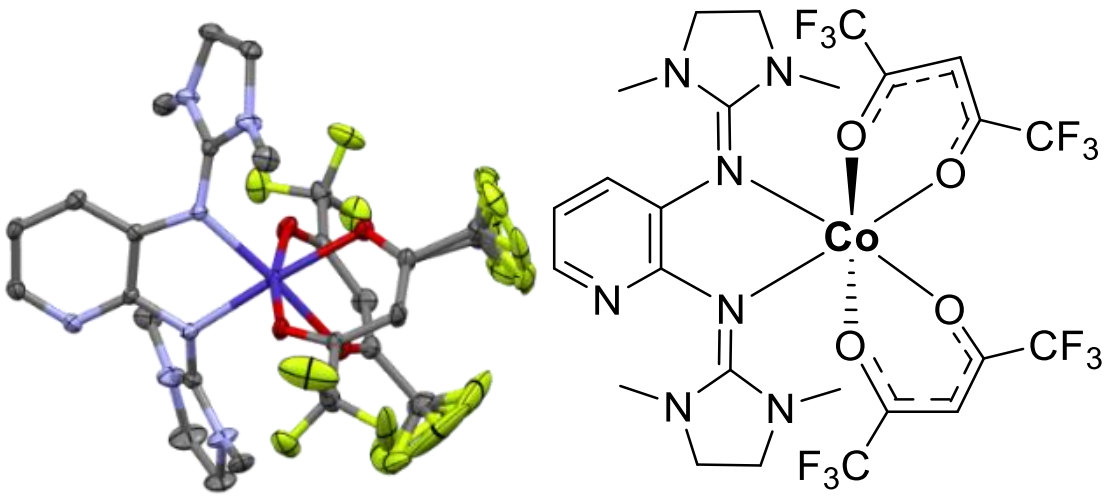

Table S13: Crystal data and structure refinement for [Co(hfac)$_2$(L2a)].

Identification code	mo_2024_hk46_01
Empirical formula	$C_{25}H_{25}CoF_{12}N_7O_4$
Formula weight	774.45
Temperature/K	100.00
Crystal system	orthorhombic
Space group	Pbca
a/Å	9.6998(4)
b/Å	21.7514(12)
c/Å	30.1963(17)
α/°	90
β/°	90
γ/°	90
Volume/Å^3	6370.9(6)
Z	8
ρ_{calc}g/cm^3	1.615
μ/mm^{-1}	0.653
F(000)	3128.0
Crystal size/mm^3	0.266 × 0.117 × 0.097
Radiation	MoKα (λ = 0.71073)
2Θ range for data collection/°	3.98 to 52.904
Index ranges	-12 ≤ h ≤ 12, -27 ≤ k ≤ 27, -37 ≤ l ≤ 37
Reflections collected	167507
Independent reflections	6562 [R_{int} = 0.1359, R_{sigma} = 0.0432]
Data/restraints/parameters	6562/264/536
Goodness-of-fit on F^2	1.038
Final R indexes [I>=2σ (I)]	R_1 = 0.0472, wR_2 = 0.1010
Final R indexes [all data]	R_1 = 0.0655, wR_2 = 0.1148
Largest diff. peak/hole / e Å^{-3}	0.77/-0.63

3.2.5 [$CuBr_2$(L2a)]

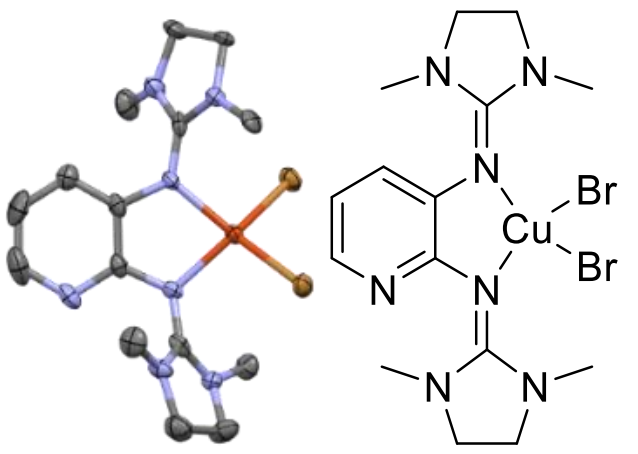

Table S14: Crystal data and structure refinement for [$CuBr_2$(L2a)].

Identification code	mo_2024_hk41_0ma
Empirical formula	$C_{15}H_{23}Br_2CuN_7$
Formula weight	524.76
Temperature/K	100.00
Crystal system	monoclinic
Space group	$P2_1$
a/Å	10.358(6)
b/Å	7.958(4)
c/Å	12.256(7)
α/°	90
β/°	99.241(12)
γ/°	90
Volume/Å^3	997.2(10)
Z	2
ρ_{calc}g/cm^3	1.748
μ/mm^{-1}	5.115
F(000)	522.0
Crystal size/mm^3	0.25 × 0.085 × 0.035
Radiation	MoKα (λ = 0.71073)
2Θ range for data collection/°	3.984 to 51.99
Index ranges	-12 ≤ h ≤ 12, -9 ≤ k ≤ 9, -15 ≤ l ≤ 15
Reflections collected	18634
Independent reflections	3902 [R_{int} = 0.1160, R_{sigma} = 0.0912]
Data/restraints/parameters	3902/1/231
Goodness-of-fit on F^2	1.068
Final R indexes [I>=2σ (I)]	R_1 = 0.0572, wR_2 = 0.1234
Final R indexes [all data]	R_1 = 0.0815, wR_2 = 0.1369
Largest diff. peak/hole / e Å^{-3}	1.05/-0.70

3.2.6 $[Cu(L2a)_2](BF_4)_2$

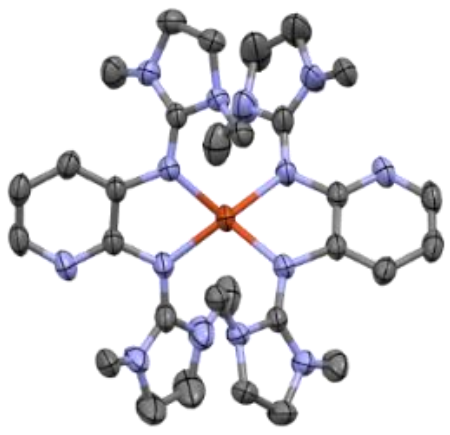
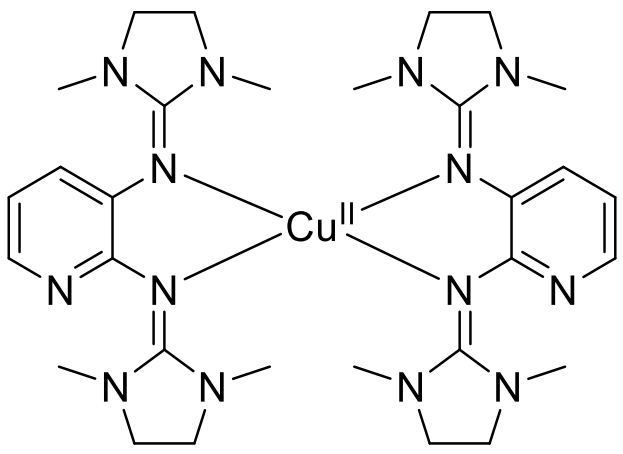

Table S15: Crystal data and structure refinement for $[Cu(L2a)_2](BF_4)_2$.

Identification code	mo_2024_hk48_01a
Empirical formula	$C_{30}H_{46}B_2CuF_8N_{14}$ + 2.4 · CH_2Cl_2
Formula weight	1043.79
Temperature/K	100.00
Crystal system	monoclinic
Space group	C2/c
a/Å	21.2968(16)
b/Å	17.0730(16)
c/Å	12.7227(11)
α/°	90
β/°	94.895(4)
γ/°	90
Volume/Å^3	4609.1(7)
Z	4
ρ_{calc}g/cm^3	1.504
μ/mm^{-1}	0.828
F(000)	2143.0
Crystal size/mm^3	0.102 × 0.086 × 0.076
Radiation	MoKα (λ = 0.71073)
2Θ range for data collection/°	3.838 to 53.12
Index ranges	-26 ≤ h ≤ 26, -21 ≤ k ≤ 21, -15 ≤ l ≤ 15
Reflections collected	98127
Independent reflections	4788 [R_{int} = 0.1959, R_{sigma} = 0.0644]
Data/restraints/parameters	4788/69/273
Goodness-of-fit on F^2	1.028
Final R indexes [I>=2σ (I)]	R_1 = 0.0577, wR_2 = 0.1347
Final R indexes [all data]	R_1 = 0.0934, wR_2 = 0.1515
Largest diff. peak/hole / e Å^{-3}	0.43/-0.47

3.2.7 [$ZnCl_2$(L4)]

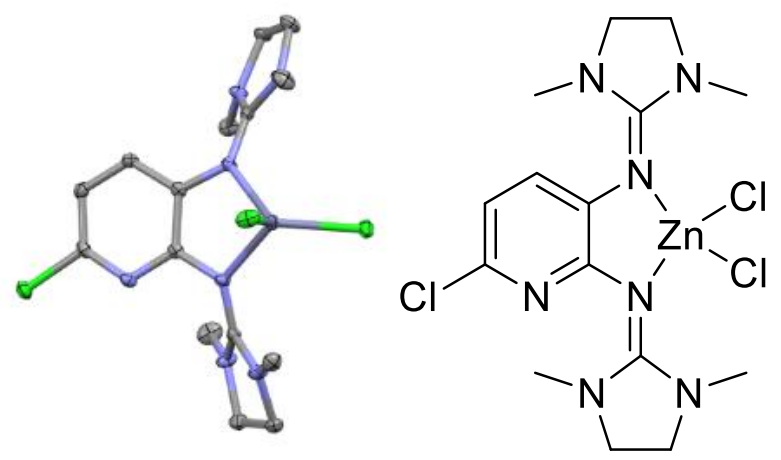

Table S16: Crystal data and structure refinement for [$ZnCl_2$(L4)].

Identification code	mo_2026_hk258_1
Empirical formula	$C_{15}H_{22}Cl_3N_7Zn$
Formula weight	472.11
Temperature/K	100.00
Crystal system	monoclinic
Space group	$P2_1/c$
a/Å	15.6450(8)
b/Å	9.8082(6)
c/Å	26.6460(15)
α/°	90
β/°	97.671(2)
γ/°	90
Volume/Å^3	4052.2(4)
Z	8
ρ_{calc}g/cm^3	1.548
μ/mm^{-1}	1.622
F(000)	1936.0
Crystal size/mm^3	0.39 × 0.3 × 0.21
Radiation	MoKα (λ = 0.71073)
2Θ range for data collection/°	3.776 to 54.996
Index ranges	-20 ≤ h ≤ 20, -12 ≤ k ≤ 12, -34 ≤ l ≤ 34
Reflections collected	235758
Independent reflections	9296 [R_{int} = 0.0722, R_{sigma} = 0.0208]
Data/restraints/parameters	9296/0/488
Goodness-of-fit on F^2	1.040
Final R indexes [I>=2σ (I)]	R_1 = 0.0228, wR_2 = 0.0552
Final R indexes [all data]	R_1 = 0.0248, wR_2 = 0.0565
Largest diff. peak/hole / e Å^{-3}	0.41/-0.37

3.2.8 [$PdCl_2$(L6)]

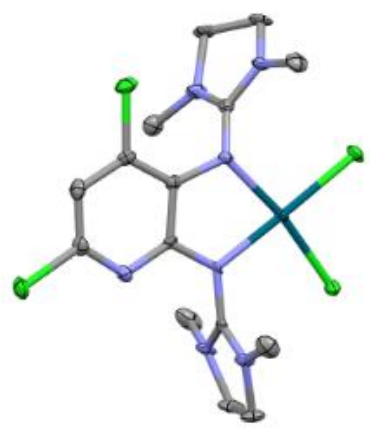 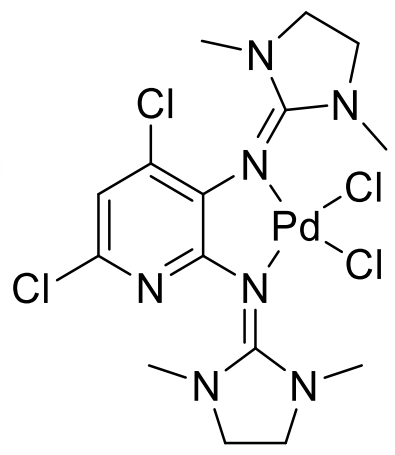

Table S17: Crystal data and structure refinement for [$PdCl_2$(L6)].

Identification code	mo_2026_hk278
Empirical formula	$C_{15}H_{21}Cl_4N_7Pd$
Formula weight	547.59
Temperature/K	100.00
Crystal system	orthorhombic
Space group	$Pna2_1$
a/Å	7.6146(4)
b/Å	27.2404(14)
c/Å	10.1113(5)
α/°	90
β/°	90
γ/°	90
Volume/Å^3	2097.33(19)
Z	4
ρ_{calc}g/cm^3	1.734
μ/mm^{-1}	1.410
F(000)	1096.0
Crystal size/mm^3	0.2 × 0.16 × 0.09
Radiation	MoKα (λ = 0.71073)
2Θ range for data collection/°	4.296 to 52.994
Index ranges	-9 ≤ h ≤ 9, -34 ≤ k ≤ 34, -12 ≤ l ≤ 12
Reflections collected	70431
Independent reflections	4353 [R_{int} = 0.0833, R_{sigma} = 0.0302]
Data/restraints/parameters	4353/259/248
Goodness-of-fit on F^2	1.124
Final R indexes [I>=2σ (I)]	R_1 = 0.0321, wR_2 = 0.0646
Final R indexes [all data]	R_1 = 0.0330, wR_2 = 0.0650
Largest diff. peak/hole / e Å^{-3}	0.74/-0.68

3.3 Pyridinium salts

3.3.1 [L2a+Me]I

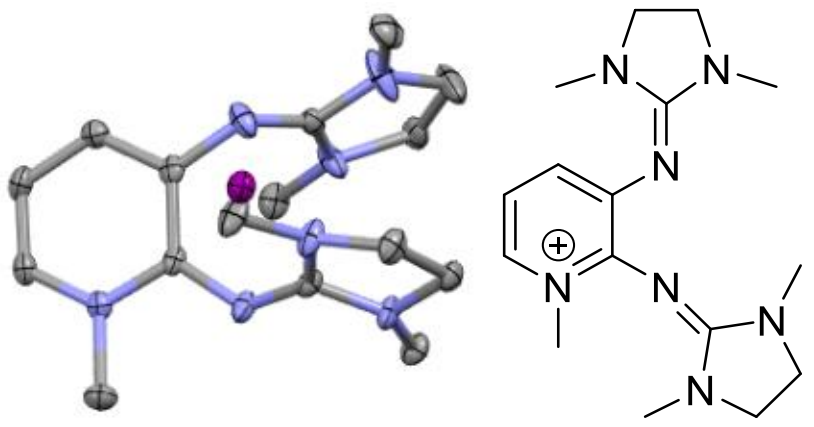

Table S18: Crystal data and structure refinement for [L2a+Me]I.

Identification code	mo_2025_hk130_0ma
Empirical formula	$C_{16}H_{26}IN_7$
Formula weight	443.34
Temperature/K	100.00
Crystal system	orthorhombic
Space group	$Pca2_1$
a/Å	17.1223(8)
b/Å	7.6556(4)
c/Å	29.0066(11)
α/°	90
β/°	90
γ/°	90
Volume/Å^3	3802.2(3)
Z	8
ρ_{calc}g/cm^3	1.549
μ/mm^{-1}	1.697
F(000)	1792.0
Crystal size/mm^3	0.33 × 0.23 × 0.15
Radiation	MoKα (λ = 0.71073)
2Θ range for data collection/°	4.758 to 57.672
Index ranges	-23 ≤ h ≤ 23, -10 ≤ k ≤ 10, -39 ≤ l ≤ 39
Reflections collected	203546
Independent reflections	9943 [R_{int} = 0.0786, R_{sigma} = 0.0276]
Data/restraints/parameters	9943/1/443
Goodness-of-fit on F^2	1.051
Final R indexes [I>=2σ (I)]	R_1 = 0.0198, wR_2 = 0.0452
Final R indexes [all data]	R_1 = 0.0206, wR_2 = 0.0456
Largest diff. peak/hole / e Å^{-3}	0.38/-0.35
Flack parameter	-0.018(6)

3.3.2 [L2a+Aly]Br

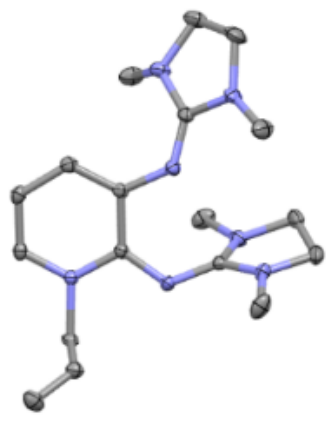 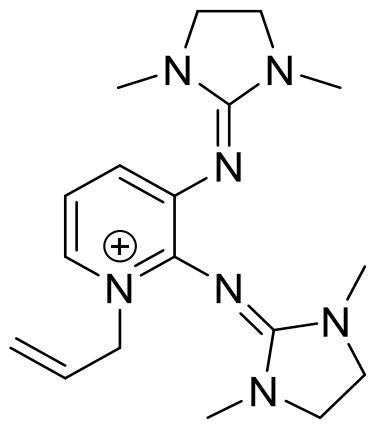

Table S19: Crystal data and structure refinement for [L2a+Aly]Br.

Identification code	mo_2025_hk132_01a
Empirical formula	$C_{18}H_{28}BrN_7$
Formula weight	422.38
Temperature/K	100.00
Crystal system	monoclinic
Space group	$P2_1/n$
a/Å	13.1660(7)
b/Å	10.7878(5)
c/Å	14.2931(8)
α/°	90
β/°	96.328(2)
γ/°	90
Volume/Å^3	2017.71(18)
Z	4
ρ_{calc}g/cm^3	1.390
μ/mm^{-1}	2.054
F(000)	880.0
Crystal size/mm^3	0.273 × 0.207 × 0.206
Radiation	MoKα (λ = 0.71073)
2Θ range for data collection/°	3.992 to 55.262
Index ranges	-17 ≤ h ≤ 17, -14 ≤ k ≤ 14, -18 ≤ l ≤ 18
Reflections collected	97543
Independent reflections	4664 [R_{int} = 0.0808, R_{sigma} = 0.0271]
Data/restraints/parameters	4664/0/239
Goodness-of-fit on F^2	1.061
Final R indexes [I>=2σ (I)]	R_1 = 0.0385, wR_2 = 0.1182
Final R indexes [all data]	R_1 = 0.0443, wR_2 = 0.1235
Largest diff. peak/hole / e Å^{-3}	0.55/-0.76

3.3.3 [L2a+Bn]Br

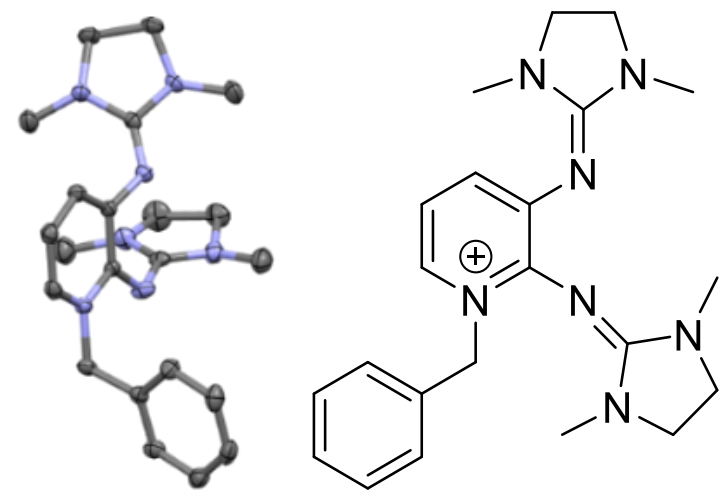

Table S20: Crystal data and structure refinement for [L2a+Bn]Br.

Identification code	mo_2024_hk23_0ma
Empirical formula	$C_{22}H_{30}BrN_7$
Formula weight	472.44
Temperature/K	100.00
Crystal system	monoclinic
Space group	$P2_1/c$
a/Å	9.4565(3)
b/Å	19.1799(7)
c/Å	12.8078(5)
α/°	90
β/°	100.9110(10)
γ/°	90
Volume/Å^3	2281.02(14)
Z	4
ρ_{calc}g/cm^3	1.376
μ/mm^{-1}	1.825
F(000)	984.0
Crystal size/mm^3	0.78 × 0.4 × 0.39
Radiation	MoKα (λ = 0.71073)
2Θ range for data collection/°	3.872 to 54.996
Index ranges	-12 ≤ h ≤ 12, -24 ≤ k ≤ 24, -16 ≤ l ≤ 16
Reflections collected	94013
Independent reflections	5238 [R_{int} = 0.0778, R_{sigma} = 0.0424]
Data/restraints/parameters	5238/0/275
Goodness-of-fit on F^2	1.033
Final R indexes [I>=2σ (I)]	R_1 = 0.0351, wR_2 = 0.0894
Final R indexes [all data]	R_1 = 0.0396, wR_2 = 0.0925
Largest diff. peak/hole / e Å^{-3}	0.76/-0.30

3.3.4 [L2a+Bn(*p*-CF_3)]Br

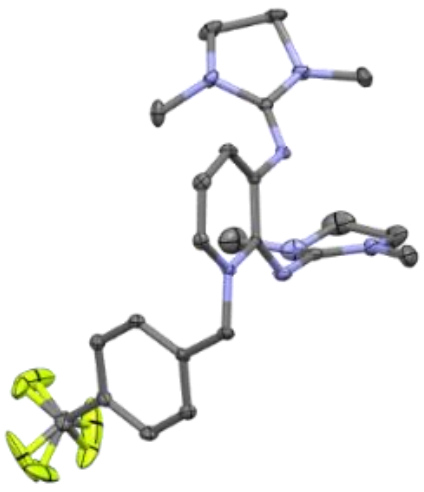

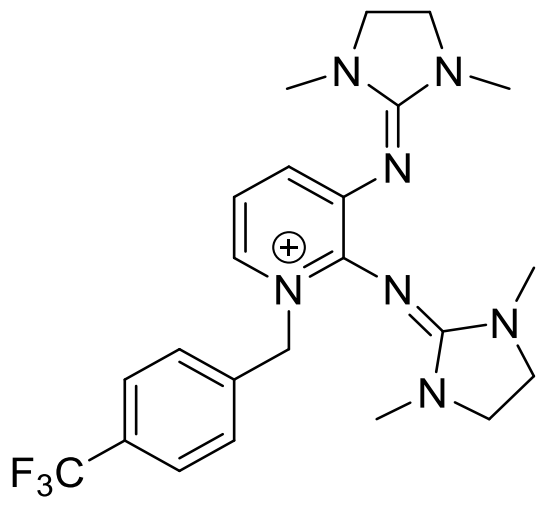

Table S21: Crystal data and structure refinement for [L2a+Bn(*p*-CF_3)]Br.

Identification code	mo_2024_hk34_0m
Empirical formula	$C_{23}H_{29}BrF_3N_7$
Formula weight	540.44
Temperature/K	100.00
Crystal system	triclinic
Space group	P-1
a/Å	7.4541(8)
b/Å	10.3508(10)
c/Å	16.3280(14)
α/°	90.713(4)
β/°	93.986(4)
γ/°	102.191(4)
Volume/Å^3	1227.9(2)
Z	2
ρ_{calc}g/cm^3	1.462
μ/mm^{-1}	1.722
F(000)	556.0
Crystal size/mm^3	0.3 × 0.21 × 0.16
Radiation	MoKα (λ = 0.71073)
2Θ range for data collection/°	4.028 to 53.994
Index ranges	-9 ≤ h ≤ 9, -13 ≤ k ≤ 13, -20 ≤ l ≤ 20
Reflections collected	59452
Independent reflections	5364 [R_{int} = 0.0547, R_{sigma} = 0.0272]
Data/restraints/parameters	5364/90/338
Goodness-of-fit on F^2	1.049
Final R indexes [I>=2σ (I)]	R_1 = 0.0305, wR_2 = 0.0802
Final R indexes [all data]	R_1 = 0.0328, wR_2 = 0.0815
Largest diff. peak/hole / e Å^{-3}	0.62/-0.27

3.3.5 [L2a+Bn(*o*,*p*-CF_3)$_2$]Br

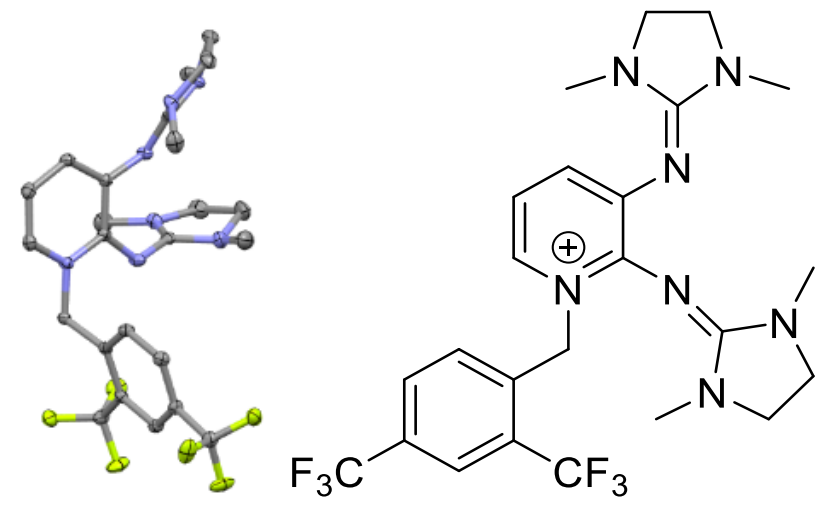

Table S22: Crystal data and structure refinement for [L2a+Bn(*o*,*p*-CF_3)$_2$]Br.

Identification code	mo_2025_hk209_0m
Empirical formula	$C_{23}H_{32}BrN_7$
Formula weight	486.46
Temperature/K	100.00
Crystal system	triclinic
Space group	P-1
a/Å	7.4160(3)
b/Å	10.4004(3)
c/Å	15.9203(6)
α/°	91.1270(10)
β/°	101.3030(10)
γ/°	104.9500(10)
Volume/Å^3	1160.15(7)
Z	2
ρ_{calc}g/cm^3	1.393
μ/mm^{-1}	1.797
F(000)	508.0
Crystal size/mm^3	0.29 × 0.24 × 0.08
Radiation	MoKα (λ = 0.71073)
2Θ range for data collection/°	4.064 to 54.372
Index ranges	-9 ≤ h ≤ 9, -13 ≤ k ≤ 13, -20 ≤ l ≤ 20
Reflections collected	37769
Independent reflections	5168 [R_{int} = 0.0523, R_{sigma} = 0.0297]
Data/restraints/parameters	5168/0/285
Goodness-of-fit on F^2	1.049
Final R indexes [I>=2σ (I)]	R_1 = 0.0245, wR_2 = 0.0591
Final R indexes [all data]	R_1 = 0.0268, wR_2 = 0.0603
Largest diff. peak/hole / e Å^{-3}	0.41/-0.21

3.3.6 [L2a+Bn(*o*-CF_3)]Br

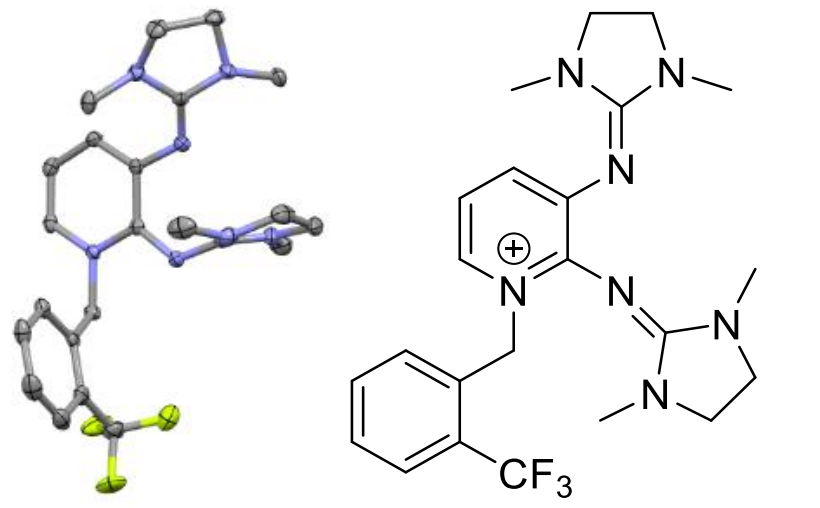

Table S23: Crystal data and structure refinement for [L2a+Bn(*o*-CF_3)]Br.

Identification code	mo_2025_hk205_0m
Empirical formula	$C_{23}H_{29}N_7F_3Br$
Formula weight	540.44
Temperature/K	100.00
Crystal system	monoclinic
Space group	$P2_1/c$
a/Å	7.4090(3)
b/Å	32.6832(15)
c/Å	10.3642(5)
α/°	90
β/°	103.884(2)
γ/°	90
Volume/Å^3	2436.36(19)
Z	4
ρ_{calc}g/cm^3	1.473
μ/mm^{-1}	1.736
F(000)	1112.0
Crystal size/mm^3	0.2 × 0.16 × 0.12
Radiation	MoKα (λ = 0.71073)
2Θ range for data collection/°	4.236 to 57
Index ranges	-9 ≤ h ≤ 9, -43 ≤ k ≤ 43, -13 ≤ l ≤ 13
Reflections collected	35352
Independent reflections	6150 [R_{int} = 0.0613, R_{sigma} = 0.0425]
Data/restraints/parameters	6150/0/311
Goodness-of-fit on F^2	1.213
Final R indexes [I>=2σ (I)]	R_1 = 0.0479, wR_2 = 0.0940
Final R indexes [all data]	R_1 = 0.0582, wR_2 = 0.0973
Largest diff. peak/hole / e Å^{-3}	0.34/-0.63

3.3.7 [L2a+Bn(*o*-Me)]Br

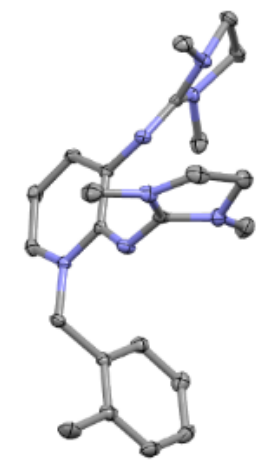 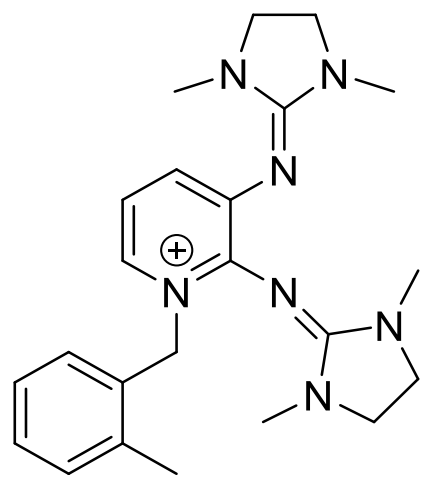

Table S24: Crystal data and structure refinement for [L2a+Bn(*o*-Me)]Br.

Identification code	mo_2025_hk194_0m
Empirical formula	$C_{24}H_{28}BrF_6N_7$
Formula weight	608.44
Temperature/K	100.00
Crystal system	triclinic
Space group	P-1
a/Å	7.5661(7)
b/Å	10.5577(9)
c/Å	16.8050(14)
α/°	91.041(3)
β/°	95.274(3)
γ/°	104.307(3)
Volume/Å^3	1294.1(2)
Z	2
ρ_{calc}g/cm^3	1.561
μ/mm^{-1}	1.660
F(000)	620.0
Crystal size/mm^3	0.23 × 0.17 × 0.1
Radiation	MoKα (λ = 0.71073)
2Θ range for data collection/°	3.984 to 54.532
Index ranges	-9 ≤ h ≤ 9, -13 ≤ k ≤ 13, -21 ≤ l ≤ 21
Reflections collected	75904
Independent reflections	5781 [R_{int} = 0.0751, R_{sigma} = 0.0303]
Data/restraints/parameters	5781/0/347
Goodness-of-fit on F^2	1.056
Final R indexes [I>=2σ (I)]	R_1 = 0.0314, wR_2 = 0.0711
Final R indexes [all data]	R_1 = 0.0385, wR_2 = 0.0738
Largest diff. peak/hole / e Å^{-3}	0.45/-0.37

3.3.8 [L2a+Br+Bn(*p*-CF_3)]$CuBr_4$

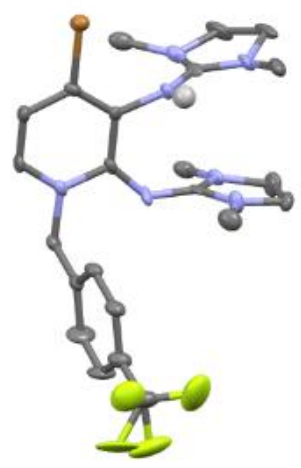 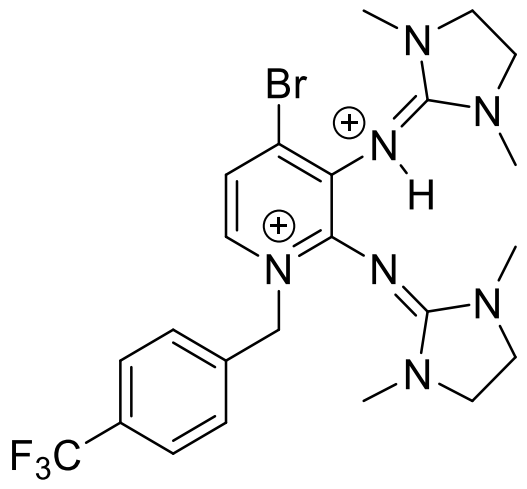

Table S25: Crystal data and structure refinement for [L2a+Br+Bn(*p*-CF_3)]$CuBr_4$.

Identification code	mo_2026_hk56_4
Empirical formula	$C_{23}H_{29}Br_5CuF_3N_7$
Formula weight	923.62
Temperature/K	100.00
Crystal system	monoclinic
Space group	$P2_1/c$
a/Å	9.1983(8)
b/Å	18.6521(19)
c/Å	18.3333(18)
α/°	90
β/°	103.286(4)
γ/°	90
Volume/Å^3	3061.2(5)
Z	4
ρ_{calc}g/cm^3	2.004
μ/mm^{-1}	7.285
F(000)	1788.0
Crystal size/mm^3	0.3 × 0.21 × 0.16
Radiation	MoKα (λ = 0.71073)
2Θ range for data collection/°	4.368 to 52.354
Index ranges	-11 ≤ h ≤ 11, -23 ≤ k ≤ 0, -22 ≤ l ≤ 22
Reflections collected	12026
Independent reflections	6125 [R_{int} = 0.0490, R_{sigma} = 0.0679]
Data/restraints/parameters	6125/58/369
Goodness-of-fit on F^2	1.100
Final R indexes [I>=2σ (I)]	R_1 = 0.0482, wR_2 = 0.1048
Final R indexes [all data]	R_1 = 0.0719, wR_2 = 0.1150
Largest diff. peak/hole / e Å^{-3}	1.03/-1.42

3.3.9 [L2b+Bn]Br

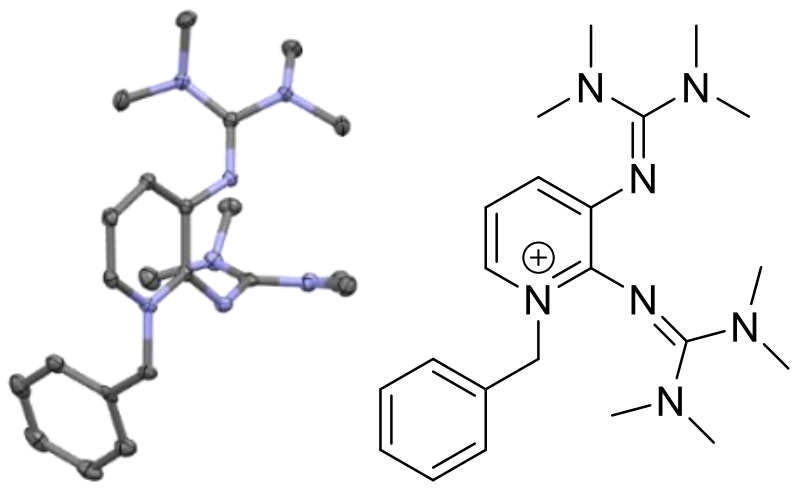

Table S26: Crystal data and structure refinement for [L2b+Bn]Br.

Identification code	mo_2024_hk21_01a
Empirical formula	$C_{22}H_{34}BrN_7$
Formula weight	476.47
Temperature/K	100.00
Crystal system	orthorhombic
Space group	Pbca
a/Å	18.4854(11)
b/Å	12.8067(9)
c/Å	20.2205(15)
α/°	90
β/°	90
γ/°	90
Volume/Å^3	4786.9(6)
Z	8
ρ_{calc}g/cm^3	1.322
μ/mm^{-1}	1.740
F(000)	2000.0
Crystal size/mm^3	0.266 × 0.241 × 0.078
Radiation	MoKα (λ = 0.71073)
2Θ range for data collection/°	4.028 to 54.376
Index ranges	-23 ≤ h ≤ 23, -16 ≤ k ≤ 16, -25 ≤ l ≤ 25
Reflections collected	126752
Independent reflections	5323 [R_{int} = 0.1185, R_{sigma} = 0.0444]
Data/restraints/parameters	5323/0/279
Goodness-of-fit on F^2	1.014
Final R indexes [I>=2σ (I)]	R_1 = 0.0339, wR_2 = 0.0826
Final R indexes [all data]	R_1 = 0.0467, wR_2 = 0.0907
Largest diff. peak/hole / e Å^{-3}	0.59/-0.36

3.3.10 [L3+Bn(*p*-CF_3)]Br

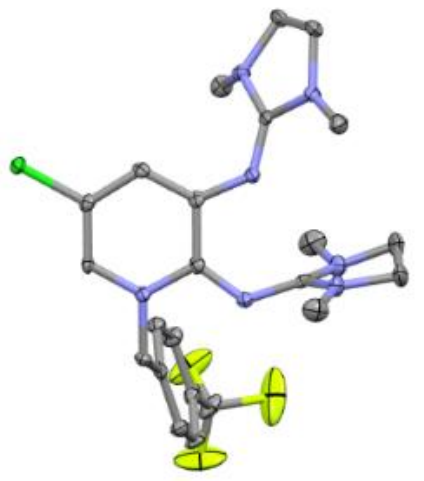

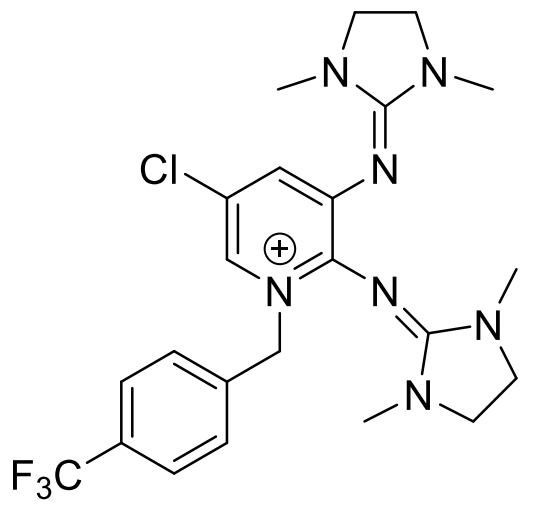

Table S27: Crystal data and structure refinement for [L3+Bn(*p*-CF_3)]Br.

Identification code	mo_2025_hkee29_0m
Empirical formula	$C_{23}H_{28}BrClF_3N_7$
Formula weight	574.88
Temperature/K	100.00
Crystal system	triclinic
Space group	P-1
a/Å	7.2075(8)
b/Å	10.1617(11)
c/Å	17.8301(18)
α/°	98.894(4)
β/°	91.687(4)
γ/°	100.455(4)
Volume/Å^3	1266.5(2)
Z	2
ρ_{calc}g/cm^3	1.507
μ/mm^{-1}	1.777
F(000)	588.0
Crystal size/mm^3	0.29 × 0.06 × 0.05
Radiation	MoKα (λ = 0.71073)
2Θ range for data collection/°	4.13 to 52.998
Index ranges	-9 ≤ h ≤ 9, -12 ≤ k ≤ 12, -22 ≤ l ≤ 22
Reflections collected	52482
Independent reflections	5232 [R_{int} = 0.1125, R_{sigma} = 0.0537]
Data/restraints/parameters	5232/84/348
Goodness-of-fit on F^2	1.052
Final R indexes [I>=2σ (I)]	R_1 = 0.0474, wR_2 = 0.1074
Final R indexes [all data]	R_1 = 0.0652, wR_2 = 0.1186
Largest diff. peak/hole / e Å^{-3}	0.67/-0.66

3.3.11 [L3+Bn(*o*,*p*-CF_3)$_2$]Br

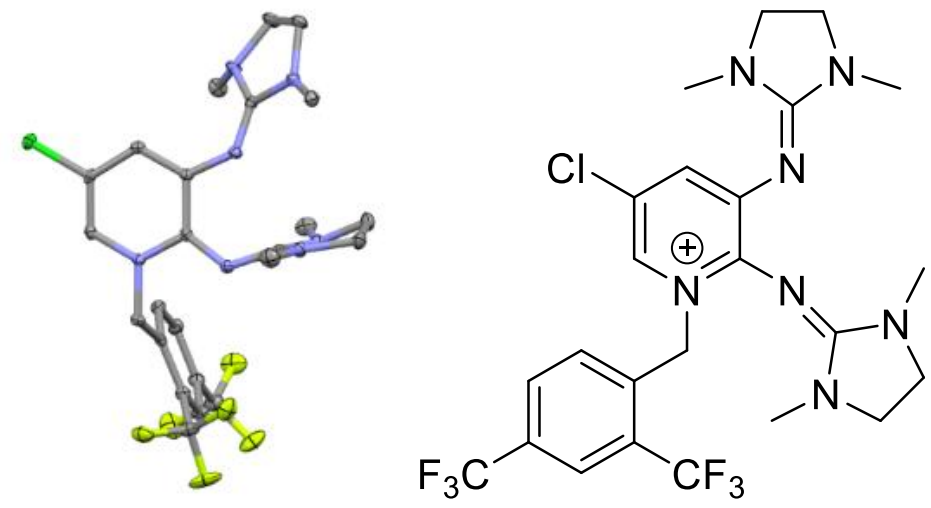

Table S28: Crystal data and structure refinement for [L3+Bn(*o*,*p*-CF_3)$_2$]Br.

Identification code	mo_2026_hkee28_0m
Empirical formula	$C_{24}H_{27}BrClF_6N_7$
Formula weight	642.88
Temperature/K	100.00
Crystal system	triclinic
Space group	P-1
a/Å	7.1636(4)
b/Å	10.2698(5)
c/Å	18.1815(11)
α/°	97.343(2)
β/°	91.683(3)
γ/°	92.197(2)
Volume/Å^3	1324.85(13)
Z	2
ρ_{calc}g/cm^3	1.612
μ/mm^{-1}	1.724
F(000)	652.0
Crystal size/mm^3	0.17 × 0.16 × 0.07
Radiation	MoKα (λ = 0.71073)
2Θ range for data collection/°	4.002 to 56.862
Index ranges	-9 ≤ h ≤ 9, -13 ≤ k ≤ 13, -24 ≤ l ≤ 24
Reflections collected	64367
Independent reflections	6664 [R_{int} = 0.0747, R_{sigma} = 0.0340]
Data/restraints/parameters	6664/0/356
Goodness-of-fit on F^2	1.039
Final R indexes [I>=2σ (I)]	R_1 = 0.0283, wR_2 = 0.0659
Final R indexes [all data]	R_1 = 0.0342, wR_2 = 0.0691
Largest diff. peak/hole / e Å^{-3}	0.45/-0.36

3.3.12 [L5a+Bn^b]Br

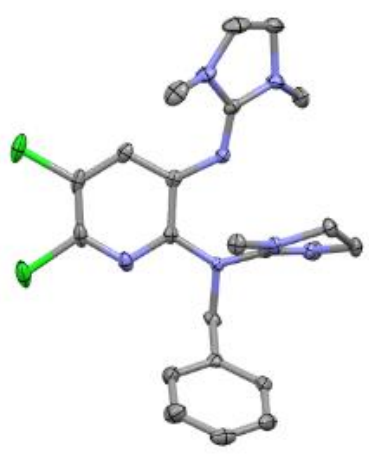

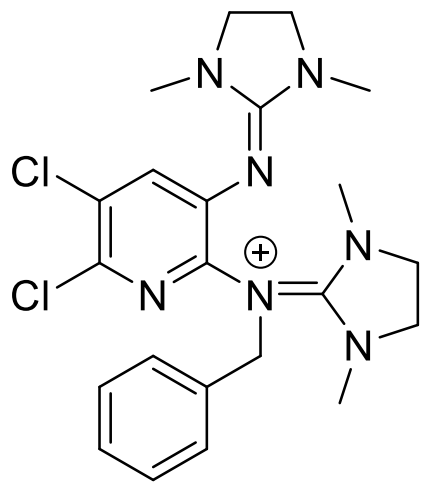

Table S29: Crystal data and structure refinement for [L5a+Bn^b]Br.

Identification code	mo_2026_hk248_0m
Empirical formula	$C_{26}H_{34}BrCl_2N_9$
Formula weight	623.43
Temperature/K	100.00
Crystal system	triclinic
Space group	P-1
a/Å	7.9680(4)
b/Å	11.7351(6)
c/Å	16.4602(8)
α/°	103.391(2)
β/°	100.985(2)
γ/°	93.244(2)
Volume/Å^3	1461.64(13)
Z	2
ρ_{calc}g/cm^3	1.417
μ/mm^{-1}	1.622
F(000)	644.0
Crystal size/mm^3	0.39 × 0.27 × 0.21
Radiation	MoKα (λ = 0.71073)
2Θ range for data collection/°	3.876 to 56.84
Index ranges	-10 ≤ h ≤ 10, -15 ≤ k ≤ 15, -22 ≤ l ≤ 21
Reflections collected	77125
Independent reflections	7350 [R_{int} = 0.0569, R_{sigma} = 0.0259]
Data/restraints/parameters	7350/0/321
Goodness-of-fit on F^2	1.035
Final R indexes [I>=2σ (I)]	R_1 = 0.0261, wR_2 = 0.0636
Final R indexes [all data]	R_1 = 0.0319, wR_2 = 0.0656
Largest diff. peak/hole / e Å^{-3}	0.36/-0.26

3.3.13 [L5a+Bn^b(p-CF_3)]Br

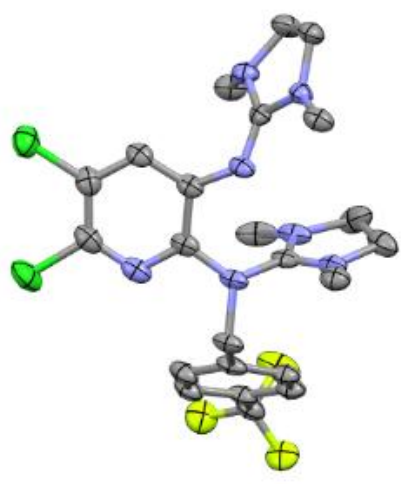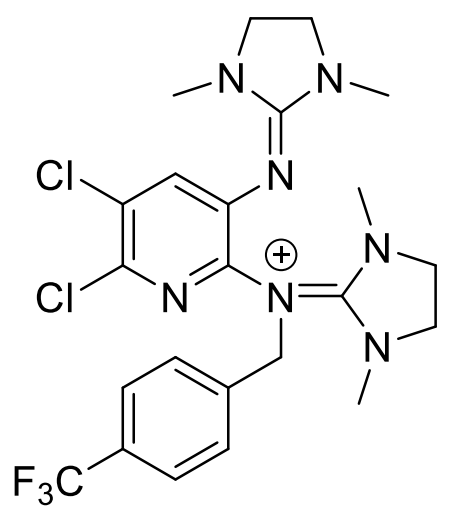

Table S30: Crystal data and structure refinement for [L5a+Bn^b(p-CF_3)]Br.

Identification code	mo_2026_hk249L_0m
Empirical formula	$C_{23}H_{27}Cl_2F_3N_7Br + 0.7 \cdot H_2O$
Formula weight	621.94
Temperature/K	100.00
Crystal system	triclinic
Space group	P-1
a/Å	12.650(2)
b/Å	14.499(3)
c/Å	17.729(3)
α/°	68.939(7)
β/°	77.235(7)
γ/°	69.458(7)
Volume/Å^3	2824.8(9)
Z	4
ρ_{calc}g/cm^3	1.462
μ/mm^{-1}	1.693
F(000)	1268.0
Crystal size/mm^3	0.24 × 0.2 × 0.08
Radiation	MoKα (λ = 0.71073)
2Θ range for data collection/°	3.854 to 51.998
Index ranges	-15 ≤ h ≤ 15, -17 ≤ k ≤ 17, -21 ≤ l ≤ 21
Reflections collected	111690
Independent reflections	11051 [R_{int} = 0.1119, R_{sigma} = 0.0693]
Data/restraints/parameters	11051/147/738
Goodness-of-fit on F^2	1.036
Final R indexes [I>=2σ (I)]	R_1 = 0.0566, wR_2 = 0.1667
Final R indexes [all data]	R_1 = 0.0760, wR_2 = 0.1846
Largest diff. peak/hole / e Å^{-3}	1.06/-0.59

3.3.14 [L6+Bn^{b}]Br

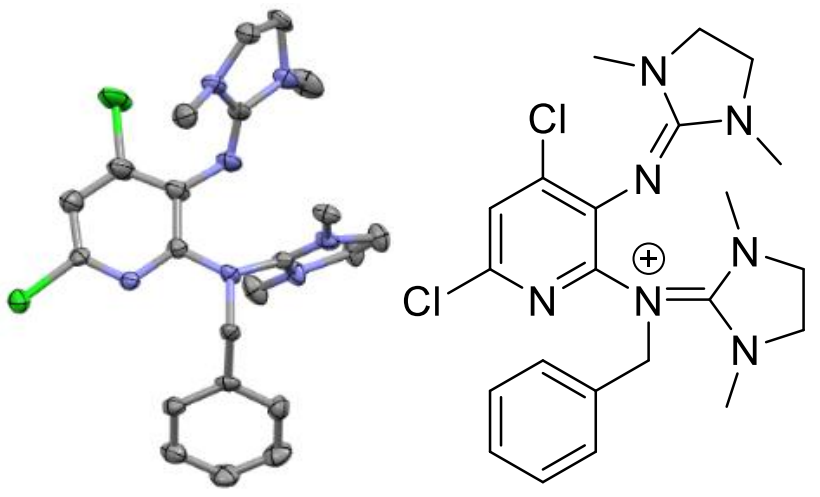

Table S31: Crystal data and structure refinement for [L6+Bn^{b}]Br.

Identification code	mo_2026_hk236_1ma
Empirical formula	$C_{22}H_{28}BrCl_2N_7$ + 0.12 · H_2O
Formula weight	543.57
Temperature/K	100.00
Crystal system	monoclinic
Space group	$P2_1/n$
a/Å	14.9862(14)
b/Å	9.9110(10)
c/Å	34.292(3)
α/°	90
β/°	101.562(4)
γ/°	90
Volume/Å^3	4989.9(8)
Z	8
ρ_{calc}g/cm^3	1.447
μ/mm^{-1}	1.887
F(000)	2234.0
Crystal size/mm^3	0.23 × 0.21 × 0.11
Radiation	MoKα (λ = 0.71073)
2Θ range for data collection/°	4.108 to 54
Index ranges	-19 ≤ h ≤ 19, -12 ≤ k ≤ 12, -43 ≤ l ≤ 43
Reflections collected	177938
Independent reflections	10889 [R_{int} = 0.0982, R_{sigma} = 0.0424]
Data/restraints/parameters	10889/0/607
Goodness-of-fit on F^2	1.280
Final R indexes [I>=2σ (I)]	R_1 = 0.0957, wR_2 = 0.1876
Final R indexes [all data]	R_1 = 0.1193, wR_2 = 0.1982
Largest diff. peak/hole / e Å^{-3}	1.22/-1.76

3.3.15 [L6+Bn^b^(*p*-CF_3)]Br

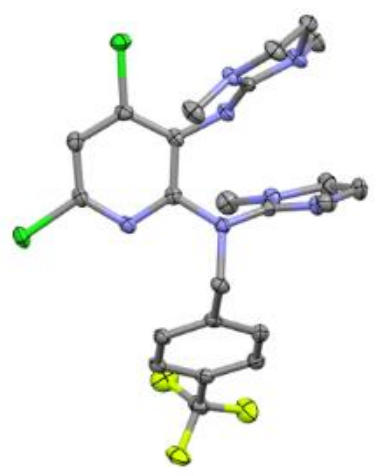

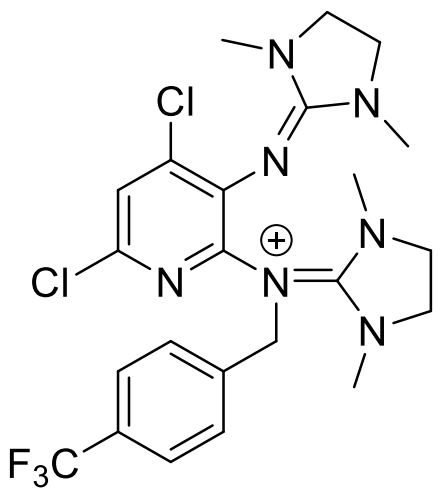

Table S32: Crystal data and structure refinement for [L6+Bn^b^(*p*-CF_3)]Br.

Identification code	mo_2025_hkee27_0ma
Empirical formula	$C_{23}H_{29}BrCl_2F_3N_7O$
Formula weight	627.34
Temperature/K	100.00
Crystal system	monoclinic
Space group	$P2_1/c$
a/Å	17.8493(9)
b/Å	10.4018(5)
c/Å	14.8336(6)
α/°	90
β/°	103.019(2)
γ/°	90
Volume/Å^3	2683.3(2)
Z	4
ρ_{calc}g/cm^3	1.553
μ/mm^{-1}	1.783
F(000)	1280.0
Crystal size/mm^3	0.14 × 0.08 × 0.05
Radiation	MoKα (λ = 0.71073)
2Θ range for data collection/°	4.562 to 56.71
Index ranges	-23 ≤ h ≤ 23, -13 ≤ k ≤ 13, -19 ≤ l ≤ 19
Reflections collected	184046
Independent reflections	6687 [R_{int} = 0.0815, R_{sigma} = 0.0202]
Data/restraints/parameters	6687/0/341
Goodness-of-fit on F^2	1.053
Final R indexes [I>=2σ (I)]	R_1 = 0.0332, wR_2 = 0.0739
Final R indexes [all data]	R_1 = 0.0431, wR_2 = 0.0795
Largest diff. peak/hole / e Å^{-3}	0.57/-0.61

4. DFT Calculations

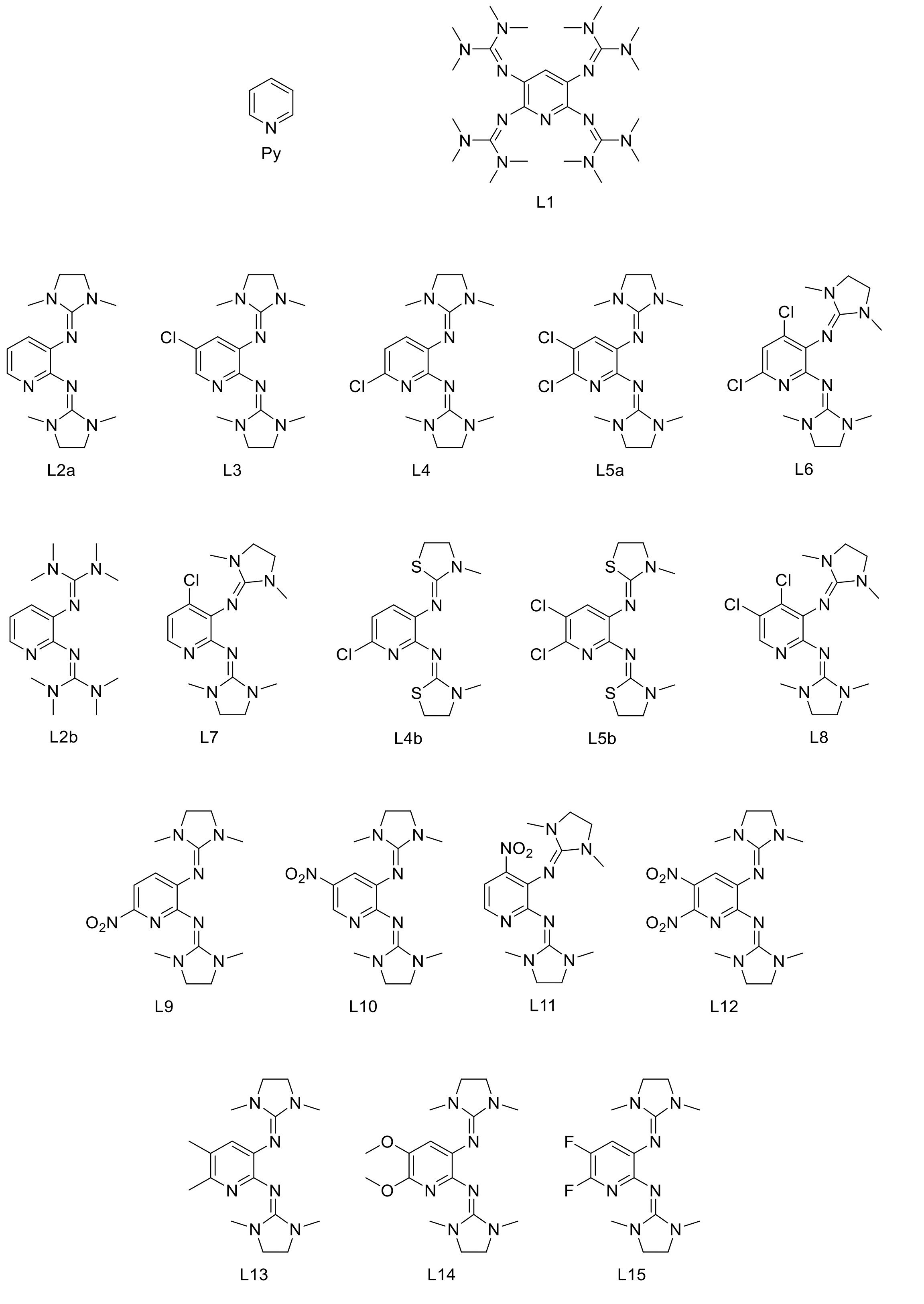

Figure S109: Overview of all calculated ligands.

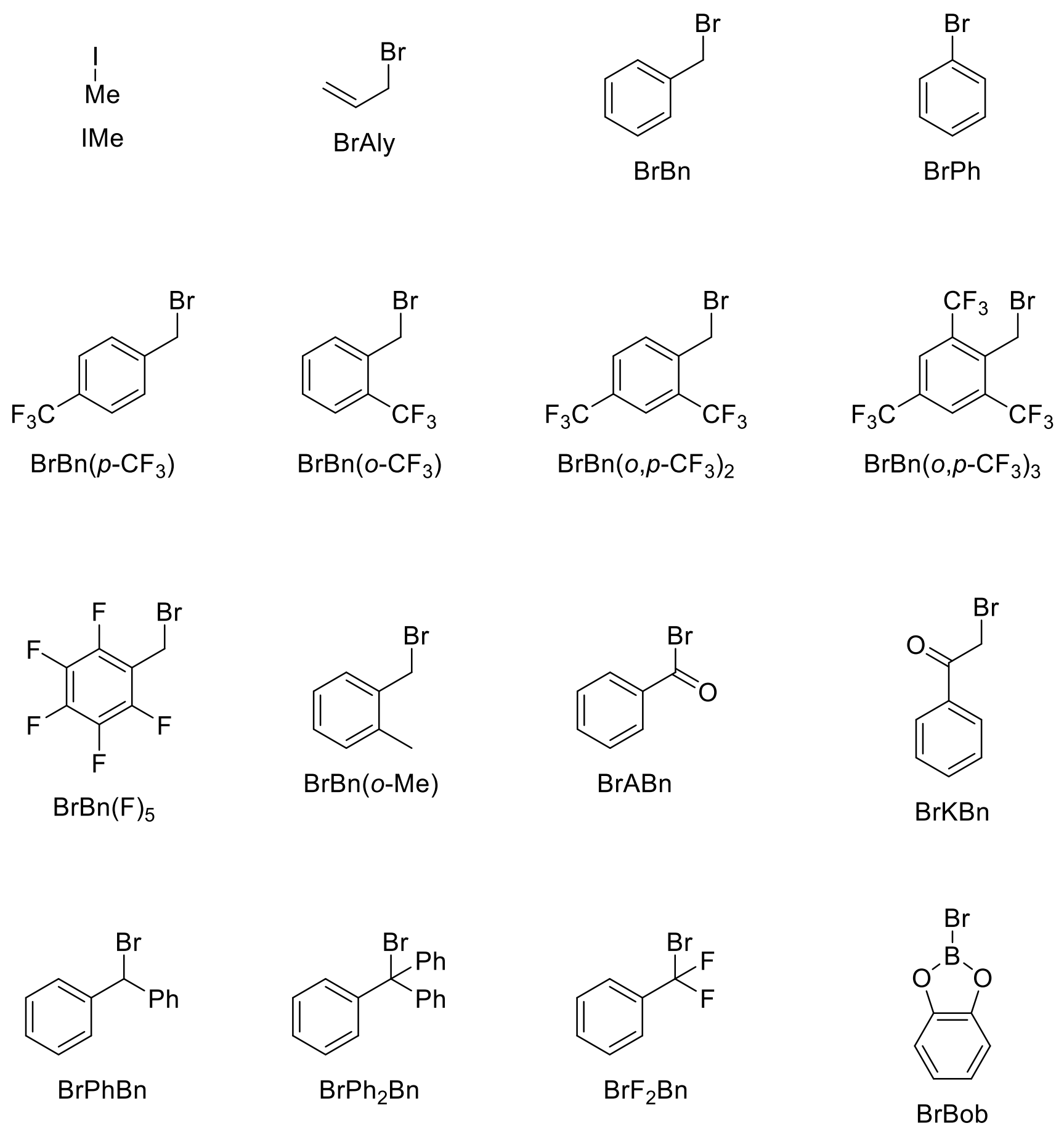

Figure S110: Overview of all calculated substrates.

4.1 Protonation

G ((L2b+H**a**)$^+$) = −2549248 kJ mol^{-1}

G ((L2b+H**b**)$^+$) = −2549279 kJ mol^{-1}

G ((L2b+H**c**)$^+$) = −2549289 kJ mol^{-1}

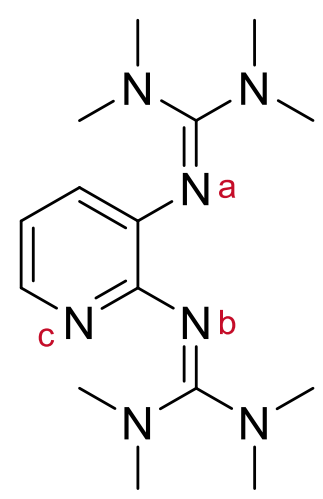

4.2 Bond cleavage energies

Table S33: Comparison of the bond cleavage between several ligands and benzyl.

ΔG [kJ mol^{-1}]	Radical ΔG^a	Ionic ΔG^b	Ionisation energy ligand ΔG^c
[Py+Bn]$^+$	338	167	857
[L1+Bn]$^+$	90.8	323	454
[L2a+Bn]$^+$	185	301	570
[L2b+Bn]$^+$	179	307	559
[L3+Bn]$^+$	144	283	547
[L4+Bn]$^+$	119	266	539
[L4b+Bn]$^+$	142	227	601
[L5a+Bn]$^+$	133	246	573
[L5b+Bn]$^+$	122	200	608
[L6+Bn]$^+$	124	259	551
[L7+Bn]$^+$	156	292	551
[L8+Bn]$^+$	164	277	574
[L9+Bn]$^+$	153	226	614
[L10+Bn]$^+$	194	264	616
[L11+Bn]$^+$	178	280	584
[L12+Bn]$^+$	164	206	645
[L13+Bn]$^+$	144	292	539
[L14+Bn]$^+$	110	274	522
[L15+Bn]$^+$	131	237	580

[a] $[L+Bn]^+ \rightarrow [L]^{\cdot+} + [Bn]^{\cdot}$ $\Delta G = \Delta G([L]^{\cdot+}) + \Delta G([Bn]^{\cdot}) - \Delta G([L+Bn]^+)$ in kJ mol^{-1}.

[b] $[L+Bn]^+ \rightarrow L + [Bn]^+$ $\Delta G = \Delta G(L) + \Delta G([Bn]^+) - \Delta G([L+Bn]^+)$ in kJ mol^{-1}.

[c] $\Delta G = \Delta G([L]^{\cdot+}) - \Delta G(L)$ in kJ mol^{-1}.

Table S34: Comparison of the bond cleavage between several adducts from L2a.

ΔG [kJ mol^{-1}]	Radical[a]	Ionic[b]
$[L2a+Me]^+$	235	621
$[L2a+Aly]^+$	163	370
$[L2a+Bn]^+$	185	301
$[L2a+Bn(p\text{-}CF_3)]^+$	171	330
$[L2a+Bn(o,p\text{-}CF_3)_2]^+$	163	359
$[L2a+Bn(o,p\text{-}CF_3)_3]^+$	147	374
$[L2a+Bn(o\text{-}CF_3)]^+$	174	329
$[L2a+Bn(o\text{-}Me)]^+$	183	280
$[L2a+Bn(F)_5]^+$	163	344
$[L2a+ABn]^+$	202	248
$[L2a+KBn]^+$	206	368
$[L2a+PhBn]^+$	158	203
$[L2a+Ph_2Bn]^+$	95.7	102
$[L2a+F_2Bn]^+$	176	272
$[L2a+Ph]^+$	283	498
$[L2a+Bob]^+$	365	612
$[L2a+NO_2]^+$	−18.2	373

[a] $[L+Bn]^+ \rightarrow [L]^{\cdot+} + [Bn]^{\cdot}$ $\Delta G = \Delta G([L]^{\cdot+}) + \Delta G([Bn]^{\cdot}) - \Delta G([L+Bn]^+)$ in kJ mol^{-1}.

[b] $[L+Bn]^+ \rightarrow L + [Bn]^+$ $\Delta G = \Delta G(L) + \Delta G([Bn]^+) - \Delta G([L+Bn]^+)$ in in kJ mol^{-1}.

4.3 HOMOs

Table S35: Isodensity surfaces for the HOMOs of several ligands and energies of the HOMO−1, HOMO and LUMO.

ligand	L	[L]$^{\cdot+}$
L2a	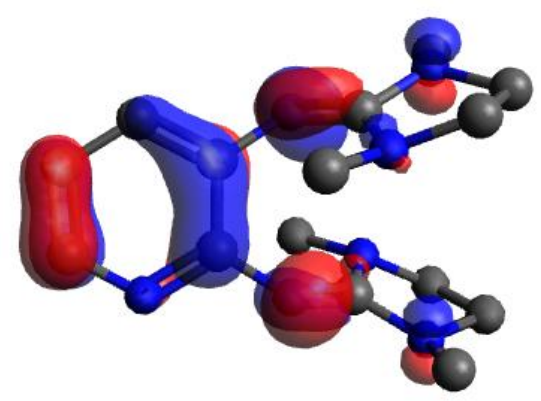<br>E(79) = −5.81 eV<br>E^{HOMO}(80) = −4.87 eV<br>E^{LUMO}(81) = −0.09 eV	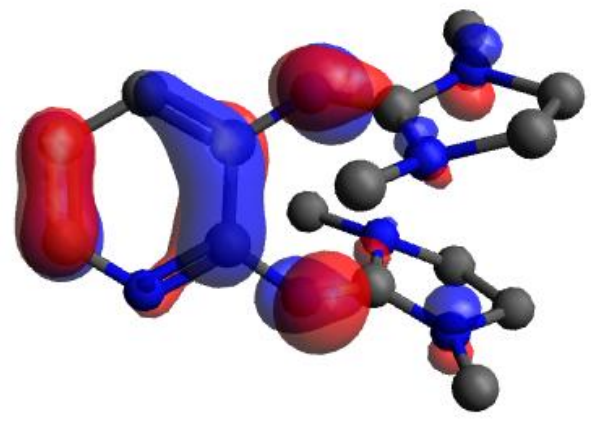<br>E(79) = −9.8348 eV<br>E^{HOMO}(80) = −8.7097 eV<br>E^{LUMO}(81) = −4.0565 eV
L2b	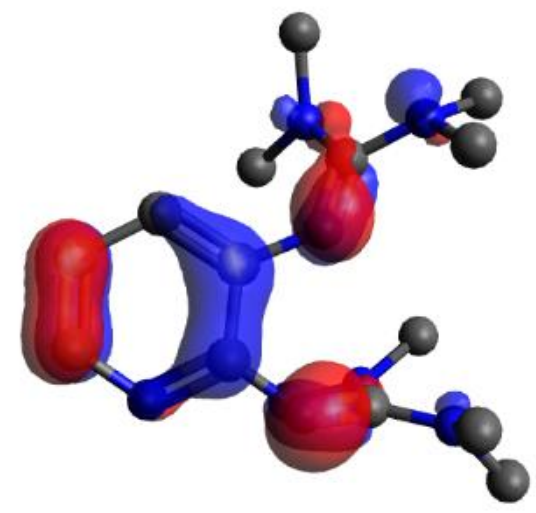<br>E(81) = −5.83 eV<br>E^{HOMO}(82) = −4.91 eV<br>E^{LUMO}(83) = −0.32 eV	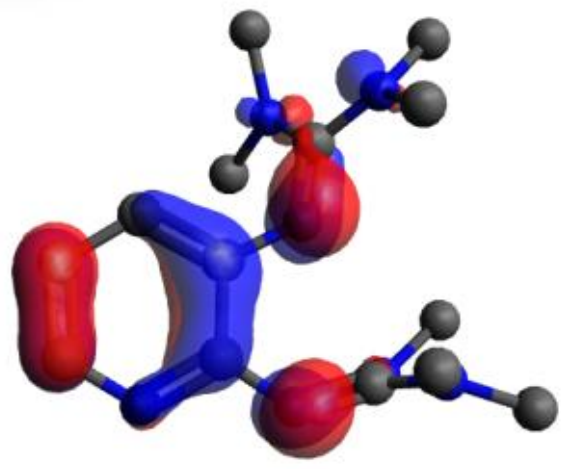<br>E(81) = −9.7094 eV<br>E^{HOMO}(82) = −8.4344 eV<br>E^{LUMO}(83) = −4.0900 eV
L3	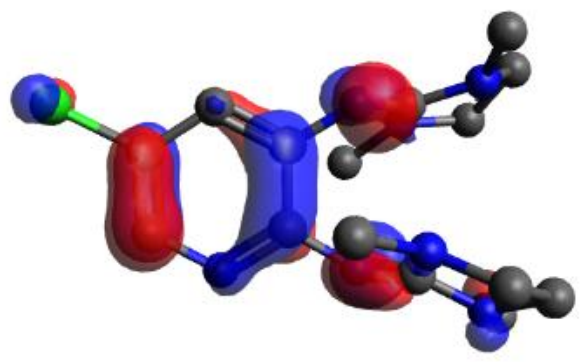<br>E(87) = −6.0014 eV<br>E^{HOMO}(88) = −5.0617 eV<br>E^{LUMO}(89) = −0.3306 eV	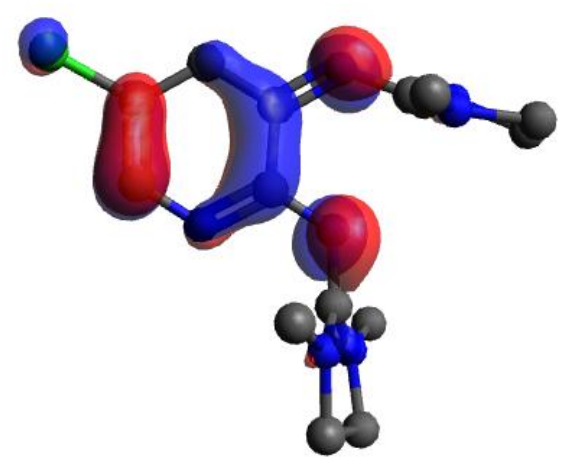<br>E(87) = −9.6862 eV<br>E^{HOMO}(88) = −8.2382 eV<br>E^{LUMO}(89) = −3.8825 eV
L4	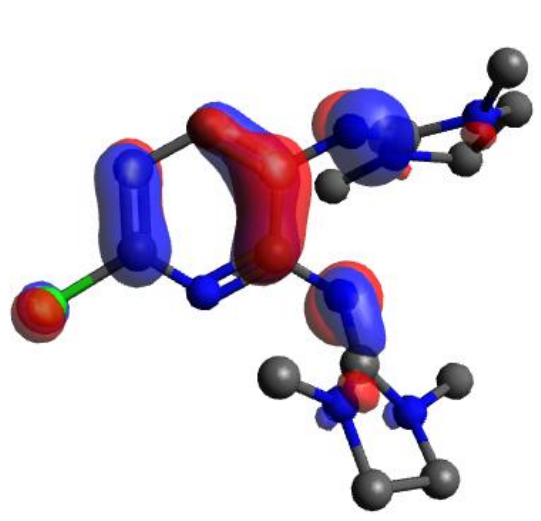<br>E(87) = −5.8968 eV<br>E^{HOMO}(88) = −4.8116 eV<br>E^{LUMO}(89) = −0.2207 eV	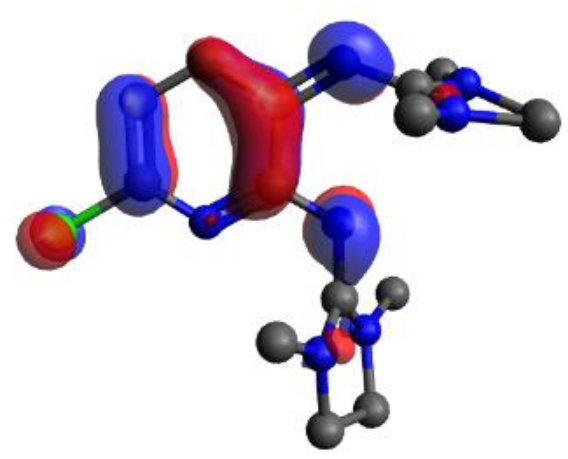<br>E(87) = −9.6884 eV<br>E^{HOMO}(88) = −8.1972 eV<br>E^{LUMO}(89) = −3.8589 eV

L4b	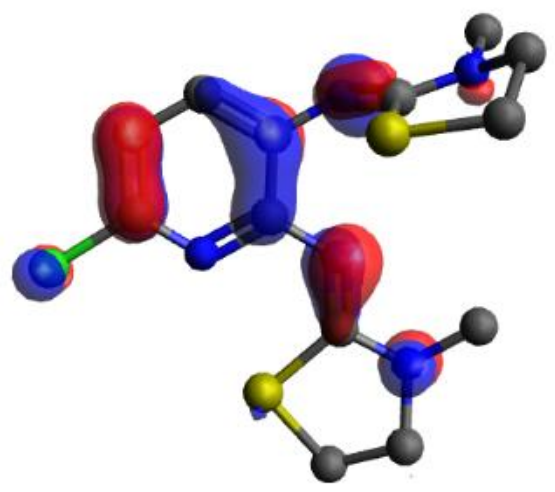<br>$E(87) = -6.2321$ eV<br>$E^{HOMO}(88) = -5.2264$ eV<br>$E^{LUMO}(89) = -0.8352$ eV	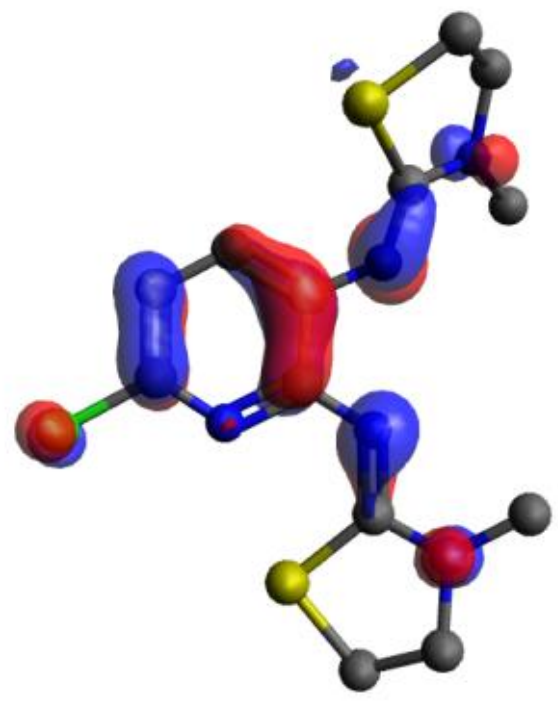<br>$E(87) = -9.7013$ eV<br>$E^{HOMO}(88) = -8.6831$ eV<br>$E^{LUMO}(89) = -4.5640$ eV
L5a	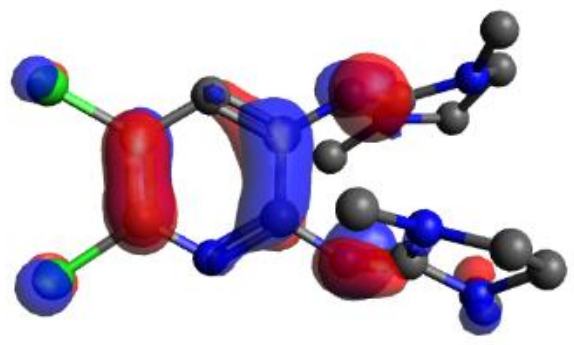<br>$E(95) = -6.1339$ eV<br>$E^{HOMO}(96) = -5.1168$ eV<br>$E^{LUMO}(97) = -0.5285$ eV	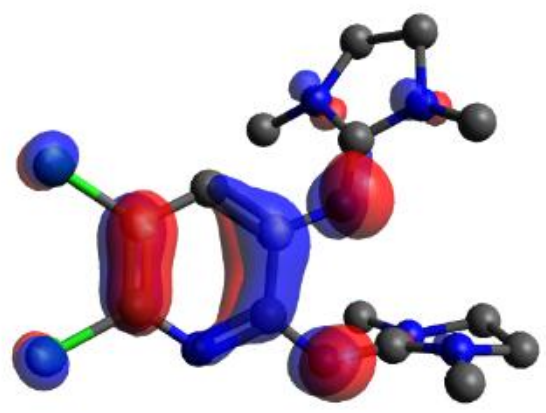<br>$E(95) = -9.8339$ eV<br>$E^{HOMO}(96) = -8.3919$ eV<br>$E^{LUMO}(97) = -4.2374$ eV
L5b	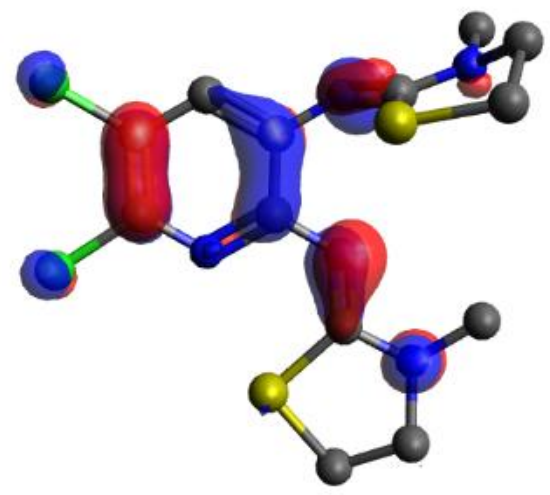<br>$E(95) = -6.3611$ eV<br>$E^{HOMO}(96) = -5.3231$ eV<br>$E^{LUMO}(97) = -1.0117$ eV	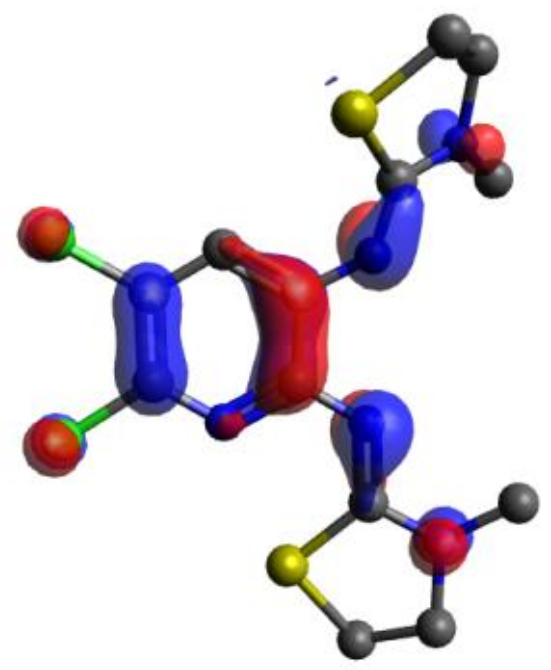<br>$E(95) = -9.7776$ eV<br>$E^{HOMO}(96) = -8.7021$ eV<br>$E^{LUMO}(97) = -4.6492$ eV
L6	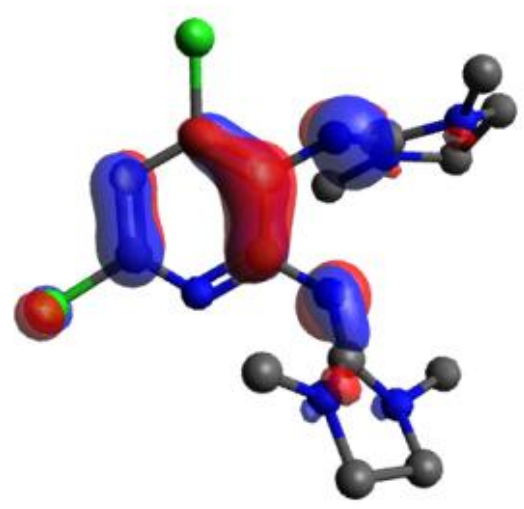<br>$E(95) = -5.9763$ eV<br>$E^{HOMO}(96) = -4.9859$ eV<br>$E^{LUMO}(97) = -0.3825$ eV	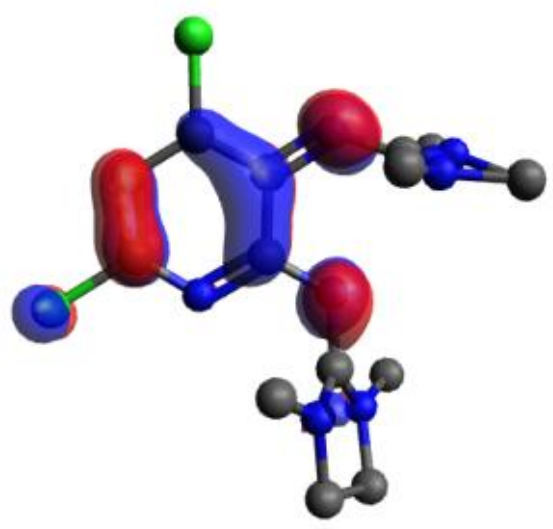<br>$E(95) = -9.7369$ eV<br>$E^{HOMO}(96) = -8.2962$ eV<br>$E^{LUMO}(97) = -3.9416$ eV

L7	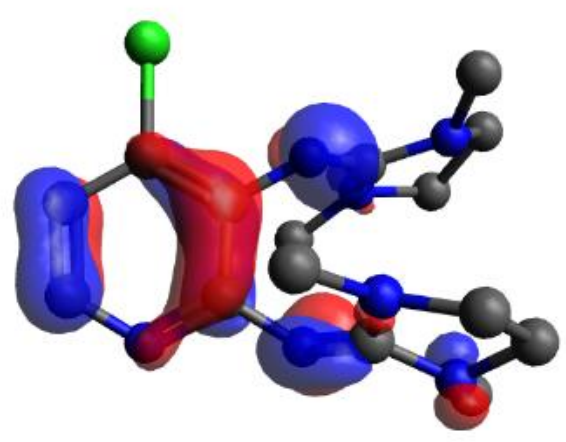<br>$E(87) = -5.9136$ eV<br>$E^{HOMO}(88) = -5.0998$ eV<br>$E^{LUMO}(89) = -0.2018$ eV	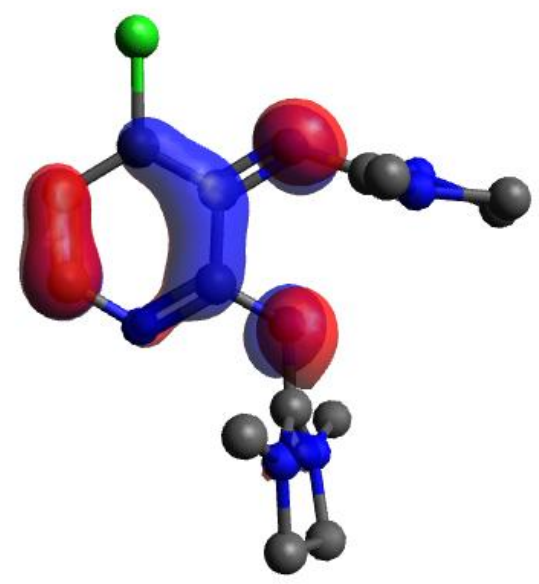<br>$E(87) = -9.6694$ eV<br>$E^{HOMO}(88) = -8.2781$ eV<br>$E^{LUMO}(89) = -3.7818$ eV

Table S36: Isodensity surfaces of the HOMOs of several pyridinium salts and energies of the HOMO−1, HOMO and LUMO.

ligand	$[L+Bn(p\text{-}CF_3)]^+$	$[L+Bn^b(p\text{-}CF_3)]^+$
L4	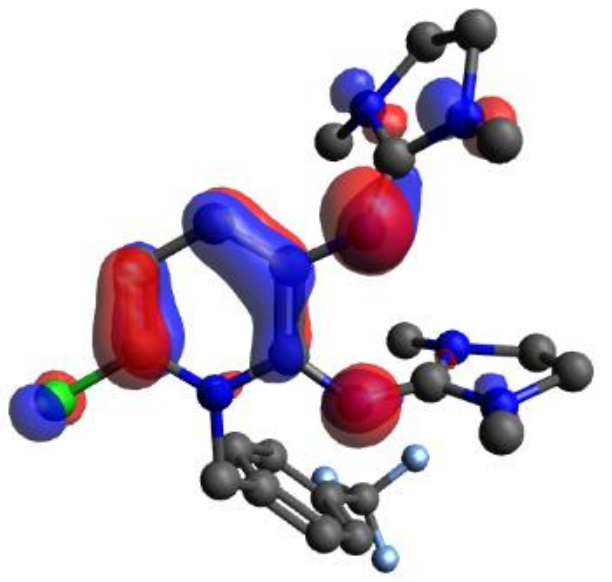<br>E(127) = −9.44417 eV<br>E^{HOMO}(128) = −8.2764 eV<br>E^{LUMO}(129) = −4.4964 eV	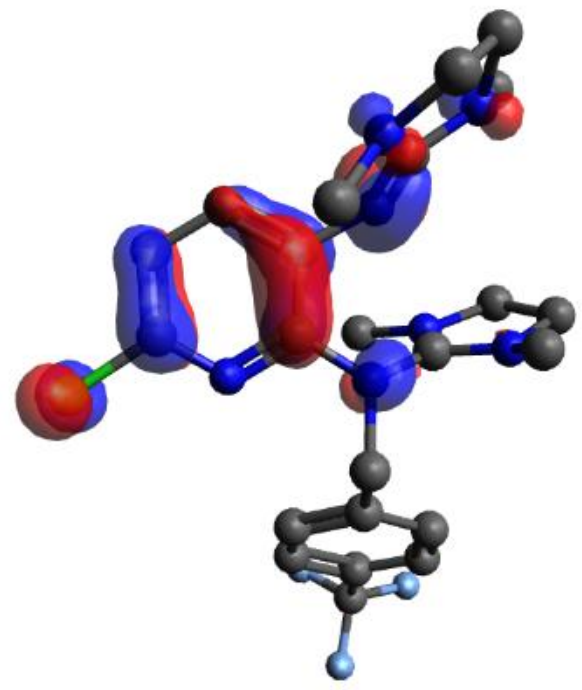<br>E(127) = −9.4915 eV<br>E^{HOMO}(128) = −8.6297 eV<br>E^{LUMO}(129) = −4.3187 eV
L5a	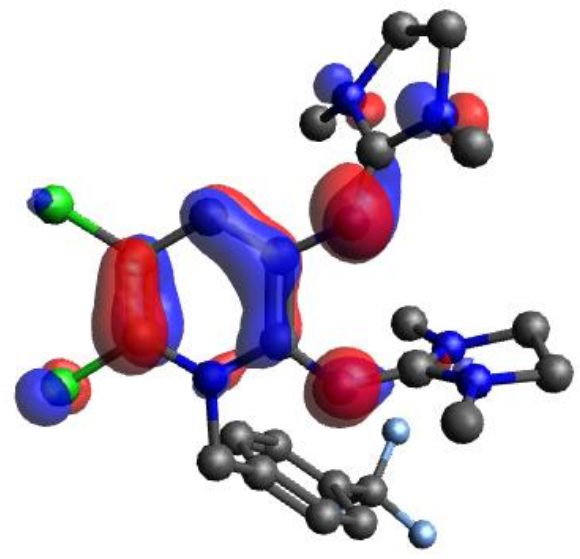<br>E(135) = −9.5088 eV<br>E^{HOMO}(136) = −8.3303 eV<br>E^{LUMO}(137) = −4.6080 eV	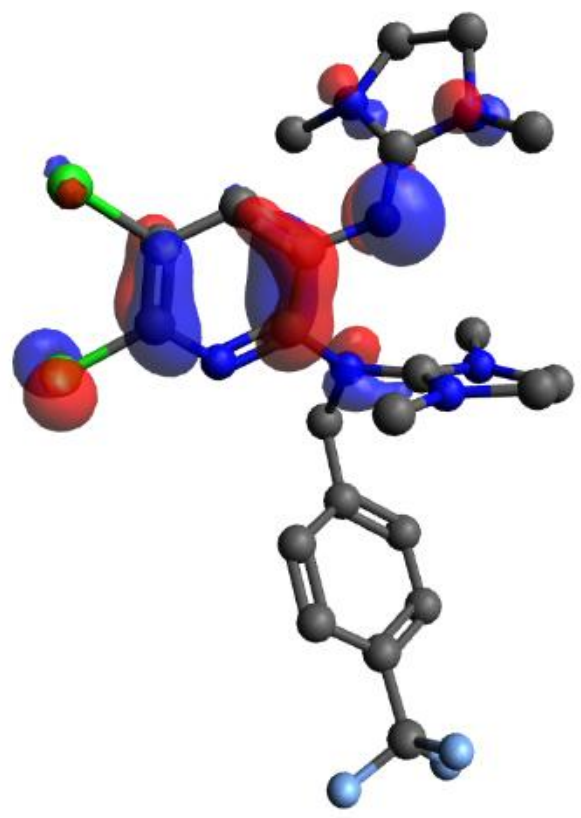<br>E(135) = −9.5387eV<br>E^{HOMO}(136) = −8.5714eV<br>E^{LUMO}(137) = −4.2965eV
L6	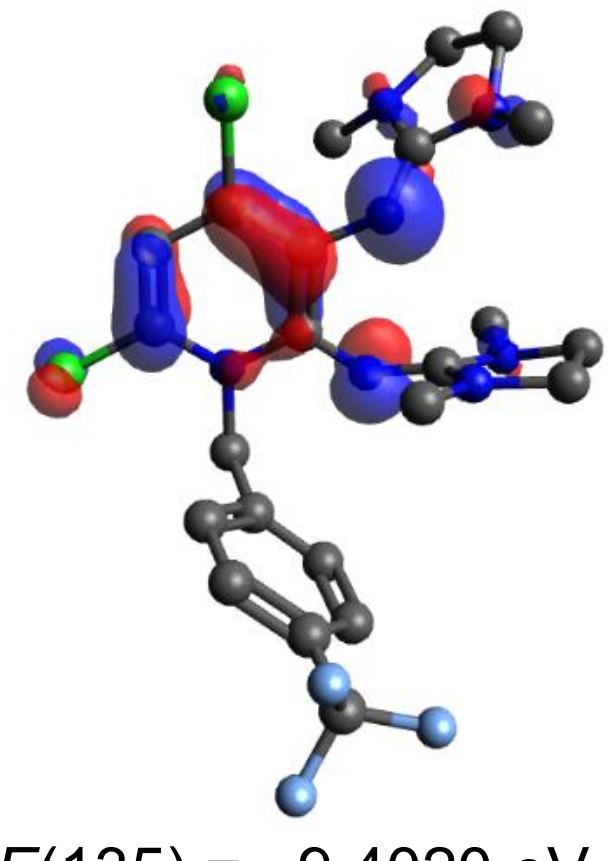<br>E(135) = −9.4020 eV<br>E^{HOMO}(136) = −8.5020 eV<br>E^{LUMO}(137) = −4.5077 eV	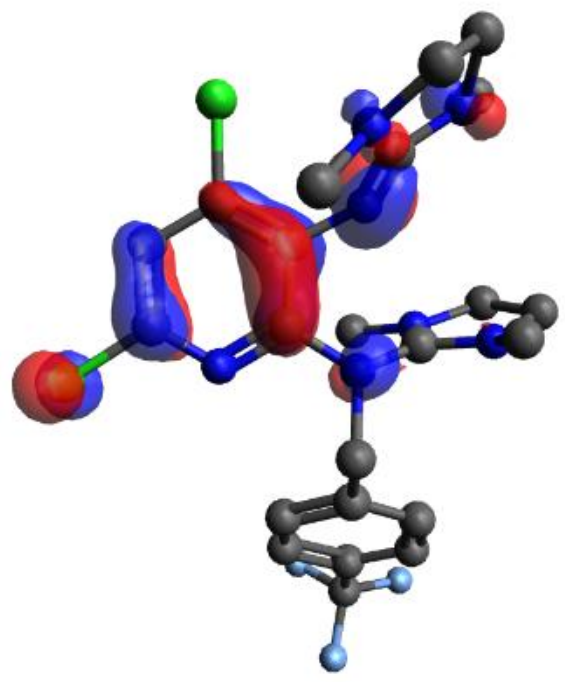<br>E(135) = −9.5494 eV<br>E^{HOMO}(136) = −8.7080 eV<br>E^{LUMO}(137) = −4.3923 eV

4.4 Gibbs free energy changes

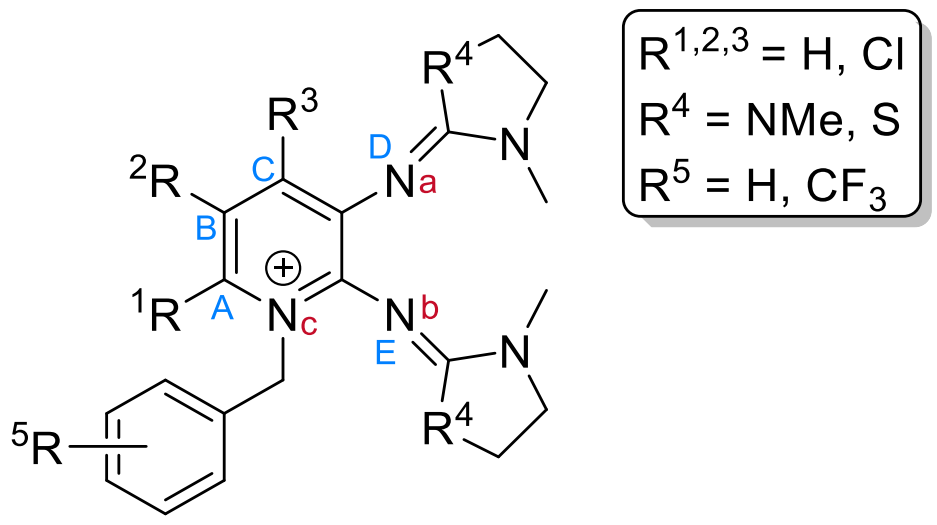

Calculations were carried out using

$\Delta G = G(\text{product}) - G(\text{educt})$

[1] the benzylated species at position **X**

[2] the benzylated pyridine

For benzylated positions at the backbone (**A**, **B**, **C**), the cleaved H is bound to one of the three N positions (**a**, **b**, **c**) in each case.

Table S37: Migration of the benzyl position on ligand L2a.

ΔG [kJ mol^{-1}]	[L2a+H**a**+Bn]$^+$	[L2a+H**b**+Bn]$^+$	[L2a+H**c**+Bn]$^+$	[L2a+Bn]$^+$
[L2a+H+Bn**A**]$^+$	0.33	9.58	−23.8	
[L2a+H+Bn**B**]$^+$	16.0	18.3	−9.53	
[L2a+H+Bn**C**]$^+$	−2.46	2.97	−25.9	
[L2a+Bn**D**]$^+$				45.7
[L2a+Bn**E**]$^+$				42.4
[L2a+Bn(*p*-CF_3)**E**]$^+$				31.8

Table S38: Migration of the benzyl position on ligand L3.

ΔG [kJ mol^{-1}]	[L3+H**a**+Bn]$^+$	[L3+H**b**+Bn]$^+$	[L3+H**c**+Bn]$^+$	[L3+Bn]$^+$
[L3+H+Bn**A**]$^+$	−4.89	−19.6	−26.3	
[L3+H+Bn**C**]$^+$	−7.81	−12.0	−12.5	
[L3+Bn**D**]$^+$				43.1
[L3+Bn**E**]$^+$				29.7
[L3+Bn(*p*-CF_3)**E**]$^+$				34.6

Table S39: Migration of the benzyl position on ligand L4.

Δ*G* [kJ mol^{-1}]	[L4+H**a**+Bn]$^+$	[L4+H**b**+Bn]$^+$	[L4+H**c**+Bn]$^+$	[L4+Bn]$^+$
[L4+H+Bn**B**]$^+$	−17.7	−9.04	−19.5	
[L4+H+Bn**C**]$^+$	−33.8	−33.4	−5.29	
[L4+Bn**D**]$^+$				19.1
[L4+Bn**E**]$^+$				13.4
[L4+Bn(*p*-CF_3)**E**]$^+$				14.1
[L4+Bn(*o*-CF_3)**E**]$^+$				30.5
[L4+Bn(*o*,*p*-CF_3)$_2$**E**]$^+$				41.7

Table S40: Migration of the benzyl position on ligand L4b.

Δ*G* [kJ mol^{-1}]	[L4b+H**a**+Bn]$^+$	[L4b+H**b**+Bn]$^+$	[L4b+H**c**+Bn]$^+$	[L4b+Bn]$^+$
[L4b+Bn**E**]$^+$				27.1
[L4b+Bn(*p*-CF_3)**E**]$^+$				30.2

Table S41: Migration of the benzyl position on ligand L5a.

Δ*G* [kJ mol^{-1}]	[L5a+H**a**+Bn]$^+$	[L5a+H**b**+Bn]$^+$	[L5a+H**c**+Bn]$^+$	[L5a+Bn]$^+$
[L5a+H+Bn**C**]$^+$	−22.4	−32.9	−39.5	
[L5a+Bn**D**]$^+$				10.0
[L5a+Bn**E**]$^+$				5.97
[L5a+Bn(*p*-CF_3)**E**]$^+$				10.9

Table S42: Migration of the benzyl position on ligand L5b.

Δ*G* [kJ mol^{-1}]	[L5b+H**a**+Bn]$^+$	[L5b+H**b**+Bn]$^+$	[L5b+H**c**+Bn]$^+$	[L5b+Bn]$^+$
[L5b+Bn**E**]$^+$				22.2
[L5b+Bn(*p*-CF_3)**E**]$^+$				10.7

Table S43: Migration of the benzyl position on ligand L6.

Δ*G* [kJ mol^{-1}]	[L6+H**a**+Bn]$^+$	[L6+H**b**+Bn]$^+$	[L6+H**c**+Bn]$^+$	[L6+Bn]$^+$
[L6+H+Bn**B**]$^+$	−14.5	−8.29	−19.1	
[L6+Bn**D**]$^+$				16.5
[L6+Bn**E**]$^+$				10.2
[L6+Bn(*p*-CF_3)**E**]$^+$				13.9
[L6+Bn(*o*-CF_3)**E**]$^+$				21.1
[L6+Bn(*o*,*p*-CF_3)$_2$**E**]$^+$				31.9

As the ligand L2a was detected in mass spectrometry data with several benzyl groups, calculations were carried out to investigate a potential second position of addition. At this process, the H remains at the attacked position and the radical is delocalised across the molecule.

$\Delta G = \Delta G([\text{L2a+Hc+BnA/C+BnX}]^{\cdot+}) - (\Delta G([\text{L2a+Hc+BnA/C}^{+}]) + \Delta G([\text{Bn}]^{\cdot}))$

Table S44: Addition of a second benzyl radical to [L2a+Hc+BnA]$^{+}$.

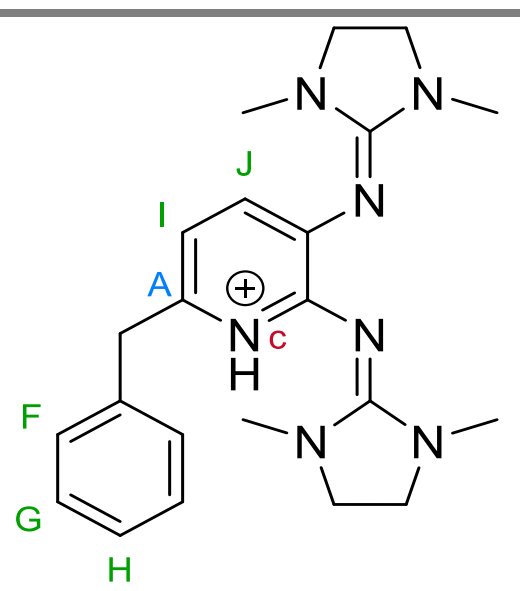	$[\text{L2a+Hc+BnA+BnF}]^{\cdot+}$	ΔG = 64.9 kJ mol^{-1}
	$[\text{L2a+Hc+BnA+BnG}]^{\cdot+}$	ΔG = 67.4 kJ mol^{-1}
	$[\text{L2a+Hc+BnA+BnH}]^{\cdot+}$	ΔG = 65.3 kJ mol^{-1}
	$[\text{L2a+Hc+BnA+BnI}]^{\cdot+}$	ΔG = 67.3 kJ mol^{-1}
	$[\text{L2a+Hc+BnA+BnJ}]^{\cdot+}$	ΔG = 11.1 kJ mol^{-1}

Table S45: Addition of a second benzyl radical to [L2a+Hc+BnC]$^{+}$.

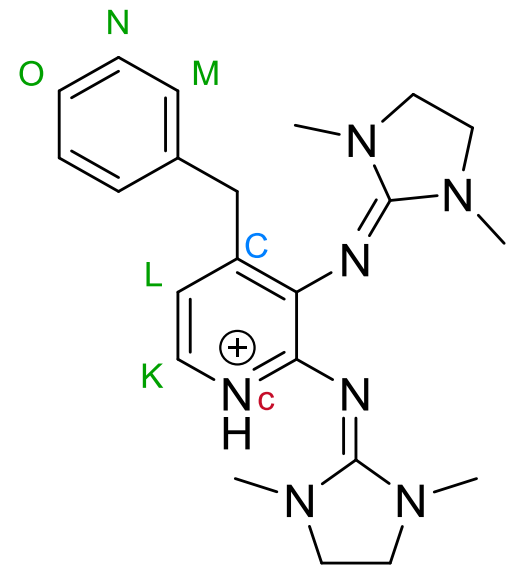	$[\text{L2a+Hc+BnC+BnK}]^{\cdot+}$	ΔG = 4.21 kJ mol^{-1}
	$[\text{L2a+Hc+BnC+BnL}]^{\cdot+}$	ΔG = 74.0 kJ mol^{-1}
	$[\text{L2a+Hc+BnC+BnM}]^{\cdot+}$	ΔG = 67.7 kJ mol^{-1}
	$[\text{L2a+Hc+BnC+BnN}]^{\cdot+}$	ΔG = 61.3 kJ mol^{-1}
	$[\text{L2a+Hc+BnC+BnO}]^{\cdot+}$	ΔG = 72.9 kJ mol^{-1}
