# Supplementary material for: The Radical Chemistry of 2,3‐Diguanidinopyridines: Coordination and Visible‐Light Photochemistry
Source: Chemistry. 2026 Aug 18;32(41):e71582. doi: 10.1002/chem.71582 (PMC13729947; doi:10.1002/chem.71582)
Supplement: Supplementary file 2 [file CHEM-32-e71582-s002.pdf]

Alkyls and Benzyls

$[Me]^{+}$
E [Eh]: −39.467365240489

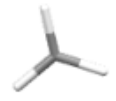

C	4.875582000	9.969635000	5.538784000
H	3.866359000	9.623910000	5.305582000
H	5.546236000	10.279094000	4.734460000
H	5.214174000	10.005962000	6.576334000

$[Me]^{\cdot}$
E [Eh]: −39.827977037905

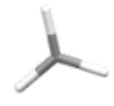

C	4.875447000	9.969569000	5.538754000
H	3.879351000	9.624448000	5.308006000
H	5.537833000	10.277234000	4.744223000
H	5.209719000	10.007349000	6.564176000

$[Aly]^{+}$
E [Eh]: −116.941342462611

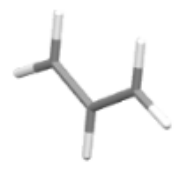

C	4.793370000	9.848181000	6.104566000
H	5.522639000	9.393659000	6.767728000
H	3.758804000	9.537799000	6.215413000
C	5.159415000	10.781290000	5.160025000
H	6.193645000	11.086151000	5.055157000
C	4.182182000	11.319168000	4.352151000
H	3.143703000	11.018202000	4.452082000
H	4.414652000	12.058360000	3.592088000

$[Aly]^{\cdot}$
E [Eh]: −117.234297510049

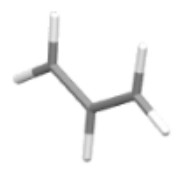

C	4.806393000	9.820373000	6.130606000
H	5.576214000	9.404714000	6.765390000
H	3.793061000	9.465837000	6.275012000
C	5.097335000	10.768006000	5.170411000
H	6.132693000	11.083669000	5.073615000
C	4.173170000	11.344834000	4.323433000
H	3.125930000	11.072030000	4.371254000
H	4.463614000	12.083347000	3.589489000

$[Ph]^{+}$
E [Eh]: −231.215084398590

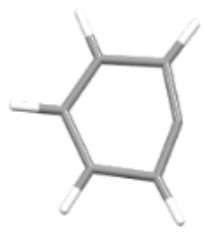

C	5.813790000	4.597434000	7.154596000
C	7.002569000	4.363823000	6.634121000
H	7.825087000	5.023921000	6.401215000
C	4.657080000	4.102360000	7.549107000
H	3.784690000	4.573601000	7.977361000
C	5.921315000	2.154168000	6.732362000
H	5.968154000	1.089498000	6.548203000
C	7.012801000	2.949553000	6.410684000
H	7.923023000	2.550415000	5.977533000
C	4.770810000	2.699612000	7.285650000
H	3.904151000	2.102326000	7.545718000

$[Ph]^{\cdot}$
E [Eh]: −231.515966958980

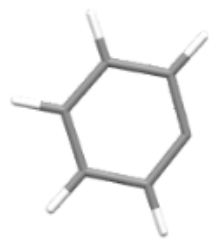

C	5.804173000	4.819056000	7.193507000
C	6.962511000	4.332353000	6.645606000
H	7.803088000	4.969765000	6.401826000
C	4.699445000	4.079914000	7.528572000
H	3.811286000	4.524955000	7.959152000
C	5.921473000	2.150653000	6.731596000
H	5.968516000	1.085017000	6.547612000
C	7.011859000	2.953679000	6.411519000
H	7.903714000	2.515504000	5.979772000
C	4.771269000	2.703813000	7.285811000
H	3.926136000	2.072000000	7.531577000

$[Bcat]^{+}$
E [Eh]: −405.993963146748

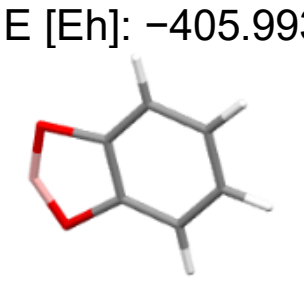

B	5.769455000	6.336537000	8.124772000
O	4.644201000	5.729994000	8.279528000
O	6.809515000	6.604636000	8.835415000
C	6.226577000	5.910058000	10.024461000
C	4.966841000	5.405381000	9.703283000
C	4.184821000	4.723951000	10.590893000
C	6.799049000	5.765715000	11.255112000
C	6.014080000	5.066553000	12.181227000
C	4.753420000	4.566209000	11.861711000
H	7.777040000	6.159103000	11.492235000
H	6.407007000	4.914606000	13.177024000
H	3.208496000	4.339417000	10.332865000
H	4.188189000	4.035720000	12.615565000

$[Bcat]^{\cdot}$
E [Eh]: −406.305930923091

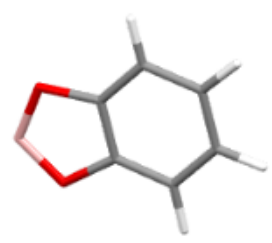

B	5.787454000	6.420152000	7.921329000
O	4.683273000	5.727854000	8.365267000
O	6.751881000	6.557742000	8.894287000
C	6.225617000	5.915495000	10.009479000
C	4.969869000	5.413345000	9.689291000
C	4.201435000	4.726635000	10.602827000
C	6.782034000	5.755400000	11.258957000
C	6.013749000	5.059323000	12.196026000
C	4.752912000	4.558084000	11.875902000
H	7.760485000	6.149053000	11.496070000
H	6.407633000	4.907617000	13.192126000
H	3.225207000	4.341413000	10.343701000
H	4.187140000	4.025767000	12.628828000

[Bn(*p*-CF_3)]$^+$
E [Eh]: −607.665929375487

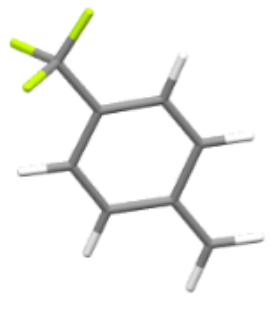

C	3.880875000	4.559630000	11.651099000
H	3.736403000	4.674993000	12.719324000
H	4.808701000	4.933124000	11.232683000
C	2.933317000	3.970500000	10.863988000
C	1.713678000	3.484318000	11.442457000
H	1.556326000	3.589370000	12.508367000
C	0.763457000	2.897008000	10.648196000
H	-0.160396000	2.524272000	11.066442000
C	1.002039000	2.782322000	9.271390000
C	2.186301000	3.245126000	8.671367000
C	3.145086000	3.832051000	9.450295000
H	4.066886000	4.196978000	9.015840000
C	-0.043067000	2.140171000	8.362987000
F	0.502687000	1.095498000	7.728893000
F	-0.445961000	3.032661000	7.450520000
F	-1.105263000	1.721138000	9.048933000
H	2.326700000	3.132810000	7.604988000

[Bn(*p*-CF_3)]$^{\cdot}$
E [Eh]: −607.940686944767

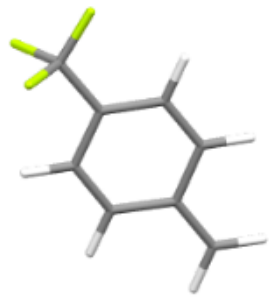

C	3.909432000	4.592651000	11.681458000
H	3.758250000	4.703404000	12.746248000
H	4.827415000	4.976580000	11.258660000
C	2.945452000	3.977698000	10.876419000
C	1.735940000	3.474306000	11.425357000
H	1.566616000	3.565302000	12.490759000
C	0.780306000	2.881149000	10.628152000
H	-0.135246000	2.507889000	11.064319000
C	0.989614000	2.763116000	9.253197000
C	2.176845000	3.241003000	8.683756000
C	3.133910000	3.833636000	9.473318000
H	4.048618000	4.203366000	9.027444000
C	-0.043306000	2.142192000	8.361449000
F	0.436754000	1.057083000	7.709339000
F	-0.461098000	3.004301000	7.404385000
F	-1.139045000	1.743951000	9.035663000
H	2.337314000	3.144342000	7.617847000

[Bn]$^+$
E [Eh]: −270.603314427858

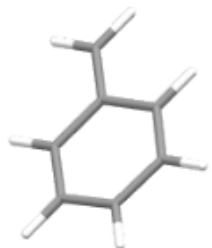

C	6.142773000	4.526672000	7.378307000
C	7.155199000	4.474286000	6.359419000
H	7.361838000	5.364433000	5.779011000
C	5.860359000	3.349483000	8.153249000
H	5.097481000	3.396113000	8.919904000
C	5.456494000	5.682327000	7.607513000
H	4.690143000	5.741628000	8.371756000
H	5.655352000	6.579972000	7.033316000
C	7.538058000	2.177405000	6.912011000
H	8.083262000	1.258902000	6.729826000
C	7.839324000	3.309779000	6.137285000
H	8.604288000	3.252944000	5.375449000
C	6.554518000	2.194167000	7.914705000
H	6.354810000	1.299360000	8.487640000

[Bn]$^{\cdot}$
E [Eh]: −270.861392574246

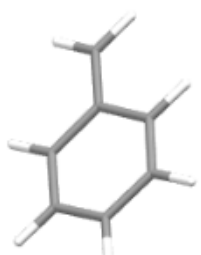

C	6.135909000	4.535609000	7.378337000
C	7.143891000	4.458346000	6.378605000
H	7.362022000	5.341251000	5.790058000
C	5.875499000	3.358330000	8.131847000
H	5.112660000	3.389848000	8.900309000
C	5.429020000	5.721435000	7.611470000
H	4.663740000	5.773603000	8.373533000
H	5.630786000	6.613810000	7.035121000
C	7.561000000	2.144548000	6.908684000
H	8.107064000	1.227829000	6.728838000
C	7.837149000	3.287183000	6.153792000
H	8.600449000	3.253439000	5.386382000
C	6.575411000	2.193002000	7.897535000
H	6.359301000	1.309236000	8.484879000

[Bn(*o*,*p*-CF_3)$_2$]$^+$
E [Eh]: −944.726720552367

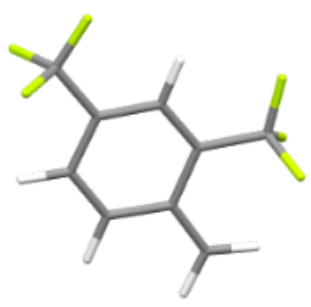

C	3.947364000	4.628886000	11.584456000
H	3.853647000	4.812581000	12.642188000
H	4.857252000	4.950457000	11.097581000
C	2.950159000	4.001095000	10.832679000
C	1.709033000	3.538229000	11.372602000
C	0.765104000	2.936590000	10.563735000
H	-0.165643000	2.596622000	10.990141000
C	1.000508000	2.766429000	9.202093000
C	2.207067000	3.200282000	8.643225000
C	3.153361000	3.799112000	9.438208000
H	4.086014000	4.135929000	9.004698000
C	-0.034422000	2.139985000	8.313457000
F	0.454150000	1.062595000	7.658957000
F	-0.459079000	3.004663000	7.363393000
F	-1.121686000	1.735112000	8.993495000

[Bn(*o*,*p*-CF_3)$_2$]$^{\cdot}$
E [Eh]: −945.015544652077

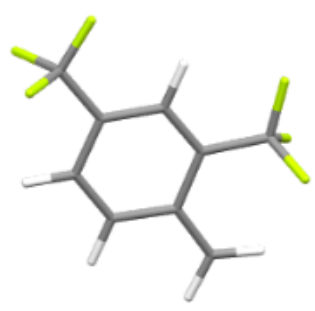

C	3.915081000	4.596176000	11.560137000
H	3.827051000	4.777804000	12.623048000
H	4.832353000	4.918530000	11.080735000
C	2.932220000	3.990610000	10.822575000
C	1.684099000	3.539338000	11.388942000
C	0.752291000	2.944643000	10.582830000
H	-0.185265000	2.597679000	10.991368000
C	1.014175000	2.782595000	9.210937000
C	2.211409000	3.206338000	8.626464000
C	3.158833000	3.801854000	9.419043000
H	4.096010000	4.139802000	8.995852000
C	-0.032682000	2.133218000	8.307772000
F	0.515173000	1.092265000	7.671698000
F	-0.442871000	3.025985000	7.399602000
F	-1.087777000	1.710847000	9.001215000

H	2.391848000	3.064843000	7.585802000
C	1.409268000	3.702828000	12.838456000
F	0.198784000	3.220533000	13.172972000
F	1.431814000	5.000397000	13.218751000
F	2.312958000	3.058879000	13.613059000

H	2.380260000	3.063299000	7.567970000
C	1.397192000	3.714470000	12.875631000
F	0.195402000	3.254451000	13.200808000
F	1.465874000	5.014343000	13.207749000
F	2.318675000	3.051805000	13.595575000

[Bn(*o*,*p*-CF_3)$_3$]$^+$
E [Eh]: −1281.785478578370

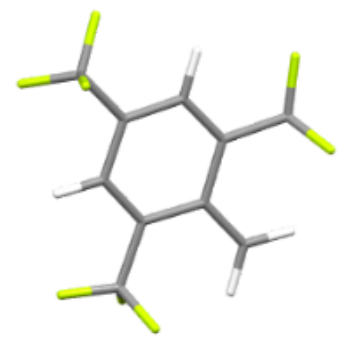

C	3.644906000	4.603741000	11.774868000
H	3.360992000	4.893329000	12.776956000
H	4.669004000	4.799485000	11.489058000
C	2.753249000	4.012662000	10.911758000
C	1.392803000	3.747276000	11.315694000
C	0.520956000	3.130939000	10.455743000
H	-0.497789000	2.933360000	10.755342000
C	0.957704000	2.757362000	9.180189000
C	2.265025000	3.003435000	8.742357000
C	3.154333000	3.622661000	9.580298000
C	0.002837000	2.015086000	8.245623000
F	0.248803000	0.703240000	8.355541000
F	0.200604000	2.386547000	6.980312000
F	-1.267356000	2.246368000	8.571805000
H	2.563287000	2.709501000	7.746385000
C	4.574661000	3.891481000	9.082964000
F	4.734859000	3.485706000	7.829582000
F	5.460898000	3.245810000	9.857834000
F	4.843298000	5.205633000	9.143985000
C	0.902481000	4.133702000	12.711446000
F	-0.379385000	3.833580000	12.876661000
F	1.061282000	5.450996000	12.911748000
F	1.618178000	3.478631000	13.640052000

[Bn(*o*,*p*-CF_3)$_3$]$^{\cdot}$
E [Eh]: −1282.087248791590

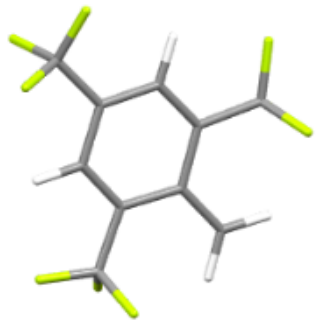

C	3.668535000	4.652599000	11.796158000
H	3.366452000	4.984521000	12.774878000
H	4.692817000	4.827295000	11.513708000
C	2.771417000	4.021023000	10.931451000
C	1.406229000	3.776406000	11.296391000
C	0.532954000	3.138162000	10.441338000
H	-0.487014000	2.965897000	10.748066000
C	0.959468000	2.705008000	9.190136000
C	2.275938000	2.927932000	8.790356000
C	3.162809000	3.566738000	9.626940000
C	0.004296000	2.036436000	8.242320000
F	0.596442000	1.027162000	7.572262000
F	-0.459996000	2.902789000	7.313282000
F	-1.066784000	1.528764000	8.877737000
H	2.604099000	2.592362000	7.818188000
C	4.573929000	3.784879000	9.133102000
F	4.746391000	3.341568000	7.876171000
F	5.482742000	3.147645000	9.903477000
F	4.909474000	5.093735000	9.133731000
C	0.888774000	4.209218000	12.648110000
F	-0.412380000	3.915031000	12.812871000
F	1.013608000	5.540571000	12.838091000
F	1.555430000	3.604788000	13.657434000

[KBn]$^+$
E [Eh]: −383.904775853448

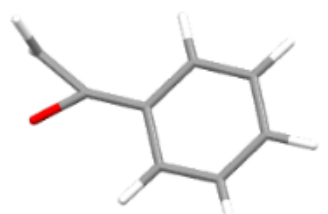

C	5.911176000	4.530227000	7.636550000
C	5.890035000	3.564274000	8.630489000
C	5.482707000	5.740488000	7.003763000
C	6.762548000	2.449415000	8.554211000
H	4.611574000	5.733222000	6.361045000
H	5.898640000	6.689807000	7.316195000
O	6.559603000	4.738484000	6.555292000
C	4.990231000	3.714245000	9.713324000
C	4.969501000	2.757709000	10.703630000
H	4.332751000	4.572808000	9.752492000
C	6.723895000	1.505535000	9.553254000
C	5.832060000	1.661095000	10.621368000
H	4.291373000	2.851639000	11.540168000
H	7.379573000	0.646747000	9.516987000
H	5.809534000	0.912274000	11.402994000
H	7.442508000	2.354049000	7.717896000

[KBn]$^{\cdot}$
E [Eh]: −384.179453538347

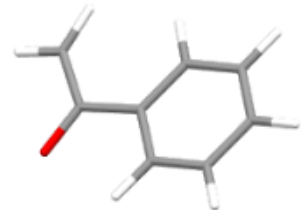

C	6.208253000	4.619378000	7.454245000
C	6.007494000	3.612996000	8.545238000
C	5.278550000	5.696605000	7.277611000
C	6.828891000	2.482408000	8.553420000
H	4.384076000	5.817896000	7.869802000
H	5.481189000	6.403404000	6.485460000
O	7.171596000	4.521554000	6.683684000
C	5.051646000	3.760861000	9.553399000
C	4.919683000	2.799100000	10.546953000
H	4.412810000	4.632583000	9.582802000
C	6.693068000	1.519582000	9.540770000
C	5.737525000	1.675699000	10.541300000
H	4.179202000	2.928143000	11.325799000
H	7.332160000	0.645901000	9.533409000
H	5.632375000	0.924623000	11.313838000
H	7.569192000	2.381286000	7.771930000

$[F_2Bn]^{+}$
E [Eh]: −469.113902480686

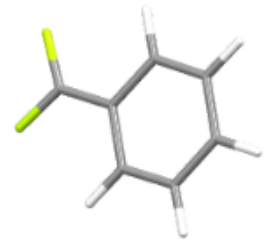

C	5.784213000	4.840079000	7.309691000
C	6.981658000	4.304394000	6.759109000
H	7.833678000	4.947308000	6.586551000
C	4.649838000	4.013361000	7.542245000
H	3.745613000	4.435030000	7.959356000
C	5.730203000	6.187609000	7.628288000
F	4.704296000	6.760068000	8.127025000
F	6.706786000	6.991963000	7.456735000
C	5.912703000	2.158685000	6.691368000
H	5.964210000	1.104110000	6.450843000
C	7.032244000	2.966423000	6.455429000
H	7.931858000	2.537503000	6.036987000
C	4.728481000	2.678359000	7.229650000
H	3.880809000	2.029007000	7.398083000

$[F_2Bn]^{\cdot}$
E [Eh]: −469.362664247421

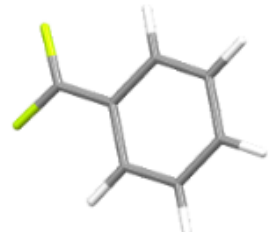

C	5.782169000	4.858087000	7.307613000
C	6.968447000	4.294422000	6.762472000
H	7.826328000	4.929061000	6.590221000
C	4.662067000	4.009608000	7.525130000
H	3.752157000	4.424952000	7.935146000
C	5.719982000	6.211254000	7.610364000
F	4.679821000	6.802638000	8.176877000
F	6.737310000	7.047171000	7.476809000
C	5.914893000	2.123408000	6.684319000
H	5.967193000	1.069394000	6.447314000
C	7.019452000	2.949944000	6.462855000
H	7.930105000	2.532913000	6.051885000
C	4.742881000	2.668635000	7.214523000
H	3.883784000	2.032414000	7.385832000

$[Bn(F)_5]^{+}$
E [Eh]: −766.800183313073

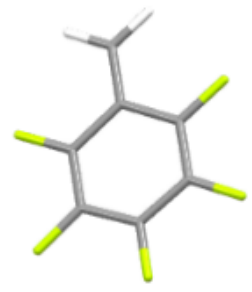

C	6.123575000	4.559214000	7.378109000
C	7.141012000	4.480352000	6.353760000
F	7.396279000	5.544243000	5.638367000
C	5.861517000	3.371824000	8.160313000
F	4.945309000	3.425918000	9.091478000
C	5.434409000	5.706099000	7.599393000
H	4.673339000	5.756980000	8.367858000
H	5.628228000	6.596063000	7.014270000
C	7.533415000	2.193586000	6.926680000
F	8.191154000	1.100783000	6.715554000
C	7.830234000	3.319890000	6.130024000
F	8.755491000	3.219087000	5.203759000
C	6.551726000	2.211013000	7.940447000
F	6.328212000	1.122420000	8.639376000

$[Bn(F)_5]^{\cdot}$
E [Eh]: −767.082913277913

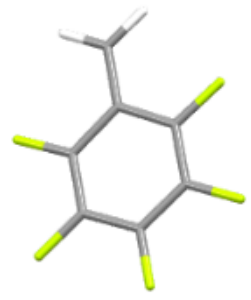

C	6.118984000	4.565841000	7.379795000
C	7.120359000	4.461094000	6.379966000
F	7.399203000	5.534058000	5.631779000
C	5.880757000	3.386934000	8.132494000
F	4.948027000	3.413519000	9.090600000
C	5.414474000	5.747606000	7.609682000
H	4.660734000	5.792562000	8.379408000
H	5.615242000	6.627501000	7.019702000
C	7.549655000	2.164898000	6.921007000
F	8.225431000	1.039964000	6.703628000
C	7.818825000	3.296653000	6.151576000
F	8.753169000	3.232865000	5.201169000
C	6.572432000	2.216117000	7.915045000
F	6.316609000	1.127858000	8.643538000

$[ABn]^{+}$
E [Eh]: −344.643625076268

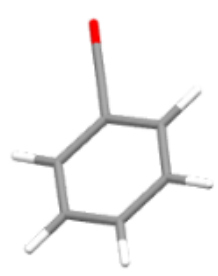

C	6.138687000	4.491059000	7.415341000
C	7.047774000	4.470731000	6.329008000
H	7.186298000	5.346823000	5.710758000
C	5.927030000	3.365331000	8.249392000
H	5.226530000	3.414221000	9.071507000
C	5.431098000	5.644963000	7.672123000
C	7.537855000	2.189356000	6.907655000
H	8.089929000	1.280253000	6.706886000
C	7.740954000	3.304781000	6.090830000
H	8.441624000	3.256374000	5.268892000
C	6.639513000	2.218680000	7.977394000
H	6.500656000	1.342797000	8.595740000
O	4.850282000	6.585511000	7.884225000

$[ABn]^{\cdot}$
E [Eh]: −344.875626071151

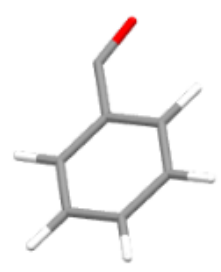

C	6.179142000	4.563600000	7.323322000
C	7.088459000	4.448124000	6.272825000
H	7.252192000	5.298277000	5.623348000
C	5.948838000	3.478837000	8.178094000
H	5.240139000	3.586831000	8.988656000
C	5.463008000	5.839437000	7.521781000
C	7.536544000	2.177366000	6.922539000
H	8.065374000	1.245533000	6.766877000
C	7.767259000	3.253856000	6.072942000
H	8.473024000	3.160800000	5.257982000
C	6.628918000	2.289717000	7.974523000
H	6.455915000	1.447634000	8.632402000
O	4.659417000	6.120870000	8.344460000

$[Bn(\textit{o}\text{-}CF_3)]^+$
E [Eh]: −607.664983675893

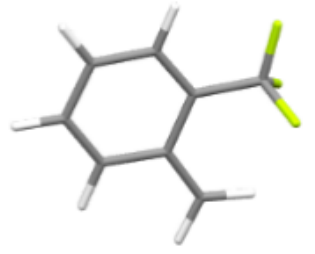

C	3.912981000	4.593000000	11.558129000
H	3.827231000	4.773236000	12.621277000
H	4.829986000	4.914679000	11.078114000
C	2.930274000	3.989803000	10.822008000
C	1.679908000	3.537671000	11.388557000
C	0.753279000	2.941339000	10.580147000
H	-0.184720000	2.595018000	10.989422000
C	1.017203000	2.776207000	9.206118000
C	2.212640000	3.203424000	8.623856000
C	3.158913000	3.801797000	9.417557000
H	4.097214000	4.142975000	8.999119000
H	2.384647000	3.063265000	7.565877000
C	1.394008000	3.717063000	12.873138000
F	0.190937000	3.258757000	13.201064000
F	1.462490000	5.017674000	13.204935000
F	2.313785000	3.055648000	13.597710000
H	0.264683000	2.302703000	8.587193000

$[Bn(\textit{o}\text{-}CF_3)]^{\cdot}$
E [Eh]: −607.938328506440

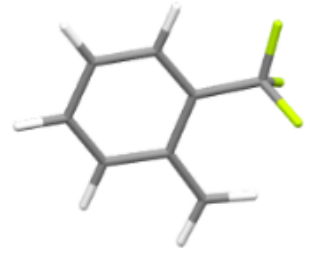

C	3.948422000	4.616744000	11.580396000
H	3.860691000	4.795573000	12.639436000
H	4.859750000	4.932056000	11.091900000
C	2.944081000	3.998252000	10.827771000
C	1.700071000	3.540377000	11.368565000
C	0.756751000	2.937804000	10.557281000
H	-0.174308000	2.600521000	10.988923000
C	0.989711000	2.760558000	9.194582000
C	2.192601000	3.197022000	8.639193000
C	3.143404000	3.798769000	9.433673000
H	4.076587000	4.136520000	9.000875000
H	2.380960000	3.063500000	7.581602000
C	1.403748000	3.710461000	12.832450000
F	0.189394000	3.236288000	13.173122000
F	1.432770000	5.008835000	13.214437000
F	2.302395000	3.063407000	13.612947000
H	0.238433000	2.287574000	8.577067000

$[BnPh]^+$
E [Eh]: −501.653527888237

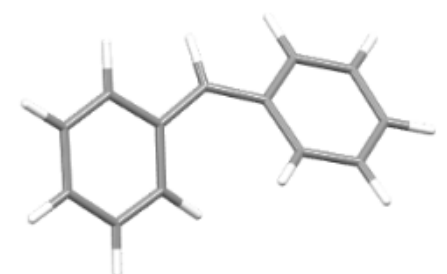

C	6.208790000	4.073846000	7.475356000
C	7.551401000	3.652992000	7.663573000
H	8.262209000	4.313037000	8.136950000
C	5.335589000	3.246863000	6.719928000
H	4.319952000	3.577425000	6.543012000
C	5.684483000	5.287480000	7.964658000
C	7.074867000	1.617672000	6.476159000
H	7.414922000	0.665596000	6.089310000
C	7.971237000	2.440424000	7.166463000
H	8.999809000	2.130508000	7.290424000
C	5.758962000	2.022960000	6.250188000
H	5.081655000	1.386744000	5.697548000
C	6.163725000	6.160089000	8.962734000
H	4.759379000	5.605044000	7.490932000
C	7.115148000	5.794851000	9.950625000
C	7.546585000	6.723082000	10.870501000
H	7.450116000	4.771437000	10.024843000
C	5.630970000	7.476265000	8.983652000
H	8.255097000	6.438820000	11.636375000
H	4.879463000	7.751509000	8.254676000
C	7.051731000	8.030825000	10.831019000
C	6.091514000	8.405450000	9.890642000
H	7.399892000	8.754566000	11.556737000
H	5.701464000	9.413795000	9.884246000

$[BnPh]^{\cdot}$
E [Eh]: −501.884065411340

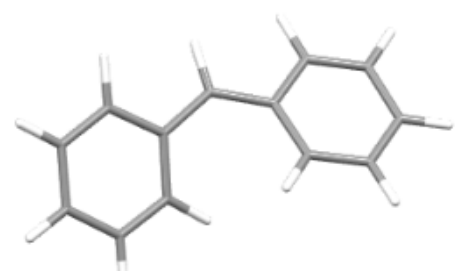

C	6.258093000	4.056646000	7.450772000
C	7.567144000	3.612110000	7.749823000
H	8.236305000	4.253824000	8.302714000
C	5.465361000	3.215692000	6.633053000
H	4.465920000	3.540747000	6.369722000
C	5.710390000	5.306044000	7.871262000
C	7.215134000	1.566750000	6.516186000
H	7.582134000	0.612122000	6.162695000
C	8.028709000	2.391268000	7.290561000
H	9.039441000	2.082045000	7.526386000
C	5.930282000	1.994498000	6.184780000
H	5.293347000	1.370259000	5.570684000
C	6.137720000	6.196335000	8.902659000
H	4.833810000	5.630696000	7.320151000
C	6.991767000	5.834800000	9.970109000
C	7.349887000	6.754047000	10.940615000
H	7.331129000	4.814061000	10.060688000
C	5.640695000	7.521699000	8.897514000
H	7.998712000	6.446292000	11.751286000
H	4.967064000	7.819805000	8.103104000
C	6.874799000	8.062477000	10.892334000
C	6.009322000	8.436274000	9.865044000
H	7.161475000	8.776760000	11.653064000
H	5.620321000	9.446028000	9.825343000

$[BnPh_2]^+$
E [Eh]: −732.687470731511

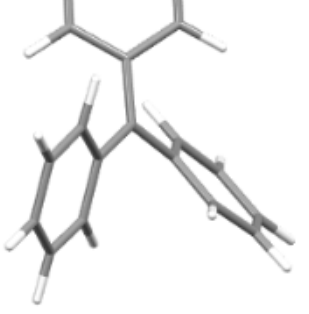

C	6.153037000	4.066544000	7.550172000
C	7.513911000	4.240168000	7.206156000
H	8.008885000	5.170381000	7.445186000

$[BnPh_2]^{\cdot}$
E [Eh]: −732.903044408004

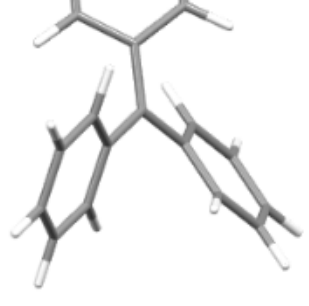

C	6.147426000	4.055126000	7.598716000
C	7.469521000	4.262932000	7.157823000
H	7.948874000	5.210764000	7.360760000

C	5.509347000	2.861731000	7.182124000
H	4.486838000	2.692594000	7.486489000
C	5.448668000	5.091336000	8.265528000
C	7.537938000	2.075470000	6.163464000
H	8.075668000	1.304718000	5.626722000
C	8.190224000	3.258141000	6.510848000
H	9.223111000	3.409620000	6.228440000
C	6.200420000	1.877495000	6.504996000
H	5.707905000	0.948748000	6.251224000
C	6.156199000	5.968402000	9.169781000
C	4.020558000	5.244066000	8.144813000
C	7.165799000	5.468068000	10.017175000
C	7.785428000	6.301365000	10.929790000
H	7.405510000	4.414015000	9.995724000
C	5.804523000	7.331689000	9.257215000
H	8.530243000	5.903104000	11.605330000
H	5.043801000	7.726429000	8.598326000
C	7.447153000	7.652220000	10.981633000
C	6.464628000	8.167384000	10.137293000
H	7.946757000	8.305718000	11.684814000
H	6.215549000	9.219200000	10.173067000
C	3.271864000	5.616818000	9.284174000
C	1.895386000	5.711478000	9.214508000
C	3.343048000	5.004330000	6.930262000
C	1.971246000	5.140957000	6.861552000
C	1.245551000	5.481192000	8.003902000
H	3.787124000	5.775023000	10.220876000
H	1.323717000	5.962809000	10.097507000
H	0.168502000	5.569305000	7.945029000
H	1.454034000	4.990431000	5.923759000
H	3.912339000	4.764251000	6.043136000

C	5.549917000	2.816335000	7.291897000
H	4.543080000	2.619940000	7.633682000
C	5.426785000	5.078342000	8.340404000
C	7.541977000	2.070184000	6.158810000
H	8.077001000	1.308743000	5.606232000
C	8.150839000	3.288659000	6.447389000
H	9.160790000	3.482909000	6.108344000
C	6.237317000	1.841753000	6.588042000
H	5.756793000	0.894015000	6.379466000
C	6.143226000	5.961058000	9.249400000
C	3.987893000	5.221246000	8.174957000
C	7.253067000	5.507629000	9.989321000
C	7.927605000	6.348257000	10.859186000
H	7.565624000	4.476950000	9.890419000
C	5.751651000	7.302777000	9.425233000
H	8.767335000	5.967351000	11.427007000
H	4.914414000	7.684268000	8.857032000
C	7.525776000	7.672294000	11.014241000
C	6.434608000	8.142869000	10.288837000
H	8.055960000	8.328849000	11.691974000
H	6.119776000	9.173847000	10.391886000
C	3.163688000	5.611033000	9.249096000
C	1.794046000	5.743102000	9.092364000
C	3.364007000	4.975911000	6.935932000
C	1.994981000	5.116675000	6.781114000
C	1.198942000	5.498767000	7.857597000
H	3.610927000	5.788193000	10.217629000
H	1.184357000	6.030290000	9.939987000
H	0.128800000	5.605248000	7.735535000
H	1.545319000	4.935448000	5.812822000
H	3.972588000	4.693438000	6.087886000

[Bn(*o*-Me)]$^{+}$
E [Eh]: −309.917697641101

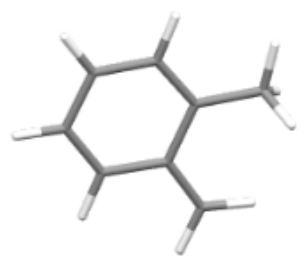

C	3.906005000	4.590709000	11.564462000
H	3.804620000	4.768417000	12.627063000
H	4.829273000	4.916818000	11.100748000
C	2.932297000	3.989666000	10.829554000
C	1.683296000	3.544826000	11.429818000
C	0.757436000	2.952217000	10.596204000
H	-0.184464000	2.608086000	11.001495000
C	1.013366000	2.785845000	9.232727000
C	2.214875000	3.206307000	8.634635000
C	3.160342000	3.798880000	9.417758000
H	4.099043000	4.138347000	8.999012000
H	2.375866000	3.059149000	7.576100000
C	1.403315000	3.717587000	12.885337000
H	0.425465000	3.315458000	13.138788000
H	1.418294000	4.773014000	13.168259000
H	2.149138000	3.203969000	13.496650000
H	0.257293000	2.314965000	8.615613000

[Bn(*o*-Me)]$^{-}$
E [Eh]: −310.169293385238

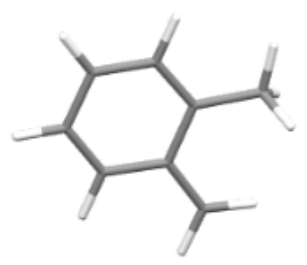

C	3.924298000	4.614677000	11.601973000
H	3.810486000	4.783956000	12.661640000
H	4.846274000	4.943164000	11.142162000
C	2.933230000	3.996764000	10.832696000
C	1.696763000	3.544459000	11.399237000
C	0.761709000	2.941867000	10.574953000
H	-0.172599000	2.601312000	11.006011000
C	0.987511000	2.761702000	9.209730000
C	2.187438000	3.196527000	8.644962000
C	3.138195000	3.800004000	9.438371000
H	4.071216000	4.138956000	9.004463000
H	2.371564000	3.060700000	7.586757000
C	1.417475000	3.718314000	12.864163000
H	0.431548000	3.330351000	13.118314000
H	1.451349000	4.771401000	13.156398000
H	2.155235000	3.193180000	13.477171000
H	0.233767000	2.286928000	8.595219000

Ligands

Py
E [Eh]: −248.241610833583

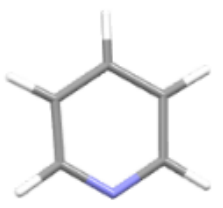

N	5.884830000	7.079467000	6.188783000
C	7.005794000	7.764488000	5.961973000
H	7.710400000	7.815355000	6.785700000
C	7.291422000	8.391076000	4.753897000
H	8.219122000	8.933395000	4.626450000
C	4.999834000	7.001925000	5.194076000
C	6.362486000	8.302499000	3.726088000
H	6.548807000	8.775588000	2.770174000
C	5.190219000	7.592940000	3.950130000
H	4.094827000	6.440066000	5.401173000
H	4.436658000	7.497491000	3.179586000

$(Py)^{\cdot+}$
E [Eh]: −247.912374324176

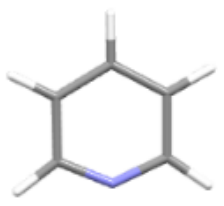

N	5.911989000	7.149528000	6.047424000
C	7.054296000	7.779475000	5.985689000
H	7.720439000	7.796154000	6.840833000
C	7.307304000	8.398337000	4.755902000
H	8.236625000	8.939209000	4.634435000
C	4.949638000	6.981174000	5.180788000
C	6.358667000	8.293143000	3.745270000
H	6.544214000	8.767729000	2.789551000
C	5.176084000	7.589266000	3.940483000
H	4.066275000	6.410440000	5.443881000
H	4.418867000	7.489834000	3.173775000

L1
E [Eh]: −1693.615711248744

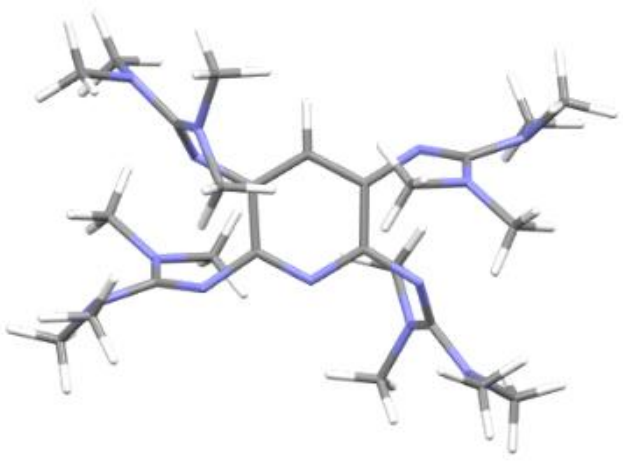

N	11.039016000	-2.626326000	-0.539326000
N	11.503110000	-4.792950000	0.084287000
N	12.060463000	-3.096376000	1.573249000
N	9.117745000	-2.123483000	1.552411000
N	7.407821000	-2.946005000	2.865787000
N	6.886502000	-2.243320000	0.712603000
N	8.945959000	0.089414000	0.810000000
C	10.542839000	-1.350817000	-0.265616000
C	9.522603000	-1.109946000	0.679624000
C	11.501451000	-3.429467000	0.352979000
C	10.707511000	-5.254654000	-1.035260000
H	11.195805000	-5.067923000	-1.999871000
H	10.543449000	-6.329321000	-0.926868000
H	9.750308000	-4.741023000	-1.041147000
C	12.700849000	-5.580777000	0.323449000
H	13.323709000	-5.099184000	1.070649000
H	12.436967000	-6.581196000	0.676443000
H	13.290388000	-5.689460000	-0.597448000
C	11.745543000	-3.849582000	2.773622000
H	11.191647000	-4.746417000	2.512643000
H	12.657947000	-4.136010000	3.308206000
H	11.123882000	-3.245663000	3.439507000
C	12.557045000	-1.759896000	1.815904000
H	11.792522000	-1.098544000	2.237897000
H	13.391227000	-1.820299000	2.521000000
H	12.917591000	-1.323876000	0.888249000
C	7.866489000	-2.401127000	1.685130000
C	8.244682000	-2.877866000	4.043235000
H	8.861233000	-1.984914000	3.995875000
H	7.609384000	-2.840662000	4.932176000
H	8.904761000	-3.749912000	4.129821000
C	6.402729000	-3.990722000	2.888960000
H	6.854780000	-4.954053000	3.158311000
H	5.623332000	-3.767896000	3.623940000
H	5.945401000	-4.085860000	1.908841000
C	7.260453000	-2.243068000	-0.687608000
H	8.145194000	-2.858148000	-0.833958000
H	6.435960000	-2.666020000	-1.268811000
H	7.477190000	-1.242128000	-1.069665000
C	5.677726000	-1.500328000	1.022361000

$(L1)^{\cdot+}$
E [Eh]: −1693.444124803905

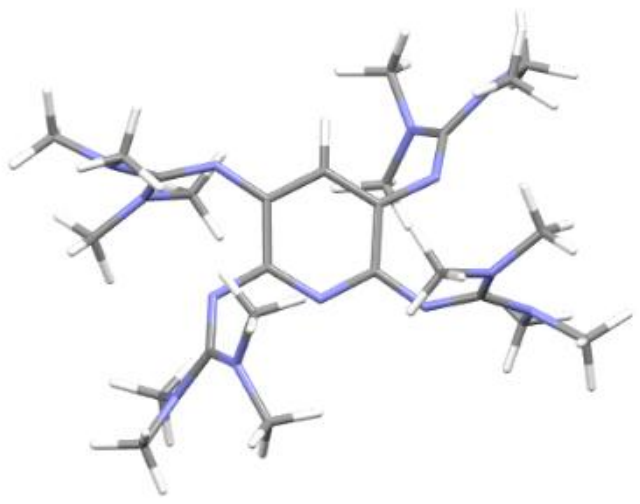

N	11.332625000	-2.439696000	-0.526888000
N	11.485407000	-4.695251000	-0.103274000
N	12.106337000	-3.203016000	1.567878000
N	9.144814000	-2.284371000	1.278247000
N	7.609078000	-2.863829000	2.909632000
N	6.836002000	-2.343314000	0.786625000
N	8.794292000	-0.068991000	0.663006000
C	10.691298000	-1.281713000	-0.279747000
C	9.506043000	-1.195508000	0.566043000
C	11.598122000	-3.406725000	0.315772000
C	10.693966000	-4.977424000	-1.288791000
H	11.283129000	-4.866331000	-2.204674000
H	10.325400000	-6.002355000	-1.228680000
H	9.849714000	-4.296854000	-1.337818000
C	12.462420000	-5.718889000	0.246577000
H	13.241820000	-5.297977000	0.874217000
H	11.998061000	-6.558392000	0.769347000
H	12.928267000	-6.099588000	-0.666275000
C	11.776420000	-4.084140000	2.678771000
H	11.050518000	-4.826364000	2.360927000
H	12.660972000	-4.589533000	3.074988000
H	11.329641000	-3.493495000	3.479910000
C	12.624455000	-1.901789000	1.942266000
H	11.846172000	-1.251086000	2.355417000
H	13.399254000	-2.037401000	2.698380000
H	13.064619000	-1.415846000	1.075524000
C	7.886797000	-2.456914000	1.650616000
C	8.555620000	-2.631497000	3.985665000
H	9.292345000	-1.901219000	3.668492000
H	8.022595000	-2.251491000	4.860026000
H	9.064160000	-3.557529000	4.268435000
C	6.476977000	-3.713785000	3.245429000
H	6.837855000	-4.575369000	3.812445000
H	5.740847000	-3.188167000	3.858672000
H	5.995880000	-4.070035000	2.339561000
C	7.060770000	-2.386792000	-0.646513000
H	7.915595000	-3.020012000	-0.867591000
H	6.175450000	-2.807882000	-1.125253000
H	7.244938000	-1.391378000	-1.058911000
C	5.597064000	-1.685301000	1.184925000

H	5.779959000	-0.451091000	0.720519000
H	4.818894000	-1.934098000	0.501574000
H	5.488745000	-1.525003000	2.092050000
N	8.807833000	2.376494000	0.374125000
N	8.286404000	4.540538000	-0.224765000
N	7.714561000	2.836486000	-1.700426000
N	10.748177000	2.070226000	-1.707472000
N	12.455466000	3.260166000	-2.711042000
N	12.841675000	2.367479000	-0.595485000
C	10.926691000	-0.270187000	-1.055880000
C	9.330859000	1.123186000	0.060583000
C	10.354895000	0.988234000	-0.901861000
C	8.305922000	3.178400000	-0.496288000
C	9.097980000	5.010978000	0.880174000
H	8.653734000	4.775143000	1.854746000
H	9.206874000	6.094371000	0.794275000
H	10.080052000	4.548287000	0.839361000
C	7.064583000	5.302814000	-0.425485000
H	6.438867000	4.820210000	-1.169516000
H	7.299134000	6.314989000	-0.764858000
H	6.490750000	5.380457000	0.508200000
C	7.995529000	3.586020000	-2.911018000
H	8.510534000	4.509199000	-2.662672000
H	7.070158000	3.825194000	-3.445925000
H	8.642609000	3.001191000	-3.571590000
C	7.234330000	1.490622000	-1.922570000
H	7.992528000	0.843528000	-2.377770000
H	6.369062000	1.532232000	-2.590688000
H	6.925837000	1.050131000	-0.978460000
C	11.949953000	2.523452000	-1.649135000
C	11.718312000	3.253014000	-3.957880000
H	11.288601000	2.268869000	-4.121872000
H	12.402029000	3.497184000	-4.774433000
H	10.900943000	3.984697000	-3.959494000
C	13.187702000	4.490215000	-2.462749000
H	12.525495000	5.363735000	-2.537183000
H	13.989910000	4.610377000	-3.195234000
H	13.620699000	4.474746000	-1.467410000
C	12.372891000	2.203352000	0.766204000
H	11.376875000	2.622309000	0.877063000
H	13.054629000	2.737089000	1.436105000
H	12.335495000	1.154299000	1.075052000
C	14.208780000	1.946838000	-0.828439000
H	14.348007000	0.902020000	-0.521461000
H	14.912817000	2.561670000	-0.257321000
H	14.449905000	2.029739000	-1.884784000
H	11.686801000	-0.422494000	-1.812901000

H	5.504784000	-0.741826000	0.640199000
H	4.727242000	-2.308306000	0.964742000
H	5.614259000	-1.454288000	2.244739000
N	8.438122000	2.154431000	0.225933000
N	8.125007000	4.379767000	-0.276727000
N	7.676443000	2.787298000	-1.911731000
N	10.721372000	2.240467000	-1.421364000
N	12.399374000	3.378209000	-2.527371000
N	12.879546000	2.432969000	-0.460059000
C	11.067476000	-0.113277000	-0.949522000
C	9.156215000	1.039794000	0.011291000
C	10.364155000	1.071801000	-0.805970000
C	8.127168000	3.072469000	-0.652289000
C	8.854586000	4.756499000	0.921823000
H	8.269782000	4.574746000	1.829063000
H	9.096802000	5.818305000	0.864081000
H	9.775849000	4.185265000	0.983448000
C	7.071472000	5.302716000	-0.680109000
H	6.372263000	4.807846000	-1.346765000
H	7.480249000	6.182665000	-1.182381000
H	6.521972000	5.637125000	0.204385000
C	7.988782000	3.642324000	-3.046763000
H	8.568628000	4.498892000	-2.719119000
H	7.082300000	3.990684000	-3.548540000
H	8.589216000	3.080007000	-3.765565000
C	7.270874000	1.437473000	-2.253784000
H	8.107170000	0.836385000	-2.627559000
H	6.508481000	1.488543000	-3.032803000
H	6.848583000	0.948177000	-1.380223000
C	11.962886000	2.645887000	-1.464784000
C	11.657686000	3.356997000	-3.774533000
H	11.103369000	2.427038000	-3.853942000
H	12.358448000	3.435193000	-4.607992000
H	10.952830000	4.191931000	-3.837015000
C	13.341384000	4.479183000	-2.386903000
H	12.867388000	5.408457000	-2.715940000
H	14.235504000	4.320768000	-2.994861000
H	13.635192000	4.592850000	-1.348104000
C	12.459016000	2.210662000	0.909700000
H	11.461506000	2.611128000	1.066149000
H	13.151892000	2.725182000	1.579479000
H	12.447396000	1.148078000	1.170736000
C	14.263846000	2.086667000	-0.741071000
H	14.471674000	1.078665000	-0.366407000
H	14.958806000	2.778942000	-0.257962000
H	14.444926000	2.095339000	-1.811730000
H	11.938039000	-0.151380000	-1.591717000

L2a
E [Eh]: −968.549554244415

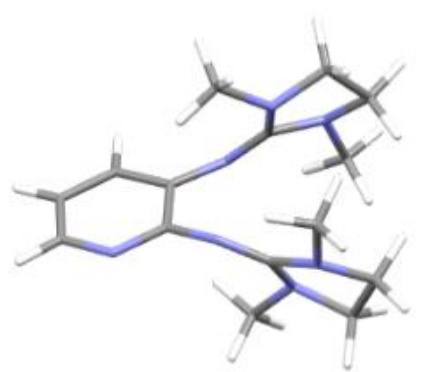

N	0.320368000	5.206257000	9.494909000
N	-0.812201000	4.295643000	11.300712000
N	-2.025360000	4.585221000	9.443014000
N	-0.088997000	3.389016000	7.227448000
N	-0.685361000	1.336520000	8.128606000
N	1.187133000	2.246641000	8.951748000
N	1.104298000	6.793123000	8.014914000
C	0.611497000	5.563790000	8.193168000
C	0.526885000	4.627095000	7.118353000
C	0.948143000	5.062983000	5.863353000
H	0.862903000	4.376700000	5.030005000
C	1.456966000	6.342910000	5.694603000
H	1.797676000	6.687809000	4.727184000
C	1.509765000	7.171841000	6.804859000
H	1.893287000	8.184167000	6.725112000
C	-0.757647000	4.741039000	9.986874000
C	-2.065299000	3.616852000	11.551616000

$(L2a)^{\cdot+}$
E [Eh]: −968.332033573364

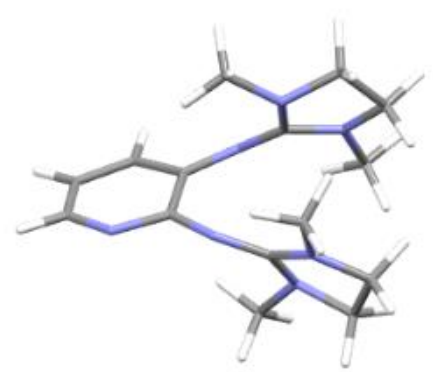

N	0.240796000	5.285765000	9.446349000
N	-0.771505000	4.121854000	11.171360000
N	-2.055277000	4.568510000	9.412170000
N	0.071580000	3.365589000	7.193436000
N	-0.744838000	1.492816000	8.288556000
N	1.229926000	2.236100000	8.977419000
N	1.266601000	6.748333000	8.037429000
C	0.649105000	5.556010000	8.211972000
C	0.520942000	4.613772000	7.096280000
C	0.831002000	5.114070000	5.802173000
H	0.664952000	4.459585000	4.957251000
C	1.371518000	6.357761000	5.662874000
H	1.648852000	6.746544000	4.692837000
C	1.610072000	7.127158000	6.831335000
H	2.101622000	8.091150000	6.750817000
C	-0.794642000	4.674991000	9.938340000
C	-2.053451000	3.523765000	11.508806000

H	-2.415831000	3.782522000	12.571540000
H	-1.972943000	2.531546000	11.392713000
C	-2.969632000	4.253291000	10.501854000
H	-3.745248000	3.578060000	10.137612000
H	-3.453874000	5.157118000	10.897116000
C	0.383074000	3.907952000	12.005327000
H	0.638620000	2.854041000	11.830637000
H	0.247179000	4.061904000	13.077406000
H	1.203715000	4.526747000	11.651528000
C	-2.504523000	5.414377000	8.355693000
H	-2.625843000	6.460986000	8.661246000
H	-3.472046000	5.030024000	8.031842000
H	-1.825951000	5.367474000	7.511216000
C	0.156039000	2.441567000	8.046541000
C	-0.345939000	0.539217000	9.289902000
H	-0.507655000	-0.524369000	9.108990000
H	-0.944950000	0.834237000	10.164752000
C	1.122740000	0.893493000	9.486034000
H	1.434749000	0.868491000	10.531312000
H	1.772541000	0.214857000	8.915852000
C	-2.061705000	1.429440000	7.707406000
H	-2.701682000	1.853205000	8.492900000
H	-2.433027000	0.436743000	7.447276000
H	-2.113817000	2.075842000	6.835477000
C	2.501631000	2.830415000	8.788416000
H	3.085663000	2.304422000	8.021668000
H	3.033051000	2.761875000	9.738245000
H	2.427730000	3.877525000	8.520850000

H	-2.336597000	3.754356000	12.535490000
H	-2.013391000	2.433517000	11.403927000
C	-2.982213000	4.166744000	10.477066000
H	-3.739470000	3.483012000	10.095996000
H	-3.486566000	5.048296000	10.887218000
C	0.424216000	3.913882000	11.952875000
H	0.765685000	2.875905000	11.896836000
H	0.226225000	4.159685000	12.996507000
H	1.205248000	4.567711000	11.572354000
C	-2.563967000	5.456642000	8.381244000
H	-2.836980000	6.433021000	8.794534000
H	-3.448728000	5.002582000	7.936833000
H	-1.830231000	5.598125000	7.595315000
C	0.185172000	2.458750000	8.130127000
C	-0.387764000	0.591340000	9.374411000
H	-0.576261000	-0.446169000	9.100395000
H	-0.973318000	0.818172000	10.272241000
C	1.099083000	0.897449000	9.564220000
H	1.407962000	0.899646000	10.608496000
H	1.729705000	0.187576000	9.018596000
C	-2.066281000	1.523788000	7.706319000
H	-2.809058000	1.888747000	8.421344000
H	-2.347126000	0.519498000	7.388934000
H	-2.052619000	2.183190000	6.842199000
C	2.566942000	2.769415000	8.788058000
H	3.144154000	2.151535000	8.092697000
H	3.076735000	2.784684000	9.750606000
H	2.528328000	3.785746000	8.413607000

L2b
E [Eh]: −970.926506732262

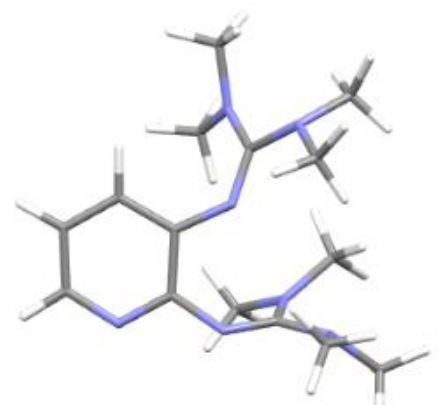

N	11.794102000	6.905081000	7.292443000
N	12.208220000	6.228606000	9.480346000
N	13.689821000	7.732997000	10.550926000
N	12.907539000	5.824662000	11.639311000
N	9.652983000	9.079137000	12.396623000
N	10.517580000	8.174971000	10.467931000
N	10.333044000	10.554935000	10.727550000
C	9.938354000	8.781147000	8.151758000
H	9.185035000	9.482437000	8.489033000
C	12.892137000	6.603258000	10.501697000
C	10.137101000	8.558519000	6.793500000
H	9.559960000	9.090747000	6.048636000
C	11.602855000	7.097279000	8.598816000
C	11.089357000	7.624568000	6.418517000
H	11.286927000	7.427662000	5.369514000
C	10.690753000	8.083674000	9.092942000
C	10.192282000	9.230971000	11.123554000
C	13.796778000	8.548919000	11.741816000
H	13.428598000	9.561934000	11.548770000
H	14.833840000	8.630954000	12.087543000
H	13.194880000	8.115838000	12.535884000
C	14.250623000	8.280975000	9.333284000
H	14.280202000	7.511504000	8.566117000
H	15.268690000	8.628890000	9.532626000
H	13.675363000	9.124124000	8.937110000
C	11.866462000	4.823986000	11.776751000
H	10.934984000	5.224173000	11.387273000
H	11.751128000	4.579879000	12.835616000
H	12.102707000	3.905028000	11.227502000
C	9.308286000	11.545569000	10.992158000
H	8.864965000	11.894561000	10.051234000
H	9.719830000	12.419061000	11.510274000
H	8.523151000	11.112830000	11.605435000
C	9.246032000	7.741262000	12.786637000
H	8.722767000	7.260008000	11.965388000

$(L2b)^{\cdot+}$
E [Eh]: −970.714864066229

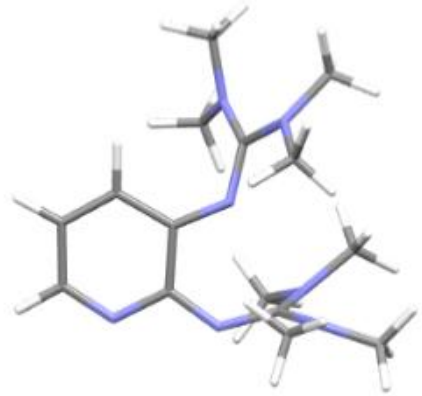

N	11.623549000	6.839271000	7.195052000
N	12.636354000	6.489542000	9.204572000
N	13.992899000	7.747633000	10.603155000
N	12.865309000	5.873281000	11.424650000
N	9.858021000	9.031492000	12.386669000
N	10.886478000	8.239199000	10.490754000
N	10.405506000	10.550348000	10.709638000
C	9.759954000	8.608027000	8.345018000
H	9.017137000	9.259505000	8.786192000
C	13.110051000	6.734964000	10.426319000
C	9.712364000	8.291012000	7.014431000
H	8.942856000	8.692707000	6.369095000
C	11.705124000	7.123820000	8.525777000
C	10.683832000	7.401105000	6.484102000
H	10.656857000	7.148221000	5.428958000
C	10.769598000	8.054309000	9.165042000
C	10.372778000	9.266186000	11.159130000
C	14.085144000	8.475651000	11.862035000
H	13.901511000	9.535528000	11.669477000
H	15.071795000	8.377609000	12.319004000
H	13.329083000	8.121594000	12.555880000
C	14.598703000	8.396890000	9.447125000
H	14.703650000	7.683165000	8.634890000
H	15.583928000	8.763908000	9.733906000
H	14.003998000	9.243881000	9.092768000
C	11.747286000	4.945334000	11.295961000
H	10.924866000	5.424761000	10.775444000
H	11.414039000	4.663080000	12.294060000
H	12.042333000	4.045075000	10.751284000
C	9.371745000	11.529037000	11.032463000
H	8.990202000	11.952242000	10.100280000
H	9.759860000	12.346980000	11.643304000
H	8.547855000	11.051411000	11.553567000
C	9.484669000	7.674114000	12.762912000
H	9.138187000	7.125744000	11.892991000

H	8.581765000	7.816985000	13.649750000
H	10.104487000	7.113018000	13.053250000
C	11.360957000	10.970744000	9.802834000
H	12.129049000	10.209411000	9.735019000
H	11.816603000	11.897822000	10.165598000
H	10.974687000	11.148591000	8.793292000
C	10.194182000	9.861293000	13.498101000
H	10.998440000	9.314726000	14.009804000
H	9.412027000	10.075281000	14.229659000
H	10.600448000	10.800701000	13.136457000
C	14.155336000	5.502652000	12.309055000
H	14.935581000	6.194134000	12.005166000
H	14.481797000	4.486613000	12.053610000
H	14.038636000	5.558055000	13.394989000

H	8.679647000	7.724746000	13.495812000
H	10.329954000	7.138462000	13.204374000
C	11.374709000	10.989349000	9.718141000
H	12.208850000	10.296552000	9.682280000
H	11.746872000	11.974518000	10.003728000
H	10.934397000	11.059269000	8.720297000
C	9.988108000	9.971880000	13.495765000
H	10.453477000	9.456207000	14.338374000
H	9.018848000	10.352936000	13.822247000
H	10.623041000	10.805856000	13.213087000
C	13.851941000	5.541856000	12.449476000
H	14.812588000	5.984826000	12.205594000
H	13.973085000	4.457942000	12.476555000
H	13.538296000	5.879147000	13.439462000

(L2b+Ha)$^+$
E [Eh]: −971.344479624014

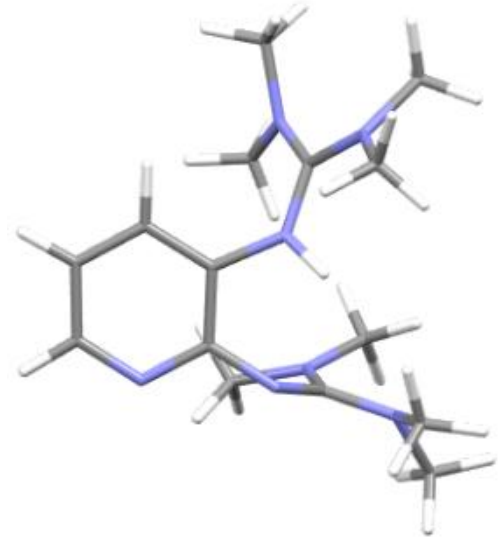

N	11.744001000	6.928663000	7.521919000
N	12.105455000	6.337051000	9.767995000
N	13.959542000	7.724351000	10.278352000
N	13.351932000	5.938627000	11.659189000
N	9.124907000	9.081769000	12.394825000
N	10.295191000	8.186903000	10.618745000
N	10.299796000	10.494561000	10.952483000
C	9.842504000	8.812221000	8.282519000
H	9.078530000	9.517444000	8.585595000
C	13.109258000	6.666627000	10.516105000
C	10.117810000	8.594961000	6.940670000
H	9.589564000	9.133144000	6.166723000
C	11.512037000	7.140178000	8.819428000
C	11.074247000	7.638710000	6.617645000
H	11.310788000	7.429776000	5.580333000
C	10.552695000	8.091483000	9.228018000
C	9.905916000	9.262797000	11.316313000
C	14.626028000	8.451736000	11.344813000
H	14.520327000	9.526288000	11.168973000
H	15.697544000	8.234004000	11.393535000
H	14.176721000	8.207102000	12.303561000
C	14.351438000	8.098517000	8.926082000
H	14.039193000	7.337803000	8.218105000
H	15.439566000	8.193200000	8.884047000
H	13.915907000	9.055957000	8.623185000
C	12.323813000	5.011720000	12.106197000
H	11.350187000	5.498488000	12.120426000
H	12.568143000	4.690105000	13.118464000
H	12.247562000	4.133817000	11.457892000
C	9.426635000	11.659699000	11.069524000
H	9.366059000	12.148152000	10.095024000
H	9.813485000	12.379588000	11.792659000
H	8.427404000	11.353940000	11.364896000
C	8.297478000	7.886341000	12.551141000
H	8.067961000	7.457080000	11.579488000
H	7.364441000	8.171222000	13.036958000
H	8.792067000	7.135818000	13.173794000
C	11.569835000	10.720637000	10.268401000
H	12.220454000	9.861604000	10.400626000
H	12.042844000	11.598954000	10.709033000
H	11.423124000	10.893556000	9.200766000
C	9.195812000	9.954522000	13.565378000
H	9.333885000	9.336322000	14.453654000
H	8.278616000	10.533077000	13.685899000
H	10.040831000	10.630725000	13.479826000
C	14.710109000	5.475933000	11.948900000
H	15.437283000	6.031387000	11.366947000

(L2b+Hb)$^+$
E [Eh]: −971.357149037511

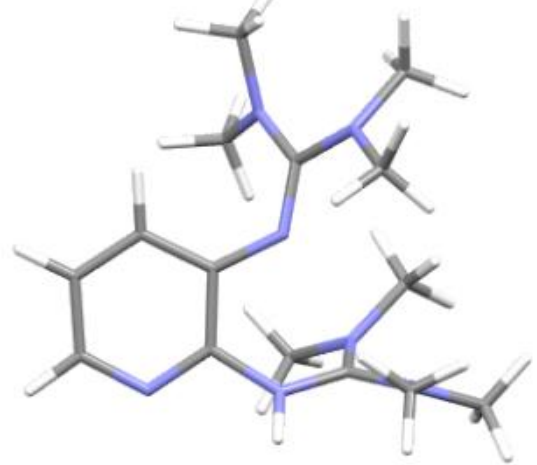

N	11.672471000	6.868779000	7.211609000
N	12.367570000	6.248215000	9.325766000
N	13.754260000	7.691166000	10.506954000
N	12.985708000	5.718364000	11.488424000
N	9.810030000	9.016305000	12.395512000
N	10.766125000	8.198521000	10.476550000
N	10.364966000	10.540541000	10.735579000
C	9.972428000	8.817022000	8.202317000
H	9.264219000	9.539174000	8.583860000
C	13.022089000	6.570157000	10.454204000
C	10.028894000	8.549226000	6.844950000
H	9.391512000	9.081967000	6.152072000
C	11.614906000	7.133881000	8.502744000
C	10.898180000	7.568010000	6.383282000
H	10.966755000	7.322365000	5.331117000
C	10.793342000	8.115779000	9.101027000
C	10.325940000	9.229947000	11.148406000
C	13.856768000	8.480016000	11.729100000
H	13.614264000	9.517366000	11.497138000
H	14.863508000	8.446757000	12.149319000
H	13.139369000	8.119950000	12.459942000
C	14.311905000	8.290642000	9.297843000
H	14.397454000	7.542634000	8.515338000
H	15.303369000	8.677964000	9.532690000
H	13.692186000	9.109715000	8.929257000
C	11.853511000	4.815330000	11.679643000
H	10.975068000	5.225222000	11.189627000
H	11.654012000	4.731203000	12.747972000
H	12.065784000	3.815417000	11.292321000
C	9.296326000	11.485879000	11.030218000
H	8.904547000	11.892593000	10.093243000
H	9.650584000	12.322570000	11.638354000
H	8.486067000	10.986013000	11.552258000
C	9.387177000	7.682119000	12.779799000
H	9.016189000	7.146818000	11.911273000
H	8.591317000	7.763888000	13.521295000
H	10.209728000	7.106801000	13.219995000
C	11.384189000	11.032501000	9.832669000
H	12.205565000	10.326040000	9.782173000
H	11.765119000	11.986311000	10.206078000
H	11.004738000	11.188787000	8.818415000
C	10.048170000	9.929835000	13.505155000
H	10.607311000	9.413338000	14.291213000
H	9.111405000	10.288543000	13.937292000
H	10.632727000	10.782597000	13.174774000
C	14.105077000	5.550441000	12.410048000
H	14.992315000	6.033699000	12.012179000

H	14.808374000	4.418057000	11.686683000	H	14.310353000	4.484688000	12.518170000
H	14.939453000	5.593843000	13.009164000	H	13.878871000	5.960756000	13.396126000
H	10.423890000	7.314240000	11.115700000	H	12.293980000	5.272168000	9.079234000

$(L2b+Hc)^+$
E [Eh]: −971.360732384253

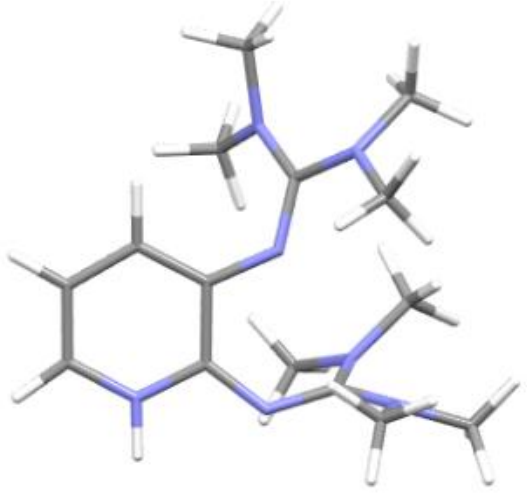

N	11.629276000	6.867350000	7.228200000
N	12.478409000	6.308409000	9.270177000
N	13.799493000	7.697312000	10.629813000
N	12.855987000	5.744544000	11.472440000
N	9.948255000	9.066524000	12.403720000
N	10.840256000	8.259521000	10.455428000
N	10.340991000	10.581908000	10.685072000
C	9.922073000	8.717335000	8.209083000
H	9.198387000	9.423097000	8.593030000
C	12.998222000	6.613257000	10.457302000
C	9.907053000	8.405849000	6.837439000
H	9.210261000	8.887729000	6.168352000
C	11.695702000	7.106970000	8.565979000
C	10.774080000	7.472882000	6.359368000
H	10.835254000	7.161131000	5.328714000
C	10.805406000	8.119113000	9.095680000
C	10.383261000	9.283324000	11.128919000
C	13.812765000	8.452504000	11.875542000
H	13.535162000	9.488262000	11.666552000
H	14.798974000	8.448575000	12.344409000
H	13.081719000	8.043437000	12.565315000
C	14.379211000	8.368695000	9.478158000
H	14.577734000	7.646702000	8.690705000
H	15.321429000	8.826324000	9.779962000
H	13.729186000	9.153517000	9.079528000
C	11.782873000	4.759615000	11.426982000
H	10.935406000	5.164974000	10.884276000
H	11.478071000	4.529542000	12.448085000
H	12.111081000	3.837717000	10.940225000
C	9.265425000	11.498148000	11.040716000
H	8.803543000	11.884361000	10.127505000
H	9.631208000	12.350378000	11.619601000
H	8.505389000	10.978534000	11.616300000
C	9.645786000	7.710659000	12.830748000
H	9.229074000	7.144682000	12.003689000
H	8.919455000	7.752436000	13.642926000
H	10.541279000	7.190873000	13.188559000
C	11.283894000	11.085906000	9.708350000
H	12.144150000	10.426830000	9.648716000
H	11.624396000	12.076300000	10.018479000
H	10.845399000	11.171866000	8.709006000
C	10.208360000	10.008828000	13.485201000
H	10.836448000	9.529855000	14.241869000
H	9.283146000	10.331385000	13.967299000
H	10.733998000	10.881100000	13.109925000
C	13.882606000	5.516583000	12.482376000
H	14.798422000	6.032846000	12.211261000
H	14.092712000	4.446715000	12.531831000
H	13.560900000	5.848389000	13.472046000
H	12.247404000	6.139737000	6.894373000

L3
E [Eh]: −1428.117136737907

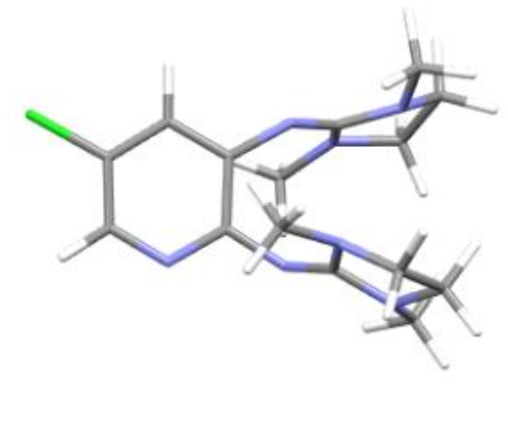

N	7.144024000	6.601219000	6.344902000
N	4.128541000	9.789212000	4.804331000
N	3.847634000	9.236580000	2.663642000
N	5.061728000	6.577340000	5.303254000
N	5.904766000	8.672662000	3.588364000
N	5.175677000	5.558620000	3.107817000
N	3.249531000	5.587070000	4.235541000
C	8.351643000	7.134509000	6.539378000
C	4.736854000	9.178937000	3.729176000
C	8.803583000	8.207888000	5.793190000
C	4.579925000	5.973035000	4.284808000
C	6.339734000	7.101860000	5.412000000
C	7.985150000	8.759741000	4.815929000
C	6.717202000	8.227527000	4.617725000
C	2.749186000	10.119569000	4.487788000
H	2.461297000	11.067451000	4.944348000
H	2.060128000	9.342145000	4.842367000
C	2.783607000	10.177573000	2.962866000
H	1.842673000	9.874082000	2.502718000
H	3.033574000	11.189203000	2.610280000
C	4.537866000	9.682054000	6.189032000
H	4.239472000	8.729087000	6.634371000
H	4.078265000	10.501282000	6.743604000
H	5.616258000	9.779550000	6.272303000
C	6.601783000	5.324941000	2.981380000
H	7.158958000	6.248678000	3.104840000
H	6.793275000	4.947416000	1.977401000
H	6.965791000	4.590744000	3.708298000
C	4.260333000	4.696184000	2.371703000
H	4.433373000	3.637652000	2.610658000
H	4.372928000	4.832914000	1.295790000
C	4.324179000	9.090629000	1.309562000
H	4.752681000	10.023739000	0.919245000
H	3.497752000	8.787973000	0.664463000
H	5.095066000	8.324423000	1.297855000
C	2.258692000	6.200759000	5.079796000
C	2.904863000	5.156736000	2.895321000
H	2.511394000	5.992521000	2.297485000
H	2.160382000	4.359616000	2.910632000
H	1.442030000	5.499906000	5.261633000
H	1.840980000	7.110628000	4.626765000
H	2.725735000	6.462856000	6.026056000
H	8.975369000	6.689843000	7.305667000
Cl	10.398501000	8.867798000	6.078394000
H	8.316397000	9.594606000	4.214093000

$(L3)^{\cdot+}$
E [Eh]: −1427.907296499318

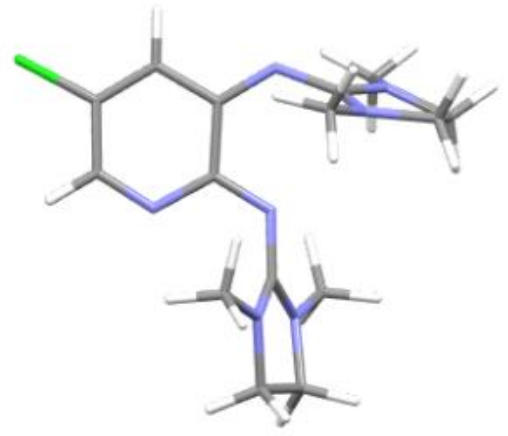

N	7.065428000	6.310898000	4.793744000
N	3.592440000	10.216215000	4.853889000
N	4.512595000	9.890622000	2.873189000
N	4.940729000	7.173291000	4.361745000
N	5.903148000	9.798582000	4.797863000
N	4.839294000	5.314489000	2.854883000
N	3.504198000	5.330382000	4.608683000
C	8.294781000	6.508029000	5.209491000
C	4.722854000	9.896684000	4.201238000
C	8.797429000	7.791325000	5.546320000
C	4.490228000	5.977205000	3.970200000
C	6.220750000	7.363789000	4.666805000
C	7.997980000	8.890541000	5.411929000
C	6.671087000	8.732576000	4.934302000
C	2.460723000	10.244400000	3.931464000
H	1.811065000	11.094710000	4.134074000
H	1.875303000	9.324750000	4.033698000
C	3.153514000	10.332730000	2.563843000
H	2.693806000	9.688328000	1.815063000
H	3.172204000	11.356327000	2.176972000
C	3.414773000	10.080809000	6.282055000
H	2.952096000	9.121700000	6.534198000
H	2.778718000	10.886183000	6.648557000
H	4.386302000	10.147364000	6.765479000
C	5.964687000	5.619857000	2.000344000
H	5.718290000	5.333699000	0.977995000
H	6.863385000	5.090428000	2.323141000
H	6.161448000	6.688340000	2.026969000
C	4.181805000	4.006223000	2.811354000
H	4.890341000	3.224843000	3.101481000
H	3.814686000	3.790367000	1.809224000
C	5.541006000	9.836074000	1.860969000
H	5.609629000	10.789334000	1.329685000
H	5.317563000	9.049619000	1.137652000
H	6.499906000	9.629710000	2.327909000
C	2.786919000	5.824356000	5.761552000
C	3.053198000	4.171050000	3.840027000
H	2.089066000	4.383204000	3.367188000
H	2.938615000	3.300467000	4.483978000
H	2.704961000	5.037866000	6.512601000
H	1.779974000	6.151894000	5.485388000
H	3.335935000	6.662200000	6.181978000
H	8.941514000	5.642752000	5.300008000
Cl	10.424338000	7.920018000	6.109740000
H	8.350072000	9.884530000	5.647905000

L4
E [Eh]: −1428.120033990617

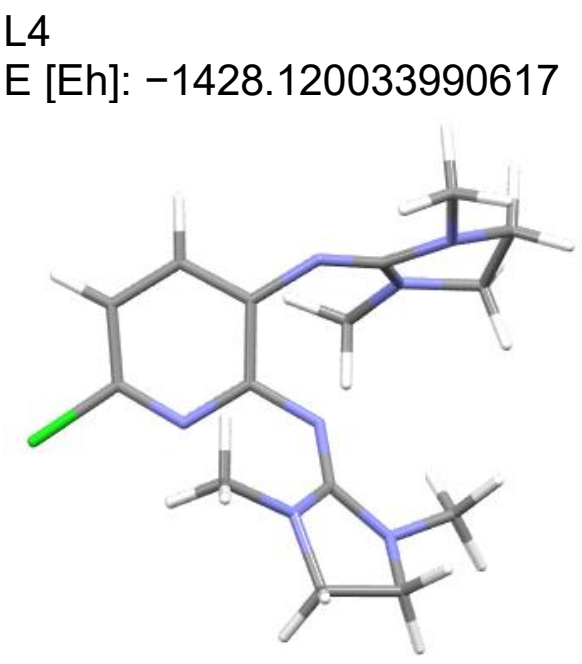

N	6.975322000	6.509717000	5.535286000
N	4.013915000	10.013136000	4.860639000
N	3.913850000	10.204372000	2.647299000
N	4.924520000	6.922720000	4.473922000

$(L4)^{\cdot+}$
E [Eh]: −1427.913795742156

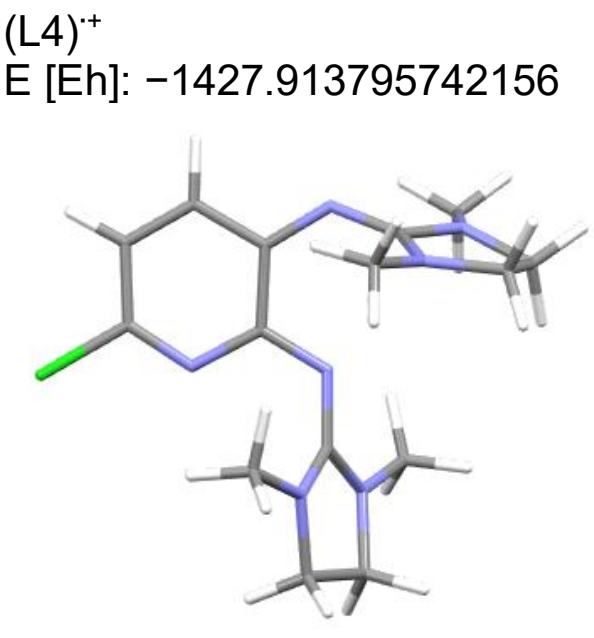

N	7.053487000	6.329434000	4.820989000
N	3.609165000	10.256438000	4.844610000
N	4.522321000	9.899962000	2.866713000
N	4.936125000	7.192052000	4.360655000

N	5.915848000	9.377435000	3.495707000	N	5.916933000	9.821147000	4.789022000
N	5.295459000	4.775099000	3.422004000	N	4.872396000	5.300266000	2.889288000
N	3.291844000	5.280233000	4.236580000	N	3.475916000	5.366934000	4.592774000
C	8.177900000	6.912272000	5.883020000	C	8.274558000	6.535782000	5.231453000
C	4.721581000	9.794944000	3.698274000	C	4.734312000	9.919878000	4.192606000
C	8.731510000	8.128552000	5.526941000	C	8.811620000	7.801909000	5.565828000
C	4.587444000	5.743744000	4.084800000	C	4.489410000	5.992808000	3.975224000
C	6.208726000	7.310697000	4.781645000	C	6.213555000	7.381485000	4.677598000
C	7.943038000	8.969850000	4.747339000	C	8.003293000	8.891365000	5.417000000
C	6.657366000	8.598362000	4.367626000	C	6.672725000	8.746527000	4.936104000
C	2.638584000	10.365681000	4.557407000	C	2.474352000	10.280633000	3.925430000
H	2.251665000	11.086081000	5.279413000	H	1.835598000	11.142147000	4.114921000
H	1.985711000	9.482107000	4.562897000	H	1.878748000	9.370110000	4.046831000
C	2.773528000	10.944136000	3.150748000	C	3.162393000	10.336263000	2.553038000
H	1.888848000	10.779882000	2.534276000	H	2.699265000	9.674302000	1.821785000
H	2.977608000	12.025115000	3.185851000	H	3.180406000	11.349931000	2.141322000
C	4.376467000	9.559399000	6.182644000	C	3.435934000	10.146513000	6.275626000
H	4.082733000	8.521671000	6.362214000	H	2.969953000	9.194099000	6.546184000
H	3.887708000	10.205256000	6.914366000	H	2.804690000	10.961012000	6.630044000
H	5.450498000	9.639451000	6.322514000	H	4.409239000	10.216963000	6.754880000
C	6.709838000	4.782115000	3.130371000	C	6.022420000	5.579836000	2.058779000
H	7.032744000	5.789543000	2.880215000	H	5.801036000	5.271103000	1.037174000
H	6.881035000	4.142762000	2.262484000	H	6.908819000	5.053113000	2.417906000
H	7.311599000	4.427224000	3.970821000	H	6.224612000	6.647543000	2.065643000
C	4.509704000	3.558318000	3.303011000	C	4.207136000	3.996024000	2.852477000
H	4.740554000	2.853049000	4.111802000	H	4.900140000	3.216468000	3.181995000
H	4.697849000	3.065035000	2.348691000	H	3.871825000	3.761107000	1.843433000
C	4.476923000	10.521234000	1.359146000	C	5.541881000	9.790996000	1.850458000
H	4.858439000	11.551073000	1.317899000	H	5.606566000	10.715660000	1.270925000
H	3.715662000	10.399943000	0.586334000	H	5.311706000	8.968443000	1.170483000
H	5.302585000	9.840663000	1.167416000	H	6.504339000	9.607594000	2.319715000
C	2.203283000	6.185885000	4.501388000	C	2.727330000	5.887202000	5.713091000
C	3.084387000	4.096685000	3.422740000	C	3.045784000	4.190677000	3.838596000
H	2.672986000	4.356278000	2.435946000	H	2.098579000	4.393602000	3.328872000
H	2.404307000	3.396016000	3.907968000	H	2.906654000	3.336765000	4.499883000
H	1.378558000	5.640570000	4.962759000	H	2.624044000	5.118968000	6.480409000
H	1.837795000	6.671021000	3.585423000	H	1.728380000	6.207761000	5.401745000
H	2.551041000	6.957027000	5.183195000	H	3.265003000	6.735049000	6.128150000
Cl	9.128740000	5.790629000	6.858180000	Cl	9.305056000	5.149416000	5.373819000
H	9.727724000	8.404223000	5.838676000	H	9.828864000	7.884276000	5.917529000
H	8.321375000	9.929536000	4.418885000	H	8.352231000	9.889177000	5.645754000

L4b
E [Eh]: −2035.159830293670

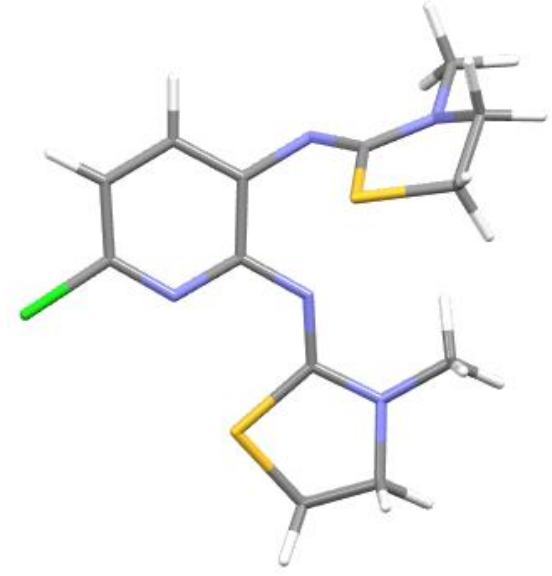

$(L4b)^{\cdot+}$
E [Eh]: −2034.930363155326

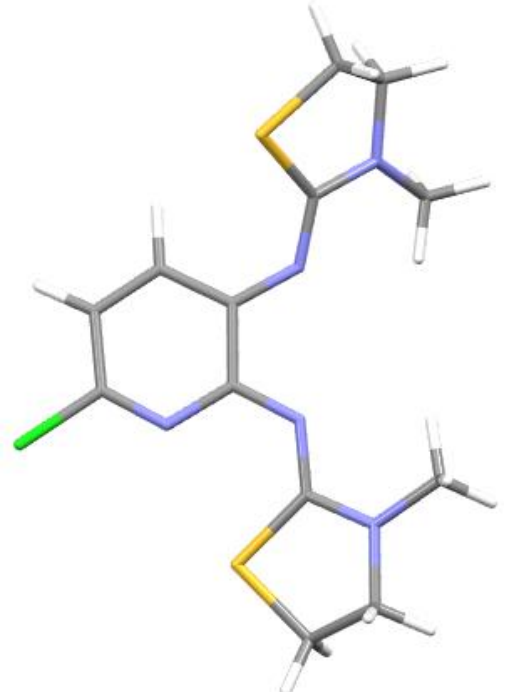

H	8.380425000	1.174661000	13.870756000	H	7.574058000	0.907223000	13.427211000
S	4.849140000	5.970712000	13.050633000	S	5.323343000	6.469329000	13.244328000
N	5.747484000	4.516871000	10.871978000	N	5.334283000	4.880515000	10.968224000
C	6.439393000	3.629614000	11.657268000	C	5.917588000	3.863348000	11.596276000
Cl	6.800691000	2.956041000	15.470153000	Cl	6.711477000	2.890961000	15.260830000
S	4.897748000	1.624484000	9.332815000	S	5.666599000	0.214581000	8.924985000
N	4.421110000	6.364154000	10.508746000	N	4.270823000	6.914310000	10.923341000
C	7.261659000	2.662215000	11.019136000	C	6.407068000	2.744681000	10.798033000
N	7.423920000	2.643513000	9.636567000	N	6.319214000	2.824103000	9.470643000
C	7.972547000	1.789477000	11.835551000	C	7.017972000	1.674338000	11.503770000
H	8.620744000	1.056967000	11.372446000	H	7.433134000	0.843496000	10.950545000
N	6.508991000	2.542712000	7.502949000	N	6.554517000	2.036421000	7.314064000
C	7.849626000	1.851536000	13.218469000	C	7.109858000	1.704246000	12.865855000
N	6.334868000	3.676246000	12.990286000	N	6.029312000	3.848186000	12.941311000
C	7.005664000	2.825290000	13.729718000	C	6.587953000	2.838157000	13.539949000
C	5.079883000	5.503724000	11.348265000	C	4.971871000	5.990043000	11.589822000
C	3.987693000	7.507192000	12.537939000	C	4.754849000	8.177654000	12.887538000

H	3.204927000	7.739903000	13.256521000
H	4.706191000	8.326602000	12.510016000
C	3.436386000	7.212451000	11.153697000
H	2.466600000	6.697796000	11.220802000
H	3.297173000	8.124226000	10.569657000
C	4.241975000	6.015656000	9.114757000
H	5.131442000	5.500117000	8.762895000
H	4.084803000	6.925775000	8.534904000
H	3.379152000	5.351523000	8.974089000
C	6.459055000	2.360253000	8.859628000
C	4.275639000	1.746763000	7.622673000
H	3.646880000	0.887252000	7.402178000
H	3.690719000	2.660278000	7.515810000
C	5.525568000	1.787675000	6.751765000
H	5.881036000	0.769866000	6.536741000
H	5.328521000	2.286994000	5.801122000
C	7.762011000	2.916009000	6.880724000
H	8.235725000	3.687669000	7.482737000
H	7.561701000	3.300615000	5.880608000
H	8.452341000	2.067117000	6.806339000

H	4.274727000	8.584315000	13.773709000
H	5.620796000	8.787975000	12.634203000
C	3.793557000	8.042643000	11.718214000
H	2.772067000	7.836143000	12.056565000
H	3.774379000	8.939114000	11.098573000
C	3.751621000	6.714106000	9.580743000
H	4.277839000	5.883828000	9.121681000
H	3.898650000	7.624311000	8.999549000
H	2.682422000	6.488240000	9.619592000
C	6.253879000	1.853001000	8.607073000
C	5.545641000	-0.123473000	7.131142000
H	5.795279000	-1.164737000	6.946255000
H	4.522653000	0.068658000	6.812257000
C	6.536038000	0.836157000	6.484302000
H	7.541320000	0.403520000	6.441440000
H	6.232467000	1.099262000	5.470995000
C	7.160312000	3.260503000	6.815497000
H	6.943114000	4.065149000	7.512549000
H	6.744758000	3.491971000	5.835498000
H	8.243993000	3.147671000	6.724780000

L5a
E [Eh]: −1887.685128548246

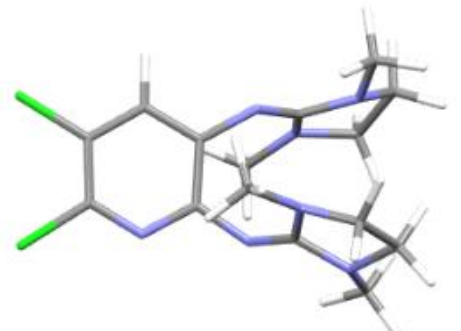

N	7.018236000	6.916266000	6.426703000
N	3.985075000	9.970105000	4.684129000
N	3.761354000	9.326571000	2.564465000
N	4.967135000	6.767438000	5.345014000
N	5.807834000	8.839447000	3.552747000
N	5.128863000	5.840397000	3.124562000
N	3.171051000	5.830300000	4.189630000
C	8.197319000	7.465346000	6.623498000
C	4.628122000	9.330459000	3.648851000
C	8.679276000	8.506625000	5.838174000
C	4.500701000	6.208340000	4.289063000
C	6.218339000	7.342793000	5.450195000
C	7.857628000	8.984807000	4.823281000
C	6.598847000	8.436195000	4.616116000
C	2.610869000	10.277659000	4.323887000
H	2.312499000	11.247528000	4.724087000
H	1.917218000	9.518734000	4.706993000
C	2.672525000	10.257868000	2.797788000
H	1.747040000	9.910207000	2.337459000
H	2.906588000	11.255160000	2.397577000
C	4.376946000	9.949914000	6.077179000
H	4.074429000	9.026274000	6.578199000
H	3.909226000	10.799957000	6.575771000
H	5.453904000	10.055334000	6.167020000
C	6.561394000	5.645301000	3.005692000
H	7.061891000	6.554735000	2.670692000
H	6.743973000	4.854710000	2.277296000
H	6.994231000	5.337417000	3.957517000
C	4.215033000	5.116601000	2.253592000
H	4.369214000	4.032866000	2.338508000
H	4.348645000	5.403271000	1.209435000
C	4.257711000	9.110106000	1.227805000
H	4.673231000	10.025863000	0.786249000
H	3.445672000	8.753354000	0.591840000
H	5.043948000	8.360729000	1.270993000
C	2.164912000	6.404610000	5.045319000
C	2.852707000	5.528738000	2.806311000
H	2.470730000	6.421910000	2.290773000
H	2.106060000	4.737409000	2.729813000
H	1.355252000	5.688697000	5.197870000
H	1.738271000	7.321748000	4.616930000
H	2.620403000	6.641611000	6.003519000
Cl	9.158950000	6.812958000	7.930610000
Cl	10.246735000	9.219534000	6.079123000

$(L5a)^{\cdot+}$
E [Eh]: −1887.465961466626

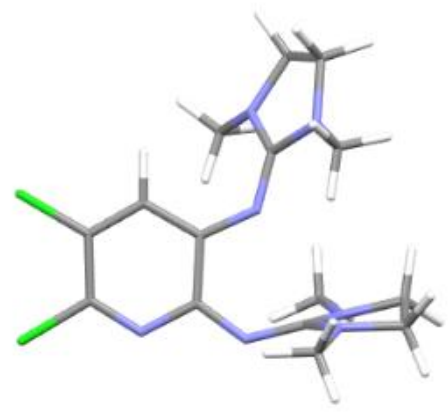

N	7.527907000	6.651343000	6.208290000
N	3.914983000	10.316331000	4.664481000
N	3.925068000	9.622622000	2.554950000
N	6.004394000	5.751431000	4.781364000
N	5.096325000	8.300227000	4.065687000
N	5.122796000	5.321970000	2.641555000
N	3.691890000	5.544101000	4.313035000
C	8.145580000	7.659711000	6.729672000
C	4.378896000	9.371610000	3.804562000
C	7.840519000	9.021285000	6.396185000
C	4.985360000	5.618285000	3.946617000
C	6.525018000	6.832615000	5.310607000
C	6.817429000	9.256485000	5.513290000
C	6.090447000	8.186398000	4.953894000
C	2.969749000	11.200816000	3.972815000
H	3.126861000	12.240022000	4.257079000
H	1.944873000	10.919345000	4.236435000
C	3.272671000	10.928507000	2.497453000
H	2.377059000	10.896081000	1.878507000
H	3.958397000	11.671254000	2.076835000
C	3.771606000	10.137712000	6.098498000
H	2.720027000	9.975427000	6.352636000
H	4.123840000	11.025544000	6.624516000
H	4.349541000	9.284365000	6.435172000
C	6.381672000	4.986813000	2.006612000
H	6.450309000	5.477098000	1.035122000
H	6.465919000	3.905861000	1.863867000
H	7.200316000	5.322058000	2.637099000
C	3.843161000	4.877683000	2.087362000
H	3.827736000	3.785818000	2.010127000
H	3.676825000	5.298355000	1.096756000
C	4.480216000	9.008239000	1.366486000
H	5.257014000	9.636826000	0.920202000
H	3.688251000	8.857926000	0.632637000
H	4.911818000	8.048820000	1.640278000
C	3.170026000	6.034227000	5.566565000
C	2.846019000	5.393746000	3.131396000
H	2.426924000	6.365553000	2.846933000
H	2.027897000	4.700644000	3.320099000
H	2.320565000	5.422724000	5.868891000
H	2.845332000	7.076300000	5.481238000
H	3.940817000	5.959858000	6.330197000
Cl	9.393515000	7.331143000	7.873235000
Cl	8.715783000	10.333538000	7.072743000

H	8.194463000	9.794089000	4.190486000	H	6.573099000	10.273259000	5.240780000

L5b
E [Eh]: −2494.723537806311

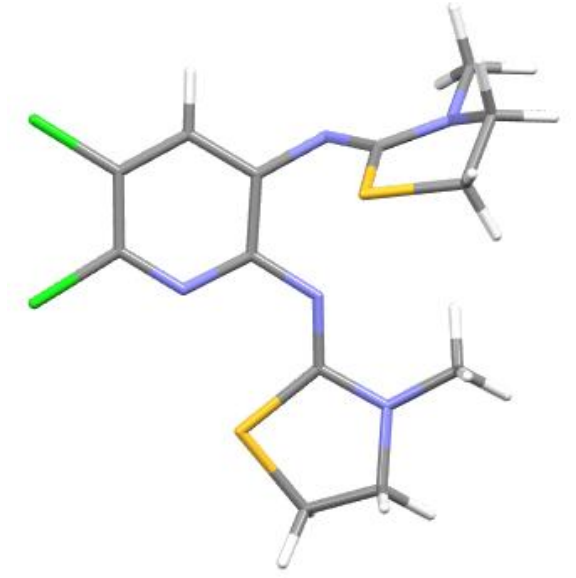

$(L5b)^{\cdot+}$
E [Eh]: −2494.491367845771

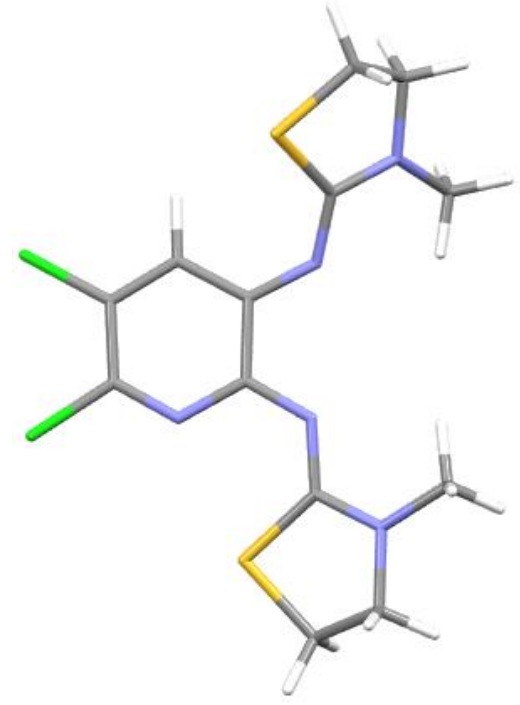

Cl	8.733557000	0.766750000	14.192405000	Cl	7.828921000	0.425810000	13.700274000
S	4.828702000	5.969546000	13.050555000	S	5.308690000	6.479994000	13.238812000
N	5.751494000	4.549675000	10.861457000	N	5.327627000	4.901589000	10.955610000
C	6.441814000	3.658012000	11.641110000	C	5.905180000	3.880162000	11.579830000
Cl	6.791462000	3.012803000	15.456379000	Cl	6.650027000	2.937812000	15.261311000
S	4.863486000	1.707647000	9.311447000	S	5.695088000	0.218337000	8.932268000
N	4.406969000	6.388516000	10.512374000	N	4.265889000	6.936290000	10.915810000
C	7.254702000	2.682015000	11.000679000	C	6.399255000	2.764041000	10.782865000
N	7.417580000	2.647844000	9.621212000	N	6.318990000	2.842169000	9.453827000
C	7.965215000	1.810965000	11.815575000	C	7.004241000	1.698950000	11.487698000
H	8.611193000	1.071309000	11.364219000	H	7.428779000	0.866662000	10.945771000
N	6.513511000	2.552792000	7.480548000	N	6.546334000	2.040464000	7.301926000
C	7.848182000	1.880543000	13.199291000	C	7.089160000	1.720531000	12.855634000
N	6.348955000	3.698099000	12.971785000	N	6.010489000	3.854839000	12.923932000
C	7.009712000	2.856130000	13.732221000	C	6.559799000	2.856863000	13.547349000
C	5.070973000	5.525452000	11.344674000	C	4.963320000	6.009074000	11.580965000
C	3.936232000	7.494133000	12.556460000	C	4.743980000	8.190465000	12.888176000
H	3.141526000	7.693989000	13.271748000	H	4.260268000	8.593247000	13.774090000
H	4.635462000	8.330364000	12.551298000	H	5.612032000	8.800652000	12.642148000
C	3.403621000	7.211275000	11.162373000	C	3.787761000	8.062399000	11.713708000
H	2.441716000	6.680217000	11.211368000	H	2.764463000	7.856060000	12.046300000
H	3.255182000	8.129675000	10.591145000	H	3.773100000	8.961478000	11.097930000
C	4.233015000	6.054391000	9.114010000	C	3.753966000	6.744058000	9.569005000
H	5.119136000	5.533493000	8.763163000	H	4.279393000	5.913688000	9.109385000
H	4.088561000	6.970916000	8.540908000	H	3.908215000	7.656232000	8.992968000
H	3.363945000	5.400716000	8.963496000	H	2.683750000	6.522703000	9.601079000
C	6.448056000	2.389047000	8.839999000	C	6.257231000	1.863349000	8.598482000
C	4.258890000	1.818457000	7.594334000	C	5.564742000	-0.133584000	7.141893000
H	3.602818000	0.977476000	7.382559000	H	5.826570000	-1.172777000	6.962521000
H	3.707757000	2.750322000	7.467664000	H	4.536968000	0.042964000	6.829638000
C	5.518448000	1.801548000	6.738636000	C	6.537377000	0.834129000	6.480892000
H	5.852616000	0.769115000	6.562902000	H	7.547956000	0.414593000	6.433453000
H	5.347635000	2.274015000	5.769477000	H	6.222666000	1.085834000	5.468133000
C	7.788771000	2.841465000	6.856616000	C	7.136782000	3.266579000	6.790326000
H	8.345945000	3.519584000	7.497358000	H	6.919585000	4.073639000	7.484294000
H	7.616091000	3.308768000	5.886707000	H	6.710405000	3.488692000	5.812835000
H	8.386503000	1.932888000	6.713186000	H	8.220430000	3.161965000	6.690202000

L6
E [Eh]: −1887.688095510445

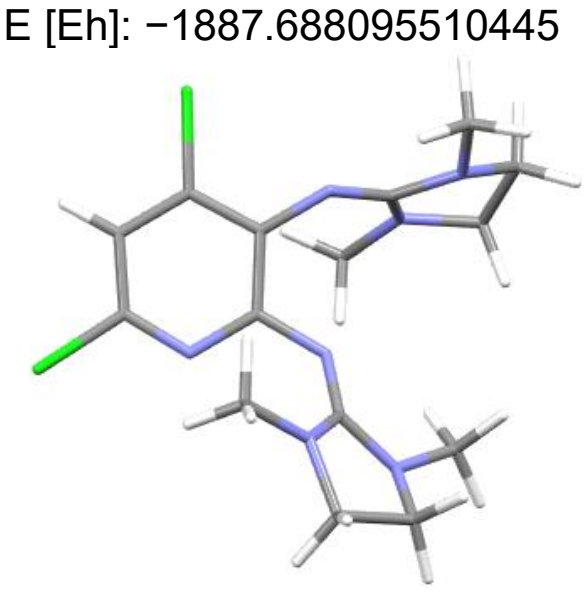

$(L6)^{\cdot+}$
E [Eh]: −1887.476948418927

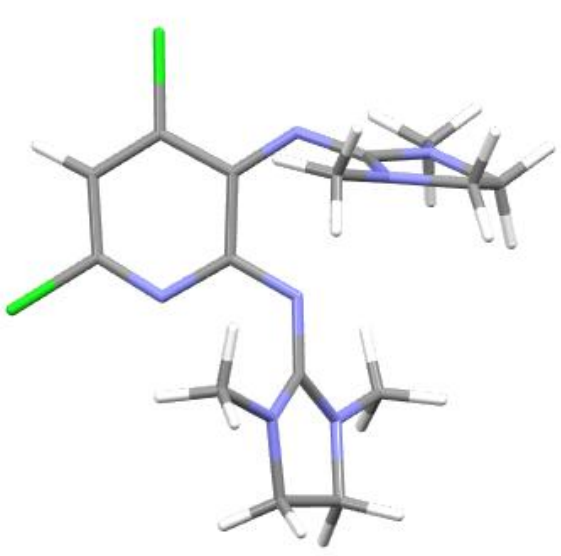

N	6.954356000	6.462382000	5.548320000	N	7.022790000	6.304208000	4.867894000
N	4.023563000	10.033742000	4.842760000	N	3.597921000	10.238991000	4.792623000
N	3.940242000	10.179592000	2.625014000	N	4.542816000	9.888129000	2.828063000
N	4.917566000	6.901876000	4.473858000	N	4.926042000	7.182934000	4.355621000

N	5.919079000	9.335548000	3.507527000
N	5.270873000	4.751096000	3.427202000
N	3.273167000	5.269398000	4.246450000
C	8.150683000	6.865584000	5.918697000
C	4.731593000	9.780853000	3.689746000
C	8.703793000	8.090502000	5.601763000
C	4.570273000	5.722543000	4.089684000
C	6.198312000	7.275314000	4.798211000
C	7.917267000	8.936839000	4.823311000
C	6.641819000	8.571470000	4.395511000
C	2.654813000	10.400119000	4.524493000
H	2.274746000	11.141696000	5.228224000
H	1.989200000	9.526281000	4.545137000
C	2.806663000	10.945884000	3.106276000
H	1.923550000	10.781279000	2.487870000
H	3.027284000	12.023710000	3.119403000
C	4.377973000	9.593669000	6.171589000
H	4.102896000	8.551141000	6.353437000
H	3.866745000	10.233071000	6.892891000
H	5.448288000	9.700287000	6.325433000
C	6.683396000	4.755024000	3.126573000
H	7.006346000	5.761828000	2.873157000
H	6.848188000	4.114477000	2.258600000
H	7.289339000	4.400302000	3.963946000
C	4.476886000	3.539141000	3.308077000
H	4.705402000	2.832227000	4.115851000
H	4.660168000	3.045698000	2.353075000
C	4.520222000	10.470013000	1.336938000
H	4.905594000	11.497227000	1.282833000
H	3.767744000	10.336721000	0.557655000
H	5.345883000	9.783723000	1.167382000
C	2.191259000	6.183697000	4.512177000
C	3.055546000	4.087273000	3.431800000
H	2.644498000	4.351860000	2.446359000
H	2.371158000	3.391379000	3.917396000
H	1.362801000	5.643355000	4.972363000
H	1.830134000	6.672289000	3.596606000
H	2.543991000	6.951255000	5.195524000
Cl	9.094773000	5.735655000	6.884699000
H	9.686002000	8.382490000	5.936982000
Cl	8.544717000	10.499273000	4.386020000

N	5.903420000	9.799999000	4.770845000
N	4.867672000	5.281767000	2.898637000
N	3.462350000	5.360099000	4.594175000
C	8.236284000	6.510066000	5.304694000
C	4.731126000	9.902684000	4.157077000
C	8.777430000	7.768269000	5.640787000
C	4.478381000	5.980293000	3.977408000
C	6.197154000	7.361097000	4.699085000
C	7.979990000	8.866497000	5.463857000
C	6.649931000	8.732157000	4.949798000
C	2.478675000	10.275462000	3.854797000
H	1.843354000	11.140718000	4.038615000
H	1.874340000	9.368912000	3.961029000
C	3.190312000	10.334203000	2.494149000
H	2.735897000	9.678708000	1.751842000
H	3.221491000	11.349889000	2.088416000
C	3.406144000	10.140463000	6.222178000
H	2.936763000	9.190499000	6.494890000
H	2.770514000	10.958030000	6.561211000
H	4.373259000	10.216495000	6.713239000
C	6.025445000	5.555604000	2.076864000
H	6.231471000	6.622655000	2.083719000
H	5.811833000	5.246101000	1.053884000
H	6.906597000	5.025899000	2.444579000
C	4.201801000	3.977426000	2.865111000
H	4.894546000	3.199102000	3.197711000
H	3.867727000	3.739554000	1.856367000
C	5.582544000	9.789724000	1.830540000
H	5.652420000	10.717870000	1.257452000
H	5.370820000	8.969212000	1.142274000
H	6.537342000	9.611086000	2.317252000
C	2.713650000	5.883646000	5.713186000
C	3.038646000	4.175298000	3.849144000
H	2.090835000	4.369548000	3.337551000
H	2.903150000	3.326512000	4.517745000
H	2.623824000	5.123047000	6.489795000
H	1.709537000	6.188302000	5.403231000
H	3.243247000	6.742517000	6.115711000
Cl	9.256134000	5.121581000	5.483749000
H	9.785675000	7.855568000	6.013996000
Cl	8.567487000	10.437960000	5.836029000

L7
E [Eh]: −1428.118567239619

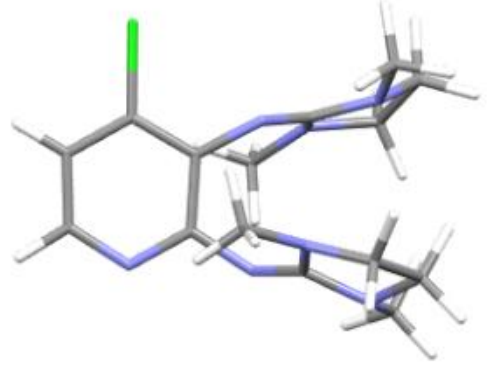

N	7.147644000	6.539863000	6.456654000
N	4.143899000	9.756599000	4.815197000
N	3.820342000	9.118759000	2.703164000
N	5.080097000	6.434912000	5.389933000
N	5.857418000	8.510688000	3.640564000
N	5.233736000	5.528458000	3.164216000
N	3.259737000	5.585899000	4.199018000
C	8.334130000	7.125818000	6.634715000
C	4.718097000	9.076747000	3.763821000
C	8.781288000	8.196902000	5.883209000
C	4.606082000	5.907179000	4.322732000
C	6.339493000	6.996680000	5.502624000
C	7.930290000	8.686386000	4.897273000
C	6.680861000	8.117213000	4.678833000
C	2.778957000	10.138154000	4.493285000
H	2.544796000	11.123406000	4.898840000
H	2.055360000	9.418491000	4.897299000
C	2.796038000	10.113377000	2.966378000
H	1.838284000	9.821438000	2.533829000
H	3.077670000	11.094764000	2.557534000

$(L7)^{\cdot+}$
E [Eh]: −1427.907847501993

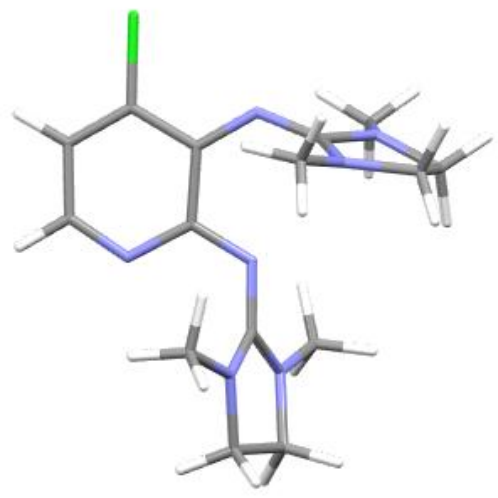

N	7.020936000	6.270934000	4.885796000
N	3.632617000	10.213380000	4.763234000
N	4.573022000	9.866020000	2.794792000
N	4.933053000	7.176860000	4.374476000
N	5.941283000	9.781772000	4.730664000
N	4.818806000	5.289364000	2.907164000
N	3.464107000	5.360918000	4.643642000
C	8.247221000	6.470454000	5.327177000
C	4.765725000	9.881096000	4.124458000
C	8.786505000	7.734160000	5.637747000
C	4.466867000	5.979994000	4.004470000
C	6.211243000	7.339046000	4.706933000
C	8.008957000	8.843986000	5.444059000
C	6.678704000	8.711696000	4.933745000
C	2.511566000	10.238537000	3.827205000
H	1.860215000	11.089786000	4.019486000
H	1.925551000	9.318801000	3.924739000
C	3.221969000	10.322476000	2.468383000
H	2.766261000	9.683721000	1.712634000
H	3.255928000	11.346844000	2.084491000

C	4.557401000	9.676494000	6.200341000
H	4.235581000	8.745216000	6.675295000
H	4.121522000	10.521685000	6.734407000
H	5.638224000	9.749260000	6.277134000
C	6.661624000	5.326257000	3.033302000
H	7.159440000	6.219156000	2.649660000
H	6.835836000	4.499727000	2.342645000
H	7.103858000	5.065993000	3.993944000
C	4.303929000	4.887196000	2.250304000
H	4.407804000	3.794629000	2.287771000
H	4.466219000	5.212257000	1.221098000
C	4.278873000	8.897864000	1.352833000
H	4.710328000	9.805135000	0.909389000
H	3.443252000	8.568484000	0.732583000
H	5.045453000	8.127281000	1.373246000
C	2.277026000	6.250251000	5.018747000
C	2.953044000	5.340600000	2.800124000
H	2.619923000	6.266418000	2.309255000
H	2.173241000	4.586543000	2.686820000
H	1.386941000	5.625029000	5.105781000
H	1.983955000	7.219058000	4.592285000
H	2.700624000	6.412858000	6.006545000
H	8.959244000	6.713395000	7.419690000
H	9.748335000	8.646891000	6.054209000
Cl	8.422892000	10.059372000	3.940297000

C	3.442210000	10.096649000	6.191549000
H	2.980565000	9.139805000	6.452757000
H	2.800645000	10.905420000	6.540268000
H	4.408898000	10.174356000	6.683239000
C	5.960684000	5.562880000	2.064010000
H	6.171934000	6.628914000	2.074595000
H	5.726243000	5.260928000	1.043354000
H	6.846698000	5.027200000	2.411078000
C	4.146156000	3.988790000	2.883393000
H	4.843316000	3.204654000	3.193019000
H	3.783406000	3.758186000	1.882907000
C	5.618307000	9.812583000	1.798703000
H	5.723159000	10.778002000	1.295936000
H	5.385240000	9.052566000	1.051004000
H	6.562519000	9.566272000	2.276176000
C	2.758913000	5.874549000	5.794863000
C	3.011343000	4.186221000	3.900161000
H	2.051711000	4.391819000	3.415698000
H	2.887299000	3.332370000	4.564586000
H	2.679164000	5.099699000	6.558357000
H	1.751526000	6.203890000	5.522992000
H	3.316839000	6.714957000	6.198023000
H	8.862979000	5.587152000	5.457928000
H	9.796101000	7.822879000	6.011069000
Cl	8.612401000	10.418193000	5.785862000

L8
E [Eh]: −1887.682604582468

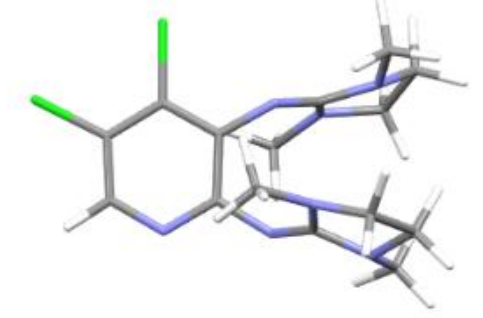

N	7.102320000	6.524251000	6.470596000
N	4.158211000	9.761775000	4.798786000
N	3.836956000	9.107487000	2.692818000
N	5.042447000	6.437930000	5.389141000
N	5.868412000	8.498110000	3.642366000
N	5.196940000	5.526695000	3.164731000
N	3.219572000	5.607647000	4.190810000
C	8.293657000	7.085939000	6.663949000
C	4.729849000	9.070611000	3.755534000
C	8.758048000	8.148658000	5.908203000
C	4.567622000	5.913911000	4.319317000
C	6.306984000	6.983835000	5.507839000
C	7.931048000	8.660970000	4.904680000
C	6.675330000	8.095012000	4.686630000
C	2.796102000	10.149979000	4.471537000
H	2.570035000	11.142079000	4.864520000
H	2.067455000	9.441389000	4.885195000
C	2.813423000	10.106332000	2.944754000
H	1.855556000	9.809805000	2.515749000
H	3.096093000	11.081847000	2.523357000
C	4.577249000	9.704266000	6.183306000
H	4.259496000	8.779827000	6.673961000
H	4.141567000	10.556590000	6.705689000
H	5.658221000	9.782031000	6.254839000
C	6.623144000	5.303337000	3.045308000
H	7.138391000	6.191331000	2.673749000
H	6.790765000	4.480940000	2.348470000
H	7.052933000	5.026966000	4.007236000
C	4.264582000	4.888736000	2.250103000
H	4.359058000	3.795736000	2.293590000
H	4.433735000	5.207524000	1.220178000
C	4.293327000	8.866418000	1.345188000
H	4.719959000	9.768165000	0.886455000
H	3.457709000	8.524055000	0.732030000
H	5.062590000	8.098923000	1.376220000
C	2.238627000	6.278855000	5.006938000
C	2.915536000	5.356389000	2.792457000
H	2.591559000	6.281892000	2.295109000
H	2.129980000	4.608146000	2.681239000
H	1.346663000	5.656878000	5.097136000

$(L8)^{\cdot+}$
E [Eh]: −1887.462406304474

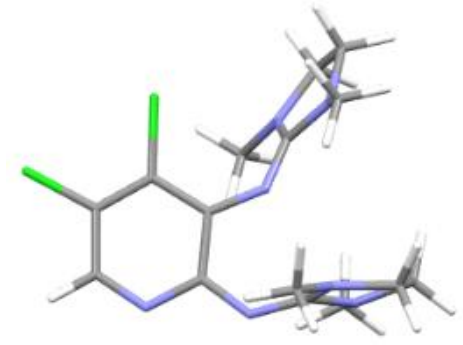

N	7.521119000	6.317952000	6.353955000
N	3.932692000	9.907106000	4.798206000
N	3.997647000	9.376469000	2.649859000
N	5.851828000	5.491581000	5.047255000
N	5.230424000	8.026587000	4.100623000
N	5.185135000	5.118804000	2.812258000
N	3.604694000	5.346756000	4.340898000
C	8.290095000	7.303351000	6.716182000
C	4.470946000	9.079819000	3.883814000
C	8.196850000	8.619116000	6.183684000
C	4.927529000	5.394099000	4.100207000
C	6.545594000	6.536803000	5.431918000
C	7.195716000	8.903887000	5.278427000
C	6.269887000	7.887992000	4.918095000
C	2.990428000	10.837214000	4.175450000
H	3.170369000	11.857439000	4.513019000
H	1.965339000	10.560763000	4.439820000
C	3.275851000	10.648618000	2.678838000
H	2.368503000	10.600525000	2.078298000
H	3.911878000	11.446517000	2.282676000
C	4.027479000	9.780307000	6.235445000
H	3.028292000	9.820341000	6.673831000
H	4.630262000	10.585822000	6.659371000
H	4.482507000	8.829024000	6.496542000
C	6.493096000	4.819488000	2.268778000
H	6.694510000	5.446816000	1.399108000
H	6.549124000	3.770435000	1.967380000
H	7.248371000	5.006197000	3.027091000
C	3.952722000	4.757462000	2.110744000
H	3.909590000	3.673727000	1.965600000
H	3.901857000	5.240119000	1.135564000
C	4.634234000	8.913095000	1.434098000
H	5.383893000	9.627810000	1.079785000
H	3.881924000	8.778511000	0.657091000
H	5.120575000	7.962049000	1.636531000
C	2.990287000	5.860890000	5.543018000
C	2.871491000	5.254038000	3.079845000
H	2.494054000	6.242640000	2.795726000
H	2.029361000	4.569287000	3.165389000
H	2.092107000	5.286322000	5.766174000

H	1.948364000	7.246037000	4.575018000	H	2.718054000	6.915493000	5.428627000
H	2.662678000	6.445540000	5.993835000	H	3.687804000	5.756424000	6.370975000
H	8.914888000	6.676875000	7.452009000	H	9.058999000	7.101141000	7.454021000
Cl	10.331503000	8.811765000	6.232136000	Cl	9.347348000	9.785137000	6.688853000
Cl	8.420198000	10.017290000	3.944077000	Cl	7.088315000	10.464800000	4.543724000

L9
E [Eh]: −1173.066199013515

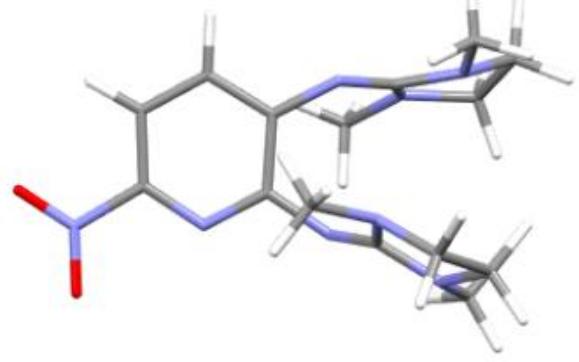

$(L9)^{\cdot+}$
E [Eh]: −1172.830588659624

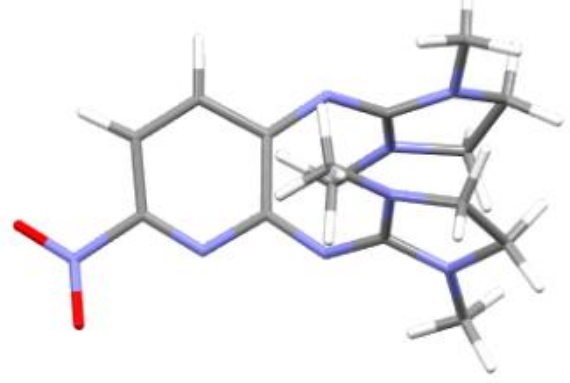

N	7.082315000	7.031016000	6.432355000	N	7.032775000	7.117037000	6.618311000
N	3.986114000	10.045877000	4.713264000	N	3.977134000	9.860387000	4.741002000
N	3.828421000	9.372066000	2.600361000	N	3.919992000	9.217351000	2.621485000
N	5.040720000	6.928860000	5.332810000	N	5.173821000	6.758831000	5.349528000
N	5.892401000	9.037733000	3.621925000	N	5.986413000	9.085127000	3.712958000
N	5.230204000	5.925566000	3.136248000	N	5.180528000	6.029591000	3.064993000
N	3.284808000	5.877201000	4.232527000	N	3.266404000	6.148119000	4.173411000
C	8.249482000	7.609746000	6.637086000	C	8.206113000	7.639331000	6.761680000
C	4.674195000	9.438040000	3.691762000	C	4.690974000	9.331080000	3.728781000
C	8.744813000	8.679609000	5.914712000	C	8.832709000	8.534604000	5.865336000
C	4.596152000	6.310287000	4.304342000	C	4.593751000	6.372785000	4.238150000
C	6.292833000	7.493662000	5.473237000	C	6.298035000	7.441546000	5.532143000
C	7.930644000	9.179178000	4.907120000	C	8.086533000	8.965664000	4.807019000
C	6.674347000	8.624011000	4.672985000	C	6.740620000	8.532884000	4.655868000
C	2.600067000	10.271676000	4.337001000	C	2.592331000	10.100750000	4.333779000
H	2.245004000	11.230002000	4.718035000	H	2.271403000	11.096869000	4.635884000
H	1.947064000	9.481867000	4.727825000	H	1.927556000	9.370497000	4.801945000
C	2.676885000	10.231572000	2.810665000	C	2.658747000	9.935801000	2.809629000
H	1.781072000	9.814803000	2.349502000	H	1.820797000	9.367891000	2.407564000
H	2.847230000	11.235122000	2.395402000	H	2.696938000	10.902158000	2.296705000
C	4.377976000	10.071572000	6.104989000	C	4.407135000	10.036327000	6.112775000
H	4.113406000	9.147088000	6.625472000	H	4.178609000	9.161045000	6.725323000
H	3.879452000	10.913051000	6.587870000	H	3.897906000	10.904666000	6.528189000
H	5.450755000	10.220595000	6.188335000	H	5.477973000	10.222808000	6.147258000
C	6.662475000	5.694818000	3.065964000	C	6.601935000	5.809467000	2.870223000
H	7.213065000	6.620358000	3.205912000	H	7.061572000	6.626607000	2.311935000
H	6.893668000	5.315303000	2.071356000	H	6.743298000	4.881911000	2.315131000
H	6.998423000	4.963786000	3.808852000	H	7.106914000	5.707353000	3.828303000
C	4.354808000	5.037255000	2.378579000	C	4.204124000	5.392407000	2.175743000
H	4.563494000	3.984518000	2.611923000	H	4.379480000	4.313236000	2.145289000
H	4.478399000	5.187850000	1.305868000	H	4.286826000	5.782570000	1.162210000
C	4.342220000	9.139469000	1.273146000	C	4.462685000	9.017136000	1.292952000
H	4.716192000	10.060242000	0.806506000	H	4.652971000	9.972645000	0.794610000
H	3.550583000	8.726451000	0.645811000	H	3.753593000	8.445809000	0.693863000
H	5.159863000	8.426568000	1.342135000	H	5.396183000	8.467954000	1.367931000
C	2.257141000	6.429691000	5.075130000	C	2.321084000	6.407461000	5.232760000
C	2.975968000	5.444095000	2.884551000	C	2.861964000	5.737234000	2.836225000
H	2.561202000	6.270734000	2.288744000	H	2.350063000	6.558620000	2.325080000
H	2.260501000	4.621033000	2.882734000	H	2.182889000	4.885883000	2.879577000
H	1.492853000	5.675780000	5.271726000	H	1.697568000	5.527017000	5.394992000
H	1.773066000	7.299680000	4.611236000	H	1.668244000	7.249202000	4.985607000
H	2.709168000	6.736394000	6.015084000	H	2.868527000	6.627861000	6.145654000
N	9.077054000	7.034331000	7.727282000	N	8.953239000	7.245644000	8.007293000
H	8.253525000	10.009577000	4.292960000	H	8.468488000	9.690081000	4.100747000
O	10.128889000	7.613479000	7.987110000	O	9.955762000	7.901703000	8.246276000
O	8.678946000	6.035585000	8.294717000	O	8.512451000	6.332608000	8.664205000
H	9.714693000	9.095545000	6.135264000	H	9.837504000	8.879183000	6.052098000

L10
E [Eh]: −1173.066388247484

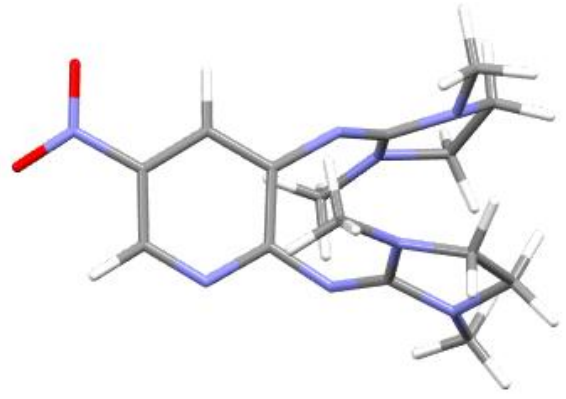

N	7.112599000	6.989444000	6.501766000
N	4.012651000	9.972011000	4.704525000
N	3.770132000	9.296338000	2.594599000
N	5.100471000	6.732969000	5.390598000
N	5.819813000	8.812469000	3.575593000
N	5.210185000	5.856637000	3.153263000
N	3.269926000	5.857518000	4.239977000
C	8.274770000	7.605764000	6.682177000
C	4.645994000	9.313933000	3.673470000
C	8.694572000	8.633797000	5.851344000
C	4.602975000	6.212855000	4.325807000
C	6.310287000	7.364353000	5.497328000
C	7.872867000	9.069881000	4.814475000
C	6.643743000	8.464705000	4.634234000
C	2.637548000	10.281468000	4.347773000
H	2.345882000	11.258086000	4.736081000
H	1.942425000	9.531329000	4.745731000
C	2.689599000	10.239780000	2.822423000
H	1.758456000	9.893996000	2.372382000
H	2.930183000	11.229235000	2.407547000
C	4.402059000	9.952179000	6.098503000
H	4.089353000	9.033879000	6.603777000
H	3.940761000	10.807311000	6.594059000
H	5.479280000	10.049319000	6.191438000
C	6.637330000	5.695061000	2.979315000
H	7.082779000	6.564499000	2.491688000
H	6.821313000	4.809302000	2.368457000
H	7.123874000	5.549060000	3.941724000
C	4.273796000	5.200552000	2.255131000
H	4.424337000	4.113481000	2.265076000
H	4.391194000	5.553799000	1.229345000
C	4.264307000	9.081017000	1.255951000
H	4.671733000	9.999252000	0.812528000
H	3.452902000	8.718048000	0.622791000
H	5.056537000	8.338089000	1.296388000
C	2.284317000	6.409128000	5.133967000
C	2.922125000	5.587753000	2.856237000
H	2.519995000	6.491185000	2.377041000
H	2.179992000	4.792502000	2.777435000
H	1.477948000	5.689382000	5.285489000
H	1.848778000	7.337036000	4.740737000
H	2.761414000	6.618302000	6.088236000
N	9.990176000	9.258940000	6.061315000
H	8.187431000	9.875309000	4.168137000
O	10.342662000	10.126947000	5.269445000
O	10.663205000	8.887132000	7.016312000
H	8.900265000	7.272315000	7.499084000

$(L10)^{\cdot+}$
E [Eh]: −1172.830497614731

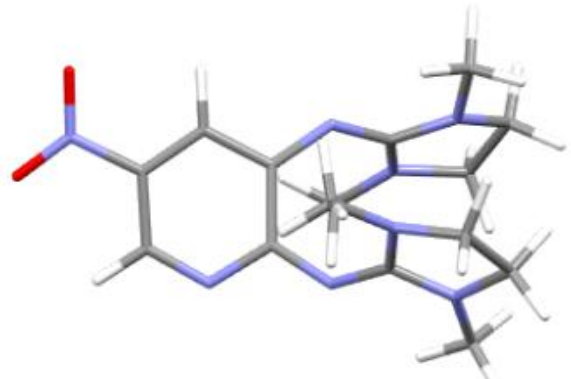

N	6.957249000	7.193540000	6.666586000
N	3.929722000	9.873044000	4.750645000
N	3.847302000	9.228223000	2.632513000
N	5.140579000	6.771739000	5.354972000
N	5.926411000	9.093004000	3.696724000
N	5.198130000	6.033200000	3.074274000
N	3.260788000	6.144988000	4.140377000
C	8.154897000	7.696367000	6.819160000
C	4.630908000	9.343622000	3.730298000
C	8.753088000	8.527785000	5.839046000
C	4.584592000	6.378500000	4.232039000
C	6.245543000	7.476619000	5.549026000
C	8.033750000	8.961508000	4.765203000
C	6.684723000	8.547972000	4.644647000
C	2.539976000	10.114364000	4.360436000
H	2.225749000	11.113166000	4.660791000
H	1.880325000	9.388881000	4.843022000
C	2.584574000	9.939949000	2.836267000
H	1.743975000	9.364571000	2.450595000
H	2.609404000	10.902806000	2.316203000
C	4.378657000	10.064235000	6.114539000
H	4.168741000	9.191488000	6.737073000
H	3.865573000	10.930100000	6.530275000
H	5.447606000	10.263237000	6.131966000
C	6.624890000	5.830339000	2.903628000
H	7.080237000	6.643741000	2.336464000
H	6.788167000	4.894466000	2.368660000
H	7.116650000	5.754602000	3.870833000
C	4.245297000	5.391453000	2.163343000
H	4.431581000	4.314242000	2.131345000
H	4.345098000	5.786857000	1.153322000
C	4.372167000	9.029115000	1.296457000
H	4.523993000	9.985102000	0.786192000
H	3.670752000	8.430630000	0.714952000
H	5.323814000	8.510470000	1.360512000
C	2.290786000	6.408804000	5.175698000
C	2.886718000	5.721043000	2.798338000
H	2.372153000	6.532350000	2.274230000
H	2.219055000	4.860304000	2.834123000
H	1.676069000	5.522773000	5.341621000
H	1.631657000	7.236631000	4.899724000
H	2.816723000	6.653678000	6.094961000
N	10.142345000	8.977707000	6.019154000
H	8.452863000	9.654453000	4.050261000
O	10.640526000	9.625694000	5.115600000
O	10.690965000	8.663269000	7.061560000
H	8.700168000	7.456721000	7.723046000

L11
E [Eh]: −1173.060595882247

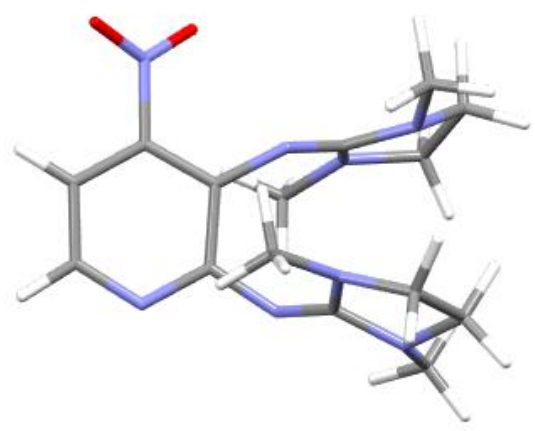

N	7.141545000	6.897440000	6.439393000
N	3.947197000	9.997096000	4.722574000
N	3.744262000	9.296171000	2.618064000

$(L11)^{\cdot+}$
E [Eh]: −1172.838356071952

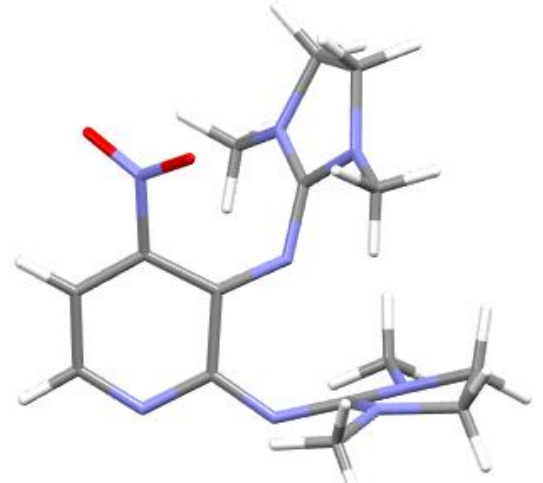

N	7.521022000	6.755054000	6.331007000
N	3.896011000	10.322961000	4.724543000
N	3.909617000	9.623092000	2.624343000

N	5.069313000	6.731299000	5.401433000
N	5.771920000	8.836221000	3.656283000
N	5.176527000	5.867283000	3.155334000
N	3.244056000	5.835718000	4.259828000
C	8.310327000	7.522624000	6.628070000
C	4.595880000	9.335134000	3.713287000
C	8.701823000	8.631353000	5.909373000
C	4.577093000	6.206055000	4.340086000
C	6.302151000	7.337963000	5.509408000
C	7.804319000	9.121185000	4.963155000
C	6.585534000	8.501024000	4.708418000
C	2.577626000	10.300315000	4.343119000
H	2.280047000	11.282789000	4.711423000
H	1.876682000	9.555183000	4.740651000
C	2.654126000	10.237255000	2.818151000
H	1.732060000	9.879261000	2.359123000
H	2.896706000	11.221729000	2.393993000
C	4.358057000	10.026856000	6.108460000
H	4.124784000	9.094622000	6.631088000
H	3.842538000	10.852214000	6.600026000
H	5.426424000	10.212642000	6.174993000
C	6.603713000	5.719125000	2.968256000
H	7.042635000	6.605614000	2.505198000
H	6.788271000	4.855955000	2.326091000
H	7.098390000	5.542901000	3.921381000
C	4.238022000	5.211214000	2.260388000
H	4.392679000	4.124372000	2.262536000
H	4.345105000	5.570771000	1.235456000
C	4.272931000	9.081561000	1.291822000
H	4.693665000	9.999396000	0.861268000
H	3.477499000	8.719331000	0.638449000
H	5.061834000	8.336165000	1.350767000
C	2.262396000	6.391865000	5.156071000
C	2.889811000	5.588689000	2.873538000
H	2.486488000	6.499836000	2.409429000
H	2.146788000	4.795134000	2.785227000
H	1.449143000	5.678878000	5.302864000
H	1.836052000	7.326592000	4.767571000
H	2.740756000	6.592791000	6.111344000
H	8.955954000	7.107835000	7.393876000
H	9.649548000	9.122194000	6.071133000
N	8.162021000	10.350222000	4.249453000
O	7.280966000	11.176669000	4.061861000
O	9.329235000	10.486981000	3.909798000

N	5.883986000	5.802706000	5.071754000
N	5.015574000	8.284791000	4.196254000
N	5.266539000	5.412216000	2.821506000
N	3.671581000	5.426059000	4.354462000
C	8.236355000	7.799322000	6.665247000
C	4.339833000	9.384241000	3.874141000
C	8.055540000	9.088227000	6.115574000
C	4.979166000	5.623422000	4.117978000
C	6.518519000	6.892935000	5.425958000
C	7.035020000	9.275139000	5.217928000
C	6.135885000	8.220740000	4.891154000
C	3.045566000	11.290019000	4.022726000
H	3.321950000	12.309546000	4.286850000
H	1.999821000	11.126959000	4.299072000
C	3.318078000	10.962489000	2.547836000
H	2.414796000	10.957176000	1.940043000
H	4.040629000	11.653572000	2.106545000
C	3.904166000	10.248375000	6.172391000
H	2.879624000	10.282097000	6.549779000
H	4.463940000	11.084100000	6.593995000
H	4.364284000	9.317884000	6.491802000
C	6.600083000	5.232762000	2.284337000
H	6.720543000	5.827496000	1.378184000
H	6.779187000	4.181426000	2.043793000
H	7.333019000	5.552521000	3.019443000
C	4.083910000	4.905581000	2.121326000
H	4.167388000	3.823054000	1.983646000
H	3.976901000	5.372871000	1.143400000
C	4.508991000	9.008355000	1.455867000
H	5.357561000	9.596976000	1.098001000
H	3.761839000	8.934339000	0.666506000
H	4.855613000	8.013382000	1.724301000
C	3.004752000	5.854983000	5.563738000
C	2.954533000	5.277776000	3.089322000
H	2.483713000	6.228585000	2.816380000
H	2.184276000	4.511962000	3.161258000
H	2.168067000	5.189372000	5.771161000
H	2.631891000	6.879703000	5.465131000
H	3.707644000	5.805996000	6.392235000
H	9.024199000	7.638882000	7.392901000
H	8.710925000	9.906085000	6.377557000
N	6.924671000	10.590919000	4.580492000
O	7.176073000	11.572687000	5.253408000
O	6.611619000	10.614686000	3.394235000

L12
E [Eh]: −1377.565967640405

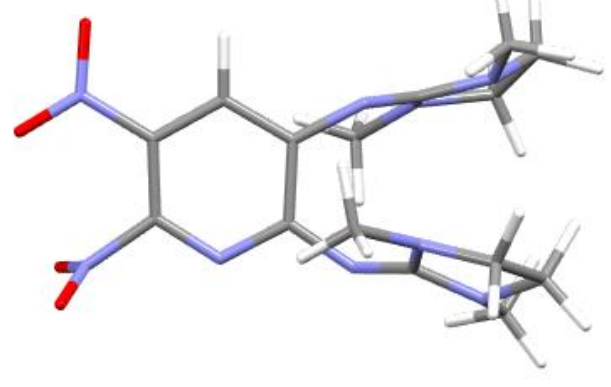

N	7.154271000	7.050416000	6.490747000
N	4.024691000	9.972666000	4.684455000
N	3.772751000	9.275573000	2.583745000
N	5.152778000	6.726641000	5.392814000
N	5.832984000	8.818288000	3.556623000
N	5.215003000	5.862273000	3.152162000
N	3.292739000	5.888302000	4.265142000
C	8.287900000	7.682897000	6.655057000
C	4.653356000	9.311317000	3.655695000
C	8.727683000	8.707998000	5.830865000
C	4.626282000	6.223340000	4.328591000
C	6.337949000	7.385137000	5.481312000
C	7.880980000	9.106072000	4.794864000
C	6.660116000	8.486287000	4.612681000
C	2.646052000	10.276964000	4.333756000
H	2.357467000	11.258410000	4.711312000
H	1.955283000	9.532155000	4.748028000
C	2.686496000	10.214813000	2.808668000
H	1.754666000	9.856510000	2.370531000

(L12)$^{\cdot+}$
E [Eh]: −1377.318635659413

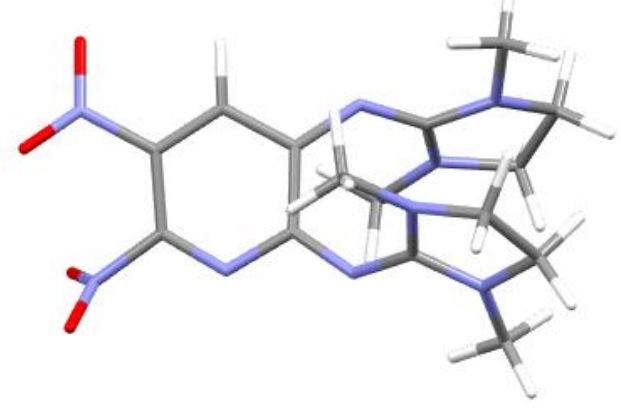

N	7.030320000	7.207057000	6.609061000
N	3.956312000	9.848167000	4.755254000
N	3.851943000	9.229447000	2.630380000
N	5.205146000	6.742829000	5.329790000
N	5.945146000	9.098436000	3.668578000
N	5.198890000	6.027441000	3.043008000
N	3.288860000	6.147456000	4.156186000
C	8.204093000	7.724841000	6.743766000
C	4.645918000	9.337540000	3.719146000
C	8.810881000	8.572266000	5.782094000
C	4.614117000	6.366916000	4.214627000
C	6.295252000	7.466968000	5.499889000
C	8.058567000	8.985207000	4.722969000
C	6.714307000	8.551319000	4.603405000
C	2.561478000	10.094497000	4.383691000
H	2.254407000	11.092555000	4.693534000
H	1.906949000	9.368543000	4.871939000
C	2.585151000	9.928230000	2.857636000
H	1.744615000	9.346799000	2.481254000

H	2.917903000	11.199291000	2.378138000
C	4.432428000	9.983368000	6.073928000
H	4.148427000	9.066085000	6.597366000
H	3.956179000	10.833552000	6.562800000
H	5.507642000	10.110792000	6.152826000
C	6.639694000	5.695471000	2.959483000
H	7.080963000	6.564135000	2.467191000
H	6.812047000	4.810245000	2.345272000
H	7.137779000	5.544324000	3.915457000
C	4.260976000	5.208538000	2.268153000
H	4.412346000	4.122223000	2.275278000
H	4.362543000	5.563165000	1.241543000
C	4.258117000	9.047722000	1.243175000
H	4.650474000	9.964842000	0.784920000
H	3.445439000	8.667496000	0.622158000
H	5.058994000	8.314415000	1.285891000
C	2.322788000	6.430159000	5.182629000
C	2.919076000	5.596602000	2.892101000
H	2.500387000	6.490878000	2.411371000
H	2.180769000	4.795800000	2.840347000
H	1.532276000	5.699200000	5.360145000
H	1.863953000	7.347792000	4.793632000
H	2.821604000	6.652362000	6.122685000
N	9.047610000	7.208841000	7.847283000
N	10.008703000	9.372820000	5.999403000
H	8.186037000	9.910832000	4.143310000
O	10.237772000	10.348993000	5.295119000
O	10.783674000	8.917707000	6.828400000
O	8.891530000	7.846810000	8.869637000
O	9.700296000	6.195542000	7.713043000

H	2.593858000	10.893310000	2.341729000
C	4.427937000	10.048120000	6.111127000
H	4.237019000	9.175759000	6.740072000
H	3.913256000	10.909544000	6.533698000
H	5.494568000	10.260055000	6.110262000
C	6.621591000	5.821842000	2.838862000
H	7.070085000	6.647741000	2.284607000
H	6.767629000	4.900582000	2.275013000
H	7.131916000	5.714647000	3.793750000
C	4.220253000	5.396599000	2.149952000
H	4.403773000	4.319783000	2.103041000
H	4.295416000	5.802076000	1.142025000
C	4.359056000	9.041932000	1.285444000
H	4.494312000	10.002064000	0.778736000
H	3.654392000	8.440447000	0.711115000
H	5.316244000	8.531570000	1.331685000
C	2.348712000	6.389403000	5.225317000
C	2.878888000	5.723299000	2.823353000
H	2.348281000	6.534108000	2.315527000
H	2.214145000	4.861443000	2.878052000
H	1.747851000	5.495425000	5.398557000
H	1.674766000	7.213916000	4.978549000
H	2.899778000	6.627940000	6.131321000
N	8.851591000	7.469673000	8.061325000
N	10.212075000	9.004442000	5.890091000
H	8.462560000	9.685279000	4.005586000
O	10.534839000	9.997764000	5.266624000
O	10.938353000	8.307690000	6.574458000
O	8.940515000	8.456846000	8.766850000
O	9.171857000	6.336245000	8.316793000

L13

E [Eh]: −1047.165539408291

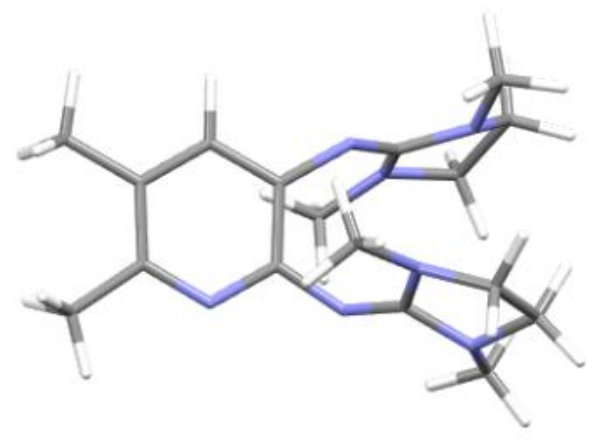

N	7.017056000	7.058114000	6.427789000
N	3.937686000	10.028755000	4.655111000
N	3.686816000	9.344084000	2.549432000
N	4.957807000	6.860497000	5.358655000
N	5.754697000	8.893538000	3.513513000
N	5.153725000	5.851828000	3.173095000
N	3.198529000	5.802497000	4.249697000
C	8.208043000	7.636729000	6.608984000
C	4.575013000	9.375199000	3.619836000
C	8.666590000	8.672527000	5.786955000
C	4.516873000	6.240359000	4.330893000
C	6.211999000	7.457236000	5.445347000
C	7.818396000	9.100996000	4.770045000
C	6.566071000	8.528832000	4.584427000
C	2.551947000	10.298789000	4.313257000
H	2.234998000	11.265026000	4.708768000
H	1.881326000	9.526424000	4.711824000
C	2.592582000	10.265931000	2.787106000
H	1.664252000	9.904984000	2.342489000
H	2.810354000	11.263172000	2.376035000
C	4.342993000	10.023595000	6.044377000
H	4.057781000	9.099413000	6.554762000
H	3.868786000	10.871980000	6.540692000
H	5.419558000	10.140738000	6.122494000
C	6.592192000	5.702302000	3.057518000
H	7.063698000	6.632200000	2.738306000
H	6.799613000	4.930309000	2.315840000
H	7.034151000	5.394935000	4.006040000
C	4.267934000	5.046972000	2.347013000
H	4.456811000	3.974605000	2.493332000
H	4.391447000	5.277531000	1.287628000

$(L13)^{\cdot+}$

E [Eh]: −1046.960085802884

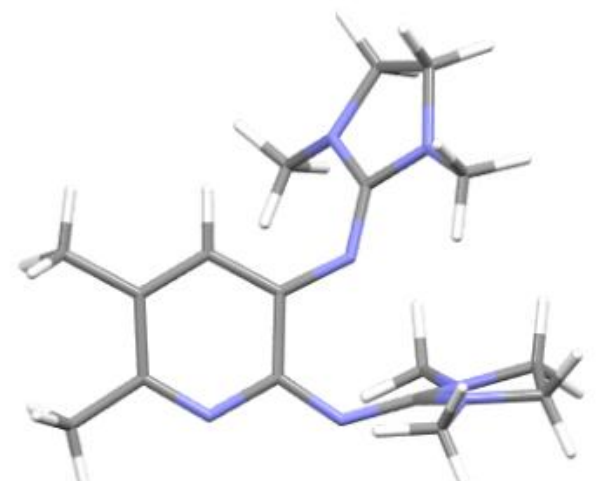

N	7.582980000	6.571078000	6.153338000
N	4.120140000	10.356696000	4.576408000
N	3.931675000	9.539079000	2.516708000
N	5.954252000	5.709740000	4.816022000
N	5.181183000	8.264497000	4.001712000
N	5.035023000	5.267198000	2.692794000
N	3.632162000	5.594036000	4.371022000
C	8.297723000	7.574635000	6.596818000
C	4.482574000	9.340585000	3.741577000
C	8.073371000	8.937300000	6.179852000
C	4.926386000	5.602548000	3.993457000
C	6.567543000	6.780036000	5.275687000
C	7.027512000	9.179332000	5.325319000
C	6.205200000	8.132940000	4.860137000
C	3.145572000	11.224932000	3.907404000
H	3.342704000	12.273981000	4.122547000
H	2.135629000	10.982572000	4.256224000
C	3.334747000	10.868097000	2.432623000
H	2.398373000	10.848179000	1.876429000
H	4.021210000	11.558937000	1.930949000
C	4.045348000	10.242696000	6.021828000
H	3.017142000	10.033160000	6.334545000
H	4.363719000	11.178140000	6.482708000
H	4.691161000	9.447577000	6.376281000
C	6.271342000	4.864051000	2.055666000
H	6.351284000	5.326064000	1.071029000
H	6.310950000	3.777061000	1.939826000
H	7.108625000	5.182190000	2.670376000
C	3.732805000	4.864615000	2.163215000
H	3.666431000	3.772566000	2.118032000
H	3.574835000	5.263237000	1.161925000

C	4.164836000	9.114832000	1.209179000	C	4.388033000	8.850818000	1.327275000
H	4.558531000	10.030145000	0.745815000	H	5.168993000	9.418749000	0.811053000
H	3.349255000	8.734590000	0.591176000	H	3.548832000	8.712609000	0.645339000
H	4.964188000	8.379243000	1.252981000	H	4.787699000	7.882487000	1.617882000
C	2.175120000	6.402360000	5.066498000	C	3.149280000	6.155673000	5.609820000
C	2.893244000	5.442336000	2.878126000	C	2.769978000	5.453764000	3.200858000
H	2.490701000	6.304842000	2.325848000	H	2.392894000	6.436130000	2.894094000
H	2.167707000	4.629197000	2.828536000	H	1.922324000	4.803911000	3.412484000
H	1.369088000	5.686124000	5.236328000	H	2.270191000	5.603175000	5.939887000
H	1.748094000	7.298790000	4.595329000	H	2.883506000	7.211227000	5.490948000
H	2.615365000	6.683053000	6.020083000	H	3.923984000	6.065569000	6.367846000
C	9.043319000	7.110399000	7.743991000	C	9.391193000	7.258138000	7.569129000
C	10.016784000	9.301738000	5.992510000	C	8.955297000	10.043139000	6.669784000
H	8.122918000	9.898712000	4.101835000	H	6.825733000	10.189554000	4.991395000
H	10.202594000	10.076919000	5.248425000	H	8.651708000	11.002306000	6.252765000
H	10.821502000	8.564794000	5.917497000	H	9.998564000	9.867736000	6.395991000
H	10.103535000	9.759951000	6.982060000	H	8.928017000	10.120113000	7.759635000
H	9.281695000	7.896248000	8.467124000	H	9.250225000	7.799435000	8.508424000
H	9.997858000	6.708716000	7.390049000	H	10.365710000	7.559984000	7.176259000
H	8.499504000	6.318354000	8.254299000	H	9.403527000	6.190531000	7.769719000

L14
E [Eh]: −1197.585955961747

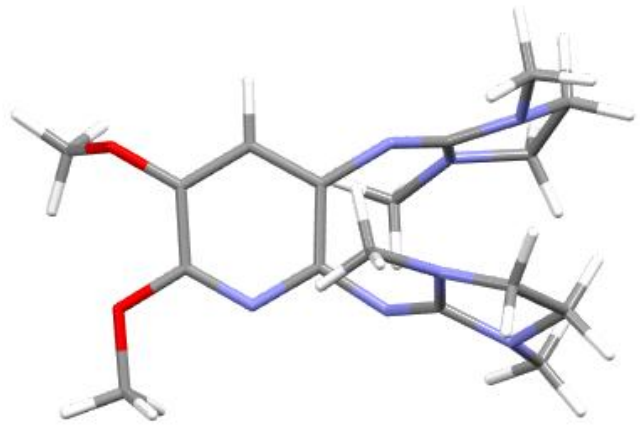

$(L14)^{\cdot+}$
E [Eh]: −1197.386529710870

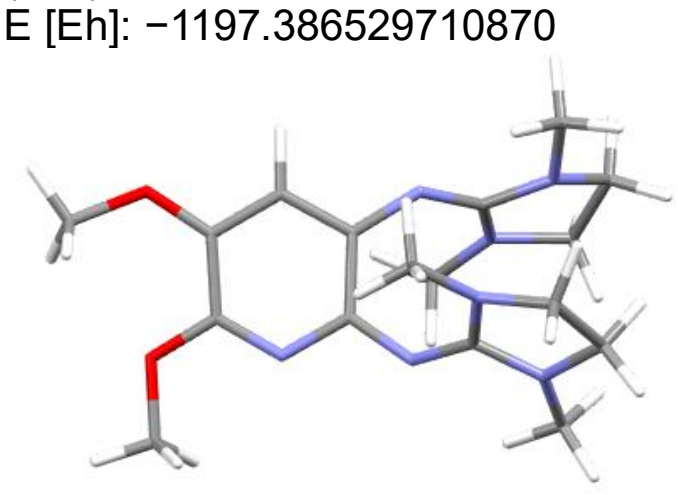

N	7.445159000	7.179291000	6.319361000	N	7.372569000	7.417715000	6.479664000
N	4.256764000	10.069481000	4.625917000	N	3.999872000	9.892954000	4.659698000
N	3.795086000	9.276827000	2.593320000	N	3.791741000	9.147566000	2.579144000
N	5.301378000	6.897847000	5.459699000	N	5.434106000	6.850648000	5.416407000
N	5.926111000	8.804286000	3.394221000	N	5.922997000	9.023626000	3.517545000
N	5.232858000	5.835677000	3.293510000	N	5.257032000	6.017203000	3.171989000
N	3.403699000	5.871579000	4.567767000	N	3.455965000	6.099756000	4.453432000
C	8.629364000	7.753239000	6.347380000	C	8.511764000	8.048801000	6.550833000
C	4.779095000	9.337641000	3.577372000	C	4.660665000	9.311918000	3.624989000
C	9.018832000	8.730023000	5.418281000	C	8.969844000	8.959053000	5.522204000
C	4.735766000	6.268059000	4.497889000	C	4.776016000	6.389672000	4.383536000
C	6.549014000	7.501944000	5.377695000	C	6.525475000	7.613751000	5.450521000
C	8.093391000	9.086274000	4.453286000	C	8.069123000	9.255547000	4.519981000
C	6.827810000	8.501446000	4.416711000	C	6.794278000	8.678140000	4.484458000
C	2.852854000	10.362514000	4.393965000	C	2.590716000	10.091757000	4.336976000
H	2.597579000	11.357571000	4.761504000	H	2.256635000	11.078590000	4.656260000
H	2.200674000	9.633367000	4.892586000	H	1.968018000	9.342286000	4.835463000
C	2.754449000	10.247137000	2.874897000	C	2.579486000	9.926914000	2.814964000
H	1.779471000	9.894677000	2.535929000	H	1.696685000	9.402886000	2.450764000
H	2.962983000	11.213154000	2.391001000	H	2.640513000	10.895874000	2.305536000
C	4.785652000	10.128667000	5.972610000	C	4.505365000	10.114811000	5.995852000
H	4.562288000	9.224381000	6.545538000	H	4.326814000	9.258407000	6.651487000
H	4.341994000	10.991317000	6.472115000	H	4.011063000	10.990997000	6.414545000
H	5.862790000	10.264754000	5.949875000	H	5.573648000	10.313887000	5.962321000
C	6.639234000	5.648701000	3.004373000	C	6.654827000	5.885192000	2.820764000
H	7.054413000	6.502353000	2.465679000	H	6.978520000	6.690941000	2.159517000
H	6.754622000	4.750735000	2.394216000	H	6.807517000	4.929143000	2.317601000
H	7.206751000	5.510267000	3.923124000	H	7.272342000	5.899555000	3.715846000
C	4.226769000	5.105433000	2.542828000	C	4.223901000	5.343436000	2.386636000
H	4.387994000	4.021918000	2.622794000	H	4.423753000	4.268029000	2.349314000
H	4.241050000	5.376550000	1.485402000	H	4.199058000	5.722338000	1.365224000
C	4.147655000	8.970496000	1.229546000	C	4.259232000	8.935615000	1.225514000
H	4.527243000	9.849763000	0.690823000	H	4.498607000	9.881955000	0.727930000
H	3.270363000	8.591039000	0.702337000	H	3.484872000	8.425784000	0.652102000
H	4.926401000	8.212055000	1.240409000	H	5.154686000	8.321929000	1.251618000
C	2.495900000	6.510461000	5.486194000	C	2.595233000	6.430425000	5.561141000
C	2.934635000	5.531182000	3.236860000	C	2.940291000	5.663445000	3.165013000
H	2.494274000	6.410354000	2.743838000	H	2.368002000	6.468911000	2.693407000
H	2.186627000	4.737631000	3.264777000	H	2.288645000	4.796607000	3.275831000
H	1.687417000	5.823787000	5.743795000	H	1.969825000	5.572677000	5.813536000
H	2.052987000	7.420799000	5.058418000	H	1.940497000	7.274085000	5.322538000
H	3.044032000	6.776766000	6.386275000	H	3.212832000	6.685242000	6.418606000

O	9.528797000	7.390283000	7.300467000
O	10.275051000	9.285174000	5.400146000
H	8.360877000	9.832781000	3.716733000
C	9.106318000	6.401672000	8.236020000
H	9.950760000	6.259320000	8.907298000
H	8.857635000	5.465560000	7.733775000
H	8.228457000	6.735088000	8.791873000
C	10.585805000	10.124269000	6.510357000
H	9.880507000	10.960519000	6.568467000
H	11.588710000	10.509693000	6.333477000
H	10.566714000	9.565226000	7.447373000

O	9.302758000	7.873954000	7.606632000
O	10.141612000	9.595793000	5.487890000
H	8.340743000	9.984644000	3.769508000
C	8.856359000	6.975702000	8.641746000
H	9.649890000	6.985840000	9.382451000
H	8.712683000	5.976427000	8.234727000
H	7.919480000	7.328234000	9.069149000
C	11.285289000	9.225128000	6.279068000
H	11.144006000	9.505177000	7.319869000
H	12.108424000	9.785661000	5.845344000
H	11.478783000	8.156402000	6.203011000

L15
E [Eh]: −1167.052793526906

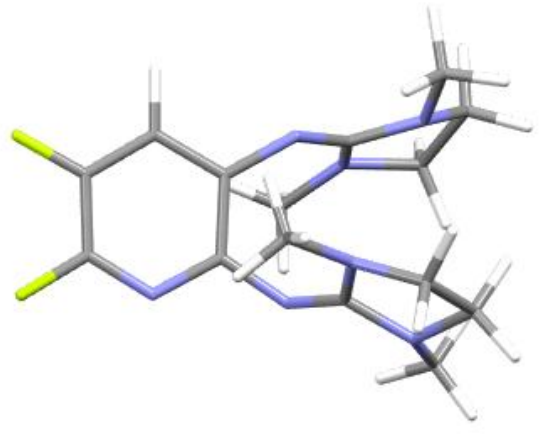

N	7.041274000	7.051048000	6.429768000
N	3.939787000	10.018182000	4.653249000
N	3.707343000	9.335533000	2.546207000
N	4.981050000	6.841987000	5.367017000
N	5.768351000	8.891870000	3.523391000
N	5.149672000	5.860261000	3.168258000
N	3.205748000	5.813321000	4.260801000
C	8.194278000	7.635901000	6.599003000
C	4.583850000	9.369222000	3.622600000
C	8.641947000	8.674603000	5.802178000
C	4.524159000	6.238631000	4.332463000
C	6.223612000	7.445431000	5.448261000
C	7.820629000	9.121073000	4.784941000
C	6.573873000	8.529041000	4.594346000
C	2.556369000	10.291436000	4.300430000
H	2.239756000	11.259179000	4.691676000
H	1.881998000	9.521623000	4.696698000
C	2.607893000	10.255078000	2.774639000
H	1.684201000	9.890263000	2.324220000
H	2.826733000	11.251026000	2.362264000
C	4.339102000	10.020907000	6.044488000
H	4.062853000	9.094713000	6.555871000
H	3.852044000	10.863615000	6.537083000
H	5.413347000	10.154761000	6.128283000
C	6.586751000	5.704555000	3.040828000
H	7.060902000	6.635992000	2.730487000
H	6.785115000	4.942617000	2.286931000
H	7.033251000	5.378724000	3.981110000
C	4.252439000	5.066368000	2.341516000
H	4.441620000	3.992994000	2.475466000
H	4.366915000	5.308377000	1.284011000
C	4.195516000	9.104119000	1.209149000
H	4.595590000	10.018060000	0.749612000
H	3.383067000	8.726972000	0.585615000
H	4.991624000	8.365553000	1.258060000
C	2.191790000	6.395378000	5.102462000
C	2.883917000	5.459151000	2.890825000
H	2.474408000	6.324524000	2.349099000
H	2.158216000	4.646208000	2.846791000
H	1.391818000	5.672513000	5.271638000
H	1.753999000	7.296807000	4.651915000
H	2.643483000	6.661683000	6.054684000
F	8.978064000	7.186153000	7.596089000
F	9.847894000	9.240643000	6.011351000
H	8.142628000	9.930362000	4.143734000

(L15)•+
E [Eh]: −1166.830926494654

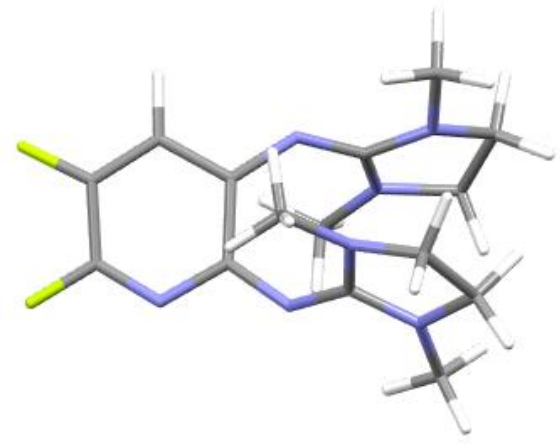

N	6.996515000	7.134568000	6.603250000
N	3.862466000	9.838293000	4.655019000
N	3.849032000	9.187099000	2.536513000
N	5.112450000	6.763714000	5.372148000
N	5.889091000	9.043422000	3.664745000
N	5.080133000	6.020071000	3.094119000
N	3.182634000	6.156871000	4.227036000
C	8.159878000	7.662620000	6.748163000
C	4.603030000	9.303926000	3.660576000
C	8.732024000	8.588185000	5.828482000
C	4.512549000	6.375113000	4.272510000
C	6.236404000	7.452168000	5.527212000
C	7.976928000	9.021593000	4.783744000
C	6.650038000	8.542932000	4.638340000
C	2.487353000	10.071646000	4.215071000
H	2.148424000	11.060700000	4.520887000
H	1.815139000	9.329182000	4.653191000
C	2.597216000	9.926779000	2.692740000
H	1.761491000	9.380370000	2.257629000
H	2.668390000	10.900310000	2.196047000
C	4.258291000	10.020425000	6.035218000
H	4.017758000	9.147876000	6.647312000
H	3.737581000	10.889588000	6.434823000
H	5.327575000	10.208894000	6.095124000
C	6.498946000	5.804618000	2.880395000
H	6.958801000	6.644333000	2.356903000
H	6.631690000	4.902953000	2.282791000
H	7.009265000	5.657405000	3.830359000
C	4.089181000	5.390508000	2.217177000
H	4.260525000	4.310998000	2.177025000
H	4.156751000	5.786450000	1.204670000
C	4.425151000	8.991405000	1.221771000
H	4.656216000	9.947654000	0.741632000
H	3.718953000	8.447443000	0.594449000
H	5.341052000	8.416375000	1.316870000
C	2.253428000	6.419202000	5.299158000
C	2.757447000	5.735940000	2.899529000
H	2.234856000	6.552261000	2.391512000
H	2.082316000	4.882225000	2.959340000
H	1.645526000	5.532882000	5.488433000
H	1.583890000	7.247290000	5.051209000
H	2.814582000	6.662963000	6.197513000
F	8.878398000	7.331104000	7.806194000
F	9.964783000	9.030260000	6.035636000
H	8.354733000	9.765844000	4.097047000

Pyridinium salts

$[Py+Bn]^+$
E [Eh]: -518.935444854840

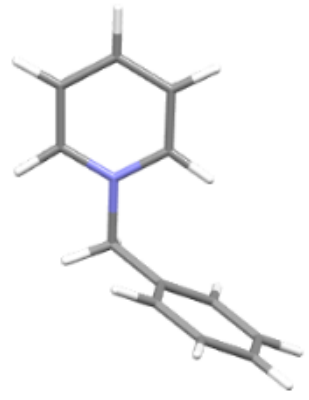

N	6.116604000	7.047270000	6.087694000
C	6.896234000	8.141963000	6.000873000
H	7.476086000	8.389034000	6.877952000
C	6.936696000	8.890200000	4.844033000
H	7.575834000	9.760163000	4.799727000
C	5.912558000	4.804263000	7.188492000
C	5.355946000	6.657169000	5.047963000
C	6.155953000	8.505057000	3.760235000
H	6.174108000	9.076520000	2.841992000
C	5.355981000	7.373887000	3.869959000
C	6.990754000	4.040490000	6.738153000
H	7.949335000	4.510695000	6.550660000
C	4.689865000	4.186546000	7.445504000
H	3.853927000	4.771519000	7.811082000
C	6.068596000	6.279604000	7.388870000
H	5.237834000	6.694852000	7.958939000
H	6.996078000	6.520605000	7.904649000
C	5.618502000	2.063993000	6.795311000
H	5.506702000	0.998168000	6.647236000
C	6.842866000	2.675736000	6.539926000
H	7.682720000	2.086235000	6.197414000
C	4.543823000	2.817754000	7.250316000
H	3.595292000	2.341894000	7.459832000
H	4.772638000	5.760997000	5.196089000
H	4.735216000	7.040754000	3.050829000

$[L1+Bn]^+$
E [Eh]: −1964.373200530203

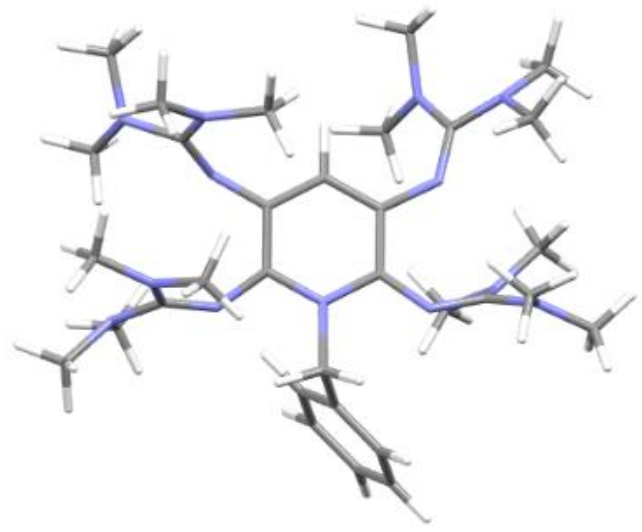

N	3.569728000	5.462343000	5.102193000
N	3.749579000	3.941635000	3.299344000
N	5.572042000	4.425133000	4.655121000
N	4.088284000	6.856891000	2.738772000
N	5.597494000	7.210836000	1.050271000
N	3.336123000	7.426987000	0.538421000
N	0.250862000	9.706726000	3.810148000
N	-0.752184000	11.764249000	3.672798000
N	1.496858000	11.713615000	4.270013000
N	-0.285040000	7.875055000	5.958154000
N	-2.255874000	8.854961000	6.590481000
N	-0.262477000	9.960632000	7.077134000
N	1.689573000	6.767421000	5.460691000
C	2.742926000	6.417856000	4.652333000
C	2.968933000	7.187672000	3.480761000
C	2.160044000	8.295715000	3.273258000
H	2.323427000	8.892565000	2.388690000
C	1.142538000	8.692269000	4.143894000
C	0.856357000	7.848509000	5.231867000
C	4.275756000	4.660410000	4.334737000
C	4.503619000	3.704064000	2.078617000
H	4.633474000	2.636327000	1.884302000
H	5.477556000	4.178224000	2.144570000
H	3.971117000	4.144038000	1.231495000
C	2.317581000	3.724274000	3.208002000
H	2.141308000	2.797949000	2.660494000
H	1.801044000	4.535350000	2.686106000
H	1.891301000	3.626190000	4.199703000
C	6.202399000	3.122351000	4.505290000
H	5.473256000	2.389962000	4.172232000
H	6.597374000	2.799586000	5.471904000
H	7.030134000	3.151825000	3.791807000
C	6.280671000	5.379410000	5.490297000
H	6.181212000	5.139434000	6.553381000
H	5.885453000	6.375289000	5.316529000
H	7.339305000	5.359907000	5.226219000
C	4.294776000	7.158807000	1.497205000
C	6.662819000	7.248336000	2.034878000
H	7.561074000	7.650240000	1.563890000
H	6.891710000	6.251866000	2.431367000
H	6.375245000	7.882728000	2.868174000
C	5.978373000	6.619777000	-0.225503000
H	6.525389000	5.684339000	-0.061898000
H	6.623676000	7.294683000	-0.791924000
H	5.097252000	6.397190000	-0.818643000
C	3.519179000	8.445799000	-0.481181000
C	0.376228000	10.982647000	3.915861000
C	-2.029925000	11.074249000	3.595955000
H	-2.822661000	11.823124000	3.625970000
H	-2.143861000	10.400085000	4.438600000
H	-2.130578000	10.489102000	2.675092000
C	-0.642253000	12.846753000	2.697167000
H	-0.889552000	12.487588000	1.690272000
H	0.365242000	13.248743000	2.673752000
H	-1.333044000	13.652111000	2.951495000
C	1.411308000	12.990776000	4.963053000
C	2.853045000	11.234937000	4.073487000
H	3.512208000	12.101566000	4.008261000
H	2.938123000	10.684235000	3.143092000
H	3.212069000	10.595867000	4.885767000
C	-0.887880000	8.890306000	6.505872000

C	-2.967821000	7.933615000	5.717146000
H	-2.502141000	7.922590000	4.735866000
H	-3.998946000	8.276559000	5.621994000
H	-2.971173000	6.913880000	6.115782000
C	-2.945661000	9.146773000	7.843389000
H	-3.367702000	8.224254000	8.253581000
H	-3.759951000	9.858150000	7.690307000
H	-2.255446000	9.553046000	8.575436000
C	-0.898673000	11.260501000	7.230697000
H	-1.823993000	11.293065000	6.665258000
H	-0.234782000	12.031570000	6.844198000
H	-1.110828000	11.489214000	8.278866000
C	1.146248000	9.905920000	7.420416000
H	1.445524000	8.880993000	7.621683000
H	1.311403000	10.494043000	8.324343000
H	1.778820000	10.304734000	6.623738000
C	1.449115000	5.922917000	6.649754000
H	0.823244000	6.501599000	7.321415000
H	2.416131000	5.740425000	7.111068000
C	0.784596000	4.612455000	6.314517000
C	-0.361854000	4.574007000	5.521132000
H	-0.781188000	5.499873000	5.152038000
C	-0.956643000	3.358602000	5.207645000
H	-1.843444000	3.338224000	4.586883000
C	-0.420312000	2.169813000	5.692537000
H	-0.885393000	1.223652000	5.448198000
C	0.715016000	2.203358000	6.493650000
H	1.138563000	1.282782000	6.873993000
C	1.315764000	3.419824000	6.797779000
H	2.216362000	3.443627000	7.398994000
C	2.027829000	6.810490000	0.571267000
H	1.776225000	6.444818000	-0.428173000
H	1.243272000	7.506292000	0.887780000
H	2.030358000	5.969832000	1.256668000
H	4.472081000	8.947465000	-0.339563000
H	2.718418000	9.189786000	-0.408207000
H	3.489778000	8.022450000	-1.490097000
H	0.375570000	13.246241000	5.155686000
H	1.864961000	13.794136000	4.374503000
H	1.945727000	12.935380000	5.917121000

$[L2a+Bn]^+$
E [Eh]: −1239.296192650670

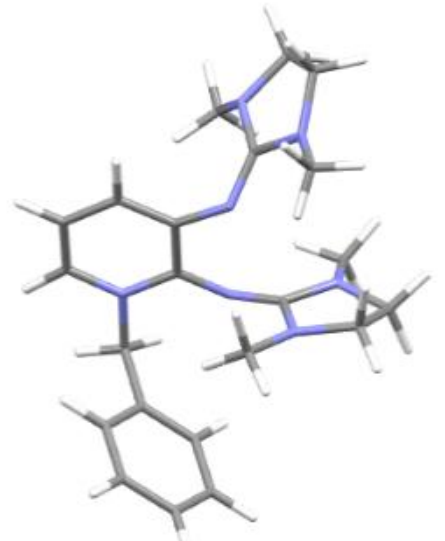

N	5.898556000	7.125956000	6.174012000
N	3.341813000	9.733907000	2.728184000
N	2.780773000	8.148410000	1.252338000
N	3.743512000	6.528278000	5.630044000
N	4.162982000	7.477094000	2.994492000
N	3.127361000	4.806538000	4.096556000
N	1.553542000	6.132411000	4.899389000
C	7.064177000	7.797218000	5.946415000
C	3.501068000	8.405615000	2.390347000
C	7.292747000	8.403343000	4.749363000
C	5.798886000	4.888879000	7.208563000
C	2.882751000	5.884639000	4.871010000
C	4.897334000	7.052970000	5.244794000
C	6.316515000	8.322698000	3.744344000
C	5.122749000	7.662445000	3.958531000
C	6.944871000	4.319634000	6.654112000
H	7.794043000	4.944359000	6.403275000
C	4.717880000	4.070997000	7.522228000
H	3.821911000	4.510524000	7.941395000
C	5.725382000	6.378377000	7.439138000

$[L2b+Bn]^+$
E [Eh]: −1241.677834059475

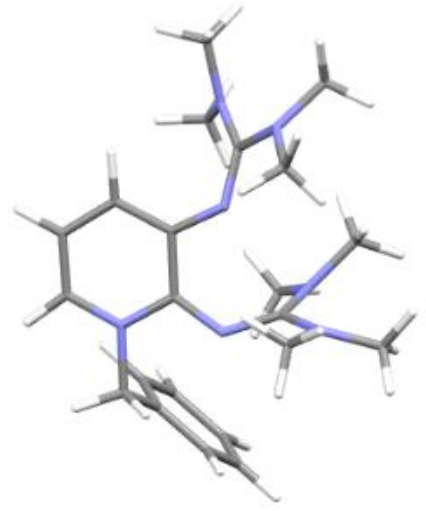

N	11.539340000	6.859831000	7.124382000
N	12.426367000	6.311274000	9.170599000
N	13.799773000	7.690564000	10.494042000
N	12.882938000	5.734019000	11.351577000
N	10.027115000	9.030990000	12.384622000
N	10.850574000	8.243344000	10.398226000
N	10.386233000	10.568608000	10.678936000
C	13.849459000	6.262461000	6.422817000
C	12.412103000	5.822143000	6.537553000
H	11.995698000	5.589569000	5.558504000
H	12.339060000	4.936397000	7.164242000
C	9.875608000	8.756292000	8.195873000
H	9.177312000	9.467679000	8.613893000
C	12.989802000	6.610313000	10.332899000
C	9.818409000	8.457147000	6.827456000
H	9.111980000	8.952146000	6.177915000
C	11.641883000	7.111170000	8.466092000
C	10.654149000	7.512536000	6.317201000
H	10.661379000	7.222565000	5.278840000
C	14.179110000	7.449842000	5.771286000

H	4.764001000	6.652733000	7.866029000
H	6.513740000	6.716082000	8.109638000
C	2.312020000	10.326972000	1.869242000
H	2.557213000	11.357555000	1.617177000
H	1.341072000	10.313037000	2.379687000
C	5.920025000	2.142327000	6.726140000
H	5.968325000	1.077161000	6.541670000
C	2.312132000	9.396108000	0.663025000
H	1.326346000	9.279635000	0.214031000
H	3.008241000	9.740803000	-0.111928000
C	7.005414000	2.953680000	6.411152000
H	7.900218000	2.521690000	5.982610000
C	3.371172000	10.190717000	4.107290000
H	2.515036000	9.808716000	4.676300000
H	3.331892000	11.278964000	4.110163000
H	4.288216000	9.887474000	4.601056000
C	4.778261000	2.702625000	7.286440000
H	3.933636000	2.074835000	7.538972000
C	4.436086000	4.338758000	3.701190000
H	4.797401000	4.862141000	2.812633000
H	4.377718000	3.270412000	3.497901000
H	5.138917000	4.485390000	4.517167000
C	1.921470000	4.427144000	3.366428000
H	1.830393000	3.344127000	3.301468000
H	1.945207000	4.841176000	2.353032000
C	3.092429000	7.027895000	0.391842000
H	3.844522000	7.295346000	-0.359665000
H	2.189588000	6.697656000	-0.122669000
H	3.481566000	6.215481000	0.998587000
C	0.910669000	7.023889000	5.835471000
C	0.820012000	5.073195000	4.214093000
H	0.008966000	5.483593000	3.614281000
H	0.396088000	4.369504000	4.939592000
H	7.770945000	7.802768000	6.760788000
H	6.485624000	8.789308000	2.782815000
H	8.220321000	8.930883000	4.585093000
H	0.129981000	7.594031000	5.329761000
H	1.654679000	7.707389000	6.235971000
H	0.457895000	6.469869000	6.664216000

H	13.398772000	8.070076000	5.346546000
C	10.778706000	8.126060000	9.036257000
C	10.428327000	9.261182000	11.099422000
C	13.844287000	8.429140000	11.747972000
H	13.557320000	9.466906000	11.561154000
H	14.844085000	8.424603000	12.187823000
H	13.136483000	8.008427000	12.454863000
C	14.864906000	5.479617000	6.963723000
H	14.612476000	4.563494000	7.482829000
C	15.504652000	7.851891000	5.669922000
H	15.750340000	8.774619000	5.160627000
C	14.358798000	8.374149000	9.339365000
H	14.524208000	7.669328000	8.532725000
H	15.316504000	8.810422000	9.623854000
H	13.711541000	9.178476000	8.976469000
C	11.804639000	4.756254000	11.344357000
H	10.940968000	5.167103000	10.831925000
H	11.533892000	4.526240000	12.375324000
H	12.107910000	3.830951000	10.846759000
C	9.329061000	11.486440000	11.080708000
H	8.838599000	11.886979000	10.188481000
H	9.719009000	12.329662000	11.657333000
H	8.585629000	10.964492000	11.675526000
C	16.193106000	5.875401000	6.856744000
H	16.974976000	5.257559000	7.278768000
C	9.724785000	7.672129000	12.800540000
H	9.272431000	7.124279000	11.979912000
H	9.028249000	7.708596000	13.638757000
H	10.626485000	7.136557000	13.117544000
C	16.515021000	7.064945000	6.213531000
H	17.548217000	7.375693000	6.131257000
C	11.303784000	11.081274000	9.683313000
H	12.154461000	10.414773000	9.584271000
H	11.664257000	12.062200000	10.001318000
H	10.835734000	11.190677000	8.699695000
C	10.340444000	9.950319000	13.471379000
H	10.994368000	9.452618000	14.193603000
H	9.438484000	10.272229000	13.996274000
H	10.858509000	10.824944000	13.090933000
C	13.957313000	5.485194000	12.304049000
H	14.859353000	6.005738000	11.996823000
H	14.168609000	4.414136000	12.325241000
H	13.687277000	5.799768000	13.314849000

$[L3+Bn]^+$
E [Eh]: −1698.856562958493

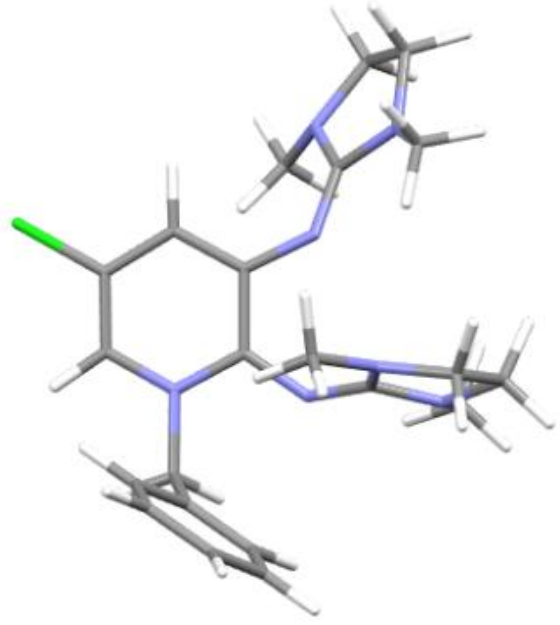

N	6.985381000	6.474901000	7.005910000
N	4.494746000	10.341698000	4.479528000
N	4.176841000	9.232920000	2.568563000
N	5.127526000	5.762033000	5.856266000
N	5.242282000	8.048410000	4.253487000
N	5.163268000	5.051514000	3.575883000
N	3.245064000	5.046550000	4.679187000
C	7.936973000	7.395139000	7.343078000
C	4.699511000	9.147681000	3.827043000
C	8.023175000	8.561792000	6.645012000
C	4.562567000	5.364000000	4.741878000
C	6.075039000	6.672757000	6.009359000
C	7.128436000	8.825311000	5.599039000
C	6.135304000	7.916986000	5.270913000
C	3.641522000	11.204823000	3.654686000
H	3.960787000	12.244035000	3.716063000

$[L4+Bn]^+$
E [Eh]: −1698.854326761390

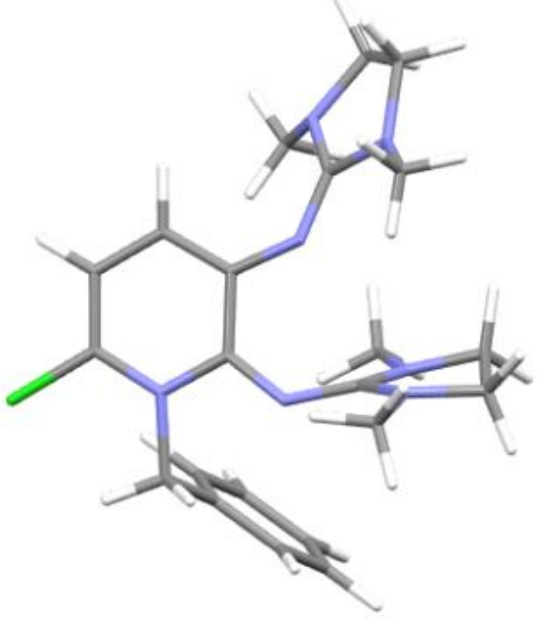

N	8.611169000	6.682944000	5.551271000
N	4.065644000	9.497777000	5.046817000
N	3.908107000	9.113334000	2.850316000
N	7.321191000	5.892180000	3.826042000
N	5.735530000	8.155223000	3.894046000
N	6.053963000	5.445002000	1.906248000
N	5.049947000	5.168653000	3.852209000
C	9.028100000	7.692795000	6.381290000
C	4.665122000	8.877871000	3.969873000
C	8.370580000	8.885994000	6.412749000
C	6.182908000	5.580525000	3.242276000
C	7.531842000	6.845643000	4.710567000
C	7.235522000	9.069716000	5.613679000
C	6.766106000	8.069338000	4.783119000
C	2.779463000	10.065381000	4.629743000
H	2.602425000	11.029080000	5.105361000

H	2.602353000	11.137470000	3.997341000
C	3.803461000	10.606870000	2.260444000
H	2.888025000	10.648832000	1.671083000
H	4.602434000	11.102497000	1.696191000
C	4.285849000	10.424243000	5.916043000
H	3.287553000	10.065369000	6.191778000
H	4.377371000	11.465860000	6.221261000
H	5.028989000	9.847009000	6.454229000
C	6.586160000	5.031774000	3.329965000
H	6.877664000	5.834747000	2.649438000
H	6.870638000	4.071783000	2.896134000
H	7.128517000	5.144658000	4.263106000
C	4.224275000	4.414487000	2.657412000
H	4.424258000	3.339503000	2.604470000
H	4.310795000	4.828134000	1.652382000
C	4.553313000	8.322252000	1.510399000
H	5.413325000	8.696806000	0.943634000
H	3.715354000	8.191808000	0.824954000
H	4.813065000	7.366087000	1.955636000
C	2.266354000	5.578514000	5.601951000
C	2.869753000	4.719203000	3.307780000
H	2.380330000	5.580382000	2.838506000
H	2.183815000	3.873600000	3.275811000
H	1.466776000	4.852463000	5.748203000
H	1.831951000	6.511228000	5.224602000
H	2.751115000	5.772321000	6.554997000
H	8.594101000	7.142203000	8.158107000
Cl	9.236925000	9.724270000	7.063156000
H	7.203933000	9.756557000	5.055358000
C	6.991122000	5.160753000	7.691006000
C	7.504867000	4.069916000	6.784010000
H	5.975397000	4.945767000	8.013034000
H	7.620293000	5.274941000	8.571661000
C	8.800480000	4.133493000	6.272378000
C	9.267392000	3.146314000	5.414286000
C	8.443017000	2.081833000	5.064456000
C	7.153180000	2.008780000	5.577533000
C	6.686071000	3.001298000	6.430804000
H	8.808520000	1.308455000	4.401599000
H	6.511018000	1.177717000	5.315944000
H	5.676031000	2.953993000	6.817084000
H	9.449680000	4.959571000	6.537897000
H	10.275725000	3.203064000	5.025754000

$[L4b+Bn]^+$
E [Eh]: −2305.878778963958

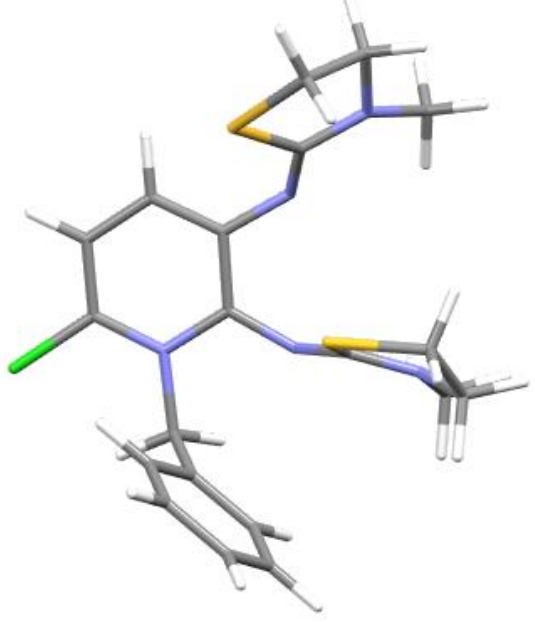

H	10.231714000	1.157561000	13.752808000
S	4.591503000	3.719543000	12.719856000
N	6.990285000	4.953958000	12.356022000
C	7.734664000	3.894869000	12.700297000
Cl	10.332553000	3.253107000	15.617125000
S	6.115101000	0.165490000	11.321725000
N	5.094303000	5.902007000	11.424331000
C	7.830526000	2.712095000	11.908026000
N	7.088033000	2.674105000	10.755909000
C	8.742285000	1.743588000	12.302846000
H	8.866569000	0.855384000	11.698730000
N	5.639764000	1.668865000	9.258797000
C	9.520256000	1.908246000	13.445750000
N	8.510226000	4.024783000	13.824940000
C	9.390661000	3.048768000	14.193442000
C	5.722685000	4.925807000	12.100212000

H	1.965403000	9.385100000	4.907578000
C	2.929268000	10.159116000	3.115972000
H	1.996488000	9.976733000	2.583684000
H	3.319472000	11.137144000	2.808897000
C	4.116142000	8.976827000	6.401368000
H	3.311522000	8.251252000	6.569862000
H	3.987799000	9.796889000	7.108509000
H	5.066208000	8.496060000	6.601678000
C	7.143072000	5.548074000	0.962847000
H	7.953431000	6.108023000	1.421404000
H	6.807545000	6.071771000	0.066834000
H	7.512050000	4.559134000	0.672894000
C	4.796235000	4.783786000	1.573799000
H	4.967003000	3.725561000	1.347165000
H	4.324844000	5.251643000	0.710508000
C	4.455244000	8.963305000	1.517904000
H	5.005722000	9.857103000	1.202588000
H	3.640502000	8.789969000	0.814762000
H	5.132991000	8.114090000	1.509538000
C	4.768195000	5.284158000	5.261695000
C	3.992223000	4.970619000	2.865862000
H	3.349244000	5.856697000	2.818186000
H	3.381706000	4.104632000	3.116730000
H	4.140708000	4.448241000	5.569322000
H	4.253363000	6.219882000	5.494721000
H	5.695695000	5.231888000	5.825630000
Cl	10.397601000	7.433782000	7.395112000
H	8.733059000	9.671447000	7.057068000
H	6.724681000	10.022704000	5.634690000
C	9.236757000	5.337121000	5.553140000
C	8.327413000	4.317842000	6.193668000
C	7.921439000	3.192368000	5.483233000
C	7.075169000	2.257149000	6.066773000
C	6.620717000	2.445483000	7.366776000
C	7.022753000	3.568039000	8.083429000
C	7.874425000	4.497982000	7.500450000
H	5.962894000	1.717609000	7.823322000
H	6.681891000	3.713464000	9.100218000
H	8.188857000	5.365113000	8.068430000
H	8.261688000	3.054562000	4.464803000
H	6.770114000	1.382838000	5.506458000
H	9.438165000	5.073780000	4.518628000
H	10.178772000	5.415504000	6.082922000

$[L5a+Bn]^+$
E [Eh]: −2158.411634054389

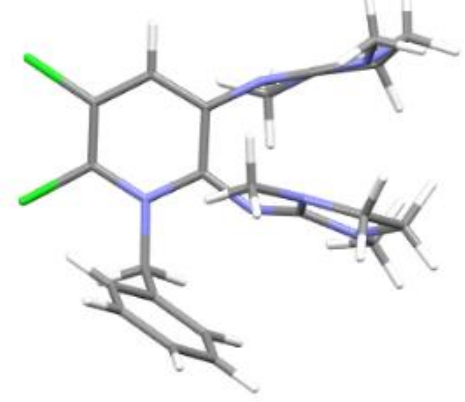

N	7.243618000	6.765904000	6.851191000
N	4.150935000	9.623270000	4.676337000
N	4.078859000	8.779091000	2.620448000
N	5.296359000	6.376995000	5.680964000
N	6.042573000	8.397605000	3.800951000
N	5.369705000	5.425980000	3.481412000
N	3.428298000	5.612389000	4.525574000
C	8.388503000	7.477503000	7.119775000
C	4.852441000	8.893815000	3.754927000
C	8.776661000	8.484840000	6.269314000
C	4.759383000	5.870596000	4.599882000
C	6.431385000	7.062147000	5.784899000
C	7.984045000	8.801961000	5.160226000
C	6.797497000	8.144583000	4.912205000
C	2.809459000	9.920858000	4.186335000
H	2.514911000	10.935051000	4.454139000

C	3.229261000	4.455122000	11.750616000
H	3.146925000	3.927418000	10.801521000
H	2.299449000	4.359880000	12.304983000
C	3.641926000	5.907840000	11.553969000
H	3.195043000	6.330872000	10.654236000
H	3.344336000	6.521445000	12.411704000
C	5.789529000	7.094024000	10.970932000
H	5.733756000	7.889960000	11.719684000
H	5.334119000	7.446304000	10.046234000
H	6.832276000	6.848956000	10.792068000
C	6.364581000	1.673700000	10.405995000
C	4.782503000	-0.349850000	10.183210000
H	4.804362000	-1.430647000	10.071648000
H	3.824703000	-0.045699000	10.602754000
C	5.084063000	0.377959000	8.881215000
H	5.800447000	-0.185907000	8.271462000
H	4.178082000	0.526811000	8.292653000
C	5.834822000	2.691442000	8.247087000
H	4.913155000	2.812952000	7.678725000
H	6.643142000	2.424516000	7.558700000
H	6.091969000	3.625892000	8.738200000
C	8.275527000	5.242538000	14.645367000
C	6.927922000	5.209453000	15.322081000
C	6.508594000	4.085048000	16.028794000
C	6.092112000	6.320172000	15.250263000
C	4.853935000	6.310759000	15.879478000
C	4.438173000	5.184334000	16.580339000
C	5.267795000	4.071792000	16.653459000
H	7.148203000	3.213515000	16.097259000
H	6.411070000	7.193624000	14.694405000
H	4.215384000	7.183011000	15.824591000
H	4.952213000	3.194400000	17.202448000
H	3.474150000	5.175239000	17.071581000
H	9.082244000	5.305068000	15.366612000
H	8.341342000	6.093213000	13.972272000

H	2.073551000	9.226667000	4.608435000
C	2.972055000	9.726823000	2.680666000
H	2.080020000	9.323883000	2.202776000
H	3.237748000	10.668381000	2.184109000
C	4.452650000	9.749230000	6.085339000
H	4.098070000	8.890769000	6.664572000
H	3.967137000	10.648226000	6.463566000
H	5.522172000	9.860962000	6.236370000
C	6.793583000	5.249277000	3.294977000
H	7.200446000	6.008964000	2.625883000
H	6.979050000	4.258715000	2.878297000
H	7.310279000	5.301642000	4.248238000
C	4.416833000	4.759605000	2.596511000
H	4.564837000	3.675975000	2.641201000
H	4.547473000	5.083622000	1.564364000
C	4.668541000	8.452282000	1.340048000
H	5.087144000	9.337217000	0.846849000
H	3.906008000	8.020641000	0.691271000
H	5.467351000	7.732344000	1.493336000
C	2.444371000	6.177676000	5.416961000
C	3.064923000	5.181712000	3.181355000
H	2.627644000	6.018717000	2.626308000
H	2.342182000	4.366957000	3.209318000
H	1.682168000	5.432889000	5.648405000
H	1.952973000	7.047056000	4.967752000
H	2.935539000	6.481649000	6.337453000
Cl	9.309729000	7.085784000	8.512160000
Cl	10.214107000	9.391953000	6.554984000
H	8.292446000	9.599420000	4.499140000
C	6.886901000	5.539986000	7.622861000
C	7.209456000	4.297134000	6.831453000
H	5.823244000	5.597234000	7.831169000
H	7.431809000	5.573074000	8.558010000
C	8.522381000	4.023933000	6.447152000
C	8.812462000	2.897443000	5.688371000
C	7.793402000	2.028671000	5.312198000
C	6.485004000	2.288822000	5.703074000
C	6.195332000	3.419980000	6.456573000
H	8.020734000	1.147783000	4.726262000
H	5.689809000	1.609405000	5.424406000
H	5.174086000	3.629762000	6.747667000
H	9.324554000	4.689529000	6.740478000
H	9.834773000	2.692965000	5.398802000

[L5b+Bn]$^+$
E [Eh]: −2765.431135829715

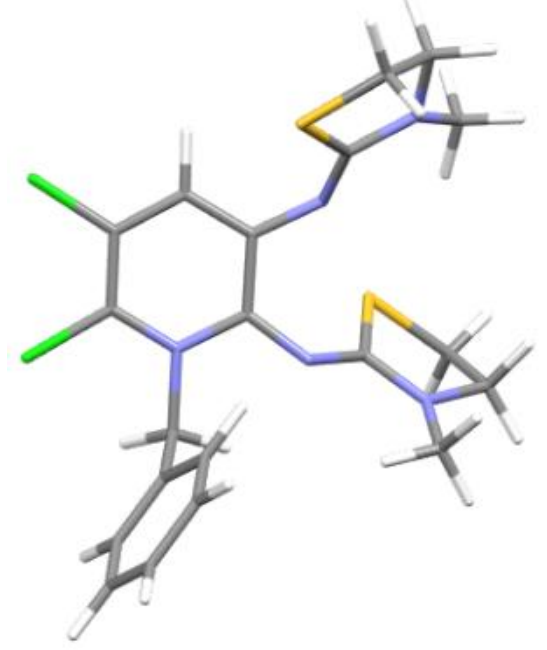

Cl	9.332383000	1.276785000	14.032616000
S	6.707958000	6.650110000	10.372086000
N	5.324551000	5.094871000	12.164107000
C	6.324185000	4.271047000	12.508024000
Cl	7.852402000	3.256593000	16.006771000
S	6.221620000	0.760433000	10.032641000
N	4.179978000	6.658847000	10.920314000
C	6.952909000	3.366013000	11.598270000
N	6.627360000	3.474047000	10.272546000
C	7.886684000	2.475600000	12.102327000
H	8.405116000	1.804952000	11.432341000
N	6.135669000	2.589094000	8.192334000
C	8.179851000	2.420957000	13.463293000
N	6.646949000	4.197396000	13.839496000
C	7.541904000	3.283396000	14.328426000
C	5.310200000	6.007810000	11.248243000

[L6+Bn]$^+$
E [Eh]: −2158.419352454044

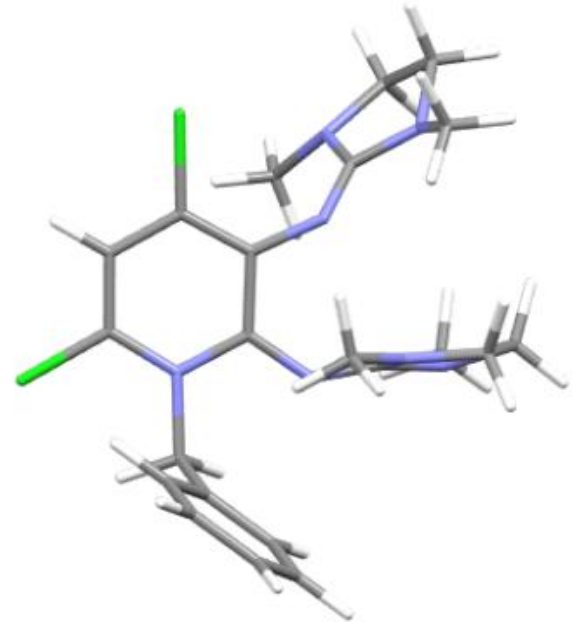

N	7.087691000	6.528838000	6.984201000
N	4.251887000	9.984796000	4.563910000
N	4.084174000	9.052234000	2.544134000
N	5.250754000	5.858570000	5.774863000
N	5.536108000	8.012718000	4.026814000
N	5.243855000	5.048280000	3.541333000
N	3.308995000	5.490288000	4.510524000
C	8.070593000	7.439805000	7.274808000
C	4.708442000	8.971101000	3.765459000
C	8.250754000	8.551426000	6.512389000
C	4.653968000	5.537134000	4.640700000
C	6.227218000	6.728753000	5.925037000
C	7.400038000	8.773757000	5.416283000
C	6.368198000	7.907525000	5.104395000
C	3.146157000	10.678280000	3.899368000
H	3.184360000	11.749082000	4.092704000

C	5.731815000	8.094478000	9.818904000
H	5.872963000	8.913845000	10.522014000
H	6.072223000	8.399464000	8.832843000
C	4.293010000	7.595849000	9.809757000
H	3.586886000	8.414374000	9.950262000
H	4.053174000	7.092740000	8.865815000
C	2.873631000	6.237475000	11.396386000
H	2.388436000	5.574605000	10.673945000
H	2.246461000	7.114723000	11.549820000
H	2.994195000	5.705613000	12.336057000
C	6.370536000	2.462602000	9.517864000
C	5.542522000	0.289186000	8.402574000
H	5.882589000	-0.711769000	8.150627000
H	4.455358000	0.301623000	8.458852000
C	6.071409000	1.343340000	7.441283000
H	7.066423000	1.074293000	7.066982000
H	5.406536000	1.465678000	6.585570000
C	6.396378000	3.833212000	7.491138000
H	6.290577000	4.659289000	8.188251000
H	5.684724000	3.941603000	6.672757000
H	7.411489000	3.848858000	7.081030000
C	5.969316000	5.130320000	14.780485000
C	4.845340000	4.479029000	15.545838000
H	5.602651000	5.948811000	14.170351000
H	6.725430000	5.517684000	15.456993000
C	3.971751000	3.581681000	14.935058000
C	4.655299000	4.813463000	16.884084000
C	3.599972000	4.266406000	17.602650000
C	2.732860000	3.368882000	16.991074000
C	2.922974000	3.026065000	15.657236000
H	4.104990000	3.312518000	13.895418000
H	5.339861000	5.498077000	17.370570000
H	3.463147000	4.531839000	18.642632000
H	1.916554000	2.934130000	17.552535000
H	2.253471000	2.324074000	15.177468000

H	2.182580000	10.291849000	4.253074000
C	3.382860000	10.326973000	2.434286000
H	2.459890000	10.225376000	1.864915000
H	4.017402000	11.073759000	1.941490000
C	4.276031000	9.934591000	6.014639000
H	3.792454000	9.031240000	6.400608000
H	3.738178000	10.801521000	6.392394000
H	5.292525000	9.982351000	6.396459000
C	6.664592000	4.894922000	3.333607000
H	7.072070000	5.741292000	2.774728000
H	6.848774000	3.974912000	2.779254000
H	7.172881000	4.816217000	4.290411000
C	4.259773000	4.659781000	2.535519000
H	4.254810000	3.572979000	2.417113000
H	4.491241000	5.108302000	1.568861000
C	4.666070000	8.455255000	1.360960000
H	5.397127000	9.121905000	0.889266000
H	3.879187000	8.233863000	0.639469000
H	5.168943000	7.534963000	1.644299000
C	2.402970000	6.210841000	5.374644000
C	2.943865000	5.190914000	3.127754000
H	2.615486000	6.107408000	2.626940000
H	2.135790000	4.461678000	3.083042000
H	1.473237000	5.652281000	5.481507000
H	2.173193000	7.201928000	4.966683000
H	2.863163000	6.323071000	6.352818000
Cl	9.097461000	7.160452000	8.629598000
H	9.033866000	9.251407000	6.754023000
Cl	7.655498000	10.191536000	4.463824000
C	6.945136000	5.244103000	7.715154000
C	7.449282000	4.083636000	6.893258000
H	5.889994000	5.120490000	7.941316000
H	7.492359000	5.338248000	8.645947000
C	8.763348000	4.062196000	6.426774000
C	9.218761000	2.998727000	5.657824000
C	8.365963000	1.942757000	5.353643000
C	7.057735000	1.954736000	5.823181000
C	6.601564000	3.023568000	6.585270000
H	8.722816000	1.111514000	4.759794000
H	6.391559000	1.132663000	5.595718000
H	5.577082000	3.042057000	6.934664000
H	9.437656000	4.876180000	6.662235000
H	10.241254000	2.990342000	5.303732000

[L7+Bn]$^+$
E [Eh]: −1698.862177786592

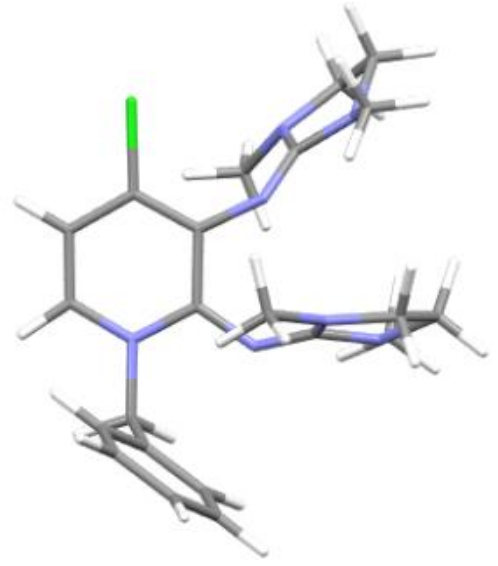

N	7.091915000	6.527578000	6.968545000
N	4.266771000	10.000521000	4.576449000
N	4.102851000	9.078280000	2.551841000
N	5.239247000	5.854062000	5.786207000
N	5.535321000	8.020176000	4.041047000
N	5.237531000	5.062257000	3.543507000
N	3.299698000	5.439386000	4.535413000
C	8.075321000	7.422656000	7.271140000
C	4.719620000	8.987268000	3.775619000
C	8.259669000	8.539975000	6.522418000
C	4.645997000	5.522212000	4.655879000
C	6.226181000	6.719638000	5.926400000
C	7.405474000	8.773138000	5.428972000
C	6.370485000	7.906801000	5.113709000
C	3.170624000	10.706135000	3.909054000
H	3.216754000	11.775865000	4.106599000
H	2.202012000	10.326091000	4.255775000

[L8+Bn]$^+$
E [Eh]: −2158.419797356556

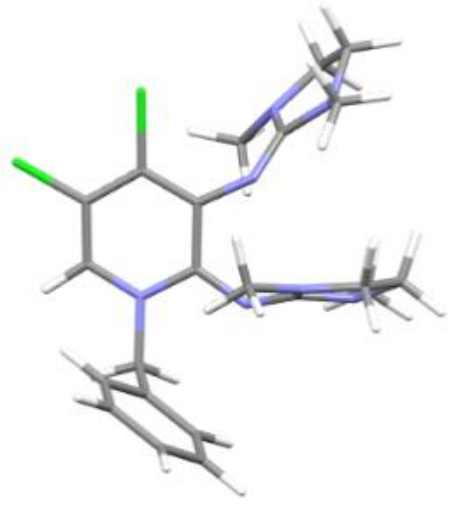

N	7.096439000	6.525302000	6.932084000
N	4.271954000	9.987442000	4.575198000
N	4.058486000	9.082764000	2.551432000
N	5.221117000	5.827441000	5.800321000
N	5.465746000	7.972235000	4.033505000
N	5.180416000	5.010894000	3.566756000
N	3.260117000	5.414166000	4.582483000
C	8.083683000	7.424916000	7.203390000
C	4.681523000	8.968817000	3.768121000
C	8.254861000	8.531109000	6.429547000
C	4.608111000	5.486895000	4.681989000
C	6.209691000	6.695629000	5.908805000
C	7.378258000	8.752762000	5.339214000
C	6.334921000	7.873187000	5.078548000
C	3.239509000	10.782860000	3.910084000
H	3.386804000	11.847436000	4.086242000
H	2.246450000	10.501806000	4.278891000

C	3.411916000	10.358543000	2.443474000	C	3.427109000	10.394689000	2.444123000
H	2.490625000	10.266023000	1.869754000	H	2.487474000	10.340523000	1.895741000
H	4.054072000	11.102484000	1.956206000	H	4.091909000	11.096586000	1.926469000
C	4.282848000	9.936711000	6.026780000	C	4.358672000	9.958559000	6.023554000
H	3.810132000	9.022235000	6.399853000	H	4.238402000	8.942156000	6.402306000
H	3.730465000	10.791975000	6.410072000	H	3.555109000	10.570189000	6.430422000
H	5.296641000	9.994198000	6.415142000	H	5.307433000	10.360915000	6.380627000
C	6.658472000	4.920539000	3.331237000	C	6.597295000	4.863550000	3.332415000
H	7.060746000	5.775949000	2.782566000	H	6.992034000	5.710478000	2.765217000
H	6.847126000	4.008428000	2.765152000	H	6.775519000	3.943847000	2.775282000
H	7.169703000	4.832928000	4.285837000	H	7.124293000	4.787607000	4.279404000
C	4.253993000	4.650417000	2.547026000	C	4.180116000	4.608253000	2.583250000
H	4.268159000	3.562805000	2.433782000	H	4.185091000	3.521014000	2.467171000
H	4.468520000	5.098635000	1.576354000	H	4.384945000	5.056802000	1.610485000
C	4.681196000	8.475607000	1.370211000	C	4.598485000	8.454256000	1.364971000
H	5.426774000	9.130583000	0.904831000	H	5.357276000	9.082360000	0.884156000
H	3.894635000	8.270124000	0.643730000	H	3.794743000	8.269981000	0.651773000
H	5.165845000	7.545458000	1.653605000	H	5.057268000	7.510498000	1.646539000
C	2.386863000	6.141337000	5.408252000	C	2.368104000	6.137541000	5.459898000
C	2.934359000	5.157777000	3.149112000	C	2.872070000	5.124558000	3.203848000
H	2.596117000	6.078662000	2.662291000	H	2.529124000	6.043434000	2.716520000
H	2.133710000	4.421207000	3.093672000	H	2.067738000	4.390939000	3.165792000
H	1.461934000	5.573359000	5.506120000	H	1.430274000	5.591674000	5.559302000
H	2.148618000	7.136343000	5.014314000	H	2.153356000	7.138977000	5.068754000
H	2.845241000	6.244385000	6.388242000	H	2.831465000	6.227440000	6.438783000
H	8.691316000	7.180253000	8.122283000	H	8.720273000	7.208809000	8.045301000
H	9.046339000	9.237474000	6.763709000	Cl	9.532965000	9.626409000	6.805070000
Cl	7.655966000	10.195362000	4.478936000	Cl	7.597294000	10.130905000	4.331711000
C	7.002909000	5.258959000	7.724301000	C	7.033365000	5.267486000	7.711642000
C	7.477381000	4.087163000	6.899921000	C	7.503260000	4.089216000	6.894420000
H	5.968499000	5.117406000	8.027658000	H	6.005449000	5.123520000	8.034949000
H	7.612012000	5.388948000	8.617267000	H	7.657712000	5.417862000	8.590523000
C	8.778511000	4.059221000	6.399187000	C	8.797649000	4.064017000	6.376152000
C	9.210608000	2.993191000	5.620892000	C	9.225241000	2.992148000	5.603458000
C	8.344882000	1.941061000	5.339749000	C	8.362108000	1.931827000	5.346053000
C	7.048857000	1.960133000	5.841647000	C	7.073075000	1.948233000	5.865869000
C	6.617138000	3.031117000	6.615251000	C	6.645464000	3.024956000	6.633698000
H	8.682509000	1.107390000	4.738113000	H	8.696635000	1.093862000	4.748678000
H	6.373291000	1.141080000	5.631608000	H	6.399925000	1.122685000	5.674433000
H	5.602130000	3.054792000	6.990689000	H	5.635834000	3.046592000	7.023441000
H	9.459683000	4.875151000	6.610154000	H	9.477152000	4.885685000	6.569688000
H	10.223383000	2.979946000	5.240096000	H	10.232839000	2.980704000	5.209199000

[L9+Bn]+
E [Eh]: −1443.784945518649

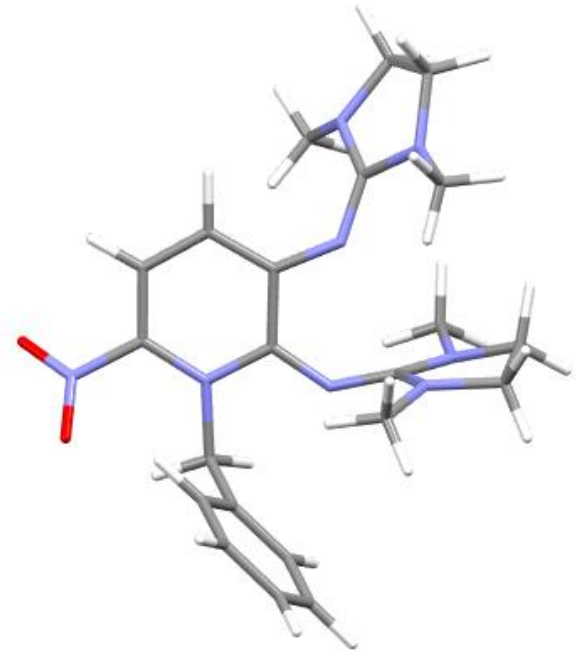

[L10+Bn]+
E [Eh]: −1443.799222756847

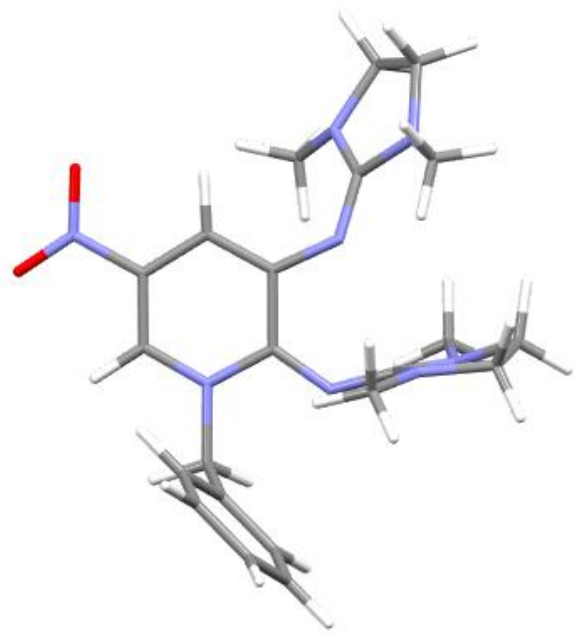

N	7.266700000	6.461981000	6.952845000	N	7.175650000	6.380089000	6.935213000
N	4.368268000	10.220865000	4.468522000	N	4.561140000	10.193968000	4.438617000
N	4.298821000	9.107216000	2.539235000	N	4.249264000	9.098812000	2.523921000
N	5.515227000	5.661520000	5.717745000	N	5.407640000	5.576035000	5.727136000
N	5.344357000	8.013158000	4.296621000	N	5.314648000	7.914291000	4.220898000
N	5.101247000	4.950309000	3.503936000	N	4.972898000	4.856475000	3.519186000
N	3.370013000	5.539659000	4.745336000	N	3.261364000	5.529989000	4.741714000
C	8.080959000	7.501038000	7.345851000	C	8.077588000	7.336632000	7.273904000
C	4.734696000	9.065361000	3.828825000	C	4.773041000	9.015033000	3.784936000
C	7.995534000	8.732933000	6.774261000	C	8.087481000	8.516249000	6.597961000
C	4.716018000	5.463076000	4.683535000	C	4.601644000	5.397948000	4.686083000
C	6.326057000	6.651722000	5.968369000	C	6.241078000	6.556437000	5.937893000
C	7.078026000	8.959702000	5.743422000	C	7.174453000	8.771780000	5.562681000
C	6.233448000	7.954395000	5.313430000	C	6.230924000	7.826482000	5.227128000
C	3.497795000	11.012064000	3.590674000	C	3.767945000	11.103283000	3.607359000
H	3.720607000	12.074606000	3.672538000	H	4.154506000	12.120467000	3.652876000
H	2.449765000	10.853074000	3.869108000	H	2.728876000	11.110704000	3.953446000

C	3.800913000	10.437873000	2.208825000
H	2.922636000	10.385689000	1.566544000
H	4.575996000	11.013300000	1.690181000
C	4.086029000	10.284023000	5.892652000
H	3.071936000	9.927774000	6.106382000
H	4.166102000	11.318765000	6.223880000
H	4.795277000	9.688828000	6.456230000
C	6.433579000	4.482726000	3.188026000
H	6.795764000	4.959675000	2.275629000
H	6.433047000	3.399667000	3.047288000
H	7.105469000	4.716807000	4.007400000
C	3.941040000	4.562197000	2.704984000
H	3.824175000	3.474128000	2.721042000
H	4.052222000	4.880921000	1.668932000
C	4.836122000	8.248903000	1.504905000
H	5.664632000	8.732083000	0.976097000
H	4.054044000	8.012810000	0.782551000
H	5.197540000	7.334496000	1.967118000
C	2.664281000	6.333808000	5.723661000
C	2.792583000	5.277207000	3.428891000
H	2.533492000	6.224405000	2.943159000
H	1.893584000	4.667829000	3.506791000
H	1.703917000	5.870468000	5.947543000
H	2.490876000	7.350980000	5.355976000
H	3.254942000	6.381223000	6.635047000
N	9.157792000	7.281904000	8.310394000
H	7.033636000	9.932204000	5.273206000
C	7.244148000	5.126433000	7.610644000
C	7.739144000	4.026058000	6.702860000
H	6.207495000	4.938490000	7.881417000
H	7.837627000	5.184187000	8.513702000
C	8.928843000	4.152256000	5.989064000
C	9.364933000	3.121480000	5.164855000
C	8.622362000	1.951216000	5.054985000
C	7.439212000	1.816669000	5.773234000
C	6.998699000	2.852510000	6.586710000
H	8.968602000	1.145154000	4.421321000
H	6.858787000	0.906056000	5.699178000
H	6.067320000	2.752820000	7.130739000
H	9.523678000	5.049258000	6.088085000
H	10.293984000	3.227822000	4.620016000
O	9.450755000	8.228740000	9.019800000
O	9.724541000	6.196195000	8.311320000
H	8.666795000	9.505692000	7.113504000

C	3.895533000	10.481333000	2.216790000
H	2.972435000	10.538716000	1.641103000
H	4.695550000	10.953953000	1.635383000
C	4.501768000	10.322255000	5.884445000
H	4.414930000	9.342844000	6.350199000
H	3.622352000	10.909620000	6.150656000
H	5.386297000	10.821246000	6.284309000
C	6.302367000	4.401356000	3.179679000
H	6.672953000	4.933248000	2.301214000
H	6.292357000	3.330152000	2.968802000
H	6.972062000	4.579486000	4.014959000
C	3.808062000	4.541003000	2.695565000
H	3.643319000	3.459544000	2.680257000
H	3.949607000	4.881421000	1.670013000
C	4.674357000	8.216958000	1.457448000
H	5.498281000	8.654873000	0.882904000
H	3.841168000	8.027958000	0.779511000
H	5.010378000	7.281371000	1.895433000
C	2.587161000	6.368176000	5.706804000
C	2.681064000	5.290224000	3.421165000
H	2.452642000	6.247148000	2.940243000
H	1.763402000	4.708786000	3.494600000
H	1.598294000	5.961507000	5.915555000
H	2.480653000	7.391556000	5.331680000
H	3.164049000	6.383836000	6.628070000
N	9.066001000	9.534914000	6.976789000
H	7.225597000	9.716482000	5.043844000
C	7.244492000	5.059300000	7.596375000
C	7.779013000	3.990583000	6.673586000
H	6.245716000	4.798836000	7.938933000
H	7.886052000	5.186006000	8.467158000
C	8.962567000	4.191136000	5.965449000
C	9.452920000	3.202109000	5.121601000
C	8.766476000	2.000180000	4.984951000
C	7.589609000	1.792427000	5.695289000
C	7.096020000	2.786823000	6.530761000
H	9.151871000	1.226631000	4.333797000
H	7.054125000	0.856996000	5.597623000
H	6.170196000	2.630518000	7.070383000
H	9.507804000	5.121925000	6.067604000
H	10.374704000	3.366150000	4.579090000
O	9.875316000	9.251167000	7.845783000
O	9.002597000	10.606354000	6.394017000
H	8.767630000	7.119126000	8.071806000

$[L11+Bn]^+$
E [Eh]: −1443.800106107596

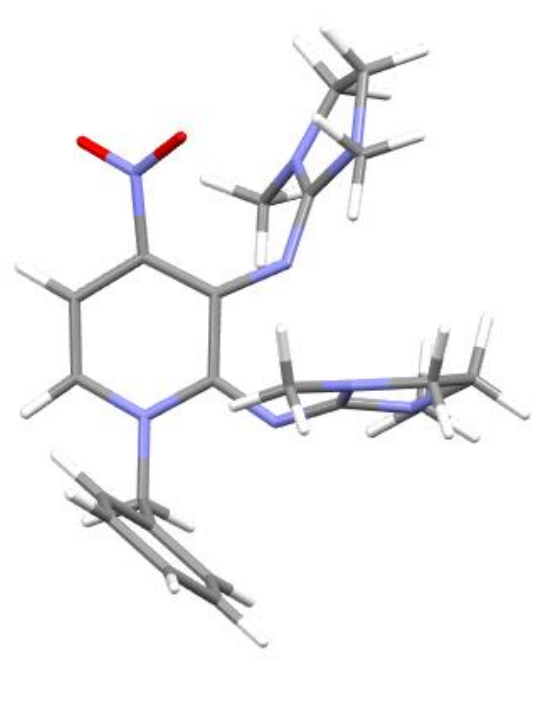

N	7.138232000	6.377198000	6.936332000
N	4.386368000	10.129529000	4.495539000
N	4.326800000	9.121049000	2.525052000
N	5.284132000	5.614426000	5.835354000
N	5.186101000	7.887569000	4.315163000
N	5.008182000	4.896276000	3.589371000
N	3.201372000	5.311856000	4.793427000
C	8.095643000	7.326325000	7.212626000
C	4.697177000	9.005012000	3.820435000
C	8.175945000	8.456179000	6.478529000
C	4.550713000	5.348141000	4.768237000
C	6.196462000	6.544703000	5.971266000
C	7.257593000	8.665821000	5.425957000
C	6.197664000	7.786993000	5.180758000

$[L12+Bn]^+$
E [Eh]: −1648.276756406678

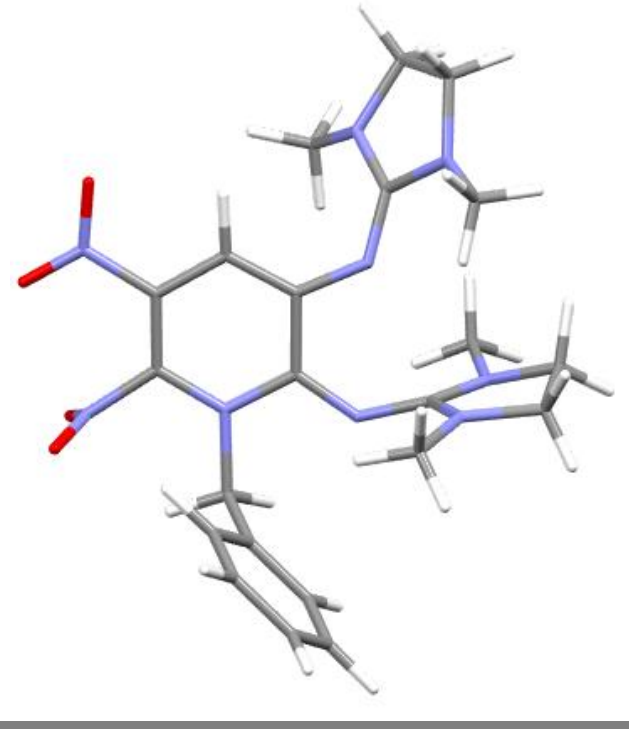

N	7.219766000	6.441458000	6.942165000
N	4.569548000	10.213428000	4.360979000
N	4.269036000	9.060795000	2.481038000
N	5.464590000	5.602928000	5.743681000
N	5.304778000	7.921712000	4.226847000
N	4.941184000	4.845982000	3.569833000
N	3.282308000	5.560898000	4.840316000
C	8.086560000	7.440308000	7.265356000
C	4.777482000	9.015046000	3.746683000
C	8.084176000	8.617667000	6.581046000
C	4.615953000	5.415289000	4.734988000
C	6.269076000	6.600534000	5.946347000
C	7.164326000	8.819575000	5.537108000
C	6.230980000	7.862476000	5.220084000

C	3.741980000	11.107857000	3.619229000
H	4.194226000	12.092345000	3.733035000
H	2.678405000	11.182558000	3.865920000
C	3.975206000	10.509605000	2.225117000
H	3.093378000	10.564906000	1.588074000
H	4.811056000	10.995705000	1.714379000
C	4.347794000	10.271023000	5.935004000
H	3.390188000	10.703582000	6.231669000
H	5.149537000	10.920287000	6.292488000
H	4.446485000	9.295206000	6.404081000
C	6.383372000	4.584701000	3.274065000
H	6.739340000	5.204737000	2.448696000
H	6.476013000	3.533256000	2.994626000
H	7.009141000	4.756710000	4.143727000
C	3.905572000	4.461313000	2.735434000
H	3.871198000	3.368100000	2.698465000
H	4.023469000	4.836206000	1.718868000
C	4.874707000	8.271232000	1.488539000
H	5.798907000	8.692336000	1.081607000
H	4.146621000	8.162014000	0.684675000
H	5.092002000	7.297600000	1.919317000
C	2.416332000	6.046325000	5.760375000
C	2.685294000	5.056584000	3.450148000
H	2.369124000	5.999445000	2.990597000
H	1.834194000	4.377939000	3.475878000
H	1.467254000	5.536354000	5.921471000
H	2.219265000	7.067331000	5.415307000
H	2.961208000	6.087089000	6.699957000
N	7.482851000	9.845811000	4.603472000
C	7.179896000	5.082559000	7.646622000
C	7.668636000	3.970013000	6.750127000
H	6.180379000	4.861957000	8.014543000
H	7.840370000	5.221105000	8.501028000
C	8.884793000	4.088647000	6.078492000
C	9.330578000	3.070622000	5.245368000
C	8.565717000	1.920258000	5.081389000
C	7.355380000	1.794062000	5.753307000
C	6.907579000	2.817765000	6.579873000
H	8.915834000	1.123515000	4.438239000
H	6.758697000	0.899041000	5.633598000
H	5.956248000	2.726847000	7.088897000
H	9.486406000	4.981573000	6.199334000
H	10.277635000	3.171065000	4.731571000
H	8.769222000	7.094209000	8.020778000
H	8.937565000	9.192816000	6.676219000
O	7.128654000	9.811406000	3.426699000
O	8.049528000	10.797178000	5.119627000

C	3.794165000	11.105199000	3.493095000
H	4.196320000	12.117132000	3.505992000
H	2.754423000	11.139966000	3.834457000
C	3.918683000	10.432971000	2.125068000
H	2.994967000	10.471922000	1.548908000
H	4.719135000	10.880711000	1.525556000
C	4.504084000	10.391280000	5.801397000
H	4.424269000	9.427742000	6.299829000
H	3.618729000	10.979668000	6.044509000
H	5.382647000	10.912218000	6.186119000
C	6.255067000	4.375682000	3.188634000
H	6.587485000	4.879520000	2.279064000
H	6.233459000	3.298980000	3.010943000
H	6.960217000	4.576507000	3.988853000
C	3.743018000	4.521461000	2.797296000
H	3.573347000	3.440993000	2.809289000
H	3.847767000	4.841710000	1.761045000
C	4.678672000	8.132952000	1.447961000
H	5.501819000	8.540291000	0.850894000
H	3.838775000	7.925539000	0.783758000
H	5.008511000	7.211966000	1.920240000
C	2.646088000	6.409512000	5.822154000
C	2.647602000	5.291295000	3.550172000
H	2.400674000	6.236782000	3.056392000
H	1.733123000	4.714108000	3.676550000
H	1.661007000	6.011586000	6.062492000
H	2.536384000	7.431921000	5.445945000
H	3.249454000	6.424138000	6.726420000
N	8.998201000	7.140750000	8.394249000
N	8.998267000	9.710072000	6.926302000
H	7.206599000	9.756679000	5.004693000
C	7.290722000	5.097360000	7.581169000
C	7.817465000	4.046315000	6.634556000
H	6.283151000	4.847144000	7.902320000
H	7.910192000	5.175546000	8.468437000
C	8.993822000	4.250691000	5.916248000
C	9.469976000	3.264984000	5.059766000
C	8.781183000	2.064532000	4.924507000
C	7.613202000	1.852953000	5.648750000
C	7.130343000	2.844036000	6.493638000
H	9.158650000	1.293708000	4.265493000
H	7.077077000	0.917504000	5.554795000
H	6.211379000	2.685109000	7.044289000
H	9.550892000	5.171604000	6.032190000
H	10.387209000	3.429868000	4.509905000
O	9.743323000	9.547770000	7.876093000
O	8.939091000	10.712736000	6.233967000
O	8.558021000	7.356228000	9.502961000
O	10.065039000	6.643313000	8.103915000

[L13+Bn]$^+$
E [Eh]: −1317.910739514950

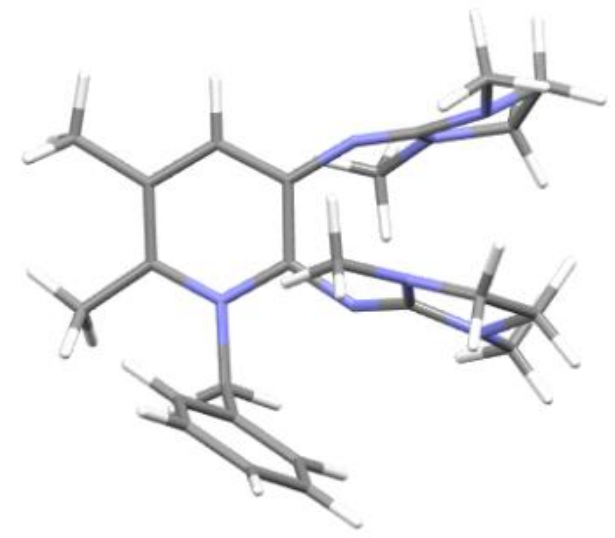

N	7.318361000	6.819324000	6.754874000
N	4.164358000	9.603622000	4.625735000
N	4.056560000	8.739546000	2.580055000
N	5.328776000	6.421798000	5.647117000
N	6.058521000	8.406368000	3.712017000
N	5.348929000	5.433192000	3.457824000
N	3.447866000	5.565451000	4.585085000
C	8.473187000	7.524352000	7.016777000
C	4.860003000	8.878610000	3.693427000
C	8.853124000	8.523501000	6.142301000
C	4.775837000	5.872807000	4.601390000
C	6.482894000	7.098979000	5.706741000

[L14+Bn]$^+$
E [Eh]: −1468.323232904069

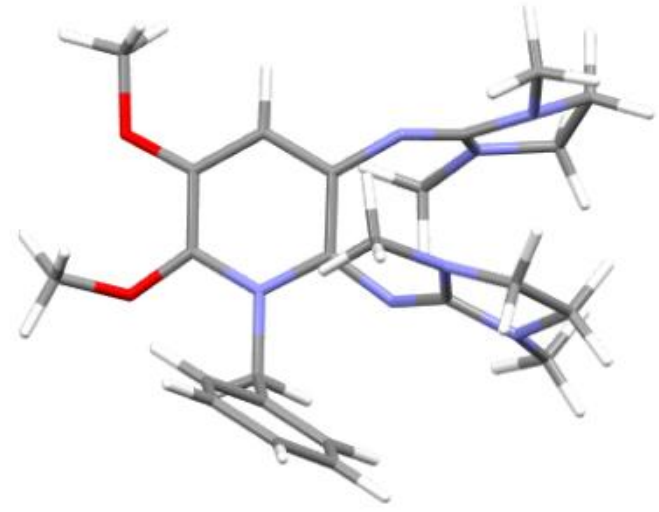

N	7.198677000	6.745191000	6.899756000
N	4.141808000	9.615041000	4.780410000
N	4.142196000	8.875095000	2.684050000
N	5.243225000	6.424594000	5.696770000
N	6.093125000	8.493699000	3.885987000
N	5.434942000	5.506603000	3.483064000
N	3.464487000	5.525873000	4.500003000
C	8.342919000	7.416673000	7.231984000
C	4.889416000	8.953719000	3.840730000
C	8.754056000	8.485742000	6.444411000
C	4.779140000	5.883173000	4.607407000
C	6.407928000	7.086825000	5.832107000

C	8.030127000	8.810431000	5.044213000
C	6.833165000	8.160857000	4.818007000
C	2.805658000	9.867054000	4.168404000
H	2.494153000	10.876164000	4.437195000
H	2.095454000	9.159245000	4.612040000
C	2.935273000	9.666677000	2.660293000
H	2.039383000	9.243869000	2.207043000
H	3.169820000	10.610704000	2.152066000
C	4.502703000	9.761305000	6.022144000
H	4.161967000	8.916953000	6.629960000
H	4.030218000	10.670898000	6.392707000
H	5.576470000	9.869697000	6.141009000
C	6.766526000	5.267880000	3.221791000
H	7.137101000	6.009533000	2.512803000
H	6.949913000	4.265482000	2.832036000
H	7.318176000	5.361354000	4.151186000
C	4.379365000	4.726987000	2.625473000
H	4.552560000	3.647396000	2.686525000
H	4.461728000	5.031527000	1.582267000
C	4.617671000	8.408311000	1.289089000
H	5.016383000	9.292651000	0.777904000
H	3.843853000	7.964077000	0.662322000
H	5.426684000	7.697137000	1.429363000
C	2.485639000	6.139576000	5.494187000
C	3.043664000	5.131119000	3.254628000
H	2.578691000	5.962698000	2.712748000
H	2.331991000	4.307721000	3.305927000
H	1.723351000	5.399683000	5.740289000
H	1.990968000	7.012967000	5.055125000
H	2.996585000	6.441458000	6.404436000
C	9.262258000	7.189429000	8.247048000
C	10.115312000	9.322833000	6.342582000
H	8.317798000	9.591638000	4.352180000
C	6.965263000	5.613164000	7.546870000
C	7.224932000	4.349062000	6.761828000
H	5.912155000	5.686052000	7.802203000
H	7.541807000	5.630887000	8.463097000
C	8.500733000	4.061831000	6.277419000
C	8.730973000	2.912671000	5.532107000
C	7.685368000	2.036078000	5.263567000
C	6.412187000	2.312869000	5.748052000
C	6.184548000	3.464529000	6.491538000
H	7.864381000	1.138777000	4.685829000
H	5.595468000	1.630907000	5.549404000
H	5.188826000	3.686832000	6.853204000
H	9.321257000	4.742173000	6.467578000
H	9.726229000	2.699916000	5.164550000
H	10.119868000	7.848618000	8.326771000
H	9.638212000	6.164700000	8.242087000
H	8.666696000	7.318171000	9.153832000
H	10.250067000	10.023181000	5.520282000
H	10.999592000	8.684428000	6.380015000
H	10.087413000	9.902388000	7.267504000

C	7.990789000	8.834519000	5.331039000
C	6.805981000	8.181711000	5.019655000
C	2.800437000	9.883090000	4.275578000
H	2.455700000	10.866877000	4.592926000
H	2.085257000	9.134551000	4.636345000
C	3.005904000	9.782958000	2.766073000
H	2.139232000	9.380768000	2.242704000
H	3.252174000	10.760407000	2.331489000
C	4.409361000	9.685737000	6.200126000
H	4.096600000	8.779279000	6.727071000
H	3.864660000	10.535636000	6.610394000
H	5.468062000	9.849784000	6.379086000
C	6.871900000	5.379144000	3.346170000
H	7.301641000	6.252717000	2.854124000
H	7.088104000	4.489274000	2.755441000
H	7.340239000	5.246428000	4.317733000
C	4.540700000	4.795720000	2.573132000
H	4.737993000	3.719422000	2.619174000
H	4.678201000	5.129534000	1.544860000
C	4.764867000	8.616420000	1.405185000
H	5.186286000	9.527344000	0.963462000
H	4.022375000	8.208102000	0.719003000
H	5.567092000	7.897148000	1.544115000
C	2.430919000	6.062591000	5.352599000
C	3.158858000	5.147190000	3.126806000
H	2.716120000	5.995730000	2.591495000
H	2.461154000	4.311197000	3.091358000
H	1.665982000	5.305551000	5.527999000
H	1.952997000	6.938898000	4.899845000
H	2.872320000	6.350547000	6.302739000
O	8.950888000	7.001728000	8.349635000
O	9.869926000	9.138283000	6.852639000
H	8.286501000	9.659481000	4.700247000
C	6.811005000	5.526581000	7.665112000
C	7.111199000	4.277520000	6.874464000
H	5.747174000	5.606183000	7.865413000
H	7.357574000	5.550359000	8.599792000
C	8.425627000	3.954822000	6.538498000
C	8.703979000	2.827660000	5.776575000
C	7.667859000	2.004749000	5.347560000
C	6.356521000	2.312206000	5.690568000
C	6.080648000	3.444414000	6.448016000
H	7.883655000	1.123657000	4.757590000
H	5.546758000	1.669481000	5.369888000
H	5.057204000	3.691667000	6.697870000
H	9.238297000	4.587990000	6.871071000
H	9.728318000	2.586521000	5.524251000
C	10.341209000	10.231824000	6.069214000
H	10.584732000	9.912402000	5.053023000
H	11.241263000	10.582112000	6.566169000
H	9.604989000	11.038630000	6.037726000
C	10.386723000	6.839213000	8.374226000
H	10.575921000	6.077005000	9.125809000
H	10.866002000	7.774309000	8.647715000
H	10.749782000	6.507472000	7.401577000

$[L15+Bn]^+$

E [Eh]: −1437.775086407294

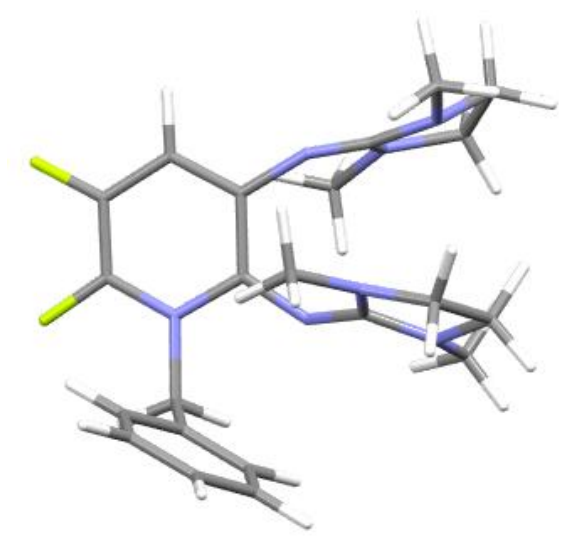

N	7.308852000	6.776342000	6.747716000
N	4.153373000	9.638948000	4.608144000
N	4.044627000	8.760609000	2.568056000
N	5.314268000	6.386921000	5.664050000
N	6.022274000	8.382549000	3.723392000
N	5.304646000	5.408934000	3.474691000

N	3.402259000	5.618403000	4.585248000
C	8.447149000	7.486536000	6.960785000
C	4.836158000	8.888582000	3.688775000
C	8.800609000	8.507273000	6.127654000
C	4.736704000	5.869667000	4.608961000
C	6.456916000	7.068746000	5.710399000
C	7.983110000	8.831236000	5.048279000
C	6.799877000	8.148309000	4.825135000
C	2.806241000	9.935600000	4.132747000
H	2.520195000	10.955023000	4.389583000
H	2.073166000	9.251108000	4.575494000
C	2.946408000	9.718012000	2.628269000
H	2.044845000	9.315065000	2.168681000
H	3.212607000	10.649961000	2.114123000
C	4.469563000	9.773997000	6.013169000
H	4.112462000	8.923583000	6.602647000
H	3.996041000	10.680623000	6.388356000
H	5.541293000	9.876829000	6.153500000
C	6.720528000	5.213690000	3.246750000
H	7.119927000	5.972681000	2.572545000
H	6.879127000	4.224366000	2.816630000
H	7.263405000	5.250242000	4.186308000
C	4.316502000	4.741401000	2.630048000
H	4.457865000	3.657113000	2.680049000
H	4.412708000	5.054539000	1.590814000
C	4.612204000	8.405901000	1.285158000
H	5.038091000	9.276452000	0.772958000
H	3.834218000	7.976844000	0.653272000
H	5.401423000	7.675556000	1.438769000
C	2.454746000	6.207762000	5.500786000
C	2.989005000	5.179053000	3.257983000
H	2.539317000	6.014725000	2.710859000
H	2.261575000	4.370237000	3.317864000
H	1.680956000	5.481099000	5.749957000
H	1.973795000	7.089728000	5.064634000
H	2.975479000	6.498455000	6.409202000
F	9.199169000	7.130742000	7.990622000
F	9.919882000	9.191363000	6.368701000
H	8.270314000	9.640890000	4.391336000
C	7.023704000	5.564952000	7.571413000
C	7.307407000	4.307842000	6.788250000
H	5.977065000	5.618847000	7.854919000
H	7.637818000	5.631494000	8.463365000
C	8.608345000	4.011074000	6.382486000
C	8.866168000	2.875033000	5.626420000
C	7.825588000	2.020706000	5.276293000
C	6.529265000	2.303957000	5.690419000
C	6.271618000	3.445009000	6.440527000
H	8.027522000	1.131585000	4.693502000
H	5.718582000	1.634406000	5.433407000
H	5.260123000	3.672648000	6.750999000
H	9.426745000	4.664727000	6.660242000
H	9.879338000	2.650654000	5.320269000

$[L2a+Me]^+$
E [Eh]: −1008.279973534884

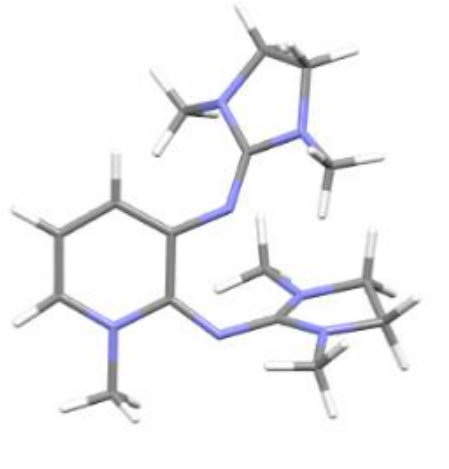

N	7.529750000	8.939879000	6.159651000
N	9.787379000	9.485420000	6.483180000
N	8.922728000	7.983322000	7.846836000
N	8.679728000	6.628089000	5.132175000
N	10.709663000	5.532511000	4.962346000
N	8.924251000	4.210138000	5.206709000
N	5.509344000	8.361633000	5.250259000
C	6.819166000	8.049071000	5.493769000
C	7.347190000	6.828255000	4.917219000

$[L2a+Aly]^+$
E [Eh]: −1085.659240552367

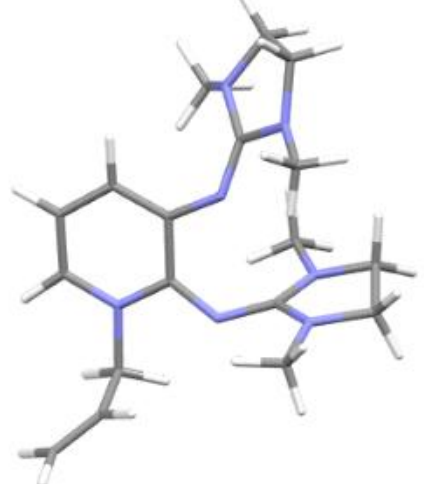

N	7.534374000	8.917518000	6.241529000
N	9.791758000	9.464408000	6.549151000
N	8.950622000	7.935630000	7.897875000
N	8.665348000	6.624924000	5.167327000
N	10.701018000	5.551592000	4.936861000
N	8.933175000	4.208413000	5.196605000
N	5.480899000	8.317288000	5.402889000
C	6.802261000	8.025101000	5.597778000
C	7.325851000	6.816830000	4.990017000

C	6.517178000	6.085081000	4.096798000
C	5.186164000	6.466971000	3.867422000
C	4.705366000	7.595313000	4.453878000
C	8.691258000	8.753276000	6.759158000
C	10.812472000	9.295777000	7.504438000
H	10.826693000	10.146733000	8.193661000
H	11.799248000	9.198213000	7.053555000
C	10.341239000	8.008088000	8.191230000
H	10.842871000	7.122914000	7.784544000
H	10.480894000	8.026541000	9.271053000
C	9.835093000	10.577412000	5.538799000
H	8.986338000	10.497555000	4.865435000
H	10.758514000	10.525831000	4.960361000
H	9.794893000	11.543664000	6.050650000
C	8.049738000	6.943759000	8.329322000
H	7.023574000	7.172760000	8.051981000
H	8.111622000	6.895376000	9.416695000
H	8.318639000	5.968094000	7.916007000
C	9.345045000	5.518563000	5.098563000
C	11.203544000	4.189625000	4.687484000
H	12.168824000	4.014670000	5.161365000
H	11.314913000	4.036274000	3.607244000
C	10.090475000	3.323158000	5.266653000
H	9.918320000	2.409373000	4.699606000
H	10.298270000	3.051655000	6.308518000
C	11.409037000	6.680094000	4.423766000
H	10.892696000	7.582898000	4.738063000
H	11.442367000	6.655480000	3.328563000
H	12.431127000	6.689264000	4.803043000
C	7.777728000	3.804957000	6.000567000
H	8.059253000	3.665408000	7.051129000
H	7.395016000	2.856709000	5.622371000
H	6.984400000	4.541008000	5.945629000
H	4.541673000	5.880543000	3.229996000
H	3.694298000	7.947301000	4.327467000
H	6.913062000	5.204823000	3.608746000
C	4.977593000	9.594159000	5.839632000
H	3.927285000	9.677938000	5.578455000
H	5.528731000	10.452470000	5.459663000
H	5.092287000	9.566892000	6.921344000

C	6.484074000	6.080541000	4.175167000
C	5.145000000	6.454104000	3.981551000
C	4.668666000	7.564649000	4.603879000
C	8.701141000	8.723070000	6.826352000
C	10.828570000	9.265133000	7.556289000
H	10.844779000	10.104206000	8.260009000
H	11.811262000	9.181741000	7.093741000
C	10.372766000	7.963167000	8.226479000
H	10.875422000	7.087716000	7.800455000
H	10.524343000	7.964916000	9.304927000
C	9.819330000	10.573128000	5.624021000
H	8.981273000	10.480084000	4.939053000
H	10.749910000	10.555413000	5.055296000
H	9.746796000	11.530287000	6.149494000
C	8.089690000	6.884603000	8.377155000
H	7.058632000	7.109588000	8.115312000
H	8.164846000	6.822446000	9.463033000
H	8.359658000	5.915981000	7.948167000
C	9.339462000	5.522639000	5.101798000
C	11.200036000	4.218549000	4.625957000
H	12.177604000	4.043858000	5.074132000
H	11.287656000	4.085603000	3.540809000
C	10.108464000	3.331713000	5.214960000
H	9.931902000	2.426114000	4.636167000
H	10.342738000	3.044137000	6.246860000
C	11.377386000	6.714859000	4.402613000
H	10.865337000	7.607633000	4.750771000
H	11.379266000	6.713369000	3.306582000
H	12.409882000	6.722618000	4.752565000
C	7.808737000	3.779060000	6.008897000
H	8.112908000	3.631221000	7.051994000
H	7.431282000	2.830056000	5.627268000
H	7.004426000	4.504691000	5.978087000
C	4.914389000	9.544467000	6.012097000
H	5.430521000	9.696288000	6.958006000
H	3.862478000	9.347799000	6.211381000
C	5.076266000	10.734259000	5.117242000
H	6.097062000	11.004416000	4.872020000
C	4.055575000	11.439375000	4.650912000
H	3.029144000	11.186350000	4.892520000
H	4.205988000	12.305347000	4.019922000
H	4.492286000	5.875484000	3.345316000
H	3.654997000	7.918083000	4.502794000
H	6.878726000	5.214183000	3.661835000

[L2a+Ph]$^{+}$
E [Eh]: −1199.984992816385

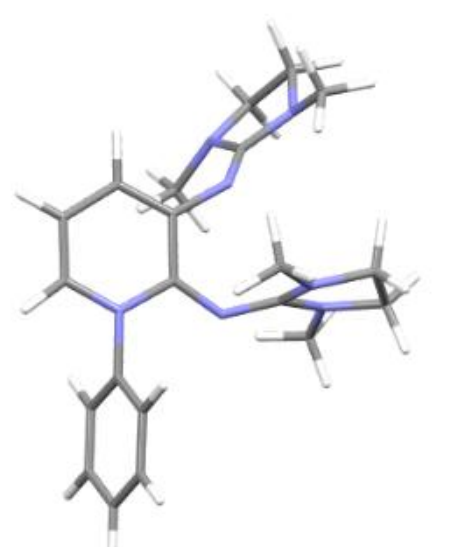

N	6.098437000	6.229969000	5.565885000
N	3.270782000	9.559183000	3.244410000
N	2.568474000	8.478863000	1.415327000
N	3.931488000	5.711236000	4.986048000
N	4.160948000	7.377640000	2.697620000
N	3.168275000	4.512299000	3.063941000
N	1.686486000	5.515525000	4.359371000
C	7.254008000	6.953566000	5.444947000
C	3.412540000	8.408878000	2.495468000
C	7.412668000	7.834072000	4.421442000
C	5.979181000	5.322971000	6.687720000
C	3.004649000	5.309743000	4.143346000
C	5.046492000	6.356301000	4.693730000
C	6.369248000	8.001050000	3.495057000
C	5.190242000	7.294232000	3.606404000
C	6.095083000	3.956817000	6.480094000

[L2a+Bcat]$^{+}$
E [Eh]: −1374.805726013340

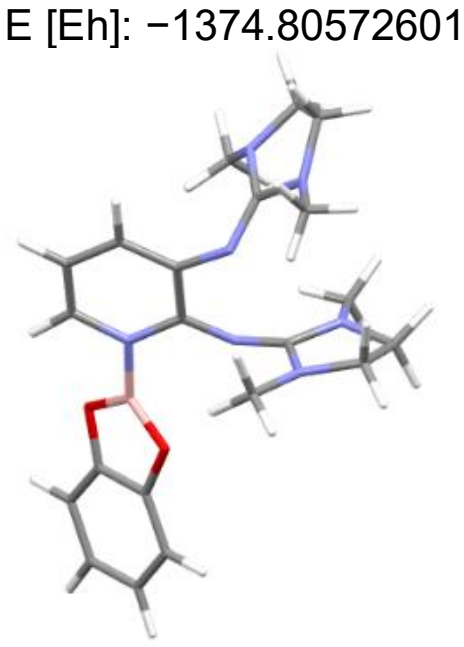

N	5.766431000	7.238184000	6.511270000
N	3.389654000	9.607746000	2.754986000
N	2.938383000	7.920785000	1.356623000
N	3.637177000	6.668419000	5.858026000
N	4.264522000	7.395651000	3.188565000
N	2.947642000	4.918107000	4.395354000
N	1.465822000	6.439477000	5.002610000
C	6.942970000	7.936848000	6.330025000
C	3.600932000	8.268094000	2.508262000
C	7.245750000	8.473329000	5.123017000
C	2.760325000	6.063422000	5.079632000
C	4.823437000	7.126335000	5.514216000
C	6.347150000	8.309587000	4.049404000
C	5.148842000	7.649357000	4.207858000
C	2.390145000	10.120716000	1.813976000
H	2.623919000	11.140115000	1.510473000

H	6.260120000	3.572927000	5.482505000
C	5.776545000	5.845491000	7.955567000
H	5.685660000	6.915268000	8.090471000
C	2.147175000	10.340677000	2.720260000
H	2.353882000	11.408518000	2.775537000
H	1.238445000	10.130307000	3.297888000
C	5.798739000	3.607692000	8.844878000
H	5.730895000	2.935355000	9.689677000
C	2.024131000	9.825303000	1.292036000
H	0.996245000	9.808058000	0.931490000
H	2.626569000	10.421964000	0.595758000
C	6.003419000	3.097648000	7.566577000
H	6.098044000	2.030643000	7.415719000
C	3.452666000	9.585658000	4.685305000
H	2.656992000	9.036308000	5.202729000
H	3.428171000	10.623073000	5.015942000
H	4.413116000	9.167278000	4.965933000
C	5.687647000	4.979123000	9.039206000
H	5.532057000	5.377808000	10.032694000
C	4.428251000	4.228002000	2.418461000
H	4.692034000	4.999424000	1.691330000
H	4.357799000	3.262349000	1.918915000
H	5.215321000	4.167803000	3.166870000
C	1.896842000	4.337552000	2.367843000
H	1.790452000	3.317711000	2.001241000
H	1.829273000	5.025791000	1.518994000
C	2.802322000	7.698073000	0.219755000
H	3.488706000	8.207092000	-0.467213000
H	1.856835000	7.528121000	-0.295789000
H	3.238022000	6.743680000	0.500291000
C	1.140070000	6.064147000	5.578944000
C	0.883871000	4.690373000	3.462200000
H	0.026162000	5.243789000	3.082882000
H	0.521697000	3.799100000	3.986698000
H	8.000547000	6.758054000	6.197770000
H	6.473581000	8.704877000	2.679749000
H	8.333304000	8.390673000	4.331366000
H	0.341962000	6.770292000	5.345269000
H	1.933007000	6.575809000	6.117634000
H	0.733604000	5.274046000	6.218189000

H	1.396188000	10.113708000	2.278465000
C	2.471997000	9.118873000	0.669391000
H	1.513327000	8.950218000	0.180187000
H	3.198955000	9.434314000	-0.089385000
C	3.363745000	10.168817000	4.094011000
H	2.462381000	9.866368000	4.639844000
H	3.370990000	11.255057000	4.014378000
H	4.238269000	9.868632000	4.660468000
C	4.232243000	4.313608000	4.127033000
H	4.709555000	4.755414000	3.249229000
H	4.089659000	3.245848000	3.965604000
H	4.879235000	4.443454000	4.992394000
C	1.757624000	4.599234000	3.611038000
H	1.562480000	3.528096000	3.616554000
H	1.887063000	4.930489000	2.575936000
C	3.333368000	6.767959000	0.575168000
H	4.125333000	7.019469000	-0.139908000
H	2.473681000	6.388643000	0.022184000
H	3.703037000	5.996701000	1.244367000
C	0.854968000	7.436518000	5.851054000
C	0.675045000	5.410277000	4.333112000
H	-0.047457000	5.854550000	3.650267000
H	0.134868000	4.806648000	5.070828000
H	7.582948000	8.000168000	7.195520000
H	6.585285000	8.721541000	3.077287000
H	8.173737000	9.009457000	4.992134000
H	0.186563000	8.068123000	5.263912000
H	1.637370000	8.047819000	6.292433000
H	0.277651000	6.969193000	6.654966000
B	5.575624000	6.547405000	7.798664000
O	5.010645000	5.307839000	7.965171000
O	6.094866000	7.061033000	8.962242000
C	5.837830000	6.087308000	9.921290000
C	5.179578000	5.025334000	9.315566000
C	4.793386000	3.906030000	10.017733000
C	6.146526000	6.093623000	11.262305000
C	5.760900000	4.963703000	11.988041000
C	5.101160000	3.896276000	11.380511000
H	6.658732000	6.925753000	11.724077000
H	5.981456000	4.917805000	13.045683000
H	4.283701000	3.081972000	9.538845000
H	4.821017000	3.038399000	11.976435000

[L2a+Bn(p-CF_3)]$^+$
E [Eh]: −1576.371953087639

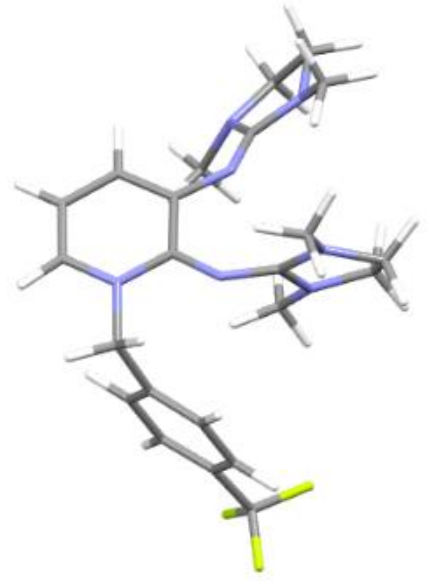

N	2.814144000	7.018646000	11.961230000
N	0.796228000	7.663400000	10.864067000
N	2.435344000	9.132142000	11.021269000
N	1.093652000	7.991352000	13.909825000
N	-0.323465000	9.763119000	14.357971000
N	-1.007480000	7.774970000	15.114633000
N	3.295051000	5.197386000	13.267719000
C	2.595747000	6.359353000	13.081946000
C	1.744806000	6.820356000	14.159709000
C	1.788841000	6.130762000	15.359374000
H	1.212761000	6.500018000	16.197077000
C	2.579439000	4.980570000	15.512472000
H	2.613980000	4.452055000	16.453336000
C	3.307154000	4.525341000	14.457838000
H	3.916413000	3.636436000	14.491642000
C	2.025016000	7.897680000	11.375778000
C	0.229198000	8.898493000	10.330453000
H	-0.443224000	9.353265000	11.066575000

[L2a+Bn(o,p-CF_3)$_2$]$^+$
E [Eh]: −1913.443951727247

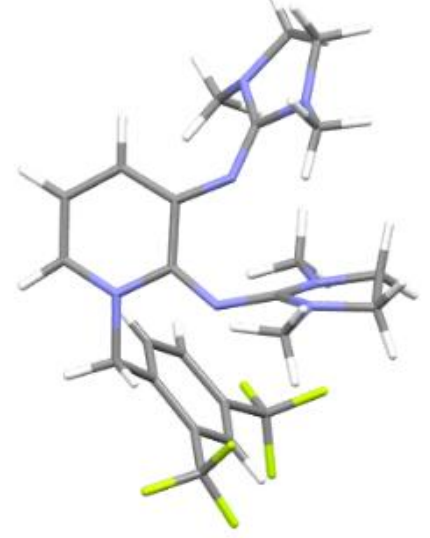

N	2.735517000	6.857482000	11.999735000
N	0.812207000	7.696191000	10.869558000
N	2.580219000	9.001359000	11.070575000
N	1.083326000	7.944068000	13.944791000
N	-0.288033000	9.764151000	14.335422000
N	-1.051325000	7.807201000	15.098058000
N	3.194084000	5.073323000	13.346414000
C	2.519397000	6.248747000	13.141427000
C	1.691616000	6.755599000	14.216635000
C	1.714854000	6.080816000	15.424090000
H	1.149182000	6.476587000	16.256879000
C	2.471879000	4.907781000	15.592359000
H	2.490021000	4.391405000	16.540343000
C	3.188934000	4.417489000	14.546823000
H	3.777319000	3.515085000	14.594110000
C	2.044969000	7.812105000	11.409361000
C	0.383486000	8.974963000	10.309982000
H	-0.267457000	9.495156000	11.021532000

H	-0.327733000	8.708001000	9.414476000
C	1.483048000	9.751150000	10.104667000
H	1.844860000	9.682628000	9.072938000
H	1.330990000	10.801508000	10.349590000
C	-0.030790000	6.521320000	11.164260000
H	0.597461000	5.672260000	11.417659000
H	-0.616768000	6.259581000	10.283595000
H	-0.709472000	6.727817000	11.995976000
C	3.781337000	9.630368000	11.186377000
H	4.290907000	9.024577000	11.930439000
H	3.753755000	10.666791000	11.525358000
H	4.338409000	9.584007000	10.245457000
C	0.000088000	8.434071000	14.443010000
C	-1.447655000	10.067680000	15.233138000
H	-2.103378000	10.818379000	14.793460000
H	-1.089860000	10.443193000	16.199477000
C	-2.113408000	8.703064000	15.371916000
H	-2.545761000	8.534130000	16.357176000
H	-2.898930000	8.564156000	14.619505000
C	0.675591000	10.776316000	14.091057000
H	0.198726000	11.637827000	13.623081000
H	1.423958000	10.362797000	13.420552000
H	1.169932000	11.107604000	15.011414000
C	-1.386022000	6.400347000	14.839775000
H	-1.883359000	5.983874000	15.716037000
H	-0.517571000	5.791937000	14.616949000
H	-2.081634000	6.347343000	13.993851000
C	3.992073000	4.622380000	12.100194000
H	4.592879000	3.792705000	12.468393000
H	4.656841000	5.376616000	11.685752000
C	2.996954000	4.161923000	11.061534000
C	2.005018000	3.246277000	11.402990000
H	1.973933000	2.826158000	12.400801000
C	1.031281000	2.881432000	10.482817000
H	0.258433000	2.177958000	10.757030000
C	1.051002000	3.431486000	9.207286000
C	2.058236000	4.321772000	8.845351000
C	3.022365000	4.685867000	9.772058000
H	3.785576000	5.403702000	9.502183000
C	-0.031366000	3.115535000	8.209804000
F	0.472020000	2.652157000	7.052417000
F	-0.743715000	4.230940000	7.906058000
F	-0.904785000	2.205213000	8.670447000
H	2.077668000	4.740136000	7.847809000

H	-0.158688000	8.830745000	9.376770000
C	1.719177000	9.702633000	10.122245000
H	2.107017000	9.590287000	9.104184000
H	1.660947000	10.764464000	10.357115000
C	-0.133209000	6.648864000	11.160902000
H	0.395499000	5.772551000	11.524110000
H	-0.666512000	6.374383000	10.250277000
H	-0.858670000	6.966854000	11.914948000
C	3.976211000	9.345036000	11.229081000
H	4.418865000	8.684591000	11.968926000
H	4.066060000	10.377447000	11.568445000
H	4.516544000	9.233419000	10.284560000
C	-0.007440000	8.427653000	14.445843000
C	-1.427407000	10.112901000	15.173783000
H	-2.047183000	10.877034000	14.706062000
H	-1.086921000	10.489702000	16.145791000
C	-2.137310000	8.770129000	15.308551000
H	-2.605251000	8.627158000	16.281515000
H	-2.902202000	8.644217000	14.532879000
C	0.747692000	10.743861000	14.082034000
H	0.311349000	11.608966000	13.582184000
H	1.505546000	10.296646000	13.444459000
H	1.221117000	11.079173000	15.011759000
C	-1.458074000	6.439260000	14.831188000
H	-1.994813000	6.049977000	15.696543000
H	-0.598724000	5.805194000	14.647590000
H	-2.125998000	6.390319000	13.962928000
C	3.903579000	4.498016000	12.193154000
H	4.497245000	3.661797000	12.559205000
H	4.579041000	5.250930000	11.803148000
C	2.916648000	4.028429000	11.139325000
C	1.772023000	3.356379000	11.559272000
H	1.625627000	3.162211000	12.613567000
C	0.800794000	2.942999000	10.659884000
H	-0.079008000	2.421381000	11.009092000
C	0.966792000	3.203445000	9.308281000
C	2.118378000	3.839510000	8.862215000
C	3.092428000	4.243916000	9.765998000
C	-0.109881000	2.846820000	8.316674000
F	0.401420000	2.361129000	7.175026000
F	-0.838574000	3.942965000	7.989464000
F	-0.967289000	1.936692000	8.806628000
H	2.261842000	4.015713000	7.806392000
C	4.335258000	4.913891000	9.225317000
F	4.422553000	4.819307000	7.892305000
F	5.454567000	4.363077000	9.741787000
F	4.376089000	6.233700000	9.526091000

[L2a+Bn(*o*,*p*-CF_3)$_3$]$^+$
E [Eh]: −2250.511204000903

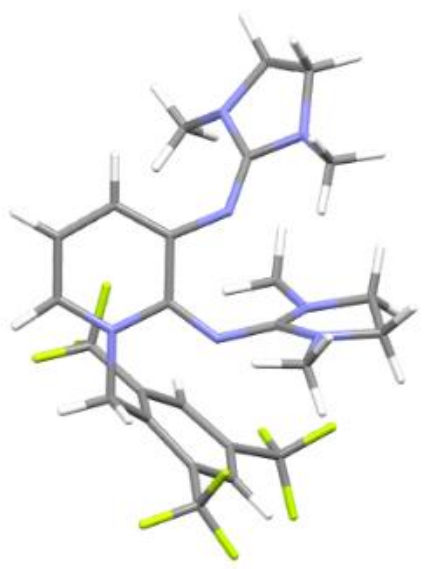

N	2.547710000	6.644186000	11.963115000
N	0.594259000	7.479718000	10.875829000
N	2.432759000	8.699184000	10.850274000
N	1.098684000	8.021544000	13.873217000
N	-0.243221000	9.891689000	14.096855000
N	-0.964213000	8.059260000	15.154575000
N	3.062658000	5.011873000	13.477302000
C	2.412570000	6.182515000	13.183682000
C	1.700357000	6.855173000	14.248921000
C	1.827726000	6.355718000	15.529940000
H	1.351068000	6.879758000	16.347594000
C	2.579345000	5.195506000	15.787380000
H	2.682511000	4.813516000	16.791819000

[L2a+Bn(*o*-CF_3)]$^+$
E [Eh]: −1576.370298394080

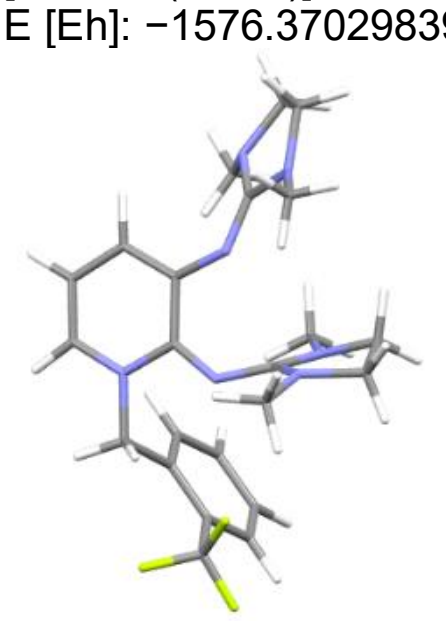

N	2.658403000	6.891195000	11.991020000
N	0.710969000	7.738137000	10.905781000
N	2.493492000	9.031524000	11.058126000
N	1.014199000	7.954866000	13.955803000
N	-0.209074000	9.849937000	14.466839000
N	-1.061608000	7.926176000	15.220609000
N	3.058216000	5.051987000	13.284240000
C	2.402309000	6.240610000	13.104535000
C	1.571729000	6.732406000	14.185446000
C	1.554671000	6.010638000	15.366816000
H	0.992004000	6.393215000	16.207462000
C	2.274684000	4.812032000	15.502584000
H	2.257255000	4.256968000	16.428481000

C	3.174929000	4.540175000	14.758785000
H	3.735421000	3.628423000	14.879028000
C	1.863309000	7.581337000	11.334097000
C	0.195373000	8.720423000	10.215078000
H	-0.403262000	9.330099000	10.900616000
H	-0.392787000	8.514905000	9.321991000
C	1.547925000	9.372037000	9.904879000
H	1.873033000	9.177114000	8.877701000
H	1.546700000	10.449024000	10.068357000
C	-0.402859000	6.567579000	11.377406000
H	0.078312000	5.740065000	11.887813000
H	-0.990678000	6.174031000	10.547315000
H	-1.072952000	7.071472000	12.079587000
C	3.832164000	9.040032000	10.970516000
H	4.291329000	8.384131000	11.703812000
H	3.934803000	10.075674000	11.298236000
H	4.347471000	8.918522000	10.014415000
C	0.040524000	8.576607000	14.367427000
C	-1.322049000	10.360140000	14.957248000
H	-1.966946000	11.068184000	14.437976000
H	-0.915813000	10.851244000	15.849836000
C	-2.029481000	9.055042000	15.306637000
H	-2.436479000	9.043697000	16.316779000
H	-2.842579000	8.842179000	14.602103000
C	0.783964000	10.814665000	13.661569000
H	0.324373000	11.624930000	13.095002000
H	1.488974000	10.280432000	13.030542000
H	1.327732000	11.245569000	14.510025000
C	-1.388194000	6.671015000	15.105351000
H	-1.845674000	6.400988000	16.057371000
H	-0.548092000	6.010357000	14.928594000
H	-2.129885000	6.516941000	14.312767000
C	3.744064000	4.298441000	12.379132000
H	4.161617000	3.387345000	12.798211000
H	4.565819000	4.923658000	12.051839000
C	2.832477000	3.981027000	11.200389000
C	1.589382000	3.348557000	11.353844000
C	0.693686000	3.258318000	10.291148000
H	-0.270423000	2.797088000	10.441375000
C	1.033354000	3.754756000	9.048913000
C	2.311263000	4.252377000	8.838960000
C	3.204396000	4.350159000	9.893880000
C	0.019999000	3.805120000	7.934479000
F	0.538311000	3.422237000	6.759282000
F	-0.428390000	5.077389000	7.775306000
F	-1.051351000	3.039889000	8.189284000
H	2.610560000	4.569002000	7.850964000
C	4.591085000	4.879588000	9.571445000
F	4.823797000	4.880292000	8.251517000
F	5.555236000	4.129597000	10.142934000
F	4.774608000	6.148925000	9.998916000
C	1.111572000	2.749579000	12.660696000
F	0.203743000	1.785727000	12.457772000
F	0.514079000	3.675465000	13.454839000
F	2.112896000	2.197672000	13.370970000

C	3.008247000	4.349529000	14.455233000
H	3.577894000	3.434417000	14.480612000
C	1.960796000	7.843330000	11.410582000
C	0.272519000	9.026175000	10.377492000
H	-0.339067000	9.550875000	11.120337000
H	-0.311951000	8.896822000	9.467866000
C	1.608304000	9.737449000	10.136615000
H	1.956432000	9.614318000	9.105207000
H	1.570601000	10.801201000	10.366967000
C	-0.222119000	6.682789000	11.207788000
H	0.322220000	5.765462000	11.417286000
H	-0.858183000	6.509243000	10.339713000
H	-0.852682000	6.932754000	12.065188000
C	3.898128000	9.358650000	11.167154000
H	4.355152000	8.700598000	11.900437000
H	4.011809000	10.393826000	11.490705000
H	4.407611000	9.228001000	10.207953000
C	-0.014746000	8.494690000	14.525898000
C	-1.264739000	10.248613000	15.388331000
H	-1.855549000	11.070931000	14.986531000
H	-0.836355000	10.564978000	16.347086000
C	-2.060335000	8.955407000	15.528265000
H	-2.477083000	8.812866000	16.524334000
H	-2.878054000	8.909835000	14.799195000
C	0.875742000	10.763878000	14.174853000
H	0.468444000	11.678307000	13.743123000
H	1.550253000	10.292670000	13.465155000
H	1.438810000	11.023445000	15.078514000
C	-1.584010000	6.604403000	14.923253000
H	-2.087637000	6.209557000	15.805948000
H	-0.787614000	5.922571000	14.648708000
H	-2.311636000	6.647491000	14.103987000
C	3.837691000	4.532568000	12.149434000
H	4.440394000	3.706505000	12.524306000
H	4.508337000	5.320182000	11.824992000
C	2.939470000	4.061781000	11.021059000
C	1.761934000	3.387769000	11.335246000
H	1.505857000	3.220956000	12.373640000
C	0.905232000	2.927102000	10.344001000
H	0.000427000	2.400066000	10.617328000
C	1.217470000	3.136645000	9.008765000
C	2.393797000	3.794152000	8.676218000
C	3.254753000	4.252265000	9.669129000
H	2.651783000	3.946842000	7.638570000
C	4.525850000	4.953692000	9.254568000
F	4.751773000	4.870003000	7.936367000
F	5.609068000	4.436246000	9.877517000
F	4.500733000	6.275201000	9.561317000
H	0.559761000	2.780265000	8.227643000

[L2a+KBn]+
E [Eh]: −1352.623355809572

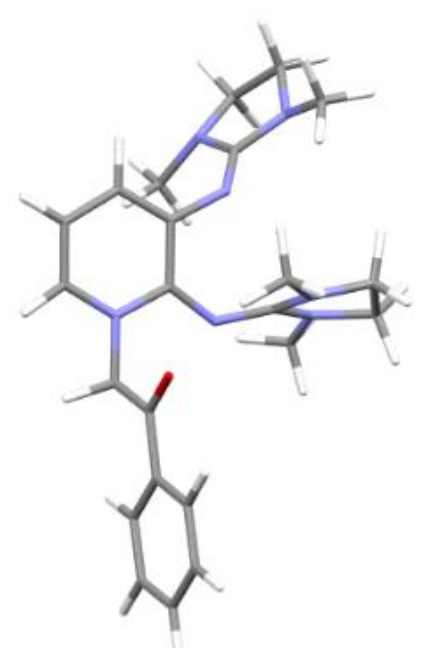

N	5.820476000	6.897282000	6.085739000
N	3.356750000	9.769172000	2.832648000
N	2.729114000	8.317652000	1.249516000

[L2a+F_2Bn]+
E [Eh]: −1437.794566675058

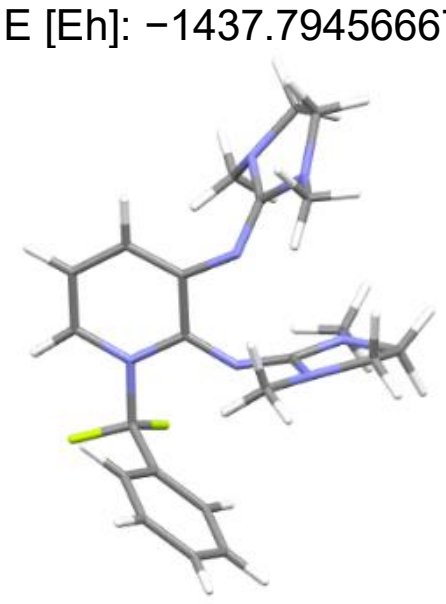

N	5.943942000	7.137839000	6.251397000
N	3.262642000	9.669265000	2.701581000
N	2.868404000	8.020281000	1.242458000

N	3.675153000	6.319169000	5.503128000
N	4.085930000	7.467921000	2.933556000
N	2.958087000	4.792929000	3.807805000
N	1.455495000	6.072365000	4.801583000
C	7.021825000	7.508781000	5.871071000
C	3.462402000	8.462957000	2.399308000
C	7.272949000	8.146803000	4.697691000
C	5.753502000	4.665740000	7.143826000
C	2.774337000	5.784523000	4.701283000
C	4.826664000	6.864446000	5.144444000
C	6.280633000	8.166460000	3.704795000
C	5.062234000	7.547827000	3.896541000
C	5.450614000	3.794812000	8.300056000
C	5.599387000	6.182498000	7.335344000
C	2.344974000	10.460569000	2.027754000
H	2.625709000	11.497643000	1.850370000
H	1.376167000	10.442584000	2.542232000
C	2.306771000	9.622059000	0.756178000
H	1.316134000	9.576495000	0.304946000
H	3.013837000	9.996525000	0.005242000
C	3.409744000	10.117983000	4.242079000
H	2.560429000	9.697115000	4.793989000
H	3.376916000	11.202883000	4.328911000
H	4.333119000	9.773056000	4.695684000
C	5.598402000	2.411729000	8.143515000
C	4.235389000	4.326685000	3.313358000
H	4.560664000	4.907111000	2.447179000
H	4.137196000	3.278250000	3.033153000
H	4.983553000	4.400204000	4.098816000
C	1.715260000	4.525275000	3.090111000
H	1.588794000	3.457921000	2.916119000
H	1.717518000	5.042279000	2.124970000
C	2.996488000	7.254303000	0.305467000
H	3.753472000	7.551212000	-0.429917000
H	2.079847000	6.994747000	-0.224877000
H	3.360261000	6.385732000	0.846314000
C	0.874502000	6.851605000	5.869744000
C	0.665288000	5.108721000	4.041492000
H	-0.155021000	5.599187000	3.519496000
H	0.249231000	4.344760000	4.708356000
H	7.733844000	7.445108000	6.678328000
H	6.460180000	8.675878000	2.767235000
H	8.227660000	8.626166000	4.542496000
H	0.058473000	7.461126000	5.479865000
H	1.639320000	7.499570000	6.289430000
H	0.481501000	6.208060000	6.664110000
H	4.598824000	6.408392000	7.697265000
H	6.323871000	6.535252000	8.068046000
O	6.123313000	4.234292000	6.073049000
C	5.031479000	4.304033000	9.533966000
C	4.765213000	3.445611000	10.590846000
H	4.910945000	5.368360000	9.683573000
C	5.329234000	1.557349000	9.198803000
C	4.912667000	2.073137000	10.423869000
H	4.444243000	3.845430000	11.543441000
H	5.446306000	0.489161000	9.073006000
H	4.704890000	1.404394000	11.249045000
H	5.928584000	2.030802000	7.187061000

N	3.880200000	6.314185000	5.646950000
N	4.156642000	7.458617000	3.094822000
N	3.048372000	4.755042000	4.060357000
N	1.645609000	6.220594000	4.934161000
C	7.023341000	7.971006000	6.066374000
C	3.499832000	8.342568000	2.416423000
C	7.156983000	8.668838000	4.912183000
C	5.831912000	4.822261000	7.171761000
C	2.932131000	5.826336000	4.866370000
C	4.937641000	7.008528000	5.316019000
C	6.191692000	8.530149000	3.897122000
C	5.092381000	7.719790000	4.063938000
C	6.888335000	4.276436000	6.447011000
H	7.697365000	4.907754000	6.101782000
C	4.796742000	4.013599000	7.622559000
H	3.979351000	4.447155000	8.179593000
C	5.863640000	6.297825000	7.477422000
F	4.804425000	6.706793000	8.207249000
F	6.964286000	6.583555000	8.222145000
C	2.276025000	10.198966000	1.755274000
H	2.505138000	11.228366000	1.483811000
H	1.274602000	10.170538000	2.201939000
C	5.867534000	2.103952000	6.614896000
H	5.882082000	1.043180000	6.401551000
C	2.386661000	9.229837000	0.584955000
H	1.436306000	9.058826000	0.080649000
H	3.116268000	9.576226000	-0.157370000
C	6.905314000	2.916304000	6.169965000
H	7.729221000	2.491056000	5.612583000
C	3.184516000	10.173247000	4.062001000
H	2.274318000	9.826090000	4.565339000
H	3.166882000	11.261661000	4.027961000
H	4.048691000	9.868805000	4.642076000
C	4.816531000	2.653418000	7.340607000
H	4.012177000	2.021636000	7.693493000
C	4.308820000	4.214466000	3.603443000
H	4.689136000	4.765612000	2.739822000
H	4.167404000	3.168192000	3.336853000
H	5.035807000	4.265540000	4.409691000
C	1.799923000	4.537391000	3.333716000
H	1.578992000	3.474987000	3.245754000
H	1.868503000	4.968801000	2.330245000
C	3.299607000	6.902891000	0.429008000
H	4.080646000	7.201814000	-0.279640000
H	2.452688000	6.509574000	-0.133853000
H	3.696658000	6.127271000	1.076493000
C	1.119432000	7.137820000	5.919081000
C	0.786119000	5.292255000	4.203506000
H	0.041327000	5.827008000	3.615798000
H	0.268357000	4.627183000	4.902957000
H	7.731006000	8.015741000	6.873317000
H	6.303564000	9.069980000	2.965981000
H	8.011337000	9.315893000	4.782254000
H	0.486433000	7.885729000	5.438548000
H	1.948708000	7.631399000	6.418467000
H	0.523703000	6.605320000	6.666339000

$[L2a+BnF_5]^+$
E [Eh]: −1735.510816384671

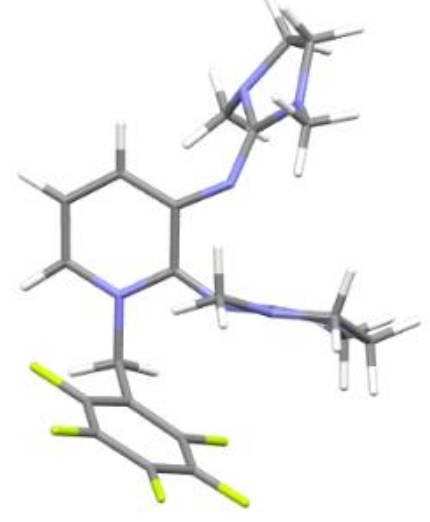

N	6.179806000	7.497531000	6.120338000
N	3.338306000	9.885511000	2.522438000

$[L2a+ABn]^+$
E [Eh]: −1313.314956854713

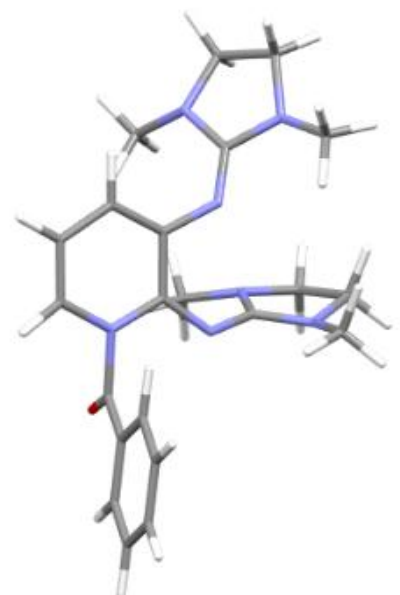

N	5.567634000	6.652544000	6.145101000
N	3.292112000	9.774867000	2.552175000

N	2.700591000	8.097215000	1.345424000
N	4.098660000	6.598947000	5.772325000
N	4.067842000	7.671931000	3.174685000
N	3.701105000	4.745334000	4.313986000
N	2.011648000	5.611758000	5.448039000
C	7.223655000	8.318345000	5.801365000
C	3.438647000	8.514584000	2.416095000
C	7.244662000	8.962167000	4.602377000
C	6.351411000	5.273797000	7.184234000
C	3.327150000	5.737612000	5.157547000
C	5.111777000	7.300911000	5.288017000
C	6.186505000	8.779684000	3.700679000
C	5.107794000	7.967749000	4.010561000
C	7.504804000	4.757843000	6.602619000
F	8.508842000	5.590293000	6.294752000
C	5.342532000	4.368677000	7.494897000
F	4.214181000	4.781845000	8.080262000
C	6.202236000	6.756840000	7.402462000
H	5.277301000	6.958995000	7.934427000
H	7.037304000	7.149769000	7.977310000
C	2.335890000	10.369718000	1.565628000
H	2.636802000	11.322189000	1.132227000
H	1.371638000	10.502982000	2.070018000
C	6.612343000	2.537442000	6.601848000
F	6.726279000	1.247647000	6.315037000
C	2.271725000	9.237955000	0.546564000
H	1.272312000	9.086174000	0.140370000
H	2.964616000	9.403415000	-0.287371000
C	7.651194000	3.411963000	6.306495000
F	8.764175000	2.958797000	5.739890000
C	3.371646000	10.566743000	3.806746000
H	2.470712000	10.354747000	4.394215000
H	3.424246000	11.639574000	3.626547000
H	4.244545000	10.279013000	4.382250000
C	5.455257000	3.018182000	7.199809000
F	4.453139000	2.185618000	7.475229000
C	4.981818000	4.623094000	3.659817000
H	5.018167000	5.178598000	2.720295000
H	5.176793000	3.568521000	3.466195000
H	5.766343000	4.996001000	4.313409000
C	2.527933000	4.007989000	3.851611000
H	2.741913000	2.942551000	3.782343000
H	2.209291000	4.368292000	2.867530000
C	2.905629000	6.810718000	0.719647000
H	3.700965000	6.846965000	-0.033838000
H	1.982076000	6.491139000	0.236863000
H	3.180132000	6.089981000	1.483823000
C	1.375479000	6.278601000	6.563225000
C	1.500193000	4.343742000	4.934883000
H	0.490523000	4.456060000	4.543787000
H	1.489226000	3.589087000	5.729715000
H	8.009686000	8.395104000	6.534451000
H	6.198899000	9.286839000	2.744973000
H	8.078963000	9.602222000	4.357015000
H	0.333911000	6.483942000	6.317033000
H	1.890735000	7.215702000	6.754907000
H	1.413360000	5.662736000	7.467507000

[L2a+BnPh]$^{+}$
E [Eh]: −1470.308857263878

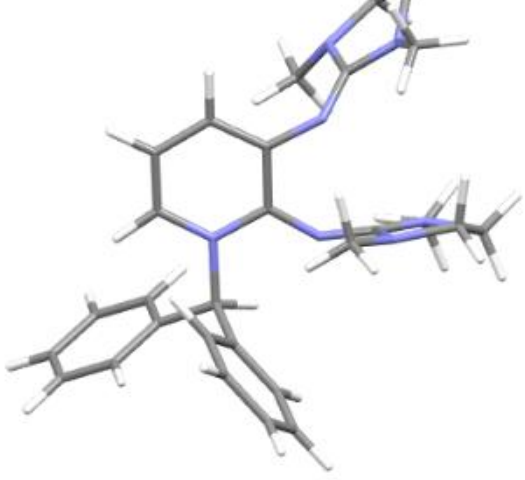

N	5.720615000	6.852437000	6.265927000
N	3.472062000	9.683829000	2.900684000
N	2.906112000	8.238542000	1.289449000
N	3.592072000	6.392112000	5.502307000

N	3.005985000	8.172794000	1.020403000
N	3.828359000	5.613371000	5.081234000
N	3.983183000	7.497862000	3.012765000
N	2.151534000	4.664592000	3.752906000
N	1.638602000	6.527121000	4.840069000
C	6.586250000	7.554244000	6.255152000
C	3.484142000	8.437210000	2.276616000
C	6.738384000	8.510549000	5.299083000
C	6.142554000	4.516840000	7.314007000
C	2.622190000	5.638334000	4.556001000
C	4.677535000	6.624810000	5.114181000
C	5.843651000	8.550087000	4.216282000
C	4.807699000	7.637922000	4.090660000
C	7.018645000	4.194068000	6.272347000
H	7.107275000	4.842912000	5.412678000
C	6.027445000	3.665692000	8.420889000
H	5.343511000	3.929601000	9.215670000
C	5.315043000	5.723362000	7.288483000
C	2.500592000	10.384128000	1.479071000
H	2.845227000	11.394029000	1.261525000
H	1.445491000	10.432559000	1.774759000
C	7.657679000	2.193373000	7.443593000
H	8.250117000	1.289115000	7.495347000
C	2.705740000	9.414961000	0.320521000
H	1.824019000	9.313577000	-0.311419000
H	3.551878000	9.712193000	-0.311041000
C	7.772443000	3.032664000	6.339891000
H	8.450547000	2.781117000	5.535576000
C	3.038736000	10.272401000	3.891790000
H	1.991519000	10.117220000	4.178014000
H	3.246829000	11.341867000	3.918765000
H	3.677924000	9.782577000	4.617100000
C	6.785120000	2.509958000	8.483061000
H	6.700584000	1.853769000	9.338817000
C	3.003752000	3.751005000	3.025289000
H	3.198474000	4.116012000	2.011994000
H	2.523894000	2.774442000	2.962739000
H	3.946556000	3.654838000	3.556664000
C	0.817447000	5.004307000	3.269802000
H	0.166752000	4.131029000	3.252168000
H	0.883380000	5.412775000	2.255317000
C	3.432293000	7.012718000	0.267211000
H	4.324003000	7.229157000	-0.332315000
H	2.631769000	6.696122000	-0.401799000
H	3.665798000	6.212109000	0.962882000
C	1.632786000	7.385110000	6.007444000
C	0.368236000	6.065799000	4.279191000
H	-0.180842000	6.885273000	3.816779000
H	-0.252612000	5.636482000	5.072871000
H	7.219427000	7.452604000	7.122996000
H	5.976392000	9.293915000	3.441568000
H	7.545065000	9.223918000	5.377546000
H	1.277851000	8.380860000	5.740499000
H	2.632733000	7.463088000	6.422790000
H	0.974681000	6.974695000	6.779448000
O	4.496608000	6.058220000	8.094422000

[L2a+BnPh$_2$]$^{+}$
E [Eh]: −1701.306028168511

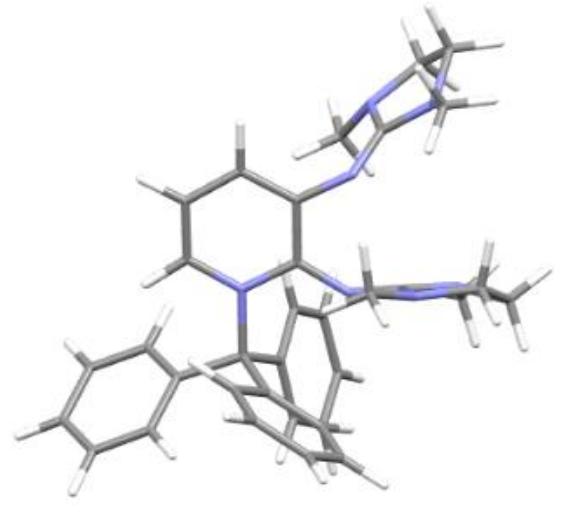

N	5.565488000	6.857113000	6.387071000
N	3.159902000	9.530404000	3.040022000
N	2.970067000	8.165863000	1.276991000
N	3.669059000	6.072848000	5.315294000

N	4.293793000	7.407630000	2.953948000
N	3.001872000	4.776427000	3.846840000
N	1.454753000	6.175945000	4.571305000
C	6.951208000	7.423981000	6.146050000
C	3.628147000	8.388289000	2.449701000
C	7.333783000	8.012660000	4.979088000
C	5.477729000	4.611964000	7.244375000
C	2.762712000	5.837041000	4.646163000
C	4.807420000	6.856284000	5.245014000
C	6.442138000	8.033224000	3.896537000
C	5.183326000	7.478871000	4.002334000
C	6.568678000	4.081057000	6.559108000
H	7.346520000	4.733270000	6.182546000
C	4.483985000	3.752730000	7.704072000
H	3.628918000	4.159887000	8.229820000
C	5.347509000	6.096377000	7.507860000
C	2.450465000	10.355939000	2.094313000
H	2.700641000	11.404509000	1.939130000
H	1.475060000	10.299395000	2.594023000
C	5.671676000	1.859059000	6.808590000
H	5.747657000	0.792778000	6.641010000
C	2.458899000	9.539198000	0.808799000
H	1.478636000	9.474819000	0.337697000
H	3.169522000	9.946967000	0.078605000
C	6.666236000	2.711320000	6.343226000
H	7.519926000	2.311051000	5.811925000
C	3.524575000	10.044857000	4.305788000
H	2.656677000	9.661345000	4.855333000
H	3.527639000	11.131495000	4.381440000
H	4.431640000	9.674875000	4.771286000
C	4.579185000	2.383500000	7.490516000
H	3.799886000	1.726560000	7.854280000
C	4.306293000	4.233529000	3.546094000
H	4.785612000	4.771357000	2.724321000
H	4.193892000	3.183913000	3.277323000
H	4.937338000	4.286738000	4.429817000
C	1.839166000	4.496806000	3.010232000
H	1.680276000	3.423834000	2.913103000
H	1.976010000	4.923168000	2.011432000
C	3.226824000	7.203888000	0.330374000
H	3.992821000	7.536450000	-0.380376000
H	2.330864000	6.930378000	-0.227635000
H	3.603184000	6.334861000	0.862144000
C	0.790764000	7.072574000	5.486203000
C	0.714186000	5.201219000	3.778081000
H	-0.001353000	5.694657000	3.121692000
H	0.170219000	4.510361000	4.432140000
H	7.577885000	7.374939000	7.020450000
H	6.726495000	8.497717000	2.961464000
H	8.315257000	8.456028000	4.902866000
H	0.130431000	7.746345000	4.937439000
H	1.542534000	7.653841000	6.013467000
H	0.194082000	6.519759000	6.219091000
C	6.087536000	6.615565000	8.722040000
H	4.290281000	6.312433000	7.643221000
C	7.111925000	5.904307000	9.339806000
C	7.763721000	6.433618000	10.449708000
H	7.396607000	4.931255000	8.964883000
C	5.716149000	7.858404000	9.239392000
H	8.555873000	5.869426000	10.924094000
H	4.911575000	8.413372000	8.770500000
C	7.396755000	7.675377000	10.950545000
C	6.366673000	8.388234000	10.343247000
H	7.902556000	8.083719000	11.815519000
H	6.067248000	9.350872000	10.736609000

N	4.379628000	7.439857000	2.971252000
N	3.497075000	4.559119000	3.477120000
N	1.669974000	5.553269000	4.228599000
C	6.718545000	7.590433000	6.390700000
C	3.577199000	8.329296000	2.500441000
C	7.108317000	8.309343000	5.303799000
C	5.344669000	4.544662000	7.055948000
C	3.017907000	5.467159000	4.352641000
C	4.783555000	6.771667000	5.254858000
C	6.312129000	8.303976000	4.149561000
C	5.156322000	7.558526000	4.102873000
C	6.596046000	4.165167000	6.562395000
H	7.419237000	4.867024000	6.585920000
C	4.304001000	3.625657000	6.984014000
H	3.314471000	3.902694000	7.310659000
C	5.193161000	5.980588000	7.588441000
C	2.090471000	10.087802000	2.209273000
H	2.157748000	11.173871000	2.161914000
H	1.109245000	9.814981000	2.617903000
C	5.766767000	1.977987000	5.998982000
H	5.929394000	0.984808000	5.601411000
C	2.335422000	9.411753000	0.866994000
H	1.418647000	9.227040000	0.308139000
H	3.013404000	10.003356000	0.238513000
C	6.810280000	2.895661000	6.044866000
H	7.792089000	2.624922000	5.678904000
C	3.058156000	9.765026000	4.468608000
H	2.254098000	9.173946000	4.923050000
H	2.843245000	10.820975000	4.627744000
H	3.993842000	9.535797000	4.967559000
C	4.513422000	2.352697000	6.463481000
H	3.687463000	1.654420000	6.420695000
C	4.892027000	4.316690000	3.196912000
H	5.259846000	4.966500000	2.399412000
H	5.016404000	3.273396000	2.908827000
H	5.478141000	4.485998000	4.095210000
C	2.447467000	4.139044000	2.554707000
H	2.515346000	3.071786000	2.348429000
H	2.529840000	4.685448000	1.609632000
C	3.537495000	7.299159000	0.267073000
H	4.323568000	7.804819000	-0.306657000
H	2.753312000	6.982558000	-0.420900000
H	3.969684000	6.427159000	0.748984000
C	0.810470000	6.135358000	5.235016000
C	1.177170000	4.523053000	3.320208000
H	0.392535000	4.911343000	2.672487000
H	0.773477000	3.677341000	3.888973000
H	7.295597000	7.559527000	7.295577000
H	6.595867000	8.881657000	3.279856000
H	8.027387000	8.874365000	5.347604000
H	0.008697000	6.701778000	4.759085000
H	1.398563000	6.793833000	5.867245000
H	0.363547000	5.359940000	5.866353000
C	6.125141000	6.251305000	8.791736000
C	3.805174000	6.369871000	8.119399000
C	6.849642000	5.237082000	9.410821000
C	7.596947000	5.493662000	10.558436000
H	6.829631000	4.232844000	9.018341000
C	6.142844000	7.525558000	9.373387000
H	8.146709000	4.685815000	11.023270000
H	5.550046000	8.322003000	8.944712000
C	7.634687000	6.767035000	11.102754000
C	6.900153000	7.786442000	10.502478000
H	8.217917000	6.965456000	11.992155000
H	6.903874000	8.782236000	10.925999000
C	3.208143000	5.571981000	9.094934000
C	2.008509000	5.945363000	9.686587000
C	3.200774000	7.583234000	7.801802000
C	2.008222000	7.967425000	8.401313000
C	1.397063000	7.143487000	9.338045000
H	3.695005000	4.663018000	9.419180000
H	1.563466000	5.306261000	10.437977000
H	0.468497000	7.441029000	9.806811000
H	1.560159000	8.917109000	8.137480000
H	3.657812000	8.244413000	7.080562000

[L2a+Bn(o-Me)]$^{+}$
E [Eh]: −1278.604780504092

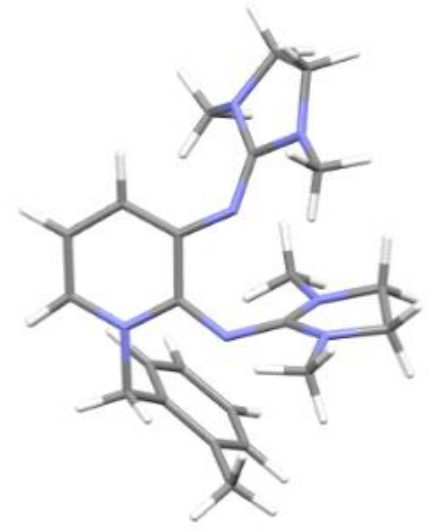

N	2.795534000	6.903216000	12.028531000
N	0.829019000	7.627358000	10.890188000
N	2.533450000	9.019187000	11.055666000
N	1.080989000	7.956664000	13.934533000
N	-0.204905000	9.827847000	14.375487000
N	-1.037937000	7.894027000	15.126200000
N	3.133575000	5.045095000	13.328341000
C	2.501328000	6.240983000	13.132758000
C	1.646295000	6.741639000	14.187882000
C	1.610930000	6.038160000	15.380564000
H	1.033258000	6.432891000	16.205338000
C	2.328059000	4.843409000	15.543664000
H	2.300377000	4.301944000	16.477384000
C	3.066242000	4.360921000	14.507329000
H	3.624260000	3.439432000	14.549896000
C	2.060224000	7.807003000	11.417060000
C	0.328276000	8.882957000	10.340854000
H	-0.323753000	9.381442000	11.067163000
H	-0.232119000	8.711365000	9.423159000
C	1.626190000	9.666247000	10.113543000
H	1.995764000	9.555708000	9.087924000
H	1.525052000	10.728090000	10.333829000
C	-0.039911000	6.510371000	11.164731000
H	0.557100000	5.625149000	11.367337000
H	-0.650704000	6.309568000	10.285028000
H	-0.696472000	6.709759000	12.015823000
C	3.904227000	9.443762000	11.220457000
H	4.372520000	8.825105000	11.981014000
H	3.934134000	10.486906000	11.537759000
H	4.464663000	9.345892000	10.285206000
C	0.019483000	8.477318000	14.459644000
C	-1.298518000	10.212404000	15.257570000
H	-1.893560000	11.017375000	14.827766000
H	-0.909473000	10.547630000	16.226778000
C	-2.069675000	8.903593000	15.384854000
H	-2.519296000	8.762878000	16.366828000
H	-2.858951000	8.831363000	14.627023000
C	0.869975000	10.762218000	14.114609000
H	0.459001000	11.665125000	13.662532000
H	1.578196000	10.300642000	13.431996000
H	1.396343000	11.039705000	15.035101000
C	-1.520500000	6.555550000	14.835977000
H	-2.030411000	6.157852000	15.713875000
H	-0.702667000	5.891038000	14.583780000
H	-2.233617000	6.570783000	14.003149000
C	3.866001000	4.460611000	12.184998000
H	4.415869000	3.604747000	12.573991000
H	4.583951000	5.196169000	11.836692000
C	2.918076000	4.043031000	11.082604000
C	1.842348000	3.217348000	11.408674000
H	1.715362000	2.885449000	12.432551000
C	0.924949000	2.818061000	10.447679000
H	0.100176000	2.172186000	10.718350000
C	1.083233000	3.250672000	9.137066000
C	2.158657000	4.063179000	8.805440000
C	3.093535000	4.470789000	9.759132000
H	2.283816000	4.388529000	7.779956000
C	4.249331000	5.340786000	9.343784000
H	4.170701000	5.603425000	8.289798000
H	5.206886000	4.833025000	9.481985000
H	4.278925000	6.263367000	9.926263000
H	0.378968000	2.948065000	8.373066000

[L3+Bn(p-CF_3)]$^+$
E [Eh]: −2035.931150967078

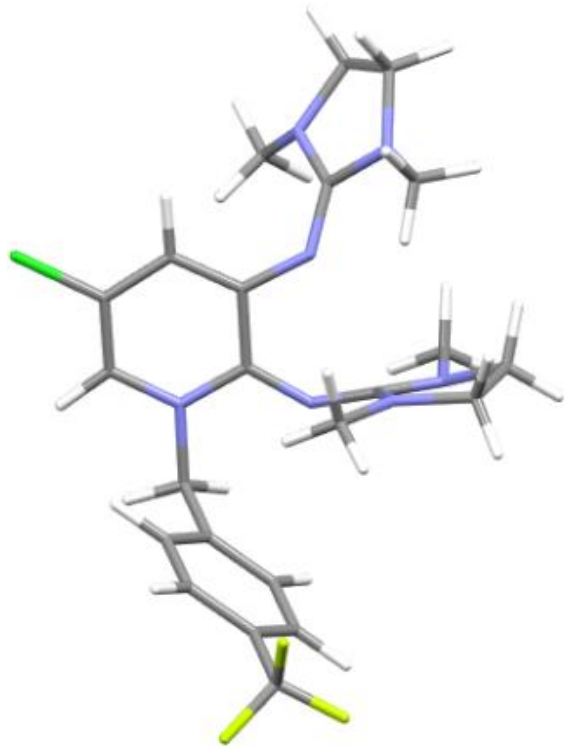

[L4+Bn(p-CF_3)]$^+$
E [Eh]: −2035.929394726632

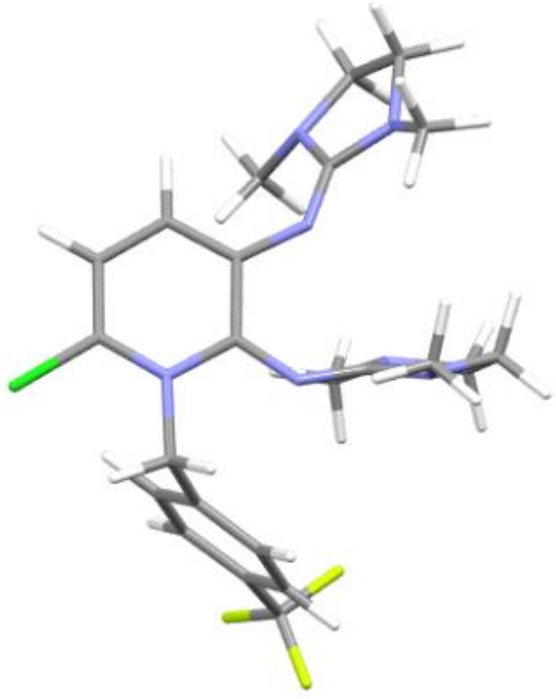

N	7.036378000	6.555736000	7.064462000	N	8.718957000	7.112131000	6.564837000
N	4.390971000	10.188918000	4.451422000	N	4.430138000	9.785835000	4.858414000
N	4.172635000	9.082073000	2.524575000	N	4.138059000	8.523897000	3.037625000
N	5.249279000	5.734885000	5.876592000	N	7.103699000	5.760083000	5.649344000
N	5.325851000	7.969270000	4.202484000	N	5.916698000	7.924532000	4.397298000
N	5.244443000	4.949659000	3.630387000	N	5.517843000	4.488758000	4.498326000
N	3.308496000	5.251385000	4.653135000	N	4.774684000	5.720729000	6.181825000
C	7.959214000	7.515392000	7.381217000	C	9.334147000	8.330226000	6.719859000
C	4.698153000	9.025461000	3.784630000	C	4.921989000	8.712560000	4.144714000
C	8.040135000	8.647849000	6.631387000	C	8.812319000	9.451424000	6.147340000
C	4.650700000	5.386787000	4.753442000	C	5.856462000	5.399486000	5.434492000
C	6.154274000	6.681210000	6.029023000	C	7.561721000	6.981919000	5.826630000
C	7.164009000	8.842801000	5.551422000	C	7.636479000	9.360454000	5.392779000
C	6.204410000	7.897784000	5.241466000	C	6.981036000	8.155870000	5.216989000
C	3.460621000	10.981517000	3.638887000	C	3.156221000	10.215599000	4.272627000
H	3.681120000	12.044808000	3.717806000	H	3.059682000	11.300051000	4.296010000
H	2.431779000	10.811473000	3.977307000	H	2.321802000	9.778916000	4.834856000
C	3.680162000	10.423440000	2.236628000	C	3.227725000	9.647400000	2.860438000
H	2.767572000	10.394040000	1.642311000	H	2.259050000	9.320666000	2.483580000
H	4.435052000	10.996629000	1.685408000	H	3.647343000	10.372543000	2.152776000
C	4.184754000	10.234356000	5.889957000	C	4.523675000	9.883097000	6.303412000
H	3.271150000	9.702221000	6.179747000	H	3.767298000	9.257823000	6.792213000
H	4.088720000	11.276540000	6.190427000	H	4.354178000	10.918336000	6.598848000
H	5.027400000	9.806656000	6.422059000	H	5.505100000	9.585668000	6.654498000
C	6.664300000	4.785105000	3.426577000	C	6.342520000	4.177823000	3.351601000
H	7.049095000	5.534190000	2.730690000	H	6.053037000	4.776500000	2.482326000
H	6.867856000	3.790554000	3.026011000	H	6.239383000	3.121981000	3.101846000
H	7.185703000	4.880337000	4.374090000	H	7.379603000	4.389241000	3.596942000
C	4.261456000	4.442302000	2.677121000	C	4.068537000	4.321867000	4.453399000
H	4.313783000	3.350441000	2.631344000	H	3.794222000	3.277318000	4.312908000
H	4.442637000	4.835497000	1.676579000	H	3.652026000	4.908056000	3.626949000
C	4.645865000	8.237350000	1.450179000	C	4.603319000	7.789306000	1.880756000
H	5.445240000	8.724395000	0.880498000	H	5.073127000	8.454231000	1.147232000
H	3.823914000	8.011003000	0.770022000	H	3.763391000	7.283433000	1.403692000
H	5.030696000	7.316437000	1.878690000	H	5.335801000	7.056659000	2.206261000
C	2.385256000	5.942912000	5.525195000	C	4.824158000	6.317114000	7.498910000
C	2.937187000	4.936597000	3.275725000	C	3.637806000	4.876772000	5.814154000
H	2.579632000	5.842005000	2.773223000	H	2.718368000	5.458760000	5.762962000
H	2.149261000	4.185644000	3.239913000	H	3.506723000	4.082495000	6.556699000
H	1.471220000	5.358807000	5.627109000	H	4.803356000	5.552711000	8.279939000
H	2.132181000	6.932128000	5.127009000	H	3.964473000	6.973943000	7.631471000
H	2.844514000	6.059804000	6.503187000	H	5.729303000	6.903328000	7.615404000
H	8.595101000	7.319054000	8.228201000	Cl	10.770921000	8.428783000	7.666539000
Cl	9.216332000	9.856598000	7.022999000	H	9.320790000	10.393967000	6.276724000
H	7.227785000	9.752864000	4.971302000	H	7.243662000	10.248962000	4.916813000
C	7.017735000	5.299451000	7.842319000	C	9.225731000	5.875449000	7.205691000
C	7.548298000	4.133460000	7.044688000	C	8.333736000	5.429892000	8.337281000
H	5.993016000	5.110149000	8.153299000	H	10.232429000	6.075809000	7.553911000
H	7.623273000	5.474004000	8.729876000	H	9.268067000	5.109664000	6.435867000
C	8.835541000	4.171826000	6.507704000	C	8.032108000	6.292921000	9.387765000
C	9.319098000	3.106680000	5.765349000	C	7.175125000	5.893962000	10.404687000
C	8.516541000	1.986935000	5.557401000	C	6.624241000	4.617850000	10.380880000
C	7.237828000	1.935631000	6.095801000	C	6.946038000	3.737009000	9.352479000
C	6.757493000	3.010692000	6.832890000	C	7.792574000	4.147405000	8.334921000
H	6.618609000	1.064648000	5.936178000	H	6.527908000	2.739621000	9.344348000
H	5.753139000	2.981034000	7.234295000	H	8.019797000	3.473286000	7.519542000
H	9.465198000	5.039420000	6.662395000	H	8.460709000	7.286888000	9.413756000
H	10.317183000	3.141760000	5.349668000	H	6.940171000	6.569691000	11.214939000
C	9.066080000	0.838766000	4.751418000	C	5.613689000	4.199312000	11.414812000

F	8.142844000	-0.110935000	4.521377000	F	5.680096000	4.940725000	12.531190000
F	10.104916000	0.249327000	5.373098000	F	5.753949000	2.909727000	11.765942000
F	9.516236000	1.261927000	3.548267000	F	4.352042000	4.335741000	10.925200000

[L4+Bn(*o*-CF_3)]$^+$
E [Eh]: −2035.923879015556

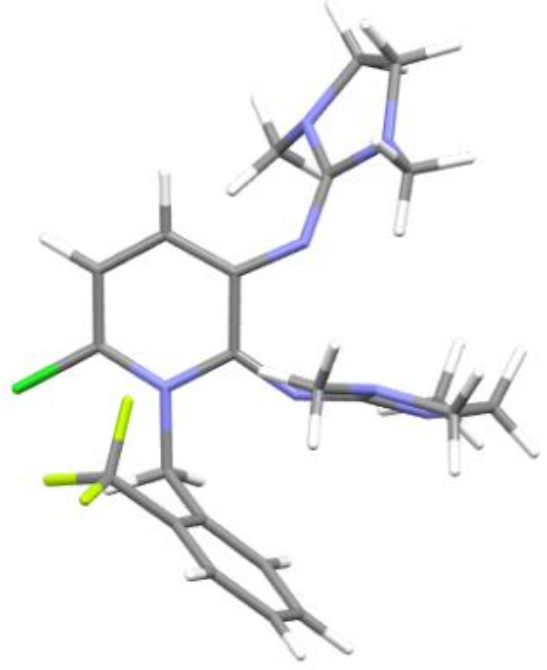

[L4+Bn(*o*,*p*-CF_3)$_2$]$^+$
E [Eh]: −2372.997839874523

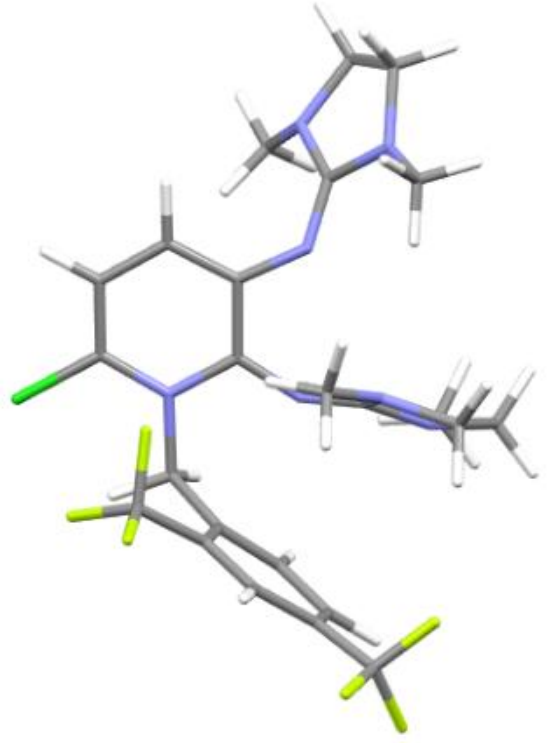

N	7.033281000	6.730023000	7.201135000	N	7.129801000	6.896309000	7.285309000
N	4.432733000	10.311995000	4.413049000	N	4.372936000	10.248528000	4.356480000
N	4.399553000	9.206505000	2.471364000	N	4.433834000	9.084196000	2.450980000
N	5.316635000	5.902475000	5.924247000	N	5.495630000	5.925692000	5.994136000
N	5.463380000	8.124878000	4.224764000	N	5.519154000	8.114242000	4.254387000
N	5.434983000	5.125519000	3.655141000	N	5.825339000	5.104239000	3.755227000
N	3.550212000	4.908721000	4.794792000	N	3.983404000	4.584897000	4.863264000
C	7.806492000	7.776981000	7.642061000	C	7.873388000	7.983099000	7.678137000
C	4.828168000	9.157199000	3.768105000	C	4.839160000	9.097622000	3.754312000
C	7.882477000	8.930776000	6.919477000	C	7.889267000	9.119284000	6.922723000
C	4.817899000	5.399075000	4.824384000	C	5.137475000	5.301007000	4.901741000
C	6.198573000	6.867968000	6.112230000	C	6.310301000	6.953058000	6.176220000
C	7.124738000	9.068714000	5.753385000	C	7.111889000	9.194276000	5.765637000
C	6.245294000	8.083286000	5.342545000	C	6.290346000	8.149188000	5.377339000
C	3.551870000	11.080093000	3.524981000	C	3.471197000	10.942207000	3.428588000
H	3.729756000	12.149513000	3.627067000	H	3.589335000	12.022130000	3.501675000
H	2.502971000	10.874697000	3.769948000	H	2.429985000	10.687099000	3.658959000
C	3.910514000	10.538845000	2.145539000	C	3.887238000	10.380461000	2.073573000
H	3.057182000	10.495599000	1.469549000	H	3.052271000	10.274472000	1.381942000
H	4.700497000	11.132925000	1.670031000	H	4.657248000	10.999539000	1.597448000
C	4.075082000	10.323580000	5.822538000	C	3.986762000	10.283367000	5.758038000
H	3.138976000	9.781186000	6.001211000	H	3.056753000	9.728107000	5.928148000
H	3.941505000	11.357884000	6.136659000	H	3.829065000	11.320976000	6.049534000
H	4.858170000	9.884530000	6.430706000	H	4.764554000	9.869085000	6.389854000
C	6.849597000	5.226145000	3.388146000	C	7.199153000	5.455045000	3.492690000
H	7.005156000	5.653259000	2.396548000	H	7.273449000	6.016947000	2.560516000
H	7.323456000	4.241250000	3.427281000	H	7.810822000	4.552571000	3.407988000
H	7.329454000	5.873950000	4.110934000	H	7.597820000	6.069824000	4.288240000
C	4.590867000	4.282624000	2.812938000	C	5.163089000	4.108786000	2.913775000
H	4.929857000	3.242235000	2.864335000	H	5.696075000	3.155825000	2.974112000
H	4.628835000	4.607374000	1.773264000	H	5.146472000	4.430320000	1.872304000
C	4.958968000	8.359971000	1.441634000	C	5.046677000	8.226212000	1.461708000
H	5.847196000	8.810759000	0.983805000	H	5.963519000	8.665195000	1.051373000
H	4.213370000	8.193027000	0.663905000	H	4.343728000	8.060405000	0.645638000
H	5.237218000	7.410086000	1.889253000	H	5.289155000	7.275869000	1.929132000
C	2.518191000	5.348118000	5.707690000	C	2.875095000	4.807280000	5.762967000
C	3.210395000	4.467441000	3.446697000	C	3.765511000	4.027539000	3.533349000
H	2.627999000	5.239704000	2.930902000	H	3.037887000	4.633774000	2.982299000
H	2.625405000	3.548945000	3.467391000	H	3.389711000	3.006657000	3.591759000
H	1.805608000	4.539674000	5.869997000	H	2.367822000	3.862120000	5.957836000
H	1.980550000	6.217790000	5.313139000	H	2.151093000	5.512401000	5.339958000
H	2.976786000	5.616178000	6.655660000	H	3.254986000	5.209977000	6.697895000
Cl	8.625304000	7.655258000	9.150671000	Cl	8.757237000	7.925927000	9.153197000
H	8.510034000	9.732546000	7.275996000	H	8.492496000	9.953470000	7.245422000
H	7.189643000	9.985749000	5.183199000	H	7.125843000	10.100150000	5.174579000
C	7.029102000	5.414134000	7.888400000	C	7.129726000	5.634568000	8.070066000
C	7.309420000	4.210653000	7.007963000	C	7.352051000	4.367779000	7.270550000
H	6.044800000	5.287490000	8.334692000	H	6.159742000	5.562649000	8.558740000
H	7.758300000	5.474846000	8.686640000	H	7.887006000	5.738797000	8.836947000
C	8.493621000	3.986660000	6.287495000	C	8.494961000	4.077314000	6.512712000
C	8.656615000	2.812764000	5.553947000	C	8.560197000	2.901290000	5.767505000
C	7.665140000	1.843743000	5.530937000	C	7.513288000	1.994291000	5.793760000
C	6.497337000	2.046810000	6.252143000	C	6.408576000	2.234503000	6.601619000
C	6.331558000	3.217359000	6.975837000	C	6.341935000	3.408065000	7.325924000

H	5.717802000	1.296376000	6.256305000
H	5.412132000	3.378909000	7.522256000
H	9.575456000	2.653374000	5.009549000
C	9.620946000	4.987984000	6.250176000
F	9.944921000	5.435418000	7.480008000
F	9.299444000	6.087496000	5.512624000
F	10.735984000	4.485032000	5.706646000
H	7.813600000	0.934033000	4.964968000

H	5.602777000	1.515625000	6.652264000
H	5.463115000	3.611647000	7.921798000
H	9.438215000	2.690980000	5.176067000
C	7.521482000	0.783309000	4.900752000
F	6.943329000	-0.275964000	5.487543000
F	8.754403000	0.428458000	4.518500000
F	6.812464000	1.036564000	3.765341000
C	9.688671000	5.003040000	6.457599000
F	10.064318000	5.411185000	7.684878000
F	9.432499000	6.130334000	5.740912000
F	10.749834000	4.423028000	5.884965000

[L4b+Bn(*p*-CF_3)]$^+$
E [Eh]: −2642.954269457723

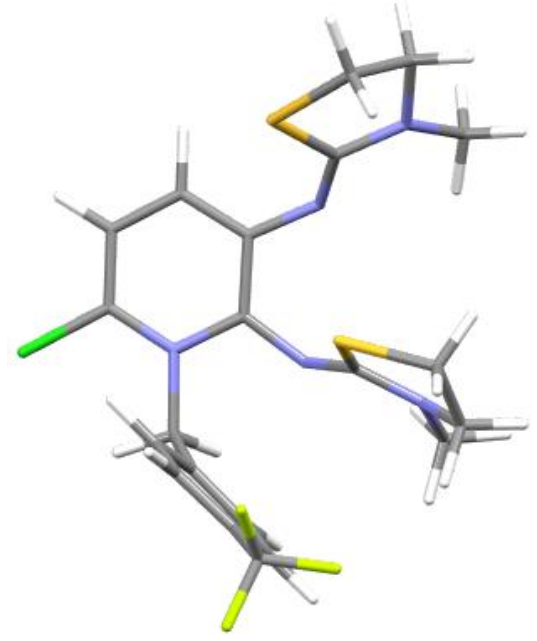

H	10.838604000	1.281711000	12.919095000
S	5.038372000	3.623816000	13.162962000
N	7.249745000	4.979448000	12.332854000
C	8.086331000	3.942652000	12.478627000
Cl	11.249421000	3.335164000	14.787480000
S	6.316803000	0.208188000	11.445773000
N	5.162617000	5.913844000	11.967832000
C	8.057102000	2.781310000	11.651805000
N	7.093060000	2.729853000	10.679396000
C	9.064926000	1.841799000	11.822790000
H	9.091261000	0.971038000	11.182057000
N	5.403113000	1.671208000	9.505421000
C	10.055203000	2.011589000	12.786042000
N	9.074988000	4.079293000	13.424679000
C	10.044408000	3.129115000	13.578313000
C	5.957899000	4.916793000	12.389825000
C	3.457507000	4.394739000	12.670694000
H	3.108832000	3.926483000	11.751720000
H	2.725405000	4.245223000	13.459873000
C	3.791904000	5.866787000	12.466198000
H	3.122225000	6.329737000	11.741546000
H	3.718996000	6.419750000	13.408988000
C	5.700129000	7.164007000	11.460063000
H	5.800362000	7.901458000	12.262380000
H	5.029925000	7.561139000	10.698715000
H	6.677061000	6.979452000	11.023416000
C	6.339409000	1.708406000	10.484248000
C	4.787298000	-0.344307000	10.613801000
H	4.804949000	-1.426225000	10.512865000
H	3.931792000	-0.048683000	11.219212000
C	4.800671000	0.367475000	9.268448000
H	5.383323000	-0.196350000	8.530020000
H	3.789844000	0.495548000	8.879708000
C	5.365486000	2.681967000	8.463710000
H	4.343145000	2.785250000	8.101351000
H	6.013731000	2.413656000	7.623386000
H	5.707855000	3.625930000	8.878707000
C	8.985204000	5.276542000	14.300803000
C	7.769080000	5.221982000	15.191629000
C	7.464870000	4.075818000	15.918801000
C	6.917476000	6.320940000	15.265271000
C	5.757859000	6.262550000	16.020262000
C	5.438758000	5.095515000	16.708421000
C	6.302581000	4.009285000	16.675266000
H	8.120389000	3.214902000	15.880716000
H	7.147697000	7.216305000	14.701959000
H	5.090594000	7.112833000	16.059216000
H	6.059883000	3.107054000	17.218249000

[L5a+Bn(*p*-CF_3)]$^+$
E [Eh]: −2495.488118661975

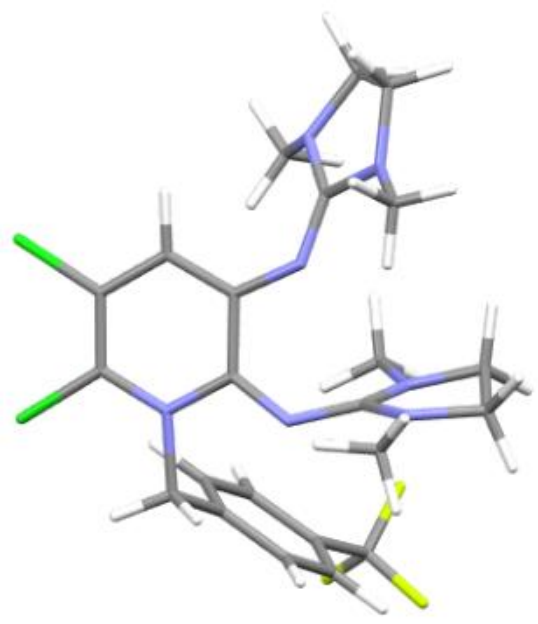

N	8.648875000	7.281324000	6.672091000
N	4.270415000	9.817189000	4.860776000
N	4.001133000	8.401042000	3.155299000
N	7.219173000	5.817638000	5.634248000
N	5.755663000	7.913582000	4.581239000
N	5.808041000	4.572212000	4.235271000
N	4.924716000	5.368175000	6.094730000
C	9.172741000	8.540727000	6.878621000
C	4.764237000	8.685013000	4.253813000
C	8.557790000	9.627597000	6.316237000
C	6.030049000	5.341226000	5.318998000
C	7.530745000	7.068082000	5.901792000
C	7.387559000	9.458519000	5.561605000
C	6.828208000	8.212762000	5.361843000
C	3.031449000	10.229532000	4.191269000
H	2.974182000	11.313633000	4.106277000
H	2.165953000	9.878125000	4.764970000
C	3.125605000	9.523929000	2.842588000
H	2.159396000	9.184703000	2.471251000
H	3.584580000	10.165241000	2.081024000
C	4.321515000	10.042703000	6.295024000
H	3.481989000	9.548349000	6.797455000
H	4.255744000	11.112742000	6.492103000
H	5.248766000	9.673995000	6.717776000
C	6.831945000	4.126162000	3.318649000
H	6.457211000	4.171718000	2.295356000
H	7.136440000	3.097291000	3.534111000
H	7.695508000	4.778737000	3.412293000
C	4.500925000	3.926960000	4.318784000
H	4.608072000	2.884166000	4.636414000
H	3.994491000	3.945508000	3.354730000
C	4.474474000	7.533817000	2.096480000
H	5.097072000	8.076864000	1.376686000
H	3.618095000	7.113141000	1.569660000
H	5.063239000	6.733101000	2.535612000
C	4.735837000	6.200967000	7.257389000
C	3.795376000	4.780560000	5.379049000
H	3.186426000	5.569770000	4.924254000
H	3.169784000	4.195490000	6.051011000
H	4.103814000	5.678050000	7.974521000
H	4.265952000	7.153523000	6.997495000
H	5.694888000	6.392426000	7.730842000
Cl	10.578657000	8.708528000	7.846120000
Cl	9.184835000	11.222422000	6.523634000
H	6.934446000	10.331556000	5.114622000
C	9.224616000	6.075707000	7.318988000
C	8.321209000	5.564737000	8.413506000
H	10.200295000	6.342245000	7.706596000

H	9.900876000	5.326744000	14.878609000
H	8.936162000	6.143825000	13.647304000
C	4.150448000	5.026748000	17.484558000
F	3.756886000	3.757850000	17.697058000
F	3.147544000	5.649626000	16.820488000
F	4.252970000	5.623197000	18.686129000

H	9.349158000	5.324094000	6.544408000
C	7.948324000	6.397556000	9.469538000
C	7.089461000	5.939835000	10.455912000
C	6.598282000	4.637485000	10.397305000
C	6.978916000	3.795448000	9.361087000
C	7.835022000	4.263630000	8.372364000
H	6.604315000	2.782716000	9.322190000
H	8.113278000	3.615057000	7.552233000
H	8.327546000	7.410212000	9.523525000
H	6.805012000	6.589274000	11.273050000
C	5.634436000	4.174785000	11.458464000
F	6.125185000	4.366883000	12.696204000
F	5.328198000	2.872152000	11.339176000
F	4.469974000	4.865596000	11.387912000

[L5b+Bn(p-CF_3)]$^+$
E [Eh]: −3102.505356226624

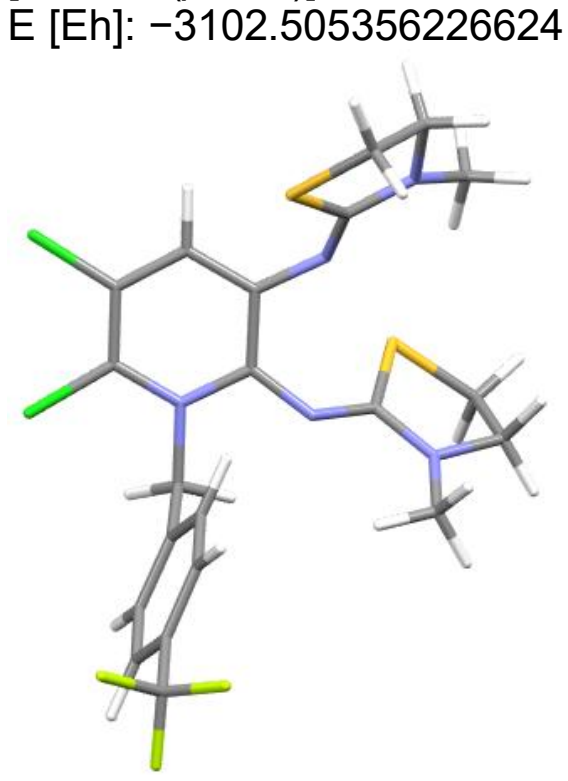

Cl	9.956438000	1.657093000	13.776195000
S	6.515609000	6.650846000	10.125293000
N	5.578490000	5.222733000	12.279881000
C	6.648098000	4.455037000	12.511266000
Cl	8.618281000	3.649815000	15.844382000
S	6.297233000	0.798304000	10.358798000
N	4.141613000	6.617115000	11.148674000
C	7.189148000	3.519222000	11.575628000
N	6.675159000	3.523256000	10.306328000
C	8.223194000	2.699062000	11.995316000
H	8.677608000	2.011098000	11.296753000
N	5.885741000	2.458157000	8.407033000
C	8.692957000	2.727306000	13.308223000
N	7.149264000	4.467112000	13.791014000
C	8.129436000	3.604720000	14.208388000
C	5.346115000	6.041861000	11.303988000
C	5.375829000	8.000344000	9.650185000
H	5.603504000	8.887526000	10.239030000
H	5.506171000	8.222086000	8.594214000
C	3.990194000	7.451481000	9.962785000
H	3.281205000	8.253300000	10.169442000
H	3.601500000	6.854752000	9.129756000
C	2.976282000	6.209302000	11.915151000
H	2.390653000	5.462887000	11.370893000
H	2.350258000	7.079756000	12.106817000
H	3.304337000	5.779534000	12.857632000
C	6.326717000	2.449900000	9.683726000
C	5.374461000	0.182493000	8.906207000
H	5.688987000	-0.834727000	8.688673000
H	4.310475000	0.193328000	9.136794000
C	5.721282000	1.151629000	7.785669000
H	6.646227000	0.854998000	7.277304000
H	4.923511000	1.197159000	7.043433000
C	5.987041000	3.638922000	7.568122000
H	6.016936000	4.519636000	8.202549000
H	5.122295000	3.686355000	6.905852000
H	6.896140000	3.610561000	6.958977000
C	6.567182000	5.432949000	14.752099000
C	5.468582000	4.843905000	15.602891000
H	6.182725000	6.255261000	14.156704000
H	7.374957000	5.809849000	15.371484000
C	4.673246000	3.790026000	15.160721000
C	5.210791000	5.416037000	16.845809000

[L6+Bn(p-CF_3)]$^+$
E [Eh]: −2495.494075396640

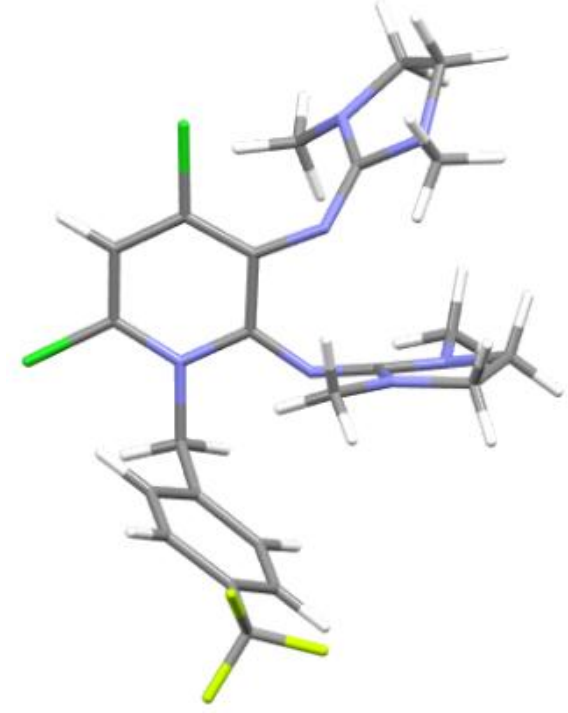

N	7.022934000	6.572419000	7.180340000
N	4.326512000	9.948096000	4.447816000
N	4.243839000	8.902335000	2.480247000
N	5.256542000	5.817232000	5.921973000
N	5.567220000	7.907764000	4.108575000
N	5.292206000	4.922971000	3.718710000
N	3.341235000	5.408211000	4.631204000
C	7.982214000	7.509789000	7.473326000
C	4.792947000	8.877109000	3.737040000
C	8.182443000	8.586826000	6.669158000
C	4.682419000	5.458479000	4.785085000
C	6.203300000	6.715573000	6.079410000
C	7.377573000	8.749258000	5.527643000
C	6.361630000	7.863528000	5.216257000
C	3.285662000	10.637437000	3.681206000
H	3.356635000	11.716418000	3.808213000
H	2.290907000	10.310367000	4.006369000
C	3.583426000	10.183880000	2.255208000
H	2.686405000	10.067785000	1.648351000
H	4.261301000	10.879898000	1.746455000
C	4.273607000	9.980397000	5.898357000
H	3.798866000	9.083173000	6.308104000
H	3.687922000	10.846890000	6.197455000
H	5.265664000	10.084405000	6.331502000
C	6.714804000	4.768114000	3.532459000
H	7.107149000	5.540898000	2.866875000
H	6.922713000	3.786384000	3.106233000
H	7.221881000	4.833213000	4.490170000
C	4.324714000	4.483328000	2.716804000
H	4.318510000	3.391782000	2.656962000
H	4.575750000	4.878689000	1.732233000
C	4.863244000	8.212614000	1.368545000
H	5.619140000	8.836211000	0.877880000
H	4.102717000	7.944330000	0.634792000
H	5.344109000	7.312524000	1.740715000
C	2.417033000	6.153851000	5.453992000
C	3.000792000	5.048264000	3.256566000
H	2.687859000	5.942964000	2.708594000
H	2.189305000	4.322112000	3.229247000
H	1.489034000	5.593488000	5.566170000
H	2.187633000	7.127918000	5.007117000
H	2.860440000	6.304975000	6.434713000
Cl	8.950290000	7.302634000	8.883122000

C	4.160978000	4.959000000	17.629886000
C	3.364634000	3.914321000	17.175583000
C	3.628471000	3.323774000	15.944672000
H	4.855377000	3.329535000	14.199888000
H	5.831687000	6.226551000	17.207596000
H	3.962274000	5.413653000	18.590058000
H	3.011694000	2.507790000	15.594017000
C	2.234732000	3.385321000	18.021201000
F	1.802382000	4.292711000	18.914045000
F	2.606709000	2.288447000	18.714111000
F	1.178668000	3.029138000	17.261670000

H	8.948053000	9.306141000	6.910320000
Cl	7.679420000	10.113981000	4.513080000
C	6.884268000	5.308181000	7.943104000
C	7.435391000	4.145406000	7.154860000
H	5.827049000	5.164528000	8.146440000
H	7.407425000	5.433932000	8.883962000
C	8.752987000	4.165069000	6.699539000
C	9.242215000	3.131631000	5.915699000
C	8.414167000	2.063127000	5.585148000
C	7.109697000	2.017661000	6.062888000
C	6.624122000	3.060579000	6.839512000
H	6.470240000	1.182817000	5.812319000
H	5.597664000	3.043266000	7.181402000
H	9.402218000	4.996199000	6.943073000
H	10.260461000	3.159548000	5.553439000
C	8.952630000	0.945485000	4.730650000
F	7.968803000	0.300778000	4.071061000
F	9.607113000	0.028157000	5.466760000
F	9.819950000	1.405710000	3.805956000

[L6+Bn(*o*-CF_3)]$^+$
E [Eh]: −2495.489703276026

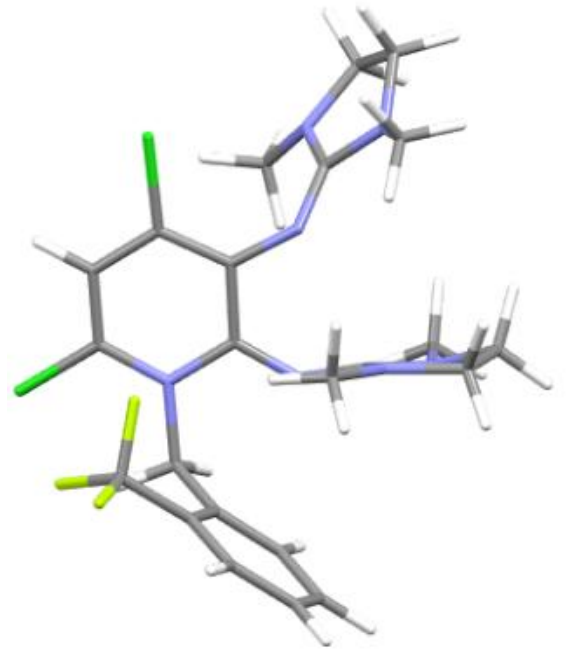

N	7.073603000	6.733988000	7.197181000
N	4.239846000	9.942590000	4.472784000
N	4.351129000	9.031414000	2.443449000
N	5.376767000	5.917135000	5.888040000
N	5.664459000	8.041169000	4.088028000
N	5.456753000	5.086458000	3.647447000
N	3.501894000	5.276425000	4.657465000
C	7.856156000	7.782231000	7.619497000
C	4.830758000	8.964197000	3.727110000
C	8.003961000	8.894871000	6.853099000
C	4.834557000	5.505129000	4.759692000
C	6.282440000	6.855331000	6.074103000
C	7.302751000	8.992808000	5.640479000
C	6.396466000	8.025904000	5.240677000
C	3.233184000	10.649232000	3.680484000
H	3.262790000	11.721408000	3.869473000
H	2.229933000	10.279319000	3.921604000
C	3.635915000	10.288822000	2.251710000
H	2.780813000	10.163235000	1.588641000
H	4.302102000	11.045991000	1.820673000
C	4.136023000	9.901761000	5.919656000
H	4.052912000	8.873866000	6.277508000
H	3.237474000	10.439554000	6.217783000
H	4.991855000	10.375323000	6.401854000
C	6.877332000	5.053778000	3.406744000
H	7.122596000	5.644401000	2.522217000
H	7.213552000	4.024992000	3.256768000
H	7.410504000	5.470625000	4.252196000
C	4.513195000	4.496564000	2.702922000
H	4.638847000	3.409915000	2.676249000
H	4.675554000	4.885897000	1.697655000
C	5.076981000	8.447254000	1.335940000
H	5.846432000	9.126516000	0.951047000
H	4.381946000	8.213282000	0.529371000
H	5.558214000	7.534932000	1.677129000
C	2.527916000	5.900322000	5.524260000
C	3.156039000	4.910306000	3.286849000
H	2.736446000	5.776245000	2.763329000

[L6+Bn(*o*,*p*-CF_3)$_2$]$^+$
E [Eh]: −2832.563691761876

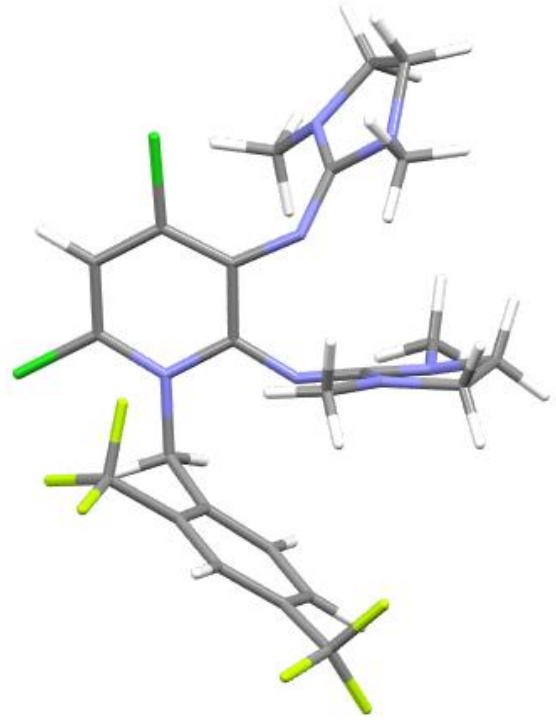

N	7.086598000	6.805367000	7.349816000
N	4.257438000	9.833760000	4.377649000
N	4.476735000	8.823863000	2.404879000
N	5.471061000	5.878197000	6.013414000
N	5.734311000	7.946399000	4.153818000
N	5.603670000	4.971493000	3.805612000
N	3.627021000	5.176628000	4.767730000
C	7.813572000	7.898552000	7.762220000
C	4.900794000	8.833554000	3.708974000
C	7.944956000	8.988564000	6.962454000
C	4.953544000	5.425972000	4.885951000
C	6.326661000	6.859695000	6.198758000
C	7.285363000	9.016856000	5.721789000
C	6.426353000	8.005750000	5.328151000
C	3.265675000	10.470167000	3.509815000
H	3.258870000	11.550892000	3.643745000
H	2.263852000	10.083924000	3.730160000
C	3.736412000	10.048756000	2.119374000
H	2.913102000	9.865235000	1.430106000
H	4.399896000	10.801122000	1.676121000
C	4.095407000	9.866338000	5.819301000
H	4.015938000	8.857308000	6.228253000
H	3.176454000	10.401606000	6.052359000
H	4.922381000	10.381587000	6.308857000
C	7.025895000	4.991718000	3.572409000
H	7.259251000	5.618385000	2.709668000
H	7.391151000	3.979155000	3.389748000
H	7.539370000	5.398134000	4.435495000
C	4.686406000	4.339546000	2.860617000
H	4.832019000	3.255667000	2.867042000
H	4.861619000	4.703700000	1.848230000
C	5.262522000	8.208016000	1.356886000
H	6.018951000	8.895338000	0.960952000
H	4.606493000	7.903086000	0.541323000
H	5.765182000	7.335352000	1.763692000
C	2.627036000	5.816802000	5.592322000
C	3.311757000	4.749152000	3.406730000
H	2.883967000	5.585243000	2.843167000

H	2.423109000	4.104832000	3.267380000
H	1.656709000	5.252926000	5.620124000
H	2.204086000	6.869959000	5.128407000
H	2.971463000	6.046016000	6.505685000
Cl	8.601619000	7.713191000	9.167914000
H	8.635460000	9.700619000	7.190377000
Cl	7.551425000	10.401447000	4.672362000
C	7.017289000	5.443880000	7.928379000
C	7.312128000	4.209978000	7.095457000
H	6.014373000	5.346067000	8.338899000
H	7.715169000	5.520184000	8.752929000
C	8.518591000	3.949405000	6.425303000
C	8.688362000	2.757680000	5.723415000
C	7.682193000	1.804356000	5.686181000
C	6.493285000	2.041985000	6.360625000
C	6.319807000	3.232348000	7.050038000
H	5.702065000	1.303951000	6.353024000
H	5.383206000	3.421866000	7.557008000
H	9.623066000	2.572209000	5.215349000
C	9.661018000	4.932830000	6.406833000
F	9.935322000	5.416672000	7.635477000
F	9.383699000	6.011319000	5.620572000
F	10.792351000	4.401068000	5.930506000
H	7.835140000	0.880124000	5.145505000

H	2.594979000	3.929141000	3.408533000
H	1.763506000	5.160358000	5.695424000
H	2.297249000	6.765689000	5.153809000
H	3.048134000	6.005633000	6.576319000
Cl	8.507483000	7.904909000	9.335496000
H	8.533109000	9.829625000	7.291973000
Cl	7.527728000	10.393501000	4.707489000
C	7.065245000	5.536205000	8.116334000
C	7.426634000	4.305039000	7.305799000
H	6.059513000	5.404839000	8.510388000
H	7.744898000	5.657623000	8.950567000
C	8.652858000	4.094794000	6.656252000
C	8.862475000	2.939755000	5.908639000
C	7.873177000	1.974110000	5.810253000
C	6.675247000	2.142093000	6.492183000
C	6.467604000	3.296471000	7.226623000
H	5.907369000	1.382529000	6.443204000
H	5.520949000	3.440045000	7.729010000
H	9.806068000	2.793603000	5.405290000
C	8.071164000	0.785191000	4.905668000
F	7.422735000	-0.302207000	5.349430000
F	9.365706000	0.468132000	4.759278000
F	7.585373000	1.052984000	3.664495000
C	9.774797000	5.105397000	6.686939000
F	9.979216000	5.598182000	7.923738000
F	9.505384000	6.168903000	5.882027000
F	10.936288000	4.593376000	6.266093000

Guanidino-/Benzyl salts

[L2a+Ha+BnA]$^+$
E [Eh]: −1239.295782303692

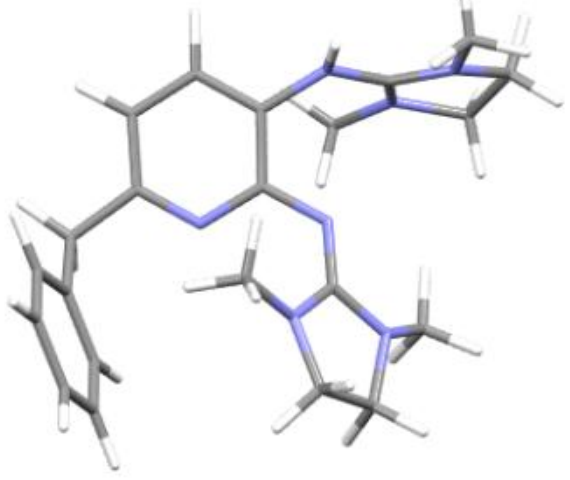

N	5.380469000	7.096386000	5.447775000
N	3.052250000	9.925919000	2.770857000
N	2.937714000	8.815473000	0.863341000
N	3.563747000	6.984662000	3.956668000
N	4.915517000	8.588053000	2.111469000
N	3.680081000	4.723055000	4.763392000
N	1.709647000	5.732270000	4.646244000
C	6.595695000	7.539652000	5.763361000
C	3.681441000	9.077000000	1.951330000
C	7.335159000	8.362821000	4.914595000
C	7.055728000	5.589140000	7.263764000
C	3.057486000	5.881035000	4.452984000
C	4.820817000	7.407769000	4.270433000
C	6.763493000	8.734897000	3.704375000
C	5.506423000	8.266974000	3.378512000
C	8.047769000	4.769657000	6.726893000
H	8.903309000	5.218182000	6.235185000
C	5.965786000	4.994367000	7.896054000
H	5.187308000	5.620866000	8.312278000
C	7.136164000	7.088330000	7.103227000
C	1.677153000	10.129411000	2.303378000
H	1.391457000	11.173375000	2.415275000
H	0.984926000	9.509358000	2.879663000
C	6.858889000	2.801618000	7.444895000
H	6.787257000	1.724504000	7.522493000
C	1.760379000	9.686300000	0.840590000
H	0.882035000	9.132775000	0.514254000
H	1.919238000	10.531113000	0.163229000
C	7.952243000	3.385726000	6.814823000
H	8.734805000	2.764165000	6.398938000
C	3.457687000	10.362340000	4.096363000
H	3.193170000	9.623024000	4.854302000
H	2.945479000	11.299406000	4.308058000
H	4.527642000	10.539615000	4.126806000
C	5.867197000	3.610988000	7.988326000
H	5.020767000	3.164080000	8.494878000
C	5.017216000	4.346602000	4.354519000
H	5.354456000	4.988409000	3.543111000
H	4.995345000	3.317058000	3.993405000
H	5.722361000	4.422072000	5.179360000
C	2.724976000	3.697959000	5.171745000
H	3.062638000	3.185926000	6.072498000
H	2.609559000	2.955610000	4.374682000
C	3.370534000	8.060342000	-0.292862000
H	4.008682000	8.648459000	-0.959742000
H	2.489593000	7.743957000	-0.847900000
H	3.898799000	7.159739000	0.021452000
C	0.832870000	6.867235000	4.814620000
C	1.443452000	4.505770000	5.391960000
H	0.552689000	3.999545000	5.020906000
H	1.291923000	4.736924000	6.452968000
H	7.289730000	9.384138000	3.015270000
H	8.320919000	8.706339000	5.193436000
H	-0.170336000	6.612295000	4.471331000
H	1.219648000	7.690590000	4.221372000
H	0.773903000	7.183209000	5.863466000
H	8.166714000	7.428069000	7.212806000
H	6.550262000	7.572074000	7.888821000
H	5.278559000	8.086441000	1.315149000

[L2a+Hb+BnA]$^+$
E [Eh]: −1239.290867581474

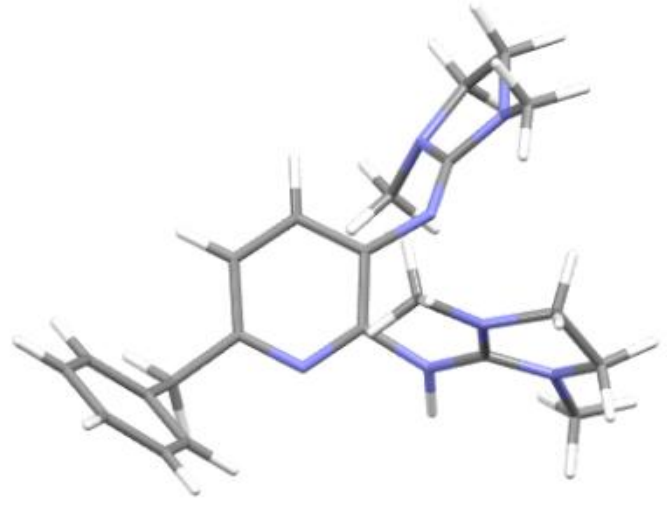

N	5.046911000	6.964494000	6.485763000
N	3.795427000	9.944892000	2.541646000
N	2.819022000	8.470794000	1.172324000
N	2.897577000	6.616105000	5.689704000
N	3.661834000	7.605285000	3.159676000
N	2.626907000	4.850765000	4.112785000
N	0.821642000	5.854640000	4.897107000
C	6.310168000	7.375394000	6.365433000
C	3.466130000	8.615035000	2.373120000
C	6.776979000	7.881086000	5.148912000
C	7.865003000	5.826026000	7.547860000
C	2.164594000	5.811006000	4.915575000
C	4.236032000	7.051857000	5.446329000
C	5.915914000	7.989284000	4.074677000
C	4.573876000	7.592469000	4.195928000
C	9.201453000	5.671544000	7.188476000
H	9.795072000	6.544865000	6.944624000
C	7.119214000	4.691699000	7.870595000
H	6.079784000	4.804059000	8.153610000
C	7.216232000	7.195536000	7.556008000
C	3.190901000	10.734359000	1.464159000
H	3.857847000	11.532879000	1.141912000
H	2.251078000	11.182151000	1.809120000
C	9.036135000	3.285780000	7.475078000
H	9.491261000	2.304146000	7.455233000
C	2.939693000	9.689914000	0.383876000
H	2.036300000	9.882528000	-0.194015000
H	3.786033000	9.616365000	-0.310555000
C	9.785773000	4.409653000	7.152341000
H	10.828177000	4.307018000	6.879329000
C	3.832280000	10.583939000	3.847212000
H	2.822500000	10.712962000	4.255816000
H	4.287408000	11.567542000	3.737383000
H	4.429084000	10.013783000	4.550024000
C	7.700104000	3.430853000	7.836360000
H	7.114424000	2.560022000	8.103079000
C	4.008849000	4.465940000	3.887168000
H	4.459319000	5.064558000	3.094782000
H	4.021500000	3.414651000	3.604788000
H	4.584415000	4.583349000	4.801373000
C	1.523631000	4.296572000	3.322769000
H	1.628371000	3.217328000	3.234469000
H	1.514483000	4.734752000	2.321191000
C	2.738984000	7.195052000	0.497880000
H	3.620730000	7.010714000	-0.127703000
H	1.851982000	7.171592000	-0.135694000
H	2.672521000	6.409797000	1.244988000
C	-0.007905000	6.691428000	5.736802000
C	0.297340000	4.713451000	4.143089000
H	-0.542456000	5.019876000	3.522496000
H	-0.034020000	3.922016000	4.822419000
H	6.257989000	8.394777000	3.131284000
H	7.811301000	8.183524000	5.052047000
H	-0.962657000	6.850809000	5.238400000
H	0.465969000	7.662933000	5.872395000
H	-0.197960000	6.237704000	6.714550000
H	7.988554000	7.965425000	7.549684000
H	6.623022000	7.322515000	8.462312000
H	2.490600000	6.926307000	6.558625000

[L2a+Hc+BnA]$^{+}$
E [Eh]: −1239.304851581702

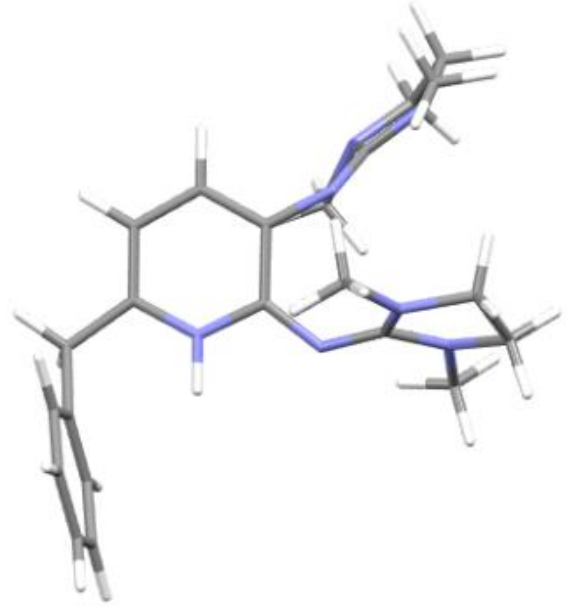

N	5.777170000	6.763951000	5.924496000
N	3.222134000	9.782537000	2.958970000
N	2.642386000	8.471494000	1.240240000
N	3.669793000	6.133113000	5.313402000
N	4.063800000	7.519292000	2.812055000
N	2.989029000	4.745588000	3.488259000
N	1.454530000	5.882997000	4.595227000
C	6.961574000	7.431556000	5.844593000
C	3.382746000	8.532328000	2.394435000
C	7.198792000	8.160941000	4.714040000
C	7.864418000	5.968347000	7.682909000
C	2.778690000	5.647494000	4.468824000
C	4.787613000	6.764227000	4.989601000
C	6.222695000	8.213739000	3.703862000
C	5.015496000	7.549089000	3.807437000
C	8.524090000	4.889754000	7.091934000
H	9.097027000	5.044049000	6.184986000
C	7.137091000	5.755046000	8.852690000
H	6.637255000	6.588777000	9.332737000
C	7.889071000	7.329128000	7.028213000
C	2.177377000	10.507392000	2.228954000
H	2.413326000	11.568082000	2.156929000
H	1.213014000	10.397083000	2.740378000
C	7.725013000	3.420247000	8.825984000
H	7.677651000	2.435083000	9.270844000
C	2.167161000	9.800771000	0.879586000
H	1.177164000	9.761106000	0.426406000
H	2.855450000	10.277562000	0.170332000
C	8.458412000	3.625573000	7.660651000
H	8.984301000	2.799862000	7.199161000
C	3.264654000	9.993566000	4.396189000
H	2.440656000	9.479099000	4.905377000
H	3.176661000	11.061802000	4.588309000
H	4.206515000	9.654738000	4.814760000
C	7.065837000	4.486705000	9.422362000
H	6.504870000	4.337722000	10.335690000
C	4.280192000	4.357893000	2.969969000
H	4.618307000	5.036355000	2.183677000
H	4.210509000	3.344727000	2.575333000
H	5.009963000	4.360510000	3.777135000
C	1.753402000	4.499895000	2.750592000
H	1.656394000	3.446256000	2.493333000
H	1.737156000	5.090644000	1.829113000
C	2.938299000	7.514841000	0.196461000
H	3.694818000	7.896324000	-0.499547000
H	2.030056000	7.293704000	-0.364774000
H	3.314595000	6.602950000	0.650531000
C	0.852464000	6.564597000	5.717944000
C	0.688791000	4.974656000	3.746710000
H	-0.139825000	5.492476000	3.266054000
H	0.286790000	4.146821000	4.341350000
H	6.408125000	8.798504000	2.811939000
H	8.130825000	8.696072000	4.607216000
H	0.058591000	7.226541000	5.369345000
H	1.616678000	7.149062000	6.223009000
H	0.425846000	5.850832000	6.429933000
H	8.894696000	7.579531000	6.689197000
H	7.606552000	8.091959000	7.760427000
H	5.609141000	6.187523000	6.742689000

[L2a+Ha+BnB]$^{+}$
E [Eh]: −1239.288137327354

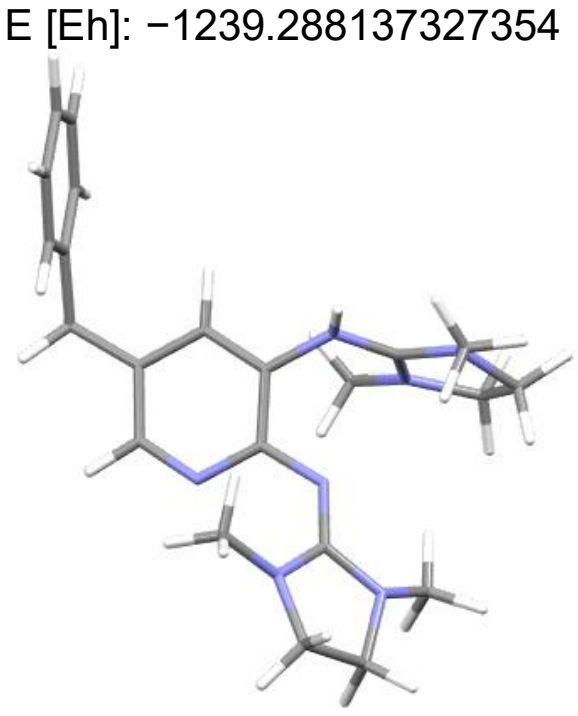

N	4.284107000	6.389858000	6.020480000
N	4.046648000	9.429393000	2.737629000
N	3.858331000	8.150103000	0.936650000
N	3.022505000	6.770215000	4.052284000
N	5.317644000	7.431118000	2.644038000
N	1.882749000	4.842421000	4.941689000
N	0.704520000	6.500981000	4.053256000
C	5.463058000	6.411600000	6.636478000
C	4.425604000	8.300498000	2.148931000
C	6.672512000	6.760225000	6.038229000
C	9.167986000	6.374980000	5.987281000
C	1.968257000	6.068862000	4.373315000
C	4.213458000	6.716252000	4.720130000
C	6.607458000	7.117552000	4.693735000
C	5.392033000	7.107029000	4.042250000
C	10.104741000	7.323448000	5.584675000
H	9.993243000	8.352834000	5.905421000
C	9.336200000	5.051740000	5.576157000
H	8.617241000	4.301816000	5.885590000
C	7.970488000	6.768985000	6.816715000
C	3.093840000	10.156335000	1.894015000
H	3.559296000	11.072125000	1.522306000
H	2.202538000	10.427334000	2.459242000
C	11.343717000	5.643452000	4.382994000
H	12.188042000	5.358486000	3.768962000
C	2.799354000	9.154434000	0.768778000
H	1.823279000	8.678712000	0.896276000
H	2.843888000	9.604583000	-0.221283000
C	11.187052000	6.962712000	4.787243000
H	11.910343000	7.711318000	4.490280000
C	4.591544000	10.016177000	3.949983000
H	4.068577000	9.647659000	4.833324000
H	4.470760000	11.096412000	3.886242000
H	5.650251000	9.789298000	4.041115000
C	10.414871000	4.686746000	4.782573000
H	10.537893000	3.654081000	4.481801000
C	2.975439000	3.900042000	5.063195000
H	3.780906000	4.165194000	4.380892000
H	2.612445000	2.905684000	4.798941000
H	3.374926000	3.883785000	6.076933000
C	0.501237000	4.368132000	4.968468000
H	0.253847000	3.922215000	5.931475000
H	0.349084000	3.613785000	4.188740000
C	3.742070000	6.864349000	0.268046000
H	4.723035000	6.420150000	0.100977000
H	3.297749000	7.029060000	-0.710910000
H	3.115699000	6.173504000	0.839747000
C	0.407720000	7.901887000	3.860717000
C	-0.292308000	5.644877000	4.688166000
H	-1.149022000	5.477717000	4.035894000
H	-0.649310000	6.106816000	5.616329000
H	7.504240000	7.394935000	4.153302000
H	-0.402267000	8.014614000	3.138668000
H	1.302196000	8.392411000	3.488084000
H	0.107224000	8.383428000	4.799197000
H	5.568711000	6.684824000	2.013046000
H	8.135596000	7.766084000	7.235403000
H	7.868165000	6.094467000	7.670149000
H	5.449548000	6.125085000	7.684422000

[L2a+Hb+BnB]+
E [Eh]: −1239.288518338323

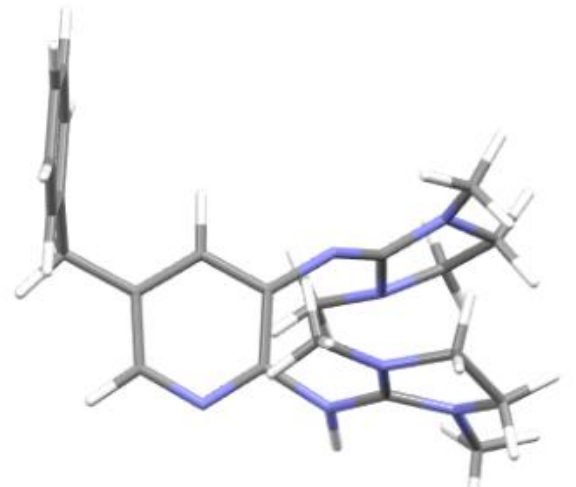

N	4.613155000	7.363660000	6.913196000
N	3.440056000	9.376547000	3.027604000
N	3.461555000	7.906438000	1.339524000
N	2.804818000	7.200901000	5.447150000
N	4.625646000	7.261600000	3.237965000
N	2.611794000	5.067938000	4.413755000
N	1.048853000	6.579585000	4.012837000
C	5.917703000	7.465095000	7.160713000
C	3.910173000	8.130211000	2.615541000
C	6.883270000	7.466665000	6.152470000
C	9.135662000	6.408964000	5.818574000
C	2.200986000	6.314505000	4.654543000
C	4.218149000	7.297439000	5.657703000
C	6.438948000	7.405480000	4.837728000
C	5.074419000	7.362104000	4.543066000
C	9.941113000	6.656000000	4.709825000
H	10.048173000	7.669805000	4.341717000
C	9.023365000	5.100435000	6.289684000
H	8.403860000	4.895908000	7.155480000
C	8.357905000	7.523740000	6.479175000
C	2.860658000	10.063670000	1.865818000
H	3.608769000	10.714475000	1.397998000
H	2.006721000	10.673252000	2.158633000
C	10.499440000	4.318502000	4.554158000
H	11.032187000	3.510674000	4.069775000
C	2.493072000	8.909883000	0.941864000
H	1.466131000	8.558430000	1.116182000
H	2.590371000	9.163701000	-0.113362000
C	10.619442000	5.617853000	4.078873000
H	11.247341000	5.827102000	3.222377000
C	4.213843000	10.231544000	3.920520000
H	4.459030000	9.721767000	4.845598000
H	3.610965000	11.106125000	4.162328000
H	5.144067000	10.570285000	3.450773000
C	9.700889000	4.062375000	5.664926000
H	9.613789000	3.053746000	6.048363000
C	3.824934000	4.418877000	4.882326000
H	4.059940000	4.746611000	5.891018000
H	4.667481000	4.632321000	4.222893000
H	3.642090000	3.345927000	4.901005000
C	1.752854000	4.450313000	3.398343000
H	1.537773000	3.418061000	3.666520000
H	2.246561000	4.467766000	2.423976000
C	3.497205000	6.612276000	0.707503000
H	4.287581000	6.023072000	1.164702000
H	3.702131000	6.724552000	-0.357645000
H	2.541227000	6.082964000	0.816190000
C	0.264186000	7.786699000	4.167244000
C	0.509060000	5.346144000	3.434622000
H	0.095879000	5.538030000	2.446122000
H	-0.278145000	4.933439000	4.072771000
H	7.143925000	7.397352000	4.016429000
H	-0.382302000	7.892917000	3.297906000
H	0.928008000	8.650443000	4.198280000
H	-0.362843000	7.763487000	5.063933000
H	8.759756000	8.487335000	6.155862000
H	8.482014000	7.480625000	7.562854000
H	6.207263000	7.528787000	8.203286000
H	2.242063000	7.950891000	5.817386000

[L2a+Hc+BnB]+
E [Eh]: −1239.298528972732

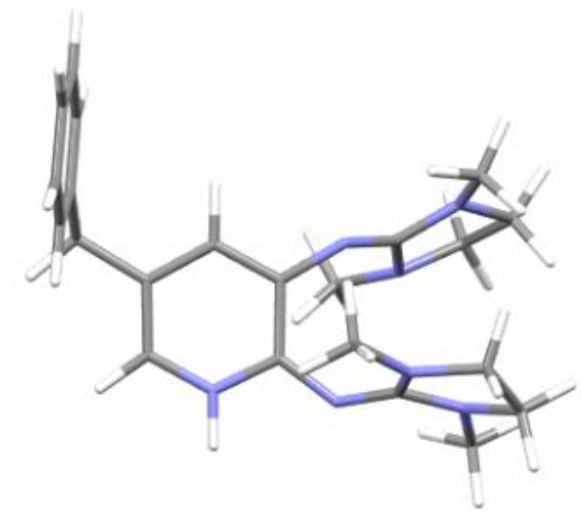

N	4.728094000	7.124201000	6.916946000
N	3.330096000	9.210432000	3.134783000
N	3.500515000	7.832726000	1.391168000
N	2.885916000	6.946403000	5.570495000
N	4.694603000	7.204442000	3.273529000
N	2.490218000	5.047225000	4.165868000
N	1.075442000	6.742376000	4.106979000
C	6.046619000	7.300256000	7.216241000
C	3.904856000	8.035890000	2.681963000
C	6.957232000	7.414935000	6.207591000
C	9.241021000	6.459929000	5.833330000
C	2.232360000	6.286407000	4.639037000
C	4.207078000	7.063010000	5.665352000
C	6.468975000	7.382834000	4.884815000
C	5.128427000	7.257346000	4.575984000
C	10.048583000	6.717545000	4.728903000
H	10.130773000	7.729620000	4.349952000
C	9.158001000	5.154080000	6.317078000
H	8.534823000	4.940453000	7.178134000
C	8.434296000	7.564116000	6.480385000
C	2.696435000	9.902514000	2.008859000
H	3.373291000	10.659879000	1.595628000
H	1.777224000	10.394605000	2.324453000
C	10.670410000	4.394356000	4.603957000
H	11.227610000	3.595704000	4.132165000
C	2.463507000	8.773119000	1.010844000
H	1.467011000	8.326031000	1.127107000
H	2.575395000	9.093000000	-0.024927000
C	10.759997000	5.691100000	4.115430000
H	11.388506000	5.907586000	3.261317000
C	3.946164000	10.057611000	4.141210000
H	4.132135000	9.510264000	5.058371000
H	3.254993000	10.867087000	4.372474000
H	4.887651000	10.495314000	3.790145000
C	9.868326000	4.127986000	5.709799000
H	9.802798000	3.121101000	6.101713000
C	3.694892000	4.280769000	4.392286000
H	4.068563000	4.469380000	5.396559000
H	4.475420000	4.529991000	3.670074000
H	3.453387000	3.221390000	4.314439000
C	1.514158000	4.664389000	3.149435000
H	1.189656000	3.634793000	3.294846000
H	1.948700000	4.752096000	2.149971000
C	3.711731000	6.606417000	0.665862000
H	4.502301000	6.044615000	1.155947000
H	4.010258000	6.826153000	-0.360375000
H	2.802240000	5.994921000	0.631587000
C	0.378785000	7.917857000	4.575884000
C	0.387962000	5.681157000	3.379136000
H	-0.038305000	6.057652000	2.449743000
H	-0.422637000	5.263511000	3.986103000
H	7.165714000	7.477477000	4.061530000
H	-0.078308000	8.433148000	3.730365000
H	1.095359000	8.579010000	5.053826000
H	-0.404980000	7.656894000	5.294188000
H	8.774891000	8.530834000	6.102255000
H	8.601161000	7.572941000	7.559426000
H	6.296780000	7.329626000	8.265710000
H	4.058911000	7.016216000	7.667756000

[L2a+Ha+BnC]$^+$
E [Eh]: −1239.296382241438

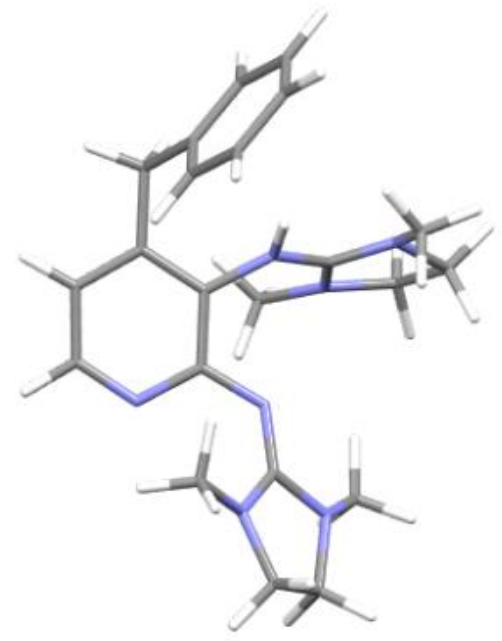

[L2a+Hb+BnC]$^+$
E [Eh]: −1239.295239931588

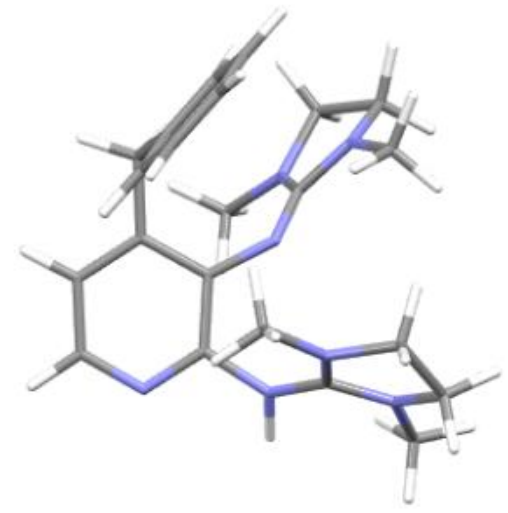

N	4.469011000	6.448109000	6.274975000	N	5.069752000	7.377091000	7.024908000
N	4.125380000	9.094794000	2.617619000	N	4.357828000	9.411844000	2.482936000
N	4.651847000	7.879009000	0.836345000	N	4.148220000	7.645537000	1.166040000
N	3.298429000	6.602109000	4.228611000	N	3.202184000	6.698851000	5.836403000
N	5.621735000	7.262199000	2.872163000	N	4.526612000	7.184702000	3.414323000
N	2.191482000	4.713031000	5.239558000	N	3.317576000	4.669146000	4.587030000
N	1.002251000	6.219187000	4.126138000	N	1.380448000	5.722501000	4.719769000
C	5.618063000	6.563889000	6.932220000	C	6.316144000	7.840922000	7.086324000
C	4.815815000	8.047638000	2.167167000	C	4.377835000	8.043054000	2.456161000
C	6.827983000	6.891283000	6.335511000	C	7.080197000	8.050711000	5.945095000
C	8.449849000	6.613186000	3.072303000	C	7.564016000	6.652655000	2.724615000
C	2.259864000	5.882165000	4.557999000	C	2.679414000	5.736407000	5.066641000
C	4.453864000	6.663353000	4.947167000	C	4.549285000	7.167471000	5.829279000
C	6.848419000	7.153574000	4.967705000	C	6.532111000	7.841235000	4.685648000
C	5.640872000	7.062595000	4.284483000	C	5.179554000	7.440843000	4.603088000
C	8.901302000	7.155859000	1.868993000	C	7.957992000	5.515892000	3.430633000
H	9.044197000	8.227012000	1.788037000	H	8.124233000	5.579897000	4.499215000
C	8.274443000	5.230511000	3.157395000	C	7.366194000	6.549768000	1.350690000
H	7.927531000	4.791830000	4.085049000	H	7.057982000	7.423835000	0.790871000
C	8.128813000	7.510584000	4.251651000	C	7.370601000	7.975771000	3.440156000
C	3.410925000	9.743548000	1.513035000	C	4.268864000	9.954003000	1.130494000
H	3.895555000	10.692798000	1.269103000	H	5.256148000	10.251376000	0.758852000
H	2.374477000	9.942320000	1.782678000	H	3.615107000	10.825200000	1.105400000
C	8.980346000	4.965925000	0.866610000	C	7.928641000	4.208272000	1.406747000
H	9.187971000	4.329117000	0.016810000	H	8.074925000	3.265130000	0.896671000
C	3.540804000	8.720562000	0.379378000	C	3.707150000	8.764772000	0.348680000
H	2.634587000	8.115772000	0.275420000	H	2.611813000	8.804952000	0.286577000
H	3.769021000	9.178895000	-0.581129000	H	4.108210000	8.695227000	-0.661697000
C	9.168179000	6.340642000	0.774355000	C	8.139259000	4.302699000	2.778448000
H	9.525194000	6.779450000	-0.148518000	H	8.454239000	3.432054000	3.339654000
C	4.266325000	9.743132000	3.911988000	C	4.613348000	10.268779000	3.617585000
H	3.626998000	9.269628000	4.655938000	H	4.062922000	11.199629000	3.479366000
H	3.975781000	10.785914000	3.798006000	H	5.674392000	10.510159000	3.736619000
H	5.298724000	9.708366000	4.252280000	H	4.264460000	9.798179000	4.532339000
C	8.536096000	4.413197000	2.063254000	C	7.546426000	5.338165000	0.693514000
H	8.400133000	3.342757000	2.148143000	H	7.391760000	5.277350000	-0.376293000
C	3.310318000	3.834611000	5.510574000	C	4.718152000	4.319142000	4.741667000
H	3.649757000	3.929259000	6.541773000	H	5.075477000	4.636385000	5.717544000
H	4.143834000	4.068403000	4.850942000	H	5.328931000	4.775407000	3.963206000
H	3.000410000	2.805554000	5.322271000	H	4.799817000	3.235102000	4.681054000
C	0.830464000	4.181396000	5.237005000	C	2.441206000	3.948391000	3.659846000
H	0.545286000	3.819645000	6.224599000	H	2.559720000	2.874642000	3.787965000
H	0.754393000	3.348873000	4.528855000	H	2.685034000	4.213493000	2.628183000
C	4.973317000	6.644109000	0.140409000	C	3.858960000	6.281061000	0.810801000
H	6.015766000	6.366994000	0.298909000	H	4.468999000	5.622582000	1.423302000
H	4.845010000	6.813165000	-0.926497000	H	4.112312000	6.119891000	-0.236688000
H	4.323762000	5.821122000	0.453273000	H	2.798309000	6.033639000	0.954099000
C	0.663422000	7.568060000	3.737651000	C	0.372270000	6.648922000	5.187568000
C	0.003858000	5.387470000	4.787765000	C	1.052611000	4.450837000	4.071414000
H	-0.803984000	5.117888000	4.107872000	H	0.393251000	4.615926000	3.221258000
H	-0.427496000	5.920445000	5.643591000	H	0.558758000	3.774223000	4.775643000
H	-0.139758000	7.546965000	2.999901000	H	-0.433951000	6.688482000	4.456784000
H	1.544478000	8.031114000	3.301927000	H	0.799496000	7.647389000	5.271704000
H	0.333031000	8.167897000	4.594620000	H	-0.048716000	6.349677000	6.152554000
H	5.573597000	6.374707000	7.999829000	H	6.717389000	8.027772000	8.074554000
H	8.068473000	8.543538000	3.896897000	H	6.924261000	8.687295000	2.743841000
H	8.949604000	7.481357000	4.971493000	H	8.343029000	8.388073000	3.718582000
H	7.734389000	6.947735000	6.922343000	H	8.109546000	8.372639000	6.038749000
H	6.221645000	6.648380000	2.337848000	H	2.607280000	7.112148000	6.538200000

[L2a+Hc+BnC]$^{+}$
E [Eh]: −1239.307308432465

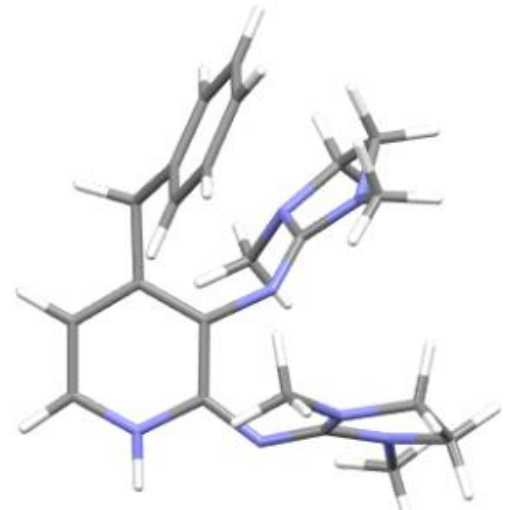

N	5.237497000	7.283315000	7.161899000
N	4.496232000	9.014888000	2.445339000
N	4.062918000	7.100478000	1.378617000
N	3.335997000	6.591983000	6.113119000
N	4.634383000	6.910707000	3.621371000
N	3.023975000	4.613723000	4.809457000
N	1.433160000	6.143027000	4.823017000
C	6.498877000	7.784124000	7.242117000
C	4.435199000	7.641128000	2.574971000
C	7.190463000	8.003192000	6.094038000
C	7.551416000	6.890274000	2.612467000
C	2.675567000	5.840358000	5.251199000
C	4.593255000	6.988673000	6.000835000
C	6.599517000	7.744496000	4.835099000
C	5.293899000	7.256852000	4.768663000
C	7.656491000	5.568975000	3.041612000
H	7.577559000	5.341238000	4.097446000
C	7.662305000	7.160468000	1.249864000
H	7.588401000	8.184977000	0.903996000
C	7.394113000	8.037113000	3.590095000
C	3.924319000	9.398654000	1.149886000
H	4.464374000	10.239567000	0.717697000
H	2.872511000	9.684184000	1.271857000
C	7.985571000	4.821711000	0.773577000
H	8.161707000	4.023044000	0.065100000
C	4.056146000	8.115920000	0.335390000
H	3.230520000	7.968389000	-0.360351000
H	4.995810000	8.086243000	-0.227949000
C	7.867910000	4.541515000	2.130642000
H	7.954071000	3.521003000	2.481707000
C	4.217598000	9.898065000	3.565749000
H	4.383090000	10.924711000	3.243301000
H	4.882963000	9.695732000	4.399363000
H	3.180614000	9.802444000	3.908922000
C	7.883667000	6.136819000	0.335447000
H	7.981280000	6.366949000	-0.717920000
C	4.333666000	4.019607000	4.939993000
H	4.775549000	4.322349000	5.887184000
H	4.998459000	4.320538000	4.126927000
H	4.229179000	2.935125000	4.943858000
C	2.041679000	4.120116000	3.848281000
H	1.851489000	3.058276000	3.997668000
H	2.399339000	4.273122000	2.825763000
C	4.296848000	5.714863000	1.037469000
H	4.452682000	5.153601000	1.953193000
H	5.187759000	5.609422000	0.413461000
H	3.436293000	5.310913000	0.501006000
C	0.647892000	7.253510000	5.309120000
C	0.821252000	4.996018000	4.159429000
H	0.282857000	5.303988000	3.264206000
H	0.118786000	4.495752000	4.834970000
H	0.136093000	7.739235000	4.477031000
H	1.311167000	7.966056000	5.791976000
H	-0.100667000	6.919626000	6.034471000
H	6.881221000	7.973652000	8.232685000
H	4.705941000	7.115944000	8.005451000
H	6.936813000	8.877325000	3.060807000
H	8.388346000	8.375376000	3.892008000
H	8.199053000	8.385987000	6.146683000

[L2a+BnD]$^{+}$
E [Eh]: −1239.277869283980

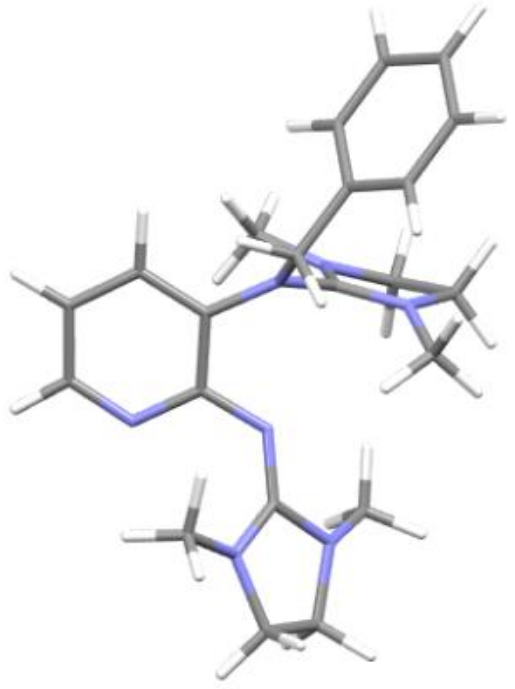

N	4.598266000	6.500764000	5.920168000
N	2.847977000	10.496040000	4.860618000
N	3.021366000	10.274706000	2.670323000
N	3.331276000	7.351461000	4.119174000
N	4.887080000	9.643053000	4.012256000
N	3.511403000	5.037336000	3.474309000
N	1.587156000	6.107842000	3.191499000
C	5.577288000	6.666280000	6.804569000
C	3.626725000	10.096690000	3.849833000
C	6.398160000	7.786574000	6.852429000
C	5.933291000	11.445024000	2.645219000
C	2.872147000	6.216843000	3.664436000
C	4.368334000	7.456055000	5.002741000
C	6.161813000	8.794843000	5.927912000
C	5.145110000	8.642259000	5.002987000
C	5.750412000	12.449658000	3.595536000
H	5.597815000	12.186607000	4.634879000
C	6.144913000	11.803937000	1.317042000
H	6.302298000	11.033934000	0.570868000
C	5.949838000	9.991103000	3.044869000
C	1.512498000	10.846625000	4.375100000
H	1.191589000	11.787364000	4.818985000
H	0.788959000	10.072150000	4.641662000
C	5.975998000	14.136104000	1.890521000
H	5.992529000	15.177918000	1.599141000
C	1.737170000	10.960322000	2.859477000
H	0.960760000	10.460281000	2.283675000
H	1.806089000	11.997925000	2.526858000
C	5.768137000	13.786217000	3.221088000
H	5.629183000	14.556818000	3.968230000
C	3.203478000	10.574834000	6.262826000
H	3.007792000	9.633480000	6.780368000
H	2.613213000	11.365681000	6.722812000
H	4.256338000	10.819623000	6.367840000
C	6.169537000	13.142181000	0.939883000
H	6.339378000	13.406344000	-0.095633000
C	4.947680000	4.853412000	3.469108000
H	5.451651000	5.806232000	3.318436000
H	5.212744000	4.189288000	2.645214000
H	5.297368000	4.424032000	4.407805000
C	2.648311000	4.072618000	2.797014000
H	2.714024000	3.088668000	3.260649000
H	2.942015000	3.980226000	1.745550000
C	3.342047000	9.560183000	1.448242000
H	3.961938000	10.158548000	0.780016000
H	2.410817000	9.319456000	0.937803000
H	3.839368000	8.626312000	1.698609000
C	0.541325000	7.002624000	3.630914000
C	1.264724000	4.705294000	2.948254000
H	0.644528000	4.586878000	2.060303000
H	0.724962000	4.287212000	3.806287000
H	6.760213000	9.698214000	5.929704000
H	7.182198000	7.870915000	7.590933000
H	-0.231697000	7.066896000	2.864403000
H	0.974526000	7.984816000	3.793582000
H	0.080011000	6.661725000	4.565676000
H	6.883615000	9.751909000	3.552627000
H	5.907356000	9.353148000	2.160359000
H	5.715092000	5.857687000	7.514663000

[L2a+BnE]$^+$
E [Eh]: −1239.281425309414

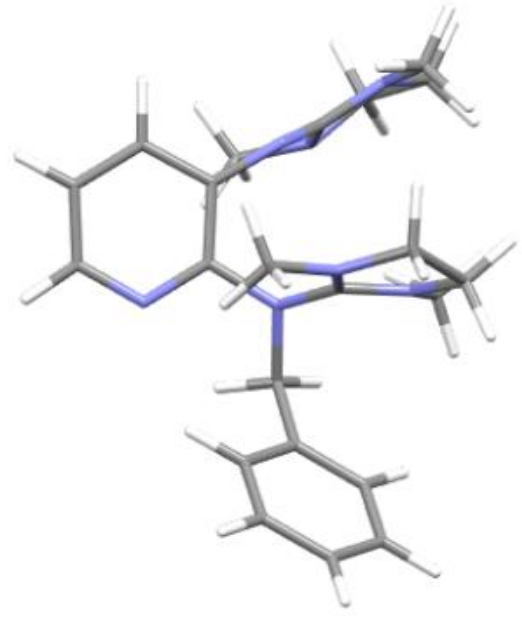

N	5.688391000	6.777001000	6.507392000
N	2.626220000	9.460763000	4.215625000
N	2.661374000	8.922554000	2.051107000
N	3.749161000	6.177236000	5.335823000
N	4.409065000	8.072092000	3.313963000
N	4.279431000	4.979754000	3.334827000
N	2.169666000	5.487969000	3.713038000
C	6.786853000	7.516697000	6.662230000
C	3.335216000	8.777907000	3.237176000
C	7.182444000	8.455789000	5.719516000
C	3.169648000	4.154957000	6.646451000
C	3.427434000	5.590492000	4.156659000
C	4.933615000	6.966238000	5.439528000
C	6.386199000	8.651829000	4.601517000
C	5.202864000	7.926863000	4.442455000
C	2.025045000	3.376848000	6.500109000
H	1.059873000	3.856057000	6.385139000
C	4.402859000	3.525196000	6.823649000
H	5.290367000	4.131583000	6.956852000
C	3.094722000	5.656587000	6.569723000
C	1.598288000	10.285155000	3.572080000
H	1.971001000	11.303194000	3.407449000
H	0.702550000	10.339763000	4.189382000
C	3.336660000	1.368086000	6.689726000
H	3.402260000	0.288234000	6.711514000
C	1.375154000	9.563635000	2.248034000
H	0.566669000	8.823037000	2.318937000
H	1.140561000	10.243220000	1.429004000
C	2.105125000	1.987792000	6.523100000
H	1.206911000	1.393157000	6.418607000
C	3.239021000	10.022595000	5.410075000
H	2.444177000	10.419775000	6.040264000
H	3.933068000	10.836232000	5.172861000
H	3.769709000	9.264324000	5.975083000
C	4.485121000	2.140201000	6.844595000
H	5.443406000	1.659543000	6.993519000
C	5.711108000	4.825637000	3.496983000
H	6.249055000	5.644400000	3.014488000
H	6.006866000	3.881091000	3.043288000
H	5.965177000	4.796823000	4.551740000
C	3.591949000	4.561868000	2.110304000
H	3.907806000	3.559752000	1.828517000
H	3.822727000	5.251492000	1.295169000
C	2.936322000	8.128139000	0.882790000
H	2.774986000	8.724093000	-0.016082000
H	2.286820000	7.243939000	0.824896000
H	3.974206000	7.807400000	0.916331000
C	1.019264000	6.209398000	4.210565000
C	2.116574000	4.621960000	2.531881000
H	1.479053000	5.065010000	1.768693000
H	1.718029000	3.639995000	2.797960000
H	6.649052000	9.381332000	3.846718000
H	8.090908000	9.024963000	5.861097000
H	0.418034000	6.535714000	3.361744000
H	1.353217000	7.091036000	4.752344000
H	0.395633000	5.587021000	4.855713000
H	7.365730000	7.339186000	7.559988000
H	3.637592000	6.118476000	7.389159000
H	2.059763000	5.992887000	6.608455000

[L2a+Bn(*p*-CF_3)E]$^+$
E [Eh]: −1576.356621869617

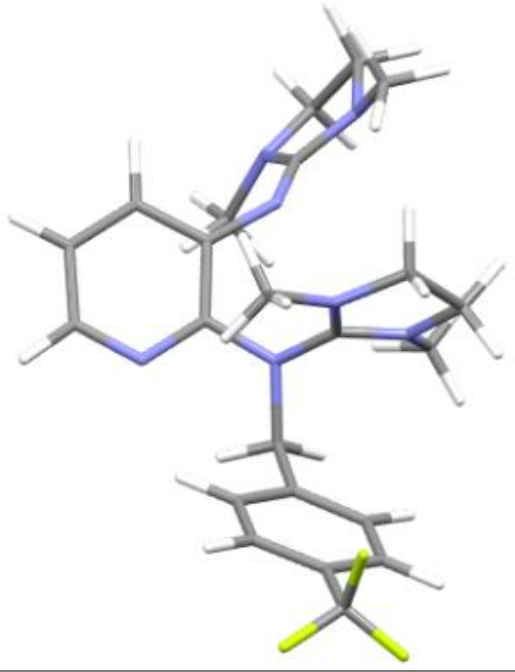

N	7.269510000	6.076267000	6.405784000
N	4.362915000	9.873345000	4.853632000
N	3.794309000	9.241722000	2.784169000
N	5.225395000	5.513635000	5.403896000
N	5.305375000	7.861259000	3.890872000
N	5.485036000	4.903190000	3.108266000
N	3.440153000	5.129096000	3.899042000
C	8.288265000	6.915354000	6.602018000
C	4.565703000	8.922335000	3.877479000
C	8.400131000	8.113738000	5.910596000
C	4.745051000	5.223877000	4.164259000
C	6.324278000	6.408910000	5.545846000
C	7.405294000	8.459183000	5.008312000
C	6.307595000	7.614914000	4.811800000
C	3.288402000	10.778900000	4.438616000
H	3.499887000	11.803253000	4.742033000
H	2.339668000	10.469400000	4.894370000
C	3.262185000	10.592431000	2.927341000
H	2.261711000	10.674760000	2.504312000
H	3.913659000	11.315633000	2.421231000
C	4.439222000	9.586936000	6.276080000
H	3.556522000	9.032263000	6.618732000
H	4.480306000	10.531018000	6.818103000
H	5.330937000	9.020666000	6.517840000
C	6.930161000	4.822906000	3.038770000
H	7.354440000	5.764223000	2.682473000
H	7.199406000	4.021262000	2.352625000
H	7.335818000	4.591161000	4.018268000
C	4.642736000	4.730107000	1.923086000
H	4.919984000	3.815350000	1.402216000
H	4.770019000	5.573057000	1.240604000
C	4.130713000	8.761578000	1.461474000
H	4.844056000	9.426491000	0.960161000
H	3.228521000	8.697840000	0.852406000
H	4.581139000	7.777741000	1.551793000
C	2.373984000	5.681876000	4.704629000
C	3.227400000	4.661407000	2.525861000
H	2.524042000	5.317584000	2.014899000
H	2.822987000	3.647392000	2.526558000
H	1.750654000	4.896397000	5.137218000
H	1.748937000	6.324192000	4.082459000
H	2.805287000	6.282994000	5.500473000
H	9.248535000	8.763512000	6.076046000
H	7.452121000	9.390429000	4.458741000
C	4.877107000	4.571518000	6.500844000
C	5.208551000	3.150401000	6.128524000
C	6.535906000	2.719481000	6.111042000
C	6.851143000	1.437605000	5.689976000
C	5.839012000	0.572359000	5.280363000
C	4.513437000	0.985696000	5.307553000
C	4.204379000	2.271990000	5.732342000
H	3.728449000	0.305326000	5.008513000
H	3.168761000	2.588469000	5.763447000
H	7.315522000	3.399942000	6.428764000
H	7.879496000	1.102868000	5.687502000
H	9.033141000	6.609500000	7.325854000
H	5.456869000	4.901096000	7.357731000
H	3.818179000	4.666613000	6.740091000
C	6.193681000	-0.795586000	4.758657000
F	5.172664000	-1.661912000	4.873426000
F	7.251029000	-1.321513000	5.400355000

F	6.518120000	-0.739130000	3.444134000

[L3+Ha+BnA]+
E [Eh]: −1698.858728704860

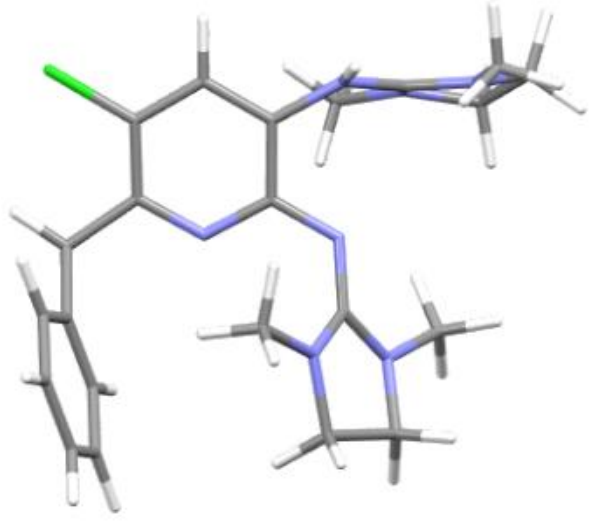

N	6.384044000	6.378650000	5.880369000
N	4.017896000	10.022354000	4.641458000
N	4.274925000	10.062890000	2.446739000
N	4.754354000	6.960006000	4.290144000
N	6.115080000	9.351214000	3.725460000
N	5.060720000	4.645978000	3.698010000
N	3.034614000	5.371742000	4.181003000
C	7.495948000	6.628703000	6.568319000
C	4.847856000	9.769483000	3.625527000
C	8.217592000	7.808285000	6.355408000
C	4.346808000	5.724494000	4.094197000
C	5.916093000	7.221146000	4.949415000
C	7.744345000	8.727678000	5.426964000
C	6.592609000	8.442835000	4.725049000
C	2.683342000	10.327929000	4.115453000
H	2.225765000	11.130112000	4.690537000
H	2.044790000	9.442060000	4.167703000
C	2.988502000	10.727566000	2.669613000
H	2.244344000	10.364235000	1.963540000
H	3.089367000	11.811049000	2.553378000
C	4.222563000	9.755345000	6.054880000
H	3.997955000	8.715758000	6.298468000
H	3.560160000	10.412152000	6.615955000
H	5.246712000	9.977006000	6.337656000
C	6.489595000	4.574485000	3.494572000
H	6.864405000	5.550917000	3.196207000
H	6.688989000	3.867644000	2.688538000
H	7.014772000	4.253988000	4.393377000
C	4.228674000	3.446615000	3.696839000
H	4.388383000	2.870985000	4.613900000
H	4.464612000	2.813638000	2.842504000
C	4.942363000	10.067225000	1.162062000
H	5.557551000	10.960458000	1.015570000
H	4.185987000	10.037276000	0.380376000
H	5.558967000	9.174363000	1.054456000
C	1.959431000	6.313642000	4.348166000
C	2.818183000	4.040214000	3.629274000
H	2.450126000	4.101114000	2.597508000
H	2.095003000	3.483035000	4.223185000
H	1.150797000	5.852913000	4.916519000
H	1.558161000	6.653042000	3.384726000
H	2.336118000	7.172202000	4.897607000
Cl	9.679624000	8.177296000	7.226592000
H	8.274382000	9.655823000	5.259066000
C	7.902279000	5.571908000	7.570454000
C	7.950818000	4.198084000	6.947235000
C	9.074016000	3.798142000	6.223770000
C	9.114387000	2.553510000	5.606227000
C	8.028819000	1.690003000	5.707226000
C	6.906489000	2.079018000	6.430388000
C	6.868531000	3.326312000	7.043158000
H	8.063608000	0.715531000	5.237699000
H	6.064854000	1.404586000	6.528220000
H	5.991033000	3.629726000	7.599998000
H	9.924899000	4.465054000	6.146991000
H	9.996729000	2.253680000	5.055600000

[L3+Hb+BnA]+
E [Eh]: −1698.863660491942

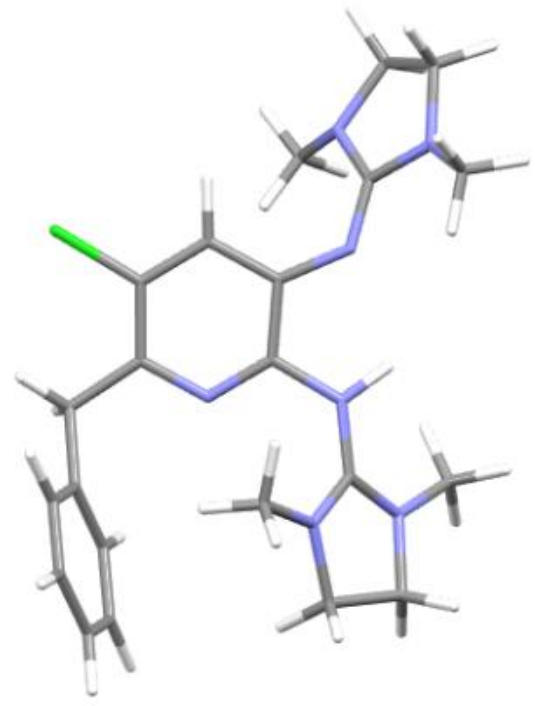

N	6.224063000	6.083844000	5.623192000
N	4.587653000	11.011943000	4.755874000
N	4.183864000	10.753718000	2.576714000
N	4.430517000	6.231589000	4.123589000
N	4.874238000	8.844163000	3.706689000
N	5.053759000	3.955798000	3.808871000
N	2.922652000	4.420485000	4.152029000
C	7.253177000	6.605430000	6.300193000
C	4.586793000	10.115633000	3.711287000
C	7.551861000	7.958803000	6.130342000
C	4.165106000	4.917138000	4.036370000
C	5.512833000	6.828653000	4.811487000
C	6.784356000	8.758096000	5.292931000
C	5.696016000	8.212748000	4.605779000
C	4.000910000	12.280879000	4.311022000
H	4.537026000	13.130360000	4.731737000
H	2.954170000	12.337089000	4.631736000
C	4.111065000	12.192735000	2.791834000
H	3.256351000	12.630721000	2.277104000
H	5.022522000	12.676753000	2.421438000
C	4.285451000	10.620132000	6.122751000
H	3.205007000	10.499871000	6.265686000
H	4.639830000	11.397345000	6.799295000
H	4.781493000	9.692396000	6.383440000
C	6.455062000	4.103046000	3.461310000
H	6.627589000	5.097312000	3.058991000
H	6.692013000	3.365787000	2.694379000
H	7.092034000	3.950762000	4.328210000
C	4.417083000	2.642856000	3.955700000
H	4.673760000	2.214254000	4.926869000
H	4.760866000	1.969685000	3.173279000
C	4.444484000	10.234882000	1.251189000
H	5.378559000	10.638294000	0.844239000
H	3.626760000	10.507532000	0.583521000
H	4.522670000	9.153256000	1.308072000
C	1.710199000	5.181806000	4.359446000
C	2.925497000	2.988789000	3.835418000
H	2.536902000	2.817936000	2.827016000
H	2.307430000	2.443712000	4.546346000
H	1.020499000	4.594390000	4.964425000
H	1.219612000	5.431832000	3.414114000
H	1.939765000	6.098804000	4.899616000
Cl	8.878298000	8.700670000	6.983001000
H	7.031885000	9.803310000	5.175029000
C	8.002259000	5.696248000	7.248843000
C	8.077832000	4.267033000	6.777486000
C	9.143938000	3.844982000	5.983628000
C	9.212214000	2.536655000	5.517196000
C	8.210245000	1.628914000	5.841299000
C	7.146384000	2.037762000	6.639442000
C	7.082378000	3.346930000	7.102021000
H	8.269124000	0.605868000	5.492913000
H	6.377534000	1.328420000	6.920887000
H	6.254775000	3.659583000	7.726557000
H	9.930669000	4.546527000	5.732329000
H	10.052903000	2.224038000	4.911309000

H	8.866156000	5.823691000	8.006292000	H	9.005386000	6.090248000	7.402243000
H	7.168249000	5.583603000	8.380020000	H	7.506955000	5.737702000	8.224655000
H	6.646597000	9.388246000	2.868894000	H	3.763806000	6.902857000	3.763826000

[L3+Hc+BnA]$^{+}$
E [Eh]: −1698.865745659530

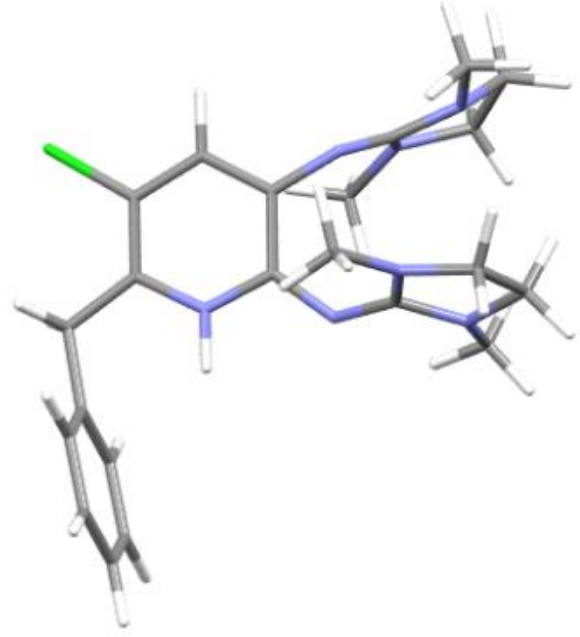

[L3+Ha+BnC]$^{+}$
E [Eh]: −1698.860233980676

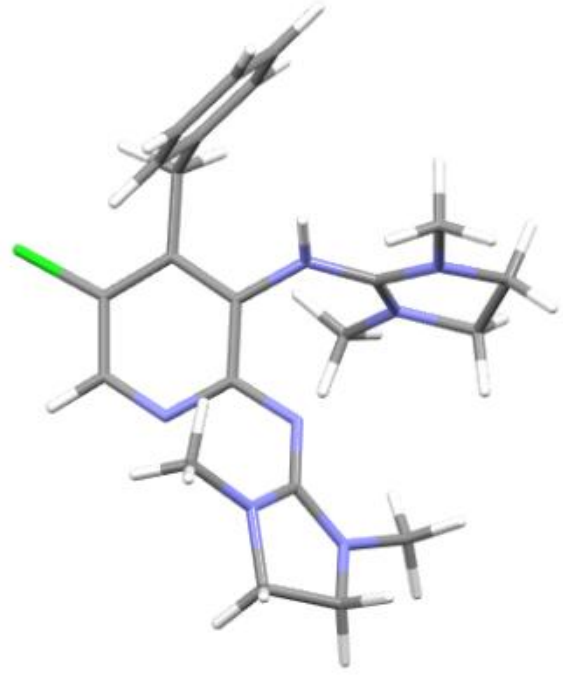

N	7.091476000	5.969951000	5.873813000	N	4.151302000	5.376840000	7.011822000
N	3.939445000	9.384426000	4.929162000	N	5.076355000	9.183633000	4.673900000
N	3.614526000	9.225955000	2.729232000	N	6.054850000	8.413012000	2.847667000
N	5.093319000	5.724361000	4.787896000	N	3.795514000	6.398257000	4.920697000
N	5.585842000	8.295742000	3.522360000	N	6.456433000	7.246985000	4.841672000
N	4.843841000	5.612141000	2.410616000	N	2.770652000	4.269279000	4.444962000
N	3.039678000	5.572896000	3.682064000	N	1.623110000	6.120746000	4.096129000
C	8.237563000	6.574891000	6.288356000	C	4.954447000	5.120147000	8.034141000
C	4.479080000	8.910097000	3.757958000	C	5.860932000	8.229149000	4.165024000
C	8.511465000	7.802868000	5.741070000	C	6.279251000	5.537665000	8.090879000
C	4.387956000	5.683546000	3.674719000	C	2.806231000	5.621933000	4.550576000
C	6.185210000	6.455454000	4.985397000	C	4.616242000	6.076688000	5.961483000
C	7.622858000	8.386115000	4.824108000	C	6.808766000	6.284486000	7.036542000
C	6.447590000	7.763290000	4.451054000	C	5.943706000	6.561181000	5.980548000
C	2.598052000	9.910411000	4.690005000	C	4.502797000	9.974458000	3.581444000
H	2.417033000	10.799481000	5.292853000	H	4.455044000	11.025471000	3.858261000
H	1.833806000	9.163483000	4.938729000	H	3.496245000	9.619567000	3.345282000
C	2.633130000	10.199416000	3.193573000	C	5.485344000	9.698912000	2.438802000
H	1.669713000	10.054300000	2.706598000	H	4.995732000	9.609625000	1.471060000
H	2.974085000	11.223204000	2.994123000	H	6.266066000	10.463471000	2.373143000
C	4.288238000	8.944075000	6.264922000	C	4.617218000	9.313600000	6.044296000
H	3.884026000	7.952982000	6.495266000	H	3.742828000	8.689792000	6.232281000
H	3.878384000	9.659124000	6.977128000	H	4.366639000	10.358854000	6.216384000
H	5.365504000	8.926476000	6.395845000	H	5.410483000	9.032765000	6.731646000
C	6.228737000	5.503295000	2.014013000	C	3.800692000	3.341407000	4.844940000
H	6.639407000	6.475275000	1.734178000	H	3.750704000	2.466823000	4.195481000
H	6.303020000	4.822580000	1.165604000	H	3.689685000	3.028539000	5.885572000
H	6.818731000	5.092355000	2.831109000	H	4.778741000	3.802238000	4.725150000
C	3.751302000	5.426531000	1.458949000	C	1.430768000	3.813143000	4.089011000
H	3.785086000	4.415064000	1.043890000	H	0.883416000	3.488319000	4.980318000
H	3.823507000	6.138085000	0.636552000	H	1.478617000	2.981839000	3.386750000
C	4.078592000	9.287898000	1.359648000	C	6.988048000	7.671073000	2.025186000
H	4.561250000	10.246440000	1.135471000	H	8.011617000	8.045608000	2.119176000
H	3.231454000	9.158676000	0.685574000	H	6.677848000	7.755472000	0.985299000
H	4.800174000	8.492769000	1.195365000	H	6.967379000	6.615907000	2.296020000
C	2.210683000	5.833494000	4.834005000	C	1.378488000	7.516955000	3.850182000
C	2.505164000	5.657029000	2.327214000	C	0.818905000	5.076120000	3.476864000
H	2.075314000	6.649833000	2.160872000	H	0.927898000	5.093705000	2.385302000
H	1.730768000	4.908621000	2.161021000	H	-0.235321000	5.196969000	3.722365000
H	1.388116000	5.118452000	4.868284000	H	0.330585000	7.747022000	4.045526000
H	1.791439000	6.844628000	4.799956000	H	1.607051000	7.796854000	2.813630000
H	2.813651000	5.723727000	5.731446000	H	2.001192000	8.101781000	4.521397000
Cl	9.943781000	8.661275000	6.202222000	Cl	7.235828000	5.099823000	9.477699000
H	7.845201000	9.362720000	4.416332000	H	8.245113000	6.737778000	6.975515000
C	9.092703000	5.860483000	7.305420000	C	9.008630000	6.102407000	5.829025000
C	8.718739000	4.413757000	7.505522000	C	9.796847000	6.879946000	4.979916000
C	9.266159000	3.424404000	6.687309000	C	10.487419000	6.300563000	3.919823000
C	8.894455000	2.094877000	6.840400000	C	10.395491000	4.932477000	3.693962000
C	7.965438000	1.737920000	7.812153000	C	9.614731000	4.147795000	4.536968000
C	7.413675000	2.714932000	8.631628000	C	8.925999000	4.728518000	5.594605000
C	7.788459000	4.045696000	8.478038000	H	10.935558000	4.478458000	2.873626000
H	7.680852000	0.701413000	7.936003000	H	9.548309000	3.079730000	4.374965000
H	6.700160000	2.441867000	9.397973000	H	8.323129000	4.110036000	6.248158000
H	7.365836000	4.802431000	9.129064000	H	9.877020000	7.946559000	5.156327000
H	9.996389000	3.696685000	5.934278000	H	11.103987000	6.916927000	3.278070000

H	9.336171000	1.336124000	6.207743000	H	8.273812000	7.824205000	6.861960000
H	10.132849000	5.947282000	6.987716000	H	8.735830000	6.505450000	7.919518000
H	9.023948000	6.404952000	8.251843000	H	7.279223000	6.839616000	4.415118000
H	6.891321000	5.049683000	6.255689000	H	4.538713000	4.545002000	8.853695000

$[L3+Hb+BnC]^+$
E [Eh]: −1698.862133630842

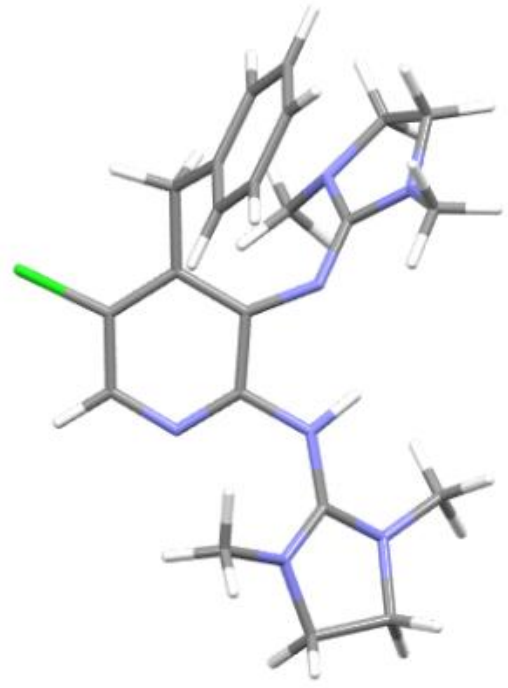

$[L3+Hc+BnC]^+$
E [Eh]: −1698.861919822699

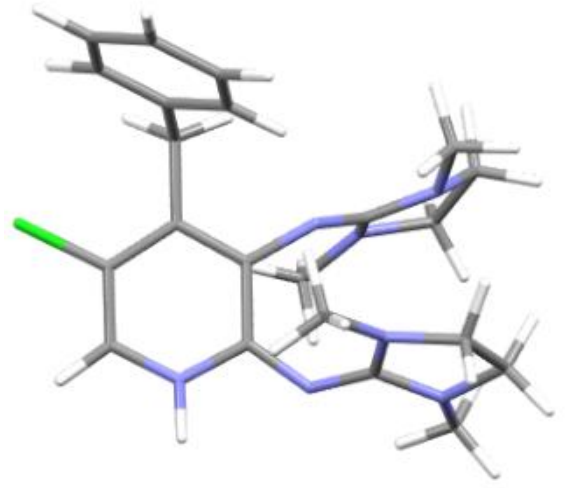

N	4.024812000	4.972521000	6.977551000	N	4.583088000	5.840663000	7.977343000
N	6.210138000	9.317822000	5.040199000	N	4.696198000	9.136152000	4.629970000
N	6.555903000	8.533743000	2.981532000	N	4.862792000	7.956270000	2.748710000
N	3.448759000	5.641715000	4.798365000	N	3.283894000	6.018854000	6.106403000
N	5.642903000	7.030665000	4.493771000	N	5.621637000	6.899802000	4.669893000
N	2.195927000	3.609262000	4.931401000	N	3.650555000	4.699430000	4.140234000
N	1.275171000	5.370558000	3.965448000	N	1.950863000	6.111308000	4.192936000
C	4.889963000	4.986715000	7.992087000	C	5.715070000	5.940420000	8.717082000
C	6.116127000	8.212202000	4.227337000	C	5.086884000	7.929994000	4.109828000
C	6.098315000	5.661624000	7.907914000	C	6.866727000	6.336225000	8.104837000
C	2.361738000	4.888389000	4.594505000	C	3.024552000	5.643398000	4.871099000
C	4.347155000	5.633486000	5.890270000	C	4.493703000	6.123133000	6.652179000
C	6.453555000	6.393879000	6.765773000	C	6.875257000	6.686097000	6.732033000
C	5.508521000	6.424089000	5.715423000	C	5.680618000	6.619407000	6.013992000
C	6.574589000	10.486271000	4.231896000	C	4.069876000	9.958352000	3.599188000
H	7.277466000	11.126175000	4.763694000	H	4.355307000	11.003707000	3.711520000
H	5.678273000	11.073162000	3.998959000	H	2.976711000	9.891775000	3.654525000
C	7.169316000	9.854623000	2.975521000	C	4.609277000	9.327171000	2.318937000
H	6.922908000	10.404010000	2.067249000	H	3.897620000	9.358195000	1.495003000
H	8.258186000	9.761845000	3.042516000	H	5.538409000	9.813253000	1.996340000
C	5.295608000	9.545880000	6.144891000	C	4.440992000	9.426338000	6.025294000
H	4.298647000	9.831749000	5.787774000	H	3.462969000	9.059906000	6.353605000
H	5.685121000	10.355487000	6.760588000	H	4.470663000	10.506621000	6.162443000
H	5.205857000	8.660975000	6.766777000	H	5.211167000	8.989892000	6.653543000
C	3.193312000	2.671941000	5.415715000	C	4.669590000	3.794636000	4.627772000
H	3.017392000	1.712752000	4.928565000	H	5.671903000	4.154507000	4.392870000
H	3.136770000	2.557311000	6.496736000	H	4.522901000	2.817887000	4.167281000
H	4.187048000	3.021318000	5.152620000	H	4.583576000	3.675202000	5.706577000
C	0.812038000	3.199831000	4.660252000	C	2.942838000	4.450153000	2.885509000
H	0.233073000	3.190412000	5.587085000	H	2.430399000	3.484584000	2.931329000
H	0.794602000	2.203048000	4.224773000	H	3.632321000	4.434703000	2.041883000
C	6.962638000	7.539028000	2.010483000	C	5.566508000	7.053269000	1.862864000
H	8.044133000	7.384116000	2.041314000	H	6.585991000	7.397169000	1.652871000
H	6.674281000	7.863562000	1.009848000	H	5.023055000	6.977860000	0.921009000
H	6.468437000	6.601363000	2.246656000	H	5.622107000	6.073383000	2.328397000
C	1.135947000	6.695957000	3.401825000	C	1.213033000	7.295206000	4.563751000
C	0.331994000	4.284413000	3.687345000	C	1.957431000	5.624432000	2.818043000
H	0.404939000	3.969648000	2.641746000	H	2.314236000	6.413623000	2.148134000
H	-0.687987000	4.607297000	3.886504000	H	0.960008000	5.321635000	2.501071000
H	0.086035000	6.983556000	3.430618000	H	0.149498000	7.141743000	4.379144000
H	1.481116000	6.736949000	2.364233000	H	1.545199000	8.164321000	3.986322000
H	1.699669000	7.411398000	3.998601000	H	1.365939000	7.486694000	5.622369000
Cl	7.156595000	5.561327000	9.289136000	Cl	8.298700000	6.439092000	9.073983000
C	7.781000000	7.092509000	6.652602000	C	8.139053000	7.135628000	6.045344000
C	8.605837000	6.742933000	5.431314000	C	8.954509000	5.955715000	5.544815000
C	9.433918000	7.709493000	4.863158000	C	10.149649000	5.599753000	6.164110000
C	10.267726000	7.397606000	3.795428000	C	10.887979000	4.513625000	5.706509000
C	10.277753000	6.109340000	3.273666000	C	10.438014000	3.769500000	4.623539000
C	9.449550000	5.140059000	3.828848000	C	9.248006000	4.122027000	3.994858000
C	8.622189000	5.455318000	4.899414000	C	8.511973000	5.207192000	4.451584000
H	10.928981000	5.861410000	2.445764000	H	11.013812000	2.926091000	4.265691000
H	9.455126000	4.132857000	3.432119000	H	8.899712000	3.555434000	3.140260000
H	7.986728000	4.690968000	5.329080000	H	7.594215000	5.495287000	3.956505000
H	9.436049000	8.712776000	5.273145000	H	10.509227000	6.175542000	7.006605000

H	10.914478000	8.158052000	3.376148000	H	11.817433000	4.252624000	6.195678000
H	7.629855000	8.173624000	6.666790000	H	7.867109000	7.775754000	5.207745000
H	8.368081000	6.859656000	7.539904000	H	8.738057000	7.724117000	6.738427000
H	3.692538000	6.355917000	4.119353000	H	3.728668000	5.522208000	8.415307000
H	4.620032000	4.446238000	8.889109000	H	5.647199000	5.690252000	9.763384000

$[L3+BnD]^+$
E [Eh]: −1698.840801286901

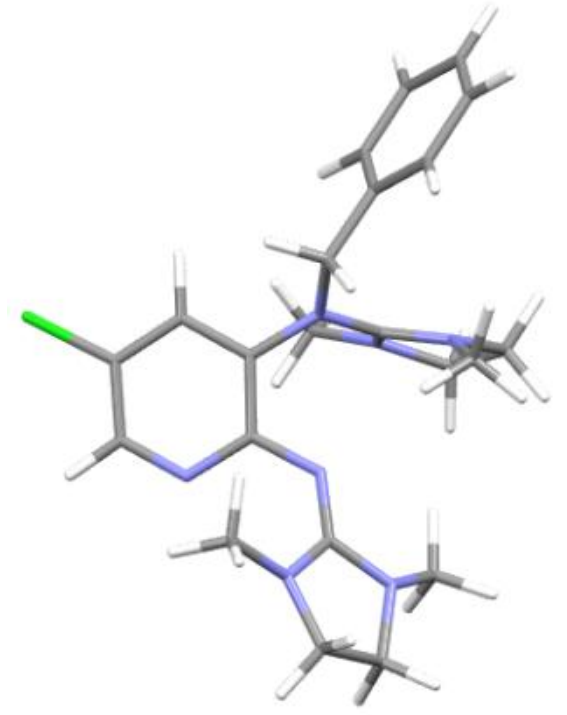

N	7.121707000	7.037106000	4.814479000
N	4.784476000	10.797441000	4.166756000
N	4.333376000	10.468718000	2.029696000
N	5.319236000	7.656807000	3.418235000
N	6.556497000	10.057024000	2.781002000
N	5.500019000	5.307158000	2.908794000
N	3.494539000	6.237011000	3.107027000
C	8.292942000	7.322992000	5.368291000
C	5.267336000	10.404423000	2.984226000
C	8.980590000	8.504457000	5.112504000
C	4.846142000	6.460455000	3.188825000
C	6.547957000	7.901751000	3.959551000
C	8.402605000	9.433395000	4.259135000
C	7.179629000	9.140876000	3.683959000
C	3.339923000	11.022178000	4.084179000
H	3.082960000	11.962551000	4.568515000
H	2.794671000	10.215515000	4.580139000
C	3.101755000	11.061268000	2.566288000
H	2.241696000	10.467019000	2.263482000
H	2.972968000	12.078754000	2.192202000
C	5.527751000	10.992460000	5.395502000
H	5.596849000	10.069229000	5.974220000
H	5.017715000	11.749330000	5.988870000
H	6.531300000	11.343588000	5.172860000
C	6.886484000	5.205649000	2.502183000
H	7.254104000	6.171817000	2.162325000
H	6.957543000	4.499621000	1.673489000
H	7.517954000	4.865370000	3.322614000
C	4.561084000	4.240427000	2.567593000
H	4.831137000	3.304435000	3.055695000
H	4.557340000	4.077588000	1.484246000
C	4.354178000	9.717998000	0.787149000
H	4.713521000	10.322880000	-0.045714000
H	3.339204000	9.390872000	0.567284000
H	4.974470000	8.834008000	0.914401000
C	2.539140000	7.114788000	3.741852000
C	3.228552000	4.802757000	3.062701000
H	2.396990000	4.571937000	2.397731000
H	2.984483000	4.431737000	4.065247000
H	1.588058000	7.067180000	3.210389000
H	2.926515000	8.128331000	3.703366000
H	2.367988000	6.841812000	4.790252000
Cl	10.515119000	8.817424000	5.858110000
H	8.901251000	10.372301000	4.056665000
C	7.260292000	10.444926000	1.538668000
C	7.002510000	11.875728000	1.138817000
C	7.020219000	12.899200000	2.086465000
C	6.810459000	14.217190000	1.704521000
C	6.586661000	14.529252000	0.367139000
C	6.578954000	13.517309000	-0.584102000
C	6.782680000	12.197018000	-0.197493000
H	6.426125000	15.556794000	0.068968000

$[L3+BnE]^+$
E [Eh]: −1698.845483538770

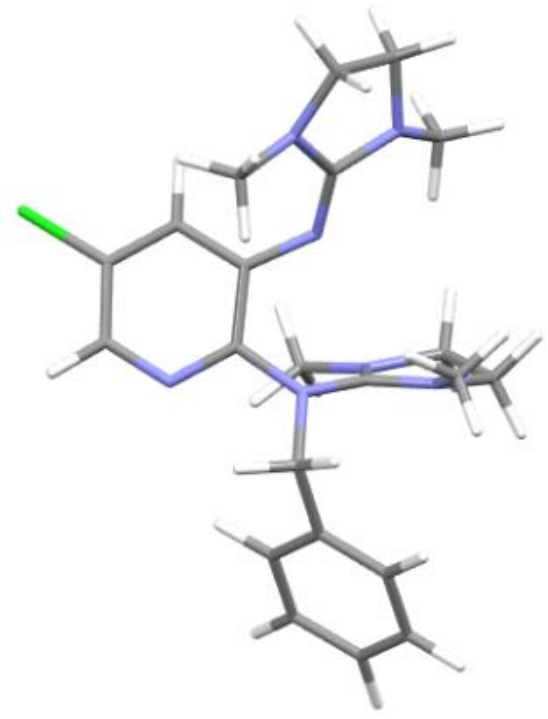

H	4.323197000	1.806088000	8.079595000
H	6.063064000	3.127490000	11.771855000
N	2.287115000	5.071641000	10.572415000
N	2.626201000	6.575431000	8.750898000
N	0.558576000	6.194870000	9.414749000
N	2.202357000	3.503298000	8.266772000
N	0.824969000	2.741727000	6.545243000
N	1.548777000	1.196752000	7.978818000
N	4.308445000	4.059756000	11.191872000
C	3.332765000	4.141946000	10.307416000
C	3.242475000	3.328805000	9.153441000
C	4.307774000	2.441039000	8.954183000
C	5.323373000	2.362051000	9.891799000
Cl	6.629390000	1.242029000	9.670929000
C	5.290044000	3.178828000	11.016779000
C	1.847926000	5.896365000	9.591658000
C	1.814451000	7.312132000	7.780126000
H	1.864470000	6.835301000	6.798418000
H	2.182109000	8.332600000	7.688505000
C	0.409095000	7.246123000	8.404503000
H	0.117820000	8.187621000	8.874677000
H	-0.356398000	6.963933000	7.683175000
C	4.074302000	6.592215000	8.711626000
H	4.473896000	6.366994000	9.695051000
H	4.401937000	7.590172000	8.423695000
H	4.454901000	5.865184000	7.990673000
C	-0.559088000	5.419044000	9.905454000
H	-1.264166000	5.256368000	9.089663000
H	-1.078116000	5.927249000	10.720558000
H	-0.198087000	4.452218000	10.246392000
C	1.597611000	2.527185000	7.659302000
C	0.481861000	1.468087000	5.922208000
H	1.187103000	1.238703000	5.113917000
H	-0.524833000	1.485615000	5.506156000
C	0.628161000	0.491153000	7.085750000
H	-0.328178000	0.322547000	7.593280000
H	1.031289000	-0.473728000	6.780610000
C	0.999177000	3.908892000	5.711121000
H	1.296771000	4.742031000	6.340124000
H	0.058949000	4.146011000	5.212414000
H	1.769938000	3.752106000	4.946885000
C	1.766099000	0.668282000	9.315623000
H	2.715634000	0.135961000	9.392479000
H	0.958733000	-0.024796000	9.556136000
H	1.760779000	1.469144000	10.051234000
C	1.900611000	5.385269000	11.976173000
H	2.544652000	4.759784000	12.588099000
H	0.866330000	5.089936000	12.152285000
C	2.099640000	6.842256000	12.297502000
C	3.388628000	7.375333000	12.353002000
H	4.239320000	6.719140000	12.212886000
C	3.577257000	8.727049000	12.604315000

H	6.414310000	13.753265000	-1.627207000	H	4.579996000	9.131020000	12.657724000
H	6.781235000	11.413126000	-0.945831000	C	2.479813000	9.560840000	12.804050000
H	7.204641000	12.666619000	3.127925000	C	1.194951000	9.035392000	12.759957000
H	6.831019000	15.003405000	2.447969000	H	0.339686000	9.676401000	12.930280000
H	8.712084000	6.586219000	6.044141000	C	1.008162000	7.680272000	12.505108000
H	8.318067000	10.298703000	1.755180000	H	0.005096000	7.270425000	12.484492000
H	7.017316000	9.768766000	0.717306000	H	2.628638000	10.613569000	13.005489000

[L3+Bn(p-CF_3)E]$^+$
E [Eh]: −2035.920098134157

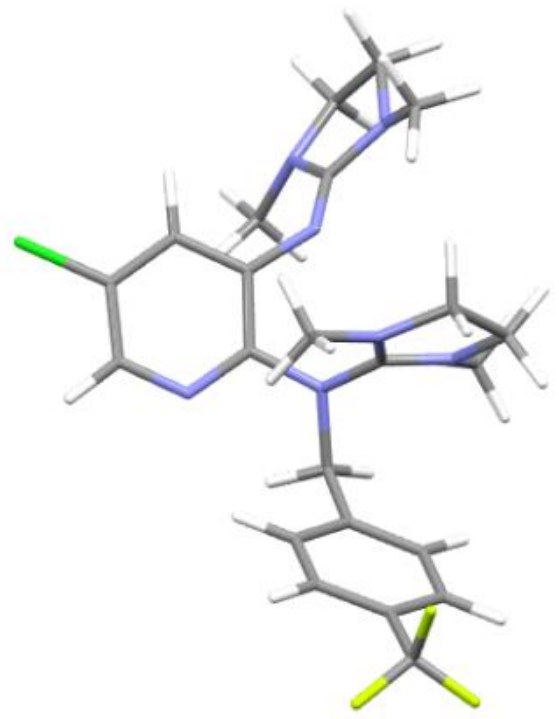

N	7.236073000	6.060984000	6.424344000
N	4.391040000	9.972734000	4.844039000
N	3.826650000	9.302733000	2.788051000
N	5.207880000	5.466094000	5.409193000
N	5.219985000	7.869227000	3.979025000
N	5.516648000	4.914011000	3.105311000
N	3.456568000	5.024575000	3.882314000
C	8.234113000	6.909738000	6.657376000
C	4.551759000	8.978686000	3.905821000
C	8.301374000	8.133534000	6.000234000
C	4.753219000	5.176474000	4.161891000
C	6.277314000	6.390594000	5.579230000
C	7.302254000	8.489511000	5.109081000
C	6.226862000	7.621361000	4.885900000
C	3.374827000	10.922140000	4.378929000
H	3.640964000	11.943218000	4.647608000
H	2.404929000	10.682774000	4.831238000
C	3.356859000	10.680930000	2.874704000
H	2.365936000	10.793610000	2.436453000
H	4.047160000	11.352292000	2.349628000
C	4.423075000	9.725046000	6.276411000
H	3.530519000	9.179992000	6.606956000
H	4.449795000	10.684752000	6.790426000
H	5.306847000	9.166635000	6.562695000
C	6.964743000	4.888210000	3.049656000
H	7.360026000	5.854706000	2.729414000
H	7.269401000	4.120427000	2.340089000
H	7.369594000	4.637713000	4.024922000
C	4.692117000	4.708263000	1.912473000
H	5.022490000	3.814900000	1.385507000
H	4.777130000	5.562246000	1.236992000
C	4.136410000	8.742714000	1.491093000
H	4.878946000	9.347958000	0.958103000
H	3.231008000	8.691295000	0.885588000
H	4.538772000	7.744121000	1.631891000
C	2.357301000	5.512168000	4.685905000
C	3.279257000	4.555232000	2.504657000
H	2.542816000	5.173491000	1.993254000
H	2.933505000	3.519729000	2.496064000
H	1.801157000	4.693498000	5.147699000
H	1.676770000	6.082160000	4.052269000
H	2.745656000	6.170898000	5.458389000
Cl	9.629415000	9.205851000	6.300845000
H	7.336478000	9.442700000	4.600093000
C	4.858449000	4.516314000	6.500028000
C	5.178419000	3.096674000	6.112234000
C	6.505909000	2.669886000	6.045833000
C	6.809658000	1.389260000	5.613482000
C	5.785651000	0.520558000	5.241183000
C	4.460949000	0.929397000	5.317661000

C	4.163598000	2.215108000	5.753639000
H	3.667607000	0.246883000	5.047186000
H	3.128734000	2.527589000	5.823143000
H	7.295234000	3.350825000	6.338049000
H	7.838326000	1.057609000	5.573606000
H	8.993841000	6.611833000	7.367663000
H	5.448100000	4.833366000	7.355096000
H	3.803086000	4.616706000	6.751944000
C	6.128448000	-0.846181000	4.707704000
F	5.100521000	-1.704808000	4.814162000
F	7.180874000	-1.386292000	5.345945000
F	6.454876000	-0.780352000	3.394213000

[L4+Ha+BnB]$^+$
E [Eh]: −1698.859518964089

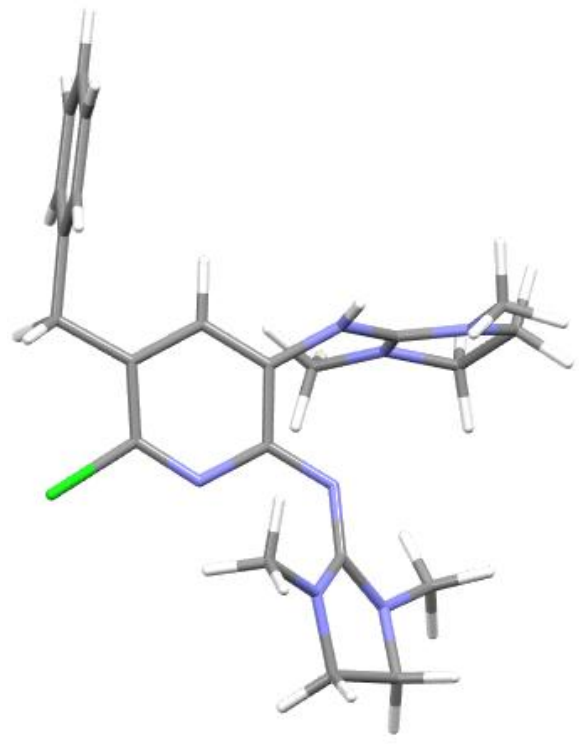

N	4.522320000	5.156329000	6.109767000
N	5.386694000	9.258509000	4.378969000
N	5.345761000	8.891566000	2.199730000
N	3.636371000	6.579172000	4.450881000
N	6.223786000	7.184186000	3.556914000
N	2.084297000	4.740836000	4.253979000
N	1.309165000	6.791772000	4.501401000
C	5.563548000	4.645044000	6.723750000
C	5.655883000	8.384505000	3.404857000
C	6.906424000	4.896403000	6.410806000
C	2.441031000	6.036584000	4.431646000
C	4.696413000	5.986700000	5.064179000
C	7.092746000	5.774646000	5.348764000
C	6.011831000	6.319217000	4.680277000
C	4.657739000	10.403211000	3.823227000
H	5.008220000	11.328752000	4.275357000
H	3.586139000	10.299439000	4.012069000
C	4.987252000	10.305763000	2.330857000
H	4.138365000	10.544688000	1.693186000
H	5.831073000	10.944568000	2.052452000
C	5.501003000	9.062412000	5.814400000
H	4.640485000	8.521999000	6.211850000
H	5.558372000	10.045029000	6.279045000
H	6.408960000	8.516240000	6.050801000
C	2.961914000	3.598247000	4.156453000
H	3.905319000	3.895141000	3.704089000
H	2.490774000	2.855300000	3.512079000
H	3.168809000	3.156354000	5.133280000
C	0.640499000	4.577538000	4.398782000
H	0.392620000	4.222293000	5.404493000
H	0.257515000	3.862695000	3.671757000
C	5.645707000	8.278172000	0.923619000
H	6.689174000	8.422564000	0.626633000
H	5.006032000	8.726433000	0.165837000
H	5.417288000	7.212529000	0.955792000
C	1.300305000	8.229797000	4.442648000
C	0.133800000	6.002134000	4.158818000
H	-0.152269000	6.162124000	3.111730000
H	-0.711974000	6.262928000	4.793396000
H	0.462789000	8.615682000	5.024559000
H	1.207613000	8.596936000	3.412586000
H	2.229929000	8.597705000	4.867927000
Cl	5.196586000	3.551377000	8.038842000
H	8.096754000	6.038285000	5.042807000
C	8.052134000	4.263009000	7.170014000

[L4+Hb+BnB]$^+$
E [Eh]: −1698.856713564654

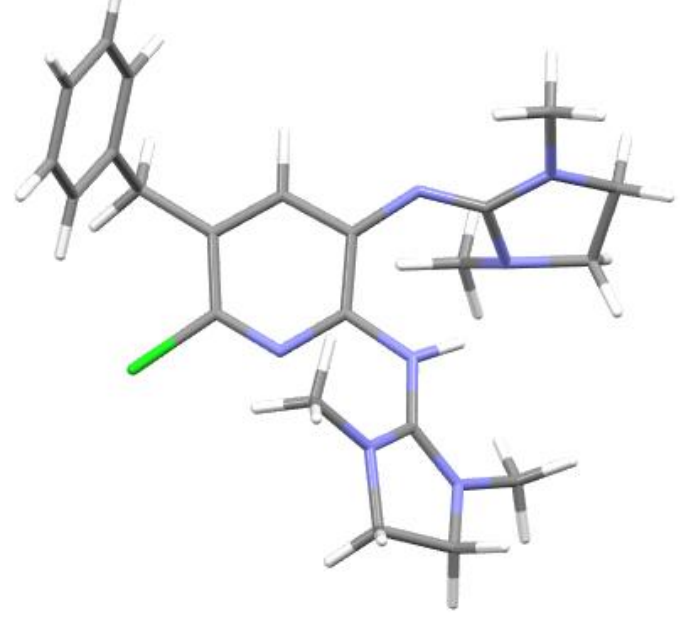

N	4.851155000	5.559252000	6.230692000
N	4.324087000	9.496476000	4.323649000
N	5.502093000	9.633038000	2.402158000
N	3.755895000	6.720619000	4.531290000
N	6.269518000	8.085620000	3.950476000
N	2.885581000	4.534680000	4.144663000
N	1.464163000	6.207315000	4.388431000
C	5.917591000	5.263521000	6.943982000
C	5.436863000	8.996254000	3.600108000
C	7.162729000	5.879256000	6.769147000
C	2.757787000	5.841788000	4.375292000
C	4.940877000	6.467547000	5.273597000
C	7.220704000	6.844608000	5.773141000
C	6.114619000	7.189095000	4.986917000
C	3.683941000	10.551763000	3.503642000
H	3.476345000	11.430925000	4.110200000
H	2.739117000	10.183480000	3.100071000
C	4.689316000	10.836073000	2.382155000
H	4.205468000	10.964220000	1.413917000
H	5.296087000	11.727939000	2.579595000
C	4.540491000	9.843799000	5.735077000
H	5.005655000	9.016027000	6.261782000
H	3.576451000	10.047456000	6.199102000
H	5.176075000	10.728143000	5.842637000
C	4.107972000	3.763586000	4.016051000
H	4.909754000	4.396925000	3.646312000
H	3.930496000	2.968806000	3.292475000
H	4.405950000	3.333049000	4.972388000
C	1.566694000	3.892903000	4.184553000
H	1.412336000	3.411823000	5.154194000
H	1.488444000	3.142345000	3.400901000
C	6.573370000	9.422845000	1.453491000
H	7.373235000	10.157953000	1.589693000
H	6.186471000	9.507828000	0.437594000
H	6.984903000	8.429111000	1.607382000
C	0.962612000	7.560644000	4.483862000
C	0.622904000	5.081770000	3.970919000
H	0.326528000	5.192616000	2.923216000
H	-0.272596000	5.027368000	4.586739000
H	-0.023205000	7.539026000	4.945764000
H	0.877866000	8.035536000	3.501302000
H	1.618497000	8.156261000	5.116733000
Cl	5.687204000	4.018840000	8.148414000
H	8.157482000	7.345321000	5.565712000
C	8.406242000	5.509390000	7.542329000

C	9.413869000	4.701223000	6.700839000
C	10.037064000	5.811283000	7.269018000
C	11.277542000	6.241951000	6.812692000
C	11.912182000	5.565599000	5.777033000
C	11.300749000	4.455880000	5.204347000
C	10.060852000	4.028544000	5.664856000
H	12.882319000	5.893349000	5.427007000
H	11.795318000	3.915014000	4.407674000
H	9.592914000	3.157359000	5.220800000
H	9.550830000	6.336694000	8.083014000
H	11.754065000	7.097764000	7.273490000
H	7.934580000	4.489662000	8.232452000
H	7.962865000	3.176244000	7.090842000
H	6.524573000	6.740314000	2.702641000

C	9.313000000	4.610222000	6.726626000
C	10.403705000	5.140929000	6.041237000
C	11.214695000	4.328050000	5.257207000
C	10.942483000	2.969891000	5.150340000
C	9.858770000	2.430095000	5.835021000
C	9.049438000	3.244638000	6.616718000
H	11.576335000	2.333747000	4.546485000
H	9.649077000	1.370132000	5.767662000
H	8.212159000	2.816503000	7.153213000
H	10.630575000	6.196889000	6.130703000
H	12.063516000	4.754614000	4.738305000
H	8.941662000	6.424110000	7.800688000
H	8.130994000	5.021484000	8.475340000
H	3.562869000	7.714398000	4.372420000

[L4+Hc+BnB]$^+$
E [Eh]: −1698.859529133924

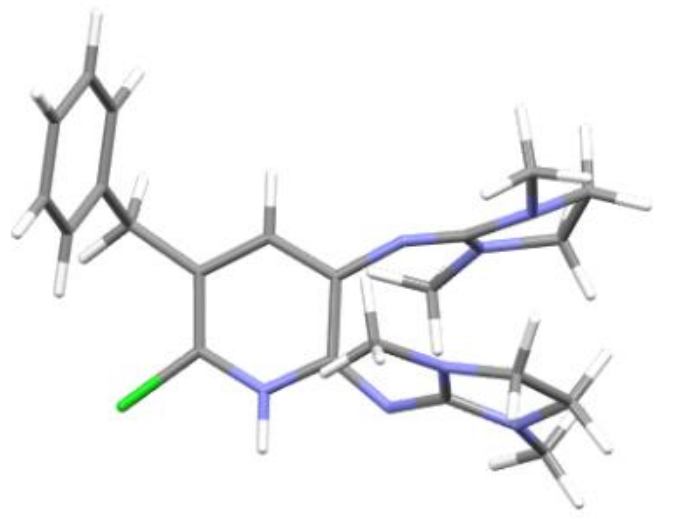

N	4.972958000	5.662974000	7.339040000
N	4.643693000	9.387922000	4.519800000
N	4.506438000	8.515765000	2.475941000
N	3.372921000	6.148974000	5.784383000
N	5.515823000	7.172157000	4.076952000
N	3.303834000	5.202311000	3.583888000
N	1.741167000	6.640022000	4.189689000
C	6.229082000	5.601459000	7.868532000
C	4.929800000	8.277461000	3.768600000
C	7.302649000	6.031706000	7.140984000
C	2.879545000	6.017306000	4.567978000
C	4.661090000	6.141741000	6.104562000
C	7.020821000	6.568799000	5.865889000
C	5.751351000	6.684054000	5.338192000
C	3.894444000	10.361613000	3.733014000
H	4.219650000	11.376347000	3.960256000
H	2.819644000	10.291266000	3.938918000
C	4.224068000	9.936336000	2.305352000
H	3.399871000	10.095671000	1.611185000
H	5.106524000	10.466752000	1.925993000
C	4.634189000	9.474434000	5.964368000
H	3.713729000	9.073532000	6.400735000
H	4.721946000	10.523185000	6.246443000
H	5.484127000	8.945030000	6.383625000
C	4.349647000	4.209085000	3.692920000
H	5.294107000	4.581151000	3.293143000
H	4.048752000	3.318064000	3.141477000
H	4.493756000	3.928609000	4.734526000
C	2.388417000	5.226435000	2.444657000
H	1.840661000	4.281366000	2.386363000
H	2.930497000	5.367645000	1.510019000
C	5.052729000	7.766231000	1.364482000
H	6.013202000	8.175710000	1.030213000
H	4.352523000	7.796094000	0.529288000
H	5.207774000	6.737084000	1.675342000
C	1.124453000	7.727687000	4.909777000
C	1.468384000	6.410628000	2.775485000
H	1.732987000	7.302235000	2.198461000
H	0.415145000	6.186463000	2.608496000
H	0.041145000	7.601924000	4.915394000
H	1.359731000	8.691652000	4.447643000
H	1.487910000	7.721201000	5.933776000
Cl	6.305392000	4.934900000	9.452632000
H	7.846939000	6.927756000	5.264985000

[L4+Ha+BnC]$^+$
E [Eh]: −1698.866707022576

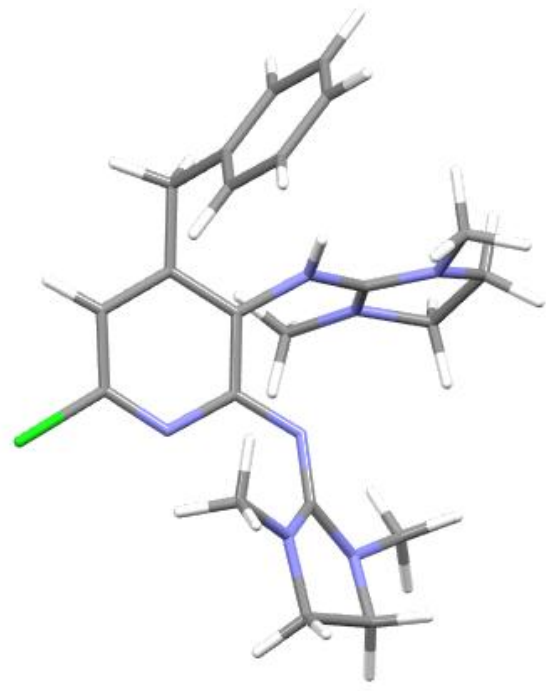

N	4.230301000	5.716651000	7.236212000
N	5.351912000	9.151009000	4.444411000
N	6.162301000	8.148051000	2.646819000
N	3.825735000	6.470635000	5.043677000
N	6.521130000	7.086260000	4.700673000
N	2.485028000	4.463502000	5.012249000
N	1.595524000	6.375196000	4.357462000
C	5.081667000	5.515878000	8.214796000
C	6.006906000	8.080930000	3.982379000
C	6.444380000	5.787435000	8.156805000
C	2.718909000	5.790033000	4.862070000
C	4.680659000	6.221566000	6.069626000
C	6.939471000	6.343022000	6.981453000
C	6.041748000	6.582153000	5.944318000
C	4.848167000	9.940794000	3.316533000
H	4.951001000	11.003932000	3.523859000
H	3.794558000	9.714694000	3.132574000
C	5.742365000	9.466246000	2.167105000
H	5.208279000	9.378161000	1.223058000
H	6.609491000	10.119392000	2.024894000
C	4.960579000	9.433164000	5.813646000
H	4.034148000	8.922378000	6.078481000
H	4.824010000	10.509046000	5.906932000
H	5.744907000	9.126206000	6.499699000
C	3.390502000	3.469527000	5.539856000
H	4.415984000	3.737852000	5.295779000
H	3.164283000	2.513406000	5.066919000
H	3.304733000	3.370570000	6.623808000
C	1.077033000	4.158277000	4.772741000
H	0.531265000	4.089837000	5.719558000
H	0.971561000	3.214717000	4.239167000
C	7.005574000	7.267227000	1.863866000
H	8.065104000	7.528666000	1.942642000
H	6.702980000	7.335425000	0.820745000
H	6.867476000	6.234463000	2.183351000
C	1.574115000	7.706869000	3.809683000
C	0.628819000	5.365418000	3.945678000
H	0.703733000	5.170354000	2.868386000
H	-0.388557000	5.680625000	4.172374000
H	0.569771000	8.120938000	3.898532000
H	1.865280000	7.719522000	2.751332000
H	2.265377000	8.326970000	4.373258000
Cl	4.430528000	4.832052000	9.682673000
H	7.089818000	5.574816000	8.995405000

C	8.732051000	5.926351000	7.611183000
C	9.498323000	4.901577000	6.801880000
C	10.451577000	5.296747000	5.867447000
C	11.129406000	4.351906000	5.103544000
C	10.857900000	3.000043000	5.268185000
C	9.908152000	2.595969000	6.201936000
C	9.232353000	3.541048000	6.961198000
H	11.388341000	2.263243000	4.679390000
H	9.700597000	1.543004000	6.342870000
H	8.498289000	3.219263000	7.690761000
H	10.677020000	6.349579000	5.743568000
H	11.873704000	4.673181000	4.386473000
H	9.209842000	6.902616000	7.510395000
H	8.756110000	5.668413000	8.668778000
H	4.198765000	5.307102000	7.885395000

C	8.409403000	6.650591000	6.826226000
C	9.055283000	5.982947000	5.627212000
C	9.943322000	6.689514000	4.815757000
C	10.522510000	6.090816000	3.702424000
C	10.214481000	4.774106000	3.378981000
C	9.333417000	4.058742000	4.182864000
C	8.759306000	4.658585000	5.298465000
H	10.665438000	4.305515000	2.514207000
H	9.098550000	3.028909000	3.946598000
H	8.075509000	4.092984000	5.919636000
H	10.183911000	7.717651000	5.059604000
H	11.216962000	6.651915000	3.090280000
H	8.542851000	7.732444000	6.738759000
H	8.927094000	6.349047000	7.739102000
H	7.235789000	6.530812000	4.249572000

$[L4+Hb+BnC]^+$
E [Eh]: −1698.866939700150

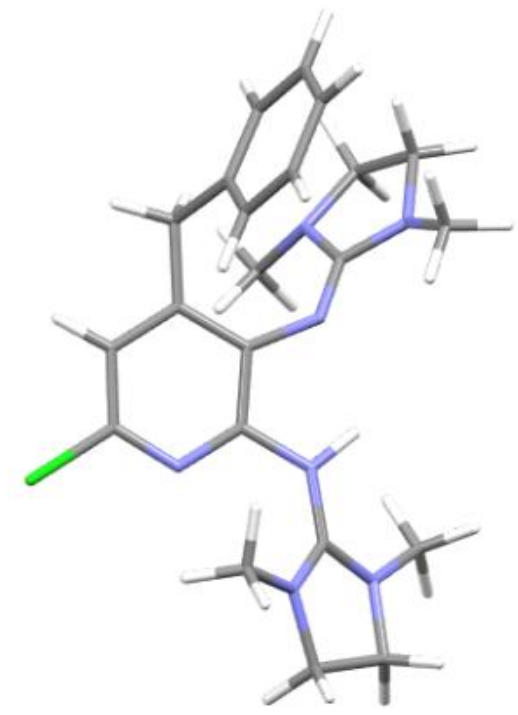

N	4.101759000	5.257404000	7.142232000
N	6.457251000	9.274955000	4.708234000
N	6.627745000	8.289363000	2.714114000
N	3.424518000	5.769591000	4.956906000
N	5.672073000	7.007838000	4.394940000
N	2.010713000	3.897950000	5.423330000
N	1.158891000	5.640620000	4.365086000
C	5.028334000	5.299244000	8.083406000
C	6.225867000	8.116670000	4.002523000
C	6.265375000	5.892883000	7.898689000
C	2.254856000	5.113698000	4.936859000
C	4.389905000	5.814691000	5.985222000
C	6.562081000	6.514832000	6.685317000
C	5.573829000	6.521807000	5.675132000
C	6.894774000	10.329360000	3.788156000
H	7.686761000	10.932047000	4.231087000
H	6.051513000	10.987693000	3.548331000
C	7.354440000	9.542164000	2.562514000
H	7.104917000	10.036429000	1.623819000
H	8.432499000	9.352940000	2.581109000
C	5.618445000	9.686863000	5.819242000
H	4.673380000	10.117979000	5.466743000
H	6.144909000	10.444503000	6.399040000
H	5.401079000	8.848258000	6.472629000
C	2.958160000	2.918813000	5.926899000
H	3.952062000	3.139549000	5.550063000
H	2.652729000	1.938121000	5.561730000
H	2.986860000	2.920436000	7.015384000
C	0.574432000	3.605195000	5.330938000
H	0.100363000	3.735989000	6.306848000
H	0.422273000	2.579570000	5.001609000
C	6.911231000	7.178728000	1.828117000
H	7.976885000	6.934810000	1.838802000
H	6.611814000	7.434811000	0.811107000
H	6.350239000	6.312568000	2.166157000
C	1.073871000	6.923275000	3.700466000
C	0.090911000	4.638938000	4.305042000
H	0.011257000	4.221707000	3.296755000
H	-0.863254000	5.086672000	4.576113000
H	0.063465000	7.313360000	3.814067000
H	1.299800000	6.845376000	2.632855000
H	1.763435000	7.626144000	4.165299000

$[L4+Hc+BnC]^+$
E [Eh]: −1698.854759890302

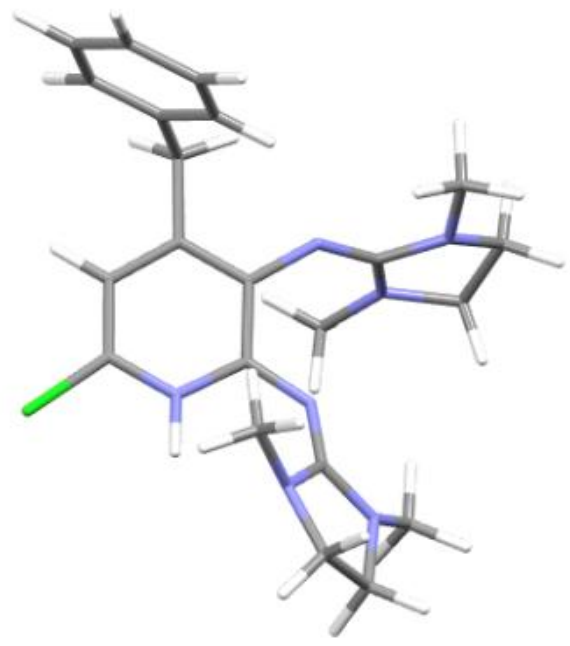

N	4.640789000	5.652338000	6.996263000
N	5.060202000	9.451309000	4.305264000
N	5.664096000	8.540828000	2.378131000
N	3.743086000	6.577013000	5.030341000
N	6.332936000	7.400977000	4.291200000
N	2.625696000	4.439304000	5.031950000
N	1.509107000	6.282696000	4.460874000
C	5.649129000	5.400181000	7.871311000
C	5.695720000	8.383626000	3.737051000
C	6.901934000	5.850440000	7.592198000
C	2.705600000	5.801186000	4.865515000
C	4.793778000	6.336536000	5.824285000
C	7.136895000	6.575296000	6.410123000
C	6.094552000	6.842868000	5.512331000
C	4.436407000	10.282733000	3.283713000
H	4.534310000	11.339818000	3.529194000
H	3.369714000	10.048710000	3.179531000
C	5.222854000	9.885182000	2.033717000
H	4.613468000	9.881209000	1.130966000
H	6.081090000	10.549979000	1.873076000
C	4.776718000	9.638613000	5.706247000
H	3.823033000	9.191166000	6.003883000
H	4.742443000	10.707475000	5.918390000
H	5.570259000	9.203660000	6.308498000
C	3.759883000	3.548322000	4.817336000
H	3.672652000	3.064474000	3.840520000
H	3.787349000	2.776600000	5.587028000
H	4.694026000	4.099937000	4.838096000
C	1.312356000	3.970593000	4.566877000
H	0.907570000	3.209042000	5.231278000
H	1.412202000	3.541239000	3.564728000
C	6.537728000	7.801472000	1.495601000
H	7.516685000	8.284439000	1.394697000
H	6.078875000	7.729191000	0.509458000
H	6.681998000	6.803036000	1.899293000
C	1.168041000	7.688665000	4.490230000
C	0.479528000	5.252844000	4.544657000
H	-0.200272000	5.301703000	3.695011000
H	-0.104551000	5.378614000	5.462995000
H	0.713647000	7.972020000	5.445503000
H	0.462347000	7.904618000	3.688596000
H	2.073486000	8.269376000	4.339415000

Cl	4.638177000	4.538583000	9.600288000
H	7.001051000	5.866237000	8.689624000
C	7.925236000	7.124647000	6.492135000
C	8.696827000	6.643543000	5.280319000
C	9.557113000	7.518767000	4.619961000
C	10.334941000	7.085850000	3.552222000
C	10.255301000	5.765804000	3.124034000
C	9.393525000	4.887045000	3.771031000
C	8.621844000	5.323346000	4.840757000
H	10.862767000	5.423348000	2.296563000
H	9.328428000	3.856425000	3.446411000
H	7.957927000	4.629646000	5.341398000
H	9.627170000	8.546829000	4.955779000
H	11.007930000	7.776400000	3.059757000
H	7.840224000	8.211159000	6.431639000
H	8.515893000	6.915861000	7.387401000
H	3.660529000	6.394566000	4.192557000

Cl	5.220269000	4.506840000	9.273893000
H	7.710049000	5.639207000	8.276130000
C	8.543182000	7.014797000	6.097527000
C	9.386827000	5.838173000	5.651081000
C	10.381913000	5.318792000	6.474652000
C	11.130794000	4.217348000	6.072772000
C	10.887968000	3.623126000	4.841298000
C	9.895831000	4.137522000	4.011777000
C	9.148617000	5.236445000	4.413847000
H	11.472229000	2.768911000	4.525215000
H	9.710308000	3.683652000	3.046458000
H	8.378787000	5.642297000	3.769128000
H	10.584453000	5.784214000	7.432260000
H	11.906444000	3.829032000	6.720086000
H	8.511479000	7.774009000	5.318079000
H	8.984721000	7.461281000	6.990515000
H	3.722891000	5.286065000	7.211742000

$[L4+BnD]^+$
E [Eh]: −1698.846920501901

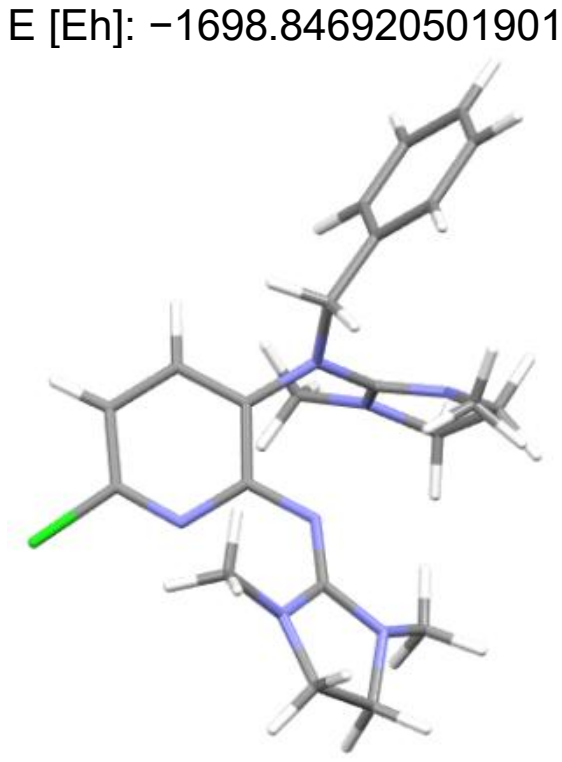

N	7.109418000	7.046261000	4.782758000
N	4.790671000	10.813240000	4.180141000
N	4.325274000	10.512713000	2.041698000
N	5.336616000	7.656608000	3.353836000
N	6.552598000	10.086356000	2.776291000
N	5.530059000	5.295073000	2.910931000
N	3.520239000	6.216529000	3.084925000
C	8.258300000	7.341962000	5.343996000
C	5.266985000	10.436384000	2.988623000
C	8.969606000	8.519765000	5.130852000
C	4.868885000	6.448452000	3.158755000
C	6.544863000	7.915783000	3.921382000
C	8.386596000	9.442743000	4.276639000
C	7.172753000	9.161305000	3.673088000
C	3.344692000	11.033121000	4.109909000
H	3.084865000	11.960970000	4.616149000
H	2.805811000	10.212200000	4.589575000
C	3.100390000	11.104467000	2.594655000
H	2.234119000	10.523983000	2.283235000
H	2.978806000	12.130492000	2.241788000
C	5.544853000	11.004784000	5.402921000
H	5.636162000	10.076454000	5.970129000
H	5.029604000	11.746243000	6.011003000
H	6.540131000	11.374572000	5.172893000
C	6.922917000	5.182068000	2.528954000
H	7.297599000	6.140503000	2.175771000
H	7.004190000	4.459816000	1.715235000
H	7.539057000	4.855797000	3.366758000
C	4.599305000	4.209906000	2.606175000
H	4.876100000	3.294320000	3.127871000
H	4.600322000	4.009318000	1.529375000
C	4.332994000	9.774897000	0.791049000
H	4.688165000	10.386979000	-0.038247000
H	3.314787000	9.454377000	0.576282000
H	4.949884000	8.886693000	0.904208000
C	2.558874000	7.109483000	3.689268000
C	3.261189000	4.779746000	3.078784000
H	2.434067000	4.527072000	2.416329000
H	3.013872000	4.436275000	4.090083000
H	1.606050000	7.028820000	3.165343000

$[L4+BnE]^+$
E [Eh]: −1698.848656172944

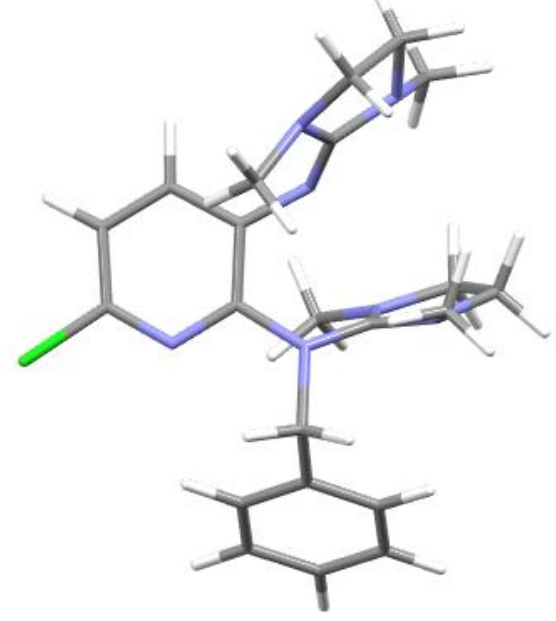

H	4.702716000	2.222947000	7.956601000
Cl	6.389175000	3.227438000	12.402633000
N	2.126761000	4.933580000	10.582537000
N	2.245164000	6.581283000	8.856347000
N	0.275949000	5.699059000	9.312631000
N	2.438350000	3.672698000	8.088727000
N	0.910989000	2.949752000	6.493043000
N	1.480875000	1.446930000	8.051280000
N	4.188475000	4.075320000	11.274759000
C	3.289719000	4.171414000	10.309407000
C	3.397649000	3.498586000	9.074348000
C	4.560626000	2.745293000	8.893830000
C	5.507208000	2.643316000	9.901894000
H	6.404808000	2.055950000	9.779343000
C	5.257561000	3.325978000	11.085275000
C	1.582850000	5.697927000	9.583608000
C	1.366922000	7.212617000	7.870595000
H	1.700954000	6.980804000	6.858374000
H	1.392167000	8.294175000	8.004568000
C	-0.014149000	6.607352000	8.198280000
H	-0.746591000	7.359274000	8.492412000
H	-0.421245000	6.034244000	7.364096000
C	3.643158000	6.944080000	8.975228000
H	4.019389000	6.656276000	9.951327000
H	3.732431000	8.025575000	8.877576000
H	4.230846000	6.457744000	8.194025000
C	-0.647718000	4.647075000	9.681340000
H	-0.947087000	4.090598000	8.789533000
H	-1.535176000	5.066580000	10.156407000
H	-0.155892000	3.968467000	10.371909000
C	1.692934000	2.734179000	7.608883000
C	0.366721000	1.681471000	6.019861000
H	1.016137000	1.248918000	5.248576000
H	-0.631073000	1.806807000	5.601139000
C	0.380791000	0.846154000	7.292565000
H	-0.561125000	0.948937000	7.846242000
H	0.561636000	-0.212087000	7.110683000
C	1.262905000	3.961211000	5.518897000
H	1.747204000	4.787151000	6.031590000
H	0.363662000	4.315842000	5.013936000

H	2.933972000	8.125156000	3.605925000
H	2.396856000	6.877844000	4.748920000
H	9.915206000	8.701634000	5.616856000
H	8.878984000	10.388545000	4.084832000
C	7.250140000	10.463270000	1.527880000
C	6.996856000	11.892827000	1.120057000
C	7.017135000	12.921568000	2.061948000
C	6.812252000	14.238157000	1.672626000
C	6.590632000	14.543739000	0.333406000
C	6.580272000	13.526604000	-0.612240000
C	6.779275000	12.207743000	-0.218214000
H	6.433589000	15.570144000	0.029503000
H	6.416971000	13.757310000	-1.656737000
H	6.775325000	11.419700000	-0.962184000
H	7.199037000	12.693725000	3.104889000
H	6.834386000	15.028264000	2.411917000
Cl	8.923472000	6.147560000	6.427228000
H	8.308507000	10.314973000	1.741005000
H	7.001814000	9.782651000	0.711772000

H	1.954025000	3.570056000	4.762975000
C	1.596108000	1.051956000	9.445444000
H	2.563290000	1.321755000	9.854081000
H	1.494580000	-0.030596000	9.503617000
H	0.809492000	1.505609000	10.060442000
C	1.810802000	5.357867000	11.978646000
H	2.273611000	4.618789000	12.624563000
H	0.729285000	5.305089000	12.103949000
C	2.309630000	6.748097000	12.276349000
C	3.605581000	6.956639000	12.749463000
H	4.240051000	6.104156000	12.949856000
C	4.079669000	8.247877000	12.944483000
H	5.083464000	8.399900000	13.319391000
C	3.267834000	9.342611000	12.665652000
C	1.971423000	9.142857000	12.204830000
H	1.328069000	9.990944000	12.008614000
C	1.495561000	7.850420000	12.016071000
H	0.477427000	7.697061000	11.675404000
H	3.638872000	10.347147000	12.821406000

[L4+Bn(p-CF_3)E]$^+$
E [Eh]: −2035.923001166286

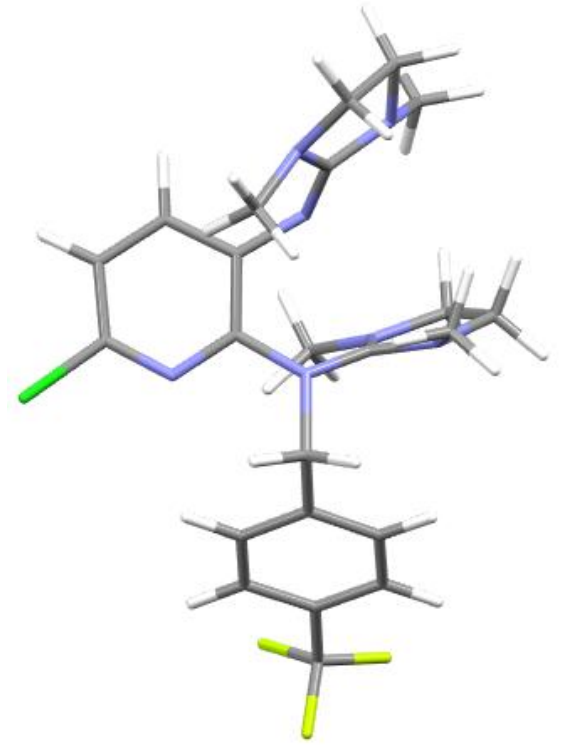

H	4.709575000	2.245083000	7.932906000
Cl	6.405538000	3.266294000	12.371991000
N	2.127764000	4.945388000	10.563329000
N	2.257317000	6.598269000	8.844544000
N	0.281593000	5.724748000	9.292776000
N	2.434713000	3.679457000	8.073000000
N	0.893991000	2.938382000	6.497947000
N	1.480501000	1.452200000	8.065088000
N	4.196180000	4.098784000	11.250935000
C	3.294185000	4.186986000	10.287943000
C	3.399712000	3.513603000	9.054186000
C	4.567798000	2.767856000	8.869893000
C	5.518657000	2.674160000	9.874251000
H	6.420316000	2.093619000	9.748816000
C	5.269542000	3.356015000	11.058791000
C	1.587662000	5.717370000	9.568759000
C	1.389070000	7.222924000	7.844944000
H	1.725236000	6.970302000	6.838390000
H	1.424484000	8.306169000	7.960110000
C	0.001046000	6.636279000	8.177342000
H	-0.720698000	7.398115000	8.472303000
H	-0.415461000	6.067472000	7.345161000
C	3.658958000	6.948652000	8.963986000
H	4.026762000	6.677207000	9.948205000
H	3.760547000	8.026401000	8.841252000
H	4.247003000	6.439464000	8.197798000
C	-0.646407000	4.671334000	9.648925000
H	-0.949813000	4.129799000	8.749636000
H	-1.531074000	5.087188000	10.132312000
H	-0.156353000	3.979567000	10.327593000
C	1.685898000	2.735045000	7.608227000
C	0.347561000	1.664475000	6.041811000
H	0.990866000	1.225661000	5.269118000
H	-0.654129000	1.784211000	5.631020000
C	0.374606000	0.841603000	7.322356000
H	-0.562226000	0.947831000	7.883763000
H	0.556353000	-0.217885000	7.149244000

[L4+Bn(o-CF_3)E]$^+$
E [Eh]: −2035.912835510891

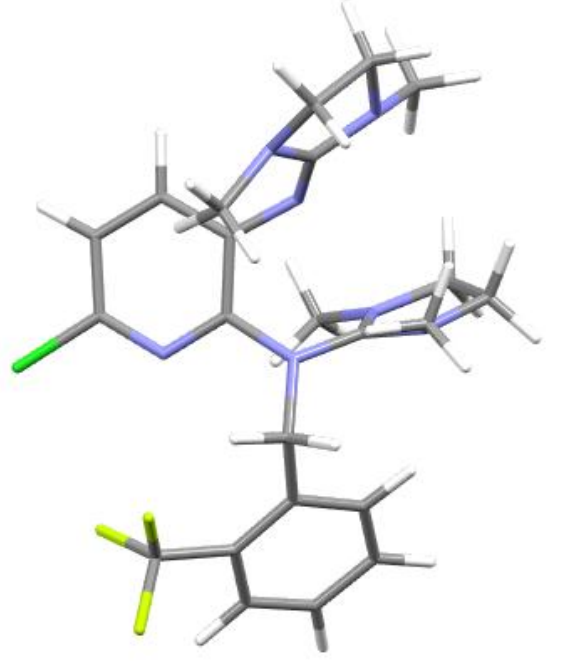

H	4.609385000	2.228456000	7.719462000
Cl	6.696966000	3.510515000	11.901115000
N	2.250912000	5.092286000	10.387376000
N	2.327559000	6.642179000	8.592648000
N	0.351494000	5.833286000	9.160615000
N	2.349754000	3.667558000	7.968757000
N	0.723845000	2.822427000	6.537614000
N	1.449780000	1.423511000	8.126459000
N	4.381565000	4.296074000	10.950826000
C	3.396994000	4.313624000	10.066790000
C	3.393912000	3.564718000	8.873580000
C	4.546857000	2.809043000	8.630693000
C	5.578815000	2.782327000	9.550621000
H	6.469849000	2.195164000	9.385245000
C	5.436212000	3.539795000	10.710395000
C	1.668599000	5.820760000	9.397251000
C	1.441143000	7.202517000	7.573107000
H	1.722847000	6.843424000	6.582111000
H	1.517440000	8.289581000	7.580643000
C	0.049994000	6.703992000	8.017378000
H	-0.615525000	7.515900000	8.312339000
H	-0.442274000	6.118145000	7.240931000
C	3.731329000	6.995622000	8.674351000
H	4.102817000	6.797274000	9.675205000
H	3.836521000	8.059591000	8.464869000
H	4.315786000	6.425433000	7.949414000
C	-0.563283000	4.779183000	9.553273000
H	-0.938930000	4.273594000	8.661117000
H	-1.403449000	5.182878000	10.119609000
H	-0.031859000	4.053915000	10.161918000
C	1.594134000	2.687388000	7.599149000
C	0.178915000	1.517622000	6.177425000
H	0.778870000	1.057462000	5.382494000
H	-0.850964000	1.596773000	5.831588000
C	0.312825000	0.754789000	7.487964000
H	-0.586906000	0.864729000	8.106191000
H	0.510083000	-0.307059000	7.349423000

C	1.232305000	3.942459000	5.511405000	C	0.971161000	3.785895000	5.486229000
H	1.714904000	4.776916000	6.011744000	H	1.459653000	4.655110000	5.916348000
H	0.326938000	4.286215000	5.009987000	H	0.026719000	4.081721000	5.028293000
H	1.920000000	3.548246000	4.754081000	H	1.622051000	3.374161000	4.705865000
C	1.612488000	1.067599000	9.460621000	C	1.686744000	1.105821000	9.524856000
H	2.587314000	1.332245000	9.854220000	H	2.678161000	1.412590000	9.838580000
H	1.503861000	-0.013659000	9.528093000	H	1.613080000	0.026383000	9.646867000
H	0.837706000	1.530869000	10.083557000	H	0.943108000	1.576837000	10.179300000
C	1.792508000	5.329041000	11.962355000	C	1.858546000	5.305981000	11.802555000
H	2.235438000	4.567214000	12.595888000	H	2.240262000	4.458139000	12.349602000
H	0.708899000	5.287660000	12.071273000	H	0.770078000	5.283325000	11.857099000
C	2.302626000	6.704189000	12.311700000	C	2.345868000	6.632291000	12.360212000
C	3.592526000	6.883445000	12.812779000	C	3.293379000	6.800108000	13.377613000
H	4.217141000	6.020157000	12.993689000	C	3.694523000	8.087700000	13.747682000
C	4.078390000	8.159176000	13.055639000	H	4.429356000	8.201061000	14.532573000
H	5.079267000	8.293427000	13.442738000	C	3.160809000	9.211174000	13.143197000
C	3.280122000	9.269486000	12.795007000	C	2.182529000	9.057990000	12.168892000
C	1.988098000	9.102601000	12.311198000	H	1.721375000	9.925168000	11.714477000
H	1.364155000	9.965701000	12.126547000	C	1.784703000	7.785366000	11.798025000
C	1.505110000	7.822221000	12.074804000	H	0.997693000	7.678416000	11.062363000
H	0.492151000	7.694780000	11.710892000	C	3.959463000	5.698800000	14.180241000
C	3.808809000	10.649197000	13.092099000	F	3.400873000	4.480480000	14.041355000
F	3.145154000	11.599178000	12.405893000	F	3.903527000	5.988252000	15.498705000
F	5.113763000	10.759181000	12.774820000	F	5.260444000	5.584791000	13.865171000
F	3.693247000	10.947726000	14.401456000	H	3.487123000	10.196283000	13.447563000

$[L4+Bn(o,p\text{-}CF_3)_2E]^+$
E [Eh]: −2372.985450124136

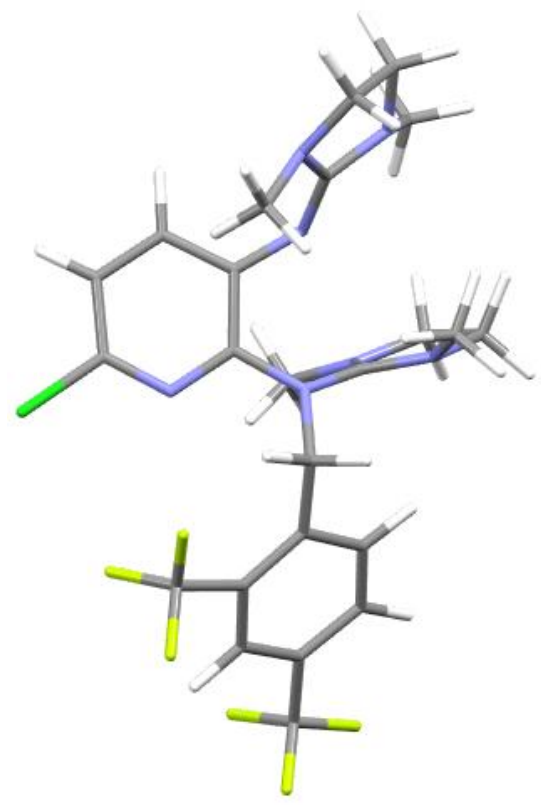

H	4.574875000	2.209077000	7.748258000
Cl	6.678118000	3.513863000	11.914479000
N	2.228822000	5.091900000	10.406930000
N	2.301090000	6.636700000	8.605902000
N	0.325962000	5.832530000	9.185758000
N	2.317622000	3.660382000	7.996890000
N	0.730462000	2.812173000	6.524374000
N	1.457985000	1.397893000	8.098470000
N	4.360389000	4.296816000	10.967947000
C	3.372816000	4.308881000	10.087177000
C	3.363936000	3.554669000	8.897590000
C	4.515217000	2.795031000	8.656271000
C	5.549795000	2.772109000	9.573089000
H	6.439304000	2.182529000	9.408137000
C	5.413419000	3.537185000	10.728786000
C	1.644499000	5.817769000	9.414185000
C	1.409435000	7.202514000	7.593309000
H	1.690712000	6.854602000	6.598378000
H	1.481825000	8.289846000	7.611092000
C	0.021272000	6.694154000	8.035781000
H	-0.653125000	7.501345000	8.322715000
H	-0.462587000	6.098741000	7.261185000
C	3.707414000	6.984028000	8.673621000
H	4.092111000	6.774088000	9.666941000
H	3.815073000	8.049409000	8.472498000
H	4.279343000	6.417709000	7.935920000
C	-0.585082000	4.774981000	9.580019000
H	-0.946884000	4.256375000	8.689435000
H	-1.434647000	5.179390000	10.131308000
H	-0.055383000	4.060805000	10.203104000

C	1.586293000	2.672761000	7.595443000
C	0.208614000	1.505976000	6.135180000
H	0.823437000	1.070475000	5.337889000
H	-0.818790000	1.576198000	5.780280000
C	0.340974000	0.720882000	7.432770000
H	-0.567432000	0.802897000	8.042278000
H	0.559288000	-0.334306000	7.276556000
C	0.974115000	3.798211000	5.493050000
H	1.456138000	4.661473000	5.942028000
H	0.029375000	4.097400000	5.037879000
H	1.629457000	3.405328000	4.706907000
C	1.675069000	1.065355000	9.496947000
H	2.657688000	1.378578000	9.831497000
H	1.611164000	-0.016173000	9.604400000
H	0.915231000	1.520817000	10.143655000
C	1.848537000	5.324116000	11.820512000
H	2.221182000	4.478298000	12.376993000
H	0.760468000	5.321290000	11.884090000
C	2.362606000	6.651434000	12.353189000
C	3.339136000	6.823162000	13.343017000
C	3.779782000	8.106590000	13.667506000
H	4.539521000	8.227382000	14.427727000
C	3.255004000	9.225206000	13.042318000
C	2.241826000	9.074602000	12.105799000
H	1.791812000	9.942436000	11.645656000
C	1.807243000	7.801618000	11.783072000
H	0.997976000	7.695754000	11.072679000
C	3.790667000	10.588506000	13.404482000
F	3.136002000	11.570974000	12.760296000
F	5.096189000	10.694914000	13.083094000
F	3.687563000	10.825116000	14.723986000
C	3.992845000	5.729787000	14.170133000
F	3.440746000	4.510994000	14.031120000
F	3.905600000	6.037412000	15.481964000
F	5.298875000	5.621534000	13.881295000

$[L4b+BnE]^+$
E [Eh]: −2305.868901659484

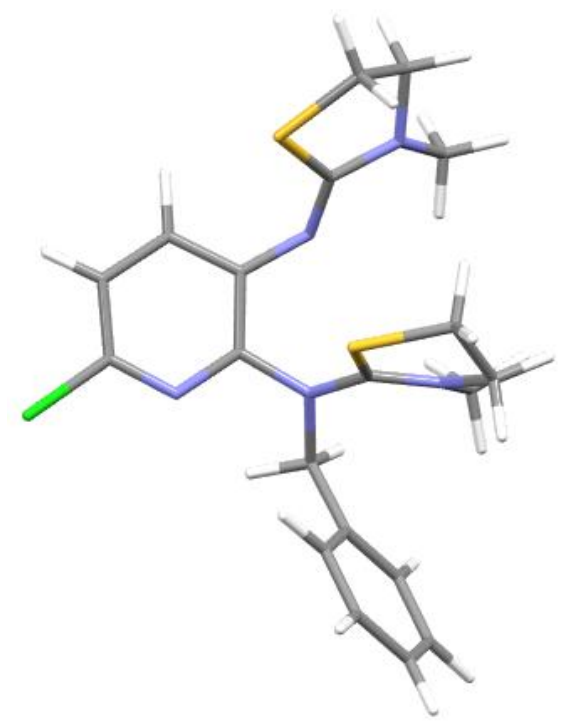

H	10.658490000	1.663677000	13.291423000
S	4.730286000	2.475610000	12.864239000
N	6.375524000	4.607835000	12.856822000
C	7.511743000	3.744559000	12.946757000
Cl	10.171780000	3.174899000	15.674464000
S	6.953455000	0.298208000	10.465619000
N	4.277403000	4.777969000	11.795727000
C	7.858688000	2.950424000	11.839124000
N	7.111379000	3.034192000	10.677949000
C	9.027857000	2.189353000	11.988525000
H	9.380470000	1.588706000	11.161407000
N	5.946567000	2.111582000	8.896259000
C	9.751295000	2.235704000	13.165049000
N	8.202566000	3.799582000	14.072423000
C	9.291097000	3.067501000	14.185043000
C	5.210544000	4.100634000	12.446540000
C	3.255108000	2.625721000	11.796790000
H	3.500588000	2.238506000	10.808351000
H	2.442143000	2.050721000	12.231604000
C	2.965422000	4.120864000	11.772203000
H	2.430406000	4.418640000	10.872279000

$[L4b+Bn(p\text{-}CF_3)E]^+$
E [Eh]: −2642.942948302781

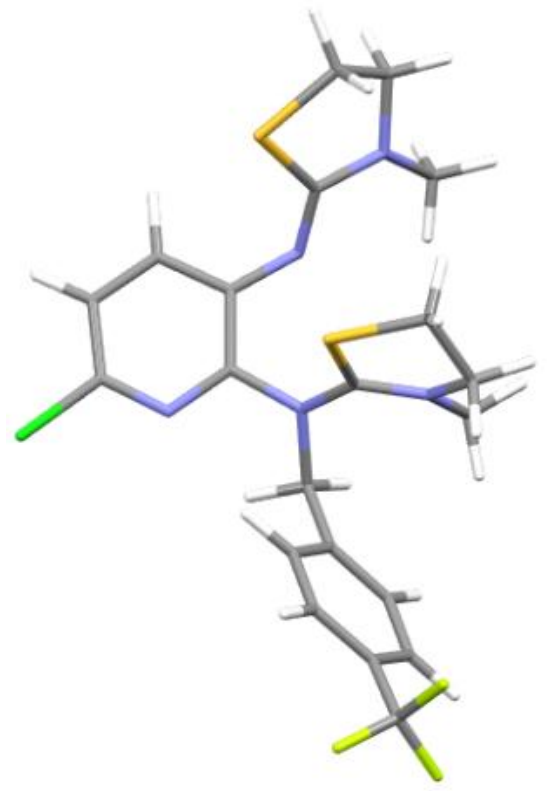

H	10.832483000	1.404509000	12.738115000
S	5.002881000	2.391738000	12.989993000
N	6.646453000	4.514080000	12.796717000
C	7.758094000	3.613422000	12.746056000
Cl	10.623134000	2.849207000	15.204298000
S	6.834497000	0.270848000	10.217632000
N	4.401092000	4.740719000	12.110926000
C	7.965590000	2.843237000	11.588641000
N	7.115446000	2.990951000	10.509816000
C	9.113193000	2.034408000	11.607783000
H	9.363733000	1.446868000	10.735264000
N	5.770900000	2.176419000	8.805191000
C	9.942453000	2.015669000	12.712908000
N	8.550045000	3.607235000	13.804474000
C	9.612920000	2.829538000	13.796308000
C	5.422424000	4.031548000	12.568599000
C	3.364052000	2.595521000	12.208187000
H	3.422532000	2.242421000	11.179336000
H	2.633078000	2.011465000	12.760734000
C	3.095009000	4.092231000	12.287686000
H	2.417931000	4.427230000	11.504026000

H	2.389044000	4.431415000	12.647138000
C	4.501676000	6.028215000	11.083506000
H	4.215434000	6.888799000	11.687425000
H	3.901622000	6.014935000	10.175299000
H	5.550356000	6.096326000	10.807733000
C	6.700900000	2.006580000	10.030724000
C	5.829146000	-0.254210000	9.136322000
H	6.198727000	-1.187763000	8.720332000
H	4.835051000	-0.412816000	9.552252000
C	5.844013000	0.880181000	8.123044000
H	6.696425000	0.784013000	7.439346000
H	4.929711000	0.894429000	7.528987000
C	5.855102000	3.380577000	8.195724000
H	4.957699000	3.384302000	7.578060000
H	6.726907000	3.551410000	7.555323000
H	5.804856000	4.184419000	8.924440000
C	6.481557000	5.976602000	13.434666000
C	5.325861000	6.319751000	14.336797000
C	4.710330000	7.565045000	14.245742000
C	4.891564000	5.414845000	15.306246000
C	3.844517000	5.746287000	16.154720000
C	3.227529000	6.989604000	16.052544000
C	3.667751000	7.902210000	15.102560000
H	5.056840000	8.285939000	13.514025000
H	5.384968000	4.456453000	15.407124000
H	3.518651000	5.039506000	16.906618000
H	3.202993000	8.876567000	15.028137000
H	7.408972000	5.949408000	14.003633000
H	6.598159000	6.715841000	12.643306000
H	2.415581000	7.248724000	16.719051000

H	2.679386000	4.373303000	13.258303000
C	4.512949000	6.015570000	11.415567000
H	4.340406000	6.855593000	12.088152000
H	3.765832000	6.037746000	10.624280000
H	5.499033000	6.091530000	10.966075000
C	6.612128000	2.002815000	9.864750000
C	5.562286000	-0.189446000	8.990039000
H	5.847879000	-1.122593000	8.511769000
H	4.608462000	-0.321909000	9.498909000
C	5.525779000	0.977253000	8.014653000
H	6.293830000	0.865291000	7.239617000
H	4.554968000	1.053306000	7.523682000
C	5.654103000	3.471305000	8.158772000
H	4.676413000	3.549349000	7.683782000
H	6.428621000	3.610919000	7.397322000
H	5.765199000	4.250468000	8.907165000
C	6.860048000	5.857260000	13.401231000
C	5.929259000	6.116553000	14.556269000
C	5.223470000	7.311493000	14.643778000
C	5.788825000	5.164324000	15.567437000
C	4.934465000	5.396106000	16.631854000
C	4.216566000	6.588200000	16.699863000
C	4.368259000	7.552147000	15.712963000
H	5.347710000	8.072089000	13.882302000
H	6.363046000	4.247867000	15.526305000
H	4.832139000	4.658928000	17.416489000
H	3.824665000	8.484060000	15.777234000
C	3.235904000	6.791554000	17.825145000
F	3.711296000	6.327242000	18.993639000
F	2.083643000	6.124567000	17.575634000
F	2.916889000	8.084657000	17.996290000
H	7.891157000	5.835909000	13.747254000
H	6.778884000	6.635745000	12.645140000

[L5a+Ha+BnC]$^+$
E [Eh]: −2158.420432016989

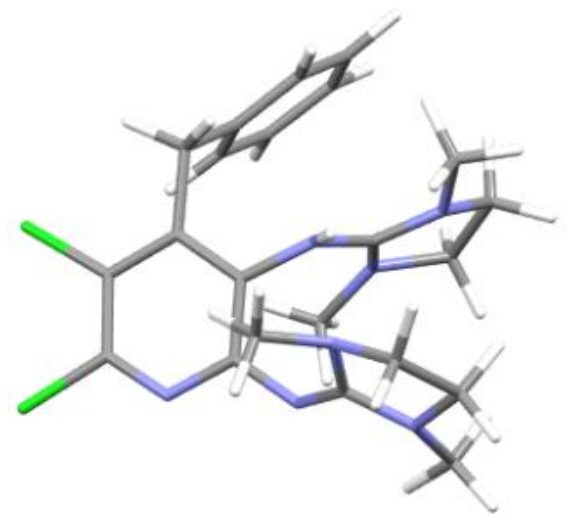

N	7.593020000	5.690012000	6.690597000
N	6.426305000	9.471823000	4.383413000
N	5.386432000	8.891380000	2.509079000
N	5.517169000	6.227263000	5.799435000
N	6.745697000	7.268595000	3.495560000
N	4.824470000	4.940302000	3.863716000
N	3.339628000	6.182144000	5.002686000
C	8.903890000	5.716951000	6.638719000
C	6.219898000	8.497080000	3.497966000
C	9.634582000	6.211412000	5.543781000
C	4.648739000	5.805282000	4.950583000
C	6.875090000	6.161750000	5.665934000
C	8.924689000	6.713962000	4.451201000
C	7.533377000	6.707608000	4.547612000
C	5.627692000	10.650554000	4.017472000
H	6.235197000	11.549842000	4.095698000
H	4.772755000	10.736957000	4.693858000
C	5.199794000	10.345868000	2.583036000
H	4.164605000	10.612673000	2.379354000
H	5.849168000	10.840965000	1.856638000
C	6.921657000	9.374159000	5.754269000
H	6.198546000	8.869177000	6.396280000
H	7.080128000	10.389460000	6.109336000
H	7.870739000	8.855314000	5.790132000
C	5.766550000	3.823931000	3.966179000
H	5.792435000	3.310254000	3.006311000
H	5.460508000	3.112744000	4.739711000

[L5a+Hb+BnC]$^+$
E [Eh]: −2158.424228018406

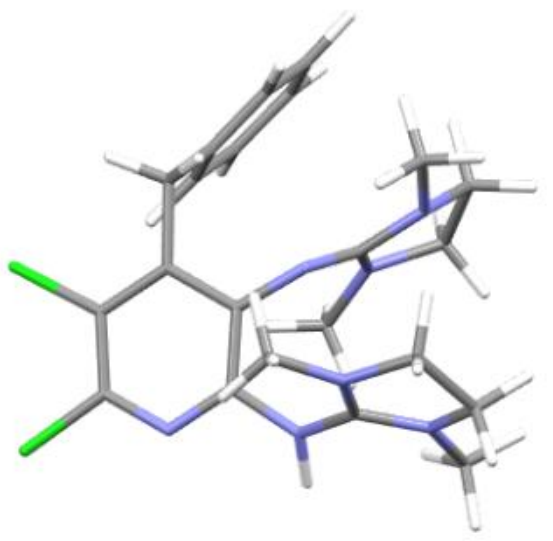

N	7.555823000	5.790024000	6.957983000
N	6.263211000	9.408291000	4.181923000
N	5.407898000	8.522726000	2.327104000
N	5.450253000	6.176196000	6.072869000
N	6.645875000	7.075587000	3.654961000
N	4.668166000	4.894034000	4.228016000
N	3.375541000	6.505434000	5.004325000
C	8.870058000	5.828514000	6.909240000
C	6.170393000	8.257991000	3.448809000
C	9.558904000	6.234338000	5.754739000
C	4.547222000	5.869488000	5.126448000
C	6.863328000	6.184390000	5.905565000
C	8.835636000	6.657419000	4.639023000
C	7.426788000	6.685671000	4.726331000
C	5.449744000	10.466815000	3.595087000
H	5.977144000	11.419585000	3.629586000
H	4.502643000	10.573276000	4.139579000
C	5.232203000	9.962828000	2.172524000
H	4.243673000	10.202008000	1.781605000
H	5.987175000	10.369171000	1.491032000
C	6.659796000	9.533508000	5.565230000
H	5.824764000	9.345715000	6.252285000
H	7.014815000	10.549446000	5.731578000
H	7.473751000	8.859293000	5.800726000
C	5.788589000	3.989907000	4.054960000
H	6.528374000	4.411973000	3.372898000
H	5.409440000	3.053207000	3.649809000

H	6.769212000	4.172842000	4.191931000
C	3.488634000	4.568251000	3.365623000
H	3.159949000	3.636070000	3.837825000
H	3.498732000	4.427026000	2.286186000
C	5.269261000	8.209372000	1.232632000
H	6.179602000	8.302738000	0.634088000
H	4.440231000	8.653528000	0.686088000
H	5.035430000	7.154576000	1.375824000
C	2.898762000	7.331042000	5.759771000
C	2.623994000	5.738134000	3.818390000
H	2.588545000	6.535526000	3.063272000
H	1.602057000	5.444360000	4.054846000
H	1.854483000	7.201735000	6.041532000
H	2.992131000	8.257027000	5.176961000
H	3.504867000	7.412933000	6.657973000
Cl	9.735986000	5.095024000	8.023100000
Cl	11.365787000	6.213036000	5.561094000
C	9.600484000	7.255758000	3.212529000
C	9.465601000	8.755580000	3.059759000
C	8.932798000	9.306017000	1.895956000
C	8.788941000	10.681886000	1.757967000
C	9.175342000	11.528918000	2.790614000
C	9.724390000	10.989929000	3.949657000
C	9.872842000	9.614778000	4.079826000
H	9.075964000	12.601201000	2.682670000
H	10.053050000	11.643313000	4.747639000
H	10.316106000	9.205797000	4.979840000
H	8.642360000	8.650075000	1.083593000
H	8.395273000	11.093438000	0.836801000
H	10.653186000	6.980376000	3.234912000
H	9.163875000	6.769170000	2.338802000
H	6.261989000	6.601535000	2.905865000

[L5a+Hc+BnC]$^{+}$
E [Eh]: −2158.426788064438

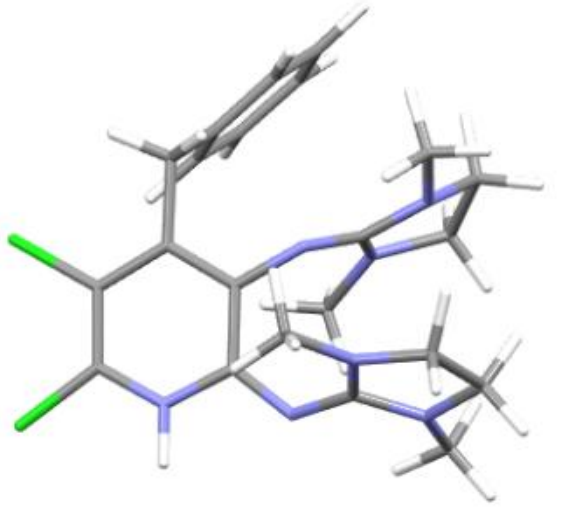

N	7.657505000	5.737833000	6.883325000
N	6.207908000	9.317554000	4.182837000
N	5.365670000	8.382834000	2.348576000
N	5.555002000	6.052177000	6.056605000
N	6.761934000	7.031559000	3.614094000
N	4.615202000	5.012502000	4.120073000
N	3.476316000	6.603503000	5.135843000
C	9.017348000	5.788579000	6.883116000
C	6.177886000	8.169437000	3.443909000
C	9.668035000	6.221996000	5.759030000
C	4.633163000	5.913331000	5.115996000
C	6.859211000	6.116589000	5.846391000
C	8.913490000	6.648625000	4.633674000
C	7.522988000	6.645181000	4.688630000
C	5.329793000	10.330731000	3.609537000
H	5.810036000	11.308725000	3.630664000
H	4.389430000	10.394093000	4.170641000
C	5.110413000	9.810471000	2.191647000
H	4.102006000	9.993562000	1.822035000
H	5.826214000	10.255488000	1.491486000
C	6.643566000	9.465445000	5.553219000
H	5.852637000	9.212582000	6.266550000
H	6.938650000	10.501905000	5.710202000
H	7.512492000	8.848388000	5.748144000
C	5.623873000	4.013860000	3.856836000
H	6.323978000	4.349476000	3.088906000
H	5.138039000	3.095836000	3.525585000

H	6.253316000	3.787296000	5.016351000
C	3.580999000	4.985816000	3.248382000
H	3.208439000	3.992919000	3.005675000
H	3.938441000	5.466371000	2.335010000
C	5.520331000	7.708327000	1.136533000
H	6.297130000	8.086558000	0.462209000
H	4.571268000	7.704639000	0.598531000
H	5.781122000	6.694310000	1.426467000
C	2.903753000	7.610115000	5.809442000
C	2.547888000	5.846594000	3.989496000
H	2.080166000	6.589716000	3.346314000
H	1.766251000	5.245857000	4.462386000
H	2.243622000	7.276625000	6.614486000
H	2.354925000	8.303646000	5.172813000
H	3.751885000	8.141612000	6.236960000
Cl	9.718275000	5.347450000	8.336278000
Cl	11.288878000	6.231420000	5.736721000
C	9.484747000	7.116858000	3.361122000
C	9.405337000	8.615917000	3.156034000
C	8.975421000	9.136873000	1.938004000
C	8.887548000	10.509729000	1.742490000
C	9.226540000	11.383293000	2.769625000
C	9.670127000	10.872549000	3.984589000
C	9.761656000	9.499267000	4.173713000
H	9.165400000	12.453155000	2.618104000
H	9.956691000	11.545027000	4.783151000
H	10.115747000	9.112132000	5.121331000
H	8.707664000	8.459262000	1.136338000
H	8.565416000	10.898515000	0.784558000
H	10.524962000	6.795631000	3.343889000
H	8.971991000	6.626890000	2.531827000
H	5.112545000	6.487627000	6.970626000

[L5a+BnD]$^{+}$
E [Eh]: −2158.407854989108

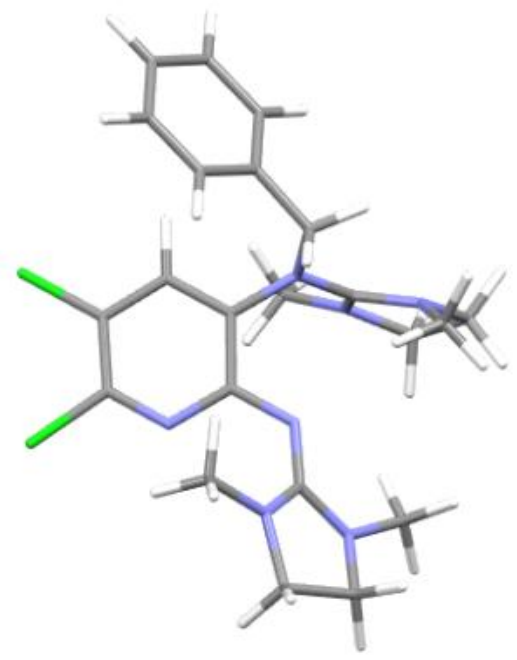

N	6.906213000	6.286483000	6.240919000
N	5.304568000	10.063832000	4.390602000
N	5.167716000	9.286063000	2.321316000
N	5.510905000	6.722824000	4.393810000
N	7.139565000	8.816443000	3.581592000
N	5.449307000	4.307330000	4.387537000
N	3.560558000	5.444658000	4.308682000
C	7.952041000	6.583667000	6.978515000
C	5.912994000	9.348773000	3.438544000
C	8.812193000	7.649987000	6.697055000
C	4.915241000	5.551338000	4.399443000
C	6.620065000	6.991467000	5.131692000
C	8.522656000	8.416002000	5.576232000
C	7.437530000	8.094045000	4.782882000
C	3.941350000	10.413186000	3.986584000
H	3.752124000	11.465397000	4.191911000
H	3.209690000	9.816501000	4.536477000
C	3.955472000	10.103747000	2.481780000
H	3.083245000	9.534046000	2.166891000
H	4.016640000	11.007242000	1.872469000
C	5.880421000	10.522596000	5.641290000
H	5.738617000	9.794447000	6.441671000
H	5.394375000	11.456413000	5.918774000
H	6.943574000	10.707123000	5.514974000
C	6.846724000	3.951748000	4.477225000
H	7.006040000	3.035583000	3.907688000
H	7.164024000	3.800049000	5.510704000

H	6.176031000	3.796396000	4.768722000
C	3.371804000	5.079167000	3.355853000
H	2.803981000	4.154487000	3.485341000
H	3.575137000	5.210060000	2.292650000
C	5.464805000	7.552803000	1.168433000
H	6.253413000	7.899758000	0.490583000
H	4.516161000	7.570056000	0.630824000
H	5.692989000	6.534780000	1.471301000
C	3.208513000	7.746509000	5.974253000
C	2.656673000	6.297362000	3.968085000
H	2.653254000	7.151317000	3.285031000
H	1.628489000	6.079849000	4.258064000
H	2.193285000	7.686929000	6.368391000
H	3.310003000	8.680534000	5.412907000
H	3.910545000	7.746373000	6.803692000
Cl	9.776264000	5.280987000	8.327069000
Cl	11.394017000	6.279512000	5.750520000
C	9.571201000	7.149091000	3.374545000
C	9.441096000	8.648061000	3.193291000
C	8.954194000	9.172378000	1.998480000
C	8.815472000	10.544156000	1.828527000
C	9.159835000	11.412770000	2.858190000
C	9.659522000	10.899064000	4.049631000
C	9.801672000	9.526696000	4.213353000
H	9.058051000	12.482114000	2.726567000
H	9.948868000	11.568520000	4.849619000
H	10.200635000	9.138425000	5.142799000
H	8.680405000	8.498560000	1.195843000
H	8.447866000	10.936200000	0.888558000
H	10.621557000	6.863057000	3.367522000
H	9.084789000	6.655108000	2.531907000
H	7.187236000	5.393900000	7.710906000

[L5a+BnE]$^{+}$
E [Eh]: −2158.409611179826

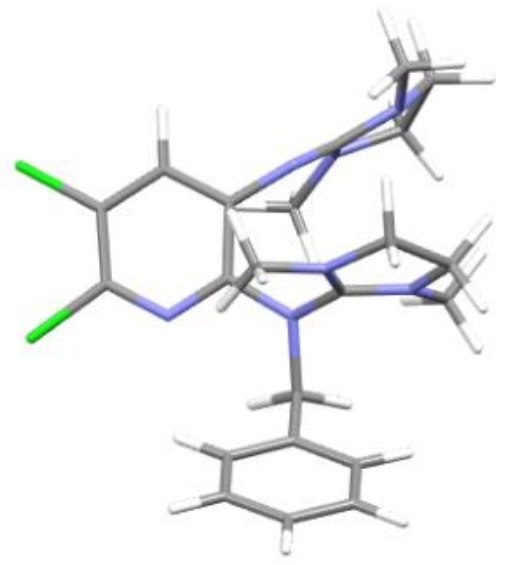

N	6.933605000	8.479397000	7.868035000
N	4.518684000	10.150853000	4.062234000
N	4.143528000	8.357668000	2.783666000
N	4.915936000	7.597567000	7.067983000
N	5.769231000	8.105405000	4.422187000
N	5.214105000	5.518065000	5.922597000
N	3.243419000	6.494135000	5.804416000
C	8.103033000	9.068786000	7.708263000
C	4.897914000	8.849671000	3.833134000
C	8.605592000	9.366820000	6.438111000
C	4.490883000	6.574850000	6.268292000
C	6.185704000	8.182920000	6.822436000
C	7.818773000	9.057975000	5.335590000
C	6.561017000	8.478401000	5.495746000
C	3.350890000	10.481034000	3.246552000
H	3.400540000	11.509944000	2.893291000
H	2.426488000	10.357254000	3.825385000
C	3.447162000	9.456943000	2.123198000
H	2.474973000	9.142272000	1.745714000
H	4.038903000	9.841056000	1.283062000
C	4.711001000	10.874669000	5.304797000
H	4.085149000	10.483424000	6.115490000
H	4.439192000	11.915360000	5.135890000
H	5.748925000	10.847384000	5.619020000
C	6.579547000	5.222198000	6.307450000
H	7.280282000	5.559605000	5.540639000

H	7.456816000	4.738606000	4.040561000
C	4.401809000	3.293181000	4.482285000
H	4.305811000	2.935638000	5.512531000
H	4.626809000	2.444395000	3.838040000
C	5.222835000	8.208705000	1.343956000
H	5.692821000	8.525691000	0.412037000
H	4.204614000	7.888345000	1.124626000
H	5.755979000	7.362804000	1.767817000
C	2.683306000	6.558579000	4.063222000
C	3.161519000	4.071054000	4.031973000
H	2.958376000	3.932495000	2.962880000
H	2.267287000	3.802087000	4.592423000
H	1.731864000	6.398361000	4.571243000
H	2.485011000	6.694076000	2.992169000
H	3.152500000	7.456704000	4.455293000
Cl	8.237830000	5.569624000	8.356297000
Cl	10.182137000	8.038495000	7.675025000
C	8.038218000	8.623246000	2.412076000
C	9.435129000	9.090736000	2.713938000
C	9.703681000	10.453318000	2.841877000
C	10.974453000	10.889292000	3.188123000
C	11.992100000	9.963725000	3.405428000
C	11.734991000	8.606130000	3.266384000
C	10.459311000	8.172668000	2.921619000
H	12.982750000	10.302778000	3.677749000
H	12.523561000	7.884157000	3.431799000
H	10.256366000	7.112631000	2.828769000
H	8.911850000	11.175568000	2.678148000
H	11.175772000	11.948145000	3.284236000
H	8.046060000	7.569595000	2.128762000
H	7.622628000	9.200339000	1.589281000
H	9.152429000	9.257911000	5.331800000

[L5a+Bn(*p*-CF_3)E]$^{+}$
E [Eh]: −2495.484253210602

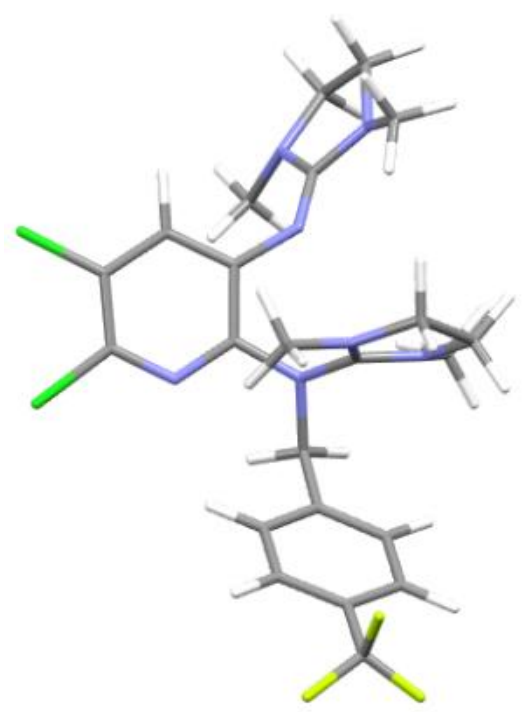

N	7.236024000	6.052610000	6.357042000
N	4.401691000	9.993384000	4.832947000
N	3.792232000	9.326781000	2.788893000
N	5.182397000	5.465304000	5.398910000
N	5.181006000	7.873690000	3.960317000
N	5.449445000	4.899731000	3.093585000
N	3.402609000	5.029224000	3.901889000
C	8.238854000	6.879598000	6.574503000
C	4.529281000	8.994144000	3.895338000
C	8.313739000	8.119050000	5.927520000
C	4.703889000	5.171646000	4.159832000
C	6.255029000	6.386119000	5.538491000
C	7.289828000	8.469307000	5.061844000
C	6.200079000	7.615505000	4.850050000
C	3.397072000	10.961161000	4.380269000
H	3.688498000	11.977948000	4.638772000
H	2.431062000	10.743760000	4.851375000
C	3.347070000	10.713053000	2.877735000
H	2.350005000	10.840348000	2.457812000
H	4.038302000	11.370112000	2.336226000
C	4.448481000	9.749270000	6.265646000
H	3.541053000	9.239983000	6.612011000
H	4.521665000	10.708145000	6.776717000
H	5.313768000	9.156714000	6.538821000
C	6.896831000	4.856285000	3.019977000
H	7.300936000	5.820315000	2.703525000

H	6.675564000	4.145052000	6.435801000
H	6.810198000	5.702042000	7.253286000
C	4.474389000	4.671709000	4.983642000
H	4.529334000	3.631948000	5.301298000
H	4.902944000	4.755431000	3.983118000
C	4.628748000	7.258414000	1.974888000
H	5.279895000	7.609244000	1.165748000
H	3.785338000	6.725758000	1.534257000
H	5.200634000	6.584067000	2.605571000
C	2.258890000	7.551710000	5.811680000
C	3.045865000	5.242408000	5.068053000
H	2.623638000	5.455000000	4.086798000
H	2.363072000	4.584546000	5.609240000
H	1.873070000	7.688724000	4.800929000
H	2.731725000	8.475512000	6.133076000
H	1.428892000	7.318053000	6.482164000
Cl	8.996130000	9.438734000	9.141873000
Cl	10.150100000	10.099956000	6.211985000
C	4.289257000	7.697372000	8.419203000
C	4.412450000	6.409737000	9.189208000
C	3.369106000	5.485785000	9.177236000
C	3.501397000	4.260587000	9.821397000
C	4.681828000	3.952678000	10.487000000
C	5.723060000	4.875649000	10.513944000
C	5.591595000	6.097572000	9.867399000
H	4.785636000	3.003133000	10.995404000
H	6.636378000	4.645409000	11.046983000
H	6.400541000	6.816397000	9.882411000
H	2.440269000	5.730679000	8.674388000
H	2.681387000	3.554190000	9.815111000
H	4.802493000	8.513608000	8.917719000
H	3.241343000	7.970168000	8.297124000
H	8.173496000	9.287269000	4.340416000

H	7.182437000	4.091412000	2.299625000
H	7.310008000	4.589739000	3.987721000
C	4.604886000	4.697384000	1.913815000
H	4.919905000	3.800450000	1.383639000
H	4.686175000	5.549641000	1.235770000
C	4.066821000	8.754319000	1.489448000
H	4.809331000	9.343499000	0.938773000
H	3.149054000	8.715803000	0.901932000
H	4.454241000	7.749463000	1.627958000
C	2.318162000	5.513909000	4.727069000
C	3.200181000	4.556948000	2.528416000
H	2.460379000	5.179099000	2.026780000
H	2.846512000	3.524070000	2.528866000
H	1.766734000	4.692661000	5.190075000
H	1.629656000	6.091891000	4.109557000
H	2.721282000	6.163697000	5.499422000
Cl	9.629249000	9.205587000	6.180901000
H	7.323186000	9.425460000	4.558439000
C	4.864327000	4.512617000	6.498392000
C	5.210672000	3.098281000	6.115236000
C	6.542100000	2.680617000	6.090031000
C	6.867302000	1.403598000	5.660581000
C	5.861509000	0.531303000	5.250674000
C	4.532079000	0.931494000	5.286474000
C	4.212871000	2.212072000	5.719814000
H	3.752216000	0.244559000	4.989096000
H	3.174111000	2.516668000	5.760602000
H	7.318591000	3.363135000	6.411228000
H	7.898679000	1.078493000	5.653487000
Cl	9.462151000	6.362319000	7.681093000
H	5.450723000	4.846834000	7.348967000
H	3.808514000	4.593130000	6.754971000
C	6.224289000	-0.832310000	4.722307000
F	5.212231000	-1.707928000	4.846345000
F	7.292551000	-1.348605000	5.352404000
F	6.533868000	-0.770124000	3.404576000

[L5b+BnE]$^+$
E [Eh]: −2765.422650328976

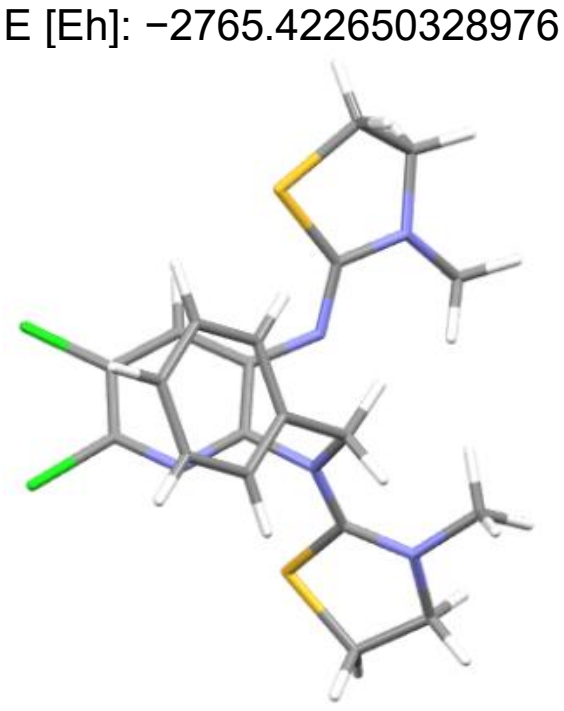

Cl	9.656644000	1.837624000	14.304743000
S	7.414913000	7.199753000	11.971254000
N	5.455741000	5.431909000	12.357239000
C	6.520051000	4.556553000	12.784599000
Cl	8.665694000	4.395024000	15.973439000
S	5.758540000	0.657627000	11.152205000
N	4.961947000	7.408122000	11.140246000
C	6.854835000	3.471798000	11.965130000
N	6.250507000	3.335259000	10.733601000
C	7.865424000	2.637014000	12.464660000
H	8.216780000	1.797394000	11.882726000
N	5.274843000	2.090351000	9.039976000
C	8.434571000	2.890463000	13.700384000
N	7.063488000	4.806880000	13.958846000
C	7.999010000	4.011292000	14.428209000
C	5.777602000	6.595202000	11.820003000
C	6.871752000	8.856519000	11.423149000
H	6.686678000	9.459642000	12.310125000
H	7.659427000	9.316152000	10.831993000

[L5b+Bn(p-CF$_3$)E]$^+$
E [Eh]: −3102.503190042379

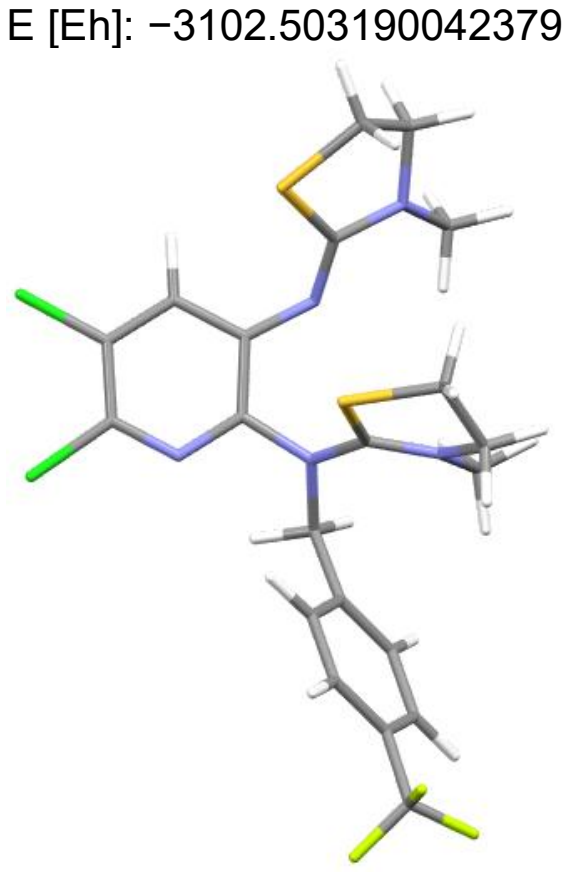

Cl	11.358004000	1.018677000	12.632902000
S	5.005718000	2.220720000	13.005722000
N	6.577800000	4.394130000	12.887465000
C	7.708994000	3.521965000	12.791208000
Cl	10.579829000	2.732439000	15.231957000
S	6.914301000	0.298156000	10.048101000
N	4.318545000	4.581942000	12.221658000
C	7.938767000	2.818556000	11.595609000
N	7.095955000	3.002007000	10.519450000
C	9.102336000	2.038244000	11.577445000
H	9.379142000	1.499616000	10.682709000
N	5.839576000	2.256990000	8.720040000
C	9.932296000	1.981239000	12.684595000
N	8.504156000	3.478114000	13.842364000
C	9.590815000	2.736078000	13.819401000
C	5.366025000	3.891710000	12.649508000
C	3.354340000	2.399558000	12.244872000
H	3.414109000	2.085815000	11.203370000
H	2.648029000	1.771287000	12.780742000

C	5.609493000	8.620993000	10.614498000
H	4.921919000	9.463165000	10.691911000
H	5.839349000	8.455800000	9.557372000
C	3.742927000	6.987060000	10.442535000
H	3.739377000	7.464845000	9.463618000
H	2.845504000	7.293598000	10.978697000
H	3.738917000	5.912123000	10.295225000
C	5.812778000	2.214435000	10.280845000
C	4.740422000	-0.092672000	9.833344000
H	5.012353000	-1.139303000	9.725182000
H	3.690182000	-0.016003000	10.110245000
C	5.058066000	0.722502000	8.589011000
H	5.954881000	0.340205000	8.086513000
H	4.231218000	0.697452000	7.878479000
C	5.426386000	3.140781000	8.049982000
H	5.447933000	4.101512000	8.556379000
H	4.586337000	3.106359000	7.356928000
H	6.357364000	3.024223000	7.485251000
C	4.062430000	4.951875000	12.631612000
C	3.999355000	4.244749000	13.954558000
C	4.057783000	2.854673000	14.008435000
C	3.942656000	4.976395000	15.138654000
C	3.949696000	4.326212000	16.363899000
C	4.016195000	2.937398000	16.412883000
C	4.067359000	2.201903000	15.234855000
H	4.110939000	2.279994000	13.091978000
H	3.909980000	6.058843000	15.103161000
H	3.907517000	4.899947000	17.280053000
H	4.115605000	1.121529000	15.270210000
H	3.415101000	5.823517000	12.640282000
H	3.762160000	4.282514000	11.826139000
H	4.025505000	2.430226000	17.368620000

C	3.036796000	3.882392000	12.382919000
H	2.342234000	4.223770000	11.617658000
H	2.619767000	4.111633000	13.366446000
C	4.381824000	5.880382000	11.562308000
H	5.355071000	5.995817000	11.094044000
H	4.202466000	6.695740000	12.262534000
H	3.618344000	5.903721000	10.787043000
C	6.646735000	2.040284000	9.795984000
C	5.711347000	-0.122581000	8.738772000
H	6.053339000	-1.009458000	8.211869000
H	4.743713000	-0.324898000	9.195739000
C	5.669271000	1.105667000	7.842410000
H	6.471723000	1.073892000	7.095490000
H	4.716625000	1.181559000	7.317398000
C	5.703035000	3.588502000	8.156041000
H	4.760071000	3.652890000	7.614187000
H	6.523430000	3.819456000	7.468597000
H	5.716011000	4.317027000	8.961565000
C	6.794327000	5.747781000	13.460645000
C	5.842607000	6.082893000	14.580044000
C	5.262757000	7.346070000	14.640091000
C	5.578925000	5.163683000	15.596600000
C	4.736027000	5.501528000	16.644003000
C	4.154514000	6.766894000	16.689922000
C	4.423786000	7.692826000	15.691484000
H	5.474534000	8.075659000	13.867619000
H	6.047862000	4.188397000	15.578824000
H	4.534270000	4.786137000	17.429758000
H	3.979053000	8.676859000	15.731030000
C	3.270241000	7.137796000	17.854180000
F	4.000110000	7.438521000	18.946568000
F	2.448662000	6.124507000	18.194016000
F	2.501256000	8.206065000	17.579341000
H	7.813545000	5.716117000	13.841744000
H	6.758424000	6.503690000	12.678359000

[L6+Ha+BnB]$^{+}$
E [Eh]: −2158.423861073028

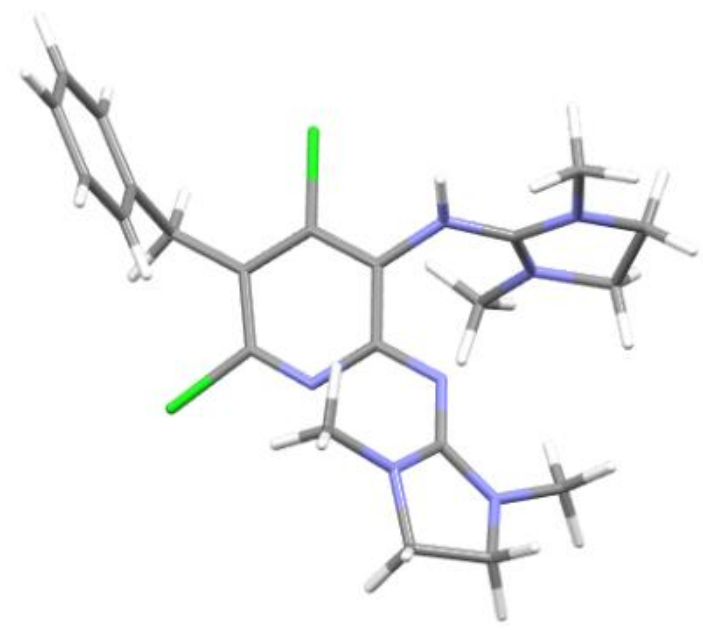

N	4.741160000	5.469045000	6.059265000
N	4.755942000	9.763846000	4.625445000
N	5.304623000	9.634207000	2.488222000
N	3.818467000	6.743559000	4.308507000
N	6.259659000	8.050167000	3.932563000
N	2.828968000	4.598091000	3.817436000
N	1.533399000	6.376169000	3.976031000
C	5.761638000	5.179225000	6.834134000
C	5.456768000	9.092627000	3.708397000
C	7.031427000	5.768210000	6.785519000
C	2.813333000	5.926175000	4.079583000
C	4.869186000	6.393210000	5.091999000
C	7.151596000	6.753241000	5.801182000
C	6.096784000	7.082086000	4.959404000
C	3.899849000	10.752955000	3.962372000
H	3.888334000	11.681288000	4.529563000
H	2.877729000	10.375207000	3.877170000
C	4.572101000	10.898675000	2.593523000
H	3.857213000	10.993659000	1.778602000
H	5.262185000	11.747410000	2.561277000
C	4.647484000	9.488580000	6.047596000
H	3.901878000	8.718760000	6.251265000
H	4.356751000	10.412930000	6.543394000
H	5.608641000	9.175400000	6.444753000

[L6+Hb+BnB]$^{+}$
E [Eh]: −2158.421307075634

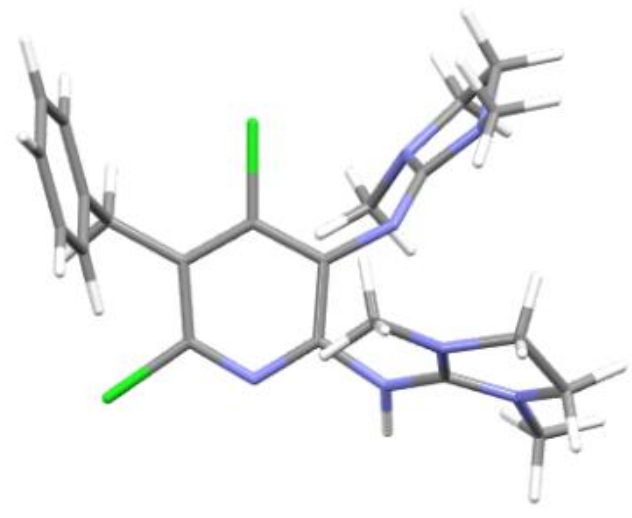

N	4.603338000	5.357136000	7.242032000
N	5.307163000	9.682237000	4.742004000
N	5.045946000	9.046423000	2.621531000
N	3.003082000	5.930053000	5.672718000
N	4.944006000	7.352823000	4.210471000
N	3.188740000	4.993584000	3.487442000
N	1.415300000	6.220040000	3.966023000
C	5.825580000	5.384127000	7.731040000
C	5.107696000	8.605974000	3.919155000
C	6.911432000	6.003405000	7.093983000
C	2.576804000	5.724466000	4.419186000
C	4.354911000	5.988198000	6.111394000
C	6.600988000	6.684028000	5.914794000
C	5.290122000	6.748470000	5.391647000
C	5.205426000	10.922928000	3.972880000
H	5.936061000	11.655288000	4.313509000
H	4.202751000	11.354588000	4.080008000
C	5.457694000	10.444042000	2.547577000
H	4.878702000	10.991654000	1.804805000
H	6.519536000	10.514462000	2.282488000
C	4.912808000	9.725486000	6.135948000
H	3.826920000	9.836448000	6.245695000
H	5.394113000	10.582437000	6.605188000
H	5.233383000	8.833197000	6.662665000

C	3.973365000	3.717359000	3.831525000	C	4.441082000	4.267960000	3.600629000
H	4.866385000	4.267715000	3.544208000	H	5.289367000	4.905756000	3.350159000
H	3.808353000	2.925672000	3.100119000	H	4.402372000	3.422996000	2.915346000
H	4.137003000	3.276395000	4.816852000	H	4.560073000	3.884898000	4.610460000
C	1.468695000	4.075831000	3.712389000	C	2.498521000	5.159216000	2.204455000
H	1.165099000	3.603840000	4.652428000	H	2.455394000	4.210356000	1.674328000
H	1.398573000	3.339432000	2.913127000	H	3.024490000	5.890387000	1.584892000
C	6.042596000	9.255427000	1.301456000	C	5.342115000	8.169056000	1.509749000
H	7.042908000	9.698693000	1.275959000	H	5.017978000	7.163452000	1.760374000
H	5.491229000	9.595676000	0.427033000	H	6.416174000	8.144389000	1.292165000
H	6.120683000	8.169982000	1.240086000	H	4.813172000	8.512499000	0.620301000
C	1.169085000	7.768759000	3.958529000	C	0.449837000	6.967837000	4.742633000
C	0.661273000	5.345215000	3.427433000	C	1.118636000	5.663633000	2.643217000
H	0.506151000	5.498476000	2.352435000	H	0.722250000	6.435931000	1.986946000
H	-0.308764000	5.349355000	3.922431000	H	0.386476000	4.854402000	2.721283000
H	0.188846000	7.898112000	4.418274000	H	-0.249931000	6.313720000	5.271386000
H	1.130600000	8.168181000	2.937338000	H	-0.117201000	7.610610000	4.071273000
H	1.907543000	8.324611000	4.529378000	H	0.966910000	7.603167000	5.460637000
Cl	5.449969000	3.913782000	8.002213000	Cl	6.041592000	4.556831000	9.249841000
Cl	8.655852000	7.595704000	5.600140000	Cl	7.859054000	7.489613000	5.038608000
C	8.186292000	5.373334000	7.663645000	C	8.325454000	5.902536000	7.599953000
C	9.220142000	4.520762000	6.951788000	C	9.140087000	4.879523000	6.829138000
C	10.578023000	4.765345000	7.140648000	C	10.375732000	5.224738000	6.288665000
C	11.536267000	3.974657000	6.517521000	C	11.134860000	4.288032000	5.596602000
C	11.145770000	2.928696000	5.690272000	C	10.662282000	2.991762000	5.433611000
C	9.792298000	2.677433000	5.494669000	C	9.428564000	2.637985000	5.969497000
C	8.837353000	3.468544000	6.120996000	C	8.673191000	3.575519000	6.661816000
H	11.890128000	2.312091000	5.203684000	H	11.252515000	2.260479000	4.897047000
H	9.479914000	1.859733000	4.857554000	H	9.058197000	1.627046000	5.855977000
H	7.786090000	3.257796000	5.968064000	H	7.720507000	3.284490000	7.086456000
H	10.888725000	5.583504000	7.779622000	H	10.746232000	6.235495000	6.408674000
H	12.587430000	4.178002000	6.676757000	H	12.094822000	4.572132000	5.185061000
H	8.672094000	6.272518000	8.044936000	H	8.807281000	6.876629000	7.520469000
H	7.801064000	4.834181000	8.528250000	H	8.305041000	5.638613000	8.656097000
H	6.944334000	7.850185000	3.219486000	H	2.307014000	6.022704000	6.396881000

$[L6+Hc+BnB]^+$
E [Eh]: −2158.425940621236

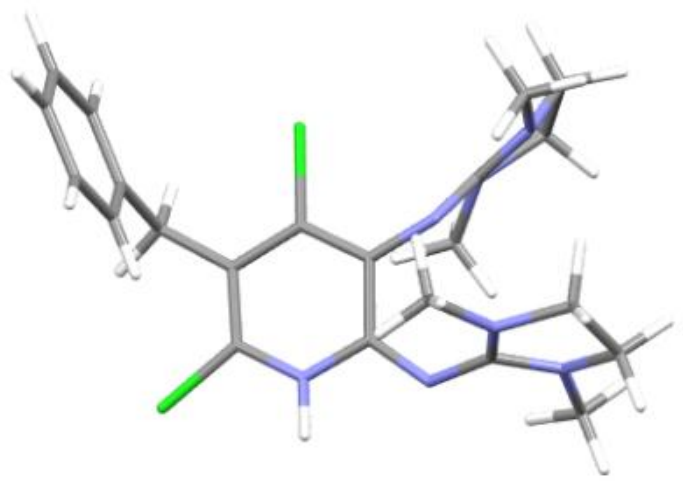

$[L6+BnD]^+$
E [Eh]: −2158.414150828355

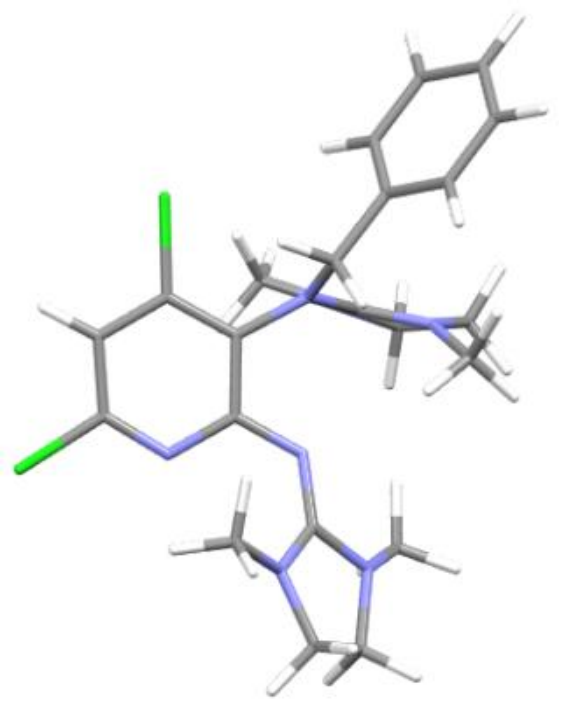

N	4.674166000	5.200293000	7.205737000	N	7.115924000	6.970983000	4.749301000
N	5.141670000	9.401977000	4.561551000	N	4.848483000	10.862004000	4.344520000
N	5.020461000	8.666703000	2.458482000	N	4.316816000	10.563921000	2.221975000
N	3.056502000	5.719061000	5.687308000	N	5.380738000	7.661612000	3.315700000
N	5.044600000	7.033103000	4.109057000	N	6.565169000	10.115119000	2.900075000
N	2.833972000	5.079917000	3.397137000	N	5.534873000	5.315354000	2.786553000
N	1.584135000	6.728171000	4.164919000	N	3.542374000	6.266016000	2.986333000
C	5.919145000	5.238290000	7.761183000	C	8.239134000	7.251384000	5.368454000
C	5.084977000	8.282046000	3.772279000	C	5.286310000	10.479810000	3.139236000
C	6.949993000	5.879738000	7.137767000	C	8.932089000	8.452143000	5.268805000
C	2.570213000	5.860712000	4.462215000	C	4.892377000	6.467404000	3.076168000
C	4.329182000	5.781118000	6.024232000	C	6.565092000	7.889829000	3.932967000
C	6.626650000	6.510009000	5.901129000	C	8.358820000	9.417834000	4.453635000
C	5.362052000	6.500292000	5.326818000	C	7.173178000	9.165944000	3.772946000
C	4.911626000	10.592853000	3.740699000	C	3.402712000	11.097098000	4.313196000
H	5.530108000	11.423935000	4.076122000	H	3.163421000	12.018616000	4.840193000
H	3.858683000	10.895417000	3.795855000	H	2.868291000	10.272339000	4.792193000
C	5.275698000	10.098725000	2.345256000	C	3.125661000	11.193379000	2.806780000
H	4.669693000	10.550542000	1.560860000	H	2.232600000	10.648221000	2.508323000
H	6.332856000	10.280989000	2.117917000	H	3.035040000	12.227052000	2.466307000
C	4.679871000	9.433014000	5.935891000	C	5.640617000	11.064345000	5.542404000
H	3.595173000	9.288874000	6.005777000	H	5.742423000	10.143447000	6.119659000

H	4.927288000	10.405228000	6.358473000
H	5.176464000	8.674786000	6.532942000
C	3.938406000	4.153919000	3.294427000
H	4.849672000	4.652887000	2.957220000
H	3.671527000	3.364842000	2.592597000
H	4.121777000	3.699602000	4.265936000
C	2.135850000	5.582534000	2.216157000
H	1.743262000	4.761915000	1.617940000
H	2.817760000	6.175198000	1.598886000
C	5.441633000	7.787330000	1.388251000
H	5.228020000	6.760674000	1.669978000
H	6.516120000	7.879551000	1.193625000
H	4.899080000	8.034710000	0.475397000
C	0.877244000	7.536679000	5.131206000
C	1.035707000	6.450761000	2.839532000
H	0.861784000	7.373313000	2.287756000
H	0.086555000	5.911648000	2.927824000
H	-0.113720000	7.122168000	5.339091000
H	0.758213000	8.552488000	4.751281000
H	1.448973000	7.559166000	6.054902000
Cl	6.048016000	4.400945000	9.258758000
Cl	7.879153000	7.362911000	5.070112000
C	8.348817000	5.892996000	7.691086000
C	9.294606000	4.964825000	6.952155000
C	10.579558000	5.391748000	6.627875000
C	11.468010000	4.539903000	5.983144000
C	11.077988000	3.248391000	5.650062000
C	9.795999000	2.814648000	5.967505000
C	8.911051000	3.668511000	6.613930000
H	11.768349000	2.583954000	5.147325000
H	9.486087000	1.808673000	5.715091000
H	7.916270000	3.316703000	6.859435000
H	10.887304000	6.399825000	6.878881000
H	12.463675000	4.886703000	5.738564000
H	8.739579000	6.909707000	7.647677000
H	8.312826000	5.615617000	8.744582000
H	3.933942000	4.721012000	7.701284000

H	5.145355000	11.814471000	6.156326000
H	6.629016000	11.431569000	5.279346000
C	6.930905000	5.195959000	2.417518000
H	7.322645000	6.162428000	2.107007000
H	7.012201000	4.505177000	1.577105000
H	7.531172000	4.828776000	3.249668000
C	4.587657000	4.260404000	2.428194000
H	4.839399000	3.321057000	2.919283000
H	4.601757000	4.099866000	1.345079000
C	4.266689000	9.841786000	0.962680000
H	4.607301000	10.457410000	0.130591000
H	3.234188000	9.547170000	0.781434000
H	4.865163000	8.938437000	1.044667000
C	2.587975000	7.157193000	3.604315000
C	3.252391000	4.837517000	2.903445000
H	2.433375000	4.636968000	2.213571000
H	2.976164000	4.451072000	3.891187000
H	1.652580000	7.138024000	3.044058000
H	2.998923000	8.162397000	3.591579000
H	2.379488000	6.872557000	4.642450000
H	9.852032000	8.629942000	5.801456000
Cl	9.139931000	10.960782000	4.311807000
C	7.222179000	10.397813000	1.603810000
C	6.950029000	11.785783000	1.081387000
C	6.910732000	12.889358000	1.933202000
C	6.694708000	14.163172000	1.424043000
C	6.524842000	14.351299000	0.056445000
C	6.575647000	13.258497000	-0.799651000
C	6.782713000	11.982854000	-0.287136000
H	6.360637000	15.344775000	-0.339311000
H	6.452937000	13.396821000	-1.865811000
H	6.823124000	11.134705000	-0.960907000
H	7.062315000	12.755001000	2.995695000
H	6.669763000	15.012545000	2.094399000
Cl	8.897190000	6.001894000	6.388624000
H	8.287775000	10.280059000	1.797694000
H	6.953307000	9.648548000	0.857828000

[L6+BnE]+
E [Eh]: −2158.414752738703

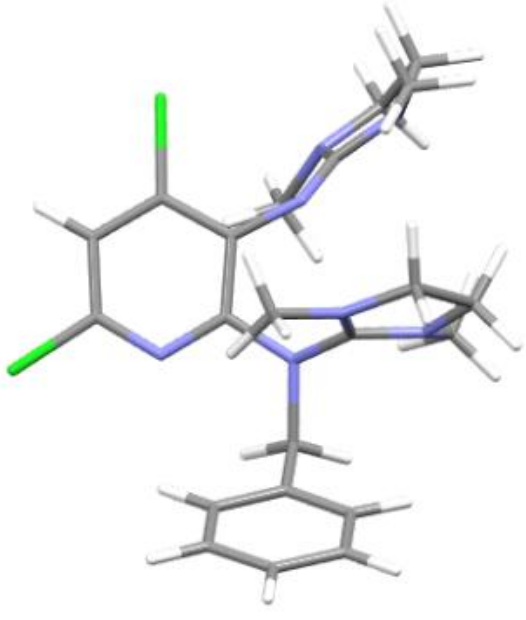

N	7.201491000	5.957463000	6.388318000
N	4.239472000	9.721913000	4.914021000
N	3.766777000	9.173611000	2.802331000
N	5.161496000	5.483216000	5.354167000
N	5.339411000	7.837617000	3.870434000
N	5.328923000	4.944219000	3.031787000
N	3.325963000	5.356659000	3.857329000
C	8.233288000	6.751622000	6.607034000
C	4.535054000	8.851783000	3.899187000
C	8.422915000	7.957365000	5.950876000
C	4.636697000	5.301888000	4.100343000
C	6.281342000	6.333458000	5.517900000
C	7.446377000	8.344801000	5.042398000
C	6.311370000	7.552679000	4.803519000
C	3.104951000	10.564585000	4.534142000
H	3.235553000	11.582362000	4.898800000
H	2.174423000	10.161658000	4.953237000
C	3.122476000	10.466741000	3.013504000
H	2.127317000	10.493539000	2.571449000
H	3.723415000	11.268441000	2.567786000

[L6+Bn(*p*-CF_3)E]+
E [Eh]: −2495.489091059087

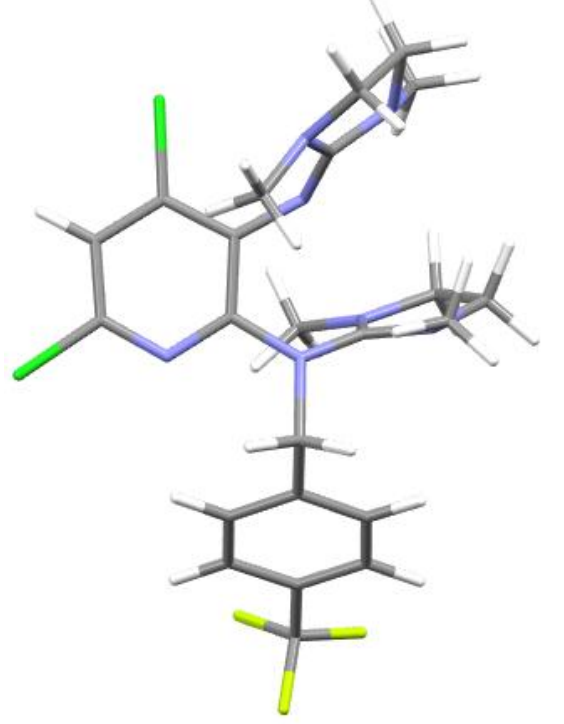

Cl	4.823354000	1.934111000	7.410394000
Cl	6.422377000	3.304889000	12.379241000
N	2.123928000	4.943266000	10.586573000
N	2.244357000	6.589072000	8.860243000
N	0.272675000	5.708304000	9.312479000
N	2.414726000	3.681075000	8.112483000
N	0.903915000	2.960040000	6.500889000
N	1.435865000	1.469705000	8.075898000
N	4.197372000	4.116249000	11.273672000
C	3.292241000	4.189788000	10.314118000
C	3.383353000	3.513391000	9.077318000
C	4.569260000	2.782204000	8.896473000
C	5.531606000	2.702407000	9.894899000
H	6.436113000	2.131143000	9.755437000
C	5.277286000	3.382820000	11.075518000
C	1.578724000	5.707358000	9.586514000
C	1.372838000	7.207026000	7.859077000
H	1.713000000	6.957724000	6.853214000
H	1.399296000	8.290455000	7.975396000
C	-0.010806000	6.608459000	8.188180000

C	4.409737000	9.423089000	6.323437000
H	3.681767000	8.680847000	6.672259000
H	4.261218000	10.341620000	6.888630000
H	5.410125000	9.060512000	6.532102000
C	6.752021000	4.679930000	2.956650000
H	7.281068000	5.548766000	2.559302000
H	6.913174000	3.825313000	2.300808000
H	7.136163000	4.433896000	3.941277000
C	4.464116000	4.852821000	1.854441000
H	4.563916000	3.864166000	1.406817000
H	4.750873000	5.599876000	1.113107000
C	4.190880000	8.820983000	1.463207000
H	4.825803000	9.600169000	1.026364000
H	3.318572000	8.684243000	0.822917000
H	4.762873000	7.899002000	1.509223000
C	2.351644000	6.022660000	4.696072000
C	3.057311000	5.099006000	2.438587000
H	2.574937000	5.972286000	1.997808000
H	2.395576000	4.240465000	2.321371000
H	1.499895000	5.368534000	4.885448000
H	2.003471000	6.932513000	4.200950000
H	2.817054000	6.290774000	5.639914000
H	9.289550000	8.572253000	6.137080000
Cl	7.621429000	9.850606000	4.207974000
C	4.891820000	4.390981000	6.337646000
C	5.431556000	3.061544000	5.878433000
C	6.749803000	2.692871000	6.148736000
C	7.256157000	1.495642000	5.658717000
C	6.453881000	0.656707000	4.891830000
C	5.135899000	1.011368000	4.628337000
C	4.628454000	2.207053000	5.123545000
H	6.849799000	-0.277044000	4.514509000
H	4.500563000	0.350660000	4.052724000
H	3.594093000	2.471351000	4.933590000
H	7.375010000	3.352456000	6.735132000
H	8.277234000	1.213923000	5.880720000
Cl	9.403096000	6.213952000	7.773647000
H	5.351345000	4.710466000	7.267275000
H	3.812870000	4.334560000	6.481313000

H	-0.742396000	7.364695000	8.472662000
H	-0.416058000	6.028109000	7.358322000
C	3.645860000	6.942347000	8.974941000
H	4.015665000	6.679225000	9.960726000
H	3.745634000	8.019217000	8.843968000
H	4.233288000	6.428652000	8.211150000
C	-0.649718000	4.651552000	9.673493000
H	-0.942970000	4.098500000	8.777798000
H	-1.540584000	5.066395000	10.145996000
H	-0.159528000	3.970850000	10.363072000
C	1.668726000	2.741465000	7.624213000
C	0.331810000	1.697289000	6.041325000
H	0.970655000	1.247600000	5.271962000
H	-0.664836000	1.838963000	5.625485000
C	0.334821000	0.869243000	7.320349000
H	-0.607635000	0.977934000	7.871585000
H	0.512907000	-0.190346000	7.144292000
C	1.288101000	3.948913000	5.514606000
H	1.800558000	4.763765000	6.017471000
H	0.400805000	4.327477000	5.005988000
H	1.965423000	3.524927000	4.764738000
C	1.610961000	1.050695000	9.454328000
H	2.611854000	1.269341000	9.810636000
H	1.465973000	-0.026923000	9.504596000
H	0.882900000	1.528163000	10.120828000
C	1.794030000	5.340041000	11.983774000
H	2.238093000	4.583650000	12.622863000
H	0.710613000	5.299158000	12.094663000
C	2.303748000	6.718094000	12.321621000
C	3.588959000	6.900906000	12.833171000
H	4.211310000	6.039117000	13.028221000
C	4.073285000	8.178455000	13.069569000
H	5.070279000	8.315577000	13.465600000
C	3.278198000	9.286797000	12.791523000
C	1.990958000	9.116584000	12.296381000
H	1.369456000	9.978506000	12.098588000
C	1.509302000	7.834474000	12.067079000
H	0.499552000	7.704447000	11.695243000
C	3.805866000	10.668285000	13.082534000
F	3.141408000	11.614706000	12.392761000
F	5.110666000	10.777483000	12.764254000
F	3.690772000	10.971523000	14.390768000

[L6+Bn(*o*-CF_3)E]$^+$
E [Eh]: −2495.483219470911

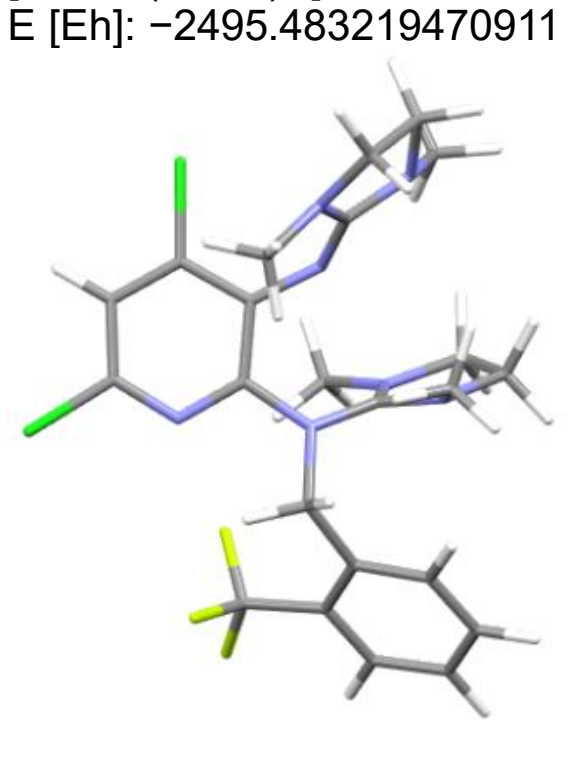

Cl	4.462326000	1.370211000	7.346988000
Cl	6.352068000	2.192823000	12.329739000
N	2.587987000	4.885268000	10.625336000
N	2.818493000	6.465392000	8.859890000
N	0.775172000	5.866128000	9.438492000
N	2.509791000	3.594191000	8.121058000
N	0.883493000	3.198563000	6.499340000
N	1.142857000	1.610421000	8.040824000
N	4.389012000	3.547493000	11.257981000
C	3.526484000	3.864782000	10.311553000
C	3.435337000	3.202489000	9.066419000
C	4.418665000	2.219577000	8.856730000
C	5.344257000	1.894321000	9.836968000
H	6.088686000	1.130854000	9.673196000
C	5.262147000	2.585329000	11.037447000

[L6+Bn(*o*,*p*-CF_3)$_2$E]$^+$
E [Eh]: −2832.551440981675

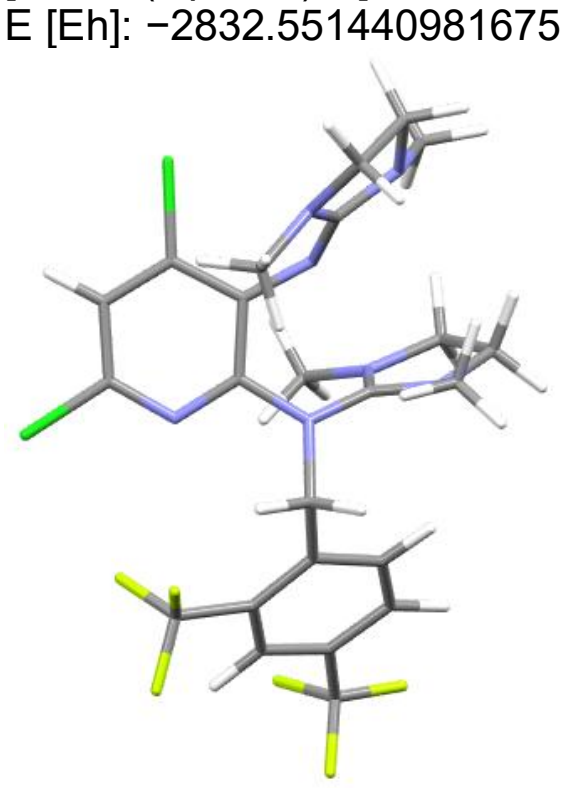

Cl	4.628020000	1.827767000	7.221754000
Cl	6.544962000	3.112128000	12.082832000
N	2.246771000	4.993593000	10.504158000
N	2.372890000	6.593907000	8.752674000
N	0.378233000	5.795501000	9.263836000
N	2.326925000	3.671743000	8.044205000
N	0.741325000	2.939540000	6.501569000
N	1.343786000	1.471371000	8.067958000
N	4.314796000	4.074875000	11.091405000
C	3.363636000	4.176613000	10.181392000
C	3.346114000	3.479803000	8.952894000
C	4.487441000	2.694627000	8.713708000
C	5.493635000	2.576439000	9.659986000
H	6.359967000	1.958826000	9.480908000
C	5.340082000	3.277255000	10.848236000

C	2.083841000	5.686649000	9.642573000
C	1.980205000	7.163050000	7.884559000
H	2.174247000	6.784107000	6.880026000
H	2.205716000	8.228448000	7.902763000
C	0.553207000	6.851841000	8.375921000
H	0.045317000	7.731687000	8.773370000
H	-0.066976000	6.413246000	7.594131000
C	4.263743000	6.499586000	8.773073000
H	4.698323000	6.157449000	9.705037000
H	4.582594000	7.525453000	8.592862000
H	4.605668000	5.864512000	7.952176000
C	-0.255205000	4.912454000	9.788100000
H	-0.620609000	4.421315000	8.882730000
H	-1.089878000	5.409960000	10.283358000
H	0.154908000	4.157958000	10.451368000
C	1.604621000	2.817834000	7.611266000
C	0.103427000	2.064568000	6.009103000
H	0.645968000	1.551548000	5.206181000
H	-0.863859000	2.382861000	5.622120000
C	-0.011625000	1.185013000	7.251185000
H	-0.940269000	1.377713000	7.801121000
H	0.037386000	0.121755000	7.019991000
C	1.441355000	4.118014000	5.528846000
H	2.149137000	4.767670000	6.034280000
H	0.647063000	4.714586000	5.077969000
H	1.970187000	3.583240000	4.731799000
C	1.361206000	1.060695000	9.366685000
H	2.305142000	0.519336000	9.430217000
H	0.553568000	0.364971000	9.586952000
H	1.356559000	1.842441000	10.127130000
C	2.216755000	5.139158000	12.048609000
H	2.999484000	4.655745000	12.623204000
H	1.274515000	4.637012000	12.276006000
C	2.082235000	6.606629000	12.389715000
C	3.146785000	7.422916000	12.811671000
C	2.933441000	8.772432000	13.073608000
H	3.759250000	9.384168000	13.404707000
C	1.670048000	9.333071000	12.937454000
C	0.603377000	8.531692000	12.562657000
H	-0.393951000	8.945213000	12.490380000
C	0.817529000	7.185261000	12.297912000
H	-0.023769000	6.559788000	12.032240000
C	4.530490000	6.864312000	13.055047000
F	4.508828000	5.850676000	13.941590000
F	5.377616000	7.786796000	13.533078000
F	5.087216000	6.382651000	11.916153000
H	1.521661000	10.382965000	13.150747000

[L7+Ha+BnA]+
E [Eh]: −1698.862887862939

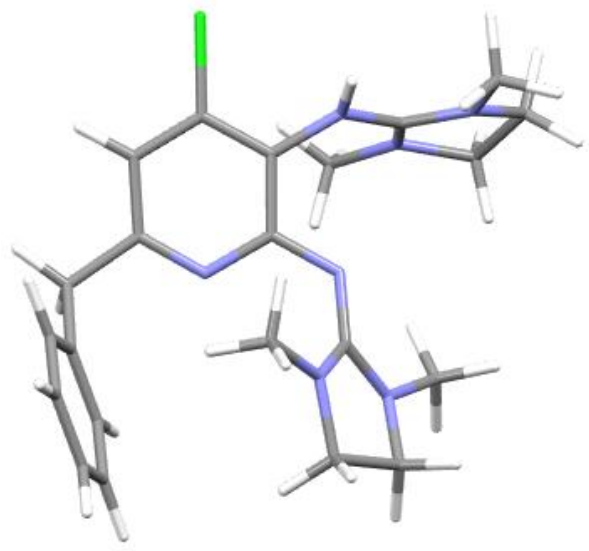

N	6.397617000	6.335082000	5.859819000
N	3.978965000	9.970991000	4.663804000
N	4.292509000	10.135544000	2.482079000
N	4.775177000	6.940986000	4.276486000
N	6.096099000	9.350961000	3.764101000
N	5.077495000	4.629938000	3.672470000
N	3.056629000	5.349741000	4.183385000
C	7.504553000	6.609064000	6.545301000
C	4.837116000	9.780606000	3.658055000
C	8.216931000	7.795935000	6.385959000

C	1.690744000	5.754339000	9.514073000
C	1.507712000	7.214684000	7.748984000
H	1.819997000	6.926252000	6.744519000
H	1.575232000	8.299456000	7.833351000
C	0.109839000	6.676839000	8.119812000
H	-0.588985000	7.467488000	8.393199000
H	-0.329826000	6.089146000	7.313267000
C	3.784626000	6.908548000	8.850360000
H	4.153648000	6.650556000	9.838463000
H	3.916478000	7.978675000	8.694123000
H	4.352682000	6.360958000	8.095571000
C	-0.560100000	4.751687000	9.626780000
H	-0.894662000	4.233314000	8.725331000
H	-1.424378000	5.172147000	10.141873000
H	-0.065989000	4.035377000	10.276073000
C	1.558986000	2.729912000	7.590673000
C	0.168752000	1.665176000	6.071925000
H	0.780910000	1.224343000	5.276148000
H	-0.846996000	1.789859000	5.699236000
C	0.239030000	0.834965000	7.349718000
H	-0.687281000	0.910199000	7.931212000
H	0.445591000	-0.217086000	7.159828000
C	1.087184000	3.914970000	5.488106000
H	1.619052000	4.736134000	5.959467000
H	0.181352000	4.287739000	5.008753000
H	1.735431000	3.481126000	4.718148000
C	1.679989000	1.035126000	9.412115000
H	2.733257000	0.772858000	9.500437000
H	1.091692000	0.148589000	9.639836000
H	1.445608000	1.801173000	10.154597000
C	1.891121000	5.268880000	11.919715000
H	2.330899000	4.469833000	12.496906000
H	0.806830000	5.206743000	12.015072000
C	2.341767000	6.644608000	12.381042000
C	3.393849000	6.916194000	13.267594000
C	3.767083000	8.236599000	13.516358000
H	4.585743000	8.435802000	14.193877000
C	3.100088000	9.295079000	12.922290000
C	2.011788000	9.044958000	12.099014000
H	1.453317000	9.864218000	11.669480000
C	1.645870000	7.734425000	11.847330000
H	0.781346000	7.548595000	11.223909000
C	3.552020000	10.703682000	13.217118000
F	2.869756000	11.607272000	12.491795000
F	4.860360000	10.862166000	12.936041000
F	3.383971000	11.011806000	14.515678000
C	4.208086000	5.888917000	14.035283000
F	3.637280000	4.673974000	14.111603000
F	4.390005000	6.301203000	15.306689000
F	5.428644000	5.741015000	13.494972000

[L7+Hb+BnA]+
E [Eh]: −1698.856319645326

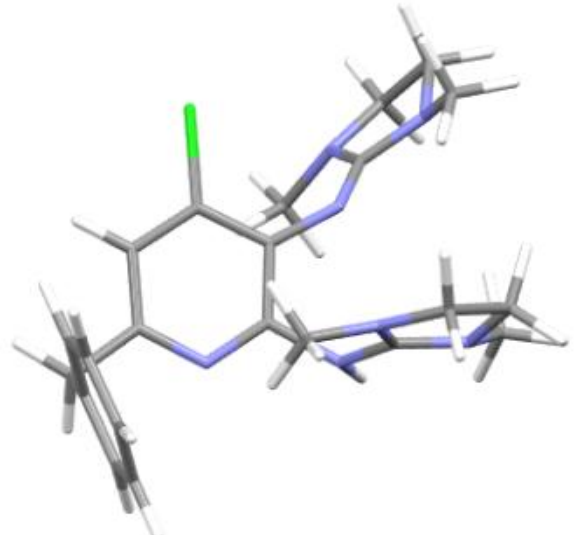

N	6.108831000	5.911411000	6.739649000
N	4.355455000	10.335756000	4.717787000
N	3.843487000	9.598301000	2.675303000
N	4.091574000	5.879810000	5.531262000
N	4.886968000	8.066351000	4.071465000
N	4.761420000	4.704388000	3.597015000
N	2.671108000	5.449177000	3.686915000
C	7.323868000	6.403002000	6.993813000
C	4.428181000	9.260344000	3.874844000
C	7.789635000	7.534446000	6.328667000

C	4.366570000	5.703931000	4.081677000	C	3.862135000	5.373640000	4.309756000
C	5.926639000	7.199556000	4.948552000	C	5.363068000	6.495451000	5.830996000
C	7.724309000	8.720813000	5.475478000	C	6.988944000	8.146972000	5.378318000
C	6.578697000	8.443953000	4.746805000	C	5.705313000	7.642156000	5.084992000
C	2.657409000	10.301707000	4.121435000	C	3.553645000	11.396530000	4.107794000
H	2.180705000	11.067362000	4.729708000	H	3.970809000	12.378010000	4.329163000
H	2.021076000	9.412838000	4.102282000	H	2.525042000	11.361344000	4.487370000
C	3.000630000	10.787413000	2.709974000	C	3.615521000	11.039291000	2.627017000
H	2.275577000	10.467902000	1.963981000	H	2.698563000	11.280021000	2.090812000
H	3.103415000	11.875962000	2.662079000	H	4.453851000	11.541517000	2.129256000
C	4.175320000	9.684317000	6.073819000	C	4.357878000	10.242454000	6.163707000
H	3.976576000	8.635515000	6.299076000	H	3.381288000	9.923928000	6.549271000
H	3.490730000	10.313805000	6.639555000	H	4.584371000	11.224831000	6.576076000
H	5.190693000	9.929816000	6.370284000	H	5.117889000	9.551385000	6.511120000
C	6.504981000	4.560858000	3.459554000	C	6.032975000	4.163156000	4.068642000
H	6.878234000	5.541540000	3.173878000	H	6.858771000	4.832147000	3.826113000
H	6.699382000	3.865445000	2.642342000	H	6.197947000	3.206351000	3.575909000
H	7.033975000	4.227593000	4.351716000	H	6.009131000	4.004872000	5.143013000
C	4.248105000	3.428625000	3.673229000	C	4.177080000	4.261038000	2.326814000
H	4.423962000	2.845890000	4.582769000	H	4.065055000	3.174416000	2.340855000
H	4.472706000	2.803554000	2.810031000	H	4.818335000	4.533341000	1.489431000
C	4.989500000	10.196552000	1.215017000	C	4.188525000	8.901244000	1.454085000
H	5.587968000	11.107191000	1.112568000	H	5.087251000	9.323018000	0.989136000
H	4.253658000	10.175244000	0.413449000	H	3.362352000	8.972643000	0.746219000
H	5.629496000	9.321894000	1.098017000	H	4.380102000	7.857744000	1.689022000
C	1.981113000	6.288385000	4.367439000	C	1.675151000	6.465807000	3.986176000
C	2.834848000	4.018797000	3.631942000	C	2.823880000	4.984289000	2.300641000
H	2.448370000	4.081810000	2.607215000	H	2.839536000	5.847455000	1.629698000
H	2.123629000	3.458021000	4.236876000	H	1.999168000	4.333698000	2.015426000
H	1.191821000	5.833063000	4.966803000	H	0.770367000	6.228276000	3.430806000
H	1.550072000	6.608720000	3.410545000	H	2.026830000	7.461865000	3.702638000
H	2.369195000	7.157853000	4.890798000	H	1.411464000	6.453179000	5.042936000
H	9.113164000	8.000907000	6.951470000	H	8.779866000	7.921100000	6.524508000
Cl	8.550366000	10.228943000	5.233948000	Cl	7.595499000	9.517026000	4.509746000
C	7.957750000	5.555345000	7.533997000	C	8.201182000	5.584679000	7.908250000
C	7.980063000	4.172804000	6.929150000	C	8.554596000	4.287153000	7.211300000
C	9.088435000	3.738801000	6.203391000	C	9.552686000	4.262204000	6.237502000
C	9.097371000	2.486264000	5.599963000	C	9.820660000	3.100091000	5.524935000
C	7.993486000	1.648944000	5.716974000	C	9.088825000	1.944111000	5.777604000
C	6.884766000	2.071744000	6.442525000	C	8.095667000	1.958425000	6.750217000
C	6.879250000	3.325726000	7.041772000	C	7.829736000	3.124001000	7.460771000
H	8.003125000	0.668793000	5.258163000	H	9.302874000	1.034695000	5.231197000
H	6.028620000	1.417841000	6.553140000	H	7.532754000	1.058525000	6.963363000
H	6.012103000	3.654982000	7.599938000	H	7.050959000	3.132934000	8.212806000
H	9.954028000	4.384642000	6.111766000	H	10.126727000	5.159179000	6.035239000
H	9.969180000	2.160580000	5.047261000	H	10.606452000	3.093439000	4.780358000
H	8.943905000	5.819612000	7.917203000	H	9.102972000	6.143885000	8.159679000
H	7.268560000	5.571971000	8.382597000	H	7.664127000	5.377214000	8.834403000
H	6.698571000	9.535567000	2.976597000	H	3.289201000	6.298213000	5.976916000

$[L7+Hc+BnA]^+$
E [Eh]: −1698.870842070935

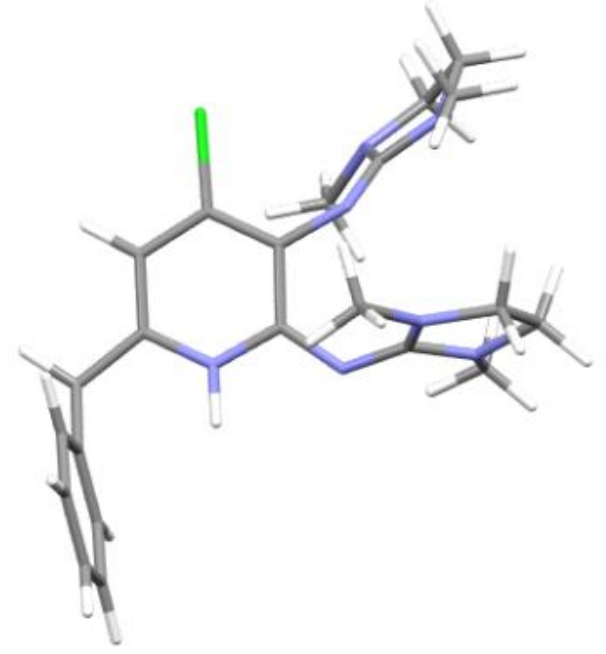

$[L7+Ha+BnB]^+$
E [Eh]: −1698.855666827719

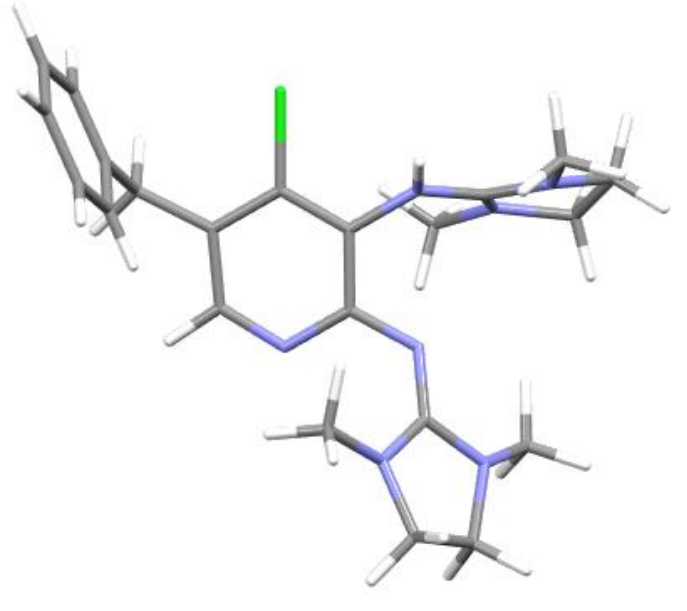

N	6.567938000	5.746534000	6.311675000	N	4.733171000	5.524918000	6.129475000
N	4.175866000	9.884872000	4.700159000	N	4.667368000	9.737226000	4.429983000
N	3.983587000	9.446520000	2.520405000	N	5.190327000	9.474171000	2.298843000
N	4.734354000	5.448513000	4.984846000	N	3.779695000	6.692607000	4.312292000
N	5.266613000	7.950340000	3.744064000	N	6.185371000	8.000132000	3.828582000
N	4.616337000	5.237409000	2.617692000	N	2.851823000	4.498617000	3.968272000
N	2.750048000	5.591929000	3.738482000	N	1.551641000	6.280966000	3.740820000
C	7.643949000	6.401270000	6.833739000	C	5.781374000	5.312775000	6.917348000
C	4.552029000	9.026228000	3.699158000	C	5.364155000	9.014356000	3.549379000

C	7.962288000	7.619078000	6.314734000
C	4.090644000	5.481510000	3.826931000
C	5.761795000	6.205701000	5.314904000
C	7.160643000	8.164464000	5.289553000
C	6.046490000	7.515068000	4.780570000
C	3.162714000	10.813872000	4.195236000
H	3.290996000	11.804480000	4.628500000
H	2.155934000	10.451398000	4.437495000
C	3.417093000	10.779458000	2.692761000
H	2.510112000	10.905419000	2.102816000
H	4.139982000	11.547492000	2.391616000
C	4.117732000	9.503462000	6.098974000
H	3.419872000	8.675750000	6.269381000
H	3.779235000	10.364690000	6.671705000
H	5.097799000	9.220752000	6.471269000
C	6.016483000	5.037192000	2.334146000
H	6.495027000	5.971011000	2.028607000
H	6.121085000	4.300262000	1.537692000
H	6.518336000	4.657160000	3.221616000
C	3.590959000	5.217042000	1.579046000
H	3.502407000	4.212197000	1.158582000
H	3.839790000	5.905346000	0.770517000
C	4.545412000	9.074106000	1.239802000
H	5.361470000	9.744121000	0.945331000
H	3.768649000	9.113371000	0.475717000
H	4.935938000	8.063031000	1.309490000
C	1.910395000	6.094170000	4.800005000
C	2.324384000	5.646605000	2.342830000
H	2.021813000	6.667143000	2.088593000
H	1.479569000	4.981860000	2.163588000
H	0.965317000	5.550808000	4.815132000
H	1.699284000	7.160737000	4.662808000
H	2.419858000	5.947016000	5.748737000
H	8.813585000	8.167529000	6.686678000
Cl	7.592845000	9.719867000	4.668641000
C	8.380178000	5.716357000	7.953203000
C	8.598629000	4.243408000	7.691785000
C	9.531173000	3.829091000	6.739332000
C	9.707331000	2.480144000	6.466069000
C	8.948177000	1.525739000	7.137877000
C	8.017672000	1.928012000	8.086190000
C	7.844729000	3.281971000	8.361482000
H	9.089776000	0.473852000	6.927634000
H	7.432314000	1.191349000	8.620701000
H	7.131463000	3.589118000	9.118065000
H	10.126466000	4.567771000	6.215165000
H	10.442023000	2.170359000	5.734220000
H	9.330128000	6.231570000	8.097669000
H	7.809664000	5.841584000	8.878480000
H	6.362216000	4.813349000	6.650554000

C	7.029004000	5.920590000	6.795418000
C	2.811114000	5.849452000	4.060445000
C	4.842537000	6.390760000	5.110301000
C	7.135937000	6.843179000	5.754153000
C	6.057497000	7.094254000	4.917215000
C	3.793074000	10.673238000	3.715985000
H	3.780752000	11.636714000	4.221262000
H	2.772624000	10.283464000	3.671141000
C	4.443469000	10.733901000	2.330161000
H	3.715643000	10.764878000	1.521737000
H	5.121939000	11.586716000	2.229829000
C	4.559659000	9.538344000	5.864907000
H	3.811807000	8.782056000	6.107222000
H	4.272837000	10.488881000	6.311140000
H	5.520024000	9.243089000	6.277040000
C	4.057765000	3.705044000	3.860302000
H	4.887349000	4.321544000	3.517315000
H	3.892749000	2.913659000	3.128220000
H	4.329282000	3.261341000	4.818186000
C	1.574629000	3.963554000	3.502138000
H	1.275141000	3.091142000	4.082310000
H	1.653651000	3.669457000	2.449909000
C	5.910917000	9.022102000	1.127613000
H	6.906167000	9.471368000	1.053043000
H	5.340688000	9.296611000	0.242239000
H	5.998766000	7.935760000	1.139877000
C	1.061534000	7.580792000	4.134895000
C	0.629662000	5.151304000	3.692142000
H	-0.087697000	5.256621000	2.878733000
H	0.074953000	5.076303000	4.634897000
H	0.574970000	7.549382000	5.117241000
H	0.340216000	7.942541000	3.401048000
H	1.904064000	8.264234000	4.181692000
Cl	8.621855000	7.700539000	5.487041000
C	8.169398000	5.568756000	7.709647000
C	9.263889000	4.761181000	7.037768000
C	10.603275000	5.079129000	7.246550000
C	11.612750000	4.325866000	6.658739000
C	11.292787000	3.242750000	5.849140000
C	9.957955000	2.918391000	5.632904000
C	8.952305000	3.673656000	6.222866000
H	12.077266000	2.654804000	5.390969000
H	9.700400000	2.073134000	5.007319000
H	7.915045000	3.413236000	6.048844000
H	10.858902000	5.925592000	7.872867000
H	12.648864000	4.586325000	6.832704000
H	6.868318000	7.770353000	3.122994000
H	8.605615000	6.477553000	8.128865000
H	7.765133000	5.001818000	8.552522000
H	5.630912000	4.595654000	7.718910000

[L7+Hb+BnB]+
E [Eh]: −1698.852816505130

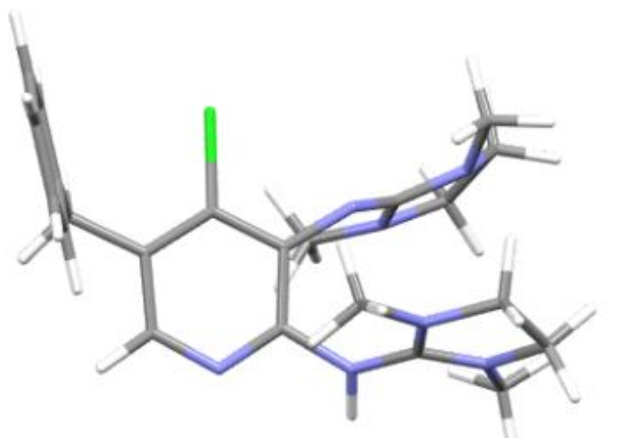

N	4.729416000	5.619699000	7.267845000
N	4.363364000	9.388908000	4.619162000
N	4.374428000	8.704424000	2.491799000
N	3.214365000	6.179756000	5.593695000
N	5.329591000	7.255281000	4.039877000
N	3.354281000	4.964092000	3.555115000
N	1.804817000	6.526139000	3.743801000
C	5.961975000	5.601625000	7.769897000
C	4.749389000	8.374314000	3.781564000
C	7.083513000	6.084710000	7.095954000
C	2.823336000	5.897869000	4.341747000
C	4.557550000	6.148788000	6.073376000

[L7+Hc+BnB]+
E [Eh]: −1698.864680928565

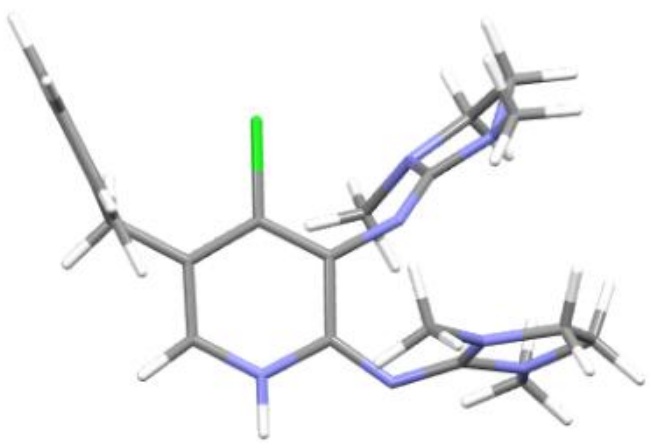

N	4.633381000	5.194931000	7.263219000
N	4.995501000	9.363426000	4.502121000
N	4.727816000	8.545601000	2.443233000
N	3.022714000	5.591958000	5.691825000
N	4.950285000	6.974948000	4.135149000
N	2.909290000	4.889310000	3.420613000
N	1.564345000	6.501528000	4.092083000
C	5.873665000	5.287226000	7.820857000
C	4.917399000	8.211267000	3.763187000
C	6.881282000	5.937050000	7.177443000
C	2.574059000	5.684657000	4.446641000
C	4.281317000	5.722979000	6.061628000

C	6.857478000	6.670254000	5.850076000	C	6.558574000	6.536312000	5.928099000
C	5.569032000	6.744579000	5.298598000	C	5.295122000	6.472422000	5.357861000
C	3.819679000	10.516669000	3.858184000	C	4.659411000	10.512747000	3.660266000
H	4.303193000	11.449759000	4.147272000	H	5.270444000	11.376583000	3.917356000
H	2.746169000	10.621798000	4.040344000	H	3.603040000	10.782363000	3.784167000
C	4.122473000	10.139335000	2.404706000	C	4.936018000	9.977496000	2.259981000
H	3.293157000	10.354462000	1.731387000	H	4.262573000	10.384423000	1.506680000
H	5.010410000	10.662996000	2.029616000	H	5.968751000	10.178816000	1.950271000
C	4.878857000	9.598857000	5.963031000	C	4.673336000	9.438549000	5.913949000
H	4.666365000	8.753640000	6.612427000	H	3.611246000	9.244323000	6.102939000
H	4.385225000	10.473297000	6.381932000	H	4.907946000	10.442138000	6.264289000
H	5.958328000	9.774292000	5.961981000	H	5.268930000	8.737214000	6.489366000
C	4.449362000	4.063899000	3.865646000	C	3.951658000	3.892044000	3.435362000
H	5.408919000	4.507292000	3.593900000	H	4.900069000	4.304398000	3.081564000
H	4.301572000	3.143368000	3.303279000	H	3.659613000	3.058460000	2.796802000
H	4.446807000	3.825494000	4.925805000	H	4.084896000	3.518186000	4.448565000
C	2.786600000	5.059951000	2.206969000	C	2.130405000	5.195754000	2.224465000
H	2.539893000	4.067054000	1.835606000	H	1.499860000	4.344241000	1.958162000
H	3.504121000	5.522310000	1.526600000	H	2.784887000	5.414278000	1.379967000
C	4.991531000	8.047013000	1.357530000	C	5.083382000	7.634963000	1.375737000
H	5.919869000	8.548920000	1.061332000	H	6.131723000	7.752709000	1.078683000
H	4.307589000	8.054264000	0.507859000	H	4.451978000	7.822781000	0.506680000
H	5.233559000	7.024068000	1.631161000	H	4.934279000	6.616135000	1.721492000
C	1.096613000	7.681847000	4.249700000	C	1.090302000	7.606910000	4.889221000
C	1.541738000	5.935345000	2.428777000	C	1.306816000	6.424225000	2.657111000
H	1.446750000	6.722312000	1.682321000	H	1.654997000	7.339827000	2.170168000
H	0.617934000	5.352467000	2.445361000	H	0.241889000	6.308247000	2.455749000
H	0.153237000	7.408154000	4.728401000	H	0.002923000	7.667395000	4.832857000
H	0.882393000	8.353909000	3.419004000	H	1.511412000	8.554991000	4.537335000
H	1.729001000	8.211882000	4.960137000	H	1.383494000	7.443685000	5.922927000
Cl	8.157856000	7.390565000	4.965442000	Cl	7.784557000	7.424168000	5.093569000
C	8.457844000	5.936045000	7.691361000	C	8.266206000	5.981103000	7.762196000
C	9.254836000	4.829310000	7.025100000	C	9.280270000	5.153361000	6.994682000
C	10.491876000	5.091490000	6.443761000	C	10.573506000	5.635206000	6.807020000
C	11.223419000	4.072275000	5.843912000	C	11.523231000	4.875044000	6.136209000
C	10.723000000	2.776859000	5.817490000	C	11.188455000	3.620543000	5.639789000
C	9.487494000	2.505160000	6.396214000	C	9.899577000	3.132467000	5.819968000
C	8.759538000	3.525676000	6.993213000	C	8.952909000	3.895184000	6.493160000
H	11.293052000	1.982390000	5.353867000	H	11.926724000	3.027753000	5.115949000
H	9.095129000	1.496221000	6.388804000	H	9.631808000	2.155415000	5.438428000
H	7.799400000	3.303886000	7.444388000	H	7.950655000	3.506286000	6.626730000
H	10.885629000	6.100249000	6.458283000	H	10.838283000	6.615196000	7.185876000
H	12.185218000	4.291808000	5.398570000	H	12.524011000	5.263584000	5.998742000
H	9.003850000	6.875857000	7.606586000	H	8.612936000	7.014923000	7.805969000
H	8.351293000	5.721758000	8.757043000	H	8.212558000	5.628174000	8.795497000
H	6.074325000	5.172011000	8.758388000	H	6.000369000	4.807742000	8.779243000
H	2.512627000	6.480058000	6.252467000	H	3.899546000	4.704196000	7.755502000

$[L7+BnD]^{+}$
E [Eh]: −1698.845934940093

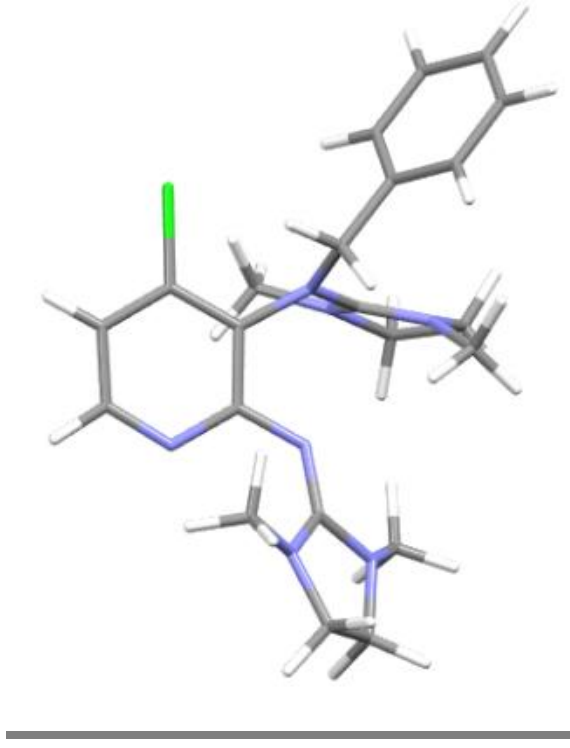

$[L7+BnE]^{+}$
E [Eh]: −1698.848665968896

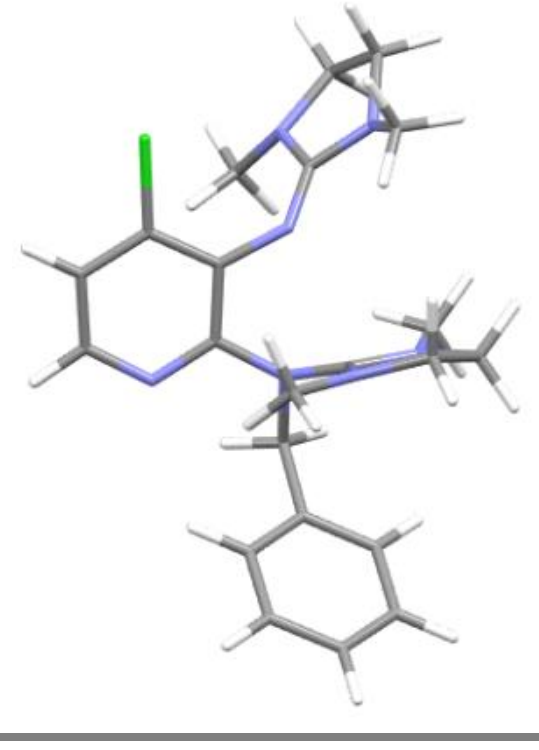

N	7.132065000	6.954494000	4.780036000	N	7.320878000	6.036797000	6.369669000
N	4.852413000	10.848544000	4.360082000	N	4.209193000	9.679483000	4.951024000
N	4.327747000	10.539448000	2.237081000	N	3.815943000	9.212445000	2.803699000
N	5.376190000	7.663998000	3.374262000	N	5.290424000	5.513203000	5.326723000
N	6.574059000	10.097098000	2.925589000	N	5.403598000	7.883108000	3.861429000
N	5.523605000	5.335371000	2.768446000	N	5.518024000	4.998244000	3.002297000
N	3.533426000	6.289055000	2.992173000	N	3.486078000	5.223289000	3.822349000
C	8.272838000	7.238880000	5.399960000	C	8.337239000	6.871240000	6.600396000
C	5.293827000	10.459195000	3.158725000	C	4.562382000	8.866131000	3.905878000
C	8.947791000	8.445228000	5.285912000	C	8.467907000	8.084795000	5.948042000

C	4.886174000	6.483530000	3.094578000	C	4.794148000	5.287308000	4.077704000
C	6.577705000	7.876846000	3.975608000	C	6.391816000	6.394453000	5.504343000
C	8.373626000	9.414397000	4.473569000	C	7.482186000	8.452487000	5.040919000
C	7.186015000	9.153501000	3.804226000	C	6.378580000	7.620738000	4.796733000
C	3.407386000	11.085262000	4.323245000	C	3.054246000	10.496393000	4.577456000
H	3.168567000	12.012130000	4.841169000	H	3.135395000	11.500907000	4.990177000
H	2.870267000	10.266151000	4.808721000	H	2.129251000	10.039769000	4.951389000
C	3.133163000	11.168252000	2.815268000	C	3.114748000	10.467849000	3.055016000
H	2.243046000	10.616511000	2.519969000	H	2.130644000	10.476098000	2.587866000
H	3.038276000	12.198682000	2.466282000	H	3.693758000	11.311888000	2.661408000
C	5.641594000	11.055571000	5.558994000	C	4.342400000	9.314284000	6.347945000
H	5.741293000	10.136690000	6.139803000	H	3.595512000	8.567163000	6.644494000
H	5.145358000	11.808725000	6.168481000	H	4.192191000	10.206793000	6.953493000
H	6.631154000	11.420360000	5.296601000	H	5.332628000	8.927615000	6.561704000
C	6.915751000	5.230716000	2.382467000	C	6.961976000	4.928025000	2.911220000
H	7.301619000	6.206357000	2.093477000	H	7.378573000	5.890422000	2.605137000
H	6.992056000	4.561273000	1.524469000	H	7.225004000	4.168763000	2.176439000
H	7.526344000	4.846767000	3.199152000	H	7.379363000	4.638142000	3.870410000
C	4.571308000	4.301799000	2.365628000	C	4.660907000	4.901381000	1.819198000
H	4.817459000	3.340383000	2.815250000	H	4.921569000	4.015353000	1.243256000
H	4.582676000	4.187806000	1.276285000	H	4.789003000	5.782401000	1.186848000
C	4.280720000	9.804115000	0.985326000	C	4.290225000	8.940711000	1.462631000
H	4.622259000	10.411458000	0.147493000	H	4.903517000	9.765294000	1.081746000
H	3.249126000	9.505651000	0.805300000	H	3.442173000	8.798884000	0.791796000
H	4.880308000	8.902604000	1.077955000	H	4.897939000	8.041075000	1.482407000
C	2.583175000	7.158613000	3.645927000	C	2.428436000	5.713293000	4.676356000
C	3.240328000	4.865019000	2.866320000	C	3.252765000	4.813468000	2.434863000
H	2.416823000	4.687097000	2.175477000	H	2.545501000	5.491668000	1.959554000
H	2.969552000	4.447095000	3.843042000	H	2.845447000	3.800878000	2.401389000
H	1.649026000	7.171544000	3.083221000	H	1.815856000	4.896243000	5.063804000
H	3.002219000	8.159952000	3.679272000	H	1.791313000	6.391583000	4.106657000
H	2.369852000	6.830476000	4.670276000	H	2.865687000	6.261057000	5.506721000
H	9.870370000	8.632942000	5.813551000	H	9.310507000	8.734054000	6.136011000
Cl	9.146745000	10.962982000	4.324385000	Cl	7.600887000	9.971955000	4.217783000
C	7.232639000	10.372843000	1.629193000	C	4.967665000	4.492279000	6.365184000
C	6.960160000	11.756958000	1.096666000	C	5.320486000	3.102066000	5.908302000
C	6.915428000	12.866447000	1.940454000	C	6.650628000	2.679735000	5.897397000
C	6.700820000	14.136389000	1.421233000	C	6.981222000	1.420525000	5.415390000
C	6.537820000	14.314952000	0.051488000	C	5.987694000	0.570353000	4.937673000
C	6.593695000	13.216167000	-0.796555000	C	4.660642000	0.981105000	4.952209000
C	6.799251000	11.944366000	-0.273963000	C	4.330857000	2.242823000	5.436304000
H	6.374878000	15.305499000	-0.352079000	H	6.247072000	-0.412868000	4.567574000
H	6.476133000	13.346766000	-1.864282000	H	3.882660000	0.317213000	4.598020000
H	6.843543000	11.091486000	-0.941500000	H	3.292972000	2.554936000	5.460691000
H	7.061895000	12.739457000	3.004525000	H	7.418118000	3.346000000	6.270705000
H	6.671621000	14.990392000	2.085534000	H	8.013733000	1.095901000	5.420824000
H	8.678870000	6.456703000	6.032587000	H	9.073798000	6.549567000	7.325765000
H	8.298134000	10.258375000	1.825161000	H	5.542538000	4.782318000	7.239102000
H	6.966143000	9.618603000	0.887292000	H	3.907570000	4.557346000	6.608971000

[L2a+2Bn]$^{\cdot+}$

[L2a+Hc+BnA+BnF]$^{\cdot+}$
E [Eh]: −1510.167564035616

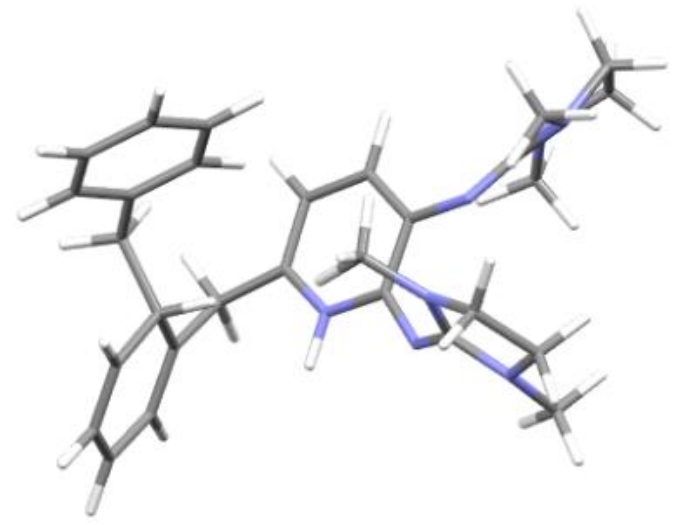

N	5.428422000	7.203517000	7.397238000
N	4.435871000	9.395618000	2.812059000
N	4.364938000	7.530607000	1.582516000
N	3.866014000	6.009445000	6.241576000
N	4.803920000	7.229182000	3.839257000
N	4.380446000	4.318749000	4.634647000
N	2.263058000	4.809775000	5.038769000
C	6.470708000	8.059679000	7.594173000
C	4.567981000	8.022218000	2.843650000
C	6.978375000	8.688545000	6.493504000
C	7.177308000	6.826149000	9.666559000
C	3.547360000	5.131581000	5.314632000
C	4.868352000	6.872954000	6.203094000
C	6.411979000	8.452688000	5.229972000
C	5.356889000	7.573147000	5.041599000
C	7.866150000	5.729218000	8.894430000
C	6.712703000	6.612045000	10.932227000
H	6.217877000	7.424186000	11.454375000
C	6.997818000	8.167899000	8.997618000
C	3.955368000	9.802041000	1.486328000
H	4.417967000	10.736452000	1.172250000
H	2.867754000	9.939794000	1.508269000
C	7.474043000	4.297138000	10.915025000
H	7.571381000	3.343305000	11.418758000
C	4.341313000	8.615097000	0.611260000
H	3.623491000	8.422190000	-0.185422000
H	5.332424000	8.749843000	0.160478000
C	7.942629000	4.439127000	9.648181000
H	8.411374000	3.605818000	9.140649000
C	3.899687000	10.146132000	3.936398000
H	2.830789000	9.946129000	4.076152000
H	4.030868000	11.208648000	3.735838000
H	4.424704000	9.907543000	4.854669000
C	6.855719000	5.373293000	11.589249000
H	6.486955000	5.249902000	12.597506000
C	5.811784000	4.240089000	4.820967000
H	6.138037000	3.213561000	4.658494000
H	6.081072000	4.520902000	5.837774000
H	6.339313000	4.895886000	4.126419000
C	3.634988000	3.319611000	3.873367000
H	3.725740000	2.339323000	4.351205000
H	4.008444000	3.238678000	2.852395000
C	4.811107000	6.212796000	1.191637000
H	5.848587000	6.224222000	0.836849000
H	4.174411000	5.835624000	0.390946000
H	4.740764000	5.554710000	2.053117000
C	1.139438000	5.659505000	5.362003000
C	2.198967000	3.859726000	3.933984000
H	1.919962000	4.377471000	3.009404000
H	1.463120000	3.079358000	4.125626000
H	6.811716000	8.967761000	4.365960000
H	7.818875000	9.358190000	6.602039000
H	0.884557000	6.316898000	4.523356000
H	1.396617000	6.266682000	6.225751000
H	0.272007000	5.044593000	5.602102000
H	5.070164000	6.696905000	8.199801000
H	8.481416000	6.281836000	5.774639000
C	9.170029000	5.460965000	5.940280000

[L2a+Hc+BnA+BnG]$^{\cdot+}$
E [Eh]: −1510.165860707728

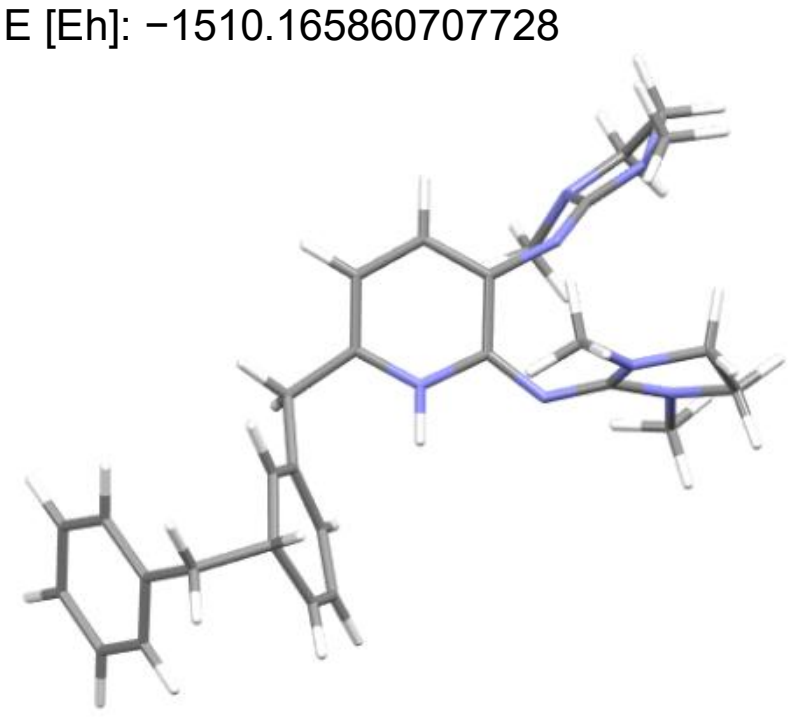

N	6.290629000	5.949437000	6.342427000
N	4.092086000	9.553660000	3.847079000
N	3.368077000	8.566833000	1.973385000
N	4.129520000	5.663992000	5.653475000
N	4.734420000	7.275440000	3.336210000
N	3.343384000	4.583851000	3.669780000
N	1.924850000	5.778003000	4.866989000
C	7.550881000	6.464095000	6.339511000
C	4.132583000	8.391113000	3.102572000
C	7.898553000	7.274728000	5.294762000
C	8.229702000	4.728186000	8.046512000
C	3.212762000	5.390495000	4.743677000
C	5.325960000	6.179666000	5.409877000
C	6.954081000	7.564775000	4.296018000
C	5.669064000	7.058502000	4.327427000
C	8.934841000	3.672413000	7.552428000
C	7.248978000	4.522063000	9.048032000
H	6.738491000	5.370393000	9.487294000
C	8.445628000	6.121496000	7.501508000
C	3.106971000	10.467579000	3.263483000
H	3.440129000	11.501796000	3.338451000
H	2.146090000	10.371639000	3.784623000
C	7.649074000	2.142989000	9.030156000
H	7.414686000	1.145334000	9.381013000
C	3.006709000	9.970831000	1.827318000
H	2.008420000	10.083950000	1.405802000
H	3.720445000	10.486851000	1.172692000
C	8.813076000	2.291230000	8.105791000
C	4.198271000	9.568001000	5.295292000
H	5.082622000	9.036059000	5.628260000
H	3.313881000	9.126745000	5.770892000
H	4.285409000	10.603172000	5.622881000
C	6.951561000	3.212399000	9.491067000
H	6.158783000	3.069101000	10.214904000
C	4.595072000	4.102991000	3.132770000
H	5.046121000	4.828922000	2.452480000
H	4.412170000	3.169198000	2.602196000
H	5.287174000	3.901770000	3.948191000
C	2.117149000	4.592882000	2.877040000
H	1.894893000	3.598812000	2.492210000
H	2.216846000	5.284251000	2.034625000
C	3.580166000	7.767337000	0.786177000
H	4.349392000	8.204643000	0.138487000
H	2.650454000	7.695602000	0.220802000
H	3.901971000	6.773460000	1.082857000
C	1.371700000	6.414441000	6.039317000
C	1.083217000	5.086839000	3.895479000
H	0.350866000	5.764923000	3.459706000
H	0.550412000	4.257275000	4.373507000
H	7.225388000	8.216221000	3.475100000
H	8.893861000	7.691470000	5.249397000
H	0.689603000	7.212354000	5.742024000
H	2.185236000	6.830942000	6.626973000
H	0.822525000	5.696088000	6.656300000
H	6.036963000	5.316093000	7.095924000
H	11.765414000	4.049828000	8.715431000
C	11.366539000	3.885419000	9.709554000
C	10.156775000	1.864832000	8.818469000

C	9.277858000	6.146260000	8.352671000
H	9.098520000	4.804223000	3.899596000
C	9.515466000	4.628792000	4.883830000
C	9.713175000	5.267068000	7.213148000
C	10.410459000	3.582300000	5.083055000
C	10.614245000	4.220256000	7.398197000
C	10.961070000	3.382880000	6.343073000
H	10.686234000	2.935881000	4.260102000
H	11.051690000	4.061307000	8.376445000
H	11.665921000	2.577661000	6.505290000
H	6.321461000	8.780467000	9.601451000
H	7.942207000	8.716640000	8.947357000
H	9.230127000	7.179361000	8.007806000
H	9.993917000	6.110706000	9.174294000
H	7.285535000	5.540813000	7.976437000

H	12.333647000	5.614000000	10.532778000
C	11.687753000	4.768309000	10.732700000
C	10.547121000	2.780758000	9.944554000
C	11.191834000	4.557989000	12.014487000
C	10.065144000	2.576192000	11.237079000
C	10.383488000	3.455379000	12.264865000
H	11.445339000	5.240441000	12.815370000
H	9.437082000	1.718183000	11.442686000
H	10.005254000	3.275882000	13.263141000
H	8.273206000	6.850281000	8.300711000
H	9.478764000	6.252345000	7.181431000
H	10.030026000	0.842133000	9.178705000
H	10.940476000	1.847181000	8.058612000
H	9.681715000	3.836381000	6.783435000
H	8.709627000	1.574989000	7.277794000

[L2a+Hc+BnA+BnH]$^{\cdot+}$
E [Eh]: −1510.169399554859

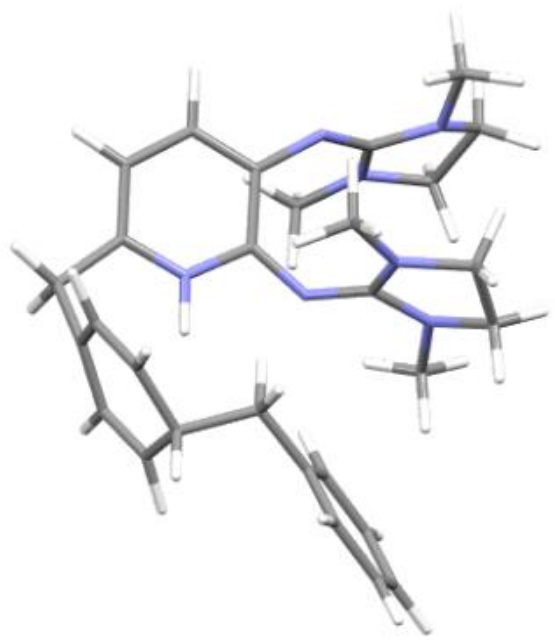

N	7.223071000	7.339471000	6.909012000
N	3.235723000	8.243992000	4.742565000
N	3.182838000	6.773924000	3.078952000
N	5.249996000	6.258532000	6.553318000
N	5.299645000	7.388498000	3.816354000
N	5.142741000	4.409530000	5.025029000
N	3.364766000	4.927936000	6.231990000
C	8.310693000	8.112929000	6.650511000
C	4.014686000	7.482815000	3.919778000
C	8.370345000	8.701598000	5.415562000
C	9.544210000	6.768474000	8.297117000
C	4.642231000	5.278553000	5.931774000
C	6.210100000	7.049291000	6.058322000
C	7.333156000	8.490343000	4.496173000
C	6.232712000	7.689136000	4.775362000
C	10.278254000	5.787776000	7.576767000
C	8.787099000	6.348574000	9.422500000
H	8.354567000	7.104281000	10.071150000
C	9.369828000	8.151525000	7.732029000
C	1.824216000	7.933370000	4.554697000
H	1.217207000	8.836529000	4.608331000
H	1.474177000	7.231025000	5.319204000
C	9.086693000	3.959851000	8.780411000
C	1.825528000	7.297282000	3.165457000
H	1.089067000	6.501327000	3.061865000
H	1.643041000	8.044593000	2.382594000
C	10.135114000	4.465233000	7.838564000
C	3.680169000	9.097108000	5.819861000
H	3.735045000	8.569121000	6.775686000
H	2.980858000	9.927729000	5.917996000
H	4.659126000	9.508349000	5.589887000
C	8.598526000	5.030946000	9.703764000
H	8.027419000	4.732618000	10.572478000
C	6.452262000	4.449766000	4.414837000
H	6.461020000	5.046272000	3.500750000
H	6.758476000	3.429215000	4.186742000
H	7.174819000	4.867252000	5.111872000
C	4.104108000	3.495250000	4.557155000
H	4.495115000	2.482810000	4.466710000
H	3.723172000	3.817227000	3.583708000
C	3.662520000	6.236115000	1.824703000
H	3.646179000	6.988815000	1.027606000
H	3.034041000	5.397527000	1.523079000
H	4.685906000	5.895869000	1.953554000

[L2a+Hc+BnA+BnI]$^{\cdot+}$
E [Eh]: −1510.166881407980

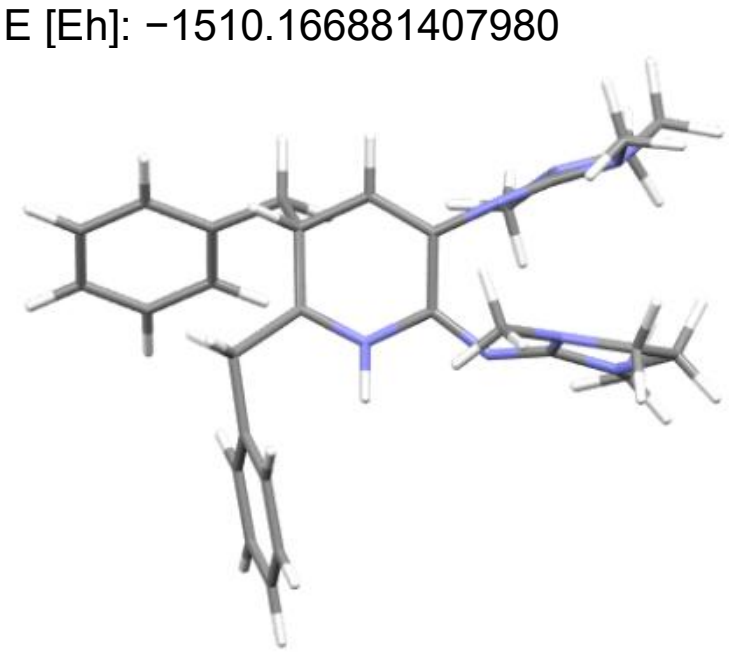

N	6.753170000	6.820148000	6.380418000
N	3.703268000	9.634039000	3.834921000
N	3.337142000	8.352680000	2.045825000
N	4.628117000	6.398955000	5.640817000
N	5.274487000	7.883195000	3.228836000
N	4.183191000	5.134878000	3.658743000
N	2.486237000	5.886560000	4.858879000
C	7.966749000	7.388464000	6.429706000
C	4.207418000	8.584905000	3.098510000
C	8.253723000	8.523532000	5.506803000
C	8.463651000	5.821622000	8.344718000
C	3.835342000	5.870238000	4.733777000
C	5.797489000	6.997684000	5.429213000
C	7.338217000	8.498798000	4.318998000
C	6.140289000	7.870173000	4.307863000
C	8.341192000	4.470898000	8.015624000
C	8.031460000	6.251739000	9.599705000
H	8.132484000	7.296232000	9.867101000
C	9.003832000	6.812717000	7.346170000
C	2.386533000	10.016031000	3.336373000
H	2.251704000	11.096411000	3.380072000
H	1.590871000	9.545250000	3.928207000
C	7.373693000	4.004954000	10.170892000
C	2.417112000	9.474255000	1.912152000
H	1.440235000	9.146610000	1.558118000
H	2.808138000	10.222810000	1.210926000
C	7.800346000	3.566246000	8.923028000
C	4.041363000	9.943059000	5.207317000
H	3.511149000	9.302972000	5.920686000
H	3.772194000	10.980433000	5.406002000
H	5.108350000	9.836611000	5.364210000
C	7.492193000	5.348986000	10.508048000
H	7.171475000	5.693204000	11.482697000
C	5.528005000	4.698201000	3.351263000
H	6.061431000	5.448366000	2.766236000
H	5.471290000	3.771693000	2.781990000
H	6.082068000	4.500320000	4.269487000
C	3.007376000	4.558729000	3.011145000
H	2.951374000	3.485579000	3.218785000
H	3.042865000	4.700826000	1.931516000
C	3.790567000	7.669556000	0.853505000
H	4.343238000	8.338992000	0.183282000
H	2.928917000	7.272778000	0.315849000
H	4.448017000	6.854762000	1.142303000

C	2.645146000	5.425027000	7.381420000
C	3.043110000	3.627424000	5.654039000
H	2.027485000	3.615187000	5.260819000
H	3.136863000	2.841391000	6.411620000
H	7.377817000	8.952523000	3.518616000
H	9.219201000	9.316636000	5.154655000
H	2.688736000	4.710988000	8.209096000
H	1.598676000	5.592813000	7.122833000
H	3.092518000	6.362125000	7.699911000
H	7.199659000	6.838148000	7.792161000
H	5.570192000	4.580703000	8.656375000
C	5.644051000	3.569042000	9.036548000
C	7.894870000	3.382163000	7.920631000
H	3.762239000	3.657572000	10.065751000
C	4.623416000	3.047315000	9.823216000
C	6.771288000	2.804201000	8.732613000
C	4.711256000	1.749210000	10.312829000
C	6.848226000	1.502673000	9.228946000
C	5.827339000	0.976598000	10.010928000
H	3.918234000	1.342896000	10.926856000
H	7.714770000	0.893372000	8.998704000
H	5.903114000	-0.035890000	10.385840000
H	9.073155000	8.854576000	8.516518000
H	10.291608000	8.529081000	7.287758000
H	10.968267000	6.114054000	6.806152000
H	7.522351000	4.196379000	7.297316000
H	8.311537000	2.623953000	7.253754000
H	10.721091000	3.733446000	7.295759000
H	9.471393000	3.115774000	9.363551000

C	1.766771000	6.762600000	5.751643000
C	1.855239000	5.332002000	3.666293000
H	1.498297000	6.143278000	3.023176000
H	1.011852000	4.693027000	3.926111000
H	7.608252000	9.107278000	3.464800000
H	0.897523000	6.242911000	6.155581000
H	1.423427000	7.665681000	5.235932000
H	2.423090000	7.045145000	6.570651000
H	10.818767000	10.300047000	6.290789000
C	10.382980000	10.251050000	7.282220000
C	8.120796000	9.923490000	6.222570000
H	12.273710000	10.463144000	8.270333000
C	11.205654000	10.343139000	8.397428000
C	9.003252000	10.097113000	7.424762000
C	10.658333000	10.289538000	9.675597000
C	8.464706000	10.058168000	8.710243000
C	9.283924000	10.153996000	9.829527000
H	11.298673000	10.364648000	10.544461000
H	7.393482000	9.950578000	8.835598000
H	8.849122000	10.128380000	10.820597000
H	9.515964000	7.629911000	7.855451000
H	9.768530000	6.334978000	6.718290000
H	8.685255000	4.123465000	7.047748000
H	7.722816000	2.519426000	8.659613000
H	6.959725000	3.301385000	10.881082000
H	7.076310000	10.055257000	6.508086000
H	8.346148000	10.678529000	5.465273000
H	6.545192000	6.111450000	7.080217000
H	9.298541000	8.446653000	5.185986000

[L2a+Hc+BnA+BnJ]$^{\cdot+}$
E [Eh]: −1510.187536348059

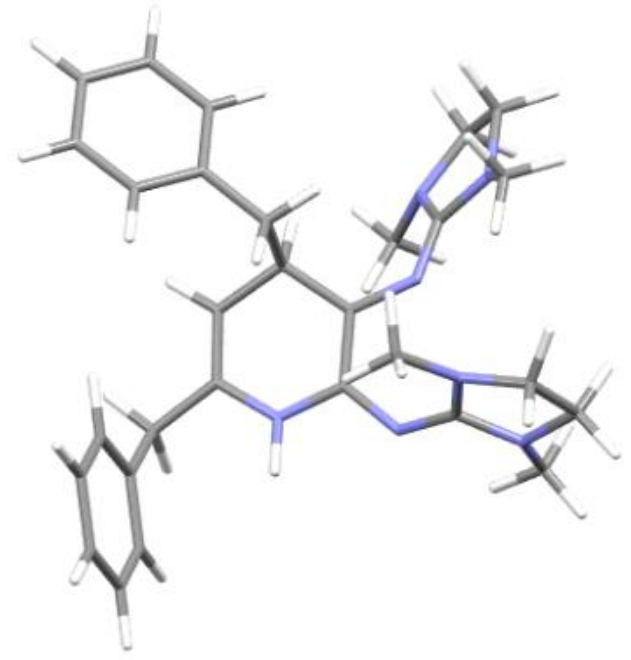

N	5.640700000	6.695014000	7.260302000
N	3.646249000	10.547107000	4.410163000
N	3.548794000	9.447526000	2.474049000
N	3.973320000	5.872882000	5.930461000
N	4.192609000	8.203516000	4.336804000
N	4.230748000	5.205007000	3.644883000
N	2.232078000	5.094277000	4.588946000
C	6.504432000	7.677243000	7.749457000
C	3.842433000	9.339517000	3.798726000
C	6.750520000	8.766078000	7.020881000
C	7.857460000	6.055434000	9.080303000
C	3.521901000	5.463054000	4.769573000
C	4.876134000	6.828029000	6.143173000
C	6.179787000	8.950793000	5.648904000
C	5.012216000	8.028780000	5.386248000
C	9.017396000	5.915576000	8.316593000
C	7.386597000	4.957613000	9.795913000
H	6.495701000	5.057906000	10.405568000
C	7.110911000	7.372243000	9.089692000
C	3.038868000	11.485311000	3.461354000
H	3.457481000	12.484264000	3.573940000
H	1.958022000	11.538925000	3.633769000
C	9.214242000	3.611340000	8.992081000
C	3.360559000	10.847724000	2.110214000
H	2.558447000	10.964864000	1.382605000
H	4.282935000	11.254004000	1.678882000
C	9.693359000	4.704648000	8.274814000
C	3.293677000	10.683062000	5.812911000
H	2.205647000	10.714366000	5.933878000

[L2a+Hc+BnC+BnK]$^{\cdot+}$
E [Eh]: −1510.190243096631

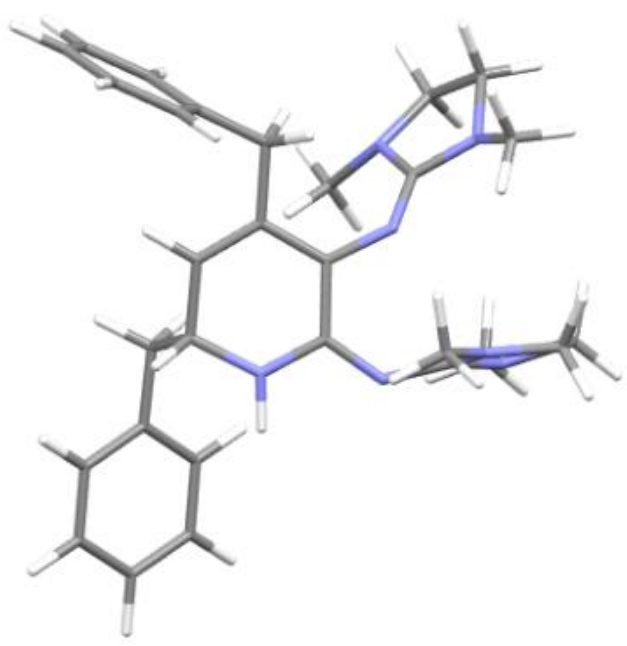

N	6.892859000	5.986394000	6.264864000
N	3.784254000	9.829719000	4.894509000
N	3.232359000	9.119196000	2.849831000
N	4.905660000	5.408978000	5.316161000
N	4.835923000	7.864473000	3.967848000
N	4.356570000	5.002035000	3.040037000
N	2.713412000	5.293392000	4.483797000
C	7.864053000	6.936588000	6.783256000
C	4.036811000	8.877639000	3.935149000
C	7.953028000	8.135463000	5.907171000
C	7.460512000	6.128946000	9.156402000
C	4.053600000	5.301190000	4.313230000
C	5.872788000	6.296022000	5.445135000
C	6.999180000	8.463528000	5.002405000
C	5.866044000	7.600471000	4.816984000
C	8.621534000	5.589756000	9.712290000
C	6.243674000	5.492557000	9.404261000
H	5.331858000	5.904606000	8.987330000
C	7.529529000	7.329506000	8.253610000
C	2.614426000	10.618359000	4.500402000
H	2.729562000	11.660840000	4.793171000
H	1.709818000	10.219725000	4.976011000
C	7.349740000	3.817509000	10.734531000
C	2.583016000	10.417876000	2.989091000
H	1.572976000	10.403348000	2.581729000
H	3.158180000	11.193529000	2.469009000
C	8.568205000	4.444396000	10.496217000
C	3.994363000	9.604903000	6.312573000
H	3.225848000	8.947552000	6.736441000

H	3.716062000	11.605842000	6.210400000
H	3.684235000	9.847291000	6.384817000
C	8.060369000	3.739827000	9.753023000
H	7.686694000	2.897603000	10.320844000
C	5.672611000	5.182157000	3.555851000
H	6.046875000	6.011946000	2.952629000
H	5.996735000	4.243290000	3.102141000
H	6.109189000	5.250018000	4.549346000
C	3.401797000	4.523501000	2.653919000
H	3.658024000	3.459440000	2.618982000
H	3.546499000	4.942254000	1.657734000
C	4.014862000	8.486814000	1.498212000
H	5.001899000	8.757103000	1.106052000
H	3.310848000	8.440120000	0.667460000
H	4.077629000	7.513064000	1.977151000
C	1.156943000	5.520393000	5.456689000
C	1.985007000	4.754848000	3.193319000
H	1.488448000	5.589896000	2.685974000
H	1.350869000	3.873246000	3.105138000
H	0.408988000	4.730747000	5.529109000
H	0.676548000	6.428209000	5.075387000
H	1.564666000	5.719522000	6.444122000
H	9.771900000	8.239030000	5.496790000
C	9.638889000	9.278603000	5.221597000
C	7.272352000	8.736286000	4.545671000
H	11.625322000	9.838289000	5.807482000
C	10.683152000	10.179297000	5.398082000
C	8.419847000	9.694275000	4.689190000
C	10.518490000	11.513761000	5.047344000
C	8.267661000	11.036286000	4.337229000
C	9.306301000	11.940786000	4.514753000
H	11.330141000	12.216387000	5.182680000
H	7.326859000	11.375424000	3.916818000
H	9.174002000	12.977651000	4.233396000
H	6.324788000	7.339468000	9.849503000
H	7.780251000	8.191031000	9.354173000
H	9.390787000	6.765682000	7.757430000
H	10.598842000	4.611897000	7.688822000
H	9.744150000	2.668326000	8.962919000
H	7.628573000	7.706975000	4.609311000
H	6.792308000	8.859604000	3.571526000
H	7.428053000	9.517532000	7.398115000
H	5.850864000	9.986117000	5.534113000
H	5.540589000	5.832231000	7.774416000

[L2a+Hc+BnC+BnL]·+
E [Eh]: −1510.164342868488

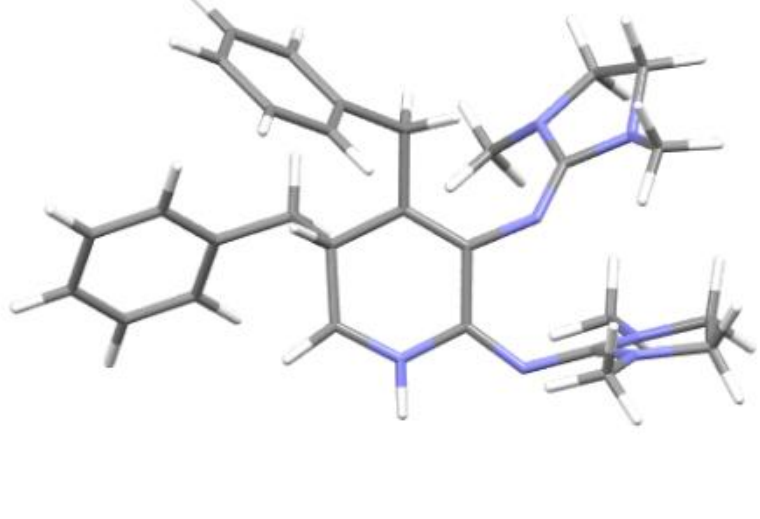

N	6.921024000	5.954932000	6.929630000
N	4.077760000	9.864731000	4.990482000
N	3.824696000	9.001719000	2.943170000
N	5.247678000	5.336536000	5.528957000
N	5.291131000	7.862465000	4.328025000
N	4.562195000	4.915861000	3.314174000
N	3.042663000	5.672356000	4.717227000
C	7.881872000	6.764597000	7.446623000
C	4.480147000	8.848133000	4.150804000
C	7.939386000	8.192054000	7.060884000
C	4.348191000	5.383054000	4.551793000
C	6.049662000	6.285321000	5.948623000
C	7.092162000	8.501462000	5.852676000
C	6.131350000	7.649251000	5.405821000
C	3.015416000	10.644806000	4.354961000
H	3.122392000	11.705636000	4.578463000
H	2.033496000	10.312492000	4.715009000

H	3.949336000	10.564565000	6.825872000
H	4.971591000	9.169386000	6.492719000
C	6.186412000	4.345135000	10.187676000
H	5.233264000	3.868174000	10.376488000
C	5.688876000	4.844175000	2.510397000
H	6.002946000	5.733853000	1.958454000
H	5.717801000	3.981945000	1.842771000
H	6.386279000	4.674218000	3.327483000
C	3.155981000	4.759746000	2.246899000
H	3.080064000	3.698317000	1.994462000
H	3.174507000	5.331184000	1.318378000
C	3.571506000	8.622509000	1.533673000
H	4.235064000	9.312920000	1.000041000
H	2.662552000	8.490270000	0.946260000
H	4.077051000	7.666897000	1.641395000
C	2.057815000	5.849877000	5.643281000
C	2.033575000	5.215023000	3.194376000
H	1.645815000	6.201913000	2.919871000
H	1.201059000	4.513138000	3.228935000
H	1.168881000	5.266370000	5.883346000
H	1.758435000	6.889466000	5.465992000
H	2.745911000	5.812834000	6.483908000
H	9.419785000	9.389175000	2.806565000
C	9.481262000	10.184643000	3.540308000
C	7.082951000	9.720855000	4.158277000
H	11.531858000	10.628879000	3.096192000
C	10.672729000	10.880350000	3.704595000
C	8.361226000	10.498648000	4.308777000
C	10.760055000	11.901304000	4.644654000
C	8.459094000	11.526009000	5.245653000
C	9.648992000	12.224260000	5.414957000
H	11.686458000	12.445925000	4.771316000
H	7.594981000	11.782473000	5.848079000
H	9.707131000	13.022477000	6.143651000
H	6.577646000	7.864833000	8.252075000
H	8.298419000	8.025978000	8.593214000
H	9.574434000	6.076399000	9.538268000
H	9.476967000	4.044197000	10.926637000
H	7.307333000	2.927857000	11.348951000
H	6.939353000	9.430775000	3.113072000
H	6.235707000	10.364547000	4.406167000
H	6.803511000	5.108377000	6.756518000
H	8.834168000	6.429289000	6.802729000
H	8.806718000	8.781270000	6.055336000

[L2a+Hc+BnC+BnM]·+
E [Eh]: −1510.168723522403

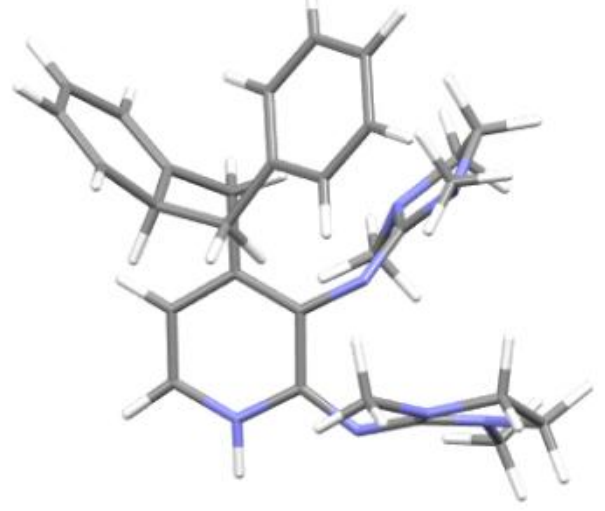

N	7.052462000	7.075747000	6.696331000
N	4.371976000	10.420862000	4.184217000
N	4.723131000	9.854263000	2.049506000
N	5.482892000	6.328824000	5.218590000
N	5.933487000	8.661515000	3.636041000
N	5.679179000	5.743385000	2.908899000
N	3.666730000	6.038129000	3.764333000
C	7.928634000	7.971980000	7.228539000
C	5.078949000	9.585949000	3.344306000
C	8.166131000	9.134346000	6.570275000
C	4.990534000	6.104389000	4.011205000
C	6.363035000	7.262267000	5.537246000
C	7.490841000	9.417021000	5.359622000
C	6.576996000	8.504868000	4.841965000
C	3.346878000	11.124618000	3.407950000
H	3.206519000	12.140724000	3.773372000
H	2.390153000	10.591837000	3.476728000

C	3.206595000	10.320140000	2.879299000	C	3.910757000	11.063809000	1.994044000
H	2.270150000	10.296277000	2.323096000	H	3.138518000	10.990951000	1.229070000
H	3.879103000	11.039318000	2.394774000	H	4.538869000	11.935148000	1.772420000
C	4.035378000	9.771025000	6.436466000	C	4.013822000	10.042333000	5.539536000
H	3.110990000	9.292731000	6.780559000	H	3.281000000	9.226501000	5.551558000
H	4.073618000	10.775179000	6.859071000	H	3.576460000	10.907677000	6.035189000
H	4.880627000	9.206381000	6.810670000	H	4.889320000	9.738379000	6.103740000
C	5.819981000	4.422651000	2.807599000	C	7.108728000	5.865531000	2.744308000
H	6.544782000	4.407793000	3.616960000	H	7.387110000	6.861909000	2.394944000
H	6.192933000	5.064789000	2.006327000	H	7.445457000	5.118319000	2.026445000
H	5.698839000	3.408686000	2.420168000	H	7.600244000	5.672455000	3.695591000
C	3.320345000	4.835318000	2.551412000	C	4.780585000	5.647600000	1.761217000
H	3.025980000	3.789699000	2.422797000	H	5.046554000	4.803507000	1.126932000
H	3.437850000	5.282656000	1.564603000	H	4.828892000	6.565855000	1.167761000
C	4.364384000	8.425071000	1.729944000	C	5.599118000	9.542773000	0.936909000
H	5.117380000	9.076949000	1.270912000	H	6.294419000	10.361586000	0.729466000
H	3.558839000	8.267777000	1.011857000	H	5.003105000	9.354449000	0.043550000
H	4.831217000	7.474971000	1.972452000	H	6.178244000	8.657856000	1.178289000
C	2.494014000	6.380443000	5.846068000	C	2.637368000	6.101415000	4.775403000
C	2.335960000	5.613295000	3.439473000	C	3.414884000	5.481862000	2.438445000
H	2.162118000	6.627501000	3.068005000	H	2.621411000	6.027413000	1.929762000
H	1.376742000	5.108437000	3.547568000	H	3.120417000	4.429738000	2.517724000
H	1.537812000	5.940471000	6.131021000	H	2.215982000	5.110162000	4.967927000
H	2.335607000	7.435834000	5.605372000	H	1.834064000	6.764862000	4.450661000
H	3.180974000	6.303478000	6.684915000	H	3.074395000	6.482367000	5.694359000
H	9.511922000	8.732401000	4.050468000	H	6.865439000	6.206973000	7.178056000
C	9.745827000	9.641832000	4.592486000	C	10.161488000	10.042747000	3.910542000
C	7.301211000	9.835688000	5.177046000	C	7.773503000	10.715525000	4.645832000
H	11.842912000	9.529051000	4.162505000	C	11.511008000	10.610203000	3.586912000
C	11.058898000	10.087312000	4.657680000	C	9.230086000	11.061070000	4.528675000
C	8.720803000	10.346546000	5.225915000	C	11.910538000	11.837298000	4.008887000
C	11.367565000	11.251852000	5.354668000	C	9.704384000	12.269689000	4.949825000
C	9.040155000	11.515278000	5.911914000	C	11.035849000	12.678201000	4.732431000
C	10.353829000	11.968103000	5.977535000	H	12.909878000	12.184913000	3.778055000
H	12.390734000	11.600152000	5.405453000	H	9.021019000	12.959542000	5.433742000
H	8.254812000	12.079277000	6.401616000	H	11.367639000	13.647596000	5.076097000
H	10.582945000	12.878809000	6.515603000	H	8.635915000	13.001161000	0.094309000
H	6.980162000	9.736877000	4.136945000	H	9.251137000	11.950954000	2.222942000
H	6.636397000	10.587004000	5.614970000	C	8.747288000	11.925402000	0.144959000
H	11.707684000	9.853356000	10.233852000	C	9.093677000	11.328192000	1.354324000
H	10.070301000	10.115828000	8.411083000	C	8.560177000	11.153571000	-0.994118000
C	10.724024000	9.411461000	10.327656000	H	8.299710000	11.621011000	-1.934754000
C	9.800825000	9.559011000	9.301130000	C	9.255505000	9.946923000	1.446065000
C	10.386151000	8.702448000	11.476350000	C	9.570772000	9.227246000	2.736214000
H	11.105080000	8.590517000	12.277340000	C	8.733245000	9.774920000	-0.919069000
C	8.525216000	9.000347000	9.406853000	C	9.078466000	9.184252000	0.288229000
C	7.551909000	9.122519000	8.268504000	H	8.610251000	9.163137000	-1.803824000
C	9.118836000	8.145372000	11.592653000	H	9.223185000	8.110330000	0.334627000
C	8.196428000	8.294010000	10.562277000	H	8.394037000	7.691504000	8.160563000
H	8.845598000	7.599079000	12.486314000	H	8.873001000	9.847491000	6.969234000
H	7.206625000	7.863089000	10.660511000	H	7.250166000	11.524774000	5.163622000
H	7.530387000	10.150410000	7.904906000	H	8.660486000	8.732126000	3.083211000
H	6.543169000	8.865417000	8.598367000	H	10.276767000	8.421863000	2.517472000
H	8.978749000	8.448287000	6.833549000	H	7.336689000	10.661708000	3.646686000
H	8.498422000	6.351233000	8.227055000	H	12.186307000	9.978122000	3.022926000
H	6.867532000	4.995156000	7.243680000	H	10.326329000	9.268402000	4.686853000

[L2a+Hc+BnC+BnN]·+
E [Eh]: −1510.169067082709

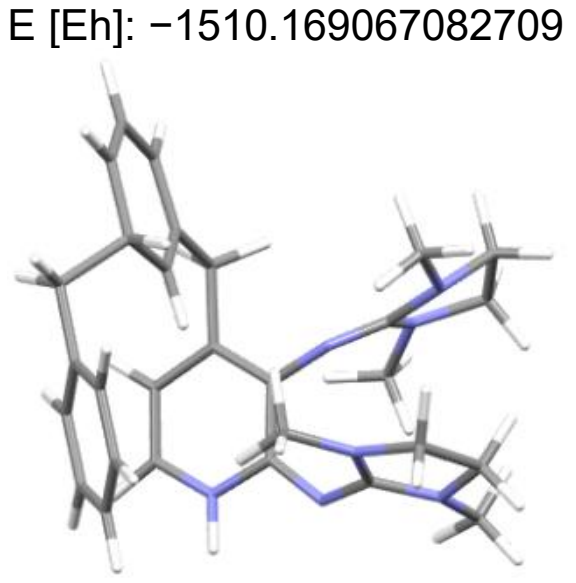

[L2a+Hc+BnC+BnO]·+
E [Eh]: −1510.164594173709

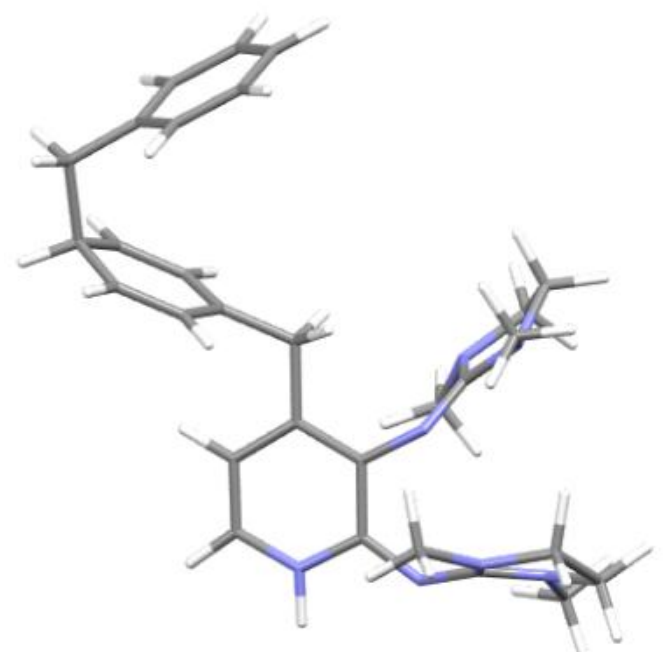

N	8.699829000	7.459248000	5.061153000	N	7.192026000	6.967279000	6.521309000
N	4.176743000	9.319178000	5.329525000	N	4.847655000	10.519113000	3.980829000

N	3.785927000	9.899054000	3.206125000	N	4.754977000	9.683484000	1.908631000
N	6.981893000	6.804509000	3.705597000	N	5.322808000	6.427278000	5.328707000
N	6.002219000	9.494944000	3.747276000	N	6.007150000	8.467513000	3.441869000
N	6.472965000	7.600914000	1.510384000	N	5.170943000	5.559016000	3.104820000
N	5.039042000	6.262123000	2.518475000	N	3.332542000	6.256674000	4.106735000
C	9.403106000	8.322666000	5.841298000	C	8.280654000	7.713302000	6.848184000
C	4.771690000	9.550444000	4.108638000	C	5.272569000	9.490757000	3.166128000
C	8.987598000	9.611277000	5.942639000	C	8.646371000	8.743124000	6.041205000
C	6.202106000	6.939431000	2.645722000	C	4.673734000	6.142556000	4.215665000
C	7.577351000	7.786256000	4.362771000	C	6.408359000	7.182030000	5.429308000
C	7.819378000	10.036551000	5.268789000	C	7.885652000	9.046236000	4.889902000
C	7.095916000	9.135628000	4.500818000	C	6.761494000	8.284178000	4.580130000
C	2.722503000	9.331482000	5.176872000	C	3.840470000	11.312363000	3.270686000
H	2.241716000	9.763642000	6.053335000	H	3.929133000	12.368916000	3.518765000
H	2.339642000	8.312442000	5.036026000	H	2.832150000	10.972295000	3.537201000
C	2.542519000	10.169730000	3.917527000	C	4.156803000	11.009145000	1.811564000
H	1.674864000	9.875615000	3.328252000	H	3.271893000	11.001795000	1.176114000
H	2.455160000	11.236859000	4.158532000	H	4.876371000	11.727154000	1.398795000
C	4.743059000	8.482918000	6.370327000	C	4.663720000	10.359228000	5.412907000
H	4.706393000	7.419245000	6.110357000	H	3.859879000	9.649994000	5.642673000
H	4.168795000	8.634702000	7.283285000	H	4.401148000	11.328308000	5.834123000
H	5.772305000	8.761098000	6.571387000	H	5.578397000	10.022709000	5.890346000
C	7.665985000	8.360362000	1.219489000	C	6.572920000	5.413739000	2.791598000
H	7.484015000	9.431504000	1.322622000	H	6.967260000	6.302447000	2.293555000
H	7.990756000	8.148113000	0.201284000	H	6.701462000	4.544705000	2.147160000
H	8.470212000	8.075580000	1.891742000	H	7.134574000	5.245173000	3.708071000
C	5.432262000	7.394763000	0.508195000	C	4.137922000	5.458645000	2.077890000
H	5.826159000	6.802915000	-0.322675000	H	4.211914000	4.513342000	1.542541000
H	5.078802000	8.346815000	0.111496000	H	4.234299000	6.279467000	1.360035000
C	4.110074000	10.675510000	2.027376000	C	5.371400000	9.098159000	0.738018000
H	4.152336000	11.748725000	2.247319000	H	6.164720000	9.740276000	0.337041000
H	3.354788000	10.505423000	1.259663000	H	4.619508000	8.950192000	-0.037652000
H	5.082954000	10.363242000	1.659421000	H	5.804990000	8.139959000	1.008586000
C	4.339956000	5.631669000	3.611745000	C	2.449214000	6.587921000	5.200245000
C	4.343768000	6.646343000	1.294779000	C	2.852995000	5.580589000	2.905584000
H	3.499269000	7.298203000	1.539381000	H	2.082959000	6.167690000	2.407235000
H	3.966760000	5.772046000	0.764437000	H	2.435084000	4.600407000	3.160487000
H	3.922043000	4.678286000	3.286404000	H	1.688049000	7.293486000	4.863748000
H	3.521565000	6.263806000	3.974001000	H	3.034791000	7.038049000	5.997206000
H	5.042970000	5.453281000	4.420970000	H	1.951143000	5.694339000	5.589251000
H	9.007460000	6.501312000	4.964272000	H	6.904309000	6.204478000	7.119072000
C	8.543058000	11.794701000	3.132758000	C	10.751940000	10.018780000	3.563587000
C	7.398989000	11.474725000	5.353173000	C	8.265648000	10.207269000	3.995733000
C	9.187544000	12.600807000	2.054231000	C	12.032305000	10.445635000	3.696506000
C	7.993775000	12.338395000	4.249029000	C	9.666701000	10.708885000	4.161987000
C	9.047309000	14.072665000	2.272749000	C	12.384469000	11.711850000	4.408948000
C	7.956750000	13.748839000	4.407434000	C	9.953650000	11.826304000	4.982044000
C	8.477631000	14.584584000	3.397501000	C	11.220595000	12.287672000	5.149408000
H	9.448006000	14.732950000	1.513132000	H	9.135008000	12.320743000	5.494640000
H	7.525609000	14.181428000	5.300833000	H	9.660336000	15.598556000	1.783467000
H	8.422685000	15.658651000	3.527461000	H	11.291490000	14.887972000	3.486445000
H	10.911320000	8.507920000	-0.936243000	C	10.280220000	14.729068000	1.604358000
H	10.593891000	10.909584000	-0.489976000	C	11.202172000	14.328523000	2.563257000
C	10.977574000	8.878747000	0.078891000	C	10.165160000	14.024074000	0.411710000
C	10.799184000	10.233735000	0.332321000	H	9.457081000	14.341938000	-0.343052000
C	11.261876000	8.004221000	1.122329000	C	12.023987000	13.221477000	2.351673000
H	11.417045000	6.951497000	0.924085000	C	12.991311000	12.769472000	3.410051000
C	10.907918000	10.743854000	1.627231000	C	10.982199000	12.922209000	0.185020000
C	10.700242000	12.208380000	1.899059000	C	11.902131000	12.526795000	1.148140000
C	11.363723000	8.497817000	2.418333000	H	10.912680000	12.377241000	-0.748109000
C	11.188505000	9.854332000	2.665851000	H	12.540615000	11.672431000	0.959911000
H	11.602895000	7.828685000	3.235791000	H	8.802949000	7.430677000	7.748925000
H	11.280629000	10.233318000	3.676174000	H	9.518954000	9.331172000	6.280716000
H	10.272571000	7.921405000	6.338174000	H	7.554561000	11.021276000	4.172198000
H	9.549162000	10.309187000	6.546151000	H	8.093474000	9.891733000	2.961846000
H	7.709246000	11.882767000	6.317627000	H	10.545547000	9.121605000	2.989355000
H	6.310279000	11.549100000	5.315959000	H	12.839762000	9.896737000	3.226838000
H	11.119404000	12.804377000	1.085760000	H	13.331483000	13.623557000	3.999099000
H	11.216534000	12.497703000	2.815468000	H	13.875032000	12.321855000	2.950590000
H	8.573022000	10.722893000	3.013580000	H	13.202160000	11.511389000	5.116669000
H	8.727233000	12.335738000	1.087834000	H	11.407488000	13.143051000	5.787284000
